# Supplementary material for: Blockchain-based isotopic big data-driven tracing of global PM sources and interventions
Source: Nat Commun. 2025 Apr 25;16:3901. doi: 10.1038/s41467-025-59220-4 (PMC12022126; doi:10.1038/s41467-025-59220-4)
Supplement: Supplementary file 1 — Supplementary Information [file 41467_2025_59220_MOESM1_ESM.pdf]

SUPPLEMENTARY INFORMATION

for

Blockchain-based isotopic big data-driven tracing of global PM sources and interventions

Yuming Huang[1,2], Xiangyu Li[1], Yuehan Wu[1], Chaoyang Xue[3], Jiashuo Li[4], Yongfeng Lin[1], Wei Nie[5], Xian Liu[1], Qian Liu[1], Greg Michalski[6], Jingwei Zhang[7,*], Zheng Zong[8,*], Dawei Lu[1,9*], Guibin Jiang[1]

[1] Key Laboratory of Environmental Chemistry and Toxicology, Research Center for Eco-Environmental Sciences, Chinese Academy of Sciences, Beijing, 100085, China

[2] Sino-Danish College, Sino-Danish Center for Education and Research, University of Chinese Academy of Sciences, Beijing 100049, China

[3] Max Planck Institute for Chemistry, Mainz 55128, Germany

[4] Institute of Blue and Green Development, Shandong University, Weihai, 264209, China.

[5] Joint International Research Laboratory of Atmospheric and Earth System Research, School of Atmospheric Sciences, Nanjing University, Nanjing, China

[6] Department of Earth, Atmospheric, and Planetary Sciences, Purdue University, 550 Stadium Mall Drive, West Lafayette, IN 47907, USA.

[7] Yunnan Key Laboratory of Meteorological Disasters and Climate Resources in the Greater Mekong Subregion, Yunnan University, Kunming, 650500, China

[8] Environment Research Institute, Shandong University, Qingdao, Shandong, 266237, China

[9] Hubei Key Laboratory of Environmental and Health Effects of Persistent Toxic Substances, School of Environment and Health, Jianghan University, Wuhan, 430056, China

Correspondence: Correspondence and requests for materials should be addressed to Jingwei Zhang (jwzhang@ynu.edu.cn), Zheng Zong (zzong@sdu.edu.cn), or Dawei Lu (dwlu@rcees.ac.cn).

Contents

1. Supplementary Note 1–4
2. Supplementary Figures S1–S15
3. Supplementary Tables S1–S5
4. References for SI

1. Supplementary discussion

Supplementary Note 1: Regional characteristics of isotopic fingerprints of PM emissions.

The formal bootstrap testing was used to characterize the global source isotopic variations. As shown in Supplementary Fig. 4, the source isotopic signatures show no significant latitude/longitude dependence within these three regions. This might be attributed to the fact that most anthropogenic point sources are randomly scattered within the regions. Thus, by combining the source differentiation capabilities of multidimensional isotopic fingerprints with regional isotopic heterogeneity, the IDGAR supports the feasibility of tracing PM and its main emissions sources at global and regional scales.

Supplementary Note 2: Temporal dynamics of global PM isotopic fingerprints.

The δ^{30}Si noticeably increased before 2016 (Supplementary Fig. 5e & f), suggesting that the main sources of Si-bearing PM shifted from isotope-depleted emissions to soil-related emissions, with the isotopic fingerprints approaching zero (Fig. 2f). In contrast,δ^{56}Fe has continuously decreased from 2004 to 2020 (Supplementary Fig. 5i & j), suggesting the dominance of isotope-enriched emissions from sources such as coal combustion and biomass burning, which should be the focus of interventions for controlling Fe-bearing PM.

The global trend of δ^{65}Cu reversed from an increase to a decrease in 2011 (Supplementary Fig. 5k), suggesting a shift in the main sources of Cu-bearing PM from isotope-enriched to isotope-depleted emissions (e.g., coal combustion). Yet, the rate of change in δ^{65}Cu show no significant continuous trend of change (Supplementary Fig. 5l). The δ^{144}Nd did not show statistically significant changes in recent years (Supplementary Fig. 5q & r), indicating stable or homogeneous source compositions.

The δ^{66}Zn values of global PM fluctuated, first increasing (2002–2009), then decreasing (2010–2012), and then increasing again (2013–2015), suggesting a shift in the major emissions of Zn-bearing PM from isotope-enriched sources (biomass burning or coal combustion) to isotope-depleted sources (vehicle emissions or soil-related emissions) and then back to isotope-enriched sources (Supplementary Fig. 5m & n, Fig. 1j).

There were two turning points in the global trend of δ^{87}Sr values in 2005 and 2011 (Supplementary Fig. 5o). The decline in δ^{87}Sr from 2001 to 2005 and from 2011 to the present

day indicates that Sr-bearing PM was dominated by isotope-depleted emissions during these periods. The increase in δ^{87}Sr from 2005 to 2011 indicates that Sr-bearing PM mainly originated from isotope-enriched emissions during this period. Additionally, the continuous decrease in the declining rates of δ^{87}Sr from 2015 to 2018 (Supplementary Fig. 5p) indicates the effectiveness of interventions targeting Sr-depleted emissions, e.g., non-exhaust emissions and ore-related emissions (Supplementary Fig. 9).

Considering the source Pb isotopic signature show notable regional difference, we analyzed the temporal dynamics of Pb in PM across Asia, the Americas, and Europe. Compared to Europe and the Americas, the temporal trends and rates of change in Pb isotopic fingerprints in Asian PM show significant dynamic characteristics. For instance, the ^{207}Pb/^{206}Pb of Asian PM tended to decrease from 2001 to 2008, and from 2018 to the present day (Supplementary Fig. 6), indicating that Pb-bearing PM was mainly emitted from isotope-depleted sources, e.g., coal combustion and brake pads with ^{207}Pb/^{206}Pb < 0.84. From 2008 to 2018, the ^{207}Pb/^{206}Pb of Asian PM increased, with an overall continuous decrease in the rate of change from 2011 to 2017 (Fig. 3f). This suggests that Pb-bearing PM was dominated by isotope-enriched sources, e.g., tires, waste incinerator, diesel/gasoline combustion, and that interventions during 2011–2017 were effective (Supplementary Fig. 9).

The δ^{202}Hg in PM decreased from 2007 to 2014 (Supplementary Fig. 5s), indicating that isotope-depleted emissions were the major source of Hg-bearing PM during this period. From 2010 to 2014, the decreasing rates of δ^{202}Hg gradually slowed (Supplementary Fig. 5t), suggesting the effectiveness of interventions targeting Hg-bearing PM from sources such as coal combustion and biomass burning (Supplementary Fig. 9, Supplementary Fig. 12). Identifying the main sources and assessing the effectiveness of interventions for PM species over past years is crucial for developing future PM pollution control strategies.

Supplementary Note 3: Details of isotope fractionation correction in using MixSIAR.

Isotope fractionation during the formation of key secondary components in $PM_{2.5}$ can result in isotopic shifts, which may impact the accuracy of the apportionment results. Here, we have corrected the isotopic fractionation in the tracing model using the empirical methods

documented in previous research. For NH_4^+, isotopic fractionation was quantified using a widely accepted method for particulate NH_4^+ [1,2]:

$$\delta^{15}\text{N-NH}_3 = \delta^{15}\text{N-NH}_4^+ - \varepsilon_{\text{NH4+/NH3}} \times (1 - f) \qquad (1)$$

where, δ^{15}N-NH_3 and δ^{15}N-NH_4^+ represent the isotopic values of gaseous NH_3 and its converted particulate NH_4^+, respectively. $\varepsilon_{\text{NH4+/NH3}}$ denotes the nitrogen equilibrium isotope fractionation factor, calculated as (12.4678×1000/T) − 7.6694, where T represents the ambient temperature in Kelvin. The parameter f represents the molar conversion ratio, defined as $[NH_4^+/(NH_4^+ + NH_3)]$. The f values has been reported to show regional and temporal variations on a global scale[3]. In our study, we selected appropriate f values tailored to each sampling location, considering both geographic and temporal contexts.

For NO_3^-, the isotope fractionation of NO_3^- (Δ^{15}N) during its formation was quantified by accounting for the combined effects of two primary pathways involving O_3 and •OH[4]:

$$\Delta^{15}\text{N} = \gamma \times \Delta(\delta^{15}\text{N-NO}_3^-)_{\text{OH}} + (1 - \gamma) \times (\delta^{15}\text{N-NO}_3^-)_{\text{O3}} \qquad (2)$$

where, γ represents the contribution ratio of the •OH pathway. Isotopic fractionation occurs during the reaction between NO_2 and photochemically produced •OH, denoted as $\Delta(\delta^{15}\text{N-NO}_3^-)_{\text{OH}}$. The remaining fractionation is attributed to the hydrolysis of N_2O_5, expressed as $\Delta(\delta^{15}\text{N-NO}_3^-)_{\text{O3}}$. Given that the NO_3^+ hydrocarbon (HC) pathway is a minor contributor on a global scale[5], its influence on fractionation was ignored. Moreover, our previous research demonstrated a significant linear relationship between γ and latitude[6]. In this study, γ was estimated based on the latitude of each sampling location. In addition, the $\Delta(\delta^{15}\text{N-NO}_3^-)_{\text{OH}}$ can be calculated using a mass-balance formula as follow[4]:

$$\triangle\,(\delta^{15}\text{N-NO}_3{}^-)_{\text{OH}} = 1000 \times \left[\frac{({}^{15}\alpha_{\text{NO}_2/\text{NO}} - 1)(1 - f_{\text{NO}_2})}{(1 - f_{NO2}) + ({}^{15}\alpha_{\text{NO}_2/\text{NO}} \times f_{\text{NO}_2})}\right] \qquad (3)$$

where ${}^{15}\alpha_{\text{NO2/NO}}$ is the equilibrium isotope fractionation factor between NO_2 and NO, which is a temperature-dependent function (refer to equation 5). The parameter f_{NO2} denotes the fraction of NO_2 in total NO_x with reported values ranging from 0.2 to 0.95[4]. Similarly, $\Delta(\delta^{15}\text{N-NO}_3^-)_{\text{O3}}$ can be determined from the following equation:

$$\triangle\,(\delta^{15}\text{N-NO}_3{}^-)_{\text{O3}} = 1000 \times \left(15\alpha_{\text{N}_2\text{O}_5/\text{NO}_2} - 1\right) \qquad (4)$$

where $^{15}\alpha_{N2O5/NO2}$ refers to the equilibrium isotope fractionation factor between N_2O_5 and NO_2, which is also a temperature-dependent function (see equation 5). For the $^{15}\alpha_{NO2/NO}$ and $^{15}\alpha_{N2O5/NO2}$ in these equations, the $^{m}\alpha_{X/Y}$ is a function of temperature, and can be expressed as:

$$1000(^{m}\alpha_{\mathrm{X/Y}} - 1) = \frac{A}{T^4} \times 10^{10} + \frac{B}{T^3} \times 10^{8} + \frac{C}{T^2} \times 10^{6} + \frac{D}{T} \times 10^{4} \tag{5}$$

where A, B, C, and D are experimental constants over the temperature range of 150-450 K. For a comprehensive and detailed discussion of these isotopic fractionations, you can find the information in the papers by Zong et al.[4] and Walters et al.[7,8].

To minimize model uncertainty arising from variations in the f_{NO2} value, an iterative approach was applied, using a simulation step of 0.01 times $\Delta^{15}N$. The results indicated that when the model used 0.66 times $\Delta^{15}N$, the probability distribution of source contributions exhibited the lowest variance. This value was therefore identified as the most likely solution.

For SO_4^{2-}, previous research has identified a strong linear relationship between the isotope fractionation factor and ambient temperature during SO_4^{2-} formation[9]. In this study, we utilized this relationship to quantify isotopic fractionation characteristics associated with SO_4^{2-} production. By combing recorded ambient temperatures for each sampling campaign with a Rayleigh distillation model, we calculated the isotopic composition of precursor to particulate SO_4^{2-}:

$$\delta^{34}\mathrm{SO}_2 = \delta^{34}\mathrm{SO}_4^{2-} \times \left(\frac{1-f}{f^{34\alpha Sg\rightarrow p}-1}\right) \tag{6}$$

where $\delta^{34}S\text{-}SO_2$ and $\delta^{34}S\text{-}SO_4^{2-}$ represent the isotopic values of gaseous SO_2 and its resulting particulate SO_4^{2-}, respectively. The term $\alpha^{34}S_{g\rightarrow p}$ refers to the isotope fractionation factor between gaseous SO_2 and particulate SO_4^{2-}, while f denotes the fraction of SO_2 remaining in gas phase, with reported values ranging from 0.1 to 0.9[10]. To further reduce uncertainties associated with f, we employed an iterative modelling approach. Our results revealed that when the model used 0.51 times isotope fractionation, the probability distribution of source contributions was more reasonable.

For OC, whose $\delta^{13}C$ isotopic fractionation effect is corrected using $f_{M\text{-}14C}$ (fractions of modern carbon, the isotopic characteristics of ^{14}C)[11,12]. The theoretical value of $\delta^{13}C$ in OC is primarily calculated through the mass balance of ^{14}C, and the correction factor is obtained by

comparing this with the observed δ^{13}C value in OC. The specific calculation formula is as follows:

$$f_{\mathrm{nf}} \times \delta^{13}\mathrm{C}_{\mathrm{nf}} + f_{\mathrm{coal}} \times \delta^{13}\mathrm{C}_{\mathrm{coal}} + f_{\mathrm{liq.fossil}} \times \delta^{13}\mathrm{C}_{\mathrm{liq.fossil}} = \delta^{13}\mathrm{C}_{\mathrm{sample}} + \beta \quad (7)$$

where $\delta^{13}C_{nf}$, $\delta^{13}C_{coal}$, $\delta^{13}C_{liq.fossil}$, and $\delta^{13}C_{sample}$ represent the δ^{13}C values for non-fossil sources, coal, liquid fossil fuels, and the OC sample, respectively. The f_{nf}, f_{coal}, and $f_{liq.fossil}$ denote the contributions of non-fossil fuels, coal, and liquid fossil fuels, respectively. The specific calculation process for these factors can be referenced in these studies[13-15]. The obtained β under different conditions will be incorporated into the tracing model to implement the correction of OC isotopic fractionation effects.

Supplementary Note 4: Comparison of MixSIAR, MixSIR, and FRUITS.

To compare the model performance, we selected six $PM_{2.5}$ pollution events that reported the isotopic compositions of EC for both ^{14}C ($f_{M\text{-}14C}$) and ^{13}C (δ^{13}C). Using these data, we calculated the relative contributions of individual sources to EC based on δ^{13}C values with MixSIAR, MixSIR, and FRUITS. Then, based on the δ^{13}C -based contributions, we calculated the $f_{M\text{-}14C}$ of EC in $PM_{2.5}$ and compared these with the observed $f_{M\text{-}14C}$ values. As shown in Supplementary Fig. 13, the $f_{M\text{-}14C}$ values calculated with MixSIAR show a smaller difference from the observed values compared to those from MixSIR and FRUITS, supporting the robustness of the MixSIAR model. All three models are Bayesian-based and suitable for isotopic tracing analysis[16]. Among these, MixSIAR combines the MixSIR and SIAR models. Its advantage is that it incorporates both fixed and random effects as covariates, enabling the explanation of variability in mixing proportions. MixSIR is implemented using the MATLAB language. Its advantage lies in its ability to account for multi-source isotopic characteristics and isotopic discrimination, though it falls short in fully addressing data uncertainty. FRUITS is primarily utilized for analyzing food source contributions. Its advantage lies in its ability to correct isotopic pathways for proteins, fats, and other substances. However, when applied to $PM_{2.5}$ tracing, this may necessitate further optimization.

2. Supplementary figures

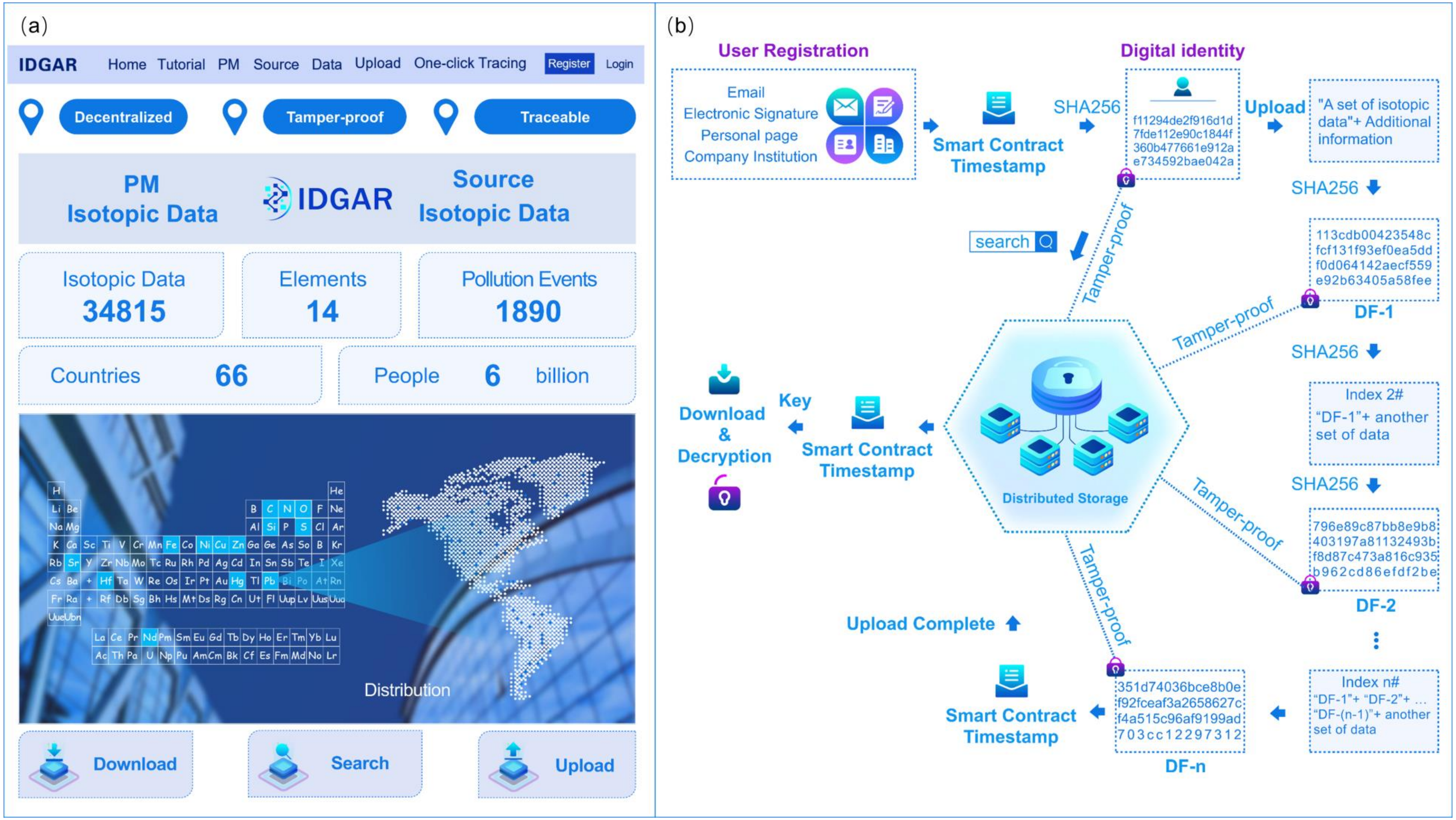

Supplementary Figure 1. IDGAR online portal and blockchain operational principles. a) Screenshot of the Isotopic Database for Global

Atmospheric Research (IDGAR), illustrating its functionalities (e.g., isotopic data download/upload), decentralized storage, tamperproof characteristics, and data volume. To share these extensive isotopic data and enhance their use, we created an online portal for the IDGAR at http://idgar.org/. **b**) Workflow of the blockchain-based data management system in the IDGAR, with comprehensive operations such as authentication, data search, download, and upload. Blockchain technology and the SHA256 function were employed to convert input data into unique digital fingerprints (alphanumeric strings). A consortium chain with 34,815 interconnected blocks, each containing detailed isotopic data and related information, was integrated into the IDGAR. The curated data are stored and managed in a decentralized manner across five node users to deter potential attacks. Users are provided with unique digital identities, generated from their authentication information. Each new operation appends a block that records details such as smart contract information, precise timestamps, isotopic file contents, previous block hashes, and current hashes within an immutable digital fingerprint. The term "DF" represents digital fingerprints.

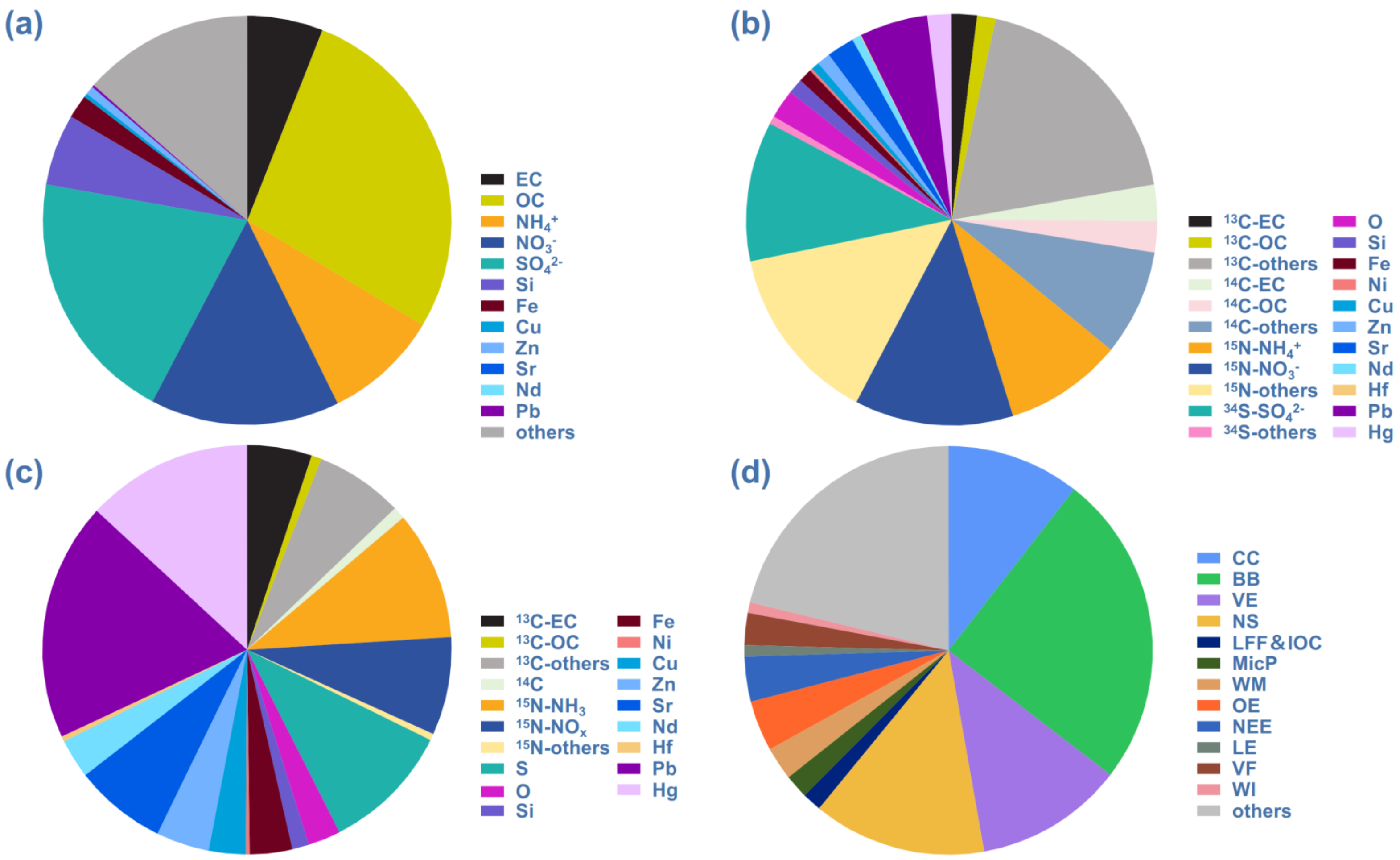
(a)
EC
OC
NH_4^+
NO_3^-
SO_4^{2-}
Si
Fe
Cu
Zn
Sr
Nd
Pb
others
(b)
^{13}C-EC
^{13}C-OC
^{13}C-others
^{14}C-EC
^{14}C-OC
^{14}C-others
$^{15}N-NH_4^+$
$^{15}N-NO_3^-$
^{15}N-others
$^{34}S-SO_4^{2-}$
^{34}S-others
O
Si
Fe
Ni
Cu
Zn
Sr
Nd
Hf
Pb
Hg
(c)
^{13}C-EC
^{13}C-OC
^{13}C-others
^{14}C
$^{15}N-NH_3$
$^{15}N-NO_x$
^{15}N-others
S
O
Si
Fe
Ni
Cu
Zn
Sr
Nd
Hf
Pb
Hg
(d)
CC
BB
VE
NS
LFF&IOC
MicP
WM
OE
NEE
LE
VF
WI
others

Supplementary Figure 2. A statistical distribution of the mass composition and amount of data for the curated isotopic species included in the IDGAR. a) Mass distribution of components containing these curated elements (e.g., EC, OC, nitrate, sulphate) in the known components of atmospheric particulate matter. **b & c**) Statistical distribution showing the count of isotope records from atmospheric particulate matter (**b**) and individual sources (**c**) within the IDGAR. A total of 27,762 atmospheric particulate matter isotope records and 7,053 source isotope records were collected. **d**) Statistical distribution of the number of isotope records in each individual source.

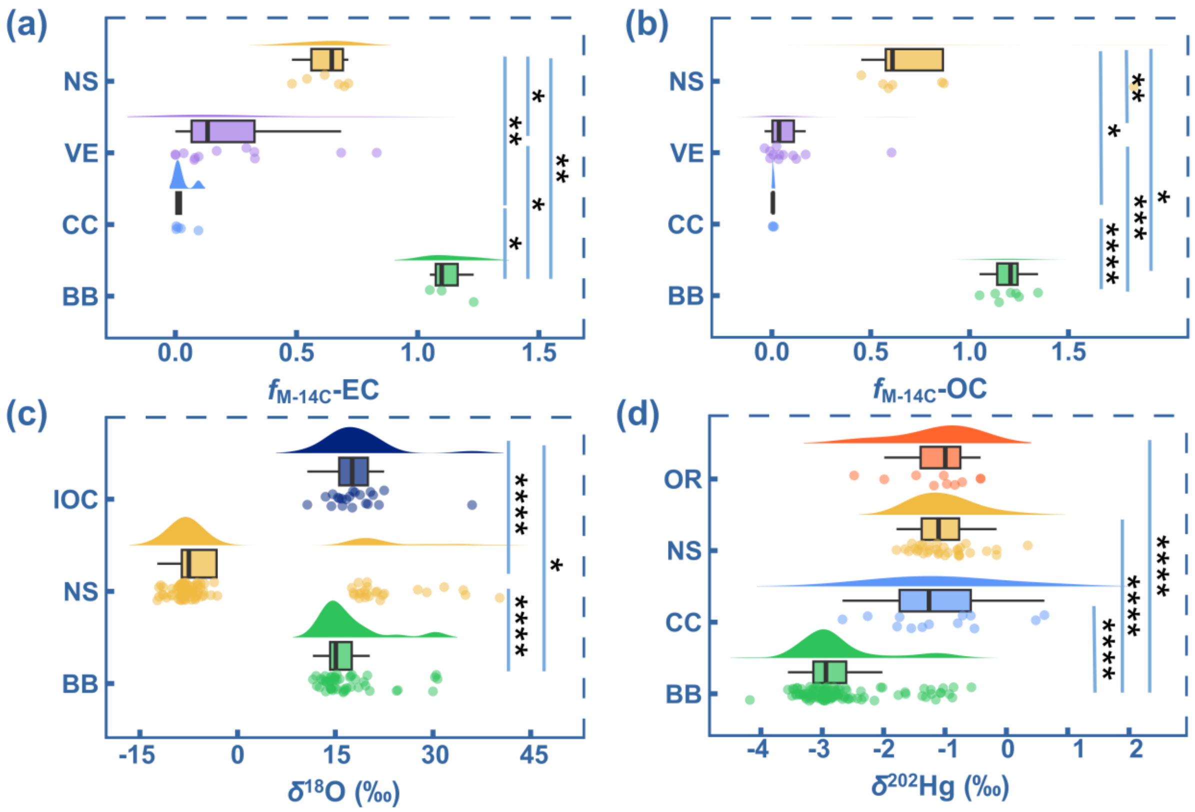

Supplementary Figure 3. Statistical variations in $f_{M\text{-}14C}$, $\delta^{18}O$, $\delta^{202}Hg$ across global atmospheric particulate matter (PM) sources. a-d) Statistical differences in isotopic fingerprints among different emissions of $f_{M\text{-}14C}$-EC, $f_{M\text{-}14C}$-OC, $\delta^{18}O$, and $\delta^{202}Hg$, respectively. The labels NS, VE, CC, BB, IOC, and OE represent natural soil, vehicle exhausts, coal combustion, biomass burning, industrial oil combustion, and ore-related emissions, respectively. The boxes span the interquartile range (25_{th} to 75_{th} percentile), with the central line marking the median, and the whiskers extending to the 5_{th} and 95_{th} percentiles. Statistical significance is denoted as follows: ****$P < 0.0001$, ***$P < 0.001$, **$P < 0.01$, *$P < 0.05$. The number of isotopic data points for each category is detailed in the Source Data file.

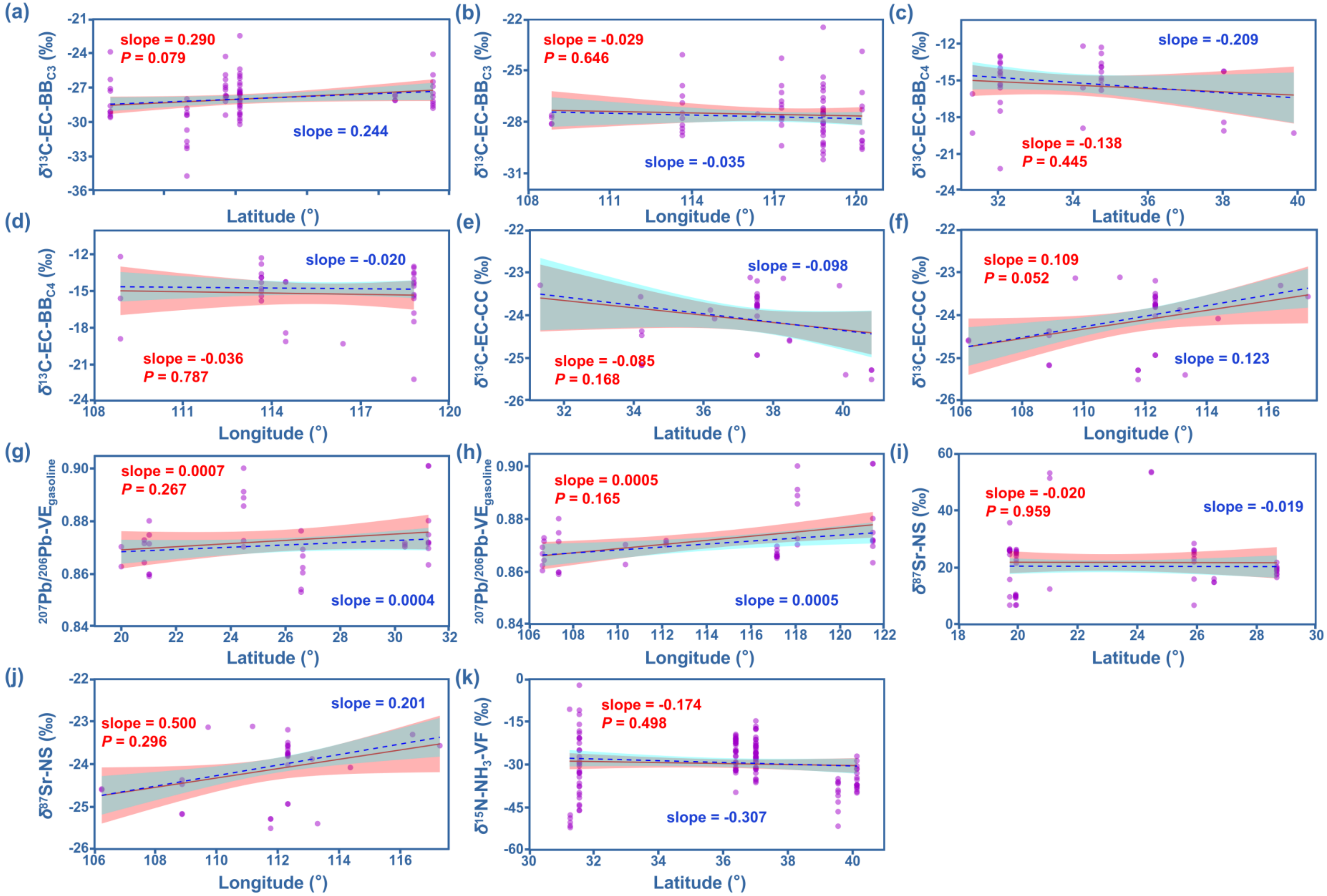
(a)
slope = 0.290
P = 0.079
slope = 0.244
δ13C-EC-BBC3 (‰)
Latitude (°)
(b)
slope = -0.029
P = 0.646
slope = -0.035
δ13C-EC-BBC3 (‰)
Longitude (°)
(c)
slope = -0.209
slope = -0.138
P = 0.445
δ13C-EC-BBC4 (‰)
Latitude (°)
(d)
slope = -0.020
slope = -0.036
P = 0.787
δ13C-EC-BBC4 (‰)
Longitude (°)
(e)
slope = -0.098
slope = -0.085
P = 0.168
δ13C-EC-CC (‰)
Latitude (°)
(f)
slope = 0.109
P = 0.052
slope = 0.123
δ13C-EC-CC (‰)
Longitude (°)
(g)
slope = 0.0007
P = 0.267
slope = 0.0004
207Pb/206Pb-VEgasoline
Latitude (°)
(h)
slope = 0.0005
P = 0.165
slope = 0.0005
207Pb/206Pb-VEgasoline
Longitude (°)
(i)
slope = -0.020
P = 0.959
slope = -0.019
δ87Sr-NS (‰)
Latitude (°)
(j)
slope = 0.201
slope = 0.500
P = 0.296
δ87Sr-NS (‰)
Longitude (°)
(k)
slope = -0.174
P = 0.498
slope = -0.307
δ15N-NH3-VF (‰)
Latitude (°)

Supplementary Figure 4. Dependence of source isotopic changes on latitude and longitude. a-k) The red segments represent linear fits obtained through formal bootstrap testing procedures. The blue statistical results were analyzed using the Theil–Sen estimator, which confirmed the robustness of the analysis from the bootstrap tests. The shading around the red and blue lines indicates their 95% confidence intervals. For further details, please refer to the ‘Methods’ section. The abbreviations BB_{C3}, BB_{C4}, CC, VE, NS, and VF correspond to C3 plants burning emissions, C4 plants burning emissions, coal combustion, vehicle exhausts, natural soil, and volatilized fertilizer, respectively.

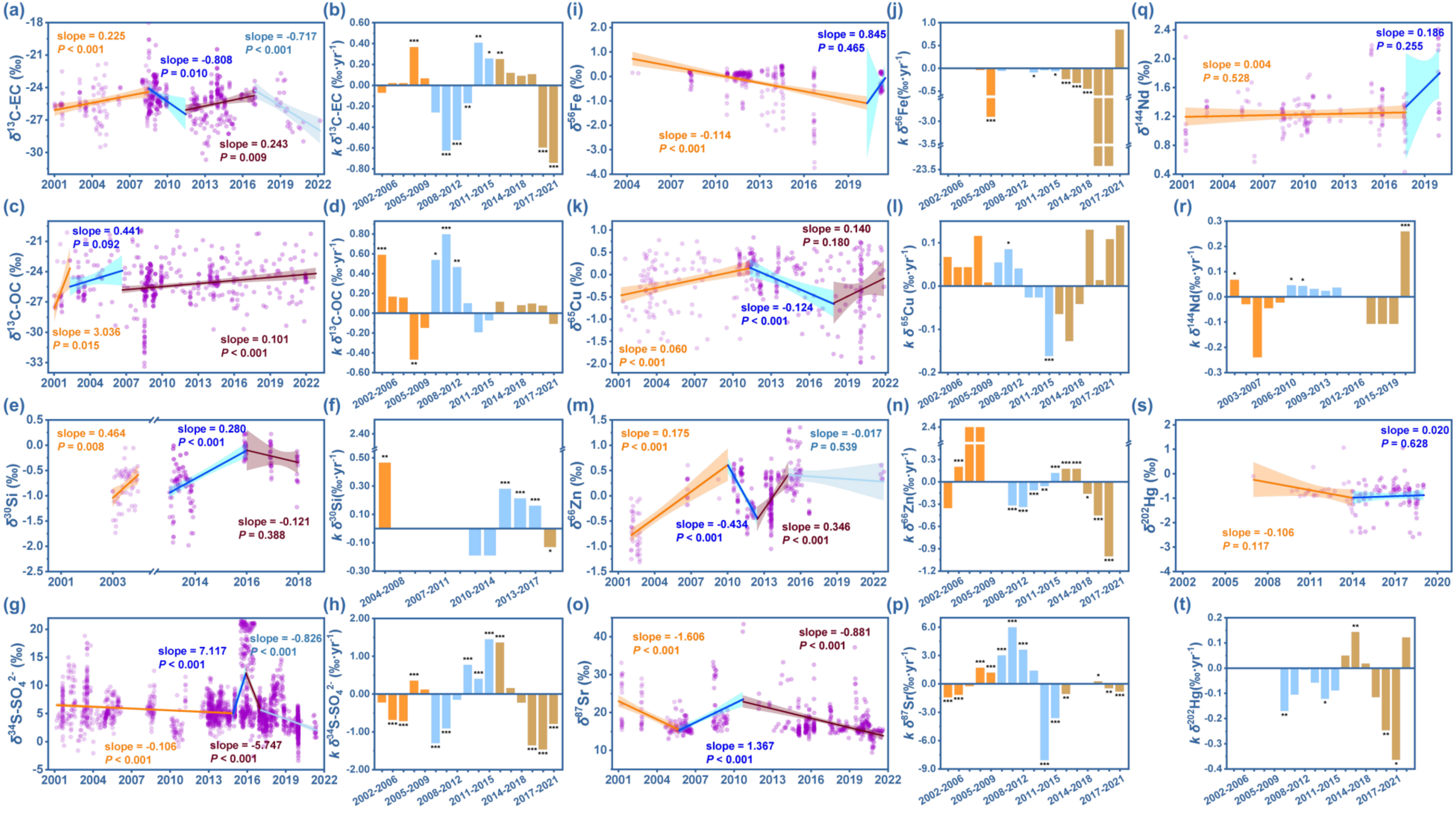

Supplementary Figure 5. Temporal tendencies of global atmospheric particulate matter (PM) isotopic fingerprints. The temporal trends of δ^{13}C -EC (**a**), δ^{13}C -OC (**c**), δ^{30}Si (**e**), δ^{34}S-SO_4^{2-} (**g**), δ^{56}Fe (**i**), δ^{65}Cu (**k**), δ^{66}Zn (**m**), δ^{87}Sr (**o**), δ^{144}Nd (**q**), and δ^{202}Hg (**s**) were analyzed using formal

bootstrap testing procedures. Moving subset window analysis of isotopic changes for δ^{13}C -EC (**b**), δ^{13}C -OC (**d**), δ^{30}Si (**f**), δ^{34}S-SO_4^{2-} (**h**), δ^{56}Fe (**j**), δ^{65}Cu (**l**), δ^{66}Zn (**n**), δ^{87}Sr (**p**), δ^{144}Nd (**r**), and δ^{202}Hg (**t**) is also presented. Each window includes a subset of PM isotopic data within a 5-year interval, with 1-year steps. The bars represent the isotopic change rates (*k*) over five years. The different-colored lines in (**a**), (**c**), (**e**), (**g**), (**i**), (**k**), (**m**), (**o**), (**q**), and (**s**) represent linear fits for different periods, with the shading indicating their 95% confidence intervals. The colour scale of the bars in **b**, **d**, **f**, **h**, **j**, **l**, **n**, **p**, **r**, and **t** correspond to temporal trends. The specific numbers of isotopic data points used for each moving window analysis are provided in the Source Data file. ***$P < 0.001$, **$P < 0.01$, *$P < 0.05$. The reliability of the statistical results was confirmed using the Theil–Sen estimator (Supplementary Fig. 7 & 8).

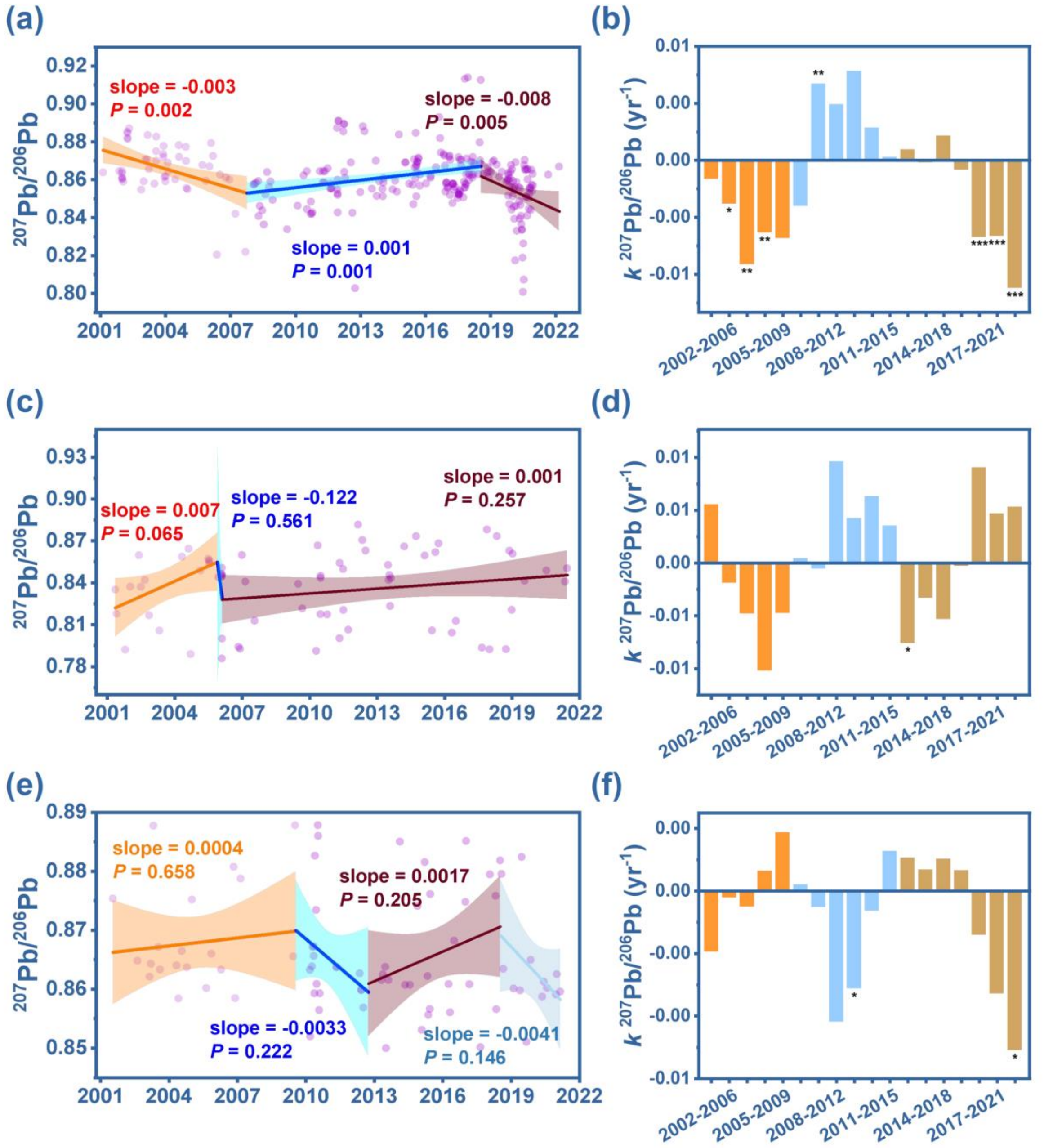

Supplementary Figure 6. Temporal tendencies of Pb isotopic fingerprints in atmospheric particulate matter (PM). The temporal trends of $^{207}Pb/^{206}Pb$ in PM from Asia **(a & b)**, the Americas **(c & d)**, and Europe **(e & f)** were analyzed using formal bootstrap testing procedures. Moving subset window analysis of isotopic changes for $^{207}Pb/^{206}Pb$ in PM is also presented. Each window includes a subset of PM isotopic data within a 5-year interval, with 1-year steps. The bars represent the isotopic change rates (*k*) over five years. The different color lines in **a**, **c**, and **e** correspond to the liner fits for different periods, with the shading indicating their 95% confidence intervals. The colour scale of the bars in **b**, **d**, and **f** correspond to temporal trends. The specific numbers of isotopic data points used for each moving window analysis are

provided in the Source Data file. ***$P < 0.001$, **$P < 0.01$, *$P < 0.05$. The robustness of the statistical results was verified using the Theil–Sen estimator (Supplementary Fig. 15).

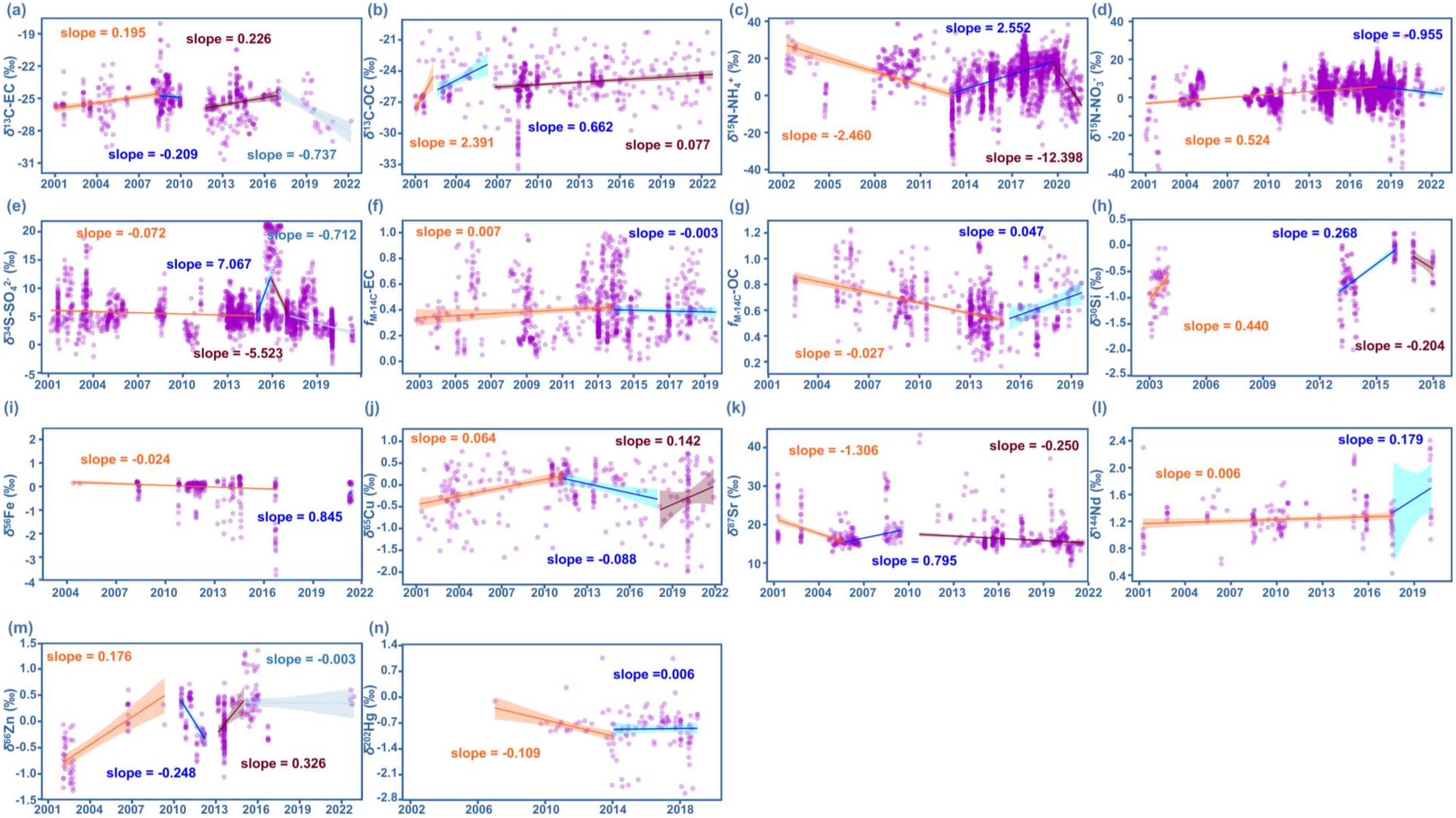

Supplementary Figure 7. Trend analyses of global atmospheric particulate matter (PM) isotopic fingerprints using the Theil-Sen estimator.

a-n) Temporal trends of δ^{13}C-EC (**a**), δ^{13}C-OC (**b**), δ^{15}N-NH_4^+ (**c**), δ^{15}N-NO_3^- (**d**), δ^{34}S-SO_4^{2-} (**e**), $f_{M\text{-}14C}$-EC (**f**), $f_{M\text{-}14C}$-OC (**g**), δ^{30}Si (**h**), δ^{56}Fe (**i**),

δ^{65}Cu (**j**), δ^{87}Sr (**k**), δ^{144}Nd (**l**), δ^{66}Zn (**m**), and δ^{202}Hg (**n**). The different color lines and shadings correspond to the liner fits for different periods and their 95% confidence intervals.

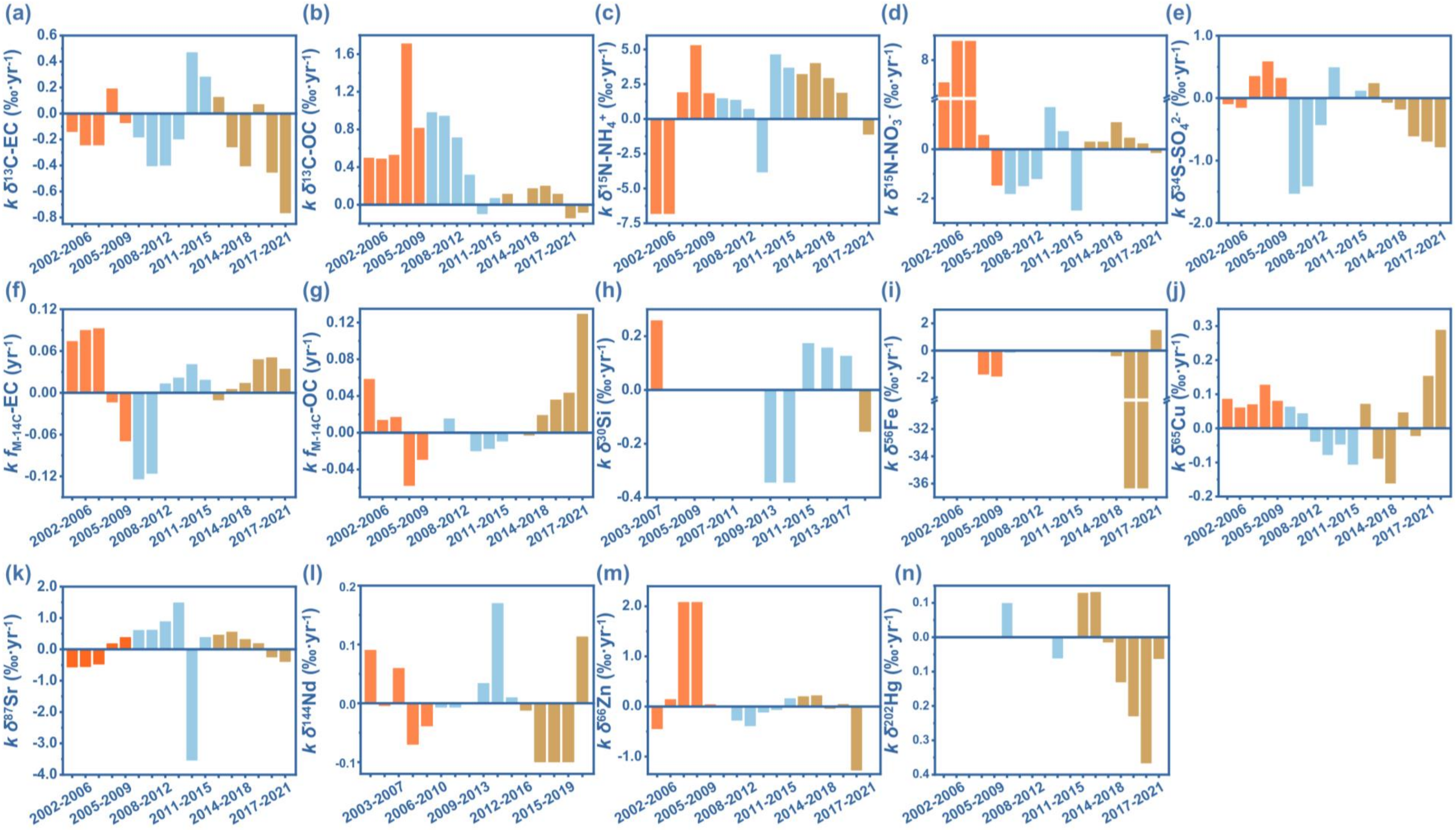
(a)
k δ13C-EC (‰·yr-1)
(b)
k δ13C-OC (‰·yr-1)
(c)
k δ15N-NH4+ (‰·yr-1)
(d)
k δ15N-NO3- (‰·yr-1)
(e)
k δ34S-SO42- (‰·yr-1)
(f)
k fM-14C-EC (yr-1)
(g)
k fM-14C-OC (yr-1)
(h)
k δ30Si (‰·yr-1)
(i)
k δ56Fe (‰·yr-1)
(j)
k δ65Cu (‰·yr-1)
(k)
k δ87Sr (‰·yr-1)
(l)
k δ144Nd (‰·yr-1)
(m)
k δ66Zn (‰·yr-1)
(n)
k δ202Hg (‰·yr-1)
2002-2006
2005-2009
2008-2012
2011-2015
2014-2018
2017-2021
2003-2007
2007-2011
2009-2013
2013-2017
2006-2010
2012-2016
2015-2019

Supplementary Figure 8. Moving window analyses of global atmospheric particulate matter (PM) isotopic fingerprints using the Theil-Sen estimator. a-n) Moving subset window analysis of the temporal change trends of δ^{13}C-EC (**a**), δ^{13}C-OC (**b**), δ^{15}N-NH_4^+ (**c**), δ^{15}N-NO_3^- (**d**), δ^{34}S-SO_4^{2-} (**e**), $f_{M\text{-}14C}$-EC (**f**), $f_{M\text{-}14C}$-OC (**g**), δ^{30}Si (**h**), δ^{56}Fe (**i**), δ^{65}Cu (**j**), δ^{87}Sr (**k**), δ^{144}Nd (**l**), δ^{66}Zn (**m**), and δ^{202}Hg (**n**). The bars indicate the rates (k) of isotopic changes per 5 years. Moreover, the subset window analysis moves forward by a 1-year step from 2001 to 2023. The color scale of the bars correspond to temporal trends.

239

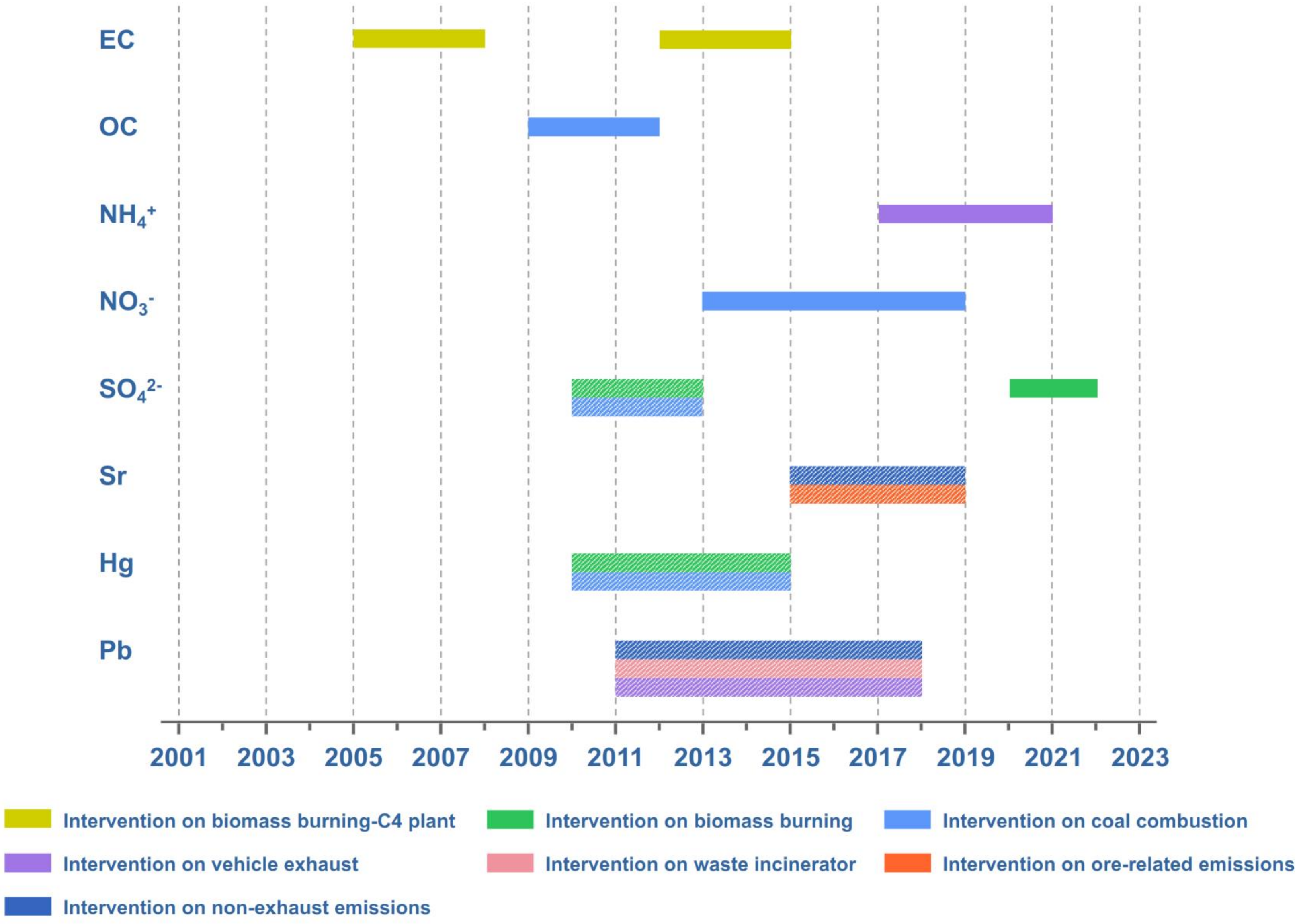
EC
OC
NH_4^+
NO_3^-
SO_4^{2-}
Sr
Hg
Pb
2001
2003
2005
2007
2009
2011
2013
2015
2017
2019
2021
2023
Intervention on biomass burning-C4 plant
Intervention on biomass burning
Intervention on coal combustion
Intervention on vehicle exhaust
Intervention on waste incinerator
Intervention on ore-related emissions
Intervention on non-exhaust emissions

Supplementary Figure 9. Identification of the effectiveness and precise timing of interventions for various atmospheric particulate matter (PM) species. The evaluation of intervention effectiveness and implementation timing primarily focuses on the main emission sources. The isotopic trends of different PM species enable to indicate the main sources of different PM species. The intervention effectiveness was assessed based on observing continuous slowdown in trending rates (i.e., the slope between isotopic fingerprints and year). The start and end periods of this continuous slowdown indicate the duration of the effective intervention. The solid area represents the effective interventions and their duration, while shaded areas suggest that one or both interventions were effective, likely due to overlapping isotopic compositions. *Note:* EC and OC can be used to distinguish whether they are emitted from the burning of C4 or C3 plants. Other PM components (e.g., sulfate) cannot be differentiated and are therefore generally referred to as biomass combustion.

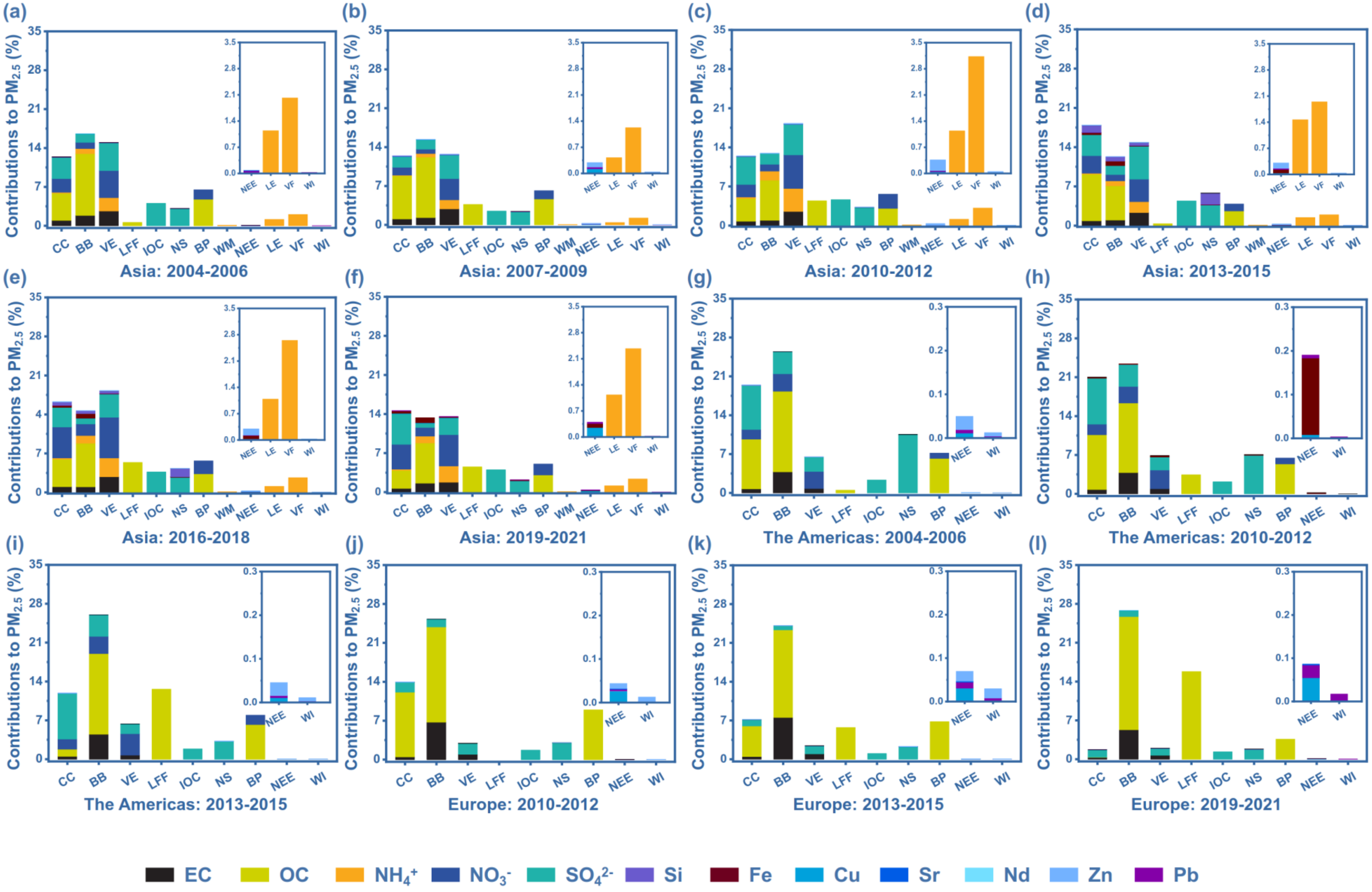

(a)
Asia: 2004-2006
(b)
Asia: 2007-2009
(c)
Asia: 2010-2012
(d)
Asia: 2013-2015
(e)
Asia: 2016-2018
(f)
Asia: 2019-2021
(g)
The Americas: 2004-2006
(h)
The Americas: 2010-2012
(i)
The Americas: 2013-2015
(j)
Europe: 2010-2012
(k)
Europe: 2013-2015
(l)
Europe: 2019-2021
Contributions to PM2.5 (%)
CC BB VE LFF IOC NS BP WM NEE LE VF WI
EC OC NH4+ NO3- SO42- Si Fe Cu Sr Nd Zn Pb

248

Supplementary Figure 10. Temporal changes of the source composition of atmospheric fine particulate matter ($PM_{2.5}$) and its components in Asia, the Americas, and Europe. Specifically, (**a-f**) Asia, (**g-i**) the Americas, and (**j-l**) Europe. The abbreviations CC, BB, VE, LFF, IOC, NS, BP, WM, NEE, LE, VF, and WI correspond to coal combustion, biomass burning, vehicle emissions, combustion of liquid fossil fuels, industrial oil combustion, natural soil, biological processes, waste materials, non-exhaust emissions, livestock emissions, volatilized fertilizer, and waste incineration. The uncertainties associated with these findings are detailed in the Source Data file, and the calculation process is outlined in the "Methods" section.

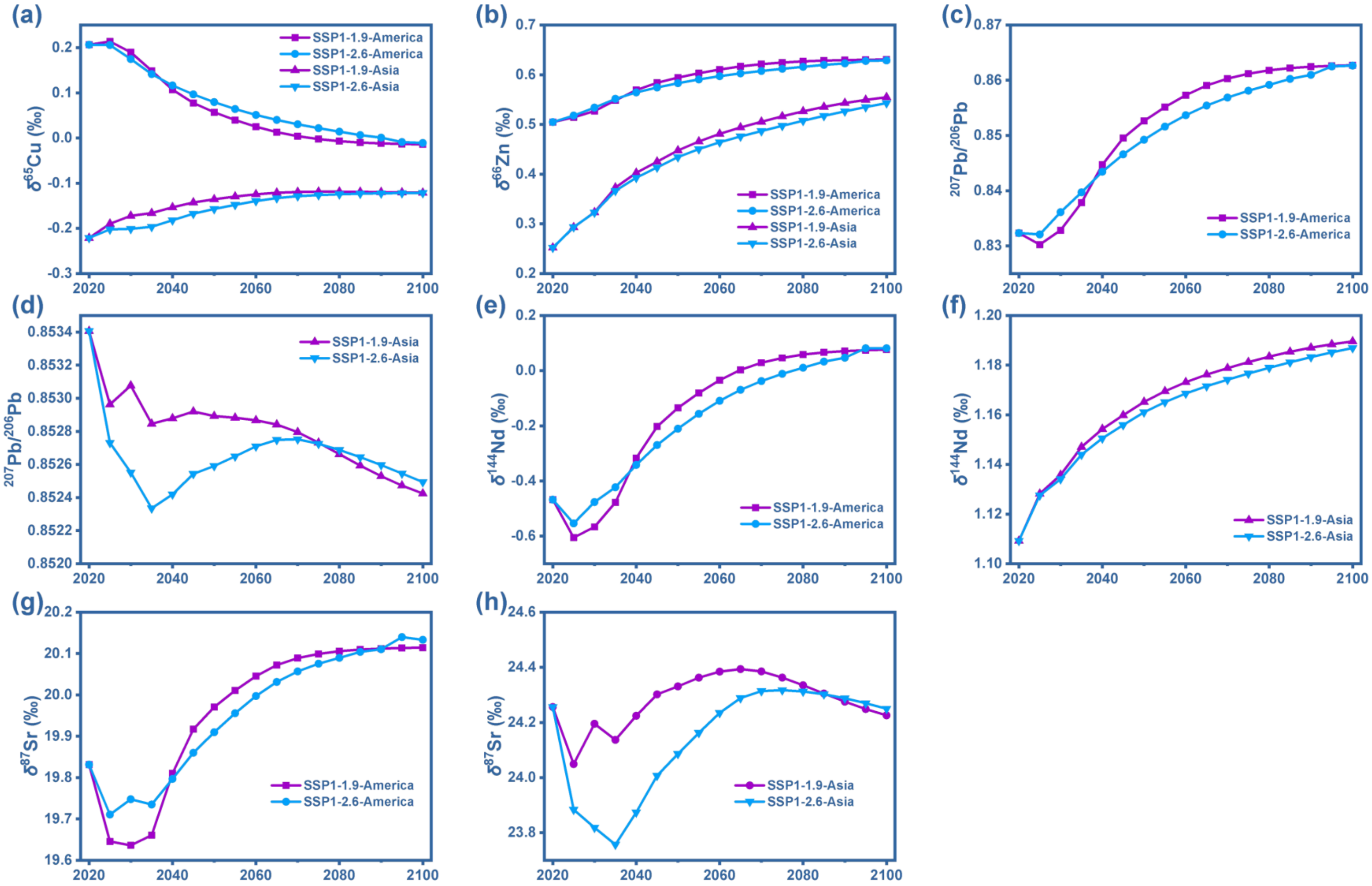

Supplementary Figure 11. Projected trends in the isotopic fingerprints of atmospheric fine particulate matter ($PM_{2.5}$) pollution in the

Americas and Asia through 2100. Specifically, **(a)** δ^{65}Cu, **(b)** δ^{66}Zn, **(c, d)** ^{207}Pb/^{206}Pb, **(e, f)** δ^{144}Nd, **(g, h)** δ^{87}Sr. The prediction was based on the established relationship between PM isotopes and source emissions, alongside future source emission scenarios projected by the global change assessment model (GCAM). The SSP1-1.9 and SSP1-2.6 scenarios, which integrate Shared Socioeconomic Pathways (SSPs) and Representative Concentration Pathways (RCPs), model the effects of future radiative forcing and climate impacts. These scenarios are designed to target global warming limits of 1.5°C and 2°C, respectively.

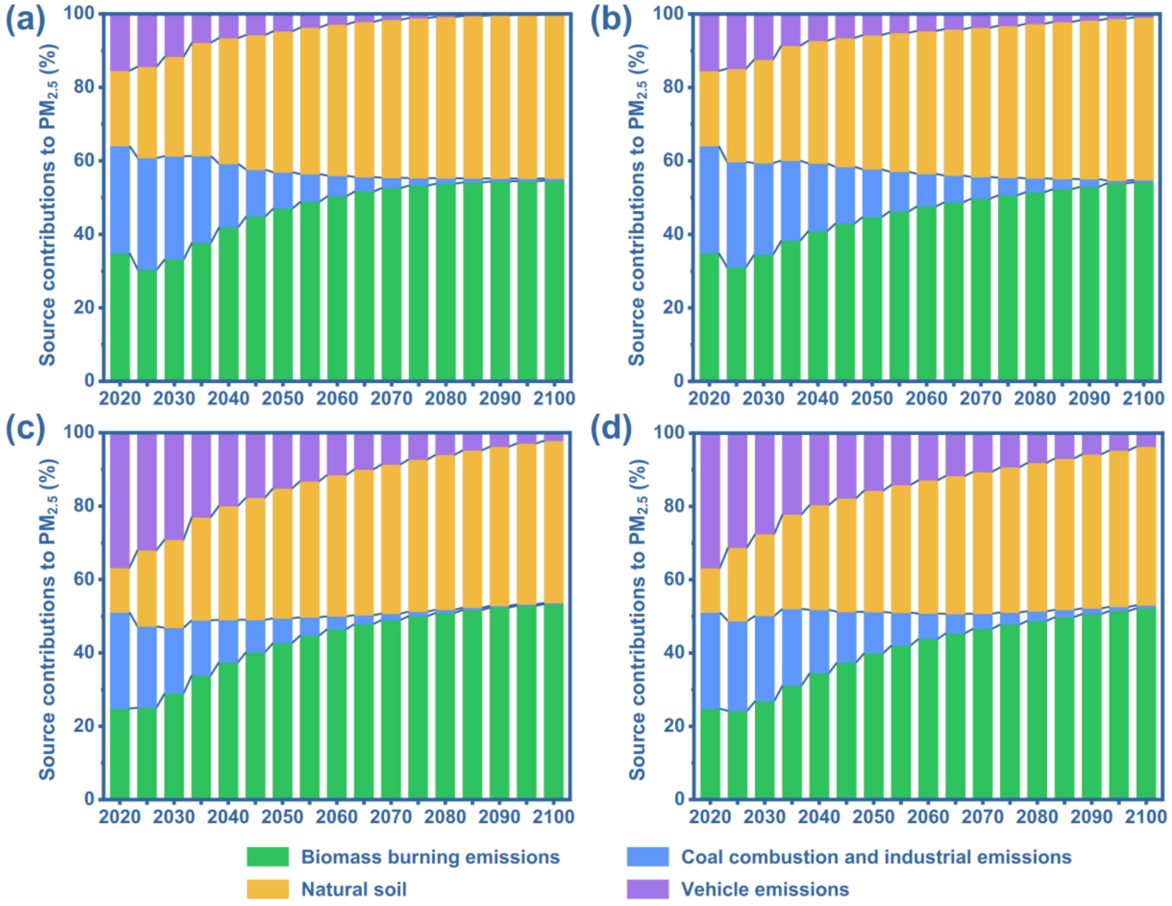

Supplementary Figure 12. Projected trends in the source composition of atmospheric fine particulate matter ($PM_{2.5}$) pollution in the Americas and Asia through 2100. **a & b**) The proportional contributions of various sources to $PM_{2.5}$ in the Americas in the SSP1-1.9 (**a**) and SSP1-2.6 (**b**) scenarios. **c & d**) The relative contributions of individual sources to $PM_{2.5}$ in Asia in SSP1-1.9 (**c**) and SSP1-2.6 (**d**) scenarios. The SSP1-1.9 and SSP1-2.6 scenarios combine Shared Socioeconomic Pathways (SSP) with Representative Concentration Pathways (RCP) to simulate future radiative forcing and climate effects. These models are tailored to achieve global warming thresholds of 1.5°C and 2°C, respectively. The emissions were projected by the global change assessment model (GCAM). Details on the uncertainties of these findings can be found in the Source Data File. *Note:* Given the current model's limitations, our analysis focuses on predicting the contributions of a few major source categories. With future improvements in data and models, more detailed analysis of subdivided sources is expected to be possible.

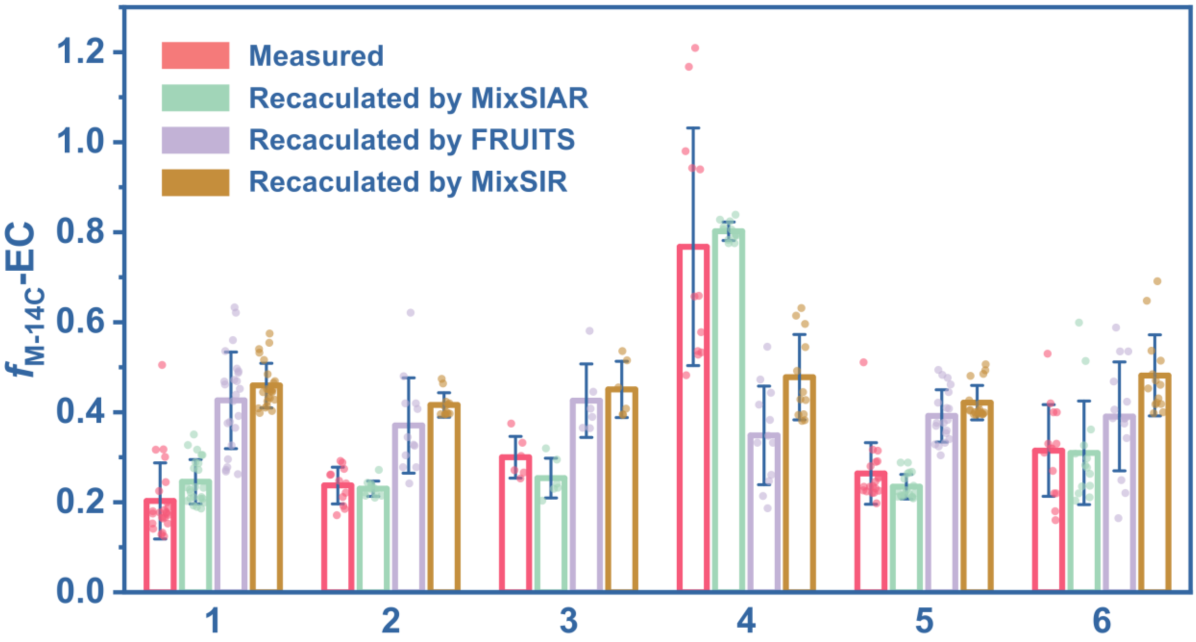

Supplementary Figure 13. Verification of EC source apportionment in atmospheric fine particulate matter ($PM_{2.5}$) using $f_{M\text{-}14C}$ and $\delta^{13}C$ dual isotopic fingerprints. The error bars for both the observed and recalculated results. For further details, refer to the 'Methods' section and Supplementary Note 4.

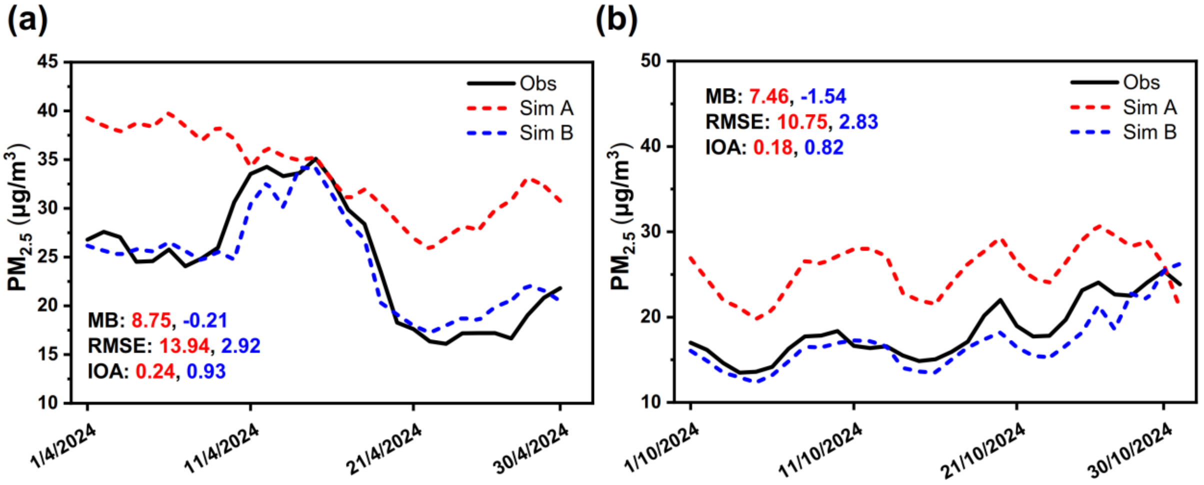

Supplementary Figure 14. Assessment of atmospheric fine particulate matter ($PM_{2.5}$) pollution level simulations. Daily mean concentrations of $PM_{2.5}$ for observation and simulation from April 1, 2024 to April 30, 2024 (**a**), from October 1, 2024 to October 30, 2024 (**b**) were used as a training set to assess the model. **a&b,** the dotted red lines was conducted without incorporating corrections from isotopic tracing results. The dotted blue lines represent the isotopic-guided prediction. The mean bias (MB), root mean square error (RMSE), and index of agreement (IOA) were used to evaluate the predictions against observations. The parameter results indicate that the isotopic-guided predictions are closer to real observations (solid black line).

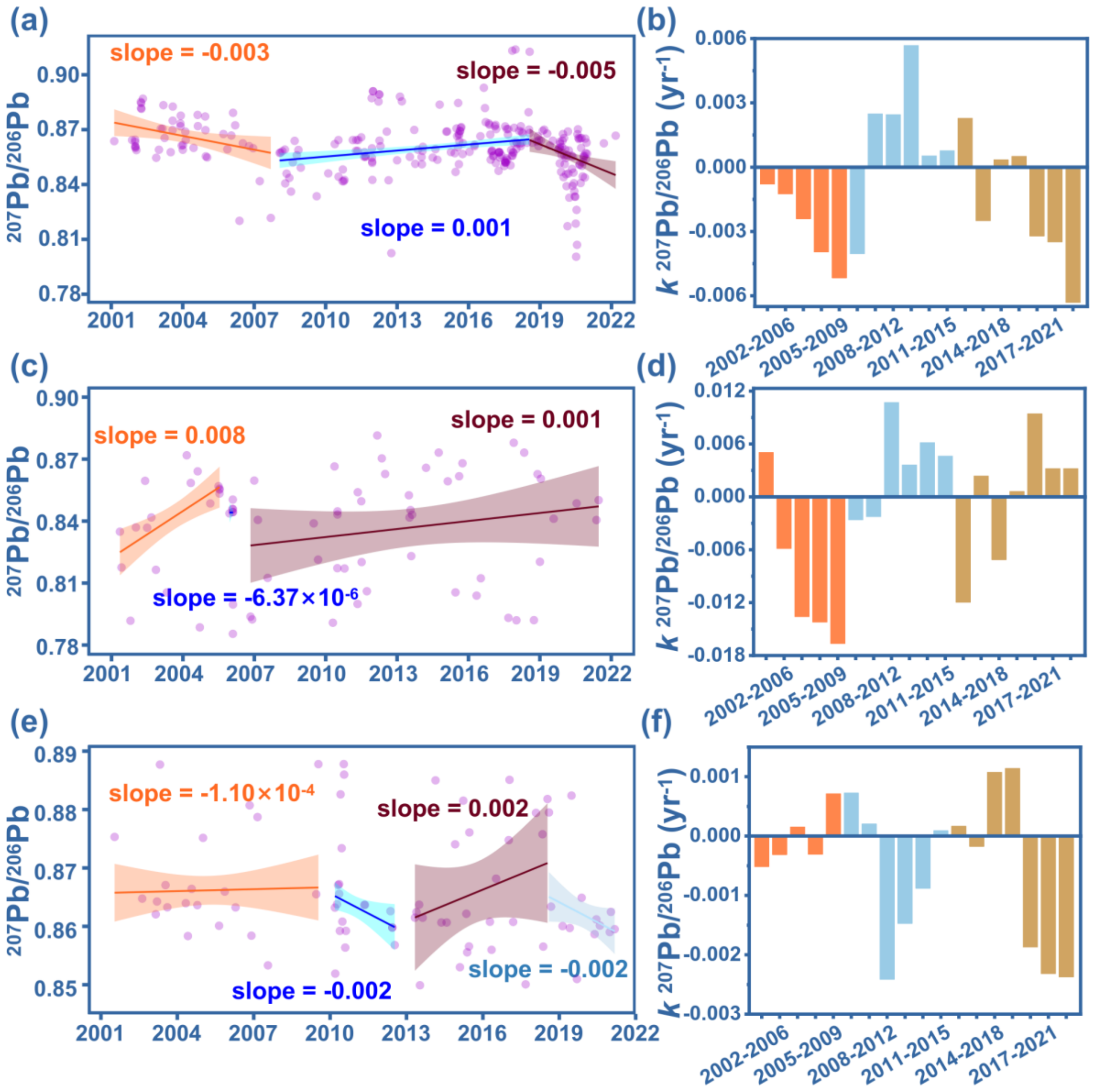

Supplementary Figure 15. Trend and moving window analyses of Pb isotopic fingerprints in atmospheric fine particulate matter ($PM_{2.5}$) using the Theil-Sen estimator. a, c, e) Temporal trends of $^{207}Pb/^{206}Pb$ of Asia (**a**), the Americas (**c**), and Europe (**e**). **b, d, f)** Moving subset window analysis of the temporal change trends of $^{207}Pb/^{206}Pb$ of Asia (**b**), the Americas (**d**), and Europe (**f**). The colored lines represent linear fits for different time periods, and the shaded areas indicate their 95% confidence intervals. The bars show the rates (*k*) of isotopic change over 5-year intervals. Additionally, the subset window analysis advances in 1-year increments from 2001 to 2022. The color gradient of the bars reflects temporal trends.

3. Supplementary tables

Supplementary Table 1. Statistical analysis reveals variations in source isotopic fingerprints across Asia, Europe, and America.

Isotopes	Sources[1]	Region A (n)	Source isotopic value[2] (Mean±SD)	Region B (n)	Source isotopic value[2] (Mean±SD)	*p*-value	Statistical[3]	Test type
δ^{13}C-EC	BB_{C3}	Asia (100)	-27.94 ± 1.77	America (7)	-24.15 ± 6.03	0.2813		Mann-Whitney test
δ^{13}C-EC	BB_{C3}	Asia (100)	-27.94 ± 1.77	Europe (53)	-27.02 ± 3.13	< 0.0001	****	Mann-Whitney test
δ^{13}C-EC	BB_{C3}	America (7)	-24.15 ± 6.03	Europe (53)	-27.02 ± 3.13	0.3221		Mann-Whitney test
δ^{13}C-EC	BB_{C4}	Asia (36)	-15.21 ± 2.37	America (5)	-18.08 ± 4.13	0.1692		Mann-Whitney test
δ^{13}C-EC	BB_{C4}	Asia (36)	-15.21 ± 2.37	Europe (5)	-13.84 ± 2.43	0.2397		Mann-Whitney test
δ^{13}C-EC	BB_{C4}	America (5)	-18.08 ± 4.13	Europe (5)	-13.84 ± 2.43	0.0912		Unpaired Student *t*-test
δ^{13}C-EC	CC	Asia (34)	-24.21 ± 0.92	America (4)	-24.24 ± 2.07	0.6513		Mann-Whitney test
δ^{13}C-EC	CC	Asia (34)	-24.21 ± 0.92	Europe (14)	-24.48 ± 1.13	0.1844		Mann-Whitney test
δ^{13}C-EC	CC	America (4)	-24.24 ± 2.07	Europe (14)	-24.48 ± 1.13	0.8339		Unpaired Student *t*-test
δ^{13}C-EC	VE	Asia (24)	-24.83 ± 1.22	America (12)	-26.52 ± 1.53	0.1395		Mann-Whitney test
δ^{13}C-EC	VE	Asia (24)	-24.83 ± 1.22	Europe (44)	-25.40 ± 1.23	0.1736		Mann-Whitney test
δ^{13}C-EC	VE	America (12)	-26.52 ± 1.53	Europe (44)	-25.40 ± 1.23	0.6035		Mann-Whitney test
δ^{13}C-OC	BB_{C3}	Asia (4)	-27.48 ± 1.02	America (7)	-27.44 ± 0.44	0.9476		Unpaired Student *t*-test
δ^{13}C-OC	BB_{C3}	Asia (4)	-27.48 ± 1.02	Europe (3)	-27.27 ± 0.05	0.7161		Mann-Whitney test
δ^{13}C-OC	BB_{C3}	America (7)	-27.44 ± 0.44	Europe (3)	-27.27 ± 0.05	0.3558		Mann-Whitney test
δ^{13}C-OC	BB_{C4}	Asia (3)	-14.64 ± 0.75	America (4)	-17.12 ± 4.06	0.3113		Unpaired Student *t*-test
δ^{13}C-OC	BB_{C4}	Asia (3)	-14.64 ± 0.75	Europe (3)	-17.96 ± 3.73	0.2610		Unpaired Student *t*-test

δ^{13}C-OC	BB_{C4}	America (4)	-17.12 ± 4.06	Europe (3)	-17.96 ± 3.73	0.7896		Unpaired Student *t*-test
δ^{13}C-OC	CC	Asia (12)	-24.66 ± 1.58	America (4)	-24.54 ± 0.79	0.7618		Mann-Whitney test
δ^{13}C-OC	CC	Asia (12)	-24.66 ± 1.58	Europe (5)	-24.55 ± 2.31	0.4930		Mann-Whitney test
δ^{13}C-OC	CC	America (4)	-24.54 ± 0.79	Europe (5)	-24.55 ± 2.31	0.3893		Mann-Whitney test
δ^{13}C-OC	LFF	Asia (4)	-27.65 ± 1.51	America (4)	-26.49 ± 0.89	0.2430		Unpaired Student *t*-test
δ^{13}C-OC	LFF	Asia (4)	-27.65 ± 1.51	Europe (6)	-26.24 ± 2.09	0.2517		Unpaired Student *t*-test
δ^{13}C-OC	LFF	America (4)	-26.49 ± 0.89	Europe (6)	-26.24 ± 2.09	0.8049		Unpaired Student *t*-test
$\delta^{15}N-NO_x$	BB	Asia (63)	-3.08 ± 3.80	America (43)	-0.10 ± 4.80	0.0003	***	Mann-Whitney test
$\delta^{15}N-NO_x$	CC	Asia (12)	8.54 ± 9.33	America (42)	14.21 ± 4.51	0.0542		Mann-Whitney test
$\delta^{15}N-NO_x$	VE_{diesel}	Asia (15)	-13.02 ± 3.90	America (28)	-11.54 ± 9.10	0.4609		Mann-Whitney test
$\delta^{15}N-NO_x$	$VE_{gasoline}$	Asia (43)	-7.75 ± 4.77	America (71)	-8.38 ± 5.89	0.1740		Mann-Whitney test
$\delta^{15}N-NO_x$	MicP	Asia (48)	-26.87 ± 15.45	America (77)	-34.62 ± 11.11	0.0158	*	Welch's *t*-test
δ^{34}S	BB	Asia (18)	2.94 ± 5.59	America (4)	0.17 ± 4.77	0.3508		Mann-Whitney test
δ^{34}S	BB	Asia (18)	2.94 ± 5.59	Europe (4)	-1.46 ± 4.38	0.1412		Mann-Whitney test
δ^{34}S	BB	America (4)	0.17 ± 4.77	Europe (4)	-1.46 ± 4.38	0.6328		Mann-Whitney test
δ^{34}S	CC	Asia (284)	5.15 ± 9.11	America (5)	1.64 ± 2.75	0.1616		Mann-Whitney test
δ^{34}S	CC	Asia (284)	5.15 ± 9.11	Europe (10)	3.42 ± 3.63	0.3657		Mann-Whitney test
δ^{34}S	CC	America (5)	1.64 ± 2.75	Europe (10)	3.42 ± 3.63	0.4260		Unpaired Student *t*-test
δ^{34}S	VE	Asia (4)	5.28 ± 3.19	America (4)	5.01 ± 2.28	0.8981		Unpaired Student *t*-test
δ^{34}S	VE	Asia (4)	5.28 ± 3.19	Europe (4)	7.14 ± 3.11	0.4352		Unpaired Student *t*-test
δ^{34}S	VE	America (4)	5.01 ± 2.28	Europe (4)	7.14 ± 3.11	0.3165		Unpaired Student *t*-test

$\delta^{34}S$	NS	Asia (139)	6.81 ± 6.11	America (6)	6.87 ± 7.26	0.7320		Unpaired Student *t*-test
$\delta^{34}S$	NS	Asia (139)	6.81 ± 6.11	Europe (5)	8.45 ± 5.36	0.1174		Unpaired Student *t*-test
$\delta^{34}S$	NS	America (6)	6.87 ± 7.26	Europe (5)	8.45 ± 5.36	0.6875		Unpaired Student *t*-test
$\delta^{34}S$	IOC	Asia (42)	5.59 ± 8.91	America (5)	5.14 ± 8.65	0.9172		Unpaired Student *t*-test
$\delta^{34}S$	IOC	Asia (42)	5.59 ± 8.91	Europe (5)	1.21 ± 7.11	0.2557		Unpaired Student *t*-test
$\delta^{34}S$	IOC	America (5)	5.14 ± 8.65	Europe (5)	1.21 ± 7.11	0.4554		Unpaired Student *t*-test
$\delta^{65}Cu$	BB	Asia (10)	-0.30 ± 0.45	America (6)	0.04 ± 0.39	0.1423		Unpaired Student *t*-test
$\delta^{65}Cu$	BB	Asia (10)	-0.30 ± 0.45	Europe (7)	-0.07 ± 0.22	0.1809		Unpaired Student *t*-test
$\delta^{65}Cu$	BB	America (6)	0.04 ± 0.39	Europe (7)	-0.07 ± 0.22	0.5810		Unpaired Student *t*-test
$\delta^{65}Cu$	VE	Asia (6)	-0.15 ± 0.09	America (3)	-0.01 ± 0.29	0.3923		Mann-Whitney test
$\delta^{65}Cu$	VE	Asia (6)	-0.15 ± 0.09	Europe (6)	-0.02 ± 0.25	0.2756		Welch's *t*-test
$\delta^{65}Cu$	VE	America (3)	-0.01 ± 0.29	Europe (6)	-0.02 ± 0.25	0.4555		Mann-Whitney test
$\delta^{65}Cu$	NS	Asia (59)	0.09 ± 0.20	America (10)	-0.58 ± 0.78	0.0241	*	Welch's *t*-test
$\delta^{65}Cu$	NS	Asia (59)	0.09 ± 0.20	Europe (15)	-1.85 ± 1.21	< 0.0001	****	Welch's *t*-test
$\delta^{65}Cu$	NS	America (10)	-0.58 ± 0.78	Europe (15)	-1.85 ± 1.21	0.0043	**	Unpaired Student *t*-test
$\delta^{65}Cu$	NEE_{BP}	Asia (17)	0.15 ± 0.06	America (7)	0.12 ± 0.22	0.2025		Mann-Whitney test
$\delta^{65}Cu$	NEE_{BP}	Asia (17)	0.15 ± 0.06	Europe (6)	0.24 ± 0.32	> 0.9999		Mann-Whitney test
$\delta^{65}Cu$	NEE_{BP}	America (7)	0.12 ± 0.22	Europe (6)	0.24 ± 0.32	0.7188		Mann-Whitney test
$\delta^{65}Cu$	NEE_{T}	Asia (3)	-0.49 ± 0.07	America (4)	0.03 ± 0.17	0.0042	**	Unpaired Student *t*-test
$\delta^{65}Cu$	NEE_{T}	Asia (3)	-0.49 ± 0.07	Europe (3)	0.26 ± 0.08	0.0003	***	Unpaired Student *t*-test

$\delta^{65}Cu$	NEE_T	America (4)	0.03 ± 0.17	Europe (3)	0.26 ± 0.08	0.0665		Unpaired Student *t*-test
$\delta^{65}Cu$	NEE_{RP}	Asia (10)	0.08 ± 0.15	America (4)	0.21 ± 0.27	0.4362		Mann-Whitney test
$\delta^{65}Cu$	NEE_{RP}	Asia (10)	0.08 ± 0.15	Europe (4)	0.36 ± 0.39	0.2288		Mann-Whitney test
$\delta^{65}Cu$	NEE_{RP}	America (4)	0.21 ± 0.27	Europe (4)	0.36 ± 0.39	0.5474		Unpaired Student *t*-test
$\delta^{65}Cu$	OE	Asia (19)	-0.62 ± 0.80	America (4)	-0.14 ± 0.52	0.1552		Mann-Whitney test
$\delta^{65}Cu$	OE	Asia (19)	-0.62 ± 0.80	Europe (6)	-0.10 ± 1.38	0.5036		Mann-Whitney test
$\delta^{65}Cu$	OE	America (4)	-0.14 ± 0.52	Europe (6)	-0.10 ± 1.38	0.9504		Unpaired Student *t*-test
$^{207}Pb/^{206}Pb$	CC	Asia (239)	0.83983 ± 0.04156	America (4)	0.72775 ± 0.13658	0.1012		Mann-Whitney test
$^{207}Pb/^{206}Pb$	CC	Asia (239)	0.83983 ± 0.04156	Europe (4)	0.78750 ± 0.10379	0.1810		Mann-Whitney test
$^{207}Pb/^{206}Pb$	CC	America (4)	0.72775 ± 0.13658	Europe (4)	0.78750 ± 0.10379	0.5139		Unpaired Student *t*-test
$^{207}Pb/^{206}Pb$	$VE_{gasoline}$	Asia (46)	0.87264 ± 0.01331	America (9)	0.87722 ± 0.02096	0.9637		Mann-Whitney test
$^{207}Pb/^{206}Pb$	$VE_{gasoline}$	Asia (46)	0.87264 ± 0.01331	Europe (4)	0.90536 ± 0.01471	0.0045	**	Mann-Whitney test
$^{207}Pb/^{206}Pb$	$VE_{gasoline}$	America (9)	0.87722 ± 0.02096	Europe (4)	0.90536 ± 0.01471	0.0233	*	Unpaired Student *t*-test
$^{207}Pb/^{206}Pb$	VE_{diesel}	Asia (11)	0.86673 ± 0.01951	America (4)	0.86433 ± 0.01561	0.7432		Mann-Whitney test
$^{207}Pb/^{206}Pb$	VE_{diesel}	Asia (11)	0.86673 ± 0.01951	Europe (3)	0.86292 ± 0.00056	0.5316		Mann-Whitney test
$^{207}Pb/^{206}Pb$	VE_{diesel}	America (4)	0.86433 ± 0.01561	Europe (3)	0.86292 ± 0.00056	0.8684		Welch's *t*-test
$^{207}Pb/^{206}Pb$	NS	Asia (121)	0.84076 ± 0.02940	America (6)	0.85359 ± 0.00707	0.0175	*	Mann-Whitney test
$^{207}Pb/^{206}Pb$	NS	Asia (121)	0.84076 ± 0.02940	Europe (16)	0.85560 ± 0.00468	0.0001	****	Mann-Whitney test
$^{207}Pb/^{206}Pb$	NS	America (6)	0.85359 ± 0.00707	Europe (16)	0.85560 ± 0.00468	0.2828		Mann-Whitney test
$^{207}Pb/^{206}Pb$	NEE_{BP}	Asia (17)	0.81921 ± 0.05883	America (3)	0.72821 ± 0.18611	0.3406		Mann-Whitney test
$^{207}Pb/^{206}Pb$	NEE_{BP}	Asia (17)	0.81921 ± 0.05883	Europe (3)	0.82499 ± 0.03099	0.8323		Mann-Whitney test
$^{207}Pb/^{206}Pb$	NEE_{BP}	America (3)	0.72821 ± 0.18611	Europe (3)	0.82499 ± 0.03099	0.4638		Unpaired Student *t*-test
$^{207}Pb/^{206}Pb$	NEE_T	Asia (3)	0.86342 ± 0.00246	America (6)	0.85098 ± 0.00980	0.0257	*	Unpaired Student *t*-test

$^{207}Pb/^{206}Pb$	NEE_T	Asia (3)	0.86342 ± 0.00246	Europe (4)	0.86157 ± 0.00544	0.5772		Unpaired Student *t*-test
$^{207}Pb/^{206}Pb$	NEE_T	America (6)	0.85098 ± 0.00980	Europe (4)	0.86157 ± 0.00544	0.0604		Unpaired Student *t*-test
$^{207}Pb/^{206}Pb$	NEE_{RP}	Asia (10)	0.81127 ± 0.06104	America (4)	0.82888 ± 0.04395	0.5641		Unpaired Student *t*-test
$^{207}Pb/^{206}Pb$	NEE_{RP}	Asia (10)	0.81127 ± 0.06104	Europe (4)	0.80725 ± 0.04737	0.8990		Unpaired Student *t*-test
$^{207}Pb/^{206}Pb$	NEE_{RP}	America (4)	0.82888 ± 0.04395	Europe (4)	0.80725 ± 0.04737	0.5284		Unpaired Student *t*-test
$^{207}Pb/^{206}Pb$	OE	Asia (48)	0.86114 ± 0.02821	America (7)	0.86791 ± 0.00048	0.0066	**	Mann-Whitney test
$^{207}Pb/^{206}Pb$	OE	Asia (48)	0.86114 ± 0.02821	Europe (6)	0.86792 ± 0.00044	0.0093	**	Mann-Whitney test
$^{207}Pb/^{206}Pb$	OE	America (7)	0.86791 ± 0.00048	Europe (6)	0.86792 ± 0.00044	0.9876		Unpaired Student *t*-test
$^{207}Pb/^{206}Pb$	WI	Asia (11)	0.86466 ± 0.00149	America (6)	0.86917 ± 0.00214	0.0019	**	Unpaired Student *t*-test
$^{207}Pb/^{206}Pb$	WI	Asia (11)	0.86466 ± 0.00149	Europe (12)	0.86977 ± 0.00329	0.0002	***	Welch's *t*-test
$^{207}Pb/^{206}Pb$	WI	America (6)	0.86917 ± 0.00214	Europe (12)	0.86977 ± 0.00329	0.6482		Unpaired Student *t*-test
$\delta^{144}Nd$	CC	Asia (7)	1.20 ± 0.36	America (4)	1.06 ± 0.24	0.4453		Unpaired Student *t*-test
$\delta^{144}Nd$	CC	Asia (7)	1.20 ± 0.36	Europe (4)	0.92 ± 0.33	0.2193		Mann-Whitney test
$\delta^{144}Nd$	CC	America (4)	1.06 ± 0.24	Europe (4)	0.92 ± 0.33	0.4705		Mann-Whitney test
$\delta^{144}Nd$	VE	Asia (8)	0.97 ± 0.34	America (4)	0.19 ± 0.98	0.2079		Welch's *t*-test
$\delta^{144}Nd$	VE	Asia (8)	0.97 ± 0.34	Europe (6)	0.17 ± 0.85	0.0688		Mann-Whitney test
$\delta^{144}Nd$	VE	America (4)	0.19 ± 0.98	Europe (6)	0.17 ± 0.85	0.9151		Mann-Whitney test
$\delta^{144}Nd$	NS	Asia (31)	1.19 ± 0.48	America (6)	-0.39 ± 1.12	0.0032	**	Mann-Whitney test
$\delta^{144}Nd$	NS	Asia (31)	1.19 ± 0.48	Europe (3)	1.04 ± 0.08	0.3312		Mann-Whitney test
$\delta^{144}Nd$	NS	America (6)	-0.39 ± 1.12	Europe (3)	1.04 ± 0.08	0.2453		Mann-Whitney test
$\delta^{144}Nd$	NEE	Asia (16)	1.48 ± 0.09	America (3)	0.91 ± 0.07	0.0007	***	Unpaired Student *t*-test

δ^{144}Nd	NEE	Asia (16)	1.48 ± 0.09	Europe (3)	1.08 ± 0.06	0.0003	***	Unpaired Student *t*-test
δ^{144}Nd	NEE	America (3)	0.91 ± 0.07	Europe (3)	1.08 ± 0.06	0.0354	*	Unpaired Student *t*-test
δ^{144}Nd	WI	Asia (3)	0.44 ± 0.17	America (4)	0.77 ± 0.22	0.0837		Unpaired Student *t*-test
δ^{144}Nd	WI	Asia (3)	0.44 ± 0.17	Europe (10)	0.98 ± 0.11	0.0135	*	Mann-Whitney test
δ^{144}Nd	WI	America (4)	0.77 ± 0.22	Europe (10)	0.98 ± 0.11	0.1012		Mann-Whitney test
δ^{87}Sr	CC	Asia (9)	20.93 ± 20.93	America (4)	26.14 ± 26.14	0.0372	*	Mann-Whitney test
δ^{87}Sr	CC	Asia (9)	20.93 ± 20.93	Europe (4)	34.44 ± 34.44	0.0372	*	Mann-Whitney test
δ^{87}Sr	CC	America (4)	26.14 ± 26.14	Europe (4)	34.44 ± 34.44	0.2620		Unpaired Student *t*-test
δ^{87}Sr	VE	Asia (8)	27.89 ± 27.89	America (4)	20.26 ± 20.26	0.1440		Unpaired Student *t*-test
δ^{87}Sr	VE	Asia (8)	27.89 ± 27.89	Europe (8)	19.03 ± 19.03	0.1275		Mann-Whitney test
δ^{87}Sr	VE	America (4)	20.26 ± 20.26	Europe (8)	19.03 ± 19.03	0.7986		Mann-Whitney test
δ^{87}Sr	NS	Asia (67)	23.24 ± 23.24	America (9)	16.53 ± 16.53	0.0366	*	Mann-Whitney test
δ^{87}Sr	NS	Asia (67)	23.24 ± 23.24	Europe (6)	24.57 ± 24.57	0.6513		Mann-Whitney test
δ^{87}Sr	NS	America (9)	16.53 ± 16.53	Europe (6)	24.57 ± 24.57	0.2061		Welch's *t*-test
δ^{87}Sr	NEE	Asia (20)	14.98 ± 14.98	America (4)	15.13 ± 15.13	> 0.9999		Mann-Whitney test
δ^{87}Sr	NEE	Asia (20)	14.98 ± 14.98	Europe (5)	15.08 ± 15.08	0.4751		Mann-Whitney test
δ^{87}Sr	NEE	America (4)	15.13 ± 15.13	Europe (5)	15.08 ± 15.08	0.9516		Unpaired Student *t*-test
δ^{87}Sr	OE	Asia (3)	18.18 ± 18.18	America (6)	17.47 ± 17.47	0.1217		Unpaired Student *t*-test
δ^{87}Sr	OE	Asia (3)	18.18 ± 18.18	Europe (3)	17.60 ± 17.60	0.1985		Unpaired Student *t*-test
δ^{87}Sr	OE	America (6)	17.47 ± 17.47	Europe (3)	17.60 ± 17.60	0.6698		Unpaired Student *t*-test
δ^{56}Fe	BB	Asia (5)	-0.41 ± 0.49	America (5)	-0.54 ± 0.28	0.6150		Unpaired Student *t*-test
δ^{56}Fe	BB	Asia (5)	-0.41 ± 0.49	Europe (90)	-0.66 ± 0.79	0.5766		Mann-Whitney test

δ^{56}Fe	BB	America (5)	-0.54 ± 0.28	Europe (90)	-0.66 ± 0.79	0.9801	Mann-Whitney test
δ^{56}Fe	CC	Asia (4)	-0.16 ± 0.36	America (3)	0.34 ± 0.37	0.1389	Unpaired Student *t*-test
δ^{56}Fe	CC	Asia (4)	-0.16 ± 0.36	Europe (3)	0.07 ± 0.01	0.3003	Welch's *t*-test
δ^{56}Fe	CC	America (3)	0.34 ± 0.37	Europe (3)	0.07 ± 0.01	0.3219	Welch's *t*-test
δ^{56}Fe	VE	Asia (5)	0.21 ± 0.09	America (6)	0.12 ± 0.07	0.1164	Unpaired Student *t*-test
δ^{56}Fe	VE	Asia (5)	0.21 ± 0.09	Europe (3)	0.16 ± 0.11	0.5773	Unpaired Student *t*-test
δ^{56}Fe	VE	America (6)	0.12 ± 0.07	Europe (3)	0.16 ± 0.11	0.6119	Unpaired Student *t*-test
δ^{56}Fe	NS	Asia (4)	0.04 ± 0.12	America (8)	-0.01 ± 0.06	0.5699	Unpaired Student *t*-test
δ^{56}Fe	NS	Asia (4)	0.04 ± 0.12	Europe (4)	-0.01 ± 0.04	0.5408	Unpaired Student *t*-test
δ^{56}Fe	NS	America (8)	-0.01 ± 0.06	Europe (4)	-0.01 ± 0.04	0.9327	Unpaired Student *t*-test
δ^{56}Fe	NEE	Asia (5)	0.23 ± 0.31	America (12)	0.12 ± 0.21	0.4769	Unpaired Student *t*-test
δ^{56}Fe	NEE	Asia (5)	0.23 ± 0.31	Europe (4)	0.13 ± 0.15	0.5311	Unpaired Student *t*-test
δ^{56}Fe	NEE	America (12)	0.12 ± 0.21	Europe (4)	0.13 ± 0.15	0.9176	Unpaired Student *t*-test
δ^{56}Fe	OE	Asia (5)	0.62 ± 0.62	America (6)	0.41 ± 0.87	0.3153	Mann-Whitney test
δ^{56}Fe	OE	Asia (5)	0.62 ± 0.62	Europe (6)	0.58 ± 0.44	0.8952	Unpaired Student *t*-test
δ^{56}Fe	OE	America (6)	0.41 ± 0.87	Europe (6)	0.58 ± 0.44	0.2980	Mann-Whitney test
δ^{66}Zn	BB	Asia (7)	0.42 ± 0.23	America (4)	0.49 ± 0.17	0.5957	Unpaired Student *t*-test
δ^{66}Zn	BB	Asia (7)	0.42 ± 0.23	Europe (66)	0.48 ± 0.25	0.6733	Mann-Whitney test
δ^{66}Zn	BB	America (4)	0.49 ± 0.17	Europe (66)	0.48 ± 0.25	0.6762	Mann-Whitney test
δ^{66}Zn	CC	Asia (5)	0.25 ± 0.23	America (26)	0.32 ± 0.51	0.6375	Unpaired Student *t*-test

$\delta^{66}Zn$	CC	Asia (5)	0.25 ± 0.23	Europe (15)	1.02 ± 0.46	0.0002	***	Unpaired Student *t*-test
$\delta^{66}Zn$	CC	America (26)	0.32 ± 0.51	Europe (15)	1.02 ± 0.46	0.0001	****	Unpaired Student *t*-test
$\delta^{66}Zn$	VE	Asia (74)	-0.14 ± 0.15	America (15)	0.28 ± 0.26	< 0.0001	****	Mann-Whitney test
$\delta^{66}Zn$	VE	Asia (74)	-0.14 ± 0.15	Europe (12)	0.29 ± 0.25	< 0.0001	****	Mann-Whitney test
$\delta^{66}Zn$	VE	America (15)	0.28 ± 0.26	Europe (12)	0.29 ± 0.25	0.5745		Mann-Whitney test
$\delta^{66}Zn$	NS	Asia (39)	0.54 ± 0.16	America (4)	0.53 ± 0.20	0.8778		Unpaired Student *t*-test
$\delta^{66}Zn$	NS	Asia (39)	0.54 ± 0.16	Europe (4)	0.29 ± 0.43	0.3208		Welch's *t*-test
$\delta^{66}Zn$	NS	America (4)	0.53 ± 0.20	Europe (4)	0.29 ± 0.43	0.3698		Unpaired Student *t*-test
$\delta^{66}Zn$	NEE_{BP}	Asia (17)	-0.03 ± 0.09	America (3)	-0.02 ± 0.06	0.7984		Unpaired Student *t*-test
$\delta^{66}Zn$	NEE_{BP}	Asia (17)	-0.03 ± 0.09	Europe (3)	0.47 ± 0.03	< 0.0001	****	Unpaired Student *t*-test
$\delta^{66}Zn$	NEE_{BP}	America (3)	-0.02 ± 0.06	Europe (3)	0.47 ± 0.03	0.0012	**	Unpaired Student *t*-test
$\delta^{66}Zn$	NEE_{T}	Asia (5)	-0.01 ± 0.06	America (8)	0.32 ± 0.30	0.0331	*	Mann-Whitney test
$\delta^{66}Zn$	NEE_{T}	Asia (5)	-0.01 ± 0.06	Europe (6)	0.45 ± 0.05	< 0.0001	****	Unpaired Student *t*-test
$\delta^{66}Zn$	NEE_{T}	America (8)	0.32 ± 0.30	Europe (6)	0.45 ± 0.05	0.6492		Mann-Whitney test
$\delta^{66}Zn$	WI	Asia (5)	0.21 ± 0.09	America (4)	0.20 ± 0.13	0.8530		Unpaired Student *t*-test
$\delta^{66}Zn$	WI	Asia (5)	0.21 ± 0.09	Europe (6)	0.19 ± 0.12	0.7575		Unpaired Student *t*-test
$\delta^{66}Zn$	WI	America (4)	0.20 ± 0.13	Europe (6)	0.19 ± 0.12	0.9484		Unpaired Student *t*-test
$\delta^{66}Zn$	NEE_{RP}	Asia (10)	-0.14 ± 0.26	America (6)	0.64 ± 0.17	0.0014	**	Mann-Whitney test
$\delta^{66}Zn$	NEE_{RP}	Asia (10)	-0.14 ± 0.26	Europe (5)	0.59 ± 0.25	0.0026	**	Mann-Whitney test
$\delta^{66}Zn$	NEE_{RP}	America (6)	0.64 ± 0.17	Europe (5)	0.59 ± 0.25	0.6974		Unpaired Student *t*-test

$\delta^{66}Zn$	OE	Asia (5)	0.03 ± 0.34	America (5)	0.05 ± 0.28	0.9059	Unpaired Student *t*-test
$\delta^{66}Zn$	OE	Asia (5)	0.03 ± 0.34	Europe (20)	0.20 ± 0.32	0.3952	Mann-Whitney test
$\delta^{66}Zn$	OE	America (5)	0.05 ± 0.28	Europe (20)	0.20 ± 0.32	0.1632	Mann-Whitney test

1. Sources types (abbreviations): Biomass Burning (BB), Biomass Burning-C3 plants (BB_{C3}), Biomass Burning-C4 plants (BB_{C4}), Coal Combustion (CC), Vehicle Exhausts (VE), Vehicle Exhausts-Gasoline Vehicle ($VE_{gasoline}$), Vehicle Exhausts-Diesel Vehicle (VE_{diesel}), Natural Soil (NS), Ore-related Emissions (OE), Waste Materials (WM), Non-exhaust emissions (NEE), Non-exhaust emissions-Brake Pads (NEE_{BP}), Non-exhaust emissions-Road Paint (NEE_{RP}), Non-exhaust emissions-Tires (NEE_{T}), Waste Incinerator (WI), Liquid Fossil Fuels (LFF), Livestock Emissions (LE), Microbial Processes (MicP), Volatilized Fertilizer (VF), Industrial Oil Combustion (IOC).

2. All results are in ‰ except for $^{207}Pb/^{206}Pb$

3. Significance code: '****' < 0.0001, '***' < 0.001, '**' < 0.01, '*' < 0.05

Supplementary Table 2. Statistical variations in isotopic fingerprints from different sources within the same region.

Isotopes	Region	Source A[1] (n)	Source isotopic value[2] (Mean±SD)	Source B[1] (n)	Source isotopic value[2] (Mean±SD)	*p*-value	Statistical[3]	Test type
δ^{13}C-EC	Asia	BB_{C3} (100)	-27.94 ± 1.77	BB_{C4} (36)	-15.21 ± 2.37	< 0.0001	****	Mann-Whitney test
δ^{13}C-EC	Asia	BB_{C3} (100)	-27.94 ± 1.77	CC (34)	-24.21 ± 0.92	< 0.0001	****	Mann-Whitney test
δ^{13}C-EC	Asia	BB_{C3} (100)	-27.94 ± 1.77	VE (24)	-24.83 ± 1.22	< 0.0001	****	Mann-Whitney test
δ^{13}C-EC	Asia	BB_{C4} (36)	-15.21 ± 2.37	CC (34)	-24.21 ± 0.92	< 0.0001	****	Mann-Whitney test
δ^{13}C-EC	Asia	BB_{C4} (36)	-15.21 ± 2.37	VE (24)	-24.83 ± 1.22	< 0.0001	****	Mann-Whitney test
δ^{13}C-EC	Asia	CC (34)	-24.21 ± 0.92	VE (24)	-24.83 ± 1.22	0.0028	**	Mann-Whitney test
δ^{13}C-EC	America	BB_{C3} (7)	-24.15 ± 6.03	BB_{C4} (5)	-18.08 ± 4.13	0.0654		Unpaired Student *t*-test
δ^{13}C-EC	America	BB_{C3} (7)	-24.15 ± 6.03	CC (4)	-24.24 ± 2.07	0.9729		Unpaired Student *t*-test
δ^{13}C-EC	America	BB_{C3} (7)	-24.15 ± 6.03	VE (12)	-26.52 ± 1.53	0.5425		Welch's *t*-test
δ^{13}C-EC	America	BB_{C4} (5)	-18.08 ± 4.13	CC (4)	-24.24 ± 2.07	0.0264	*	Unpaired Student *t*-test
δ^{13}C-EC	America	BB_{C4} (5)	-18.08 ± 4.13	VE (12)	-26.52 ± 1.53	0.0131	*	Welch's *t*-test
δ^{13}C-EC	America	CC (4)	-24.24 ± 2.07	VE (12)	-26.52 ± 1.53	0.2769		Unpaired Student *t*-test
δ^{13}C-EC	Europe	BB_{C3} (53)	-27.02 ± 3.13	BB_{C4} (5)	-13.84 ± 2.43	0.0003	***	Mann-Whitney test
δ^{13}C-EC	Europe	BB_{C3} (53)	-27.02 ± 3.13	CC (14)	-24.48 ± 1.13	0.0003	***	Mann-Whitney test
δ^{13}C-EC	Europe	BB_{C3} (53)	-27.02 ± 3.13	VE (44)	-25.40 ± 1.23	0.0048	**	Mann-Whitney test
δ^{13}C-EC	Europe	BB_{C4} (5)	-13.84 ± 2.43	CC (14)	-24.48 ± 1.13	0.0003	***	Welch's *t*-test
δ^{13}C-EC	Europe	BB_{C4} (5)	-13.84 ± 2.43	VE (44)	-25.40 ± 1.23	0.0003	***	Mann-Whitney test
δ^{13}C-EC	Europe	CC (14)	-24.48 ± 1.13	VE (44)	-25.40 ± 1.23	0.0248	*	Mann-Whitney test
δ^{13}C-OC	Asia	BB_{C3} (4)	-27.48 ± 1.02	BB_{C4} (3)	-14.64 ± 0.75	< 0.0001	****	Unpaired Student *t*-test
δ^{13}C-OC	Asia	BB_{C3} (4)	-27.48 ± 1.02	CC (12)	-24.66 ± 1.58	0.0249	*	Mann-Whitney test

δ^{13}C-OC	Asia	BB_{C3} (4)	-27.48 ± 1.02	LFF (4)	-27.65 ± 1.51	0.8547		Unpaired Student *t*-test
δ^{13}C-OC	Asia	BB_{C4} (3)	-14.64 ± 0.75	CC (12)	-24.66 ± 1.58	0.0115	*	Mann-Whitney test
δ^{13}C-OC	Asia	BB_{C4} (3)	-14.64 ± 0.75	LFF (4)	-27.65 ± 1.51	< 0.0001	****	Unpaired Student *t*-test
δ^{13}C-OC	Asia	CC (12)	-24.66 ± 1.58	LFF (4)	-27.65 ± 1.51	0.0180	*	Mann-Whitney test
δ^{13}C-OC	America	BB_{C3} (7)	-27.44 ± 0.44	BB_{C4} (4)	-17.12 ± 4.06	0.0144	*	Welch's *t*-test
δ^{13}C-OC	America	BB_{C3} (7)	-27.44 ± 0.44	CC (4)	-24.54 ± 0.79	0.0024	**	Unpaired Student *t*-test
δ^{13}C-OC	America	BB_{C3} (7)	-27.44 ± 0.44	LFF (4)	-26.49 ± 0.89	0.1193		Unpaired Student *t*-test
δ^{13}C-OC	America	BB_{C4} (4)	-17.12 ± 4.06	CC (4)	-24.54 ± 0.79	0.0330	*	Welch's *t*-test
δ^{13}C-OC	America	BB_{C4} (4)	-17.12 ± 4.06	LFF (4)	-26.49 ± 0.89	0.0168	*	Welch's *t*-test
δ^{13}C-OC	America	CC (4)	-24.54 ± 0.79	LFF (4)	-26.49 ± 0.89	0.0175	*	Unpaired Student *t*-test
δ^{13}C-OC	Europe	BB_{C3} (3)	-27.27 ± 0.05	BB_{C4} (3)	-17.96 ± 3.73	0.0496	*	Welch's *t*-test
δ^{13}C-OC	Europe	BB_{C3} (3)	-27.27 ± 0.05	CC (5)	-24.55 ± 2.31	0.2302		Mann-Whitney test
δ^{13}C-OC	Europe	BB_{C3} (3)	-27.27 ± 0.05	LFF (6)	-26.24 ± 2.09	0.2826		Welch's *t*-test
δ^{13}C-OC	Europe	BB_{C4} (3)	-17.96 ± 3.73	CC (5)	-24.55 ± 2.31	0.0358	*	Mann-Whitney test
δ^{13}C-OC	Europe	BB_{C4} (3)	-17.96 ± 3.73	LFF (6)	-26.24 ± 2.09	0.0456	*	Unpaired Student *t*-test
δ^{13}C-OC	Europe	CC (5)	-24.55 ± 2.31	LFF (6)	-26.24 ± 2.09	0.1699		Mann-Whitney test
δ^{15}N-NH_3	Asia	BB (22)	-11.57 ± 6.25	CC (46)	-9.39 ± 11.56	0.3180		Welch's *t*-test
δ^{15}N-NH_3	Asia	BB (22)	-11.57 ± 6.25	VE (104)	1.82 ± 4.59	< 0.0001	****	Mann-Whitney test
δ^{15}N-NH_3	Asia	BB (22)	-11.57 ± 6.25	VF (141)	-22.84 ± 8.96	< 0.0001	****	Mann-Whitney test
δ^{15}N-NH_3	Asia	BB (22)	-11.57 ± 6.25	LE (49)	-19.45 ± 4.77	< 0.0001	****	Unpaired Student *t*-test
δ^{15}N-NH_3	Asia	BB (22)	-11.57 ± 6.25	WM (181)	-19.15 ± 4.60	< 0.0001	****	Mann-Whitney test
δ^{15}N-NH_3	Asia	CC (46)	-9.39 ± 11.56	VE (104)	1.82 ± 4.59	< 0.0001	****	Mann-Whitney test
δ^{15}N-NH_3	Asia	CC (46)	-9.39 ± 11.56	VF (141)	-22.84 ± 8.96	< 0.0001	****	Mann-Whitney test

$\delta^{15}N$-NH_3	Asia	CC (46)	-9.39 ± 11.56	LE (49)	-19.45 ± 4.77	< 0.0001	****	Welch's *t*-test
$\delta^{15}N$-NH_3	Asia	CC (46)	-9.39 ± 11.56	WM (181)	-19.15 ± 4.60	< 0.0001	****	Mann-Whitney test
$\delta^{15}N$-NH_3	Asia	VE (104)	1.82 ± 4.59	VF (141)	-22.84 ± 8.96	< 0.0001	****	Mann-Whitney test
$\delta^{15}N$-NH_3	Asia	VE (104)	1.82 ± 4.59	LE (49)	-19.45 ± 4.77	< 0.0001	****	Mann-Whitney test
$\delta^{15}N$-NH_3	Asia	VE (104)	1.82 ± 4.59	WM (181)	-19.15 ± 4.60	< 0.0001	****	Mann-Whitney test
$\delta^{15}N$-NH_3	Asia	VF (141)	-22.84 ± 8.96	LE (49)	-19.45 ± 4.77	0.0003	***	Mann-Whitney test
$\delta^{15}N$-NH_3	Asia	VF (141)	-22.84 ± 8.96	WM (181)	-19.15 ± 4.60	< 0.0001	****	Mann-Whitney test
$\delta^{15}N$-NH_3	Asia	LE (49)	-19.45 ± 4.77	WM (181)	-19.15 ± 4.60	0.7004		Mann-Whitney test
$\delta^{15}N$-NO_x	Asia	BB (63)	-3.08 ± 3.80	CC (12)	8.54 ± 9.33	< 0.0001	****	Mann-Whitney test
$\delta^{15}N$-NO_x	Asia	BB (63)	-3.08 ± 3.80	MicP (48)	-26.87 ± 15.45	< 0.0001	****	Mann-Whitney test
$\delta^{15}N$-NO_x	Asia	BB (63)	-3.08 ± 3.80	$VE_{gasoline}$ (43)	-7.75 ± 4.77	< 0.0001	****	Mann-Whitney test
$\delta^{15}N$-NO_x	Asia	BB (63)	-3.08 ± 3.80	VE_{diesel} (15)	-13.02 ± 3.90	< 0.0001	****	Mann-Whitney test
$\delta^{15}N$-NO_x	Asia	CC (12)	8.54 ± 9.33	MicP (48)	-26.87 ± 15.45	< 0.0001	****	Mann-Whitney test
$\delta^{15}N$-NO_x	Asia	CC (12)	8.54 ± 9.33	$VE_{gasoline}$ (43)	-7.75 ± 4.77	< 0.0001	****	Mann-Whitney test
$\delta^{15}N$-NO_x	Asia	CC (12)	8.54 ± 9.33	VE_{diesel} (15)	-13.02 ± 3.90	< 0.0001	****	Mann-Whitney test
$\delta^{15}N$-NO_x	Asia	MicP (48)	-26.87 ± 15.45	$VE_{gasoline}$ (43)	-7.75 ± 4.77	< 0.0001	****	Mann-Whitney test
$\delta^{15}N$-NO_x	Asia	MicP (48)	-26.87 ± 15.45	VE_{diesel} (15)	-13.02 ± 3.90	< 0.0001	****	Mann-Whitney test
$\delta^{15}N$-NO_x	Asia	$VE_{gasoline}$ (43)	-7.75 ± 4.77	VE_{diesel} (15)	-13.02 ± 3.90	0.0004	***	Mann-Whitney test
$\delta^{15}N$-NO_x	America	BB (43)	-0.10 ± 4.80	CC (42)	14.21 ± 4.51	< 0.0001	****	Mann-Whitney test
$\delta^{15}N$-NO_x	America	BB (43)	-0.10 ± 4.80	MicP (77)	-34.62 ± 11.11	< 0.0001	****	Mann-Whitney test
$\delta^{15}N$-NO_x	America	BB (43)	-0.10 ± 4.80	$VE_{gasoline}$ (71)	-8.38 ± 5.89	< 0.0001	****	Mann-Whitney test
$\delta^{15}N$-NO_x	America	BB (43)	-0.10 ± 4.80	VE_{diesel} (28)	-11.54 ± 9.10	< 0.0001	****	Welch's *t*-test
$\delta^{15}N$-NO_x	America	CC (42)	14.21 ± 4.51	MicP (77)	-34.62 ± 11.11	< 0.0001	****	Mann-Whitney test

$\delta^{15}N-NO_x$	America	CC (42)	14.21 ± 4.51	$VE_{gasoline}$ (71)	-8.38 ± 5.89	< 0.0001	****	Mann-Whitney test
$\delta^{15}N-NO_x$	America	CC (42)	14.21 ± 4.51	VE_{diesel} (28)	-11.54 ± 9.10	< 0.0001	****	Mann-Whitney test
$\delta^{15}N-NO_x$	America	MicP (77)	-34.62 ± 11.11	$VE_{gasoline}$ (71)	-8.38 ± 5.89	< 0.0001	****	Mann-Whitney test
$\delta^{15}N-NO_x$	America	MicP (77)	-34.62 ± 11.11	VE_{diesel} (28)	-11.54 ± 9.10	< 0.0001	****	Mann-Whitney test
$\delta^{15}N-NO_x$	America	$VE_{gasoline}$ (71)	-8.38 ± 5.89	VE_{diesel} (28)	-11.54 ± 9.10	0.0363	*	Mann-Whitney test
$\delta^{34}S$	Asia	CC (284)	5.15 ± 9.11	NS (139)	6.81 ± 6.11	0.0327	*	Mann-Whitney test
$\delta^{34}S$	Asia	CC (284)	5.15 ± 9.11	BB (18)	2.94 ± 5.59	0.3349		Mann-Whitney test
$\delta^{34}S$	Asia	CC (284)	5.15 ± 9.11	VE (4)	5.28 ± 3.19	0.9952		Mann-Whitney test
$\delta^{34}S$	Asia	CC (284)	5.15 ± 9.11	IOC (42)	5.59 ± 8.91	0.9239		Mann-Whitney test
$\delta^{34}S$	Asia	NS (139)	6.81 ± 6.11	BB (18)	2.94 ± 5.59	0.0595		Mann-Whitney test
$\delta^{34}S$	Asia	NS (139)	6.81 ± 6.11	VE (4)	5.28 ± 3.19	0.5445		Mann-Whitney test
$\delta^{34}S$	Asia	NS (139)	6.81 ± 6.11	IOC (42)	5.59 ± 8.91	0.2718		Mann-Whitney test
$\delta^{34}S$	Asia	BB (18)	2.94 ± 5.59	VE (4)	5.28 ± 3.19	0.2927		Unpaired Student *t*-test
$\delta^{34}S$	Asia	BB (18)	2.94 ± 5.59	IOC (42)	5.59 ± 8.91	0.1708		Welch's *t*-test
$\delta^{34}S$	Asia	VE (4)	5.28 ± 3.19	IOC (42)	5.59 ± 8.91	0.8842		Unpaired Student *t*-test
$\delta^{34}S$	America	CC (5)	1.64 ± 2.75	NS (6)	6.87 ± 7.26	0.4113		Mann-Whitney test
$\delta^{34}S$	America	CC (5)	1.64 ± 2.75	BB (4)	0.17 ± 4.77	0.7133		Mann-Whitney test
$\delta^{34}S$	America	CC (5)	1.64 ± 2.75	VE (4)	5.01 ± 2.28	0.1113		Mann-Whitney test
$\delta^{34}S$	America	CC (5)	1.64 ± 2.75	IOC (5)	5.14 ± 8.65	0.8345		Mann-Whitney test
$\delta^{34}S$	America	NS (6)	6.87 ± 7.26	BB (4)	0.17 ± 4.77	0.1150		Unpaired Student *t*-test
$\delta^{34}S$	America	NS (6)	6.87 ± 7.26	VE (4)	5.01 ± 2.28	0.5793		Unpaired Student *t*-test
$\delta^{34}S$	America	NS (6)	6.87 ± 7.26	IOC (5)	5.14 ± 8.65	0.7326		Unpaired Student *t*-test
$\delta^{34}S$	America	BB (4)	0.17 ± 4.77	VE (4)	5.01 ± 2.28	0.1330		Unpaired Student *t*-test
$\delta^{34}S$	America	BB (4)	0.17 ± 4.77	IOC (5)	5.14 ± 8.65	0.3120		Unpaired Student *t*-test

$\delta^{34}S$	America	VE (4)	5.01 ± 2.28	IOC (5)	5.14 ± 8.65	0.9757		Unpaired Student *t*-test
$\delta^{34}S$	Europe	CC (10)	3.42 ± 3.63	NS (5)	8.45 ± 5.36	0.1080		Unpaired Student *t*-test
$\delta^{34}S$	Europe	CC (10)	3.42 ± 3.63	BB (4)	-1.46 ± 4.38	0.1087		Unpaired Student *t*-test
$\delta^{34}S$	Europe	CC (10)	3.42 ± 3.63	VE (4)	7.14 ± 3.11	0.0991		Unpaired Student *t*-test
$\delta^{34}S$	Europe	CC (10)	3.42 ± 3.63	IOC (5)	1.21 ± 7.11	0.5409		Unpaired Student *t*-test
$\delta^{34}S$	Europe	NS (5)	8.45 ± 5.36	BB (4)	-1.46 ± 4.38	0.0186	*	Unpaired Student *t*-test
$\delta^{34}S$	Europe	NS (5)	8.45 ± 5.36	VE (4)	7.14 ± 3.11	0.6600		Unpaired Student *t*-test
$\delta^{34}S$	Europe	NS (5)	8.45 ± 5.36	IOC (5)	1.21 ± 7.11	0.1092		Unpaired Student *t*-test
$\delta^{34}S$	Europe	BB (4)	-1.46 ± 4.38	VE (4)	7.14 ± 3.11	0.0216	*	Unpaired Student *t*-test
$\delta^{34}S$	Europe	BB (4)	-1.46 ± 4.38	IOC (5)	1.21 ± 7.11	0.5136		Unpaired Student *t*-test
$\delta^{34}S$	Europe	VE (4)	7.14 ± 3.11	IOC (5)	1.21 ± 7.11	0.1475		Unpaired Student *t*-test
$\delta^{65}Cu$	Asia	BB (10)	-0.30 ± 0.45	VE (6)	-0.15 ± 0.09	0.3356		Welch's *t*-test
$\delta^{65}Cu$	Asia	BB (10)	-0.30 ± 0.45	NS (59)	0.09 ± 0.20	0.0246	*	Welch's *t*-test
$\delta^{65}Cu$	Asia	BB (10)	-0.30 ± 0.45	NEE_{BP} (17)	0.15 ± 0.06	0.0007	***	Mann-Whitney test
$\delta^{65}Cu$	Asia	BB (10)	-0.30 ± 0.45	NEE_{T} (3)	-0.49 ± 0.07	0.2161		Welch's *t*-test
$\delta^{65}Cu$	Asia	BB (10)	-0.30 ± 0.45	NEE_{RP} (10)	0.08 ± 0.15	0.0046	**	Mann-Whitney test
$\delta^{65}Cu$	Asia	BB (10)	-0.30 ± 0.45	OE (19)	-0.62 ± 0.80	0.0697		Mann-Whitney test
$\delta^{65}Cu$	Asia	VE (6)	-0.15 ± 0.09	NS (59)	0.09 ± 0.20	0.0003	***	Unpaired Student *t*-test
$\delta^{65}Cu$	Asia	VE (6)	-0.15 ± 0.09	NEE_{BP} (17)	0.15 ± 0.06	0.0004	***	Mann-Whitney test
$\delta^{65}Cu$	Asia	VE (6)	-0.15 ± 0.09	NEE_{T} (3)	-0.49 ± 0.07	0.0013	**	Unpaired Student *t*-test
$\delta^{65}Cu$	Asia	VE (6)	-0.15 ± 0.09	NEE_{RP} (10)	0.08 ± 0.15	0.0016	**	Mann-Whitney test
$\delta^{65}Cu$	Asia	VE (6)	-0.15 ± 0.09	OE (19)	-0.62 ± 0.80	0.0914		Mann-Whitney test
$\delta^{65}Cu$	Asia	NS (59)	0.09 ± 0.20	NEE_{BP} (17)	0.15 ± 0.06	0.0946		Mann-Whitney test
$\delta^{65}Cu$	Asia	NS (59)	0.09 ± 0.20	NEE_{T} (3)	-0.49 ± 0.07	0.0003	***	Unpaired Student *t*-test

$\delta^{65}Cu$	Asia	NS (59)	0.09 ± 0.20	NEE_{RP} (10)	0.08 ± 0.15	0.9932		Mann-Whitney test
$\delta^{65}Cu$	Asia	NS (59)	0.09 ± 0.20	OE (19)	-0.62 ± 0.80	0.0019	**	Mann-Whitney test
$\delta^{65}Cu$	Asia	NEE_{BP} (17)	0.15 ± 0.06	NEE_{T} (3)	-0.49 ± 0.07	0.0078	**	Mann-Whitney test
$\delta^{65}Cu$	Asia	NEE_{BP} (17)	0.15 ± 0.06	NEE_{RP} (10)	0.08 ± 0.15	0.0388	*	Mann-Whitney test
$\delta^{65}Cu$	Asia	NEE_{BP} (17)	0.15 ± 0.06	OE (19)	-0.62 ± 0.80	0.0159	*	Mann-Whitney test
$\delta^{65}Cu$	Asia	NEE_{T} (3)	-0.49 ± 0.07	NEE_{RP} (10)	0.08 ± 0.15	0.0141	*	Mann-Whitney test
$\delta^{65}Cu$	Asia	NEE_{T} (3)	-0.49 ± 0.07	OE (19)	-0.62 ± 0.80	0.2128		Mann-Whitney test
$\delta^{65}Cu$	Asia	NEE_{RP} (10)	0.08 ± 0.15	OE (19)	-0.62 ± 0.80	0.0410	*	Mann-Whitney test
$\delta^{65}Cu$	America	BB (6)	0.04 ± 0.39	VE (3)	-0.01 ± 0.29	0.8475		Unpaired Student *t*-test
$\delta^{65}Cu$	America	BB (6)	0.04 ± 0.39	NS (10)	-0.58 ± 0.78	0.0532		Unpaired Student *t*-test
$\delta^{65}Cu$	America	BB (6)	0.04 ± 0.39	NEE_{BP} (7)	0.12 ± 0.22	0.7206		Mann-Whitney test
$\delta^{65}Cu$	America	BB (6)	0.04 ± 0.39	NEE_{T} (4)	0.03 ± 0.17	0.9604		Unpaired Student *t*-test
$\delta^{65}Cu$	America	BB (6)	0.04 ± 0.39	NEE_{RP} (4)	0.21 ± 0.27	0.4366		Unpaired Student *t*-test
$\delta^{65}Cu$	America	BB (6)	0.04 ± 0.39	OE (4)	-0.14 ± 0.52	0.5969		Unpaired Student *t*-test
$\delta^{65}Cu$	America	VE (3)	-0.01 ± 0.29	NS (10)	-0.58 ± 0.78	0.0853		Unpaired Student *t*-test
$\delta^{65}Cu$	America	VE (3)	-0.01 ± 0.29	NEE_{BP} (7)	0.12 ± 0.22	0.3605		Mann-Whitney test
$\delta^{65}Cu$	America	VE (3)	-0.01 ± 0.29	NEE_{T} (4)	0.03 ± 0.17	0.8552		Unpaired Student *t*-test
$\delta^{65}Cu$	America	VE (3)	-0.01 ± 0.29	NEE_{RP} (4)	0.21 ± 0.27	0.3705		Unpaired Student *t*-test
$\delta^{65}Cu$	America	VE (3)	-0.01 ± 0.29	OE (4)	-0.14 ± 0.52	0.7050		Unpaired Student *t*-test
$\delta^{65}Cu$	America	NS (10)	-0.58 ± 0.78	NEE_{BP} (7)	0.12 ± 0.22	0.1568		Mann-Whitney test
$\delta^{65}Cu$	America	NS (10)	-0.58 ± 0.78	NEE_{T} (4)	0.03 ± 0.17	0.0395	*	Welch's *t*-test
$\delta^{65}Cu$	America	NS (10)	-0.58 ± 0.78	NEE_{RP} (4)	0.21 ± 0.27	0.0160	*	Unpaired Student *t*-test
$\delta^{65}Cu$	America	NS (10)	-0.58 ± 0.78	OE (4)	-0.14 ± 0.52	0.2450		Unpaired Student *t*-test

$\delta^{65}Cu$	America	NEE_{BP} (7)	0.12 ± 0.22	NEE_{T} (4)	0.03 ± 0.17	0.9245		Mann-Whitney test
$\delta^{65}Cu$	America	NEE_{BP} (7)	0.12 ± 0.22	NEE_{RP} (4)	0.21 ± 0.27	0.7763		Mann-Whitney test
$\delta^{65}Cu$	America	NEE_{BP} (7)	0.12 ± 0.22	OE (4)	-0.14 ± 0.52	0.6358		Mann-Whitney test
$\delta^{65}Cu$	America	NEE_{T} (4)	0.03 ± 0.17	NEE_{RP} (4)	0.21 ± 0.27	0.3112		Unpaired Student *t*-test
$\delta^{65}Cu$	America	NEE_{T} (4)	0.03 ± 0.17	OE (4)	-0.14 ± 0.52	0.5881		Unpaired Student *t*-test
$\delta^{65}Cu$	America	NEE_{RP} (4)	0.21 ± 0.27	OE (4)	-0.14 ± 0.52	0.3023		Unpaired Student *t*-test
$\delta^{65}Cu$	Europe	BB (7)	-0.07 ± 0.22	VE (6)	-0.02 ± 0.25	0.7323		Unpaired Student *t*-test
$\delta^{65}Cu$	Europe	BB (7)	-0.07 ± 0.22	NS (15)	-1.85 ± 1.21	< 0.0001	****	Welch's *t*-test
$\delta^{65}Cu$	Europe	BB (7)	-0.07 ± 0.22	NEE_{BP} (6)	0.24 ± 0.32	0.0999		Mann-Whitney test
$\delta^{65}Cu$	Europe	BB (7)	-0.07 ± 0.22	NEE_{T} (3)	0.26 ± 0.08	0.0090	**	Unpaired Student *t*-test
$\delta^{65}Cu$	Europe	BB (7)	-0.07 ± 0.22	NEE_{RP} (4)	0.36 ± 0.39	0.1123		Unpaired Student *t*-test
$\delta^{65}Cu$	Europe	BB (7)	-0.07 ± 0.22	OE (6)	-0.10 ± 1.38	0.9608		Welch's *t*-test
$\delta^{65}Cu$	Europe	VE (6)	-0.02 ± 0.25	NS (15)	-1.85 ± 1.21	< 0.0001	****	Welch's *t*-test
$\delta^{65}Cu$	Europe	VE (6)	-0.02 ± 0.25	NEE_{BP} (6)	0.24 ± 0.32	0.0927		Mann-Whitney test
$\delta^{65}Cu$	Europe	VE (6)	-0.02 ± 0.25	NEE_{T} (3)	0.26 ± 0.08	0.0439	*	Unpaired Student *t*-test
$\delta^{65}Cu$	Europe	VE (6)	-0.02 ± 0.25	NEE_{RP} (4)	0.36 ± 0.39	0.1482		Unpaired Student *t*-test
$\delta^{65}Cu$	Europe	VE (6)	-0.02 ± 0.25	OE (6)	-0.10 ± 1.38	0.9005		Welch's *t*-test
$\delta^{65}Cu$	Europe	NS (15)	-1.85 ± 1.21	NEE_{BP} (6)	0.24 ± 0.32	0.0012	**	Mann-Whitney test
$\delta^{65}Cu$	Europe	NS (15)	-1.85 ± 1.21	NEE_{T} (3)	0.26 ± 0.08	< 0.0001	****	Welch's *t*-test
$\delta^{65}Cu$	Europe	NS (15)	-1.85 ± 1.21	NEE_{RP} (4)	0.36 ± 0.39	< 0.0001	****	Unpaired Student *t*-test
$\delta^{65}Cu$	Europe	NS (15)	-1.85 ± 1.21	OE (6)	-0.10 ± 1.38	0.0253	*	Unpaired Student *t*-test
$\delta^{65}Cu$	Europe	NEE_{BP} (6)	0.24 ± 0.32	NEE_{T} (3)	0.26 ± 0.08	0.8973		Mann-Whitney test
$\delta^{65}Cu$	Europe	NEE_{BP} (6)	0.24 ± 0.32	NEE_{RP} (4)	0.36 ± 0.39	0.7491		Mann-Whitney test

δ^{65}Cu	Europe	NEE_{BP} (6)	0.24 ± 0.32	OE (6)	-0.10 ± 1.38	> 0.9999		Mann-Whitney test
δ^{65}Cu	Europe	NEE_{T} (3)	0.26 ± 0.08	NEE_{RP} (4)	0.36 ± 0.39	0.6378		Unpaired Student *t*-test
δ^{65}Cu	Europe	NEE_{T} (3)	0.26 ± 0.08	OE (6)	-0.10 ± 1.38	0.5604		Welch's *t*-test
δ^{65}Cu	Europe	NEE_{RP} (4)	0.36 ± 0.39	OE (6)	-0.10 ± 1.38	0.4733		Unpaired Student *t*-test
^{207}Pb/^{206}Pb	Asia	CC (239)	0.83983 ± 0.04156	VE_{diesel} (11)	0.86673 ± 0.01951	0.0008	***	Mann-Whitney test
^{207}Pb/^{206}Pb	Asia	CC (239)	0.83983 ± 0.04156	$VE_{gasoline}$ (46)	0.87264 ± 0.01331	< 0.0001	****	Mann-Whitney test
^{207}Pb/^{206}Pb	Asia	CC (239)	0.83983 ± 0.04156	NS (121)	0.84076 ± 0.02940	0.7911		Mann-Whitney test
^{207}Pb/^{206}Pb	Asia	CC (239)	0.83983 ± 0.04156	NEE_{T} (3)	0.86342 ± 0.00246	0.0607		Mann-Whitney test
^{207}Pb/^{206}Pb	Asia	CC (239)	0.83983 ± 0.04156	NEE_{RP} (10)	0.81127 ± 0.06104	0.3455		Mann-Whitney test
^{207}Pb/^{206}Pb	Asia	CC (239)	0.83983 ± 0.04156	NEE_{BP} (17)	0.81921 ± 0.05883	0.0740		Mann-Whitney test
^{207}Pb/^{206}Pb	Asia	CC (239)	0.83983 ± 0.04156	OE (48)	0.86114 ± 0.02821	< 0.0001	****	Mann-Whitney test
^{207}Pb/^{206}Pb	Asia	CC (239)	0.83983 ± 0.04156	WI (11)	0.86466 ± 0.00149	0.0002	***	Mann-Whitney test
^{207}Pb/^{206}Pb	Asia	VE_{diesel} (11)	0.86673 ± 0.01951	$VE_{gasoline}$ (46)	0.87264 ± 0.01331	0.0025	**	Mann-Whitney test
^{207}Pb/^{206}Pb	Asia	VE_{diesel} (11)	0.86673 ± 0.01951	NS (121)	0.84076 ± 0.02940	< 0.0001	****	Mann-Whitney test
^{207}Pb/^{206}Pb	Asia	VE_{diesel} (11)	0.86673 ± 0.01951	NEE_{T} (3)	0.86342 ± 0.00246	0.3481		Mann-Whitney test
^{207}Pb/^{206}Pb	Asia	VE_{diesel} (11)	0.86673 ± 0.01951	NEE_{RP} (10)	0.81127 ± 0.06104	0.0219	*	Mann-Whitney test
^{207}Pb/^{206}Pb	Asia	VE_{diesel} (11)	0.86673 ± 0.01951	NEE_{BP} (17)	0.81921 ± 0.05883	< 0.0001	****	Mann-Whitney test
^{207}Pb/^{206}Pb	Asia	VE_{diesel} (11)	0.86673 ± 0.01951	OE (48)	0.86114 ± 0.02821	0.8229		Mann-Whitney test
^{207}Pb/^{206}Pb	Asia	VE_{diesel} (11)	0.86673 ± 0.01951	WI (11)	0.86466 ± 0.00149	0.0759		Mann-Whitney test
^{207}Pb/^{206}Pb	Asia	$VE_{gasoline}$ (46)	0.87264 ± 0.01331	NS (121)	0.84076 ± 0.02940	< 0.0001	****	Mann-Whitney test
^{207}Pb/^{206}Pb	Asia	$VE_{gasoline}$ (46)	0.87264 ± 0.01331	NEE_{T} (3)	0.86342 ± 0.00246	0.0835		Mann-Whitney test
^{207}Pb/^{206}Pb	Asia	$VE_{gasoline}$ (46)	0.87264 ± 0.01331	NEE_{RP} (10)	0.81127 ± 0.06104	0.0006	***	Mann-Whitney test
^{207}Pb/^{206}Pb	Asia	$VE_{gasoline}$ (46)	0.87264 ± 0.01331	NEE_{BP} (17)	0.81921 ± 0.05883	< 0.0001	****	Mann-Whitney test

$^{207}Pb/^{206}Pb$	Asia	$VE_{gasoline}$ (46)	0.87264 ± 0.01331	OE (48)	0.86114 ± 0.02821	< 0.0001	****	Mann-Whitney test
$^{207}Pb/^{206}Pb$	Asia	$VE_{gasoline}$ (46)	0.87264 ± 0.01331	WI (11)	0.86466 ± 0.00149	0.0044	**	Mann-Whitney test
$^{207}Pb/^{206}Pb$	Asia	NS (121)	0.84076 ± 0.02940	NEE_T (3)	0.86342 ± 0.00246	0.0147	*	Mann-Whitney test
$^{207}Pb/^{206}Pb$	Asia	NS (121)	0.84076 ± 0.02940	NEE_{RP} (10)	0.81127 ± 0.06104	0.6869		Mann-Whitney test
$^{207}Pb/^{206}Pb$	Asia	NS (121)	0.84076 ± 0.02940	NEE_{BP} (17)	0.81921 ± 0.05883	0.1686		Mann-Whitney test
$^{207}Pb/^{206}Pb$	Asia	NS (121)	0.84076 ± 0.02940	OE (48)	0.86114 ± 0.02821	< 0.0001	****	Mann-Whitney test
$^{207}Pb/^{206}Pb$	Asia	NS (121)	0.84076 ± 0.02940	WI (11)	0.86466 ± 0.00149	< 0.0001	****	Mann-Whitney test
$^{207}Pb/^{206}Pb$	Asia	NEE_T (3)	0.86342 ± 0.00246	NEE_{RP} (10)	0.81127 ± 0.06104	0.0244	*	Welch's *t*-test
$^{207}Pb/^{206}Pb$	Asia	NEE_T (3)	0.86342 ± 0.00246	NEE_{BP} (17)	0.81921 ± 0.05883	0.0081	**	Mann-Whitney test
$^{207}Pb/^{206}Pb$	Asia	NEE_T (3)	0.86342 ± 0.00246	OE (48)	0.86114 ± 0.02821	0.9840		Mann-Whitney test
$^{207}Pb/^{206}Pb$	Asia	NEE_T (3)	0.86342 ± 0.00246	WI (11)	0.86466 ± 0.00149	0.4805		Unpaired Student *t*-test
$^{207}Pb/^{206}Pb$	Asia	NEE_{RP} (10)	0.81127 ± 0.06104	NEE_{BP} (17)	0.81921 ± 0.05883	0.8802		Mann-Whitney test
$^{207}Pb/^{206}Pb$	Asia	NEE_{RP} (10)	0.81127 ± 0.06104	OE (48)	0.86114 ± 0.02821	0.0195	*	Mann-Whitney test
$^{207}Pb/^{206}Pb$	Asia	NEE_{RP} (10)	0.81127 ± 0.06104	WI (11)	0.86466 ± 0.00149	0.0219	*	Welch's *t*-test
$^{207}Pb/^{206}Pb$	Asia	NEE_{BP} (17)	0.81921 ± 0.05883	OE (48)	0.86114 ± 0.02821	< 0.0001	****	Mann-Whitney test
$^{207}Pb/^{206}Pb$	Asia	NEE_{BP} (17)	0.81921 ± 0.05883	WI (11)	0.86466 ± 0.00149	< 0.0001	****	Mann-Whitney test
$^{207}Pb/^{206}Pb$	Asia	OE (48)	0.86114 ± 0.02821	WI (11)	0.86466 ± 0.00149	0.3706		Mann-Whitney test
$^{207}Pb/^{206}Pb$	America	CC (4)	0.72775 ± 0.13658	VE_{diesel} (4)	0.86433 ± 0.01561	0.1388		Welch's *t*-test
$^{207}Pb/^{206}Pb$	America	CC (4)	0.72775 ± 0.13658	$VE_{gasoline}$ (9)	0.87722 ± 0.02096	0.1158		Welch's *t*-test
$^{207}Pb/^{206}Pb$	America	CC (4)	0.72775 ± 0.13658	NS (6)	0.85359 ± 0.00707	0.2410		Mann-Whitney test
$^{207}Pb/^{206}Pb$	America	CC (4)	0.72775 ± 0.13658	NEE_T (6)	0.85098 ± 0.00980	0.1688		Welch's *t*-test
$^{207}Pb/^{206}Pb$	America	CC (4)	0.72775 ± 0.13658	NEE_{RP} (4)	0.82888 ± 0.04395	0.2386		Unpaired Student *t*-test
$^{207}Pb/^{206}Pb$	America	CC (4)	0.72775 ± 0.13658	NEE_{BP} (3)	0.72821 ± 0.18611	0.9973		Unpaired Student *t*-test

$^{207}Pb/^{206}Pb$	America	CC (4)	0.72775 ± 0.13658	OE (7)	0.86791 ± 0.00048	0.1324		Welch's *t*-test
$^{207}Pb/^{206}Pb$	America	CC (4)	0.72775 ± 0.13658	WI (6)	0.86917 ± 0.00214	0.1301		Welch's *t*-test
$^{207}Pb/^{206}Pb$	America	VE_{diesel} (4)	0.86433 ± 0.01561	$VE_{gasoline}$ (9)	0.87722 ± 0.02096	0.2540		Unpaired Student *t*-test
$^{207}Pb/^{206}Pb$	America	VE_{diesel} (4)	0.86433 ± 0.01561	NS (6)	0.85359 ± 0.00707	0.1658		Mann-Whitney test
$^{207}Pb/^{206}Pb$	America	VE_{diesel} (4)	0.86433 ± 0.01561	NEE_T (6)	0.85098 ± 0.00980	0.1936		Unpaired Student *t*-test
$^{207}Pb/^{206}Pb$	America	VE_{diesel} (4)	0.86433 ± 0.01561	NEE_{RP} (4)	0.82888 ± 0.04395	0.2079		Unpaired Student *t*-test
$^{207}Pb/^{206}Pb$	America	VE_{diesel} (4)	0.86433 ± 0.01561	NEE_{BP} (3)	0.72821 ± 0.18611	0.3326		Welch's *t*-test
$^{207}Pb/^{206}Pb$	America	VE_{diesel} (4)	0.86433 ± 0.01561	OE (7)	0.86791 ± 0.00048	0.6770		Welch's *t*-test
$^{207}Pb/^{206}Pb$	America	VE_{diesel} (4)	0.86433 ± 0.01561	WI (6)	0.86917 ± 0.00214	0.5803		Welch's *t*-test
$^{207}Pb/^{206}Pb$	America	$VE_{gasoline}$ (9)	0.87722 ± 0.02096	NS (6)	0.85359 ± 0.00707	0.0112	*	Mann-Whitney test
$^{207}Pb/^{206}Pb$	America	$VE_{gasoline}$ (9)	0.87722 ± 0.02096	NEE_T (6)	0.85098 ± 0.00980	0.0068	**	Unpaired Student *t*-test
$^{207}Pb/^{206}Pb$	America	$VE_{gasoline}$ (9)	0.87722 ± 0.02096	NEE_{RP} (4)	0.82888 ± 0.04395	0.1113		Unpaired Student *t*-test
$^{207}Pb/^{206}Pb$	America	$VE_{gasoline}$ (9)	0.87722 ± 0.02096	NEE_{BP} (3)	0.72821 ± 0.18611	0.2996		Welch's *t*-test
$^{207}Pb/^{206}Pb$	America	$VE_{gasoline}$ (9)	0.87722 ± 0.02096	OE (7)	0.86791 ± 0.00048	0.2197		Welch's *t*-test
$^{207}Pb/^{206}Pb$	America	$VE_{gasoline}$ (9)	0.87722 ± 0.02096	WI (6)	0.86917 ± 0.00214	0.2849		Welch's *t*-test
$^{207}Pb/^{206}Pb$	America	NS (6)	0.85359 ± 0.00707	NEE_T (6)	0.85098 ± 0.00980	0.6889		Mann-Whitney test
$^{207}Pb/^{206}Pb$	America	NS (6)	0.85359 ± 0.00707	NEE_{RP} (4)	0.82888 ± 0.04395	0.3374		Mann-Whitney test
$^{207}Pb/^{206}Pb$	America	NS (6)	0.85359 ± 0.00707	NEE_{BP} (3)	0.72821 ± 0.18611	0.0528		Mann-Whitney test
$^{207}Pb/^{206}Pb$	America	NS (6)	0.85359 ± 0.00707	OE (7)	0.86791 ± 0.00048	0.0034	**	Mann-Whitney test
$^{207}Pb/^{206}Pb$	America	NS (6)	0.85359 ± 0.00707	WI (6)	0.86917 ± 0.00214	0.0050	**	Mann-Whitney test
$^{207}Pb/^{206}Pb$	America	NEE_T (6)	0.85098 ± 0.00980	NEE_{RP} (4)	0.82888 ± 0.04395	0.3912		Welch's *t*-test
$^{207}Pb/^{206}Pb$	America	NEE_T (6)	0.85098 ± 0.00980	NEE_{BP} (3)	0.72821 ± 0.18611	0.3716		Welch's *t*-test
$^{207}Pb/^{206}Pb$	America	NEE_T (6)	0.85098 ± 0.00980	OE (7)	0.86791 ± 0.00048	0.0082	**	Welch's *t*-test

$^{207}Pb/^{206}Pb$	America	NEE_T (6)	0.85098 ± 0.00980	WI (6)	0.86917 ± 0.00214	0.0054	**	Welch's *t*-test
$^{207}Pb/^{206}Pb$	America	NEE_{RP} (4)	0.82888 ± 0.04395	NEE_{BP} (3)	0.72821 ± 0.18611	0.4491		Welch's *t*-test
$^{207}Pb/^{206}Pb$	America	NEE_{RP} (4)	0.82888 ± 0.04395	OE (7)	0.86791 ± 0.00048	0.1737		Welch's *t*-test
$^{207}Pb/^{206}Pb$	America	NEE_{RP} (4)	0.82888 ± 0.04395	WI (6)	0.86917 ± 0.00214	0.1641		Welch's *t*-test
$^{207}Pb/^{206}Pb$	America	NEE_{BP} (3)	0.72821 ± 0.18611	OE (7)	0.86791 ± 0.00048	0.3232		Welch's *t*-test
$^{207}Pb/^{206}Pb$	America	NEE_{BP} (3)	0.72821 ± 0.18611	WI (6)	0.86917 ± 0.00214	0.3199		Welch's *t*-test
$^{207}Pb/^{206}Pb$	America	OE (7)	0.86791 ± 0.00048	WI (6)	0.86917 ± 0.00214	0.2144		Welch's *t*-test
$^{207}Pb/^{206}Pb$	Europe	CC (4)	0.78750 ± 0.10379	VE_{diesel} (3)	0.86292 ± 0.00056	0.2421		Welch's *t*-test
$^{207}Pb/^{206}Pb$	Europe	CC (4)	0.78750 ± 0.10379	$VE_{gasoline}$ (4)	0.90536 ± 0.01471	0.1066		Welch's *t*-test
$^{207}Pb/^{206}Pb$	Europe	CC (4)	0.78750 ± 0.10379	NS (16)	0.85560 ± 0.00468	0.1405		Mann-Whitney test
$^{207}Pb/^{206}Pb$	Europe	CC (4)	0.78750 ± 0.10379	NEE_T (4)	0.86157 ± 0.00544	0.2488		Welch's *t*-test
$^{207}Pb/^{206}Pb$	Europe	CC (4)	0.78750 ± 0.10379	NEE_{RP} (4)	0.80725 ± 0.04737	0.7458		Unpaired Student *t*-test
$^{207}Pb/^{206}Pb$	Europe	CC (4)	0.78750 ± 0.10379	NEE_{BP} (3)	0.82499 ± 0.03099	0.5352		Unpaired Student *t*-test
$^{207}Pb/^{206}Pb$	Europe	CC (4)	0.78750 ± 0.10379	OE (6)	0.86792 ± 0.00044	0.2190		Welch's *t*-test
$^{207}Pb/^{206}Pb$	Europe	CC (4)	0.78750 ± 0.10379	WI (12)	0.86977 ± 0.00329	0.2111		Welch's *t*-test
$^{207}Pb/^{206}Pb$	Europe	VE_{diesel} (3)	0.86292 ± 0.00056	$VE_{gasoline}$ (4)	0.90536 ± 0.01471	0.0103	*	Welch's *t*-test
$^{207}Pb/^{206}Pb$	Europe	VE_{diesel} (3)	0.86292 ± 0.00056	NS (16)	0.85560 ± 0.00468	0.0081	**	Mann-Whitney test
$^{207}Pb/^{206}Pb$	Europe	VE_{diesel} (3)	0.86292 ± 0.00056	NEE_T (4)	0.86157 ± 0.00544	0.6566		Welch's *t*-test
$^{207}Pb/^{206}Pb$	Europe	VE_{diesel} (3)	0.86292 ± 0.00056	NEE_{RP} (4)	0.80725 ± 0.04737	0.1003		Welch's *t*-test
$^{207}Pb/^{206}Pb$	Europe	VE_{diesel} (3)	0.86292 ± 0.00056	NEE_{BP} (3)	0.82499 ± 0.03099	0.1681		Welch's *t*-test
$^{207}Pb/^{206}Pb$	Europe	VE_{diesel} (3)	0.86292 ± 0.00056	OE (6)	0.86792 ± 0.00044	0.0005	***	Unpaired Student *t*-test
$^{207}Pb/^{206}Pb$	Europe	VE_{diesel} (3)	0.86292 ± 0.00056	WI (12)	0.86977 ± 0.00329	< 0.0001	****	Unpaired Student *t*-test
$^{207}Pb/^{206}Pb$	Europe	$VE_{gasoline}$ (4)	0.90536 ± 0.01471	NS (16)	0.85560 ± 0.00468	0.0027	**	Mann-Whitney test

$^{207}Pb/^{206}Pb$	Europe	$VE_{gasoline}$ (4)	0.90536 ± 0.01471	NEE_{T} (4)	0.86157 ± 0.00544	0.0058	**	Unpaired Student *t*-test
$^{207}Pb/^{206}Pb$	Europe	$VE_{gasoline}$ (4)	0.90536 ± 0.01471	NEE_{RP} (4)	0.80725 ± 0.04737	0.0208	*	Unpaired Student *t*-test
$^{207}Pb/^{206}Pb$	Europe	$VE_{gasoline}$ (4)	0.90536 ± 0.01471	NEE_{BP} (3)	0.82499 ± 0.03099	0.0314	*	Unpaired Student *t*-test
$^{207}Pb/^{206}Pb$	Europe	$VE_{gasoline}$ (4)	0.90536 ± 0.01471	OE (6)	0.86792 ± 0.00044	0.0146	*	Welch's *t*-test
$^{207}Pb/^{206}Pb$	Europe	$VE_{gasoline}$ (4)	0.90536 ± 0.01471	WI (12)	0.86977 ± 0.00329	0.0160	*	Welch's *t*-test
$^{207}Pb/^{206}Pb$	Europe	NS (16)	0.85560 ± 0.00468	NEE_{T} (4)	0.86157 ± 0.00544	0.2162		Mann-Whitney test
$^{207}Pb/^{206}Pb$	Europe	NS (16)	0.85560 ± 0.00468	NEE_{RP} (4)	0.80725 ± 0.04737	0.1405		Mann-Whitney test
$^{207}Pb/^{206}Pb$	Europe	NS (16)	0.85560 ± 0.00468	NEE_{BP} (3)	0.82499 ± 0.03099	0.0631		Mann-Whitney test
$^{207}Pb/^{206}Pb$	Europe	NS (16)	0.85560 ± 0.00468	OE (6)	0.86792 ± 0.00044	0.0004	***	Mann-Whitney test
$^{207}Pb/^{206}Pb$	Europe	NS (16)	0.85560 ± 0.00468	WI (12)	0.86977 ± 0.00329	< 0.0001	****	Mann-Whitney test
$^{207}Pb/^{206}Pb$	Europe	NEE_{T} (4)	0.86157 ± 0.00544	NEE_{RP} (4)	0.80725 ± 0.04737	0.1048		Welch's *t*-test
$^{207}Pb/^{206}Pb$	Europe	NEE_{T} (4)	0.86157 ± 0.00544	NEE_{BP} (3)	0.82499 ± 0.03099	0.1750		Welch's *t*-test
$^{207}Pb/^{206}Pb$	Europe	NEE_{T} (4)	0.86157 ± 0.00544	OE (6)	0.86792 ± 0.00044	0.1017		Welch's *t*-test
$^{207}Pb/^{206}Pb$	Europe	NEE_{T} (4)	0.86157 ± 0.00544	WI (12)	0.86977 ± 0.00329	0.0502		Unpaired Student *t*-test
$^{207}Pb/^{206}Pb$	Europe	NEE_{RP} (4)	0.80725 ± 0.04737	NEE_{BP} (3)	0.82499 ± 0.03099	0.5763		Unpaired Student *t*-test
$^{207}Pb/^{206}Pb$	Europe	NEE_{RP} (4)	0.80725 ± 0.04737	OE (6)	0.86792 ± 0.00044	0.0831		Welch's *t*-test
$^{207}Pb/^{206}Pb$	Europe	NEE_{RP} (4)	0.80725 ± 0.04737	WI (12)	0.86977 ± 0.00329	0.0776		Welch's *t*-test
$^{207}Pb/^{206}Pb$	Europe	NEE_{BP} (3)	0.82499 ± 0.03099	OE (6)	0.86792 ± 0.00044	0.1385		Welch's *t*-test
$^{207}Pb/^{206}Pb$	Europe	NEE_{BP} (3)	0.82499 ± 0.03099	WI (12)	0.86977 ± 0.00329	0.1290		Welch's *t*-test
$^{207}Pb/^{206}Pb$	Europe	OE (6)	0.86792 ± 0.00044	WI (12)	0.86977 ± 0.00329	0.0805		Welch's *t*-test
$\delta^{144}Nd$	Asia	CC (7)	1.20 ± 0.36	VE (8)	0.97 ± 0.34	0.2140		Unpaired Student *t*-test
$\delta^{144}Nd$	Asia	CC (7)	1.20 ± 0.36	NS (31)	1.19 ± 0.48	0.6513		Mann-Whitney test
$\delta^{144}Nd$	Asia	CC (7)	1.20 ± 0.36	NEE (16)	1.48 ± 0.09	0.0880		Welch's *t*-test

δ^{144}Nd	Asia	CC (7)	1.20 ± 0.36	WI (3)	0.44 ± 0.17	0.0022	**	Unpaired Student *t*-test
δ^{144}Nd	Asia	VE (8)	0.97 ± 0.34	NS (31)	1.19 ± 0.48	0.0732		Mann-Whitney test
δ^{144}Nd	Asia	VE (8)	0.97 ± 0.34	NEE (16)	1.48 ± 0.09	0.0035	**	Welch's *t*-test
δ^{144}Nd	Asia	VE (8)	0.97 ± 0.34	WI (3)	0.44 ± 0.17	0.0111	*	Unpaired Student *t*-test
δ^{144}Nd	Asia	NS (31)	1.19 ± 0.48	NEE (16)	1.48 ± 0.09	0.1324		Mann-Whitney test
δ^{144}Nd	Asia	NS (31)	1.19 ± 0.48	WI (3)	0.44 ± 0.17	0.0335	*	Mann-Whitney test
δ^{144}Nd	Asia	NEE (16)	1.48 ± 0.09	WI (3)	0.44 ± 0.17	0.0069	**	Unpaired Student *t*-test
δ^{144}Nd	America	CC (4)	1.06 ± 0.24	VE (4)	0.19 ± 0.98	0.1709		Welch's *t*-test
δ^{144}Nd	America	CC (4)	1.06 ± 0.24	NS (6)	-0.39 ± 1.12	0.1098		Mann-Whitney test
δ^{144}Nd	America	CC (4)	1.06 ± 0.24	NEE (3)	0.91 ± 0.07	0.3052		Unpaired Student *t*-test
δ^{144}Nd	America	CC (4)	1.06 ± 0.24	WI (4)	0.77 ± 0.22	0.1259		Unpaired Student *t*-test
δ^{144}Nd	America	VE (4)	0.19 ± 0.98	NS (6)	-0.39 ± 1.12	0.2410		Mann-Whitney test
δ^{144}Nd	America	VE (4)	0.19 ± 0.98	NEE (3)	0.91 ± 0.07	0.2359		Welch's *t*-test
δ^{144}Nd	America	VE (4)	0.19 ± 0.98	WI (4)	0.77 ± 0.22	0.3221		Welch's *t*-test
δ^{144}Nd	America	NS (6)	-0.39 ± 1.12	NEE (3)	0.91 ± 0.07	0.5186		Mann-Whitney test
δ^{144}Nd	America	NS (6)	-0.39 ± 1.12	WI (4)	0.77 ± 0.22	0.4542		Mann-Whitney test
δ^{144}Nd	America	NEE (3)	0.91 ± 0.07	WI (4)	0.77 ± 0.22	0.3068		Unpaired Student *t*-test
δ^{144}Nd	Europe	CC (4)	0.92 ± 0.33	VE (6)	0.17 ± 0.85	0.1098		Mann-Whitney test
δ^{144}Nd	Europe	CC (4)	0.92 ± 0.33	NS (3)	1.04 ± 0.08	0.3768		Mann-Whitney test
δ^{144}Nd	Europe	CC (4)	0.92 ± 0.33	NEE (3)	1.08 ± 0.06	0.3768		Mann-Whitney test
δ^{144}Nd	Europe	CC (4)	0.92 ± 0.33	WI (10)	0.98 ± 0.11	0.1762		Mann-Whitney test
δ^{144}Nd	Europe	VE (6)	0.17 ± 0.85	NS (3)	1.04 ± 0.08	0.0282	*	Mann-Whitney test
δ^{144}Nd	Europe	VE (6)	0.17 ± 0.85	NEE (3)	1.08 ± 0.06	0.0282	*	Mann-Whitney test
δ^{144}Nd	Europe	VE (6)	0.17 ± 0.85	WI (10)	0.98 ± 0.11	0.0013	**	Mann-Whitney test
δ^{144}Nd	Europe	NS (3)	1.04 ± 0.08	NEE (3)	1.08 ± 0.06	0.5530		Unpaired Student *t*-test

δ^{144}Nd	Europe	NS (3)	1.04 ± 0.08	WI (10)	0.98 ± 0.11	0.2679		Mann-Whitney test
δ^{144}Nd	Europe	NEE (3)	1.08 ± 0.06	WI (10)	0.98 ± 0.11	0.0735		Mann-Whitney test
δ^{87}Sr	Asia	CC (9)	20.93 ± 20.93	VE (8)	27.89 ± 27.89	0.0750		Mann-Whitney test
δ^{87}Sr	Asia	CC (9)	20.93 ± 20.93	NS (67)	23.24 ± 23.24	0.0600		Mann-Whitney test
δ^{87}Sr	Asia	CC (9)	20.93 ± 20.93	NEE (20)	14.98 ± 14.98	0.2478		Mann-Whitney test
δ^{87}Sr	Asia	CC (9)	20.93 ± 20.93	OE (3)	18.18 ± 18.18	0.0645		Mann-Whitney test
δ^{87}Sr	Asia	VE (8)	27.89 ± 27.89	NS (67)	23.24 ± 23.24	0.2911		Mann-Whitney test
δ^{87}Sr	Asia	VE (8)	27.89 ± 27.89	NEE (20)	14.98 ± 14.98	0.0047	**	Mann-Whitney test
δ^{87}Sr	Asia	VE (8)	27.89 ± 27.89	OE (3)	18.18 ± 18.18	0.0580		Welch's *t*-test
δ^{87}Sr	Asia	NS (67)	23.24 ± 23.24	NEE (20)	14.98 ± 14.98	0.0001	***	Mann-Whitney test
δ^{87}Sr	Asia	NS (67)	23.24 ± 23.24	OE (3)	18.18 ± 18.18	0.2898		Mann-Whitney test
δ^{87}Sr	Asia	NEE (20)	14.98 ± 14.98	OE (3)	18.18 ± 18.18	0.0070	**	Mann-Whitney test
δ^{87}Sr	America	CC (4)	26.14 ± 26.14	VE (4)	20.26 ± 20.26	0.1979		Unpaired Student *t*-test
δ^{87}Sr	America	CC (4)	26.14 ± 26.14	NS (9)	16.53 ± 16.53	0.0554		Unpaired Student *t*-test
δ^{87}Sr	America	CC (4)	26.14 ± 26.14	NEE (4)	15.13 ± 15.13	0.0427	*	Welch's *t*-test
δ^{87}Sr	America	CC (4)	26.14 ± 26.14	OE (6)	17.47 ± 17.47	0.0808		Welch's *t*-test
δ^{87}Sr	America	VE (4)	20.26 ± 20.26	NS (9)	16.53 ± 16.53	0.2002		Unpaired Student *t*-test
δ^{87}Sr	America	VE (4)	20.26 ± 20.26	NEE (4)	15.13 ± 15.13	0.0912		Unpaired Student *t*-test
δ^{87}Sr	America	VE (4)	20.26 ± 20.26	OE (6)	17.47 ± 17.47	0.2828		Welch's *t*-test
δ^{87}Sr	America	NS (9)	16.53 ± 16.53	NEE (4)	15.13 ± 15.13	0.4125		Unpaired Student *t*-test
δ^{87}Sr	America	NS (9)	16.53 ± 16.53	OE (6)	17.47 ± 17.47	0.5415		Welch's *t*-test
δ^{87}Sr	America	NEE (4)	15.13 ± 15.13	OE (6)	17.47 ± 17.47	0.0521		Welch's *t*-test
δ^{87}Sr	Europe	CC (4)	34.44 ± 34.44	VE (8)	19.03 ± 19.03	0.0334	*	Mann-Whitney test
δ^{87}Sr	Europe	CC (4)	34.44 ± 34.44	NS (6)	24.57 ± 24.57	0.2461		Unpaired Student *t*-test
δ^{87}Sr	Europe	CC (4)	34.44 ± 34.44	NEE (5)	15.08 ± 15.08	0.0408	*	Welch's *t*-test

δ^{87}Sr	Europe	CC (4)	34.44 ± 34.44	OE (3)	17.60 ± 17.60	0.0579		Welch's *t*-test
δ^{87}Sr	Europe	VE (8)	19.03 ± 19.03	NS (6)	24.57 ± 24.57	0.4772		Mann-Whitney test
δ^{87}Sr	Europe	VE (8)	19.03 ± 19.03	NEE (5)	15.08 ± 15.08	0.6079		Mann-Whitney test
δ^{87}Sr	Europe	VE (8)	19.03 ± 19.03	OE (3)	17.60 ± 17.60	0.6090		Mann-Whitney test
δ^{87}Sr	Europe	NS (6)	24.57 ± 24.57	NEE (5)	15.08 ± 15.08	0.1420		Welch's *t*-test
δ^{87}Sr	Europe	NS (6)	24.57 ± 24.57	OE (3)	17.60 ± 17.60	0.2565		Welch's *t*-test
δ^{87}Sr	Europe	NEE (5)	15.08 ± 15.08	OE (3)	17.60 ± 17.60	0.0028	**	Unpaired Student *t*-test
δ^{56}Fe	Asia	BB (5)	-0.41 ± 0.49	CC (4)	-0.16 ± 0.36	0.4114		Unpaired Student *t*-test
δ^{56}Fe	Asia	BB (5)	-0.41 ± 0.49	VE (5)	0.21 ± 0.09	0.0479	*	Welch's *t*-test
δ^{56}Fe	Asia	BB (5)	-0.41 ± 0.49	NS (4)	0.04 ± 0.12	0.1147		Welch's *t*-test
δ^{56}Fe	Asia	BB (5)	-0.41 ± 0.49	NEE (5)	0.23 ± 0.31	0.0438	*	Unpaired Student *t*-test
δ^{56}Fe	Asia	BB (5)	-0.41 ± 0.49	OE (5)	0.62 ± 0.62	0.0209	*	Unpaired Student *t*-test
δ^{56}Fe	Asia	CC (4)	-0.16 ± 0.36	VE (5)	0.21 ± 0.09	0.1331		Welch's *t*-test
δ^{56}Fe	Asia	CC (4)	-0.16 ± 0.36	NS (4)	0.04 ± 0.12	0.3677		Unpaired Student *t*-test
δ^{56}Fe	Asia	CC (4)	-0.16 ± 0.36	NEE (5)	0.23 ± 0.31	0.1327		Unpaired Student *t*-test
δ^{56}Fe	Asia	CC (4)	-0.16 ± 0.36	OE (5)	0.62 ± 0.62	0.0531		Unpaired Student *t*-test
δ^{56}Fe	Asia	VE (5)	0.21 ± 0.09	NS (4)	0.04 ± 0.12	0.0612		Unpaired Student *t*-test
δ^{56}Fe	Asia	VE (5)	0.21 ± 0.09	NEE (5)	0.23 ± 0.31	0.8550		Welch's *t*-test
δ^{56}Fe	Asia	VE (5)	0.21 ± 0.09	OE (5)	0.62 ± 0.62	0.2114		Welch's *t*-test
δ^{56}Fe	Asia	NS (4)	0.04 ± 0.12	NEE (5)	0.23 ± 0.31	0.2370		Unpaired Student *t*-test
δ^{56}Fe	Asia	NS (4)	0.04 ± 0.12	OE (5)	0.62 ± 0.62	0.1025		Welch's *t*-test
δ^{56}Fe	Asia	NEE (5)	0.23 ± 0.31	OE (5)	0.62 ± 0.62	0.2598		Unpaired Student *t*-test
δ^{56}Fe	America	BB (5)	-0.54 ± 0.28	CC (3)	0.34 ± 0.37	0.0290	*	Unpaired Student *t*-test
δ^{56}Fe	America	BB (5)	-0.54 ± 0.28	VE (6)	0.12 ± 0.07	0.0055	**	Welch's *t*-test
δ^{56}Fe	America	BB (5)	-0.54 ± 0.28	NS (8)	-0.01 ± 0.06	0.0123	*	Welch's *t*-test

δ^{56}Fe	America	BB (5)	-0.54 ± 0.28	NEE (12)	0.12 ± 0.21	0.0034	**	Unpaired Student *t*-test
δ^{56}Fe	America	BB (5)	-0.54 ± 0.28	OE (6)	0.41 ± 0.87	0.0225	*	Mann-Whitney test
δ^{56}Fe	America	CC (3)	0.34 ± 0.37	VE (6)	0.12 ± 0.07	0.4023		Welch's *t*-test
δ^{56}Fe	America	CC (3)	0.34 ± 0.37	NS (8)	-0.01 ± 0.06	0.2424		Welch's *t*-test
δ^{56}Fe	America	CC (3)	0.34 ± 0.37	NEE (12)	0.12 ± 0.21	0.4073		Unpaired Student *t*-test
δ^{56}Fe	America	CC (3)	0.34 ± 0.37	OE (6)	0.41 ± 0.87	0.5186		Mann-Whitney test
δ^{56}Fe	America	VE (6)	0.12 ± 0.07	NS (8)	-0.01 ± 0.06	0.0057	**	Unpaired Student *t*-test
δ^{56}Fe	America	VE (6)	0.12 ± 0.07	NEE (12)	0.12 ± 0.21	0.9776		Welch's *t*-test
δ^{56}Fe	America	VE (6)	0.12 ± 0.07	OE (6)	0.41 ± 0.87	0.3785		Mann-Whitney test
δ^{56}Fe	America	NS (8)	-0.01 ± 0.06	NEE (12)	0.12 ± 0.21	0.0784		Welch's *t*-test
δ^{56}Fe	America	NS (8)	-0.01 ± 0.06	OE (6)	0.41 ± 0.87	0.3651		Mann-Whitney test
δ^{56}Fe	America	NEE (12)	0.12 ± 0.21	OE (6)	0.41 ± 0.87	0.4260		Mann-Whitney test
δ^{56}Fe	Europe	BB (90)	-0.66 ± 0.79	CC (3)	0.07 ± 0.01	0.0529		Mann-Whitney test
δ^{56}Fe	Europe	BB (90)	-0.66 ± 0.79	VE (3)	0.16 ± 0.11	0.0289	*	Mann-Whitney test
δ^{56}Fe	Europe	BB (90)	-0.66 ± 0.79	NS (4)	-0.01 ± 0.04	0.0610		Mann-Whitney test
δ^{56}Fe	Europe	BB (90)	-0.66 ± 0.79	NEE (4)	0.13 ± 0.15	0.0187	*	Mann-Whitney test
δ^{56}Fe	Europe	BB (90)	-0.66 ± 0.79	OE (6)	0.58 ± 0.44	0.0003	***	Mann-Whitney test
δ^{56}Fe	Europe	CC (3)	0.07 ± 0.01	VE (3)	0.16 ± 0.11	0.2927		Welch's *t*-test
δ^{56}Fe	Europe	CC (3)	0.07 ± 0.01	NS (4)	-0.01 ± 0.04	0.0239	*	Welch's *t*-test
δ^{56}Fe	Europe	CC (3)	0.07 ± 0.01	NEE (4)	0.13 ± 0.15	0.4526		Welch's *t*-test
δ^{56}Fe	Europe	CC (3)	0.07 ± 0.01	OE (6)	0.58 ± 0.44	0.0350	*	Welch's *t*-test
δ^{56}Fe	Europe	VE (3)	0.16 ± 0.11	NS (4)	-0.01 ± 0.04	0.1204		Unpaired Student *t*-test
δ^{56}Fe	Europe	VE (3)	0.16 ± 0.11	NEE (4)	0.13 ± 0.15	0.7878		Unpaired Student *t*-test
δ^{56}Fe	Europe	VE (3)	0.16 ± 0.11	OE (6)	0.58 ± 0.44	0.0692		Unpaired Student *t*-test
δ^{56}Fe	Europe	NS (4)	-0.01 ± 0.04	NEE (4)	0.13 ± 0.15	0.1651		Welch's *t*-test

δ^{56}Fe	Europe	NS (4)	-0.01 ± 0.04	OE (6)	0.58 ± 0.44	0.0218	*	Welch's *t*-test
δ^{56}Fe	Europe	NEE (4)	0.13 ± 0.15	OE (6)	0.58 ± 0.44	0.0568		Unpaired Student *t*-test
δ^{66}Zn	Asia	BB (7)	0.42 ± 0.23	CC (5)	0.25 ± 0.23	0.2426		Unpaired Student *t*-test
δ^{66}Zn	Asia	BB (7)	0.42 ± 0.23	VE (74)	-0.14 ± 0.15	< 0.0001	****	Mann-Whitney test
δ^{66}Zn	Asia	BB (7)	0.42 ± 0.23	NS (39)	0.54 ± 0.16	0.2198		Unpaired Student *t*-test
δ^{66}Zn	Asia	BB (7)	0.42 ± 0.23	NEE_{BP} (17)	-0.03 ± 0.09	0.0019	**	Welch's *t*-test
δ^{66}Zn	Asia	BB (7)	0.42 ± 0.23	NEE_{T} (5)	-0.01 ± 0.06	0.0024	**	Welch's *t*-test
δ^{66}Zn	Asia	BB (7)	0.42 ± 0.23	WI (5)	0.21 ± 0.09	0.0638		Unpaired Student *t*-test
δ^{66}Zn	Asia	BB (7)	0.42 ± 0.23	NEE_{RP} (10)	-0.14 ± 0.26	0.0007	***	Mann-Whitney test
δ^{66}Zn	Asia	BB (7)	0.42 ± 0.23	OE (5)	0.03 ± 0.34	0.0659		Unpaired Student *t*-test
δ^{66}Zn	Asia	CC (5)	0.25 ± 0.23	VE (74)	-0.14 ± 0.15	0.0008	***	Mann-Whitney test
δ^{66}Zn	Asia	CC (5)	0.25 ± 0.23	NS (39)	0.54 ± 0.16	0.0428	*	Unpaired Student *t*-test
δ^{66}Zn	Asia	CC (5)	0.25 ± 0.23	NEE_{BP} (17)	-0.03 ± 0.09	0.0485	*	Welch's *t*-test
δ^{66}Zn	Asia	CC (5)	0.25 ± 0.23	NEE_{T} (5)	-0.01 ± 0.06	0.0615		Welch's *t*-test
δ^{66}Zn	Asia	CC (5)	0.25 ± 0.23	WI (5)	0.21 ± 0.09	0.7335		Unpaired Student *t*-test
δ^{66}Zn	Asia	CC (5)	0.25 ± 0.23	NEE_{RP} (10)	-0.14 ± 0.26	0.0119	*	Mann-Whitney test
δ^{66}Zn	Asia	CC (5)	0.25 ± 0.23	OE (5)	0.03 ± 0.34	0.2664		Unpaired Student *t*-test
δ^{66}Zn	Asia	VE (74)	-0.14 ± 0.15	NS (39)	0.54 ± 0.16	< 0.0001	****	Mann-Whitney test
δ^{66}Zn	Asia	VE (74)	-0.14 ± 0.15	NEE_{BP} (17)	-0.03 ± 0.09	< 0.0001	****	Mann-Whitney test
δ^{66}Zn	Asia	VE (74)	-0.14 ± 0.15	NEE_{T} (5)	-0.01 ± 0.06	0.0022	**	Mann-Whitney test
δ^{66}Zn	Asia	VE (74)	-0.14 ± 0.15	WI (5)	0.21 ± 0.09	0.0009	***	Mann-Whitney test
δ^{66}Zn	Asia	VE (74)	-0.14 ± 0.15	NEE_{RP} (10)	-0.14 ± 0.26	0.1354		Mann-Whitney test
δ^{66}Zn	Asia	VE (74)	-0.14 ± 0.15	OE (5)	0.03 ± 0.34	0.5387		Mann-Whitney test
δ^{66}Zn	Asia	NS (39)	0.54 ± 0.16	NEE_{BP} (17)	-0.03 ± 0.09	< 0.0001	****	Welch's *t*-test

δ^{66}Zn	Asia	NS (39)	0.54 ± 0.16	NEE_T (5)	-0.01 ± 0.06	< 0.0001	****	Unpaired Student *t*-test
δ^{66}Zn	Asia	NS (39)	0.54 ± 0.16	WI (5)	0.21 ± 0.09	0.0001	***	Unpaired Student *t*-test
δ^{66}Zn	Asia	NS (39)	0.54 ± 0.16	NEE_{RP} (10)	-0.14 ± 0.26	< 0.0001	****	Mann-Whitney test
δ^{66}Zn	Asia	NS (39)	0.54 ± 0.16	OE (5)	0.03 ± 0.34	0.0277	*	Welch's *t*-test
δ^{66}Zn	Asia	NEE_{BP} (17)	-0.03 ± 0.09	NEE_T (5)	-0.01 ± 0.06	0.5349		Unpaired Student *t*-test
δ^{66}Zn	Asia	NEE_{BP} (17)	-0.03 ± 0.09	WI (5)	0.21 ± 0.09	0.0011	**	Unpaired Student *t*-test
δ^{66}Zn	Asia	NEE_{BP} (17)	-0.03 ± 0.09	NEE_{RP} (10)	-0.14 ± 0.26	0.9398		Mann-Whitney test
δ^{66}Zn	Asia	NEE_{BP} (17)	-0.03 ± 0.09	OE (5)	0.03 ± 0.34	0.7266		Welch's *t*-test
δ^{66}Zn	Asia	NEE_T (5)	-0.01 ± 0.06	WI (5)	0.21 ± 0.09	0.0023	**	Unpaired Student *t*-test
δ^{66}Zn	Asia	NEE_T (5)	-0.01 ± 0.06	NEE_{RP} (10)	-0.14 ± 0.26	0.8539		Mann-Whitney test
δ^{66}Zn	Asia	NEE_T (5)	-0.01 ± 0.06	OE (5)	0.03 ± 0.34	0.8305		Welch's *t*-test
δ^{66}Zn	Asia	WI (5)	0.21 ± 0.09	NEE_{RP} (10)	-0.14 ± 0.26	0.0039	**	Mann-Whitney test
δ^{66}Zn	Asia	WI (5)	0.21 ± 0.09	OE (5)	0.03 ± 0.34	0.3048		Welch's *t*-test
δ^{66}Zn	Asia	NEE_{RP} (10)	-0.14 ± 0.26	OE (5)	0.03 ± 0.34	0.2438		Mann-Whitney test
δ^{66}Zn	America	BB (4)	0.49 ± 0.17	CC (26)	0.32 ± 0.51	0.2201		Unpaired Student *t*-test
δ^{66}Zn	America	BB (4)	0.49 ± 0.17	VE (15)	0.28 ± 0.26	0.3415		Mann-Whitney test
δ^{66}Zn	America	BB (4)	0.49 ± 0.17	NS (4)	0.53 ± 0.20	0.7758		Unpaired Student *t*-test
δ^{66}Zn	America	BB (4)	0.49 ± 0.17	NEE_{BP} (3)	-0.02 ± 0.06	0.0056	**	Unpaired Student *t*-test
δ^{66}Zn	America	BB (4)	0.49 ± 0.17	NEE_T (8)	0.32 ± 0.30	0.5508		Mann-Whitney test
δ^{66}Zn	America	BB (4)	0.49 ± 0.17	WI (4)	0.20 ± 0.13	0.0357	*	Unpaired Student *t*-test
δ^{66}Zn	America	BB (4)	0.49 ± 0.17	NEE_{RP} (6)	0.64 ± 0.17	0.2023		Unpaired Student *t*-test
δ^{66}Zn	America	BB (4)	0.49 ± 0.17	OE (5)	0.05 ± 0.28	0.0247	*	Unpaired Student *t*-test
δ^{66}Zn	America	CC (26)	0.32 ± 0.51	VE (15)	0.28 ± 0.26	0.5071		Mann-Whitney test
δ^{66}Zn	America	CC (26)	0.32 ± 0.51	NS (4)	0.53 ± 0.20	0.1761		Unpaired Student *t*-test

$\delta^{66}Zn$	America	CC (26)	0.32 ± 0.51	NEE_{BP} (3)	-0.02 ± 0.06	0.0032	**	Welch's *t*-test
$\delta^{66}Zn$	America	CC (26)	0.32 ± 0.51	NEE_{T} (8)	0.32 ± 0.30	0.6696		Mann-Whitney test
$\delta^{66}Zn$	America	CC (26)	0.32 ± 0.51	WI (4)	0.20 ± 0.13	0.3122		Welch's *t*-test
$\delta^{66}Zn$	America	CC (26)	0.32 ± 0.51	NEE_{RP} (6)	0.64 ± 0.17	0.0134	*	Welch's *t*-test
$\delta^{66}Zn$	America	CC (26)	0.32 ± 0.51	OE (5)	0.05 ± 0.28	0.1244		Unpaired Student *t*-test
$\delta^{66}Zn$	America	VE (15)	0.28 ± 0.26	NS (4)	0.53 ± 0.20	0.1207		Mann-Whitney test
$\delta^{66}Zn$	America	VE (15)	0.28 ± 0.26	NEE_{BP} (3)	-0.02 ± 0.06	0.2354		Mann-Whitney test
$\delta^{66}Zn$	America	VE (15)	0.28 ± 0.26	NEE_{T} (8)	0.32 ± 0.30	0.5822		Mann-Whitney test
$\delta^{66}Zn$	America	VE (15)	0.28 ± 0.26	WI (4)	0.20 ± 0.13	0.2107		Mann-Whitney test
$\delta^{66}Zn$	America	VE (15)	0.28 ± 0.26	NEE_{RP} (6)	0.64 ± 0.17	0.0072	**	Mann-Whitney test
$\delta^{66}Zn$	America	VE (15)	0.28 ± 0.26	OE (5)	0.05 ± 0.28	0.0545		Mann-Whitney test
$\delta^{66}Zn$	America	NS (4)	0.53 ± 0.20	NEE_{BP} (3)	-0.02 ± 0.06	0.0092	**	Unpaired Student *t*-test
$\delta^{66}Zn$	America	NS (4)	0.53 ± 0.20	NEE_{T} (8)	0.32 ± 0.30	0.2679		Mann-Whitney test
$\delta^{66}Zn$	America	NS (4)	0.53 ± 0.20	WI (4)	0.20 ± 0.13	0.0402	*	Unpaired Student *t*-test
$\delta^{66}Zn$	America	NS (4)	0.53 ± 0.20	NEE_{RP} (6)	0.64 ± 0.17	0.3906		Unpaired Student *t*-test
$\delta^{66}Zn$	America	NS (4)	0.53 ± 0.20	OE (5)	0.05 ± 0.28	0.0216	*	Unpaired Student *t*-test
$\delta^{66}Zn$	America	NEE_{BP} (3)	-0.02 ± 0.06	NEE_{T} (8)	0.32 ± 0.30	0.0813		Mann-Whitney test
$\delta^{66}Zn$	America	NEE_{BP} (3)	-0.02 ± 0.06	WI (4)	0.20 ± 0.13	0.0334	*	Unpaired Student *t*-test
$\delta^{66}Zn$	America	NEE_{BP} (3)	-0.02 ± 0.06	NEE_{RP} (6)	0.64 ± 0.17	0.0001	****	Unpaired Student *t*-test
$\delta^{66}Zn$	America	NEE_{BP} (3)	-0.02 ± 0.06	OE (5)	0.05 ± 0.28	0.6030		Unpaired Student *t*-test
$\delta^{66}Zn$	America	NEE_{T} (8)	0.32 ± 0.30	WI (4)	0.20 ± 0.13	0.1054		Mann-Whitney test
$\delta^{66}Zn$	America	NEE_{T} (8)	0.32 ± 0.30	NEE_{RP} (6)	0.64 ± 0.17	0.0236	*	Mann-Whitney test
$\delta^{66}Zn$	America	NEE_{T} (8)	0.32 ± 0.30	OE (5)	0.05 ± 0.28	0.0333	*	Mann-Whitney test

$\delta^{66}Zn$	America	WI (4)	0.20 ± 0.13	NEE_{RP} (6)	0.64 ± 0.17	0.0015	**	Unpaired Student *t*-test
$\delta^{66}Zn$	America	WI (4)	0.20 ± 0.13	OE (5)	0.05 ± 0.28	0.3391		Unpaired Student *t*-test
$\delta^{66}Zn$	America	NEE_{RP} (6)	0.64 ± 0.17	OE (5)	0.05 ± 0.28	0.0055	**	Unpaired Student *t*-test
$\delta^{66}Zn$	Europe	BB (66)	0.48 ± 0.25	CC (15)	1.02 ± 0.46	0.0001	****	Mann-Whitney test
$\delta^{66}Zn$	Europe	BB (66)	0.48 ± 0.25	VE (12)	0.29 ± 0.25	0.0508		Mann-Whitney test
$\delta^{66}Zn$	Europe	BB (66)	0.48 ± 0.25	NS (4)	0.29 ± 0.43	0.3689		Mann-Whitney test
$\delta^{66}Zn$	Europe	BB (66)	0.48 ± 0.25	NEE_{BP} (3)	0.47 ± 0.03	0.7573		Mann-Whitney test
$\delta^{66}Zn$	Europe	BB (66)	0.48 ± 0.25	NEE_{T} (6)	0.45 ± 0.05	0.8305		Mann-Whitney test
$\delta^{66}Zn$	Europe	BB (66)	0.48 ± 0.25	WI (6)	0.19 ± 0.12	0.0016	**	Mann-Whitney test
$\delta^{66}Zn$	Europe	BB (66)	0.48 ± 0.25	NEE_{RP} (5)	0.59 ± 0.25	0.2907		Mann-Whitney test
$\delta^{66}Zn$	Europe	BB (66)	0.48 ± 0.25	OE (20)	0.20 ± 0.32	0.0037	**	Mann-Whitney test
$\delta^{66}Zn$	Europe	CC (15)	1.02 ± 0.46	VE (12)	0.29 ± 0.25	< 0.0001	****	Welch's *t*-test
$\delta^{66}Zn$	Europe	CC (15)	1.02 ± 0.46	NS (4)	0.29 ± 0.43	0.0306	*	Unpaired Student *t*-test
$\delta^{66}Zn$	Europe	CC (15)	1.02 ± 0.46	NEE_{BP} (3)	0.47 ± 0.03	0.0004	***	Welch's *t*-test
$\delta^{66}Zn$	Europe	CC (15)	1.02 ± 0.46	NEE_{T} (6)	0.45 ± 0.05	0.0003	***	Welch's *t*-test
$\delta^{66}Zn$	Europe	CC (15)	1.02 ± 0.46	WI (6)	0.19 ± 0.12	< 0.0001	****	Welch's *t*-test
$\delta^{66}Zn$	Europe	CC (15)	1.02 ± 0.46	NEE_{RP} (5)	0.59 ± 0.25	0.0189	*	Unpaired Student *t*-test
$\delta^{66}Zn$	Europe	CC (15)	1.02 ± 0.46	OE (20)	0.20 ± 0.32	0.0001	****	Mann-Whitney test
$\delta^{66}Zn$	Europe	VE (12)	0.29 ± 0.25	NS (4)	0.29 ± 0.43	0.9916		Unpaired Student *t*-test
$\delta^{66}Zn$	Europe	VE (12)	0.29 ± 0.25	NEE_{BP} (3)	0.47 ± 0.03	0.0323	*	Welch's *t*-test
$\delta^{66}Zn$	Europe	VE (12)	0.29 ± 0.25	NEE_{T} (6)	0.45 ± 0.05	0.0517		Welch's *t*-test
$\delta^{66}Zn$	Europe	VE (12)	0.29 ± 0.25	WI (6)	0.19 ± 0.12	0.2701		Unpaired Student *t*-test
$\delta^{66}Zn$	Europe	VE (12)	0.29 ± 0.25	NEE_{RP} (5)	0.59 ± 0.25	0.0555		Unpaired Student *t*-test
$\delta^{66}Zn$	Europe	VE (12)	0.29 ± 0.25	OE (20)	0.20 ± 0.32	0.4593		Mann-Whitney test

δ^{66}Zn	Europe	NS (4)	0.29 ± 0.43	NEE_{BP} (3)	0.47 ± 0.03	0.4650		Welch's *t*-test
δ^{66}Zn	Europe	NS (4)	0.29 ± 0.43	NEE_{T} (6)	0.45 ± 0.05	0.5045		Welch's *t*-test
δ^{66}Zn	Europe	NS (4)	0.29 ± 0.43	WI (6)	0.19 ± 0.12	0.6897		Welch's *t*-test
δ^{66}Zn	Europe	NS (4)	0.29 ± 0.43	NEE_{RP} (5)	0.59 ± 0.25	0.2754		Unpaired Student *t*-test
δ^{66}Zn	Europe	NS (4)	0.29 ± 0.43	OE (20)	0.20 ± 0.32	0.5602		Mann-Whitney test
δ^{66}Zn	Europe	NEE_{BP} (3)	0.47 ± 0.03	NEE_{T} (6)	0.45 ± 0.05	0.5425		Unpaired Student *t*-test
δ^{66}Zn	Europe	NEE_{BP} (3)	0.47 ± 0.03	WI (6)	0.19 ± 0.12	0.0017	**	Unpaired Student *t*-test
δ^{66}Zn	Europe	NEE_{BP} (3)	0.47 ± 0.03	NEE_{RP} (5)	0.59 ± 0.25	0.3402		Welch's *t*-test
δ^{66}Zn	Europe	NEE_{BP} (3)	0.47 ± 0.03	OE (20)	0.20 ± 0.32	0.0548		Mann-Whitney test
δ^{66}Zn	Europe	NEE_{T} (6)	0.45 ± 0.05	WI (6)	0.19 ± 0.12	0.0018	**	Unpaired Student *t*-test
δ^{66}Zn	Europe	NEE_{T} (6)	0.45 ± 0.05	NEE_{RP} (5)	0.59 ± 0.25	0.2868		Welch's *t*-test
δ^{66}Zn	Europe	NEE_{T} (6)	0.45 ± 0.05	OE (20)	0.20 ± 0.32	0.0381	*	Mann-Whitney test
δ^{66}Zn	Europe	WI (6)	0.19 ± 0.12	NEE_{RP} (5)	0.59 ± 0.25	0.0188	*	Unpaired Student *t*-test
δ^{66}Zn	Europe	WI (6)	0.19 ± 0.12	OE (20)	0.20 ± 0.32	0.3296		Mann-Whitney test
δ^{66}Zn	Europe	NEE_{RP} (5)	0.59 ± 0.25	OE (20)	0.20 ± 0.32	0.0485	*	Mann-Whitney test

1. Sources types (abbreviations): Biomass Burning (BB), Biomass Burning-C3 plants (BB_{C3}), Biomass Burning-C4 plants (BB_{C4}), Coal Combustion (CC), Vehicle Exhausts (VE), Vehicle Exhausts-Gasoline Vehicle ($VE_{gasoline}$), Vehicle Exhausts-Diesel Vehicle (VE_{diesel}), Natural Soil (NS), Ore-related Emissions (OE), Waste Materials (WM), Non-exhaust emissions (NEE), Non-exhaust emissions-Brake Pads (NEE_{BP}), Non-exhaust emissions-Road Paint (NEE_{RP}), Non-exhaust emissions-Tires (NEE_{T}), Waste Incinerator (WI), Liquid Fossil Fuels (LFF), Livestock Emissions (LE), Microbial Processes (MicP), Volatilized Fertilizer (VF), Industrial Oil Combustion (IOC).
2. All results are in ‰ except for 207Pb/206Pb. Significance code: '****' < 0.0001, '***' < 0.001, '**' < 0.01, '*' < 0.0

Supplementary Table 3. Basic information on PM and source isotopic fingerprints.

Element	Searching keyword 1#	Searching keyword 2#	Isotope	Data count	SRM[a]	Number of reference
C	"atmospher* particulate matter" or "$PM_{2.5}$" or "PM_{10}" or "aerosol"	"Carbon isotope*" or "13-Carbon" or "14-Carbon" or "Radiocarbon" or "stable carbon isotop*" or "$\delta^{13}C$" or "$\Delta^{14}C$" or "fraction modern carbon"	$\delta^{13}C$	3974	Vienna Pee Dee Belemnite (VPDB)	402[17-418]
			$f_{M\text{-}^{14}C}$	1537	the oxalic acid II 4990C (NIST)	
N		"Nitrogen isotop*" or "15-Nitrogen" or "$\delta^{15}N$"	$\delta^{15}N$	3096	atmospheric N_2 (air N_2)	189[347-416,419-545,813-820]
O		"Oxygen isotop*" or "17-Oxygen" or "18-Oxygen" or "$\delta^{17}O$" or "$\delta^{18}O$" or "$\Delta^{17}O$"	$\delta^{17}O$	16	Vienna Standard Mean Ocean Water (VSMOW)	91[412,442,507-595]
			$\Delta^{17}O^{b}$	142		
			$\delta^{18}O$	666		
S		"Sulfur isotop*" or "33-Sulfur" or "34-Sulfur" or "36-Sulfur" or "$\delta^{32}S$" or "$\delta^{34}S$" or "$\delta^{36}S$"	$\delta^{33}S$	31	Vienna Canyon Diablo Troilite (VCDT)	68[413-416,418,544,545,589-649]
			$\Delta^{33}S^{c}$	156		
			$\delta^{34}S$	1024		
			$\delta^{36}S$	3		
			$\Delta^{36}S^{d}$	12		
Cu		"Copper isotop*" or "65-Copper" or "$\delta^{65}Cu$"	$\delta^{65}Cu$	430	ERM-AE633	13[650-662]

Fe	"Iron isotop*" or "56-Iron" or "57-Iron" or "δ^{56}Fe" or "δ^{57}Fe"	δ^{56}Fe<br>δ^{57}Fe	495<br>6	IRMM-014	16[663-678]
Si	"Silicon isotop*" or "30-Silicon" or "29-Silicon" or "δ^{30}Si" or "δ^{29}Si"	δ^{30}Si<br>δ^{29}Si	311<br>115	NIST SRM-8546	4[650,679-681]
Zn	"Zinc isotop*" or "66-Zinc" or "δ^{66}Zn"	δ^{66}Zn<br>δ^{68}Zn	616<br>28	IRMM-3702[e]	21[654-662,682-693]
Pb	"Lead isotop*" or "^{206}Pb/^{204}Pb" or "^{207}Pb/^{204}Pb" or "^{208}Pb/^{204}Pb" or "^{207}Pb/^{206}Pb" or "^{208}Pb/^{206}Pb" or "^{208}Pb/^{207}Pb" or "^{206}Pb/^{207}Pb" or "^{204}Pb/^{207}Pb"	^{206}Pb/^{204}Pb<br>^{207}Pb/^{204}Pb<br>^{207}Pb/^{206}Pb<br>^{208}Pb/^{204}Pb<br>^{208}Pb/^{206}Pb<br>^{208}Pb/^{207}Pb	385<br>224<br>1446<br>209<br>462<br>199	-	79[417,649,653-658,682,683,694-762]
Sr	"Strontium isotop*" or "^{87}Sr/^{86}Sr"	δ^{87}Sr	1130	basaltic achondrite best initial (BABI)	32[695-702,759-782]
Nd	"Neodymium isotop*" or "^{144}Nd/^{143}Nd" or "^{143}Nd/^{144}Nd" or "εNd"	δ^{144}Nd	437	the Chondrite Uniform Reservoir (CHUR)	21[694-702,763-771,783-785]
Ni	"Nickel isotop*" or "60-Nickel" or "δ^{60}Ni"	δ^{60}Ni	76	NIST SRM-986	3[651,652,682]
Hg[f]		δ^{199}Hg	79	NIST-3133	27[362,786-811]

	“Mercury isotop*” or “199-Mercury” or “200-Mercury” or “201-Mercury” or “204-Mercury” or “δ^{199}Hg” or “δ^{200}Hg” or “δ^{201}Hg” or “δ^{204}Hg” or “Δ^{199}Hg” or “Δ^{200}Hg” or “Δ^{201}Hg” or “Δ^{204}Hg”	Δ^{199}Hg	277		
		δ^{200}Hg	79		
		Δ^{200}Hg	255		
		δ^{201}Hg	78		
		Δ^{201}Hg	257		
		δ^{202}Hg	279		
		δ^{204}Hg	69		
		Δ^{204}Hg	107		
Hf	“Hafnium isotop*” or “^{176}Hf/^{177}Hf” or “^{177}Hf/^{176}Hf” or “εHf”	δ^{177}Hf	54	the Chondrite Uniform Reservoir	4[763-765,820]

[a] SRM represents isotopic standard reference material.

[b] $\Delta^{17}O = \delta^{17}O - 0.52\ \delta^{18}O$

[c] $\Delta^{33}S = (\delta^{33}S + 1) - (\delta^{34}S + 1)^{0.52}$

[d] $\Delta^{36}S = (\delta^{36}S + 1) - (\delta^{34}S + 1)^{1.89}$

[e] $\delta^{66}Zn_{IRMM} = \delta^{66}Zn_{JMC\text{-}Lyon} + 0.3‰$, $\delta^{66}Zn_{JMC\text{-}Lyon} = \delta^{66}Zn_{Imperial} - 0.09‰$

[f] $\Delta^{xxx}Hg = \delta^{xxx}Hg - \beta \times \delta^{202}Hg$, β is 0.252, 0.502, 0.752, and 1.49 for ^{199}Hg, ^{200}Hg, ^{201}Hg, and ^{204}Hg

Supplementary Table 4. Summary of source emission sampling and isotopic analysis.

Isotope	Content	Source type	Sampling method	Analytical method	Quality Control Materials
$\delta^{15}N$	NH_3	Biomass Burning<br>Coal Combustion<br>Vehicle Exhausts<br>Waste Materials<br>Volatilized Fertilizer<br>Livestock Emissions	1. Passive sampling<br>(Corrected by adding 15.4‰)<br>2. Active sampling	Chemical method[821,822];<br>Biological method [823];<br>analyzed by Isotope Ratio Mass Spectrometer (including IRMS, PT-IRMS, and CF-IRMS)	IAEA-N, USGS25, USGS26[a]
$\delta^{15}N$	NO_x	Biomass Burning<br>Coal Combustion<br>Microbial Processes<br>Vehicle Exhausts	Active sampling	Biological method[823], analyzed by Isotope Ratio Mass Spectrometer (including IRMS, EA-IRMS, and CF-IRMS)	IAEA-N3, USGS32, USGS34, USGS35[b]
$\delta^{34}S$	SO_2	Biomass Burning<br>Coal Combustion<br>Industrial Oil Combustion<br>Natural Soil<br>Vehicle Exhausts	1. Active sampling<br>2. Passive sampling (only 1 publication, the data was not used for source apportionment)	Isotope Ratio Mass Spectrometer or VG Iso-gas Mass Spectrometer	IAEA S-1, IAEA S-2, IAEA-SO5, IAEA-SO6, LTB-2, LTB-5, CSIRD, NBS127[c]
$\delta^{13}C$	EC/OC	Biomass Burning<br>Coal Combustion<br>Vehicle Exhausts (EC)<br>Liquid Fossil Fuels (OC)	Active sampling: Directly collected in PM form	Isotope Ratio Mass Spectrometer (IRMS)	IAEA-CH6, IAEA-CH7, USGS24, NBS-19, RM 8573, RM 8542[d]

$\delta^{87}Sr$<br>$\delta^{144}Nd$<br>$^{207}Pb/^{206}Pb$	Sr<br>Nd<br>Pb	Coal Combustion<br>Vehicle Exhausts<br>Non-exhaust emissions<br>Natural Soil<br>Ore-related Emissions (Pb & Sr)<br>Waste Incinerator (Pb&Nd)	Active sampling: Directly collected in PM form	1. MC-ICP-MS<br>2. Thermal ionization mass spectrometer (TIMS)<br>3. ICP-MS (only Pb)	Sr: NBS987; Pb: NIST SRM981; Nd: JMC, JNdi-1, La Jolla Standard[e]
$\delta^{30}Si$<br>$\delta^{56}Fe$<br>$\delta^{65}Cu$<br>$\delta^{66}Zn$	Si<br>Fe<br>Cu<br>Zn	Biomass Burning<br>Coal Combustion (Si, Fe, Zn)<br>Natural Soil<br>Vehicle Exhausts<br>Ore-related Emissions<br>Non-exhaust emissions (Cu, Fe)<br>Waste Incinerator (Zn)	Active sampling: Directly collected in PM form	MC-ICP-MS	Si: NIST-SRM-8546, IRMM-017; LVLK-132; Cu: ERM-AE633, ERM-AE647, SRM NIST 976, CAG-Cu; Zn: IRMM-3702, AA-ETH, SRM-683, JMC 3-0749L[f]

[a] The analytical precision (1σ) 0.005‰ − 0.9‰

[b] The analytical precision (1σ) 0.2‰ − 1.5‰

[c] The analytical precision (1σ) 0.15‰ − 0.4‰

[d] The analytical precision (1σ) 0.1‰ − 0.3‰

[e] The analytical precision (1σ) of $\delta^{87}Sr$: 0.08‰ − 0.3‰, $^{144}Nd/^{143}Nd$: 0.004% − 0.005%, $^{207}Pb/^{206}Pb$: 0.0004 − 0.0019 or 0.2%−0.5%

[e] The analytical precision (1σ) of $\delta^{30}Si$ about 0.18‰, $\delta^{56}Fe$: 0.03‰ − 0.09‰, $\delta^{65}Cu$: 0.04‰ − 0.09‰, $\delta^{66}Zn$: 0.03‰ − 0.08‰.

Supplementary Table 5. Detailed information on source emission sampling and isotopic analysis.

Isotope Type	Content	Source type	Isotopic composition	Lat. (S-,N+)	Lon. (W-,E+)	Sampling Date	Sampling method & Correction	Analytical method	Measurement correction	Analytical precision (1σ, ‰)	Reference
$\delta^{13}C$	EC	Biomass Burning-C3	-28.00‰	31.32	138.25	April, 2011	Active sampling	IRMS	Standard Calibration Method	0.14	10.1016/j.atmosenv.2011.05.015
$\delta^{13}C$	EC	Biomass Burning-C3	-29.40‰	31.32	138.25	January, 2011	Active sampling	IRMS	Standard Calibration Method	0.14	10.1016/j.atmosenv.2011.05.015
$\delta^{13}C$	EC	Biomass Burning-C3	-29.20‰	31.32	138.25	July, 2011	Active sampling	IRMS	Standard Calibration Method	0.14	10.1016/j.atmosenv.2011.05.015
$\delta^{13}C$	EC	Biomass Burning-C3	-32.10‰	31.32	138.25	October, 2011	Active sampling	IRMS	Standard Calibration Method	0.14	10.1016/j.atmosenv.2011.05.015
$\delta^{13}C$	EC	Biomass Burning-C3	-32.30‰	31.32	138.25	April, 2011	Active sampling	IRMS	Standard Calibration Method	0.14	10.1016/j.atmosenv.2011.05.015
$\delta^{13}C$	EC	Biomass Burning-C3	-30.70‰	31.32	138.25	January, 2011	Active sampling	IRMS	Standard Calibration Method	0.14	10.1016/j.atmosenv.2011.05.015
$\delta^{13}C$	EC	Biomass Burning-C3	-31.70‰	31.32	138.25	July, 2011	Active sampling	IRMS	Standard Calibration Method	0.14	10.1016/j.atmosenv.2011.05.015
$\delta^{13}C$	EC	Biomass Burning-C3	-34.70‰	31.32	138.25	October, 2011	Active sampling	IRMS	Standard Calibration Method	0.14	10.1016/j.atmosenv.2011.05.015
$\delta^{13}C$	EC	Biomass Burning-C3	-28.80‰	31.32	138.25	July, 2011	Active sampling	IRMS	Standard Calibration Method	0.14	10.1016/j.atmosenv.2011.05.015
$\delta^{13}C$	EC	Biomass Burning-C3	-29.40‰	31.32	138.25	October, 2011	Active sampling	IRMS	Standard Calibration Method	0.14	10.1016/j.atmosenv.2011.05.015
$\delta^{13}C$	EC	Biomass Burning-C3	-28.20‰	23.13	113.26	July, 2020	Active sampling	IRMS	Standard Calibration Method	0.3	10.1029/2020JD033920
$\delta^{13}C$	EC	Biomass Burning-C3	-28.03‰	37.51	121.05	April, 2012	Active sampling	IRMS	Standard Calibration Method	0.3	10.13227/j.hjkx.2012.03.021 in Chinese
$\delta^{13}C$	EC	Biomass Burning-C3	-28.09‰	37.51	121.05	January, 2012	Active sampling	IRMS	Standard Calibration Method	0.3	10.13227/j.hjkx.2012.03.021 in Chinese
$\delta^{13}C$	EC	Biomass Burning-C3	-26.51‰	37.51	121.05	July, 2012	Active sampling	IRMS	Standard Calibration Method	0.3	10.13227/j.hjkx.2012.03.021 in Chinese

$\delta^{13}C$	EC	Biomass Burning-C3	-26.44‰	37.51	121.05	October, 2012	Active sampling	IRMS	Standard Calibration Method	0.3	10.13227/j.hjkx.2012.03.021 in Chinese
$\delta^{13}C$	EC	Biomass Burning-C3	-28.20‰	37.51	121.05	April, 2012	Active sampling	IRMS	Standard Calibration Method	0.3	10.13227/j.hjkx.2012.03.021 in Chinese
$\delta^{13}C$	EC	Biomass Burning-C3	-26.54‰	37.51	121.05	January, 2012	Active sampling	IRMS	Standard Calibration Method	0.3	10.13227/j.hjkx.2012.03.021 in Chinese
$\delta^{13}C$	EC	Biomass Burning-C3	-24.95‰	37.51	121.05	July, 2012	Active sampling	IRMS	Standard Calibration Method	0.3	10.13227/j.hjkx.2012.03.021 in Chinese
$\delta^{13}C$	EC	Biomass Burning-C3	-28.33‰	37.51	121.05	October, 2012	Active sampling	IRMS	Standard Calibration Method	0.3	10.13227/j.hjkx.2012.03.021 in Chinese
$\delta^{13}C$	EC	Biomass Burning-C3	-27.60‰	37.51	121.05	April, 2012	Active sampling	IRMS	Standard Calibration Method	0.3	10.13227/j.hjkx.2012.03.021 in Chinese
$\delta^{13}C$	EC	Biomass Burning-C3	-27.35‰	37.51	121.05	January, 2012	Active sampling	IRMS	Standard Calibration Method	0.3	10.13227/j.hjkx.2012.03.021 in Chinese
$\delta^{13}C$	EC	Biomass Burning-C3	-26.91‰	37.51	121.05	July, 2012	Active sampling	IRMS	Standard Calibration Method	0.3	10.13227/j.hjkx.2012.03.021 in Chinese
$\delta^{13}C$	EC	Biomass Burning-C3	-28.01‰	37.51	121.05	October, 2012	Active sampling	IRMS	Standard Calibration Method	0.3	10.13227/j.hjkx.2012.03.021 in Chinese
$\delta^{13}C$	EC	Biomass Burning-C3	-26.97‰	37.51	121.05	July, 2012	Active sampling	IRMS	Standard Calibration Method	0.3	10.13227/j.hjkx.2012.03.021 in Chinese
$\delta^{13}C$	EC	Biomass Burning-C3	-24.49‰	37.51	121.05	October, 2012	Active sampling	IRMS	Standard Calibration Method	0.3	10.13227/j.hjkx.2012.03.021 in Chinese
$\delta^{13}C$	EC	Biomass Burning-C3	-28.83‰	32.06	118.79	July, 2016	Active sampling	IRMS	Standard Calibration Method	0.2	10.13227/j.hjkx.2016.01.004 in Chinese
$\delta^{13}C$	EC	Biomass Burning-C3	-28.86‰	32.06	118.79	July, 2016	Active sampling	IRMS	Standard Calibration Method	0.2	10.13227/j.hjkx.2016.01.004 in Chinese
$\delta^{13}C$	EC	Biomass Burning-C3	-30.42‰	32.06	118.79	July, 2016	Active sampling	IRMS	Standard Calibration Method	0.2	10.13227/j.hjkx.2016.01.004 in Chinese
$\delta^{13}C$	EC	Biomass Burning-C3	-29.81‰	32.06	118.79	July, 2016	Active sampling	IRMS	Standard Calibration Method	0.2	10.13227/j.hjkx.2016.01.004 in Chinese

$\delta^{13}C$	EC	Biomass Burning-C3	-29.83‰	32.06	118.79	July, 2016	Active sampling	IRMS	Standard Calibration Method	0.2	10.13227/j.hjkx.2016.01.004 in Chinese
$\delta^{13}C$	EC	Biomass Burning-C3	-13.66‰	43.06	141.35	July, 2014	Active sampling	IRMS	Standard Calibration Method	0.3	http://dx.doi.org/10.1016/j.atmosenv.2014.05.045
$\delta^{13}C$	EC	Biomass Burning-C3	-25.65‰	51.17	10.45	January, 2021	Active sampling	IRMS	Standard Calibration Method	0.21	https://doi.org/10.1016/j.atmosenv.2021.118842
$\delta^{13}C$	EC	Biomass Burning-C3	-25.31‰	51.17	23.88	April, 2021	Active sampling	IRMS	Standard Calibration Method	0.21	https://doi.org/10.1016/j.atmosenv.2021.118842
$\delta^{13}C$	EC	Biomass Burning-C3	-25.57‰	51.17	10.45	July, 2021	Active sampling	IRMS	Standard Calibration Method	0.21	https://doi.org/10.1016/j.atmosenv.2021.118842
$\delta^{13}C$	EC	Biomass Burning-C3	-26.26‰	51.17	23.88	October, 2021	Active sampling	IRMS	Standard Calibration Method	0.21	https://doi.org/10.1016/j.atmosenv.2021.118842
$\delta^{13}C$	EC	Biomass Burning-C3	-25.27‰	51.17	23.88	January, 2021	Active sampling	IRMS	Standard Calibration Method	0.21	https://doi.org/10.1016/j.atmosenv.2021.118842
$\delta^{13}C$	EC	Biomass Burning-C3	-26.60‰	51.17	23.88	April, 2021	Active sampling	IRMS	Standard Calibration Method	0.21	https://doi.org/10.1016/j.atmosenv.2021.118842
$\delta^{13}C$	EC	Biomass Burning-C3	-26.93‰	51.17	10.45	July, 2021	Active sampling	IRMS	Standard Calibration Method	0.21	https://doi.org/10.1016/j.atmosenv.2021.118842
$\delta^{13}C$	EC	Biomass Burning-C3	-25.93‰	51.17	23.88	October, 2021	Active sampling	IRMS	Standard Calibration Method	0.21	https://doi.org/10.1016/j.atmosenv.2021.118842
$\delta^{13}C$	EC	Biomass Burning-C3	-26.48‰	51.17	23.88	January, 2021	Active sampling	IRMS	Standard Calibration Method	0.21	https://doi.org/10.1016/j.atmosenv.2021.118842
$\delta^{13}C$	EC	Biomass Burning-C3	-26.64‰	51.17	23.88	April, 2021	Active sampling	IRMS	Standard Calibration Method	0.21	https://doi.org/10.1016/j.atmosenv.2021.118842
$\delta^{13}C$	EC	Biomass Burning-C3	-27.22‰	51.17	23.88	July, 2021	Active sampling	IRMS	Standard Calibration Method	0.21	https://doi.org/10.1016/j.atmosenv.2021.118842
$\delta^{13}C$	EC	Biomass Burning-C3	-27.72‰	51.17	23.88	October, 2021	Active sampling	IRMS	Standard Calibration Method	0.21	https://doi.org/10.1016/j.atmosenv.2021.118842
$\delta^{13}C$	EC	Biomass Burning-C3	-24.87‰	61.53	25.25	January, 2021	Active sampling	IRMS	Standard Calibration Method	0.21	https://doi.org/10.1016/j.atmosenv.2021.118842

δ^{13}C	EC	Biomass Burning-C3	-23.65‰	51.17	10.45	April, 2021	Active sampling	IRMS	Standard Calibration Method	0.21	https://doi.org/10.1016/j.atmosenv.2021.118842
δ^{13}C	EC	Biomass Burning-C3	-25.56‰	51.17	23.88	July, 2021	Active sampling	IRMS	Standard Calibration Method	0.21	https://doi.org/10.1016/j.atmosenv.2021.118842
δ^{13}C	EC	Biomass Burning-C3	-25.90‰	51.17	10.45	October, 2021	Active sampling	IRMS	Standard Calibration Method	0.21	https://doi.org/10.1016/j.atmosenv.2021.118842
δ^{13}C	EC	Biomass Burning-C3	-28.33‰	51.17	23.88	January, 2021	Active sampling	IRMS	Standard Calibration Method	0.21	https://doi.org/10.1016/j.atmosenv.2021.118842
δ^{13}C	EC	Biomass Burning-C3	-25.96‰	51.17	10.45	April, 2021	Active sampling	IRMS	Standard Calibration Method	0.21	https://doi.org/10.1016/j.atmosenv.2021.118842
δ^{13}C	EC	Biomass Burning-C3	-26.36‰	51.17	23.88	July, 2021	Active sampling	IRMS	Standard Calibration Method	0.21	https://doi.org/10.1016/j.atmosenv.2021.118842
δ^{13}C	EC	Biomass Burning-C3	-24.14‰	51.17	23.88	October, 2021	Active sampling	IRMS	Standard Calibration Method	0.21	https://doi.org/10.1016/j.atmosenv.2021.118842
δ^{13}C	EC	Biomass Burning-C3	-27.84‰	51.17	23.88	January, 2021	Active sampling	IRMS	Standard Calibration Method	0.21	https://doi.org/10.1016/j.atmosenv.2021.118842
δ^{13}C	EC	Biomass Burning-C3	-28.96‰	51.17	10.45	April, 2021	Active sampling	IRMS	Standard Calibration Method	0.21	https://doi.org/10.1016/j.atmosenv.2021.118842
δ^{13}C	EC	Biomass Burning-C3	-26.57‰	51.17	23.88	July, 2021	Active sampling	IRMS	Standard Calibration Method	0.21	https://doi.org/10.1016/j.atmosenv.2021.118842
δ^{13}C	EC	Biomass Burning-C3	-26.49‰	51.17	23.88	October, 2021	Active sampling	IRMS	Standard Calibration Method	0.21	https://doi.org/10.1016/j.atmosenv.2021.118842
δ^{13}C	EC	Biomass Burning-C3	-27.23‰	51.17	23.88	January, 2021	Active sampling	IRMS	Standard Calibration Method	0.21	https://doi.org/10.1016/j.atmosenv.2021.118842
δ^{13}C	EC	Biomass Burning-C3	-27.28‰	51.17	23.88	April, 2021	Active sampling	IRMS	Standard Calibration Method	0.21	https://doi.org/10.1016/j.atmosenv.2021.118842
δ^{13}C	EC	Biomass Burning-C3	-29.03‰	61.53	105.32	July, 2021	Active sampling	IRMS	Standard Calibration Method	0.21	https://doi.org/10.1016/j.atmosenv.2021.118842
δ^{13}C	EC	Biomass Burning-C3	-24.73‰	51.17	23.88	October, 2021	Active sampling	IRMS	Standard Calibration Method	0.21	https://doi.org/10.1016/j.atmosenv.2021.118842

$\delta^{13}C$	EC	Biomass Burning-C3	-24.35‰	61.53	105.32	January, 2021	Active sampling	IRMS	Standard Calibration Method	0.21	https://doi.org/10.1016/j.atmosenv.2021.118842
$\delta^{13}C$	EC	Biomass Burning-C3	-27.32‰	51.17	23.88	April, 2021	Active sampling	IRMS	Standard Calibration Method	0.21	https://doi.org/10.1016/j.atmosenv.2021.118842
$\delta^{13}C$	EC	Biomass Burning-C3	-24.44‰	51.17	10.45	July, 2021	Active sampling	IRMS	Standard Calibration Method	0.21	https://doi.org/10.1016/j.atmosenv.2021.118842
$\delta^{13}C$	EC	Biomass Burning-C3	-26.76‰	51.17	23.88	October, 2021	Active sampling	IRMS	Standard Calibration Method	0.21	https://doi.org/10.1016/j.atmosenv.2021.118842
$\delta^{13}C$	EC	Biomass Burning-C3	-24.63‰	51.17	10.45	January, 2021	Active sampling	IRMS	Standard Calibration Method	0.21	https://doi.org/10.1016/j.atmosenv.2021.118842
$\delta^{13}C$	EC	Biomass Burning-C3	-24.69‰	51.17	23.88	April, 2021	Active sampling	IRMS	Standard Calibration Method	0.21	https://doi.org/10.1016/j.atmosenv.2021.118842
$\delta^{13}C$	EC	Biomass Burning-C3	-22.76‰	51.17	23.88	July, 2021	Active sampling	IRMS	Standard Calibration Method	0.21	https://doi.org/10.1016/j.atmosenv.2021.118842
$\delta^{13}C$	EC	Biomass Burning-C3	-26.19‰	51.17	23.88	October, 2021	Active sampling	IRMS	Standard Calibration Method	0.21	https://doi.org/10.1016/j.atmosenv.2021.118842
$\delta^{13}C$	EC	Biomass Burning-C3	-26.93‰	51.17	23.88	January, 2021	Active sampling	IRMS	Standard Calibration Method	0.21	https://doi.org/10.1016/j.atmosenv.2021.118842
$\delta^{13}C$	EC	Biomass Burning-C3	-25.22‰	51.17	23.88	April, 2021	Active sampling	IRMS	Standard Calibration Method	0.21	https://doi.org/10.1016/j.atmosenv.2021.118842
$\delta^{13}C$	EC	Biomass Burning-C3	-25.03‰	55.17	10.45	July, 2021	Active sampling	IRMS	Standard Calibration Method	0.21	https://doi.org/10.1016/j.atmosenv.2021.118842
$\delta^{13}C$	EC	Biomass Burning-C3	-25.41‰	51.17	23.88	October, 2021	Active sampling	IRMS	Standard Calibration Method	0.21	https://doi.org/10.1016/j.atmosenv.2021.118842
$\delta^{13}C$	EC	Biomass Burning-C3	-25.39‰	51.17	23.88	January, 2021	Active sampling	IRMS	Standard Calibration Method	0.21	https://doi.org/10.1016/j.atmosenv.2021.118842
$\delta^{13}C$	EC	Biomass Burning-C3	-27.55‰	51.17	23.88	April, 2021	Active sampling	IRMS	Standard Calibration Method	0.21	https://doi.org/10.1016/j.atmosenv.2021.118842
$\delta^{13}C$	EC	Biomass Burning-C3	-23.16‰	61.53	105.32	July, 2021	Active sampling	IRMS	Standard Calibration Method	0.21	https://doi.org/10.1016/j.atmosenv.2021.118842

$\delta^{13}C$	EC	Biomass Burning-C3	-22.90‰	61.53	105.32	October, 2021	Active sampling	IRMS	Standard Calibration Method	0.21	https://doi.org/10.1016/j.atmosenv.2021.118842
$\delta^{13}C$	EC	Biomass Burning-C3	-25.44‰	51.17	23.88	October, 2021	Active sampling	IRMS	Standard Calibration Method	0.21	https://doi.org/10.1016/j.atmosenv.2021.118842
$\delta^{13}C$	EC	Biomass Burning-C3	-26.70‰	37.64	127.03	July, 2020	Active sampling	IRMS	Standard Calibration Method	0.3	https://doi.org/10.1016/j.envpol.2020.115163
$\delta^{13}C$	EC	Biomass Burning-C3	-28.13‰	34.23	108.88	April, 2021	Active sampling	IRMS	Standard Calibration Method	0.19	https://doi.org/10.1016/j.scitotenv.2021.151284
$\delta^{13}C$	EC	Biomass Burning-C3	-27.75‰	34.23	108.88	January, 2021	Active sampling	IRMS	Standard Calibration Method	0.19	https://doi.org/10.1016/j.scitotenv.2021.151284
$\delta^{13}C$	EC	Biomass Burning-C3	-27.73‰	31.87	117.28	July, 2021	Active sampling	IRMS	Standard Calibration Method	0.19	https://doi.org/10.1016/j.scitotenv.2021.151284
$\delta^{13}C$	EC	Biomass Burning-C3	-25.80‰	32.06	118.79	July, 2010	Active sampling	IRMS	Standard Calibration Method	0.3	https://doi.org/10.1016/j.atmosenv.2014.04.042
$\delta^{13}C$	EC	Biomass Burning-C3	-27.50‰	32.06	118.79	July, 2010	Active sampling	IRMS	Standard Calibration Method	0.3	https://doi.org/10.1016/j.atmosenv.2014.04.042
$\delta^{13}C$	EC	Biomass Burning-C3	-24.30‰	31.86	117.27	July, 2010	Active sampling	IRMS	Standard Calibration Method	0.3	https://doi.org/10.1016/j.atmosenv.2014.04.042
$\delta^{13}C$	EC	Biomass Burning-C3	-26.30‰	31.86	117.27	July, 2010	Active sampling	IRMS	Standard Calibration Method	0.3	https://doi.org/10.1016/j.atmosenv.2014.04.042
$\delta^{13}C$	EC	Biomass Burning-C3	-23.90‰	30.25	120.21	July, 2010	Active sampling	IRMS	Standard Calibration Method	0.3	https://doi.org/10.1016/j.atmosenv.2014.04.042
$\delta^{13}C$	EC	Biomass Burning-C3	-26.30‰	30.25	120.21	July, 2010	Active sampling	IRMS	Standard Calibration Method	0.3	https://doi.org/10.1016/j.atmosenv.2014.04.042
$\delta^{13}C$	EC	Biomass Burning-C3	-30.20‰	32.06	118.79	July, 2010	Active sampling	IRMS	Standard Calibration Method	0.3	https://doi.org/10.1016/j.atmosenv.2014.04.042
$\delta^{13}C$	EC	Biomass Burning-C3	-29.30‰	32.06	118.79	July, 2010	Active sampling	IRMS	Standard Calibration Method	0.3	https://doi.org/10.1016/j.atmosenv.2014.04.042
$\delta^{13}C$	EC	Biomass Burning-C3	-26.20‰	32.06	118.79	July, 2010	Active sampling	IRMS	Standard Calibration Method	0.3	https://doi.org/10.1016/j.atmosenv.2014.04.042

$\delta^{13}C$	EC	Biomass Burning-C3	-28.10‰	32.06	118.79	July, 2010	Active sampling	IRMS	Standard Calibration Method	0.3	https://doi.org/10.1016/j.atmosenv.2014.04.042
$\delta^{13}C$	EC	Biomass Burning-C3	-27.80‰	34.76	113.65	July, 2010	Active sampling	IRMS	Standard Calibration Method	0.3	https://doi.org/10.1016/j.atmosenv.2014.04.042
$\delta^{13}C$	EC	Biomass Burning-C3	-28.60‰	34.76	113.65	July, 2010	Active sampling	IRMS	Standard Calibration Method	0.3	https://doi.org/10.1016/j.atmosenv.2014.04.042
$\delta^{13}C$	EC	Biomass Burning-C3	-27.50‰	32.06	118.79	July, 2010	Active sampling	IRMS	Standard Calibration Method	0.3	https://doi.org/10.1016/j.atmosenv.2014.04.042
$\delta^{13}C$	EC	Biomass Burning-C3	-28.00‰	32.06	118.79	July, 2010	Active sampling	IRMS	Standard Calibration Method	0.3	https://doi.org/10.1016/j.atmosenv.2014.04.042
$\delta^{13}C$	EC	Biomass Burning-C3	-28.20‰	31.86	117.27	July, 2010	Active sampling	IRMS	Standard Calibration Method	0.3	https://doi.org/10.1016/j.atmosenv.2014.04.042
$\delta^{13}C$	EC	Biomass Burning-C3	-27.70‰	31.86	117.27	July, 2010	Active sampling	IRMS	Standard Calibration Method	0.3	https://doi.org/10.1016/j.atmosenv.2014.04.042
$\delta^{13}C$	EC	Biomass Burning-C3	-29.10‰	30.25	120.21	July, 2010	Active sampling	IRMS	Standard Calibration Method	0.3	https://doi.org/10.1016/j.atmosenv.2014.04.042
$\delta^{13}C$	EC	Biomass Burning-C3	-29.50‰	30.25	120.21	July, 2010	Active sampling	IRMS	Standard Calibration Method	0.3	https://doi.org/10.1016/j.atmosenv.2014.04.042
$\delta^{13}C$	EC	Biomass Burning-C3	-29.90‰	32.06	118.79	July, 2010	Active sampling	IRMS	Standard Calibration Method	0.3	https://doi.org/10.1016/j.atmosenv.2014.04.042
$\delta^{13}C$	EC	Biomass Burning-C3	-28.80‰	32.06	118.79	July, 2010	Active sampling	IRMS	Standard Calibration Method	0.3	https://doi.org/10.1016/j.atmosenv.2014.04.042
$\delta^{13}C$	EC	Biomass Burning-C3	-28.30‰	32.06	118.79	July, 2010	Active sampling	IRMS	Standard Calibration Method	0.3	https://doi.org/10.1016/j.atmosenv.2014.04.042
$\delta^{13}C$	EC	Biomass Burning-C3	-26.90‰	34.76	113.65	July, 2010	Active sampling	IRMS	Standard Calibration Method	0.3	https://doi.org/10.1016/j.atmosenv.2014.04.042
$\delta^{13}C$	EC	Biomass Burning-C3	-28.80‰	34.76	113.65	July, 2010	Active sampling	IRMS	Standard Calibration Method	0.3	https://doi.org/10.1016/j.atmosenv.2014.04.042
$\delta^{13}C$	EC	Biomass Burning-C3	-27.60‰	32.06	118.79	July, 2010	Active sampling	IRMS	Standard Calibration Method	0.3	https://doi.org/10.1016/j.atmosenv.2014.04.042

$\delta^{13}C$	EC	Biomass Burning-C3	-28.60‰	32.06	118.79	July, 2010	Active sampling	IRMS	Standard Calibration Method	0.3	https://doi.org/10.1016/j.atmosenv.2014.04.042
$\delta^{13}C$	EC	Biomass Burning-C3	-29.40‰	31.86	117.27	July, 2010	Active sampling	IRMS	Standard Calibration Method	0.3	https://doi.org/10.1016/j.atmosenv.2014.04.042
$\delta^{13}C$	EC	Biomass Burning-C3	-27.90‰	31.86	117.27	July, 2010	Active sampling	IRMS	Standard Calibration Method	0.3	https://doi.org/10.1016/j.atmosenv.2014.04.042
$\delta^{13}C$	EC	Biomass Burning-C3	-29.30‰	30.25	120.21	July, 2010	Active sampling	IRMS	Standard Calibration Method	0.3	https://doi.org/10.1016/j.atmosenv.2014.04.042
$\delta^{13}C$	EC	Biomass Burning-C3	-29.10‰	30.25	120.21	July, 2010	Active sampling	IRMS	Standard Calibration Method	0.3	https://doi.org/10.1016/j.atmosenv.2014.04.042
$\delta^{13}C$	EC	Biomass Burning-C3	-29.40‰	32.06	118.79	July, 2010	Active sampling	IRMS	Standard Calibration Method	0.3	https://doi.org/10.1016/j.atmosenv.2014.04.042
$\delta^{13}C$	EC	Biomass Burning-C3	-28.50‰	32.06	118.79	July, 2011	Active sampling	IRMS	Standard Calibration Method	0.3	https://doi.org/10.1016/j.atmosenv.2014.04.042
$\delta^{13}C$	EC	Biomass Burning-C3	-25.40‰	32.06	118.79	July, 2011	Active sampling	IRMS	Standard Calibration Method	0.3	https://doi.org/10.1016/j.atmosenv.2014.04.042
$\delta^{13}C$	EC	Biomass Burning-C3	-28.10‰	32.06	118.79	July, 2011	Active sampling	IRMS	Standard Calibration Method	0.3	https://doi.org/10.1016/j.atmosenv.2014.04.042
$\delta^{13}C$	EC	Biomass Burning-C3	-28.20‰	34.76	113.65	July, 2011	Active sampling	IRMS	Standard Calibration Method	0.3	https://doi.org/10.1016/j.atmosenv.2014.04.042
$\delta^{13}C$	EC	Biomass Burning-C3	-28.40‰	34.76	113.65	July, 2011	Active sampling	IRMS	Standard Calibration Method	0.3	https://doi.org/10.1016/j.atmosenv.2014.04.042
$\delta^{13}C$	EC	Biomass Burning-C3	-27.40‰	32.06	118.79	July, 2011	Active sampling	IRMS	Standard Calibration Method	0.3	https://doi.org/10.1016/j.atmosenv.2014.04.042
$\delta^{13}C$	EC	Biomass Burning-C3	-26.60‰	32.06	118.79	July, 2011	Active sampling	IRMS	Standard Calibration Method	0.3	https://doi.org/10.1016/j.atmosenv.2014.04.042
$\delta^{13}C$	EC	Biomass Burning-C3	-26.80‰	31.86	117.27	July, 2011	Active sampling	IRMS	Standard Calibration Method	0.3	https://doi.org/10.1016/j.atmosenv.2014.04.042
$\delta^{13}C$	EC	Biomass Burning-C3	-27.00‰	31.86	117.27	July, 2011	Active sampling	IRMS	Standard Calibration Method	0.3	https://doi.org/10.1016/j.atmosenv.2014.04.042

$\delta^{13}C$	EC	Biomass Burning-C3	-29.60‰	30.25	120.21	July, 2011	Active sampling	IRMS	Standard Calibration Method	0.3	https://doi.org/10.1016/j.atmosenv.2014.04.042
$\delta^{13}C$	EC	Biomass Burning-C3	-27.30‰	30.25	120.21	July, 2011	Active sampling	IRMS	Standard Calibration Method	0.3	https://doi.org/10.1016/j.atmosenv.2014.04.042
$\delta^{13}C$	EC	Biomass Burning-C3	-29.70‰	32.06	118.79	July, 2011	Active sampling	IRMS	Standard Calibration Method	0.3	https://doi.org/10.1016/j.atmosenv.2014.04.042
$\delta^{13}C$	EC	Biomass Burning-C3	-27.30‰	32.06	118.79	July, 2011	Active sampling	IRMS	Standard Calibration Method	0.3	https://doi.org/10.1016/j.atmosenv.2014.04.042
$\delta^{13}C$	EC	Biomass Burning-C3	-26.00‰	32.06	118.79	July, 2011	Active sampling	IRMS	Standard Calibration Method	0.3	https://doi.org/10.1016/j.atmosenv.2014.04.042
$\delta^{13}C$	EC	Biomass Burning-C3	-27.60‰	32.06	118.79	July, 2011	Active sampling	IRMS	Standard Calibration Method	0.3	https://doi.org/10.1016/j.atmosenv.2014.04.042
$\delta^{13}C$	EC	Biomass Burning-C3	-26.50‰	34.76	113.65	July, 2011	Active sampling	IRMS	Standard Calibration Method	0.3	https://doi.org/10.1016/j.atmosenv.2014.04.042
$\delta^{13}C$	EC	Biomass Burning-C3	-27.50‰	34.76	113.65	July, 2011	Active sampling	IRMS	Standard Calibration Method	0.3	https://doi.org/10.1016/j.atmosenv.2014.04.042
$\delta^{13}C$	EC	Biomass Burning-C3	-27.20‰	32.06	118.79	July, 2011	Active sampling	IRMS	Standard Calibration Method	0.3	https://doi.org/10.1016/j.atmosenv.2014.04.042
$\delta^{13}C$	EC	Biomass Burning-C3	-27.60‰	32.06	118.79	July, 2011	Active sampling	IRMS	Standard Calibration Method	0.3	https://doi.org/10.1016/j.atmosenv.2014.04.042
$\delta^{13}C$	EC	Biomass Burning-C3	-27.20‰	31.86	117.27	July, 2011	Active sampling	IRMS	Standard Calibration Method	0.3	https://doi.org/10.1016/j.atmosenv.2014.04.042
$\delta^{13}C$	EC	Biomass Burning-C3	-25.90‰	31.86	117.27	July, 2011	Active sampling	IRMS	Standard Calibration Method	0.3	https://doi.org/10.1016/j.atmosenv.2014.04.042
$\delta^{13}C$	EC	Biomass Burning-C3	-28.60‰	30.25	120.21	July, 2011	Active sampling	IRMS	Standard Calibration Method	0.3	https://doi.org/10.1016/j.atmosenv.2014.04.042
$\delta^{13}C$	EC	Biomass Burning-C3	-27.10‰	30.25	120.21	July, 2011	Active sampling	IRMS	Standard Calibration Method	0.3	https://doi.org/10.1016/j.atmosenv.2014.04.042
$\delta^{13}C$	EC	Biomass Burning-C3	-29.20‰	32.06	118.79	July, 2011	Active sampling	IRMS	Standard Calibration Method	0.3	https://doi.org/10.1016/j.atmosenv.2014.04.042

$\delta^{13}C$	EC	Biomass Burning-C3	-27.50‰	32.06	118.79	July, 2011	Active sampling	IRMS	Standard Calibration Method	0.3	https://doi.org/10.1016/j.atmosenv.2014.04.042
$\delta^{13}C$	EC	Biomass Burning-C3	-22.50‰	32.06	118.79	July, 2011	Active sampling	IRMS	Standard Calibration Method	0.3	https://doi.org/10.1016/j.atmosenv.2014.04.042
$\delta^{13}C$	EC	Biomass Burning-C3	-26.80‰	32.06	118.79	July, 2011	Active sampling	IRMS	Standard Calibration Method	0.3	https://doi.org/10.1016/j.atmosenv.2014.04.042
$\delta^{13}C$	EC	Biomass Burning-C3	-24.10‰	34.76	113.65	July, 2011	Active sampling	IRMS	Standard Calibration Method	0.3	https://doi.org/10.1016/j.atmosenv.2014.04.042
$\delta^{13}C$	EC	Biomass Burning-C3	-25.90‰	34.76	113.65	July, 2011	Active sampling	IRMS	Standard Calibration Method	0.3	https://doi.org/10.1016/j.atmosenv.2014.04.042
$\delta^{13}C$	EC	Biomass Burning-C3	-28.11‰	34.23	108.88	October, 2021	Active sampling	IRMS	Standard Calibration Method	0.19	https://doi.org/10.1016/j.scitotenv.2021.151284
$\delta^{13}C$	EC	Biomass Burning-C3	-27.62‰	34.23	108.88	July, 2021	Active sampling	IRMS	Standard Calibration Method	0.19	https://doi.org/10.1016/j.scitotenv.2021.151284
$\delta^{13}C$	EC	Biomass Burning-C3	-27.78‰	31.87	117.28	July, 2021	Active sampling	IRMS	Standard Calibration Method	0.19	https://doi.org/10.1016/j.scitotenv.2021.151284
$\delta^{13}C$	EC	Biomass Burning-C3	-28.30‰	20.51	78.96	July, 2011	Active sampling	IRMS	Standard Calibration Method	0.3	10.1016/j.atmosenv.2011.03.003
$\delta^{13}C$	EC	Biomass Burning-C3	-28.40‰	20.51	78.96	July, 2011	Active sampling	IRMS	Standard Calibration Method	0.3	10.1016/j.atmosenv.2011.03.003
$\delta^{13}C$	EC	Biomass Burning-C3	-26.90‰	20.51	78.96	July, 2011	Active sampling	IRMS	Standard Calibration Method	0.3	10.1016/j.atmosenv.2011.03.003
$\delta^{13}C$	EC	Biomass Burning-C3	-29.40‰	20.51	78.96	July, 2011	Active sampling	IRMS	Standard Calibration Method	0.3	10.1016/j.atmosenv.2011.03.003
$\delta^{13}C$	EC	Biomass Burning-C3	-28.80‰	20.51	78.96	July, 2011	Active sampling	IRMS	Standard Calibration Method	0.3	10.1016/j.atmosenv.2011.03.003
$\delta^{13}C$	EC	Biomass Burning-C3	-27.55‰	39.90	116.40	July, 2016	Active sampling	IRMS	Standard Calibration Method	0.1	10.1039/c6em00037a
$\delta^{13}C$	EC	Biomass Burning-C3	-28.83‰	32.06	118.79	July, 2015	Active sampling	IRMS	Standard Calibration Method	0.15	http://dx.doi.org/10.1016/j.atmosres.2015.09.006

$\delta^{13}C$	EC	Biomass Burning-C3	-28.86‰	32.06	118.79	July, 2015	Active sampling	IRMS	Standard Calibration Method	0.15	http://dx.doi.org/10.1016/j.atmosres.2015.09.006
$\delta^{13}C$	EC	Biomass Burning-C3	-30.42‰	32.06	118.79	July, 2015	Active sampling	IRMS	Standard Calibration Method	0.15	http://dx.doi.org/10.1016/j.atmosres.2015.09.006
$\delta^{13}C$	EC	Biomass Burning-C3	-29.81‰	32.06	118.79	July, 2015	Active sampling	IRMS	Standard Calibration Method	0.15	http://dx.doi.org/10.1016/j.atmosres.2015.09.006
$\delta^{13}C$	EC	Biomass Burning-C3	-29.83‰	32.06	118.79	July, 2015	Active sampling	IRMS	Standard Calibration Method	0.15	http://dx.doi.org/10.1016/j.atmosres.2015.09.006
$\delta^{13}C$	EC	Biomass Burning-C4	-16.10‰	31.32	138.25	April, 2011	Active sampling	IRMS	Standard Calibration Method	0.14	10.1016/j.atmosenv.2011.05.015
$\delta^{13}C$	EC	Biomass Burning-C4	-19.30‰	31.32	138.25	January, 2011	Active sampling	IRMS	Standard Calibration Method	0.14	10.1016/j.atmosenv.2011.05.015
$\delta^{13}C$	EC	Biomass Burning-C4	-14.60‰	23.13	113.26	July, 2020	Active sampling	IRMS	Standard Calibration Method	0.3	10.1029/2020JD033920
$\delta^{13}C$	EC	Biomass Burning-C4	-13.09‰	37.51	121.05	April, 2012	Active sampling	IRMS	Standard Calibration Method	0.3	10.13227/j.hjkx.2012.03.021 in Chinese
$\delta^{13}C$	EC	Biomass Burning-C4	-12.00‰	37.51	121.05	January, 2012	Active sampling	IRMS	Standard Calibration Method	0.3	10.13227/j.hjkx.2012.03.021 in Chinese
$\delta^{13}C$	EC	Biomass Burning-C4	-19.30‰	39.90	116.40	July, 2016	Active sampling	IRMS	Standard Calibration Method	0.2	10.13227/j.hjkx.2016.01.004 in Chinese
$\delta^{13}C$	EC	Biomass Burning-C4	-29.18‰	43.06	141.35	July, 2014	Active sampling	IRMS	Standard Calibration Method	0.3	http://dx.doi.org/10.1016/j.atmosenv.2014.05.045
$\delta^{13}C$	EC	Biomass Burning-C4	-13.78‰	51.17	10.45	April, 2021	Active sampling	IRMS	Standard Calibration Method	0.21	https://doi.org/10.1016/j.atmosenv.2021.118842
$\delta^{13}C$	EC	Biomass Burning-C4	-12.53‰	51.17	10.45	July, 2021	Active sampling	IRMS	Standard Calibration Method	0.21	https://doi.org/10.1016/j.atmosenv.2021.118842
$\delta^{13}C$	EC	Biomass Burning-C4	-10.94‰	51.17	10.45	October, 2021	Active sampling	IRMS	Standard Calibration Method	0.21	https://doi.org/10.1016/j.atmosenv.2021.118842
$\delta^{13}C$	EC	Biomass Burning-C4	-12.80‰	37.64	127.03	July, 2020	Active sampling	IRMS	Standard Calibration Method	0.3	https://doi.org/10.1016/j.envpol.2020.115163

$\delta^{13}C$	EC	Biomass Burning-C4	-18.42‰	38.03	114.47	July, 2021	Active sampling	IRMS	Standard Calibration Method	0.19	https://doi.org/10.1016/j.scitotenv.2021.151284
$\delta^{13}C$	EC	Biomass Burning-C4	-14.25‰	38.03	114.47	July, 2021	Active sampling	IRMS	Standard Calibration Method	0.19	https://doi.org/10.1016/j.scitotenv.2021.151284
$\delta^{13}C$	EC	Biomass Burning-C4	-13.10‰	32.06	118.79	July, 2011	Active sampling	IRMS	Standard Calibration Method	0.3	https://doi.org/10.1016/j.atmosenv.2014.04.042
$\delta^{13}C$	EC	Biomass Burning-C4	-14.00‰	32.06	118.79	July, 2011	Active sampling	IRMS	Standard Calibration Method	0.3	https://doi.org/10.1016/j.atmosenv.2014.04.042
$\delta^{13}C$	EC	Biomass Burning-C4	-16.80‰	32.06	118.79	July, 2011	Active sampling	IRMS	Standard Calibration Method	0.3	https://doi.org/10.1016/j.atmosenv.2014.04.042
$\delta^{13}C$	EC	Biomass Burning-C4	-12.80‰	34.76	113.65	July, 2012	Active sampling	IRMS	Standard Calibration Method	0.3	https://doi.org/10.1016/j.atmosenv.2014.04.042
$\delta^{13}C$	EC	Biomass Burning-C4	-13.60‰	34.76	113.65	July, 2012	Active sampling	IRMS	Standard Calibration Method	0.3	https://doi.org/10.1016/j.atmosenv.2014.04.042
$\delta^{13}C$	EC	Biomass Burning-C4	-12.20‰	34.27	108.90	July, 2012	Active sampling	IRMS	Standard Calibration Method	0.3	https://doi.org/10.1016/j.atmosenv.2014.04.042
$\delta^{13}C$	EC	Biomass Burning-C4	-22.20‰	32.06	118.79	July, 2012	Active sampling	IRMS	Standard Calibration Method	0.3	https://doi.org/10.1016/j.atmosenv.2014.04.042
$\delta^{13}C$	EC	Biomass Burning-C4	-17.50‰	32.06	118.79	July, 2012	Active sampling	IRMS	Standard Calibration Method	0.3	https://doi.org/10.1016/j.atmosenv.2014.04.042
$\delta^{13}C$	EC	Biomass Burning-C4	-15.60‰	32.06	118.79	July, 2012	Active sampling	IRMS	Standard Calibration Method	0.3	https://doi.org/10.1016/j.atmosenv.2014.04.042
$\delta^{13}C$	EC	Biomass Burning-C4	-15.80‰	34.76	113.65	July, 2012	Active sampling	IRMS	Standard Calibration Method	0.3	https://doi.org/10.1016/j.atmosenv.2014.04.042
$\delta^{13}C$	EC	Biomass Burning-C4	-15.00‰	34.76	113.65	July, 2012	Active sampling	IRMS	Standard Calibration Method	0.3	https://doi.org/10.1016/j.atmosenv.2014.04.042
$\delta^{13}C$	EC	Biomass Burning-C4	-13.50‰	32.06	118.79	July, 2012	Active sampling	IRMS	Standard Calibration Method	0.3	https://doi.org/10.1016/j.atmosenv.2014.04.042
$\delta^{13}C$	EC	Biomass Burning-C4	-13.00‰	32.06	118.79	July, 2012	Active sampling	IRMS	Standard Calibration Method	0.3	https://doi.org/10.1016/j.atmosenv.2014.04.042

$\delta^{13}C$	EC	Biomass Burning-C4	-13.90‰	34.76	113.65	July, 2012	Active sampling	IRMS	Standard Calibration Method	0.3	https://doi.org/10.1016/j.atmosenv.2014.04.042
$\delta^{13}C$	EC	Biomass Burning-C4	-13.90‰	34.76	113.65	July, 2012	Active sampling	IRMS	Standard Calibration Method	0.3	https://doi.org/10.1016/j.atmosenv.2014.04.042
$\delta^{13}C$	EC	Biomass Burning-C4	-14.40‰	32.06	118.79	July, 2012	Active sampling	IRMS	Standard Calibration Method	0.3	https://doi.org/10.1016/j.atmosenv.2014.04.042
$\delta^{13}C$	EC	Biomass Burning-C4	-14.60‰	32.06	118.79	July, 2012	Active sampling	IRMS	Standard Calibration Method	0.3	https://doi.org/10.1016/j.atmosenv.2014.04.042
$\delta^{13}C$	EC	Biomass Burning-C4	-15.40‰	32.06	118.79	July, 2012	Active sampling	IRMS	Standard Calibration Method	0.3	https://doi.org/10.1016/j.atmosenv.2014.04.042
$\delta^{13}C$	EC	Biomass Burning-C4	-14.80‰	34.76	113.65	July, 2012	Active sampling	IRMS	Standard Calibration Method	0.3	https://doi.org/10.1016/j.atmosenv.2014.04.042
$\delta^{13}C$	EC	Biomass Burning-C4	-15.40‰	34.76	113.65	July, 2012	Active sampling	IRMS	Standard Calibration Method	0.3	https://doi.org/10.1016/j.atmosenv.2014.04.042
$\delta^{13}C$	EC	Biomass Burning-C4	-15.60‰	34.27	108.90	July, 2012	Active sampling	IRMS	Standard Calibration Method	0.3	https://doi.org/10.1016/j.atmosenv.2014.04.042
$\delta^{13}C$	EC	Biomass Burning-C4	-13.60‰	32.06	118.79	July, 2012	Active sampling	IRMS	Standard Calibration Method	0.3	https://doi.org/10.1016/j.atmosenv.2014.04.042
$\delta^{13}C$	EC	Biomass Burning-C4	-14.30‰	32.06	118.79	July, 2012	Active sampling	IRMS	Standard Calibration Method	0.3	https://doi.org/10.1016/j.atmosenv.2014.04.042
$\delta^{13}C$	EC	Biomass Burning-C4	-15.30‰	32.06	118.79	July, 2012	Active sampling	IRMS	Standard Calibration Method	0.3	https://doi.org/10.1016/j.atmosenv.2014.04.042
$\delta^{13}C$	EC	Biomass Burning-C4	-14.30‰	34.76	113.65	July, 2012	Active sampling	IRMS	Standard Calibration Method	0.3	https://doi.org/10.1016/j.atmosenv.2014.04.042
$\delta^{13}C$	EC	Biomass Burning-C4	-12.30‰	34.76	113.65	July, 2012	Active sampling	IRMS	Standard Calibration Method	0.3	https://doi.org/10.1016/j.atmosenv.2014.04.042
$\delta^{13}C$	EC	Biomass Burning-C4	-18.90‰	34.27	108.90	July, 2012	Active sampling	IRMS	Standard Calibration Method	0.3	https://doi.org/10.1016/j.atmosenv.2014.04.042
$\delta^{13}C$	EC	Biomass Burning-C4	-19.12‰	38.03	114.47	July, 2021	Active sampling	IRMS	Standard Calibration Method	0.19	https://doi.org/10.1016/j.scitotenv.2021.151284

$\delta^{13}C$	EC	Biomass Burning-C4	-14.27‰	38.03	114.47	July, 2021	Active sampling	IRMS	Standard Calibration Method	0.19	https://doi.org/10.1016/j.scitotenv.2021.151284
$\delta^{13}C$	EC	Biomass Burning-C4	-19.30‰	32.06	118.79	July, 2015	Active sampling	IRMS	Standard Calibration Method	0.15	http://dx.doi.org/10.1016/j.atmosres.2015.09.006
$\delta^{13}C$	EC	Coal Combustion	-24.40‰	48.86	2.35	July, 2004	Active sampling	IRMS	Standard Calibration Method	0.1	10.1016/j.atmosenv.2003.11.001
$\delta^{13}C$	EC	Coal Combustion	-24.00‰	48.86	2.35	July, 2004	Active sampling	IRMS	Standard Calibration Method	0.1	10.1016/j.atmosenv.2003.11.001
$\delta^{13}C$	EC	Coal Combustion	-24.00‰	48.86	2.35	July, 2004	Active sampling	IRMS	Standard Calibration Method	0.1	10.1016/j.atmosenv.2003.11.001
$\delta^{13}C$	EC	Coal Combustion	-23.91‰	48.86	2.35	July, 2004	Active sampling	IRMS	Standard Calibration Method	0.1	10.1016/j.atmosenv.2003.11.001
$\delta^{13}C$	EC	Coal Combustion	-23.42‰	48.86	2.35	July, 2004	Active sampling	IRMS	Standard Calibration Method	0.1	10.1016/j.atmosenv.2003.11.001
$\delta^{13}C$	EC	Coal Combustion	-24.01‰	48.86	2.35	July, 2004	Active sampling	IRMS	Standard Calibration Method	0.1	10.1016/j.atmosenv.2003.11.001
$\delta^{13}C$	EC	Coal Combustion	-24.01‰	48.86	2.35	July, 2004	Active sampling	IRMS	Standard Calibration Method	0.1	10.1016/j.atmosenv.2003.11.001
$\delta^{13}C$	EC	Coal Combustion	-24.00‰	48.86	2.35	July, 2004	Active sampling	IRMS	Standard Calibration Method	0.1	10.1016/j.atmosenv.2003.11.001
$\delta^{13}C$	EC	Coal Combustion	-23.30‰	31.32	138.25	April, 2011	Active sampling	IRMS	Standard Calibration Method	0.14	10.1016/j.atmosenv.2011.05.015
$\delta^{13}C$	EC	Coal Combustion	-27.40‰	31.32	138.25	January, 2011	Active sampling	IRMS	Standard Calibration Method	0.14	10.1016/j.atmosenv.2011.05.015
$\delta^{13}C$	EC	Coal Combustion	-23.40‰	23.13	113.26	July, 2020	Active sampling	IRMS	Standard Calibration Method	0.3	10.1029/2020JD033920
$\delta^{13}C$	EC	Coal Combustion	-23.31‰	39.90	116.40	July, 2016	Active sampling	IRMS	Standard Calibration Method	0.1	10.1039/c6em00037a
$\delta^{13}C$	EC	Coal Combustion	-23.14‰	38.29	109.73	April, 2012	Active sampling	IRMS	Standard Calibration Method	0.3	10.13227/j.hjkx.2012.03.021 in Chinese

$\delta^{13}C$	EC	Coal Combustion	-23.12‰	37.34	111.18	January, 2012	Active sampling	IRMS	Standard Calibration Method	0.3	10.13227/j.hjkx.2012.03.021 in Chinese
$\delta^{13}C$	EC	Coal Combustion	-24.08‰	36.32	114.37	July, 2012	Active sampling	IRMS	Standard Calibration Method	0.3	10.13227/j.hjkx.2012.03.021 in Chinese
$\delta^{13}C$	EC	Coal Combustion	-23.88‰	36.20	113.12	October, 2012	Active sampling	IRMS	Standard Calibration Method	0.3	10.13227/j.hjkx.2012.03.021 in Chinese
$\delta^{13}C$	EC	Coal Combustion	-25.50‰	40.82	111.77	July, 2016	Active sampling	IRMS	Standard Calibration Method	0.2	10.13227/j.hjkx.2016.01.004 in Chinese
$\delta^{13}C$	EC	Coal Combustion	-25.39‰	40.08	113.30	July, 2016	Active sampling	IRMS	Standard Calibration Method	0.2	10.13227/j.hjkx.2016.01.004 in Chinese
$\delta^{13}C$	EC	Coal Combustion	-23.57‰	34.20	117.29	July, 2016	Active sampling	IRMS	Standard Calibration Method	0.2	10.13227/j.hjkx.2016.01.004 in Chinese
$\delta^{13}C$	EC	Coal Combustion	-24.56‰	43.06	141.35	July, 2014	Active sampling	IRMS	Standard Calibration Method	0.3	http://dx.doi.org/10.1016/j.atmosenv.2014.05.045
$\delta^{13}C$	EC	Coal Combustion	-26.15‰	32.06	118.79	July, 2015	Active sampling	IRMS	Standard Calibration Method	0.15	http://dx.doi.org/10.1016/j.atmosres.2015.09.006
$\delta^{13}C$	EC	Coal Combustion	-24.39‰	32.06	118.79	July, 2015	Active sampling	IRMS	Standard Calibration Method	0.15	http://dx.doi.org/10.1016/j.atmosres.2015.09.006
$\delta^{13}C$	EC	Coal Combustion	-22.17‰	32.06	118.79	July, 2015	Active sampling	IRMS	Standard Calibration Method	0.15	http://dx.doi.org/10.1016/j.atmosres.2015.09.006
$\delta^{13}C$	EC	Coal Combustion	-26.70‰	51.17	10.45	April, 2021	Active sampling	IRMS	Standard Calibration Method	0.21	https://doi.org/10.1016/j.atmosenv.2021.118842
$\delta^{13}C$	EC	Coal Combustion	-24.89‰	61.53	105.32	July, 2021	Active sampling	IRMS	Standard Calibration Method	0.21	https://doi.org/10.1016/j.atmosenv.2021.118842
$\delta^{13}C$	EC	Coal Combustion	-25.93‰	51.17	10.45	October, 2021	Active sampling	IRMS	Standard Calibration Method	0.21	https://doi.org/10.1016/j.atmosenv.2021.118842
$\delta^{13}C$	EC	Coal Combustion	-25.57‰	61.53	105.32	April, 2021	Active sampling	IRMS	Standard Calibration Method	0.21	https://doi.org/10.1016/j.atmosenv.2021.118842
$\delta^{13}C$	EC	Coal Combustion	-22.36‰	51.17	10.45	July, 2021	Active sampling	IRMS	Standard Calibration Method	0.21	https://doi.org/10.1016/j.atmosenv.2021.118842

$\delta^{13}C$	EC	Coal Combustion	-25.49‰	61.53	105.32	October, 2021	Active sampling	IRMS	Standard Calibration Method	0.21	https://doi.org/10.1016/j.atmosenv.2021.118842
$\delta^{13}C$	EC	Coal Combustion	-23.78‰	37.54	112.33	July, 2020	Active sampling	IRMS	Standard Calibration Method	0.3	https://doi.org/10.1016/j.envpol.2020.115768
$\delta^{13}C$	EC	Coal Combustion	-23.66‰	37.54	112.33	July, 2020	Active sampling	IRMS	Standard Calibration Method	0.3	https://doi.org/10.1016/j.envpol.2020.115768
$\delta^{13}C$	EC	Coal Combustion	-23.74‰	37.54	112.33	July, 2020	Active sampling	IRMS	Standard Calibration Method	0.3	https://doi.org/10.1016/j.envpol.2020.115768
$\delta^{13}C$	EC	Coal Combustion	-23.56‰	37.54	112.33	July, 2020	Active sampling	IRMS	Standard Calibration Method	0.3	https://doi.org/10.1016/j.envpol.2020.115768
$\delta^{13}C$	EC	Coal Combustion	-23.50‰	37.54	112.33	July, 2020	Active sampling	IRMS	Standard Calibration Method	0.3	https://doi.org/10.1016/j.envpol.2020.115768
$\delta^{13}C$	EC	Coal Combustion	-23.74‰	37.54	112.33	July, 2020	Active sampling	IRMS	Standard Calibration Method	0.3	https://doi.org/10.1016/j.envpol.2020.115768
$\delta^{13}C$	EC	Coal Combustion	-23.40‰	37.64	127.03	July, 2020	Active sampling	IRMS	Standard Calibration Method	0.3	https://doi.org/10.1016/j.envpol.2020.115163
$\delta^{13}C$	EC	Coal Combustion	-25.16‰	34.23	108.88	July, 2021	Active sampling	IRMS	Standard Calibration Method	0.19	https://doi.org/10.1016/j.scitotenv.2021.151284
$\delta^{13}C$	EC	Coal Combustion	-24.37‰	34.23	108.88	July, 2021	Active sampling	IRMS	Standard Calibration Method	0.19	https://doi.org/10.1016/j.scitotenv.2021.151284
$\delta^{13}C$	EC	Coal Combustion	-24.58‰	38.47	106.26	July, 2021	Active sampling	IRMS	Standard Calibration Method	0.19	https://doi.org/10.1016/j.scitotenv.2021.151284
$\delta^{13}C$	EC	Coal Combustion	-24.93‰	37.54	112.33	July, 2021	Active sampling	IRMS	Standard Calibration Method	0.19	https://doi.org/10.1016/j.scitotenv.2021.151284
$\delta^{13}C$	EC	Coal Combustion	-25.28‰	40.82	111.77	July, 2021	Active sampling	IRMS	Standard Calibration Method	0.19	https://doi.org/10.1016/j.scitotenv.2021.151284
$\delta^{13}C$	EC	Coal Combustion	-23.60‰	37.54	112.33	July, 2012	Active sampling	IRMS	Standard Calibration Method	0.12	Isotopic compositions of carbon in PM10 and its emission sources in Taiyuan. in Chinese
$\delta^{13}C$	EC	Coal Combustion	-23.40‰	59.33	18.05	July, 2016	Active sampling	IRMS	Standard Calibration Method	0.3	www.pnas.org/cgi/doi/10.1073/pnas.1613401114

$\delta^{13}C$	EC	Coal Combustion	-23.77‰	37.54	112.33	July, 2020	Active sampling	IRMS	Standard Calibration Method	0.3	https://doi.org/10.1016/j.envpol.2020.115768
$\delta^{13}C$	EC	Coal Combustion	-23.82‰	37.54	112.33	July, 2020	Active sampling	IRMS	Standard Calibration Method	0.3	https://doi.org/10.1016/j.envpol.2020.115768
$\delta^{13}C$	EC	Coal Combustion	-23.77‰	37.54	112.33	July, 2020	Active sampling	IRMS	Standard Calibration Method	0.3	https://doi.org/10.1016/j.envpol.2020.115768
$\delta^{13}C$	EC	Coal Combustion	-23.57‰	37.54	112.33	July, 2020	Active sampling	IRMS	Standard Calibration Method	0.3	https://doi.org/10.1016/j.envpol.2020.115768
$\delta^{13}C$	EC	Coal Combustion	-23.80‰	37.54	112.33	July, 2020	Active sampling	IRMS	Standard Calibration Method	0.3	https://doi.org/10.1016/j.envpol.2020.115768
$\delta^{13}C$	EC	Coal Combustion	-24.02‰	37.54	112.33	July, 2020	Active sampling	IRMS	Standard Calibration Method	0.3	https://doi.org/10.1016/j.envpol.2020.115768
$\delta^{13}C$	EC	Coal Combustion	-25.17‰	34.23	108.88	July, 2021	Active sampling	IRMS	Standard Calibration Method	0.19	https://doi.org/10.1016/j.scitotenv.2021.151284
$\delta^{13}C$	EC	Coal Combustion	-24.47‰	34.23	108.88	July, 2021	Active sampling	IRMS	Standard Calibration Method	0.19	https://doi.org/10.1016/j.scitotenv.2021.151284
$\delta^{13}C$	EC	Coal Combustion	-24.60‰	38.47	106.26	July, 2021	Active sampling	IRMS	Standard Calibration Method	0.19	https://doi.org/10.1016/j.scitotenv.2021.151284
$\delta^{13}C$	EC	Coal Combustion	-24.93‰	37.54	112.33	July, 2021	Active sampling	IRMS	Standard Calibration Method	0.19	https://doi.org/10.1016/j.scitotenv.2021.151284
$\delta^{13}C$	EC	Coal Combustion	-25.28‰	40.82	111.77	July, 2021	Active sampling	IRMS	Standard Calibration Method	0.19	https://doi.org/10.1016/j.scitotenv.2021.151284
$\delta^{13}C$	EC	Coal Combustion	-23.20‰	37.54	112.33	July, 2012	Active sampling	IRMS	Standard Calibration Method	0.12	Isotopic compositions of carbon in PM10 and its emission sources in Taiyuan. in Chinese
$\delta^{13}C$	EC	Vehicle Exhausts	-25.69‰	48.86	2.35	July, 2004	Active sampling	IRMS	Standard Calibration Method	0.1	10.1016/j.atmosenv.2003.11.001
$\delta^{13}C$	EC	Vehicle Exhausts	-25.89‰	48.86	2.35	July, 2004	Active sampling	IRMS	Standard Calibration Method	0.1	10.1016/j.atmosenv.2003.11.001
$\delta^{13}C$	EC	Vehicle Exhausts	-26.09‰	48.86	2.35	July, 2004	Active sampling	IRMS	Standard Calibration Method	0.1	10.1016/j.atmosenv.2003.11.001

$\delta^{13}C$	EC	Vehicle Exhausts	-26.79‰	48.86	2.35	July, 2004	Active sampling	IRMS	Standard Calibration Method	0.1	10.1016/j.atmosenv.2003.11.001
$\delta^{13}C$	EC	Vehicle Exhausts	-25.40‰	48.86	2.35	July, 2004	Active sampling	IRMS	Standard Calibration Method	0.1	10.1016/j.atmosenv.2003.11.001
$\delta^{13}C$	EC	Vehicle Exhausts	-25.60‰	23.13	113.26	July, 2020	Active sampling	IRMS	Standard Calibration Method	0.3	10.1029/2020JD033920
$\delta^{13}C$	EC	Vehicle Exhausts	-25.50‰	37.64	127.03	July, 2020	Active sampling	IRMS	Standard Calibration Method	0.3	https://doi.org/10.1016/j.envpol.2020.115163
$\delta^{13}C$	EC	Vehicle Exhausts	-26.02‰	43.06	141.35	July, 2011	Active sampling	IRMS	Standard Calibration Method	0.3	10.1029/2011JD015617
$\delta^{13}C$	EC	Vehicle Exhausts	-25.11‰	48.86	2.35	July, 2004	Active sampling	IRMS	Standard Calibration Method	0.1	10.1016/j.atmosenv.2003.11.001
$\delta^{13}C$	EC	Vehicle Exhausts	-24.71‰	48.86	2.35	July, 2004	Active sampling	IRMS	Standard Calibration Method	0.1	10.1016/j.atmosenv.2003.11.001
$\delta^{13}C$	EC	Vehicle Exhausts	-24.81‰	48.86	2.35	July, 2004	Active sampling	IRMS	Standard Calibration Method	0.1	10.1016/j.atmosenv.2003.11.001
$\delta^{13}C$	EC	Vehicle Exhausts	-24.01‰	48.86	2.35	July, 2004	Active sampling	IRMS	Standard Calibration Method	0.1	10.1016/j.atmosenv.2003.11.001
$\delta^{13}C$	EC	Vehicle Exhausts	-23.53‰	48.86	2.35	July, 2004	Active sampling	IRMS	Standard Calibration Method	0.1	10.1016/j.atmosenv.2003.11.001
$\delta^{13}C$	EC	Vehicle Exhausts	-23.82‰	48.86	2.35	July, 2004	Active sampling	IRMS	Standard Calibration Method	0.1	10.1016/j.atmosenv.2003.11.001
$\delta^{13}C$	EC	Vehicle Exhausts	-23.72‰	48.86	2.35	July, 2004	Active sampling	IRMS	Standard Calibration Method	0.1	10.1016/j.atmosenv.2003.11.001
$\delta^{13}C$	EC	Vehicle Exhausts	-25.11‰	48.86	2.35	July, 2004	Active sampling	IRMS	Standard Calibration Method	0.1	10.1016/j.atmosenv.2003.11.001
$\delta^{13}C$	EC	Vehicle Exhausts	-25.28‰	48.86	2.35	July, 2004	Active sampling	IRMS	Standard Calibration Method	0.1	10.1016/j.atmosenv.2003.11.001
$\delta^{13}C$	EC	Vehicle Exhausts	-24.08‰	48.86	2.35	July, 2004	Active sampling	IRMS	Standard Calibration Method	0.1	10.1016/j.atmosenv.2003.11.001

$\delta^{13}C$	EC	Vehicle Exhausts	-24.67‰	48.86	2.35	July, 2004	Active sampling	IRMS	Standard Calibration Method	0.1	10.1016/j.atmosenv.2003.11.001
$\delta^{13}C$	EC	Vehicle Exhausts	-24.88‰	48.86	2.35	July, 2004	Active sampling	IRMS	Standard Calibration Method	0.1	10.1016/j.atmosenv.2003.11.001
$\delta^{13}C$	EC	Vehicle Exhausts	-24.08‰	48.86	2.35	July, 2004	Active sampling	IRMS	Standard Calibration Method	0.1	10.1016/j.atmosenv.2003.11.001
$\delta^{13}C$	EC	Vehicle Exhausts	-23.79‰	48.86	2.35	July, 2004	Active sampling	IRMS	Standard Calibration Method	0.1	10.1016/j.atmosenv.2003.11.001
$\delta^{13}C$	EC	Vehicle Exhausts	-23.89‰	48.86	2.35	July, 2004	Active sampling	IRMS	Standard Calibration Method	0.1	10.1016/j.atmosenv.2003.11.001
$\delta^{13}C$	EC	Vehicle Exhausts	-24.88‰	48.86	2.35	July, 2004	Active sampling	IRMS	Standard Calibration Method	0.1	10.1016/j.atmosenv.2003.11.001
$\delta^{13}C$	EC	Vehicle Exhausts	-24.88‰	48.86	2.35	July, 2004	Active sampling	IRMS	Standard Calibration Method	0.1	10.1016/j.atmosenv.2003.11.001
$\delta^{13}C$	EC	Vehicle Exhausts	-23.98‰	48.86	2.35	July, 2004	Active sampling	IRMS	Standard Calibration Method	0.1	10.1016/j.atmosenv.2003.11.001
$\delta^{13}C$	EC	Vehicle Exhausts	-24.08‰	48.86	2.35	July, 2004	Active sampling	IRMS	Standard Calibration Method	0.1	10.1016/j.atmosenv.2003.11.001
$\delta^{13}C$	EC	Vehicle Exhausts	-23.97‰	48.86	2.35	July, 2004	Active sampling	IRMS	Standard Calibration Method	0.1	10.1016/j.atmosenv.2003.11.001
$\delta^{13}C$	EC	Vehicle Exhausts	-22.59‰	48.86	2.35	July, 2004	Active sampling	IRMS	Standard Calibration Method	0.1	10.1016/j.atmosenv.2003.11.001
$\delta^{13}C$	EC	Vehicle Exhausts	-25.77‰	48.86	2.35	July, 2004	Active sampling	IRMS	Standard Calibration Method	0.1	10.1016/j.atmosenv.2003.11.001
$\delta^{13}C$	EC	Vehicle Exhausts	-27.19‰	48.86	2.35	July, 2004	Active sampling	IRMS	Standard Calibration Method	0.1	10.1016/j.atmosenv.2003.11.001
$\delta^{13}C$	EC	Vehicle Exhausts	-26.99‰	48.86	2.35	July, 2004	Active sampling	IRMS	Standard Calibration Method	0.1	10.1016/j.atmosenv.2003.11.001
$\delta^{13}C$	EC	Vehicle Exhausts	-26.59‰	48.86	2.35	July, 2004	Active sampling	IRMS	Standard Calibration Method	0.1	10.1016/j.atmosenv.2003.11.001

$\delta^{13}C$	EC	Vehicle Exhausts	-27.08‰	48.86	2.35	July, 2004	Active sampling	IRMS	Standard Calibration Method	0.1	10.1016/j.atmosenv.2003.11.001
$\delta^{13}C$	EC	Vehicle Exhausts	-27.11‰	48.86	2.35	July, 2004	Active sampling	IRMS	Standard Calibration Method	0.1	10.1016/j.atmosenv.2003.11.001
$\delta^{13}C$	EC	Vehicle Exhausts	-26.98‰	48.86	2.35	July, 2004	Active sampling	IRMS	Standard Calibration Method	0.1	10.1016/j.atmosenv.2003.11.001
$\delta^{13}C$	EC	Vehicle Exhausts	-27.19‰	48.86	2.35	July, 2004	Active sampling	IRMS	Standard Calibration Method	0.1	10.1016/j.atmosenv.2003.11.001
$\delta^{13}C$	EC	Vehicle Exhausts	-26.68‰	48.86	2.35	July, 2004	Active sampling	IRMS	Standard Calibration Method	0.1	10.1016/j.atmosenv.2003.11.001
$\delta^{13}C$	EC	Vehicle Exhausts	-26.49‰	48.86	2.35	July, 2004	Active sampling	IRMS	Standard Calibration Method	0.1	10.1016/j.atmosenv.2003.11.001
$\delta^{13}C$	EC	Vehicle Exhausts	-26.19‰	48.86	2.35	July, 2004	Active sampling	IRMS	Standard Calibration Method	0.1	10.1016/j.atmosenv.2003.11.001
$\delta^{13}C$	EC	Vehicle Exhausts	-26.19‰	48.86	2.35	July, 2004	Active sampling	IRMS	Standard Calibration Method	0.1	10.1016/j.atmosenv.2003.11.001
$\delta^{13}C$	EC	Vehicle Exhausts	-26.39‰	48.86	2.35	July, 2004	Active sampling	IRMS	Standard Calibration Method	0.1	10.1016/j.atmosenv.2003.11.001
$\delta^{13}C$	EC	Vehicle Exhausts	-26.18‰	48.86	2.35	July, 2004	Active sampling	IRMS	Standard Calibration Method	0.1	10.1016/j.atmosenv.2003.11.001
$\delta^{13}C$	EC	Vehicle Exhausts	-26.01‰	48.86	2.35	July, 2004	Active sampling	IRMS	Standard Calibration Method	0.1	10.1016/j.atmosenv.2003.11.001
$\delta^{13}C$	EC	Vehicle Exhausts	-26.18‰	48.86	2.35	July, 2004	Active sampling	IRMS	Standard Calibration Method	0.1	10.1016/j.atmosenv.2003.11.001
$\delta^{13}C$	EC	Vehicle Exhausts	-26.09‰	48.86	2.35	July, 2004	Active sampling	IRMS	Standard Calibration Method	0.1	10.1016/j.atmosenv.2003.11.001
$\delta^{13}C$	EC	Vehicle Exhausts	-22.67‰	19.43	-99.13	July, 2003	Active sampling	IRMS	Standard Calibration Method	0.1	10.1016/j.atmosenv.2009.06.036
$\delta^{13}C$	EC	Vehicle Exhausts	-22.80‰	19.43	-99.13	June, 2002	Active sampling	IRMS	Standard Calibration Method	0.1	10.1016/j.atmosenv.2009.06.036

$\delta^{13}C$	EC	Vehicle Exhausts	-24.69‰	19.43	-99.13	March, 2002	Active sampling	IRMS	Standard Calibration Method	0.1	10.1016/j.atmosenv.2009.06.036
$\delta^{13}C$	EC	Vehicle Exhausts	-24.86‰	19.43	-99.13	March, 2002	Active sampling	IRMS	Standard Calibration Method	0.1	10.1016/j.atmosenv.2009.06.036
$\delta^{13}C$	EC	Vehicle Exhausts	-24.88‰	19.43	-99.13	June, 2002	Active sampling	IRMS	Standard Calibration Method	0.1	10.1016/j.atmosenv.2009.06.036
$\delta^{13}C$	EC	Vehicle Exhausts	-24.90‰	19.43	-99.13	March, 2002	Active sampling	IRMS	Standard Calibration Method	0.1	10.1016/j.atmosenv.2009.06.036
$\delta^{13}C$	EC	Vehicle Exhausts	-25.18‰	19.43	-99.13	March, 2002	Active sampling	IRMS	Standard Calibration Method	0.1	10.1016/j.atmosenv.2009.06.036
$\delta^{13}C$	EC	Vehicle Exhausts	-27.29‰	19.43	-99.13	June, 2002	Active sampling	IRMS	Standard Calibration Method	0.1	10.1016/j.atmosenv.2009.06.036
$\delta^{13}C$	EC	Vehicle Exhausts	-27.31‰	19.43	-99.13	July, 2009	Active sampling	IRMS	Standard Calibration Method	0.1	10.1016/j.atmosenv.2009.06.036
$\delta^{13}C$	EC	Vehicle Exhausts	-28.57‰	19.43	-99.13	June, 2002	Active sampling	IRMS	Standard Calibration Method	0.1	10.1016/j.atmosenv.2009.06.036
$\delta^{13}C$	EC	Vehicle Exhausts	-26.40‰	40.46	-3.75	12 December, 2008	Active sampling	IRMS	Standard Calibration Method	0.3	10.1016/j.atmosenv.2011.01.029
$\delta^{13}C$	EC	Vehicle Exhausts	-24.40‰	31.32	138.25	July, 2011	Active sampling	IRMS	Standard Calibration Method	0.14	10.1016/j.atmosenv.2011.05.015
$\delta^{13}C$	EC	Vehicle Exhausts	-24.10‰	31.32	138.25	October, 2011	Active sampling	IRMS	Standard Calibration Method	0.14	10.1016/j.atmosenv.2011.05.015
$\delta^{13}C$	EC	Vehicle Exhausts	-20.60‰	31.32	138.25	July, 2011	Active sampling	IRMS	Standard Calibration Method	0.14	10.1016/j.atmosenv.2011.05.015
$\delta^{13}C$	EC	Vehicle Exhausts	-24.30‰	31.32	138.25	October, 2011	Active sampling	IRMS	Standard Calibration Method	0.14	10.1016/j.atmosenv.2011.05.015
$\delta^{13}C$	EC	Vehicle Exhausts	-24.40‰	31.32	138.25	July, 2011	Active sampling	IRMS	Standard Calibration Method	0.14	10.1016/j.atmosenv.2011.05.015
$\delta^{13}C$	EC	Vehicle Exhausts	-24.20‰	31.32	138.25	October, 2011	Active sampling	IRMS	Standard Calibration Method	0.14	10.1016/j.atmosenv.2011.05.015

$\delta^{13}C$	EC	Vehicle Exhausts	-24.90‰	31.32	138.25	October, 2011	Active sampling	IRMS	Standard Calibration Method	0.14	10.1016/j.atmosenv.2011.05.015
$\delta^{13}C$	EC	Vehicle Exhausts	-26.50‰	43.06	141.35	July, 2011	Active sampling	IRMS	Standard Calibration Method	0.3	10.1029/2011JD015617
$\delta^{13}C$	EC	Vehicle Exhausts	-24.20‰	43.06	141.35	July, 2011	Active sampling	IRMS	Standard Calibration Method	0.3	10.1029/2011JD015617
$\delta^{13}C$	EC	Vehicle Exhausts	-24.52‰	43.06	141.35	July, 2011	Active sampling	IRMS	Standard Calibration Method	0.3	10.1029/2011JD015617
$\delta^{13}C$	EC	Vehicle Exhausts	-24.40‰	39.38	140.05	July, 2016	Active sampling	IRMS	Standard Calibration Method	0.1	10.1039/c6em00037a
$\delta^{13}C$	EC	Vehicle Exhausts	-24.30‰	39.38	140.05	July, 2016	Active sampling	IRMS	Standard Calibration Method	0.1	10.1039/c6em00037a
$\delta^{13}C$	EC	Vehicle Exhausts	-24.92‰	37.51	121.05	April, 2012	Active sampling	IRMS	Standard Calibration Method	0.3	10.13227/j.hjkx.2012.03.021 in Chinese
$\delta^{13}C$	EC	Vehicle Exhausts	-24.97‰	37.51	121.05	January, 2012	Active sampling	IRMS	Standard Calibration Method	0.3	10.13227/j.hjkx.2012.03.021 in Chinese
$\delta^{13}C$	EC	Vehicle Exhausts	-25.39‰	37.51	121.05	July, 2012	Active sampling	IRMS	Standard Calibration Method	0.3	10.13227/j.hjkx.2012.03.021 in Chinese
$\delta^{13}C$	EC	Vehicle Exhausts	-25.77‰	37.51	121.05	October, 2012	Active sampling	IRMS	Standard Calibration Method	0.3	10.13227/j.hjkx.2012.03.021 in Chinese
$\delta^{13}C$	EC	Vehicle Exhausts	-25.41‰	37.51	121.05	October, 2012	Active sampling	IRMS	Standard Calibration Method	0.3	10.13227/j.hjkx.2012.03.021 in Chinese
$\delta^{13}C$	EC	Vehicle Exhausts	-25.19‰	39.90	116.40	July, 2016	Active sampling	IRMS	Standard Calibration Method	0.2	10.13227/j.hjkx.2016.01.004 in Chinese
$\delta^{13}C$	EC	Vehicle Exhausts	-26.26‰	39.90	116.40	July, 2016	Active sampling	IRMS	Standard Calibration Method	0.2	10.13227/j.hjkx.2016.01.004 in Chinese
$\delta^{13}C$	EC	Vehicle Exhausts	-26.32‰	39.90	116.40	July, 2016	Active sampling	IRMS	Standard Calibration Method	0.2	10.13227/j.hjkx.2016.01.004 in Chinese
$\delta^{13}C$	EC	Vehicle Exhausts	-24.97‰	39.90	116.40	July, 2016	Active sampling	IRMS	Standard Calibration Method	0.2	10.13227/j.hjkx.2016.01.004 in Chinese

$\delta^{13}C$	EC	Vehicle Exhausts	-23.57‰	39.90	116.40	July, 2016	Active sampling	IRMS	Standard Calibration Method	0.2	10.13227/j.hjkx.2016.01.004 in Chinese
$\delta^{13}C$	EC	Vehicle Exhausts	-26.00‰	32.50	118.82	26-30 August, 2020	Active sampling	IRMS	Standard Calibration Method	0.15	10.13671/j.hjkxxb.2021.0226 in Chinese
$\delta^{13}C$	EC	Vehicle Exhausts	-26.68‰	43.06	141.35	July, 2014	Active sampling	IRMS	Standard Calibration Method	0.3	http://dx.doi.org/10.1016/j.atmosenv.2014.05.045
$\delta^{13}C$	EC	Vehicle Exhausts	-24.97‰	43.06	141.35	July, 2014	Active sampling	IRMS	Standard Calibration Method	0.3	http://dx.doi.org/10.1016/j.atmosenv.2014.05.045
$\delta^{13}C$	EC	Vehicle Exhausts	-25.19‰	32.06	118.79	July, 2015	Active sampling	IRMS	Standard Calibration Method	0.15	http://dx.doi.org/10.1016/j.atmosres.2015.09.006
$\delta^{13}C$	EC	Vehicle Exhausts	-26.26‰	32.06	118.79	July, 2015	Active sampling	IRMS	Standard Calibration Method	0.15	http://dx.doi.org/10.1016/j.atmosres.2015.09.006
$\delta^{13}C$	EC	Vehicle Exhausts	-26.32‰	32.06	118.79	July, 2015	Active sampling	IRMS	Standard Calibration Method	0.15	http://dx.doi.org/10.1016/j.atmosres.2015.09.006
$\delta^{13}C$	EC	Vehicle Exhausts	-23.57‰	32.06	118.79	July, 2015	Active sampling	IRMS	Standard Calibration Method	0.15	http://dx.doi.org/10.1016/j.atmosres.2015.09.006
$\delta^{13}C$	EC	Vehicle Exhausts	-26.30‰	21.53	-77.78	July, 2018	Active sampling	IRMS	Standard Calibration Method	0.2	https://doi.org/10.1016/j.scitotenv.2018.06.106
$\delta^{13}C$	EC	Vehicle Exhausts	-25.20‰	21.53	-77.78	July, 2018	Active sampling	IRMS	Standard Calibration Method	0.2	https://doi.org/10.1016/j.scitotenv.2018.06.106
$\delta^{13}C$	EC	Vehicle Exhausts	-25.70‰	21.53	-77.78	July, 2018	Active sampling	IRMS	Standard Calibration Method	0.2	https://doi.org/10.1016/j.scitotenv.2018.06.106
$\delta^{13}C$	EC	Vehicle Exhausts	-27.00‰	37.54	112.33	July, 2012	Active sampling	IRMS	Standard Calibration Method	0.12	Isotopic compositions of carbon in PM10 and its emission sources in Taiyuan. in Chinese
$\delta^{13}C$	EC	Vehicle Exhausts	-24.80‰	37.54	112.33	July, 2012	Active sampling	IRMS	Standard Calibration Method	0.12	Isotopic compositions of carbon in PM10 and its emission sources in Taiyuan. in Chinese
$\delta^{13}C$	EC	Vehicle Exhausts	-31.40‰	59.33	18.05	July, 2016	Active sampling	IRMS	Standard Calibration Method	0.3	www.pnas.org/cgi/doi/10.1073/pnas.1613401114

$\delta^{13}C$	EC	Vehicle Exhausts	-25.50‰	37.54	112.33	July, 2012	Active sampling	IRMS	Standard Calibration Method	0.12	Isotopic compositions of carbon in PM10 and its emission sources in Taiyuan. in Chinese
$\delta^{13}C$	EC	Vehicle Exhausts	-24.30‰	37.54	112.33	July, 2012	Active sampling	IRMS	Standard Calibration Method	0.12	Isotopic compositions of carbon in PM10 and its emission sources in Taiyuan. in Chinese
$\delta^{13}C$	OC	Biomass Burning-C3	-27.32‰	47.37	8.55	July, 2017	Active sampling	IRMS	Standard Calibration Method	0.1	10.1002/lom3.10219
$\delta^{13}C$	OC	Biomass Burning-C3	-26.37‰	22.76	114.65	April, 2020	Active sampling	IRMS	Standard Calibration Method	0.2	https://doi.org/10.1016/j.atmosres.2020.104958
$\delta^{13}C$	OC	Biomass Burning-C3	-28.27‰	34.23	108.88	July, 2021	Active sampling	IRMS	Standard Calibration Method	0.19	https://doi.org/10.1016/j.scitotenv.2021.151284
$\delta^{13}C$	OC	Biomass Burning-C3	-28.42‰	34.23	108.88	July, 2021	Active sampling	IRMS	Standard Calibration Method	0.19	https://doi.org/10.1016/j.scitotenv.2021.151284
$\delta^{13}C$	OC	Biomass Burning-C3	-26.84‰	31.87	117.28	July, 2021	Active sampling	IRMS	Standard Calibration Method	0.19	https://doi.org/10.1016/j.scitotenv.2021.151284
$\delta^{13}C$	OC	Biomass Burning-C3	-27.22‰	47.37	8.55	July, 2017	Active sampling	IRMS	Standard Calibration Method	0.1	10.1002/lom3.10219
$\delta^{13}C$	OC	Biomass Burning-C4	-15.38‰	38.03	114.47	July, 2021	Active sampling	IRMS	Standard Calibration Method	0.19	https://doi.org/10.1016/j.scitotenv.2021.151284
$\delta^{13}C$	OC	Biomass Burning-C4	-13.89‰	38.03	114.47	July, 2021	Active sampling	IRMS	Standard Calibration Method	0.19	https://doi.org/10.1016/j.scitotenv.2021.151284
$\delta^{13}C$	OC	Coal Combustion	-28.66‰	22.76	114.65	April, 2020	Active sampling	IRMS	Standard Calibration Method	0.2	https://doi.org/10.1016/j.atmosres.2020.104958
$\delta^{13}C$	OC	Coal Combustion	-23.61‰	37.54	112.33	July, 2020	Active sampling	IRMS	Standard Calibration Method	0.2	https://doi.org/10.1016/j.envpol.2020.115768
$\delta^{13}C$	OC	Coal Combustion	-23.72‰	37.54	112.33	July, 2020	Active sampling	IRMS	Standard Calibration Method	0.2	https://doi.org/10.1016/j.envpol.2020.115768
$\delta^{13}C$	OC	Coal Combustion	-23.49‰	37.54	112.33	July, 2020	Active sampling	IRMS	Standard Calibration Method	0.2	https://doi.org/10.1016/j.envpol.2020.115768

$\delta^{13}C$	OC	Coal Combustion	-22.99‰	37.54	112.33	July, 2020	Active sampling	IRMS	Standard Calibration Method	0.2	https://doi.org/10.1016/j.envpol.2020.115768
$\delta^{13}C$	OC	Coal Combustion	-23.59‰	37.54	112.33	July, 2020	Active sampling	IRMS	Standard Calibration Method	0.2	https://doi.org/10.1016/j.envpol.2020.115768
$\delta^{13}C$	OC	Coal Combustion	-24.92‰	34.23	108.88	July, 2021	Active sampling	IRMS	Standard Calibration Method	0.19	https://doi.org/10.1016/j.scitotenv.2021.151284
$\delta^{13}C$	OC	Coal Combustion	-24.22‰	34.23	108.88	July, 2021	Active sampling	IRMS	Standard Calibration Method	0.19	https://doi.org/10.1016/j.scitotenv.2021.151284
$\delta^{13}C$	OC	Coal Combustion	-24.19‰	38.47	106.26	July, 2021	Active sampling	IRMS	Standard Calibration Method	0.19	https://doi.org/10.1016/j.scitotenv.2021.151284
$\delta^{13}C$	OC	Coal Combustion	-24.94‰	37.54	112.33	July, 2021	Active sampling	IRMS	Standard Calibration Method	0.19	https://doi.org/10.1016/j.scitotenv.2021.151284
$\delta^{13}C$	OC	Coal Combustion	-25.12‰	40.82	111.77	July, 2021	Active sampling	IRMS	Standard Calibration Method	0.19	https://doi.org/10.1016/j.scitotenv.2021.151284
$\delta^{13}C$	OC	Coal Combustion	-26.50‰	37.54	112.33	July, 2012	Active sampling	IRMS	Standard Calibration Method	0.12	Isotopic compositions of carbon in PM10 and its emission sources in Taiyuan. in Chinese
$\delta^{13}C$	OC	Liquid Fossil Fuels	-25.43‰	40.46	-3.75	12 December, 2008	Active sampling	IRMS	Standard Calibration Method	0.2	10.1016/j.atmosenv.2011.01.029
$\delta^{13}C$	OC	Liquid Fossil Fuels	-29.37‰	22.76	114.65	April, 2020	Active sampling	IRMS	Standard Calibration Method	0.2	https://doi.org/10.1016/j.atmosres.2020.104958
$\delta^{13}C$	OC	Liquid Fossil Fuels	-27.83‰	53.22	6.57	July, 2007	Active sampling	IRMS	Standard Calibration Method	0.2	https://doi.org/10.1016/j.jaerosci.2020.105534
$\delta^{13}C$	OC	Liquid Fossil Fuels	-27.70‰	37.54	112.33	July, 2012	Active sampling	IRMS	Standard Calibration Method	0.12	Isotopic compositions of carbon in PM10 and its emission sources in Taiyuan. in Chinese
$\delta^{13}C$	OC	Liquid Fossil Fuels	-25.70‰	37.54	112.33	July, 2012	Active sampling	IRMS	Standard Calibration Method	0.12	Isotopic compositions of carbon in PM10 and its emission sources in Taiyuan. in Chinese

$\delta^{15}N$	NH_3	Biomass Burning	-20.80‰	36.31	120.32	1-19 September, 2020	Active sampling	IRMS	Standard Calibration Method	0.3	https://doi.org/10.1021/acs.est.3c04027
$\delta^{15}N$	NH_3	Biomass Burning	-19.90‰	36.31	120.32	1-19 September, 2020	Active sampling	IRMS	Standard Calibration Method	0.3	https://doi.org/10.1021/acs.est.3c04027
$\delta^{15}N$	NH_3	Biomass Burning	-21.70‰	36.31	120.32	1-19 September, 2020	Active sampling	IRMS	Standard Calibration Method	0.3	https://doi.org/10.1021/acs.est.3c04027
$\delta^{15}N$	NH_3	Biomass Burning	-7.80‰	36.31	120.32	1-19 September, 2020	Active sampling	IRMS	Standard Calibration Method	0.3	https://doi.org/10.1021/acs.est.3c04027
$\delta^{15}N$	NH_3	Biomass Burning	-3.00‰	36.31	120.32	1-19 September, 2020	Active sampling	IRMS	Standard Calibration Method	0.3	https://doi.org/10.1021/acs.est.3c04027
$\delta^{15}N$	NH_3	Biomass Burning	-6.70‰	36.31	120.32	1-19 September, 2020	Active sampling	IRMS	Standard Calibration Method	0.3	https://doi.org/10.1021/acs.est.3c04027
$\delta^{15}N$	NH_3	Biomass Burning	-8.90‰	36.31	120.32	1-19 September, 2020	Active sampling	IRMS	Standard Calibration Method	0.3	https://doi.org/10.1021/acs.est.3c04027
$\delta^{15}N$	NH_3	Biomass Burning	-21.70‰	36.31	120.32	1-19 September, 2020	Active sampling	IRMS	Standard Calibration Method	0.3	https://doi.org/10.1021/acs.est.3c04027
$\delta^{15}N$	NH_3	Biomass Burning	-9.30‰	36.31	120.32	1-19 September, 2020	Active sampling	IRMS	Standard Calibration Method	0.3	https://doi.org/10.1021/acs.est.3c04027
$\delta^{15}N$	NH_3	Biomass Burning	-6.10‰	36.31	120.32	1-19 September, 2020	Active sampling	IRMS	Standard Calibration Method	0.3	https://doi.org/10.1021/acs.est.3c04027
$\delta^{15}N$	NH_3	Biomass Burning	-6.00‰	36.31	120.32	1-19 September, 2020	Active sampling	IRMS	Standard Calibration Method	0.3	https://doi.org/10.1021/acs.est.3c04027
$\delta^{15}N$	NH_3	Biomass Burning	-4.60‰	36.31	120.32	1-19 September, 2020	Active sampling	IRMS	Standard Calibration Method	0.3	https://doi.org/10.1021/acs.est.3c04027
$\delta^{15}N$	NH_3	Biomass Burning	-9.80‰	36.31	120.32	1-19 September, 2020	Active sampling	IRMS	Standard Calibration Method	0.3	https://doi.org/10.1021/acs.est.3c04027
$\delta^{15}N$	NH_3	Biomass Burning	-6.70‰	36.31	120.32	1-19 September, 2020	Active sampling	IRMS	Standard Calibration Method	0.3	https://doi.org/10.1021/acs.est.3c04027
$\delta^{15}N$	NH_3	Biomass Burning	-18.60‰	36.31	120.32	1-19 September, 2020	Active sampling	IRMS	Standard Calibration Method	0.3	https://doi.org/10.1021/acs.est.3c04027

$\delta^{15}N$	NH_3	Biomass Burning	-15.90‰	36.31	120.32	1-19 September, 2020	Active sampling	IRMS	Standard Calibration Method	0.3	https://doi.org/10.1021/acs.est.3c04027
$\delta^{15}N$	NH_3	Biomass Burning	-14.00‰	36.31	120.32	1-19 September, 2020	Active sampling	IRMS	Standard Calibration Method	0.3	https://doi.org/10.1021/acs.est.3c04027
$\delta^{15}N$	NH_3	Biomass Burning	-11.10‰	36.31	120.32	1-19 September, 2020	Active sampling	IRMS	Standard Calibration Method	0.3	https://doi.org/10.1021/acs.est.3c04027
$\delta^{15}N$	NH_3	Biomass Burning	-12.70‰	36.31	120.32	1-19 September, 2020	Active sampling	IRMS	Standard Calibration Method	0.3	https://doi.org/10.1021/acs.est.3c04027
$\delta^{15}N$	NH_3	Biomass Burning	-10.50‰	36.31	120.32	1-19 September, 2020	Active sampling	IRMS	Standard Calibration Method	0.3	https://doi.org/10.1021/acs.est.3c04027
$\delta^{15}N$	NH_3	Biomass Burning	-16.90‰	36.31	120.32	1-19 September, 2020	Active sampling	IRMS	Standard Calibration Method	0.3	https://doi.org/10.1021/acs.est.3c04027
$\delta^{15}N$	NH_3	Biomass Burning	-1.80‰	36.31	120.32	1-19 September, 2020	Active sampling	IRMS	Standard Calibration Method	0.3	https://doi.org/10.1021/acs.est.3c04027
$\delta^{15}N$	NH_3	Coal Combustion	-1.40‰	36.31	120.32	December, 2020	Active sampling	IRMS	Standard Calibration Method	0.3	https://doi.org/10.1021/acs.est.3c04027
$\delta^{15}N$	NH_3	Coal Combustion	-1.50‰	36.31	120.32	December, 2020	Active sampling	IRMS	Standard Calibration Method	0.3	https://doi.org/10.1021/acs.est.3c04027
$\delta^{15}N$	NH_3	Coal Combustion	-1.10‰	36.31	120.32	December, 2020	Active sampling	IRMS	Standard Calibration Method	0.3	https://doi.org/10.1021/acs.est.3c04027
$\delta^{15}N$	NH_3	Coal Combustion	5.20‰	36.31	120.32	December, 2020	Active sampling	IRMS	Standard Calibration Method	0.3	https://doi.org/10.1021/acs.est.3c04027
$\delta^{15}N$	NH_3	Coal Combustion	0.40‰	36.31	120.32	December, 2020	Active sampling	IRMS	Standard Calibration Method	0.3	https://doi.org/10.1021/acs.est.3c04027
$\delta^{15}N$	NH_3	Coal Combustion	-7.70‰	36.31	120.32	December, 2020	Active sampling	IRMS	Standard Calibration Method	0.3	https://doi.org/10.1021/acs.est.3c04027
$\delta^{15}N$	NH_3	Coal Combustion	-4.90‰	36.31	120.32	December, 2020	Active sampling	IRMS	Standard Calibration Method	0.3	https://doi.org/10.1021/acs.est.3c04027
$\delta^{15}N$	NH_3	Coal Combustion	-1.20‰	36.31	120.32	December, 2020	Active sampling	IRMS	Standard Calibration Method	0.3	https://doi.org/10.1021/acs.est.3c04027

$\delta^{15}N$	NH_3	Coal Combustion	-2.40‰	36.31	120.32	December, 2020	Active sampling	IRMS	Standard Calibration Method	0.3	https://doi.org/10.1021/acs.est.3c04027
$\delta^{15}N$	NH_3	Coal Combustion	-6.90‰	36.31	120.32	December, 2020	Active sampling	IRMS	Standard Calibration Method	0.3	https://doi.org/10.1021/acs.est.3c04027
$\delta^{15}N$	NH_3	Coal Combustion	-8.10‰	36.31	120.32	December, 2020	Active sampling	IRMS	Standard Calibration Method	0.3	https://doi.org/10.1021/acs.est.3c04027
$\delta^{15}N$	NH_3	Coal Combustion	-9.50‰	36.31	120.32	December, 2020	Active sampling	IRMS	Standard Calibration Method	0.3	https://doi.org/10.1021/acs.est.3c04027
$\delta^{15}N$	NH_3	Coal Combustion	9.80‰	36.31	120.32	December, 2020	Active sampling	IRMS	Standard Calibration Method	0.3	https://doi.org/10.1021/acs.est.3c04027
$\delta^{15}N$	NH_3	Coal Combustion	12.90‰	36.31	120.32	December, 2020	Active sampling	IRMS	Standard Calibration Method	0.3	https://doi.org/10.1021/acs.est.3c04027
$\delta^{15}N$	NH_3	Coal Combustion	8.70‰	36.31	120.32	December, 2020	Active sampling	IRMS	Standard Calibration Method	0.3	https://doi.org/10.1021/acs.est.3c04027
$\delta^{15}N$	NH_3	Coal Combustion	2.90‰	36.31	120.32	December, 2020	Active sampling	IRMS	Standard Calibration Method	0.3	https://doi.org/10.1021/acs.est.3c04027
$\delta^{15}N$	NH_3	Coal Combustion	8.80‰	36.31	120.32	December, 2020	Active sampling	IRMS	Standard Calibration Method	0.3	https://doi.org/10.1021/acs.est.3c04027
$\delta^{15}N$	NH_3	Coal Combustion	4.20‰	36.31	120.32	December, 2020	Active sampling	IRMS	Standard Calibration Method	0.3	https://doi.org/10.1021/acs.est.3c04027
$\delta^{15}N$	NH_3	Coal Combustion	-9.00‰	36.31	120.32	December, 2020	Active sampling	IRMS	Standard Calibration Method	0.3	https://doi.org/10.1021/acs.est.3c04027
$\delta^{15}N$	NH_3	Coal Combustion	4.00‰	36.31	120.32	December, 2020	Active sampling	IRMS	Standard Calibration Method	0.3	https://doi.org/10.1021/acs.est.3c04027
$\delta^{15}N$	NH_3	Coal Combustion	-21.90‰	36.31	120.32	27 November-6 December, 2020	Active sampling	IRMS	Standard Calibration Method	0.3	https://doi.org/10.1021/acs.est.3c04027
$\delta^{15}N$	NH_3	Coal Combustion	-13.20‰	36.31	120.32	27 November-6 December, 2020	Active sampling	IRMS	Standard Calibration Method	0.3	https://doi.org/10.1021/acs.est.3c04027
$\delta^{15}N$	NH_3	Coal Combustion	-12.30‰	36.31	120.32	27 November-6 December, 2020	Active sampling	IRMS	Standard Calibration Method	0.3	https://doi.org/10.1021/acs.est.3c04027

$\delta^{15}N$	NH_3	Coal Combustion	-20.00‰	36.31	120.32	27 November-6 December, 2020	Active sampling	IRMS	Standard Calibration Method	0.3	https://doi.org/10.1021/acs.est.3c04027
$\delta^{15}N$	NH_3	Coal Combustion	-18.20‰	36.31	120.32	27 November-6 December, 2020	Active sampling	IRMS	Standard Calibration Method	0.3	https://doi.org/10.1021/acs.est.3c04027
$\delta^{15}N$	NH_3	Coal Combustion	-26.20‰	36.31	120.32	27 November-6 December, 2020	Active sampling	IRMS	Standard Calibration Method	0.3	https://doi.org/10.1021/acs.est.3c04027
$\delta^{15}N$	NH_3	Coal Combustion	-15.30‰	36.31	120.32	27 November-6 December, 2020	Active sampling	IRMS	Standard Calibration Method	0.3	https://doi.org/10.1021/acs.est.3c04027
$\delta^{15}N$	NH_3	Coal Combustion	-20.80‰	36.31	120.32	27 November-6 December, 2020	Active sampling	IRMS	Standard Calibration Method	0.3	https://doi.org/10.1021/acs.est.3c04027
$\delta^{15}N$	NH_3	Coal Combustion	-14.90‰	36.31	120.32	27 November-6 December, 2020	Active sampling	IRMS	Standard Calibration Method	0.3	https://doi.org/10.1021/acs.est.3c04027
$\delta^{15}N$	NH_3	Coal Combustion	-17.00‰	36.31	120.32	27 November-6 December, 2020	Active sampling	IRMS	Standard Calibration Method	0.3	https://doi.org/10.1021/acs.est.3c04027
$\delta^{15}N$	NH_3	Coal Combustion	-22.70‰	36.31	120.32	27 November-6 December, 2020	Active sampling	IRMS	Standard Calibration Method	0.3	https://doi.org/10.1021/acs.est.3c04027
$\delta^{15}N$	NH_3	Coal Combustion	-25.90‰	36.31	120.32	27 November-6 December, 2020	Active sampling	IRMS	Standard Calibration Method	0.3	https://doi.org/10.1021/acs.est.3c04027
$\delta^{15}N$	NH_3	Coal Combustion	-15.90‰	36.31	120.32	27 November-6 December, 2020	Active sampling	IRMS	Standard Calibration Method	0.3	https://doi.org/10.1021/acs.est.3c04027
$\delta^{15}N$	NH_3	Coal Combustion	-28.20‰	36.31	120.32	27 November-6 December, 2020	Active sampling	IRMS	Standard Calibration Method	0.3	https://doi.org/10.1021/acs.est.3c04027
$\delta^{15}N$	NH_3	Coal Combustion	-15.30‰	36.31	120.32	27 November-6 December, 2020	Active sampling	IRMS	Standard Calibration Method	0.3	https://doi.org/10.1021/acs.est.3c04027
$\delta^{15}N$	NH_3	Coal Combustion	-27.20‰	36.31	120.32	27 November-6 December, 2020	Active sampling	IRMS	Standard Calibration Method	0.3	https://doi.org/10.1021/acs.est.3c04027
$\delta^{15}N$	NH_3	Coal Combustion	-24.90‰	36.31	120.32	27 November-6 December, 2020	Active sampling	IRMS	Standard Calibration Method	0.3	https://doi.org/10.1021/acs.est.3c04027
$\delta^{15}N$	NH_3	Coal Combustion	-15.90‰	36.31	120.32	27 November-6 December, 2020	Active sampling	IRMS	Standard Calibration Method	0.3	https://doi.org/10.1021/acs.est.3c04027

$\delta^{15}N$	NH_3	Coal Combustion	-20.70‰	36.31	120.32	27 November-6 December, 2020	Active sampling	IRMS	Standard Calibration Method	0.3	https://doi.org/10.1021/acs.est.3c04027
$\delta^{15}N$	NH_3	Coal Combustion	-20.60‰	36.31	120.32	27 November-6 December, 2020	Active sampling	IRMS	Standard Calibration Method	0.3	https://doi.org/10.1021/acs.est.3c04027
$\delta^{15}N$	NH_3	Coal Combustion	-23.50‰	36.31	120.32	27 November-6 December, 2020	Active sampling	IRMS	Standard Calibration Method	0.3	https://doi.org/10.1021/acs.est.3c04027
$\delta^{15}N$	NH_3	Coal Combustion	-17.50‰	36.31	120.32	27 November-6 December, 2020	Active sampling	IRMS	Standard Calibration Method	0.3	https://doi.org/10.1021/acs.est.3c04027
$\delta^{15}N$	NH_3	Coal Combustion	1.70‰	36.31	120.32	27 November-6 December, 2020	Active sampling	IRMS	Standard Calibration Method	0.3	https://doi.org/10.1021/acs.est.3c04027
$\delta^{15}N$	NH_3	Coal Combustion	-1.50‰	36.31	120.32	27 November-6 December, 2020	Active sampling	IRMS	Standard Calibration Method	0.3	https://doi.org/10.1021/acs.est.3c04027
$\delta^{15}N$	NH_3	Coal Combustion	2.40‰	36.31	120.32	27 November-6 December, 2020	Active sampling	IRMS	Standard Calibration Method	0.3	https://doi.org/10.1021/acs.est.3c04027
$\delta^{15}N$	NH_3	Coal Combustion	0.30‰	36.31	120.32	27 November-6 December, 2020	Active sampling	IRMS	Standard Calibration Method	0.3	https://doi.org/10.1021/acs.est.3c04027
$\delta^{15}N$	NH_3	Vehicle Exhausts	-4.40‰	36.26	120.58	2019 and 2020	Active sampling	IRMS	Standard Calibration Method	0.3	https://doi.org/10.1021/acs.est.3c04027
$\delta^{15}N$	NH_3	Vehicle Exhausts	-7.50‰	36.26	120.58	2019 and 2020	Active sampling	IRMS	Standard Calibration Method	0.3	https://doi.org/10.1021/acs.est.3c04027
$\delta^{15}N$	NH_3	Vehicle Exhausts	-5.30‰	36.26	120.58	2019 and 2020	Active sampling	IRMS	Standard Calibration Method	0.3	https://doi.org/10.1021/acs.est.3c04027
$\delta^{15}N$	NH_3	Vehicle Exhausts	-6.50‰	36.26	120.58	2019 and 2020	Active sampling	IRMS	Standard Calibration Method	0.3	https://doi.org/10.1021/acs.est.3c04027
$\delta^{15}N$	NH_3	Vehicle Exhausts	4.40‰	36.26	120.58	2019 and 2020	Active sampling	IRMS	Standard Calibration Method	0.3	https://doi.org/10.1021/acs.est.3c04027
$\delta^{15}N$	NH_3	Vehicle Exhausts	-2.90‰	36.26	120.58	2019 and 2020	Active sampling	IRMS	Standard Calibration Method	0.3	https://doi.org/10.1021/acs.est.3c04027
$\delta^{15}N$	NH_3	Vehicle Exhausts	2.90‰	36.26	120.58	2019 and 2020	Active sampling	IRMS	Standard Calibration Method	0.3	https://doi.org/10.1021/acs.est.3c04027

$\delta^{15}N$	NH_3	Vehicle Exhausts	-13.30‰	36.26	120.58	2019 and 2020	Active sampling	IRMS	Standard Calibration Method	0.3	https://doi.org/10.1021/acs.est.3c04027
$\delta^{15}N$	NH_3	Vehicle Exhausts	-11.70‰	36.26	120.58	2019 and 2020	Active sampling	IRMS	Standard Calibration Method	0.3	https://doi.org/10.1021/acs.est.3c04027
$\delta^{15}N$	NH_3	Vehicle Exhausts	8.10‰	36.26	120.58	2019 and 2020	Active sampling	IRMS	Standard Calibration Method	0.3	https://doi.org/10.1021/acs.est.3c04027
$\delta^{15}N$	NH_3	Vehicle Exhausts	6.20‰	36.26	120.58	2019 and 2020	Active sampling	IRMS	Standard Calibration Method	0.3	https://doi.org/10.1021/acs.est.3c04027
$\delta^{15}N$	NH_3	Vehicle Exhausts	-10.70‰	36.26	120.58	2019 and 2020	Active sampling	IRMS	Standard Calibration Method	0.3	https://doi.org/10.1021/acs.est.3c04027
$\delta^{15}N$	NH_3	Vehicle Exhausts	1.70‰	36.26	120.58	2019 and 2020	Active sampling	IRMS	Standard Calibration Method	0.3	https://doi.org/10.1021/acs.est.3c04027
$\delta^{15}N$	NH_3	Vehicle Exhausts	7.10‰	36.26	120.58	2019 and 2020	Active sampling	IRMS	Standard Calibration Method	0.3	https://doi.org/10.1021/acs.est.3c04027
$\delta^{15}N$	NH_3	Vehicle Exhausts	0.90‰	36.26	120.58	2019 and 2020	Active sampling	IRMS	Standard Calibration Method	0.3	https://doi.org/10.1021/acs.est.3c04027
$\delta^{15}N$	NH_3	Vehicle Exhausts	2.30‰	36.26	120.58	2019 and 2020	Active sampling	IRMS	Standard Calibration Method	0.3	https://doi.org/10.1021/acs.est.3c04027
$\delta^{15}N$	NH_3	Vehicle Exhausts	6.60‰	36.26	120.58	2019 and 2020	Active sampling	IRMS	Standard Calibration Method	0.3	https://doi.org/10.1021/acs.est.3c04027
$\delta^{15}N$	NH_3	Vehicle Exhausts	-12.50‰	36.26	120.58	2019 and 2020	Active sampling	IRMS	Standard Calibration Method	0.3	https://doi.org/10.1021/acs.est.3c04027
$\delta^{15}N$	NH_3	Vehicle Exhausts	-4.00‰	36.26	120.58	2019 and 2020	Active sampling	IRMS	Standard Calibration Method	0.3	https://doi.org/10.1021/acs.est.3c04027
$\delta^{15}N$	NH_3	Vehicle Exhausts	1.80‰	36.26	120.58	2019 and 2020	Active sampling	IRMS	Standard Calibration Method	0.3	https://doi.org/10.1021/acs.est.3c04027
$\delta^{15}N$	NH_3	Vehicle Exhausts	-4.10‰	36.26	120.58	2019 and 2020	Active sampling	IRMS	Standard Calibration Method	0.3	https://doi.org/10.1021/acs.est.3c04027
$\delta^{15}N$	NH_3	Vehicle Exhausts	-13.5‰	40.35	116.02	March-April, 2017	Passive sampling/+15.4‰	PT-IRMS	Standard Calibration Method	0.3	10.3389/fenvs.2022.903013

$\delta^{15}N$	NH_3	Vehicle Exhausts	-15.4‰	40.35	116.02	March-April, 2017	Passive sampling/+15.4‰	PT-IRMS	Standard Calibration Method	0.3	10.3389/fenvs.2022.903013
$\delta^{15}N$	NH_3	Vehicle Exhausts	-15.4‰	40.35	116.02	June-August, 2017	Passive sampling/+15.4‰	PT-IRMS	Standard Calibration Method	0.3	10.3389/fenvs.2022.903013
$\delta^{15}N$	NH_3	Vehicle Exhausts	-9.2‰	40.35	116.02	June-August, 2017	Passive sampling/+15.4‰	PT-IRMS	Standard Calibration Method	0.3	10.3389/fenvs.2022.903013
$\delta^{15}N$	NH_3	Vehicle Exhausts	-4.2‰	40.35	116.02	June-August, 2017	Passive sampling/+15.4‰	PT-IRMS	Standard Calibration Method	0.3	10.3389/fenvs.2022.903013
$\delta^{15}N$	NH_3	Vehicle Exhausts	-10.4‰	40.35	116.02	June-August, 2017	Passive sampling/+15.4‰	PT-IRMS	Standard Calibration Method	0.3	10.3389/fenvs.2022.903013
$\delta^{15}N$	NH_3	Vehicle Exhausts	-12.3‰	40.35	116.02	June-August, 2017	Passive sampling/+15.4‰	PT-IRMS	Standard Calibration Method	0.3	10.3389/fenvs.2022.903013
$\delta^{15}N$	NH_3	Vehicle Exhausts	-19.2‰	40.35	116.02	October, 2017	Passive sampling/+15.4‰	PT-IRMS	Standard Calibration Method	0.3	10.3389/fenvs.2022.903013
$\delta^{15}N$	NH_3	Vehicle Exhausts	-21.3‰	40.35	116.02	October, 2017	Passive sampling/+15.4‰	PT-IRMS	Standard Calibration Method	0.3	10.3389/fenvs.2022.903013
$\delta^{15}N$	NH_3	Vehicle Exhausts	-18.4‰	40.35	116.02	January, 2018	Passive sampling/+15.4‰	PT-IRMS	Standard Calibration Method	0.3	10.3389/fenvs.2022.903013
$\delta^{15}N$	NH_3	Vehicle Exhausts	-10.8‰	40.35	116.02	January, 2018	Passive sampling/+15.4‰	PT-IRMS	Standard Calibration Method	0.3	10.3389/fenvs.2022.903013
$\delta^{15}N$	NH_3	Vehicle Exhausts	-17.3‰	40.35	116.02	January, 2018	Passive sampling/+15.4‰	PT-IRMS	Standard Calibration Method	0.3	10.3389/fenvs.2022.903013
$\delta^{15}N$	NH_3	Vehicle Exhausts	4.85‰	41.8	123.44	30 October, 2018	Active sampling	PT-IRMS	Standard Calibration Method	0.5	https://doi.org/10.1016/j.atmosenv.2021.118430
$\delta^{15}N$	NH_3	Vehicle Exhausts	9.29‰	41.8	123.44	30 October, 2018	Active sampling	PT-IRMS	Standard Calibration Method	0.5	https://doi.org/10.1016/j.atmosenv.2021.118430
$\delta^{15}N$	NH_3	Vehicle Exhausts	0.38‰	41.8	123.44	31 October, 2018	Active sampling	PT-IRMS	Standard Calibration Method	0.5	https://doi.org/10.1016/j.atmosenv.2021.118430
$\delta^{15}N$	NH_3	Vehicle Exhausts	3.88‰	41.8	123.44	31 October, 2018	Active sampling	PT-IRMS	Standard Calibration Method	0.5	https://doi.org/10.1016/j.atmosenv.2021.118430

$\delta^{15}N$	NH_3	Vehicle Exhausts	4.61‰	41.8	123.44	31 October, 2018	Active sampling	PT-IRMS	Standard Calibration Method	0.5	https://doi.org/10.1016/j.atmosenv.2021.118430
$\delta^{15}N$	NH_3	Vehicle Exhausts	-2.01‰	41.8	123.44	1 November, 2018	Active sampling	PT-IRMS	Standard Calibration Method	0.5	https://doi.org/10.1016/j.atmosenv.2021.118430
$\delta^{15}N$	NH_3	Vehicle Exhausts	2.01‰	41.8	123.44	1 November, 2018	Active sampling	PT-IRMS	Standard Calibration Method	0.5	https://doi.org/10.1016/j.atmosenv.2021.118430
$\delta^{15}N$	NH_3	Vehicle Exhausts	4.35‰	41.8	123.44	1 November, 2018	Active sampling	PT-IRMS	Standard Calibration Method	0.5	https://doi.org/10.1016/j.atmosenv.2021.118430
$\delta^{15}N$	NH_3	Vehicle Exhausts	-1.20‰	41.8	123.44	2 November, 2018	Active sampling	PT-IRMS	Standard Calibration Method	0.5	https://doi.org/10.1016/j.atmosenv.2021.118430
$\delta^{15}N$	NH_3	Vehicle Exhausts	3.71‰	41.8	123.44	2 November, 2018	Active sampling	PT-IRMS	Standard Calibration Method	0.5	https://doi.org/10.1016/j.atmosenv.2021.118430
$\delta^{15}N$	NH_3	Vehicle Exhausts	5.13‰	41.8	123.44	2 November, 2018	Active sampling	PT-IRMS	Standard Calibration Method	0.5	https://doi.org/10.1016/j.atmosenv.2021.118430
$\delta^{15}N$	NH_3	Vehicle Exhausts	-1.44‰	41.8	123.44	3 November, 2018	Active sampling	PT-IRMS	Standard Calibration Method	0.5	https://doi.org/10.1016/j.atmosenv.2021.118430
$\delta^{15}N$	NH_3	Vehicle Exhausts	2.24‰	41.8	123.44	3 November, 2018	Active sampling	PT-IRMS	Standard Calibration Method	0.5	https://doi.org/10.1016/j.atmosenv.2021.118430
$\delta^{15}N$	NH_3	Vehicle Exhausts	1.77‰	41.8	123.44	3 November, 2018	Active sampling	PT-IRMS	Standard Calibration Method	0.5	https://doi.org/10.1016/j.atmosenv.2021.118430
$\delta^{15}N$	NH_3	Vehicle Exhausts	0.27‰	41.8	123.44	4 November, 2018	Active sampling	PT-IRMS	Standard Calibration Method	0.5	https://doi.org/10.1016/j.atmosenv.2021.118430
$\delta^{15}N$	NH_3	Vehicle Exhausts	4.97‰	41.8	123.44	4 November, 2018	Active sampling	PT-IRMS	Standard Calibration Method	0.5	https://doi.org/10.1016/j.atmosenv.2021.118430
$\delta^{15}N$	NH_3	Vehicle Exhausts	4.35‰	41.8	123.44	4 November, 2018	Active sampling	PT-IRMS	Standard Calibration Method	0.5	https://doi.org/10.1016/j.atmosenv.2021.118430
$\delta^{15}N$	NH_3	Vehicle Exhausts	-0.95‰	41.8	123.44	5 November, 2018	Active sampling	PT-IRMS	Standard Calibration Method	0.5	https://doi.org/10.1016/j.atmosenv.2021.118430
$\delta^{15}N$	NH_3	Vehicle Exhausts	4.49‰	41.8	123.44	5 November, 2018	Active sampling	PT-IRMS	Standard Calibration Method	0.5	https://doi.org/10.1016/j.atmosenv.2021.118430

$\delta^{15}N$	NH_3	Vehicle Exhausts	4.13‰	41.8	123.44	5 November, 2018	Active sampling	PT-IRMS	Standard Calibration Method	0.5	https://doi.org/10.1016/j.atmosenv.2021.118430
$\delta^{15}N$	NH_3	Vehicle Exhausts	0.00‰	41.8	123.44	6 November, 2018	Active sampling	PT-IRMS	Standard Calibration Method	0.5	https://doi.org/10.1016/j.atmosenv.2021.118430
$\delta^{15}N$	NH_3	Vehicle Exhausts	6.80‰	41.8	123.44	30 October, 2018	Active sampling	PT-IRMS	Standard Calibration Method	0.5	https://doi.org/10.1016/j.atmosenv.2021.118430
$\delta^{15}N$	NH_3	Vehicle Exhausts	8.16‰	41.8	123.44	30 October, 2018	Active sampling	PT-IRMS	Standard Calibration Method	0.5	https://doi.org/10.1016/j.atmosenv.2021.118430
$\delta^{15}N$	NH_3	Vehicle Exhausts	3.97‰	41.8	123.44	31 October, 2018	Active sampling	PT-IRMS	Standard Calibration Method	0.5	https://doi.org/10.1016/j.atmosenv.2021.118430
$\delta^{15}N$	NH_3	Vehicle Exhausts	7.26‰	41.8	123.44	31 October, 2018	Active sampling	PT-IRMS	Standard Calibration Method	0.5	https://doi.org/10.1016/j.atmosenv.2021.118430
$\delta^{15}N$	NH_3	Vehicle Exhausts	7.92‰	41.8	123.44	31 October, 2018	Active sampling	PT-IRMS	Standard Calibration Method	0.5	https://doi.org/10.1016/j.atmosenv.2021.118430
$\delta^{15}N$	NH_3	Vehicle Exhausts	3.09‰	41.8	123.44	1 November, 2018	Active sampling	PT-IRMS	Standard Calibration Method	0.5	https://doi.org/10.1016/j.atmosenv.2021.118430
$\delta^{15}N$	NH_3	Vehicle Exhausts	6.28‰	41.8	123.44	1 November, 2018	Active sampling	PT-IRMS	Standard Calibration Method	0.5	https://doi.org/10.1016/j.atmosenv.2021.118430
$\delta^{15}N$	NH_3	Vehicle Exhausts	6.65‰	41.8	123.44	1 November, 2018	Active sampling	PT-IRMS	Standard Calibration Method	0.5	https://doi.org/10.1016/j.atmosenv.2021.118430
$\delta^{15}N$	NH_3	Vehicle Exhausts	1.91‰	41.8	123.44	2 November, 2018	Active sampling	PT-IRMS	Standard Calibration Method	0.5	https://doi.org/10.1016/j.atmosenv.2021.118430
$\delta^{15}N$	NH_3	Vehicle Exhausts	4.61‰	41.8	123.44	2 November, 2018	Active sampling	PT-IRMS	Standard Calibration Method	0.5	https://doi.org/10.1016/j.atmosenv.2021.118430
$\delta^{15}N$	NH_3	Vehicle Exhausts	3.81‰	41.8	123.44	2 November, 2018	Active sampling	PT-IRMS	Standard Calibration Method	0.5	https://doi.org/10.1016/j.atmosenv.2021.118430
$\delta^{15}N$	NH_3	Vehicle Exhausts	0.95‰	41.8	123.44	3 November, 2018	Active sampling	PT-IRMS	Standard Calibration Method	0.5	https://doi.org/10.1016/j.atmosenv.2021.118430
$\delta^{15}N$	NH_3	Vehicle Exhausts	5.09‰	41.8	123.44	3 November, 2018	Active sampling	PT-IRMS	Standard Calibration Method	0.5	https://doi.org/10.1016/j.atmosenv.2021.118430

$\delta^{15}N$	NH_3	Vehicle Exhausts	4.78‰	41.8	123.44	3 November, 2018	Active sampling	PT-IRMS	Standard Calibration Method	0.5	https://doi.org/10.1016/j.atmosenv.2021.118430
$\delta^{15}N$	NH_3	Vehicle Exhausts	2.65‰	41.8	123.44	4 November, 2018	Active sampling	PT-IRMS	Standard Calibration Method	0.5	https://doi.org/10.1016/j.atmosenv.2021.118430
$\delta^{15}N$	NH_3	Vehicle Exhausts	6.08‰	41.8	123.44	4 November, 2018	Active sampling	PT-IRMS	Standard Calibration Method	0.5	https://doi.org/10.1016/j.atmosenv.2021.118430
$\delta^{15}N$	NH_3	Vehicle Exhausts	6.04‰	41.8	123.44	4 November, 2018	Active sampling	PT-IRMS	Standard Calibration Method	0.5	https://doi.org/10.1016/j.atmosenv.2021.118430
$\delta^{15}N$	NH_3	Vehicle Exhausts	2.21‰	41.8	123.44	5 November, 2018	Active sampling	PT-IRMS	Standard Calibration Method	0.5	https://doi.org/10.1016/j.atmosenv.2021.118430
$\delta^{15}N$	NH_3	Vehicle Exhausts	5.30‰	41.8	123.44	5 November, 2018	Active sampling	PT-IRMS	Standard Calibration Method	0.5	https://doi.org/10.1016/j.atmosenv.2021.118430
$\delta^{15}N$	NH_3	Vehicle Exhausts	6.54‰	41.8	123.44	5 November, 2018	Active sampling	PT-IRMS	Standard Calibration Method	0.5	https://doi.org/10.1016/j.atmosenv.2021.118430
$\delta^{15}N$	NH_3	Vehicle Exhausts	1.93‰	41.8	123.44	6 November, 2018	Active sampling	PT-IRMS	Standard Calibration Method	0.5	https://doi.org/10.1016/j.atmosenv.2021.118430
$\delta^{15}N$	NH_3	Vehicle Exhausts	4.32‰	41.8	123.45	30 October-5 November, 2018	Active sampling	CF-IRMS	Standard Calibration Method	0.69	https://doi.org/10.5194/acp-20-11551-2020
$\delta^{15}N$	NH_3	Vehicle Exhausts	8.98‰	41.8	123.45	30 October-5 November, 2018	Active sampling	CF-IRMS	Standard Calibration Method	0.69	https://doi.org/10.5194/acp-20-11551-2020
$\delta^{15}N$	NH_3	Vehicle Exhausts	0.02‰	41.8	123.45	30 October-5 November, 2018	Active sampling	CF-IRMS	Standard Calibration Method	0.69	https://doi.org/10.5194/acp-20-11551-2020
$\delta^{15}N$	NH_3	Vehicle Exhausts	3.46‰	41.8	123.45	30 October-5 November, 2018	Active sampling	CF-IRMS	Standard Calibration Method	0.69	https://doi.org/10.5194/acp-20-11551-2020
$\delta^{15}N$	NH_3	Vehicle Exhausts	4.22‰	41.8	123.45	30 October-5 November, 2018	Active sampling	CF-IRMS	Standard Calibration Method	0.69	https://doi.org/10.5194/acp-20-11551-2020
$\delta^{15}N$	NH_3	Vehicle Exhausts	-1.97‰	41.8	123.45	30 October-5 November, 2018	Active sampling	CF-IRMS	Standard Calibration Method	0.69	https://doi.org/10.5194/acp-20-11551-2020
$\delta^{15}N$	NH_3	Vehicle Exhausts	2.03‰	41.8	123.45	30 October-5 November, 2018	Active sampling	CF-IRMS	Standard Calibration Method	0.69	https://doi.org/10.5194/acp-20-11551-2020

$\delta^{15}N$	NH_3	Vehicle Exhausts	3.92‰	41.8	123.45	30 October-5 November, 2018	Active sampling	CF-IRMS	Standard Calibration Method	0.69	https://doi.org/10.5194/acp-20-11551-2020
$\delta^{15}N$	NH_3	Vehicle Exhausts	-0.77‰	41.8	123.45	30 October-5 November, 2018	Active sampling	CF-IRMS	Standard Calibration Method	0.69	https://doi.org/10.5194/acp-20-11551-2020
$\delta^{15}N$	NH_3	Vehicle Exhausts	3.28‰	41.8	123.45	30 October-5 November, 2018	Active sampling	CF-IRMS	Standard Calibration Method	0.69	https://doi.org/10.5194/acp-20-11551-2020
$\delta^{15}N$	NH_3	Vehicle Exhausts	5.40‰	41.8	123.45	30 October-5 November, 2018	Active sampling	CF-IRMS	Standard Calibration Method	0.69	https://doi.org/10.5194/acp-20-11551-2020
$\delta^{15}N$	NH_3	Vehicle Exhausts	0.06‰	41.8	123.45	30 October-5 November, 2018	Active sampling	CF-IRMS	Standard Calibration Method	0.69	https://doi.org/10.5194/acp-20-11551-2020
$\delta^{15}N$	NH_3	Vehicle Exhausts	1.86‰	41.8	123.45	30 October-5 November, 2018	Active sampling	CF-IRMS	Standard Calibration Method	0.69	https://doi.org/10.5194/acp-20-11551-2020
$\delta^{15}N$	NH_3	Vehicle Exhausts	2.15‰	41.8	123.45	30 October-5 November, 2018	Active sampling	CF-IRMS	Standard Calibration Method	0.69	https://doi.org/10.5194/acp-20-11551-2020
$\delta^{15}N$	NH_3	Vehicle Exhausts	2.62‰	41.8	123.45	30 October-5 November, 2018	Active sampling	CF-IRMS	Standard Calibration Method	0.69	https://doi.org/10.5194/acp-20-11551-2020
$\delta^{15}N$	NH_3	Vehicle Exhausts	4.75‰	41.8	123.45	30 October-5 November, 2018	Active sampling	CF-IRMS	Standard Calibration Method	0.69	https://doi.org/10.5194/acp-20-11551-2020
$\delta^{15}N$	NH_3	Vehicle Exhausts	4.05‰	41.8	123.45	30 October-5 November, 2018	Active sampling	CF-IRMS	Standard Calibration Method	0.69	https://doi.org/10.5194/acp-20-11551-2020
$\delta^{15}N$	NH_3	Vehicle Exhausts	-1.15‰	41.8	123.45	30 October-5 November, 2018	Active sampling	CF-IRMS	Standard Calibration Method	0.69	https://doi.org/10.5194/acp-20-11551-2020
$\delta^{15}N$	NH_3	Vehicle Exhausts	4.21‰	41.8	123.45	30 October-5 November, 2018	Active sampling	CF-IRMS	Standard Calibration Method	0.69	https://doi.org/10.5194/acp-20-11551-2020
$\delta^{15}N$	NH_3	Vehicle Exhausts	3.85‰	41.8	123.45	30 October-5 November, 2018	Active sampling	CF-IRMS	Standard Calibration Method	0.69	https://doi.org/10.5194/acp-20-11551-2020
$\delta^{15}N$	NH_3	Vehicle Exhausts	-0.32‰	41.8	123.45	30 October-5 November, 2018	Active sampling	CF-IRMS	Standard Calibration Method	0.69	https://doi.org/10.5194/acp-20-11551-2020
$\delta^{15}N$	NH_3	Vehicle Exhausts	-9.6‰	31.28	121.51	July, 2014	Passive sampling/+15.4‰	PT-IRMS	Standard Calibration Method	0.3	https://doi.org/10.5194/acp-16-11635-2016

$\delta^{15}N$	NH_3	Vehicle Exhausts	-12.2‰	31.28	121.51	August, 2014	Passive sampling/+15.4‰	PT-IRMS	Standard Calibration Method	0.3	https://doi.org/10.5194/acp-16-11635-2016
$\delta^{15}N$	NH_3	Vehicle Exhausts	-16.7‰	31.28	121.51	December, 2014	Passive sampling/+15.4‰	PT-IRMS	Standard Calibration Method	0.3	https://doi.org/10.5194/acp-16-11635-2016
$\delta^{15}N$	NH_3	Vehicle Exhausts	-17.8‰	31.28	121.51	January, 2015	Passive sampling/+15.4‰	PT-IRMS	Standard Calibration Method	0.3	https://doi.org/10.5194/acp-16-11635-2016
$\delta^{15}N$	NH_3	Vehicle Exhausts	-11.9‰	31.28	121.51	July, 2014	Passive sampling/+15.4‰	PT-IRMS	Standard Calibration Method	0.3	https://doi.org/10.5194/acp-16-11635-2016
$\delta^{15}N$	NH_3	Vehicle Exhausts	-14.1‰	31.28	121.51	August, 2014	Passive sampling/+15.4‰	PT-IRMS	Standard Calibration Method	0.3	https://doi.org/10.5194/acp-16-11635-2016
$\delta^{15}N$	NH_3	Vehicle Exhausts	-16.4‰	31.28	121.51	December, 2014	Passive sampling/+15.4‰	PT-IRMS	Standard Calibration Method	0.3	https://doi.org/10.5194/acp-16-11635-2016
$\delta^{15}N$	NH_3	Vehicle Exhausts	-15.1‰	31.28	121.51	January, 2015	Passive sampling/+15.4‰	PT-IRMS	Standard Calibration Method	0.3	https://doi.org/10.5194/acp-16-11635-2016
$\delta^{15}N$	NH_3	Volatilized Fertilizer	-27.1‰	40.13	116.2	March-April, 2017	Passive sampling/+15.4‰	PT-IRMS	Standard Calibration Method	0.3	10.3389/fenvs.2022.903013
$\delta^{15}N$	NH_3	Volatilized Fertilizer	-39.9‰	40.13	116.2	March-April, 2017	Passive sampling/+15.4‰	PT-IRMS	Standard Calibration Method	0.3	10.3389/fenvs.2022.903013
$\delta^{15}N$	NH_3	Volatilized Fertilizer	-28.7‰	40.13	116.2	March-April, 2017	Passive sampling/+15.4‰	PT-IRMS	Standard Calibration Method	0.3	10.3389/fenvs.2022.903013
$\delta^{15}N$	NH_3	Volatilized Fertilizer	-34.6‰	40.13	116.2	March-April, 2017	Passive sampling/+15.4‰	PT-IRMS	Standard Calibration Method	0.3	10.3389/fenvs.2022.903013
$\delta^{15}N$	NH_3	Volatilized Fertilizer	-37.1‰	40.13	116.2	March-April, 2017	Passive sampling/+15.4‰	PT-IRMS	Standard Calibration Method	0.3	10.3389/fenvs.2022.903013
$\delta^{15}N$	NH_3	Volatilized Fertilizer	-31.5‰	40.13	116.2	March-April, 2017	Passive sampling/+15.4‰	PT-IRMS	Standard Calibration Method	0.3	10.3389/fenvs.2022.903013
$\delta^{15}N$	NH_3	Volatilized Fertilizer	-37.9‰	40.13	116.2	June-August, 2017	Passive sampling/+15.4‰	PT-IRMS	Standard Calibration Method	0.3	10.3389/fenvs.2022.903013
$\delta^{15}N$	NH_3	Volatilized Fertilizer	-37.1‰	40.13	116.2	June-August, 2017	Passive sampling/+15.4‰	PT-IRMS	Standard Calibration Method	0.3	10.3389/fenvs.2022.903013

$\delta^{15}N$	NH_3	Volatilized Fertilizer	-31.6‰	40.13	116.2	June-August, 2017	Passive sampling/+15.4‰	PT-IRMS	Standard Calibration Method	0.3	10.3389/fenvs.2022.903013
$\delta^{15}N$	NH_3	Volatilized Fertilizer	-30.5‰	40.13	116.2	June-August, 2017	Passive sampling/+15.4‰	PT-IRMS	Standard Calibration Method	0.3	10.3389/fenvs.2022.903013
$\delta^{15}N$	NH_3	Volatilized Fertilizer	-38.6‰	40.13	116.2	June-August, 2017	Passive sampling/+15.4‰	PT-IRMS	Standard Calibration Method	0.3	10.3389/fenvs.2022.903013
$\delta^{15}N$	NH_3	Volatilized Fertilizer	-33‰	40.13	116.2	June-August, 2017	Passive sampling/+15.4‰	PT-IRMS	Standard Calibration Method	0.3	10.3389/fenvs.2022.903013
$\delta^{15}N$	NH_3	Volatilized Fertilizer	-37.1‰	40.13	116.2	June-August, 2017	Passive sampling/+15.4‰	PT-IRMS	Standard Calibration Method	0.3	10.3389/fenvs.2022.903013
$\delta^{15}N$	NH_3	Volatilized Fertilizer	-37.9‰	40.13	116.2	June-August, 2017	Passive sampling/+15.4‰	PT-IRMS	Standard Calibration Method	0.3	10.3389/fenvs.2022.903013
$\delta^{15}N$	NH_3	Volatilized Fertilizer	-35.3‰	40.13	116.2	June-August, 2017	Passive sampling/+15.4‰	PT-IRMS	Standard Calibration Method	0.3	10.3389/fenvs.2022.903013
$\delta^{15}N$	NH_3	Volatilized Fertilizer	-37.2‰	40.13	116.2	June-August, 2017	Passive sampling/+15.4‰	PT-IRMS	Standard Calibration Method	0.3	10.3389/fenvs.2022.903013
$\delta^{15}N$	NH_3	Volatilized Fertilizer	-39.5‰	40.13	116.2	October, 2017	Passive sampling/+15.4‰	PT-IRMS	Standard Calibration Method	0.3	10.3389/fenvs.2022.903013
$\delta^{15}N$	NH_3	Volatilized Fertilizer	-10.74‰	31.26	120.2	November, 2013-March, 2015	Active sampling	IRMS	Standard Calibration Method	0.3	https://doi.org/10.1007/s10533-018-0432-3
$\delta^{15}N$	NH_3	Volatilized Fertilizer	-2.37‰	31.56	120.71	November, 2018	Passive sampling/+15.4‰	IRMS	Standard Calibration Method	0.3	https://doi.org/10.1016/j.envpol.2020.116204
$\delta^{15}N$	NH_3	Volatilized Fertilizer	-12.54‰	31.56	120.71	November, 2018	Passive sampling/+15.4‰	IRMS	Standard Calibration Method	0.3	https://doi.org/10.1016/j.envpol.2020.116204
$\delta^{15}N$	NH_3	Volatilized Fertilizer	-19.07‰	31.56	120.71	November, 2018	Passive sampling/+15.4‰	IRMS	Standard Calibration Method	0.3	https://doi.org/10.1016/j.envpol.2020.116204
$\delta^{15}N$	NH_3	Volatilized Fertilizer	-24.97‰	31.56	120.71	November, 2018	Passive sampling/+15.4‰	IRMS	Standard Calibration Method	0.3	https://doi.org/10.1016/j.envpol.2020.116204
$\delta^{15}N$	NH_3	Volatilized Fertilizer	-22.55‰	31.56	120.71	November, 2018	Passive sampling/+15.4‰	IRMS	Standard Calibration Method	0.3	https://doi.org/10.1016/j.envpol.2020.116204

$\delta^{15}N$	NH_3	Volatilized Fertilizer	-20.77‰	31.56	120.71	November, 2018	Passive sampling/+15.4‰	IRMS	Standard Calibration Method	0.3	https://doi.org/10.1016/j.envpol.2020.116204
$\delta^{15}N$	NH_3	Volatilized Fertilizer	-16.01‰	31.56	120.71	November, 2018	Passive sampling/+15.4‰	IRMS	Standard Calibration Method	0.3	https://doi.org/10.1016/j.envpol.2020.116204
$\delta^{15}N$	NH_3	Volatilized Fertilizer	-11.09‰	31.56	120.71	November, 2018	Passive sampling/+15.4‰	IRMS	Standard Calibration Method	0.3	https://doi.org/10.1016/j.envpol.2020.116204
$\delta^{15}N$	NH_3	Volatilized Fertilizer	-18.35‰	31.56	120.71	November, 2018	Passive sampling/+15.4‰	IRMS	Standard Calibration Method	0.3	https://doi.org/10.1016/j.envpol.2020.116204
$\delta^{15}N$	NH_3	Volatilized Fertilizer	-21.02‰	31.56	120.71	November, 2018	Passive sampling/+15.4‰	IRMS	Standard Calibration Method	0.3	https://doi.org/10.1016/j.envpol.2020.116204
$\delta^{15}N$	NH_3	Volatilized Fertilizer	-30.46‰	31.56	120.71	November, 2018	Passive sampling/+15.4‰	IRMS	Standard Calibration Method	0.3	https://doi.org/10.1016/j.envpol.2020.116204
$\delta^{15}N$	NH_3	Volatilized Fertilizer	-32.72‰	31.56	120.71	November, 2018	Passive sampling/+15.4‰	IRMS	Standard Calibration Method	0.3	https://doi.org/10.1016/j.envpol.2020.116204
$\delta^{15}N$	NH_3	Volatilized Fertilizer	-29.74‰	31.56	120.71	November, 2018	Passive sampling/+15.4‰	IRMS	Standard Calibration Method	0.3	https://doi.org/10.1016/j.envpol.2020.116204
$\delta^{15}N$	NH_3	Volatilized Fertilizer	-29.49‰	31.56	120.71	November, 2018	Passive sampling/+15.4‰	IRMS	Standard Calibration Method	0.3	https://doi.org/10.1016/j.envpol.2020.116204
$\delta^{15}N$	NH_3	Volatilized Fertilizer	-25.06‰	31.56	120.71	November, 2018	Passive sampling/+15.4‰	IRMS	Standard Calibration Method	0.3	https://doi.org/10.1016/j.envpol.2020.116204
$\delta^{15}N$	NH_3	Volatilized Fertilizer	-20.69‰	31.56	120.71	November, 2018	Passive sampling/+15.4‰	IRMS	Standard Calibration Method	0.3	https://doi.org/10.1016/j.envpol.2020.116204
$\delta^{15}N$	NH_3	Volatilized Fertilizer	-34.34‰	31.56	120.71	November, 2018	Passive sampling/+15.4‰	IRMS	Standard Calibration Method	0.3	https://doi.org/10.1016/j.envpol.2020.116204
$\delta^{15}N$	NH_3	Volatilized Fertilizer	-36.52‰	31.56	120.71	November, 2018	Passive sampling/+15.4‰	IRMS	Standard Calibration Method	0.3	https://doi.org/10.1016/j.envpol.2020.116204
$\delta^{15}N$	NH_3	Volatilized Fertilizer	-39.83‰	31.56	120.71	November, 2018	Passive sampling/+15.4‰	IRMS	Standard Calibration Method	0.3	https://doi.org/10.1016/j.envpol.2020.116204
$\delta^{15}N$	NH_3	Volatilized Fertilizer	-40.54‰	31.56	120.71	November, 2018	Passive sampling/+15.4‰	IRMS	Standard Calibration Method	0.3	https://doi.org/10.1016/j.envpol.2020.116204

$\delta^{15}N$	NH_3	Volatilized Fertilizer	-37.73‰	31.56	120.71	November, 2018	Passive sampling/+15.4‰	IRMS	Standard Calibration Method	0.3	https://doi.org/10.1016/j.envpol.2020.116204
$\delta^{15}N$	NH_3	Volatilized Fertilizer	-33.05‰	31.56	120.71	November, 2018	Passive sampling/+15.4‰	IRMS	Standard Calibration Method	0.3	https://doi.org/10.1016/j.envpol.2020.116204
$\delta^{15}N$	NH_3	Volatilized Fertilizer	-28.85‰	31.56	120.71	November, 2018	Passive sampling/+15.4‰	IRMS	Standard Calibration Method	0.3	https://doi.org/10.1016/j.envpol.2020.116204
$\delta^{15}N$	NH_3	Volatilized Fertilizer	-31.40‰	36.39	120.58	October, 2019	Active sampling	IRMS	Standard Calibration Method	0.3	https://doi.org/10.1021/acs.est.3c04027
$\delta^{15}N$	NH_3	Volatilized Fertilizer	-27.00‰	36.39	120.58	August, 2020	Active sampling	IRMS	Standard Calibration Method	0.3	https://doi.org/10.1021/acs.est.3c04027
$\delta^{15}N$	NH_3	Volatilized Fertilizer	-23.20‰	36.39	120.58	March, 2021	Active sampling	IRMS	Standard Calibration Method	0.3	https://doi.org/10.1021/acs.est.3c04027
$\delta^{15}N$	NH_3	Volatilized Fertilizer	-29.80‰	36.39	120.58	November, 2019	Active sampling	IRMS	Standard Calibration Method	0.3	https://doi.org/10.1021/acs.est.3c04027
$\delta^{15}N$	NH_3	Volatilized Fertilizer	-39.70‰	36.39	120.58	July, 2020	Active sampling	IRMS	Standard Calibration Method	0.3	https://doi.org/10.1021/acs.est.3c04027
$\delta^{15}N$	NH_3	Volatilized Fertilizer	-29.60‰	36.39	120.58	June, 2020	Active sampling	IRMS	Standard Calibration Method	0.3	https://doi.org/10.1021/acs.est.3c04027
$\delta^{15}N$	NH_3	Volatilized Fertilizer	-32.10‰	36.39	120.58	March, 2021	Active sampling	IRMS	Standard Calibration Method	0.3	https://doi.org/10.1021/acs.est.3c04027
$\delta^{15}N$	NH_3	Volatilized Fertilizer	-25.70‰	36.39	120.58	July, 2021	Active sampling	IRMS	Standard Calibration Method	0.3	https://doi.org/10.1021/acs.est.3c04027
$\delta^{15}N$	NH_3	Volatilized Fertilizer	-22.60‰	36.39	120.58	June, 2021	Active sampling	IRMS	Standard Calibration Method	0.3	https://doi.org/10.1021/acs.est.3c04027
$\delta^{15}N$	NH_3	Volatilized Fertilizer	-32.00‰	36.39	120.58	October, 2019	Active sampling	IRMS	Standard Calibration Method	0.3	https://doi.org/10.1021/acs.est.3c04027
$\delta^{15}N$	NH_3	Volatilized Fertilizer	-25.40‰	36.39	120.58	August, 2020	Active sampling	IRMS	Standard Calibration Method	0.3	https://doi.org/10.1021/acs.est.3c04027
$\delta^{15}N$	NH_3	Volatilized Fertilizer	-29.10‰	36.39	120.58	March, 2021	Active sampling	IRMS	Standard Calibration Method	0.3	https://doi.org/10.1021/acs.est.3c04027

$\delta^{15}N$	NH_3	Volatilized Fertilizer	-32.20‰	36.39	120.58	November, 2019	Active sampling	IRMS	Standard Calibration Method	0.3	https://doi.org/10.1021/acs.est.3c04027
$\delta^{15}N$	NH_3	Volatilized Fertilizer	-32.90‰	36.39	120.58	July, 2020	Active sampling	IRMS	Standard Calibration Method	0.3	https://doi.org/10.1021/acs.est.3c04027
$\delta^{15}N$	NH_3	Volatilized Fertilizer	-31.10‰	36.39	120.58	June, 2020	Active sampling	IRMS	Standard Calibration Method	0.3	https://doi.org/10.1021/acs.est.3c04027
$\delta^{15}N$	NH_3	Volatilized Fertilizer	-22.60‰	36.39	120.58	March, 2021	Active sampling	IRMS	Standard Calibration Method	0.3	https://doi.org/10.1021/acs.est.3c04027
$\delta^{15}N$	NH_3	Volatilized Fertilizer	-29.30‰	36.39	120.58	July, 2021	Active sampling	IRMS	Standard Calibration Method	0.3	https://doi.org/10.1021/acs.est.3c04027
$\delta^{15}N$	NH_3	Volatilized Fertilizer	-20.30‰	36.39	120.58	June, 2021	Active sampling	IRMS	Standard Calibration Method	0.3	https://doi.org/10.1021/acs.est.3c04027
$\delta^{15}N$	NH_3	Volatilized Fertilizer	-21.40‰	36.39	120.58	October, 2019	Active sampling	IRMS	Standard Calibration Method	0.3	https://doi.org/10.1021/acs.est.3c04027
$\delta^{15}N$	NH_3	Volatilized Fertilizer	-27.20‰	36.39	120.58	August, 2020	Active sampling	IRMS	Standard Calibration Method	0.3	https://doi.org/10.1021/acs.est.3c04027
$\delta^{15}N$	NH_3	Volatilized Fertilizer	-21.80‰	36.39	120.58	March, 2021	Active sampling	IRMS	Standard Calibration Method	0.3	https://doi.org/10.1021/acs.est.3c04027
$\delta^{15}N$	NH_3	Volatilized Fertilizer	-22.00‰	36.39	120.58	November, 2019	Active sampling	IRMS	Standard Calibration Method	0.3	https://doi.org/10.1021/acs.est.3c04027
$\delta^{15}N$	NH_3	Volatilized Fertilizer	-20.90‰	36.39	120.58	July, 2020	Active sampling	IRMS	Standard Calibration Method	0.3	https://doi.org/10.1021/acs.est.3c04027
$\delta^{15}N$	NH_3	Volatilized Fertilizer	-19.50‰	36.39	120.58	June, 2020	Active sampling	IRMS	Standard Calibration Method	0.3	https://doi.org/10.1021/acs.est.3c04027
$\delta^{15}N$	NH_3	Volatilized Fertilizer	-19.90‰	36.39	120.58	March, 2021	Active sampling	IRMS	Standard Calibration Method	0.3	https://doi.org/10.1021/acs.est.3c04027
$\delta^{15}N$	NH_3	Volatilized Fertilizer	-22.60‰	36.39	120.58	July, 2021	Active sampling	IRMS	Standard Calibration Method	0.3	https://doi.org/10.1021/acs.est.3c04027
$\delta^{15}N$	NH_3	Volatilized Fertilizer	-20.80‰	36.39	120.58	June, 2021	Active sampling	IRMS	Standard Calibration Method	0.3	https://doi.org/10.1021/acs.est.3c04027

$\delta^{15}N$	NH_3	Volatilized Fertilizer	-25.30‰	36.39	120.58	October, 2019	Active sampling	IRMS	Standard Calibration Method	0.3	https://doi.org/10.1021/acs.est.3c04027
$\delta^{15}N$	NH_3	Volatilized Fertilizer	-31.90‰	36.39	120.58	August, 2020	Active sampling	IRMS	Standard Calibration Method	0.3	https://doi.org/10.1021/acs.est.3c04027
$\delta^{15}N$	NH_3	Volatilized Fertilizer	-21.90‰	36.39	120.58	March, 2021	Active sampling	IRMS	Standard Calibration Method	0.3	https://doi.org/10.1021/acs.est.3c04027
$\delta^{15}N$	NH_3	Volatilized Fertilizer	-21.40‰	36.39	120.58	November, 2019	Active sampling	IRMS	Standard Calibration Method	0.3	https://doi.org/10.1021/acs.est.3c04027
$\delta^{15}N$	NH_3	Volatilized Fertilizer	-25.30‰	36.39	120.58	July, 2020	Active sampling	IRMS	Standard Calibration Method	0.3	https://doi.org/10.1021/acs.est.3c04027
$\delta^{15}N$	NH_3	Volatilized Fertilizer	-24.80‰	36.39	120.58	June, 2020	Active sampling	IRMS	Standard Calibration Method	0.3	https://doi.org/10.1021/acs.est.3c04027
$\delta^{15}N$	NH_3	Volatilized Fertilizer	-21.20‰	36.39	120.58	March, 2021	Active sampling	IRMS	Standard Calibration Method	0.3	https://doi.org/10.1021/acs.est.3c04027
$\delta^{15}N$	NH_3	Volatilized Fertilizer	-21.00‰	36.39	120.58	July, 2021	Active sampling	IRMS	Standard Calibration Method	0.3	https://doi.org/10.1021/acs.est.3c04027
$\delta^{15}N$	NH_3	Volatilized Fertilizer	-26.20‰	37.01	120.28	June, 2021	Active sampling	IRMS	Standard Calibration Method	0.3	https://doi.org/10.1021/acs.est.3c04027
$\delta^{15}N$	NH_3	Volatilized Fertilizer	-35.00‰	37.01	120.28	October, 2019	Active sampling	IRMS	Standard Calibration Method	0.3	https://doi.org/10.1021/acs.est.3c04027
$\delta^{15}N$	NH_3	Volatilized Fertilizer	-28.90‰	37.01	120.28	August, 2020	Active sampling	IRMS	Standard Calibration Method	0.3	https://doi.org/10.1021/acs.est.3c04027
$\delta^{15}N$	NH_3	Volatilized Fertilizer	-23.40‰	37.01	120.28	March, 2021	Active sampling	IRMS	Standard Calibration Method	0.3	https://doi.org/10.1021/acs.est.3c04027
$\delta^{15}N$	NH_3	Volatilized Fertilizer	-20.50‰	37.01	120.28	November, 2019	Active sampling	IRMS	Standard Calibration Method	0.3	https://doi.org/10.1021/acs.est.3c04027
$\delta^{15}N$	NH_3	Volatilized Fertilizer	-23.70‰	37.01	120.28	July, 2020	Active sampling	IRMS	Standard Calibration Method	0.3	https://doi.org/10.1021/acs.est.3c04027
$\delta^{15}N$	NH_3	Volatilized Fertilizer	-28.00‰	37.01	120.28	June, 2020	Active sampling	IRMS	Standard Calibration Method	0.3	https://doi.org/10.1021/acs.est.3c04027

$\delta^{15}N$	NH_3	Volatilized Fertilizer	-31.00‰	37.01	120.28	March, 2021	Active sampling	IRMS	Standard Calibration Method	0.3	https://doi.org/10.1021/acs.est.3c04027
$\delta^{15}N$	NH_3	Volatilized Fertilizer	-23.40‰	37.01	120.28	July, 2021	Active sampling	IRMS	Standard Calibration Method	0.3	https://doi.org/10.1021/acs.est.3c04027
$\delta^{15}N$	NH_3	Volatilized Fertilizer	-29.20‰	37.01	120.28	June, 2021	Active sampling	IRMS	Standard Calibration Method	0.3	https://doi.org/10.1021/acs.est.3c04027
$\delta^{15}N$	NH_3	Volatilized Fertilizer	-33.00‰	37.01	120.28	October, 2019	Active sampling	IRMS	Standard Calibration Method	0.3	https://doi.org/10.1021/acs.est.3c04027
$\delta^{15}N$	NH_3	Volatilized Fertilizer	-24.20‰	37.01	120.28	August, 2020	Active sampling	IRMS	Standard Calibration Method	0.3	https://doi.org/10.1021/acs.est.3c04027
$\delta^{15}N$	NH_3	Volatilized Fertilizer	-27.10‰	37.01	120.28	March, 2021	Active sampling	IRMS	Standard Calibration Method	0.3	https://doi.org/10.1021/acs.est.3c04027
$\delta^{15}N$	NH_3	Volatilized Fertilizer	-22.70‰	37.01	120.28	November, 2019	Active sampling	IRMS	Standard Calibration Method	0.3	https://doi.org/10.1021/acs.est.3c04027
$\delta^{15}N$	NH_3	Volatilized Fertilizer	-36.30‰	37.01	120.28	July, 2020	Active sampling	IRMS	Standard Calibration Method	0.3	https://doi.org/10.1021/acs.est.3c04027
$\delta^{15}N$	NH_3	Volatilized Fertilizer	-31.40‰	37.01	120.28	June, 2020	Active sampling	IRMS	Standard Calibration Method	0.3	https://doi.org/10.1021/acs.est.3c04027
$\delta^{15}N$	NH_3	Volatilized Fertilizer	-17.50‰	37.01	120.28	March, 2021	Active sampling	IRMS	Standard Calibration Method	0.3	https://doi.org/10.1021/acs.est.3c04027
$\delta^{15}N$	NH_3	Volatilized Fertilizer	-21.30‰	37.01	120.28	July, 2021	Active sampling	IRMS	Standard Calibration Method	0.3	https://doi.org/10.1021/acs.est.3c04027
$\delta^{15}N$	NH_3	Volatilized Fertilizer	-27.40‰	37.01	120.28	June, 2021	Active sampling	IRMS	Standard Calibration Method	0.3	https://doi.org/10.1021/acs.est.3c04027
$\delta^{15}N$	NH_3	Volatilized Fertilizer	-31.70‰	37.01	120.28	July, 2021	Active sampling	IRMS	Standard Calibration Method	0.3	https://doi.org/10.1021/acs.est.3c04027
$\delta^{15}N$	NH_3	Volatilized Fertilizer	-14.80‰	37.01	120.28	June, 2021	Active sampling	IRMS	Standard Calibration Method	0.3	https://doi.org/10.1021/acs.est.3c04027
$\delta^{15}N$	NH_3	Volatilized Fertilizer	-26.30‰	37.01	120.28	2020 and 2023	Active sampling	IRMS	Standard Calibration Method	0.3	https://doi.org/10.1021/acs.est.3c04027

$\delta^{15}N$	NH_3	Volatilized Fertilizer	-22.40‰	37.01	120.28	2020 and 2023	Active sampling	IRMS	Standard Calibration Method	0.3	https://doi.org/10.1021/acs.est.3c04027
$\delta^{15}N$	NH_3	Volatilized Fertilizer	-35.30‰	37.01	120.28	2020 and 2023	Active sampling	IRMS	Standard Calibration Method	0.3	https://doi.org/10.1021/acs.est.3c04027
$\delta^{15}N$	NH_3	Volatilized Fertilizer	-24.90‰	37.01	120.28	2020 and 2023	Active sampling	IRMS	Standard Calibration Method	0.3	https://doi.org/10.1021/acs.est.3c04027
$\delta^{15}N$	NH_3	Volatilized Fertilizer	-17.80‰	37.01	120.28	2020 and 2023	Active sampling	IRMS	Standard Calibration Method	0.3	https://doi.org/10.1021/acs.est.3c04027
$\delta^{15}N$	NH_3	Volatilized Fertilizer	-21.30‰	37.01	120.28	2020 and 2023	Active sampling	IRMS	Standard Calibration Method	0.3	https://doi.org/10.1021/acs.est.3c04027
$\delta^{15}N$	NH_3	Volatilized Fertilizer	-25.90‰	37.01	120.28	2020 and 2023	Active sampling	IRMS	Standard Calibration Method	0.3	https://doi.org/10.1021/acs.est.3c04027
$\delta^{15}N$	NH_3	Volatilized Fertilizer	-25.80‰	37.01	120.28	2020 and 2023	Active sampling	IRMS	Standard Calibration Method	0.3	https://doi.org/10.1021/acs.est.3c04027
$\delta^{15}N$	NH_3	Volatilized Fertilizer	-25.80‰	37.01	120.28	2020 and 2023	Active sampling	IRMS	Standard Calibration Method	0.3	https://doi.org/10.1021/acs.est.3c04027
$\delta^{15}N$	NH_3	Volatilized Fertilizer	-31.00‰	37.01	120.28	2020 and 2023	Active sampling	IRMS	Standard Calibration Method	0.3	https://doi.org/10.1021/acs.est.3c04027
$\delta^{15}N$	NH_3	Volatilized Fertilizer	-16.70‰	37.01	120.28	2020 and 2023	Active sampling	IRMS	Standard Calibration Method	0.3	https://doi.org/10.1021/acs.est.3c04027
$\delta^{15}N$	NH_3	Volatilized Fertilizer	-17.50‰	37.01	120.28	2020 and 2023	Active sampling	IRMS	Standard Calibration Method	0.3	https://doi.org/10.1021/acs.est.3c04027
$\delta^{15}N$	NH_3	Volatilized Fertilizer	-26.10‰	37.01	120.28	2020 and 2023	Active sampling	IRMS	Standard Calibration Method	0.3	https://doi.org/10.1021/acs.est.3c04027
$\delta^{15}N$	NH_3	Volatilized Fertilizer	-27.80‰	37.01	120.28	2020 and 2023	Active sampling	IRMS	Standard Calibration Method	0.3	https://doi.org/10.1021/acs.est.3c04027
$\delta^{15}N$	NH_3	Volatilized Fertilizer	-27.60‰	37.01	120.28	2020 and 2023	Active sampling	IRMS	Standard Calibration Method	0.3	https://doi.org/10.1021/acs.est.3c04027
$\delta^{15}N$	NH_3	Volatilized Fertilizer	-27.40‰	37.01	120.28	2020 and 2023	Active sampling	IRMS	Standard Calibration Method	0.3	https://doi.org/10.1021/acs.est.3c04027

$\delta^{15}N$	NH_3	Volatilized Fertilizer	-24.00‰	37.01	120.28	2020 and 2023	Active sampling	IRMS	Standard Calibration Method	0.3	https://doi.org/10.1021/acs.est.3c04027
$\delta^{15}N$	NH_3	Volatilized Fertilizer	-35.80‰	37.01	120.28	2020 and 2023	Active sampling	IRMS	Standard Calibration Method	0.3	https://doi.org/10.1021/acs.est.3c04027
$\delta^{15}N$	NH_3	Volatilized Fertilizer	-31.70‰	37.01	120.28	2020 and 2023	Active sampling	IRMS	Standard Calibration Method	0.3	https://doi.org/10.1021/acs.est.3c04027
$\delta^{15}N$	NH_3	Volatilized Fertilizer	-28.30‰	37.01	120.28	2020 and 2023	Active sampling	IRMS	Standard Calibration Method	0.3	https://doi.org/10.1021/acs.est.3c04027
$\delta^{15}N$	NH_3	Volatilized Fertilizer	-27.31‰	31.56	120.71	November, 2018	Passive sampling/+15.4‰	IRMS	Standard Calibration Method	0.3	https://doi.org/10.1016/j.envpol.2020.116204
$\delta^{15}N$	NH_3	Volatilized Fertilizer	-44.02‰	31.56	120.71	November, 2018	Passive sampling/+15.4‰	IRMS	Standard Calibration Method	0.3	https://doi.org/10.1016/j.envpol.2020.116204
$\delta^{15}N$	NH_3	Volatilized Fertilizer	-45.96‰	31.56	120.71	November, 2018	Passive sampling/+15.4‰	IRMS	Standard Calibration Method	0.3	https://doi.org/10.1016/j.envpol.2020.116204
$\delta^{15}N$	NH_3	Volatilized Fertilizer	-44.43‰	31.56	120.71	November, 2018	Passive sampling/+15.4‰	IRMS	Standard Calibration Method	0.3	https://doi.org/10.1016/j.envpol.2020.116204
$\delta^{15}N$	NH_3	Volatilized Fertilizer	-46.04‰	31.56	120.71	November, 2018	Passive sampling/+15.4‰	IRMS	Standard Calibration Method	0.3	https://doi.org/10.1016/j.envpol.2020.116204
$\delta^{15}N$	NH_3	Volatilized Fertilizer	-41.6‰	31.56	120.71	November, 2018	Passive sampling/+15.4‰	IRMS	Standard Calibration Method	0.3	https://doi.org/10.1016/j.envpol.2020.116204
$\delta^{15}N$	NH_3	Volatilized Fertilizer	-37.49‰	31.56	120.71	November, 2018	Passive sampling/+15.4‰	IRMS	Standard Calibration Method	0.3	https://doi.org/10.1016/j.envpol.2020.116204
$\delta^{15}N$	NH_3	Volatilized Fertilizer	-32.96‰	31.56	120.71	November, 2018	Passive sampling/+15.4‰	IRMS	Standard Calibration Method	0.3	https://doi.org/10.1016/j.envpol.2020.116204
$\delta^{15}N$	NH_3	Volatilized Fertilizer	-32.24‰	31.56	120.71	November, 2018	Passive sampling/+15.4‰	IRMS	Standard Calibration Method	0.3	https://doi.org/10.1016/j.envpol.2020.116204
$\delta^{15}N$	NH_3	Volatilized Fertilizer	-52‰	31.28	121.5	10 May, 2014	Passive sampling/+15.4‰	PT-IRMS	Standard Calibration Method	0.3	https://doi.org/10.5194/acp-16-11635-2016
$\delta^{15}N$	NH_3	Volatilized Fertilizer	-51.4‰	31.28	121.5	10 May, 2014	Passive sampling/+15.4‰	PT-IRMS	Standard Calibration Method	0.3	https://doi.org/10.5194/acp-16-11635-2016

$\delta^{15}N$	NH_3	Volatilized Fertilizer	-50.2‰	31.28	121.5	10 May, 2014	Passive sampling/+15.4‰	PT-IRMS	Standard Calibration Method	0.3	https://doi.org/10.5194/acp-16-11635-2016
$\delta^{15}N$	NH_3	Volatilized Fertilizer	-47.6‰	31.28	121.5	10 May, 2014	Passive sampling/+15.4‰	PT-IRMS	Standard Calibration Method	0.3	https://doi.org/10.5194/acp-16-11635-2016
$\delta^{15}N$	NH_3	Volatilized Fertilizer	-48.7‰	31.28	121.5	10-11 May, 2014	Passive sampling/+15.4‰	PT-IRMS	Standard Calibration Method	0.3	https://doi.org/10.5194/acp-16-11635-2016
$\delta^{15}N$	NH_3	Volatilized Fertilizer	-51.51‰	39.55	115.84	28 May-18 June, 2019	Passive sampling/+15.4‰	CF-IRMS	Standard Calibration Method	0.03	https://doi.org/10.1016/j.scitotenv.2020.141361
$\delta^{15}N$	NH_3	Volatilized Fertilizer	-46.43‰	39.55	115.84	28 May-18 June, 2019	Passive sampling/+15.4‰	CF-IRMS	Standard Calibration Method	0.03	https://doi.org/10.1016/j.scitotenv.2020.141361
$\delta^{15}N$	NH_3	Volatilized Fertilizer	-43.07‰	39.55	115.84	28 May-18 June, 2019	Passive sampling/+15.4‰	CF-IRMS	Standard Calibration Method	0.03	https://doi.org/10.1016/j.scitotenv.2020.141361
$\delta^{15}N$	NH_3	Volatilized Fertilizer	-40.88‰	39.55	115.84	28 May-18 June, 2019	Passive sampling/+15.4‰	CF-IRMS	Standard Calibration Method	0.03	https://doi.org/10.1016/j.scitotenv.2020.141361
$\delta^{15}N$	NH_3	Volatilized Fertilizer	-39.62‰	39.55	115.84	28 May-18 June, 2019	Passive sampling/+15.4‰	CF-IRMS	Standard Calibration Method	0.03	https://doi.org/10.1016/j.scitotenv.2020.141361
$\delta^{15}N$	NH_3	Volatilized Fertilizer	-38.95‰	39.55	115.84	28 May-18 June, 2019	Passive sampling/+15.4‰	CF-IRMS	Standard Calibration Method	0.03	https://doi.org/10.1016/j.scitotenv.2020.141361
$\delta^{15}N$	NH_3	Volatilized Fertilizer	-36.68‰	39.55	115.84	28 May-18 June, 2019	Passive sampling/+15.4‰	CF-IRMS	Standard Calibration Method	0.03	https://doi.org/10.1016/j.scitotenv.2020.141361
$\delta^{15}N$	NH_3	Volatilized Fertilizer	-36.26‰	39.55	115.84	28 May-18 June, 2019	Passive sampling/+15.4‰	CF-IRMS	Standard Calibration Method	0.03	https://doi.org/10.1016/j.scitotenv.2020.141361
$\delta^{15}N$	NH_3	Volatilized Fertilizer	-35.97‰	39.55	115.84	28 May-18 June, 2019	Passive sampling/+15.4‰	CF-IRMS	Standard Calibration Method	0.03	https://doi.org/10.1016/j.scitotenv.2020.141361
$\delta^{15}N$	NH_3	Volatilized Fertilizer	-34.96‰	39.55	115.84	28 May-18 June, 2019	Passive sampling/+15.4‰	CF-IRMS	Standard Calibration Method	0.03	https://doi.org/10.1016/j.scitotenv.2020.141361
$\delta^{15}N$	NH_3	Livestock Emissions	-38.3‰	41	116.58	March-April, 2017	Passive sampling/+15.4‰	PT-IRMS	Standard Calibration Method	0.3	10.3389/fenvs.2022.903013
$\delta^{15}N$	NH_3	Livestock Emissions	-40.8‰	41	116.58	March-April, 2017	Passive sampling/+15.4‰	PT-IRMS	Standard Calibration Method	0.3	10.3389/fenvs.2022.903013

$\delta^{15}N$	NH_3	Livestock Emissions	-41.6‰	41	116.58	March-April, 2017	Passive sampling/+15.4‰	PT-IRMS	Standard Calibration Method	0.3	10.3389/fenvs.2022.903013
$\delta^{15}N$	NH_3	Livestock Emissions	-40.6‰	41	116.58	March-April, 2017	Passive sampling/+15.4‰	PT-IRMS	Standard Calibration Method	0.3	10.3389/fenvs.2022.903013
$\delta^{15}N$	NH_3	Livestock Emissions	-39.2‰	41	116.58	March-April, 2017	Passive sampling/+15.4‰	PT-IRMS	Standard Calibration Method	0.3	10.3389/fenvs.2022.903013
$\delta^{15}N$	NH_3	Livestock Emissions	-34.4‰	41	116.58	March-April, 2017	Passive sampling/+15.4‰	PT-IRMS	Standard Calibration Method	0.3	10.3389/fenvs.2022.903013
$\delta^{15}N$	NH_3	Livestock Emissions	-35.1‰	41	116.58	March-April, 2017	Passive sampling/+15.4‰	PT-IRMS	Standard Calibration Method	0.3	10.3389/fenvs.2022.903013
$\delta^{15}N$	NH_3	Livestock Emissions	-35.1‰	41	116.58	March-April, 2017	Passive sampling/+15.4‰	PT-IRMS	Standard Calibration Method	0.3	10.3389/fenvs.2022.903013
$\delta^{15}N$	NH_3	Livestock Emissions	-34.2‰	41	116.58	March-April, 2017	Passive sampling/+15.4‰	PT-IRMS	Standard Calibration Method	0.3	10.3389/fenvs.2022.903013
$\delta^{15}N$	NH_3	Livestock Emissions	-33‰	41	116.58	March-April, 2017	Passive sampling/+15.4‰	PT-IRMS	Standard Calibration Method	0.3	10.3389/fenvs.2022.903013
$\delta^{15}N$	NH_3	Livestock Emissions	-38.8‰	41	116.58	March-April, 2017	Passive sampling/+15.4‰	PT-IRMS	Standard Calibration Method	0.3	10.3389/fenvs.2022.903013
$\delta^{15}N$	NH_3	Livestock Emissions	-41.1‰	41	116.58	March-April, 2017	Passive sampling/+15.4‰	PT-IRMS	Standard Calibration Method	0.3	10.3389/fenvs.2022.903013
$\delta^{15}N$	NH_3	Livestock Emissions	-37.8‰	41	116.58	March-April, 2017	Passive sampling/+15.4‰	PT-IRMS	Standard Calibration Method	0.3	10.3389/fenvs.2022.903013
$\delta^{15}N$	NH_3	Livestock Emissions	-39.9‰	41	116.58	March-April, 2017	Passive sampling/+15.4‰	PT-IRMS	Standard Calibration Method	0.3	10.3389/fenvs.2022.903013
$\delta^{15}N$	NH_3	Livestock Emissions	-35.2‰	41	116.58	March-April, 2017	Passive sampling/+15.4‰	PT-IRMS	Standard Calibration Method	0.3	10.3389/fenvs.2022.903013
$\delta^{15}N$	NH_3	Livestock Emissions	-37.8‰	41	116.58	March-April, 2017	Passive sampling/+15.4‰	PT-IRMS	Standard Calibration Method	0.3	10.3389/fenvs.2022.903013
$\delta^{15}N$	NH_3	Livestock Emissions	-38.5‰	41	116.58	March-April, 2017	Passive sampling/+15.4‰	PT-IRMS	Standard Calibration Method	0.3	10.3389/fenvs.2022.903013

$\delta^{15}N$	NH_3	Livestock Emissions	-35.4‰	41	116.58	March-April, 2017	Passive sampling/+15.4‰	PT-IRMS	Standard Calibration Method	0.3	10.3389/fenvs.2022.903013
$\delta^{15}N$	NH_3	Livestock Emissions	-38.8‰	41	116.58	March-April, 2017	Passive sampling/+15.4‰	PT-IRMS	Standard Calibration Method	0.3	10.3389/fenvs.2022.903013
$\delta^{15}N$	NH_3	Livestock Emissions	-34.1‰	41	116.58	March-April, 2017	Passive sampling/+15.4‰	PT-IRMS	Standard Calibration Method	0.3	10.3389/fenvs.2022.903013
$\delta^{15}N$	NH_3	Livestock Emissions	-27.4‰	41	116.58	June-August, 2017	Passive sampling/+15.4‰	PT-IRMS	Standard Calibration Method	0.3	10.3389/fenvs.2022.903013
$\delta^{15}N$	NH_3	Livestock Emissions	-30.6‰	41	116.58	June-August, 2017	Passive sampling/+15.4‰	PT-IRMS	Standard Calibration Method	0.3	10.3389/fenvs.2022.903013
$\delta^{15}N$	NH_3	Livestock Emissions	-30.2‰	41	116.58	June-August, 2017	Passive sampling/+15.4‰	PT-IRMS	Standard Calibration Method	0.3	10.3389/fenvs.2022.903013
$\delta^{15}N$	NH_3	Livestock Emissions	-30.5‰	41	116.58	June-August, 2017	Passive sampling/+15.4‰	PT-IRMS	Standard Calibration Method	0.3	10.3389/fenvs.2022.903013
$\delta^{15}N$	NH_3	Livestock Emissions	-30.5‰	41	116.58	June-August, 2017	Passive sampling/+15.4‰	PT-IRMS	Standard Calibration Method	0.3	10.3389/fenvs.2022.903013
$\delta^{15}N$	NH_3	Livestock Emissions	-25.7‰	41	116.58	June-August, 2017	Passive sampling/+15.4‰	PT-IRMS	Standard Calibration Method	0.3	10.3389/fenvs.2022.903013
$\delta^{15}N$	NH_3	Livestock Emissions	-25.7‰	41	116.58	June-August, 2017	Passive sampling/+15.4‰	PT-IRMS	Standard Calibration Method	0.3	10.3389/fenvs.2022.903013
$\delta^{15}N$	NH_3	Livestock Emissions	-33.8‰	41	116.58	October, 2017	Passive sampling/+15.4‰	PT-IRMS	Standard Calibration Method	0.3	10.3389/fenvs.2022.903013
$\delta^{15}N$	NH_3	Livestock Emissions	-35.9‰	41	116.58	October, 2017	Passive sampling/+15.4‰	PT-IRMS	Standard Calibration Method	0.3	10.3389/fenvs.2022.903013
$\delta^{15}N$	NH_3	Livestock Emissions	-31.9‰	41	116.58	October, 2017	Passive sampling/+15.4‰	PT-IRMS	Standard Calibration Method	0.3	10.3389/fenvs.2022.903013
$\delta^{15}N$	NH_3	Livestock Emissions	-35.4‰	41	116.58	January, 2018	Passive sampling/+15.4‰	PT-IRMS	Standard Calibration Method	0.3	10.3389/fenvs.2022.903013
$\delta^{15}N$	NH_3	Livestock Emissions	-38.4‰	41	116.58	January, 2018	Passive sampling/+15.4‰	PT-IRMS	Standard Calibration Method	0.3	10.3389/fenvs.2022.903013

$\delta^{15}N$	NH_3	Livestock Emissions	-29.7‰	41	116.58	January, 2018	Passive sampling/+15.4‰	PT-IRMS	Standard Calibration Method	0.3	10.3389/fenvs.2022.903013
$\delta^{15}N$	NH_3	Livestock Emissions	-32.3‰	41	116.58	January, 2018	Passive sampling/+15.4‰	PT-IRMS	Standard Calibration Method	0.3	10.3389/fenvs.2022.903013
$\delta^{15}N$	NH_3	Livestock Emissions	-27.2‰	31.25	121.39	9 December, 2014	Passive sampling/+15.4‰	PT-IRMS	Standard Calibration Method	0.3	https://doi.org/10.5194/acp-16-11635-2016
$\delta^{15}N$	NH_3	Livestock Emissions	-28.2‰	31.25	121.39	10 December, 2014	Passive sampling/+15.4‰	PT-IRMS	Standard Calibration Method	0.3	https://doi.org/10.5194/acp-16-11635-2016
$\delta^{15}N$	NH_3	Livestock Emissions	-29.3‰	31.25	121.39	3 June, 2014	Passive sampling/+15.4‰	PT-IRMS	Standard Calibration Method	0.3	https://doi.org/10.5194/acp-16-11635-2016
$\delta^{15}N$	NH_3	Livestock Emissions	-31.7‰	31.25	121.39	4 June, 2014	Passive sampling/+15.4‰	PT-IRMS	Standard Calibration Method	0.3	https://doi.org/10.5194/acp-16-11635-2016
$\delta^{15}N$	NH_3	Livestock Emissions	-27.1‰	31.25	121.39	9 December, 2014	Passive sampling/+15.4‰	PT-IRMS	Standard Calibration Method	0.3	https://doi.org/10.5194/acp-16-11635-2016
$\delta^{15}N$	NH_3	Livestock Emissions	-30.3‰	31.25	121.39	10 December, 2014	Passive sampling/+15.4‰	PT-IRMS	Standard Calibration Method	0.3	https://doi.org/10.5194/acp-16-11635-2016
$\delta^{15}N$	NH_3	Livestock Emissions	-29.8‰	31.25	121.39	4 June, 2014	Passive sampling/+15.4‰	PT-IRMS	Standard Calibration Method	0.3	https://doi.org/10.5194/acp-16-11635-2016
$\delta^{15}N$	NH_3	Livestock Emissions	-42.48‰	37.95	114.72	2-4 April, 2018	Passive sampling/+15.4‰	CF-IRMS	Standard Calibration Method	0.03	https://doi.org/10.1016/j.scitotenv.2020.141361
$\delta^{15}N$	NH_3	Livestock Emissions	-42.31‰	37.95	114.72	2-4 April, 2018	Passive sampling/+15.4‰	CF-IRMS	Standard Calibration Method	0.03	https://doi.org/10.1016/j.scitotenv.2020.141361
$\delta^{15}N$	NH_3	Livestock Emissions	-39.54‰	37.95	114.72	2-4 April, 2018	Passive sampling/+15.4‰	CF-IRMS	Standard Calibration Method	0.03	https://doi.org/10.1016/j.scitotenv.2020.141361
$\delta^{15}N$	NH_3	Livestock Emissions	-38.49‰	37.95	114.72	2-4 April, 2018	Passive sampling/+15.4‰	CF-IRMS	Standard Calibration Method	0.03	https://doi.org/10.1016/j.scitotenv.2020.141361
$\delta^{15}N$	NH_3	Livestock Emissions	-37.61‰	37.95	114.72	2-4 April, 2018	Passive sampling/+15.4‰	CF-IRMS	Standard Calibration Method	0.03	https://doi.org/10.1016/j.scitotenv.2020.141361
$\delta^{15}N$	NH_3	Livestock Emissions	-36.81‰	37.95	114.72	2-4 April, 2018	Passive sampling/+15.4‰	CF-IRMS	Standard Calibration Method	0.03	https://doi.org/10.1016/j.scitotenv.2020.141361

$\delta^{15}N$	NH_3	Livestock Emissions	-30.17‰	37.95	114.72	2-4 April, 2018	Passive sampling/+15.4‰	CF-IRMS	Standard Calibration Method	0.03	https://doi.org/10.1016/j.scitotenv.2020.141361
$\delta^{15}N$	NH_3	Livestock Emissions	-29.16‰	37.95	114.72	2-4 April, 2018	Passive sampling/+15.4‰	CF-IRMS	Standard Calibration Method	0.03	https://doi.org/10.1016/j.scitotenv.2020.141361
$\delta^{15}N$	NH_3	Waste Materials	-26.2‰	40.05	116.22	March-April, 2017	Passive sampling/+15.4‰	PT-IRMS	Standard Calibration Method	0.3	10.3389/fenvs.2022.903013
$\delta^{15}N$	NH_3	Waste Materials	-31.1‰	40.05	116.22	March-April, 2017	Passive sampling/+15.4‰	PT-IRMS	Standard Calibration Method	0.3	10.3389/fenvs.2022.903013
$\delta^{15}N$	NH_3	Waste Materials	-35.8‰	40.05	116.22	March-April, 2017	Passive sampling/+15.4‰	PT-IRMS	Standard Calibration Method	0.3	10.3389/fenvs.2022.903013
$\delta^{15}N$	NH_3	Waste Materials	-28.9‰	40.05	116.22	March-April, 2017	Passive sampling/+15.4‰	PT-IRMS	Standard Calibration Method	0.3	10.3389/fenvs.2022.903013
$\delta^{15}N$	NH_3	Waste Materials	-34.6‰	40.05	116.22	March-April, 2017	Passive sampling/+15.4‰	PT-IRMS	Standard Calibration Method	0.3	10.3389/fenvs.2022.903013
$\delta^{15}N$	NH_3	Waste Materials	-29.1‰	40.05	116.22	March-April, 2017	Passive sampling/+15.4‰	PT-IRMS	Standard Calibration Method	0.3	10.3389/fenvs.2022.903013
$\delta^{15}N$	NH_3	Waste Materials	-35.7‰	40.05	116.22	March-April, 2017	Passive sampling/+15.4‰	PT-IRMS	Standard Calibration Method	0.3	10.3389/fenvs.2022.903013
$\delta^{15}N$	NH_3	Waste Materials	-30.5‰	40.05	116.22	March-April, 2017	Passive sampling/+15.4‰	PT-IRMS	Standard Calibration Method	0.3	10.3389/fenvs.2022.903013
$\delta^{15}N$	NH_3	Waste Materials	-25.6‰	40.05	116.22	March-April, 2017	Passive sampling/+15.4‰	PT-IRMS	Standard Calibration Method	0.3	10.3389/fenvs.2022.903013
$\delta^{15}N$	NH_3	Waste Materials	-30.1‰	40.05	116.22	March-April, 2017	Passive sampling/+15.4‰	PT-IRMS	Standard Calibration Method	0.3	10.3389/fenvs.2022.903013
$\delta^{15}N$	NH_3	Waste Materials	-29.7‰	40.05	116.22	March-April, 2017	Passive sampling/+15.4‰	PT-IRMS	Standard Calibration Method	0.3	10.3389/fenvs.2022.903013
$\delta^{15}N$	NH_3	Waste Materials	-32.4‰	40.05	116.22	March-April, 2017	Passive sampling/+15.4‰	PT-IRMS	Standard Calibration Method	0.3	10.3389/fenvs.2022.903013
$\delta^{15}N$	NH_3	Waste Materials	-32.6‰	40.05	116.22	March-April, 2017	Passive sampling/+15.4‰	PT-IRMS	Standard Calibration Method	0.3	10.3389/fenvs.2022.903013

$\delta^{15}N$	NH_3	Waste Materials	-32‰	40.05	116.22	March-April, 2017	Passive sampling/+15.4‰	PT-IRMS	Standard Calibration Method	0.3	10.3389/fenvs.2022.903013
$\delta^{15}N$	NH_3	Waste Materials	-34.4‰	40.05	116.22	June-August, 2017	Passive sampling/+15.4‰	PT-IRMS	Standard Calibration Method	0.3	10.3389/fenvs.2022.903013
$\delta^{15}N$	NH_3	Waste Materials	-25.6‰	40.05	116.22	June-August, 2017	Passive sampling/+15.4‰	PT-IRMS	Standard Calibration Method	0.3	10.3389/fenvs.2022.903013
$\delta^{15}N$	NH_3	Waste Materials	-36.5‰	40.05	116.22	June-August, 2017	Passive sampling/+15.4‰	PT-IRMS	Standard Calibration Method	0.3	10.3389/fenvs.2022.903013
$\delta^{15}N$	NH_3	Waste Materials	-31.8‰	40.05	116.22	June-August, 2017	Passive sampling/+15.4‰	PT-IRMS	Standard Calibration Method	0.3	10.3389/fenvs.2022.903013
$\delta^{15}N$	NH_3	Waste Materials	-32.4‰	40.05	116.22	June-August, 2017	Passive sampling/+15.4‰	PT-IRMS	Standard Calibration Method	0.3	10.3389/fenvs.2022.903013
$\delta^{15}N$	NH_3	Waste Materials	-30.4‰	40.05	116.22	June-August, 2017	Passive sampling/+15.4‰	PT-IRMS	Standard Calibration Method	0.3	10.3389/fenvs.2022.903013
$\delta^{15}N$	NH_3	Waste Materials	-29.8‰	40.05	116.22	June-August, 2017	Passive sampling/+15.4‰	PT-IRMS	Standard Calibration Method	0.3	10.3389/fenvs.2022.903013
$\delta^{15}N$	NH_3	Waste Materials	-30.8‰	40.05	116.22	June-August, 2017	Passive sampling/+15.4‰	PT-IRMS	Standard Calibration Method	0.3	10.3389/fenvs.2022.903013
$\delta^{15}N$	NH_3	Waste Materials	-36.3‰	40.05	116.22	October, 2017	Passive sampling/+15.4‰	PT-IRMS	Standard Calibration Method	0.3	10.3389/fenvs.2022.903013
$\delta^{15}N$	NH_3	Waste Materials	-38.8‰	40.05	116.22	October, 2017	Passive sampling/+15.4‰	PT-IRMS	Standard Calibration Method	0.3	10.3389/fenvs.2022.903013
$\delta^{15}N$	NH_3	Waste Materials	-34.4‰	40.05	116.22	October, 2017	Passive sampling/+15.4‰	PT-IRMS	Standard Calibration Method	0.3	10.3389/fenvs.2022.903013
$\delta^{15}N$	NH_3	Waste Materials	-42.3‰	40.05	116.22	October, 2017	Passive sampling/+15.4‰	PT-IRMS	Standard Calibration Method	0.3	10.3389/fenvs.2022.903013
$\delta^{15}N$	NH_3	Waste Materials	-35.5‰	40.05	116.22	October, 2017	Passive sampling/+15.4‰	PT-IRMS	Standard Calibration Method	0.3	10.3389/fenvs.2022.903013
$\delta^{15}N$	NH_3	Waste Materials	-41.7‰	40.05	116.22	October, 2017	Passive sampling/+15.4‰	PT-IRMS	Standard Calibration Method	0.3	10.3389/fenvs.2022.903013

$\delta^{15}N$	NH_3	Waste Materials	-37.6‰	40.05	116.22	October, 2017	Passive sampling/+15.4‰	PT-IRMS	Standard Calibration Method	0.3	10.3389/fenvs.2022.903013
$\delta^{15}N$	NH_3	Waste Materials	-42.7‰	40.05	116.22	October, 2017	Passive sampling/+15.4‰	PT-IRMS	Standard Calibration Method	0.3	10.3389/fenvs.2022.903013
$\delta^{15}N$	NH_3	Waste Materials	-32‰	40.05	116.22	October, 2017	Passive sampling/+15.4‰	PT-IRMS	Standard Calibration Method	0.3	10.3389/fenvs.2022.903013
$\delta^{15}N$	NH_3	Waste Materials	-32.2‰	40.05	116.22	October, 2017	Passive sampling/+15.4‰	PT-IRMS	Standard Calibration Method	0.3	10.3389/fenvs.2022.903013
$\delta^{15}N$	NH_3	Waste Materials	-29.1‰	40.05	116.22	October, 2017	Passive sampling/+15.4‰	PT-IRMS	Standard Calibration Method	0.3	10.3389/fenvs.2022.903013
$\delta^{15}N$	NH_3	Waste Materials	-35.9‰	40.05	116.22	October, 2017	Passive sampling/+15.4‰	PT-IRMS	Standard Calibration Method	0.3	10.3389/fenvs.2022.903013
$\delta^{15}N$	NH_3	Waste Materials	-40.8‰	40.05	116.22	January, 2018	Passive sampling/+15.4‰	PT-IRMS	Standard Calibration Method	0.3	10.3389/fenvs.2022.903013
$\delta^{15}N$	NH_3	Waste Materials	-33.3‰	40.05	116.22	January, 2018	Passive sampling/+15.4‰	PT-IRMS	Standard Calibration Method	0.3	10.3389/fenvs.2022.903013
$\delta^{15}N$	NH_3	Waste Materials	-35.4‰	40.05	116.22	January, 2018	Passive sampling/+15.4‰	PT-IRMS	Standard Calibration Method	0.3	10.3389/fenvs.2022.903013
$\delta^{15}N$	NH_3	Waste Materials	-35.3‰	40.05	116.22	January, 2018	Passive sampling/+15.4‰	PT-IRMS	Standard Calibration Method	0.3	10.3389/fenvs.2022.903013
$\delta^{15}N$	NH_3	Waste Materials	-42.1‰	40.05	116.22	January, 2018	Passive sampling/+15.4‰	PT-IRMS	Standard Calibration Method	0.3	10.3389/fenvs.2022.903013
$\delta^{15}N$	NH_3	Waste Materials	-26.2‰	40.08	116.25	March-April, 2017	Passive sampling/+15.4‰	PT-IRMS	Standard Calibration Method	0.3	10.3389/fenvs.2022.903013
$\delta^{15}N$	NH_3	Waste Materials	-33.9‰	40.08	116.25	March-April, 2017	Passive sampling/+15.4‰	PT-IRMS	Standard Calibration Method	0.3	10.3389/fenvs.2022.903013
$\delta^{15}N$	NH_3	Waste Materials	-25.8‰	40.08	116.25	March-April, 2017	Passive sampling/+15.4‰	PT-IRMS	Standard Calibration Method	0.3	10.3389/fenvs.2022.903013
$\delta^{15}N$	NH_3	Waste Materials	-38.6‰	40.08	116.25	March-April, 2017	Passive sampling/+15.4‰	PT-IRMS	Standard Calibration Method	0.3	10.3389/fenvs.2022.903013

$\delta^{15}N$	NH_3	Waste Materials	-32.2‰	40.08	116.25	March-April, 2017	Passive sampling/+15.4‰	PT-IRMS	Standard Calibration Method	0.3	10.3389/fenvs.2022.903013
$\delta^{15}N$	NH_3	Waste Materials	-36.8‰	40.08	116.25	March-April, 2017	Passive sampling/+15.4‰	PT-IRMS	Standard Calibration Method	0.3	10.3389/fenvs.2022.903013
$\delta^{15}N$	NH_3	Waste Materials	-32.5‰	40.08	116.25	March-April, 2017	Passive sampling/+15.4‰	PT-IRMS	Standard Calibration Method	0.3	10.3389/fenvs.2022.903013
$\delta^{15}N$	NH_3	Waste Materials	-31.3‰	40.08	116.25	March-April, 2017	Passive sampling/+15.4‰	PT-IRMS	Standard Calibration Method	0.3	10.3389/fenvs.2022.903013
$\delta^{15}N$	NH_3	Waste Materials	-25‰	40.08	116.25	March-April, 2017	Passive sampling/+15.4‰	PT-IRMS	Standard Calibration Method	0.3	10.3389/fenvs.2022.903013
$\delta^{15}N$	NH_3	Waste Materials	-31.1‰	40.08	116.25	March-April, 2017	Passive sampling/+15.4‰	PT-IRMS	Standard Calibration Method	0.3	10.3389/fenvs.2022.903013
$\delta^{15}N$	NH_3	Waste Materials	-27.2‰	40.08	116.25	March-April, 2017	Passive sampling/+15.4‰	PT-IRMS	Standard Calibration Method	0.3	10.3389/fenvs.2022.903013
$\delta^{15}N$	NH_3	Waste Materials	-34.5‰	40.08	116.25	March-April, 2017	Passive sampling/+15.4‰	PT-IRMS	Standard Calibration Method	0.3	10.3389/fenvs.2022.903013
$\delta^{15}N$	NH_3	Waste Materials	-38.7‰	40.08	116.25	March-April, 2017	Passive sampling/+15.4‰	PT-IRMS	Standard Calibration Method	0.3	10.3389/fenvs.2022.903013
$\delta^{15}N$	NH_3	Waste Materials	-38.9‰	40.08	116.25	March-April, 2017	Passive sampling/+15.4‰	PT-IRMS	Standard Calibration Method	0.3	10.3389/fenvs.2022.903013
$\delta^{15}N$	NH_3	Waste Materials	-37.5‰	40.08	116.25	March-April, 2017	Passive sampling/+15.4‰	PT-IRMS	Standard Calibration Method	0.3	10.3389/fenvs.2022.903013
$\delta^{15}N$	NH_3	Waste Materials	-35.2‰	40.08	116.25	March-April, 2017	Passive sampling/+15.4‰	PT-IRMS	Standard Calibration Method	0.3	10.3389/fenvs.2022.903013
$\delta^{15}N$	NH_3	Waste Materials	-31.3‰	40.08	116.25	March-April, 2017	Passive sampling/+15.4‰	PT-IRMS	Standard Calibration Method	0.3	10.3389/fenvs.2022.903013
$\delta^{15}N$	NH_3	Waste Materials	-31.7‰	40.08	116.25	March-April, 2017	Passive sampling/+15.4‰	PT-IRMS	Standard Calibration Method	0.3	10.3389/fenvs.2022.903013
$\delta^{15}N$	NH_3	Waste Materials	-25‰	40.08	116.25	March-April, 2017	Passive sampling/+15.4‰	PT-IRMS	Standard Calibration Method	0.3	10.3389/fenvs.2022.903013

$\delta^{15}N$	NH_3	Waste Materials	-28‰	40.08	116.25	March-April, 2017	Passive sampling/+15.4‰	PT-IRMS	Standard Calibration Method	0.3	10.3389/fenvs.2022.903013
$\delta^{15}N$	NH_3	Waste Materials	-27.3‰	40.08	116.25	June-August, 2017	Passive sampling/+15.4‰	PT-IRMS	Standard Calibration Method	0.3	10.3389/fenvs.2022.903013
$\delta^{15}N$	NH_3	Waste Materials	-29.3‰	40.08	116.25	June-August, 2017	Passive sampling/+15.4‰	PT-IRMS	Standard Calibration Method	0.3	10.3389/fenvs.2022.903013
$\delta^{15}N$	NH_3	Waste Materials	-33.1‰	40.08	116.25	June-August, 2017	Passive sampling/+15.4‰	PT-IRMS	Standard Calibration Method	0.3	10.3389/fenvs.2022.903013
$\delta^{15}N$	NH_3	Waste Materials	-29.7‰	40.08	116.25	June-August, 2017	Passive sampling/+15.4‰	PT-IRMS	Standard Calibration Method	0.3	10.3389/fenvs.2022.903013
$\delta^{15}N$	NH_3	Waste Materials	-27.3‰	40.08	116.25	June-August, 2017	Passive sampling/+15.4‰	PT-IRMS	Standard Calibration Method	0.3	10.3389/fenvs.2022.903013
$\delta^{15}N$	NH_3	Waste Materials	-30.1‰	40.08	116.25	June-August, 2017	Passive sampling/+15.4‰	PT-IRMS	Standard Calibration Method	0.3	10.3389/fenvs.2022.903013
$\delta^{15}N$	NH_3	Waste Materials	-35.8‰	40.08	116.25	June-August, 2017	Passive sampling/+15.4‰	PT-IRMS	Standard Calibration Method	0.3	10.3389/fenvs.2022.903013
$\delta^{15}N$	NH_3	Waste Materials	-33.5‰	40.08	116.25	June-August, 2017	Passive sampling/+15.4‰	PT-IRMS	Standard Calibration Method	0.3	10.3389/fenvs.2022.903013
$\delta^{15}N$	NH_3	Waste Materials	-35.7‰	40.08	116.25	June-August, 2017	Passive sampling/+15.4‰	PT-IRMS	Standard Calibration Method	0.3	10.3389/fenvs.2022.903013
$\delta^{15}N$	NH_3	Waste Materials	-35.8‰	40.08	116.25	June-August, 2017	Passive sampling/+15.4‰	PT-IRMS	Standard Calibration Method	0.3	10.3389/fenvs.2022.903013
$\delta^{15}N$	NH_3	Waste Materials	-27.2‰	40.08	116.25	June-August, 2017	Passive sampling/+15.4‰	PT-IRMS	Standard Calibration Method	0.3	10.3389/fenvs.2022.903013
$\delta^{15}N$	NH_3	Waste Materials	-34.2‰	40.08	116.25	October, 2017	Passive sampling/+15.4‰	PT-IRMS	Standard Calibration Method	0.3	10.3389/fenvs.2022.903013
$\delta^{15}N$	NH_3	Waste Materials	-31.8‰	40.08	116.25	October, 2017	Passive sampling/+15.4‰	PT-IRMS	Standard Calibration Method	0.3	10.3389/fenvs.2022.903013
$\delta^{15}N$	NH_3	Waste Materials	-36.2‰	40.08	116.25	October, 2017	Passive sampling/+15.4‰	PT-IRMS	Standard Calibration Method	0.3	10.3389/fenvs.2022.903013

$\delta^{15}N$	NH_3	Waste Materials	-37.6‰	40.08	116.25	October, 2017	Passive sampling/+15.4‰	PT-IRMS	Standard Calibration Method	0.3	10.3389/fenvs.2022.903013
$\delta^{15}N$	NH_3	Waste Materials	-35.2‰	40.08	116.25	October, 2017	Passive sampling/+15.4‰	PT-IRMS	Standard Calibration Method	0.3	10.3389/fenvs.2022.903013
$\delta^{15}N$	NH_3	Waste Materials	-37.7‰	40.08	116.25	October, 2017	Passive sampling/+15.4‰	PT-IRMS	Standard Calibration Method	0.3	10.3389/fenvs.2022.903013
$\delta^{15}N$	NH_3	Waste Materials	-41.2‰	40.08	116.25	October, 2017	Passive sampling/+15.4‰	PT-IRMS	Standard Calibration Method	0.3	10.3389/fenvs.2022.903013
$\delta^{15}N$	NH_3	Waste Materials	-43.5‰	40.08	116.25	October, 2017	Passive sampling/+15.4‰	PT-IRMS	Standard Calibration Method	0.3	10.3389/fenvs.2022.903013
$\delta^{15}N$	NH_3	Waste Materials	-43.8‰	40.08	116.25	October, 2017	Passive sampling/+15.4‰	PT-IRMS	Standard Calibration Method	0.3	10.3389/fenvs.2022.903013
$\delta^{15}N$	NH_3	Waste Materials	-39.7‰	40.08	116.25	October, 2017	Passive sampling/+15.4‰	PT-IRMS	Standard Calibration Method	0.3	10.3389/fenvs.2022.903013
$\delta^{15}N$	NH_3	Waste Materials	-34.4‰	40.08	116.25	October, 2017	Passive sampling/+15.4‰	PT-IRMS	Standard Calibration Method	0.3	10.3389/fenvs.2022.903013
$\delta^{15}N$	NH_3	Waste Materials	-35.2‰	40.08	116.25	October, 2017	Passive sampling/+15.4‰	PT-IRMS	Standard Calibration Method	0.3	10.3389/fenvs.2022.903013
$\delta^{15}N$	NH_3	Waste Materials	-40.5‰	40.08	116.25	October, 2017	Passive sampling/+15.4‰	PT-IRMS	Standard Calibration Method	0.3	10.3389/fenvs.2022.903013
$\delta^{15}N$	NH_3	Waste Materials	-41.2‰	40.08	116.25	October, 2017	Passive sampling/+15.4‰	PT-IRMS	Standard Calibration Method	0.3	10.3389/fenvs.2022.903013
$\delta^{15}N$	NH_3	Waste Materials	-38.4‰	40.08	116.25	October, 2017	Passive sampling/+15.4‰	PT-IRMS	Standard Calibration Method	0.3	10.3389/fenvs.2022.903013
$\delta^{15}N$	NH_3	Waste Materials	-35.6‰	40.08	116.25	October, 2017	Passive sampling/+15.4‰	PT-IRMS	Standard Calibration Method	0.3	10.3389/fenvs.2022.903013
$\delta^{15}N$	NH_3	Waste Materials	-31.4‰	40.08	116.25	October, 2017	Passive sampling/+15.4‰	PT-IRMS	Standard Calibration Method	0.3	10.3389/fenvs.2022.903013
$\delta^{15}N$	NH_3	Waste Materials	-43‰	40.08	116.25	October, 2017	Passive sampling/+15.4‰	PT-IRMS	Standard Calibration Method	0.3	10.3389/fenvs.2022.903013

$\delta^{15}N$	NH_3	Waste Materials	-33.7‰	40.08	116.25	January, 2018	Passive sampling/+15.4‰	PT-IRMS	Standard Calibration Method	0.3	10.3389/fenvs.2022.903013
$\delta^{15}N$	NH_3	Waste Materials	-39.2‰	40.08	116.25	January, 2018	Passive sampling/+15.4‰	PT-IRMS	Standard Calibration Method	0.3	10.3389/fenvs.2022.903013
$\delta^{15}N$	NH_3	Waste Materials	-30‰	40.08	116.25	January, 2018	Passive sampling/+15.4‰	PT-IRMS	Standard Calibration Method	0.3	10.3389/fenvs.2022.903013
$\delta^{15}N$	NH_3	Waste Materials	-29.9‰	40.08	116.25	January, 2018	Passive sampling/+15.4‰	PT-IRMS	Standard Calibration Method	0.3	10.3389/fenvs.2022.903013
$\delta^{15}N$	NH_3	Waste Materials	-38.9‰	40.08	116.25	January, 2018	Passive sampling/+15.4‰	PT-IRMS	Standard Calibration Method	0.3	10.3389/fenvs.2022.903013
$\delta^{15}N$	NH_3	Waste Materials	-28.5‰	40.08	116.25	January, 2018	Passive sampling/+15.4‰	PT-IRMS	Standard Calibration Method	0.3	10.3389/fenvs.2022.903013
$\delta^{15}N$	NH_3	Waste Materials	-39.6‰	40.08	116.25	January, 2018	Passive sampling/+15.4‰	PT-IRMS	Standard Calibration Method	0.3	10.3389/fenvs.2022.903013
$\delta^{15}N$	NH_3	Waste Materials	-33.5‰	40.08	116.25	January, 2018	Passive sampling/+15.4‰	PT-IRMS	Standard Calibration Method	0.3	10.3389/fenvs.2022.903013
$\delta^{15}N$	NH_3	Waste Materials	-37.2‰	40.08	116.25	January, 2018	Passive sampling/+15.4‰	PT-IRMS	Standard Calibration Method	0.3	10.3389/fenvs.2022.903013
$\delta^{15}N$	NH_3	Waste Materials	-34.2‰	40.08	116.25	January, 2018	Passive sampling/+15.4‰	PT-IRMS	Standard Calibration Method	0.3	10.3389/fenvs.2022.903013
$\delta^{15}N$	NH_3	Waste Materials	-36‰	40.08	116.25	January, 2018	Passive sampling/+15.4‰	PT-IRMS	Standard Calibration Method	0.3	10.3389/fenvs.2022.903013
$\delta^{15}N$	NH_3	Waste Materials	-26.6‰	40.15	116.05	March-April, 2017	Passive sampling/+15.4‰	PT-IRMS	Standard Calibration Method	0.3	10.3389/fenvs.2022.903013
$\delta^{15}N$	NH_3	Waste Materials	-32.1‰	40.15	116.05	March-April, 2017	Passive sampling/+15.4‰	PT-IRMS	Standard Calibration Method	0.3	10.3389/fenvs.2022.903013
$\delta^{15}N$	NH_3	Waste Materials	-26.3‰	40.15	116.05	March-April, 2017	Passive sampling/+15.4‰	PT-IRMS	Standard Calibration Method	0.3	10.3389/fenvs.2022.903013
$\delta^{15}N$	NH_3	Waste Materials	-32.9‰	40.15	116.05	March-April, 2017	Passive sampling/+15.4‰	PT-IRMS	Standard Calibration Method	0.3	10.3389/fenvs.2022.903013

$\delta^{15}N$	NH_3	Waste Materials	-40.4‰	40.15	116.05	March-April, 2017	Passive sampling/+15.4‰	PT-IRMS	Standard Calibration Method	0.3	10.3389/fenvs.2022.903013
$\delta^{15}N$	NH_3	Waste Materials	-41.1‰	40.15	116.05	March-April, 2017	Passive sampling/+15.4‰	PT-IRMS	Standard Calibration Method	0.3	10.3389/fenvs.2022.903013
$\delta^{15}N$	NH_3	Waste Materials	-36.3‰	40.15	116.05	March-April, 2017	Passive sampling/+15.4‰	PT-IRMS	Standard Calibration Method	0.3	10.3389/fenvs.2022.903013
$\delta^{15}N$	NH_3	Waste Materials	-28.7‰	40.15	116.05	March-April, 2017	Passive sampling/+15.4‰	PT-IRMS	Standard Calibration Method	0.3	10.3389/fenvs.2022.903013
$\delta^{15}N$	NH_3	Waste Materials	-27.4‰	40.15	116.05	March-April, 2017	Passive sampling/+15.4‰	PT-IRMS	Standard Calibration Method	0.3	10.3389/fenvs.2022.903013
$\delta^{15}N$	NH_3	Waste Materials	-34.7‰	40.15	116.05	March-April, 2017	Passive sampling/+15.4‰	PT-IRMS	Standard Calibration Method	0.3	10.3389/fenvs.2022.903013
$\delta^{15}N$	NH_3	Waste Materials	-34.4‰	40.15	116.05	March-April, 2017	Passive sampling/+15.4‰	PT-IRMS	Standard Calibration Method	0.3	10.3389/fenvs.2022.903013
$\delta^{15}N$	NH_3	Waste Materials	-28.7‰	40.15	116.05	March-April, 2017	Passive sampling/+15.4‰	PT-IRMS	Standard Calibration Method	0.3	10.3389/fenvs.2022.903013
$\delta^{15}N$	NH_3	Waste Materials	-35.1‰	40.15	116.05	March-April, 2017	Passive sampling/+15.4‰	PT-IRMS	Standard Calibration Method	0.3	10.3389/fenvs.2022.903013
$\delta^{15}N$	NH_3	Waste Materials	-32.4‰	40.15	116.05	March-April, 2017	Passive sampling/+15.4‰	PT-IRMS	Standard Calibration Method	0.3	10.3389/fenvs.2022.903013
$\delta^{15}N$	NH_3	Waste Materials	-30.3‰	40.15	116.05	March-April, 2017	Passive sampling/+15.4‰	PT-IRMS	Standard Calibration Method	0.3	10.3389/fenvs.2022.903013
$\delta^{15}N$	NH_3	Waste Materials	-32.9‰	40.15	116.05	March-April, 2017	Passive sampling/+15.4‰	PT-IRMS	Standard Calibration Method	0.3	10.3389/fenvs.2022.903013
$\delta^{15}N$	NH_3	Waste Materials	-33.7‰	40.15	116.05	March-April, 2017	Passive sampling/+15.4‰	PT-IRMS	Standard Calibration Method	0.3	10.3389/fenvs.2022.903013
$\delta^{15}N$	NH_3	Waste Materials	-26.3‰	40.15	116.05	June-August, 2017	Passive sampling/+15.4‰	PT-IRMS	Standard Calibration Method	0.3	10.3389/fenvs.2022.903013
$\delta^{15}N$	NH_3	Waste Materials	-30‰	40.15	116.05	June-August, 2017	Passive sampling/+15.4‰	PT-IRMS	Standard Calibration Method	0.3	10.3389/fenvs.2022.903013

$\delta^{15}N$	NH_3	Waste Materials	-29.6‰	40.15	116.05	June-August, 2017	Passive sampling/+15.4‰	PT-IRMS	Standard Calibration Method	0.3	10.3389/fenvs.2022.903013
$\delta^{15}N$	NH_3	Waste Materials	-29.2‰	40.15	116.05	June-August, 2017	Passive sampling/+15.4‰	PT-IRMS	Standard Calibration Method	0.3	10.3389/fenvs.2022.903013
$\delta^{15}N$	NH_3	Waste Materials	-28.6‰	40.15	116.05	June-August, 2017	Passive sampling/+15.4‰	PT-IRMS	Standard Calibration Method	0.3	10.3389/fenvs.2022.903013
$\delta^{15}N$	NH_3	Waste Materials	-25‰	40.15	116.05	June-August, 2017	Passive sampling/+15.4‰	PT-IRMS	Standard Calibration Method	0.3	10.3389/fenvs.2022.903013
$\delta^{15}N$	NH_3	Waste Materials	-34.9‰	40.15	116.05	June-August, 2017	Passive sampling/+15.4‰	PT-IRMS	Standard Calibration Method	0.3	10.3389/fenvs.2022.903013
$\delta^{15}N$	NH_3	Waste Materials	-31.3‰	40.15	116.05	June-August, 2017	Passive sampling/+15.4‰	PT-IRMS	Standard Calibration Method	0.3	10.3389/fenvs.2022.903013
$\delta^{15}N$	NH_3	Waste Materials	-34.3‰	40.15	116.05	June-August, 2017	Passive sampling/+15.4‰	PT-IRMS	Standard Calibration Method	0.3	10.3389/fenvs.2022.903013
$\delta^{15}N$	NH_3	Waste Materials	-33.3‰	40.15	116.05	June-August, 2017	Passive sampling/+15.4‰	PT-IRMS	Standard Calibration Method	0.3	10.3389/fenvs.2022.903013
$\delta^{15}N$	NH_3	Waste Materials	-31.7‰	40.15	116.05	June-August, 2017	Passive sampling/+15.4‰	PT-IRMS	Standard Calibration Method	0.3	10.3389/fenvs.2022.903013
$\delta^{15}N$	NH_3	Waste Materials	-32.2‰	40.15	116.05	October, 2017	Passive sampling/+15.4‰	PT-IRMS	Standard Calibration Method	0.3	10.3389/fenvs.2022.903013
$\delta^{15}N$	NH_3	Waste Materials	-26.6‰	40.15	116.05	October, 2017	Passive sampling/+15.4‰	PT-IRMS	Standard Calibration Method	0.3	10.3389/fenvs.2022.903013
$\delta^{15}N$	NH_3	Waste Materials	-27.8‰	40.15	116.05	October, 2017	Passive sampling/+15.4‰	PT-IRMS	Standard Calibration Method	0.3	10.3389/fenvs.2022.903013
$\delta^{15}N$	NH_3	Waste Materials	-34.1‰	40.15	116.05	October, 2017	Passive sampling/+15.4‰	PT-IRMS	Standard Calibration Method	0.3	10.3389/fenvs.2022.903013
$\delta^{15}N$	NH_3	Waste Materials	-28.1‰	40.15	116.05	October, 2017	Passive sampling/+15.4‰	PT-IRMS	Standard Calibration Method	0.3	10.3389/fenvs.2022.903013
$\delta^{15}N$	NH_3	Waste Materials	-31.9‰	40.15	116.05	October, 2017	Passive sampling/+15.4‰	PT-IRMS	Standard Calibration Method	0.3	10.3389/fenvs.2022.903013

$\delta^{15}N$	NH_3	Waste Materials	-35.5‰	40.15	116.05	October, 2017	Passive sampling/+15.4‰	PT-IRMS	Standard Calibration Method	0.3	10.3389/fenvs.2022.903013
$\delta^{15}N$	NH_3	Waste Materials	-25.3‰	40.15	116.05	October, 2017	Passive sampling/+15.4‰	PT-IRMS	Standard Calibration Method	0.3	10.3389/fenvs.2022.903013
$\delta^{15}N$	NH_3	Waste Materials	-32.3‰	40.15	116.05	October, 2017	Passive sampling/+15.4‰	PT-IRMS	Standard Calibration Method	0.3	10.3389/fenvs.2022.903013
$\delta^{15}N$	NH_3	Waste Materials	-30.6‰	40.15	116.05	October, 2017	Passive sampling/+15.4‰	PT-IRMS	Standard Calibration Method	0.3	10.3389/fenvs.2022.903013
$\delta^{15}N$	NH_3	Waste Materials	-40.4‰	40.15	116.05	October, 2017	Passive sampling/+15.4‰	PT-IRMS	Standard Calibration Method	0.3	10.3389/fenvs.2022.903013
$\delta^{15}N$	NH_3	Waste Materials	-32.2‰	40.15	116.05	October, 2017	Passive sampling/+15.4‰	PT-IRMS	Standard Calibration Method	0.3	10.3389/fenvs.2022.903013
$\delta^{15}N$	NH_3	Waste Materials	-34.5‰	40.15	116.05	October, 2017	Passive sampling/+15.4‰	PT-IRMS	Standard Calibration Method	0.3	10.3389/fenvs.2022.903013
$\delta^{15}N$	NH_3	Waste Materials	-33‰	40.15	116.05	October, 2017	Passive sampling/+15.4‰	PT-IRMS	Standard Calibration Method	0.3	10.3389/fenvs.2022.903013
$\delta^{15}N$	NH_3	Waste Materials	-30.5‰	40.15	116.05	October, 2017	Passive sampling/+15.4‰	PT-IRMS	Standard Calibration Method	0.3	10.3389/fenvs.2022.903013
$\delta^{15}N$	NH_3	Waste Materials	-33.6‰	40.15	116.05	January, 2018	Passive sampling/+15.4‰	PT-IRMS	Standard Calibration Method	0.3	10.3389/fenvs.2022.903013
$\delta^{15}N$	NH_3	Waste Materials	-37.3‰	40.15	116.05	January, 2018	Passive sampling/+15.4‰	PT-IRMS	Standard Calibration Method	0.3	10.3389/fenvs.2022.903013
$\delta^{15}N$	NH_3	Waste Materials	-35.5‰	40.15	116.05	January, 2018	Passive sampling/+15.4‰	PT-IRMS	Standard Calibration Method	0.3	10.3389/fenvs.2022.903013
$\delta^{15}N$	NH_3	Waste Materials	-35‰	40.15	116.05	January, 2018	Passive sampling/+15.4‰	PT-IRMS	Standard Calibration Method	0.3	10.3389/fenvs.2022.903013
$\delta^{15}N$	NH_3	Waste Materials	-34.7‰	40.15	116.05	January, 2018	Passive sampling/+15.4‰	PT-IRMS	Standard Calibration Method	0.3	10.3389/fenvs.2022.903013
$\delta^{15}N$	NH_3	Waste Materials	-34.3‰	40.15	116.05	January, 2018	Passive sampling/+15.4‰	PT-IRMS	Standard Calibration Method	0.3	10.3389/fenvs.2022.903013

$\delta^{15}N$	NH_3	Waste Materials	-34.1‰	40.15	116.05	January, 2018	Passive sampling/+15.4‰	PT-IRMS	Standard Calibration Method	0.3	10.3389/fenvs.2022.903013
$\delta^{15}N$	NH_3	Waste Materials	-29.9‰	31.28	121.47	June, 2014	Passive sampling/+15.4‰	PT-IRMS	Standard Calibration Method	0.3	https://doi.org/10.5194/acp-16-11635-2016
$\delta^{15}N$	NH_3	Waste Materials	-31.4‰	31.28	121.47	June, 2014	Passive sampling/+15.4‰	PT-IRMS	Standard Calibration Method	0.3	https://doi.org/10.5194/acp-16-11635-2016
$\delta^{15}N$	NH_3	Waste Materials	-37.2‰	31.28	121.47	January, 2015	Passive sampling/+15.4‰	PT-IRMS	Standard Calibration Method	0.3	https://doi.org/10.5194/acp-16-11635-2016
$\delta^{15}N$	NH_3	Waste Materials	-36‰	31.28	121.47	January, 2015	Passive sampling/+15.4‰	PT-IRMS	Standard Calibration Method	0.3	https://doi.org/10.5194/acp-16-11635-2016
$\delta^{15}N$	NH_3	Waste Materials	-32.2‰	31.28	121.5	June, 2014	Passive sampling/+15.4‰	PT-IRMS	Standard Calibration Method	0.3	https://doi.org/10.5194/acp-16-11635-2016
$\delta^{15}N$	NH_3	Waste Materials	-30.7‰	31.28	121.5	June, 2014	Passive sampling/+15.4‰	PT-IRMS	Standard Calibration Method	0.3	https://doi.org/10.5194/acp-16-11635-2016
$\delta^{15}N$	NH_3	Waste Materials	-35.7‰	31.28	121.5	January, 2015	Passive sampling/+15.4‰	PT-IRMS	Standard Calibration Method	0.3	https://doi.org/10.5194/acp-16-11635-2016
$\delta^{15}N$	NH_3	Waste Materials	-37.6‰	31.28	121.5	January, 2015	Passive sampling/+15.4‰	PT-IRMS	Standard Calibration Method	0.3	https://doi.org/10.5194/acp-16-11635-2016
$\delta^{15}N$	NH_3	Waste Materials	-40.7‰	31.28	121.49	5-6 July, 2014	Passive sampling/+15.4‰	PT-IRMS	Standard Calibration Method	0.3	https://doi.org/10.5194/acp-16-11635-2016
$\delta^{15}N$	NH_3	Waste Materials	-41.9‰	31.28	121.49	5-6 July, 2014	Passive sampling/+15.4‰	PT-IRMS	Standard Calibration Method	0.3	https://doi.org/10.5194/acp-16-11635-2016
$\delta^{15}N$	NH_3	Waste Materials	-40.6‰	31.28	121.49	10-11 January, 2015	Passive sampling/+15.4‰	PT-IRMS	Standard Calibration Method	0.3	https://doi.org/10.5194/acp-16-11635-2016
$\delta^{15}N$	NH_3	Waste Materials	-40.9‰	31.28	121.49	10-11 January, 2015	Passive sampling/+15.4‰	PT-IRMS	Standard Calibration Method	0.3	https://doi.org/10.5194/acp-16-11635-2016
$\delta^{15}N$	NH_3	Waste Materials	-40.7‰	31.28	121.49	5-6 July, 2014	Passive sampling/+15.4‰	PT-IRMS	Standard Calibration Method	0.3	https://doi.org/10.5194/acp-16-11635-2016
$\delta^{15}N$	NH_3	Waste Materials	-42‰	31.28	121.49	5-6 July, 2014	Passive sampling/+15.4‰	PT-IRMS	Standard Calibration Method	0.3	https://doi.org/10.5194/acp-16-11635-2016

$\delta^{15}N$	NH_3	Waste Materials	-39.2‰	31.28	121.49	10-11 January, 2015	Passive sampling/+15.4‰	PT-IRMS	Standard Calibration Method	0.3	https://doi.org/10.5194/acp-16-11635-2016
$\delta^{15}N$	NH_3	Waste Materials	-41.9‰	31.28	121.49	10-11 January, 2015	Passive sampling/+15.4‰	PT-IRMS	Standard Calibration Method	0.3	https://doi.org/10.5194/acp-16-11635-2016
$\delta^{15}N$	NH_3	Waste Materials	-39.3‰	31.28	121.5	16 July, 2014	Passive sampling/+15.4‰	PT-IRMS	Standard Calibration Method	0.3	https://doi.org/10.5194/acp-16-11635-2016
$\delta^{15}N$	NH_3	Waste Materials	-38.1‰	31.28	121.5	7 August, 2014	Passive sampling/+15.4‰	PT-IRMS	Standard Calibration Method	0.3	https://doi.org/10.5194/acp-16-11635-2016
$\delta^{15}N$	NH_3	Waste Materials	-37.9‰	31.28	121.5	9 January, 2015	Passive sampling/+15.4‰	PT-IRMS	Standard Calibration Method	0.3	https://doi.org/10.5194/acp-16-11635-2016
$\delta^{15}N$	NH_3	Waste Materials	-39.2‰	31.28	121.5	12 January, 2015	Passive sampling/+15.4‰	PT-IRMS	Standard Calibration Method	0.3	https://doi.org/10.5194/acp-16-11635-2016
$\delta^{15}N$	NH_3	Waste Materials	-37.3‰	31.28	121.5	13 July, 2014	Passive sampling/+15.4‰	PT-IRMS	Standard Calibration Method	0.3	https://doi.org/10.5194/acp-16-11635-2016
$\delta^{15}N$	NH_3	Waste Materials	-39‰	31.28	121.5	5 August, 2014	Passive sampling/+15.4‰	PT-IRMS	Standard Calibration Method	0.3	https://doi.org/10.5194/acp-16-11635-2016
$\delta^{15}N$	NH_3	Waste Materials	-37.4‰	31.28	121.5	7 January, 2015	Passive sampling/+15.4‰	PT-IRMS	Standard Calibration Method	0.3	https://doi.org/10.5194/acp-16-11635-2016
$\delta^{15}N$	NH_3	Waste Materials	-39.6‰	31.28	121.5	14 January, 2015	Passive sampling/+15.4‰	PT-IRMS	Standard Calibration Method	0.3	https://doi.org/10.5194/acp-16-11635-2016
$\delta^{15}N$	NH_3	Waste Materials	-39.3‰	31.22	121.47	July-August, 2014	Passive sampling/+15.4‰	PT-IRMS	Standard Calibration Method	0.3	https://doi.org/10.1371/journal.pone.0144661
$\delta^{15}N$	NH_3	Waste Materials	-38.1‰	31.22	121.47	July-August, 2014	Passive sampling/+15.4‰	PT-IRMS	Standard Calibration Method	0.3	https://doi.org/10.1371/journal.pone.0144661
$\delta^{15}N$	NH_3	Waste Materials	-37.9‰	31.22	121.47	December, 2014-March, 2015	Passive sampling/+15.4‰	PT-IRMS	Standard Calibration Method	0.3	https://doi.org/10.1371/journal.pone.0144661
$\delta^{15}N$	NH_3	Waste Materials	-39.2‰	31.22	121.47	December, 2014-March, 2015	Passive sampling/+15.4‰	PT-IRMS	Standard Calibration Method	0.3	https://doi.org/10.1371/journal.pone.0144661
$\delta^{15}N$	NH_3	Waste Materials	-37.3‰	31.22	121.47	July-August, 2014	Passive sampling/+15.4‰	PT-IRMS	Standard Calibration Method	0.3	https://doi.org/10.1371/journal.pone.0144661

$\delta^{15}N$	NH_3	Waste Materials	-39‰	31.22	121.47	July-August, 2014	Passive sampling/+15.4‰	PT-IRMS	Standard Calibration Method	0.3	https://doi.org/10.1371/journal.pone.0144661
$\delta^{15}N$	NH_3	Waste Materials	-37.4‰	31.22	121.47	December, 2014-March, 2015	Passive sampling/+15.4‰	PT-IRMS	Standard Calibration Method	0.3	https://doi.org/10.1371/journal.pone.0144661
$\delta^{15}N$	NH_3	Waste Materials	-39.6‰	31.22	121.47	December, 2014-March, 2015	Passive sampling/+15.4‰	PT-IRMS	Standard Calibration Method	0.3	https://doi.org/10.1371/journal.pone.0144661
$\delta^{15}N$	NO_x	Biomass Burning	-5.20‰	34.81	117.32	January, 2022	Active sampling	IRMS	Standard Calibration Method	0.5	https://doi.org/10.1016/j.envpol.2022.119238
$\delta^{15}N$	NO_x	Biomass Burning	-5.80‰	34.81	117.32	April, 2022	Active sampling	IRMS	Standard Calibration Method	0.5	https://doi.org/10.1016/j.envpol.2022.119238
$\delta^{15}N$	NO_x	Biomass Burning	-0.10‰	34.81	117.32	July, 2022	Active sampling	IRMS	Standard Calibration Method	0.5	https://doi.org/10.1016/j.envpol.2022.119238
$\delta^{15}N$	NO_x	Biomass Burning	0.50‰	37.52	122.13	October, 2022	Active sampling	IRMS	Standard Calibration Method	0.5	https://doi.org/10.1016/j.envpol.2022.119238
$\delta^{15}N$	NO_x	Biomass Burning	-1.40‰	37.52	122.13	January, 2022	Active sampling	IRMS	Standard Calibration Method	0.5	https://doi.org/10.1016/j.envpol.2022.119238
$\delta^{15}N$	NO_x	Biomass Burning	-1.50‰	37.52	122.13	April, 2022	Active sampling	IRMS	Standard Calibration Method	0.5	https://doi.org/10.1016/j.envpol.2022.119238
$\delta^{15}N$	NO_x	Biomass Burning	-2.90‰	37.52	122.13	July, 2022	Active sampling	IRMS	Standard Calibration Method	0.5	https://doi.org/10.1016/j.envpol.2022.119238
$\delta^{15}N$	NO_x	Biomass Burning	-2.60‰	37.52	122.13	October, 2022	Active sampling	IRMS	Standard Calibration Method	0.5	https://doi.org/10.1016/j.envpol.2022.119238
$\delta^{15}N$	NO_x	Biomass Burning	-0.60‰	34.81	117.32	January, 2022	Active sampling	IRMS	Standard Calibration Method	0.5	https://doi.org/10.1016/j.envpol.2022.119238
$\delta^{15}N$	NO_x	Biomass Burning	-2.00‰	31.22	121.53	April, 2022	Active sampling	IRMS	Standard Calibration Method	0.5	https://doi.org/10.1016/j.envpol.2022.119238
$\delta^{15}N$	NO_x	Biomass Burning	-1.30‰	37.52	122.13	July, 2022	Active sampling	IRMS	Standard Calibration Method	0.5	https://doi.org/10.1016/j.envpol.2022.119238
$\delta^{15}N$	NO_x	Biomass Burning	1.80‰	34.81	117.32	October, 2022	Active sampling	IRMS	Standard Calibration Method	0.5	https://doi.org/10.1016/j.envpol.2022.119238

$\delta^{15}N$	NO_x	Biomass Burning	-2.20‰	34.81	117.32	January, 2022	Active sampling	IRMS	Standard Calibration Method	0.5	https://doi.org/10.1016/j.envpol.2022.119238
$\delta^{15}N$	NO_x	Biomass Burning	-3.70‰	37.52	122.13	April, 2022	Active sampling	IRMS	Standard Calibration Method	0.5	https://doi.org/10.1016/j.envpol.2022.119238
$\delta^{15}N$	NO_x	Biomass Burning	-6.90‰	34.81	117.32	July, 2022	Active sampling	IRMS	Standard Calibration Method	0.5	https://doi.org/10.1016/j.envpol.2022.119238
$\delta^{15}N$	NO_x	Biomass Burning	-2.50‰	37.52	122.13	October, 2022	Active sampling	IRMS	Standard Calibration Method	0.5	https://doi.org/10.1016/j.envpol.2022.119238
$\delta^{15}N$	NO_x	Biomass Burning	-1.40‰	34.81	117.32	January, 2022	Active sampling	IRMS	Standard Calibration Method	0.5	https://doi.org/10.1016/j.envpol.2022.119238
$\delta^{15}N$	NO_x	Biomass Burning	-0.20‰	31.22	121.53	April, 2022	Active sampling	IRMS	Standard Calibration Method	0.5	https://doi.org/10.1016/j.envpol.2022.119238
$\delta^{15}N$	NO_x	Biomass Burning	2.30‰	34.81	117.32	July, 2022	Active sampling	IRMS	Standard Calibration Method	0.5	https://doi.org/10.1016/j.envpol.2022.119238
$\delta^{15}N$	NO_x	Biomass Burning	0.70‰	37.52	122.13	October, 2022	Active sampling	IRMS	Standard Calibration Method	0.5	https://doi.org/10.1016/j.envpol.2022.119238
$\delta^{15}N$	NO_x	Biomass Burning	0.80‰	31.22	121.53	October, 2022	Active sampling	IRMS	Standard Calibration Method	0.5	https://doi.org/10.1016/j.envpol.2022.119238
$\delta^{15}N$	NO_x	Biomass Burning	-3.00‰	30.26	120.19	January, 2021	Active sampling	IRMS	Standard Calibration Method	0.2	https://doi.org/10.1016/j.scitotenv.2021.149857
$\delta^{15}N$	NO_x	Biomass Burning	-0.90‰	30.26	120.19	April, 2021	Active sampling	IRMS	Standard Calibration Method	0.2	https://doi.org/10.1016/j.scitotenv.2021.149857
$\delta^{15}N$	NO_x	Biomass Burning	0.20‰	30.26	120.19	July, 2021	Active sampling	IRMS	Standard Calibration Method	0.2	https://doi.org/10.1016/j.scitotenv.2021.149857
$\delta^{15}N$	NO_x	Biomass Burning	-7.90‰	30.26	120.19	October, 2021	Active sampling	IRMS	Standard Calibration Method	0.2	https://doi.org/10.1016/j.scitotenv.2021.149857
$\delta^{15}N$	NO_x	Biomass Burning	-5.70‰	30.26	120.19	January, 2021	Active sampling	IRMS	Standard Calibration Method	0.2	https://doi.org/10.1016/j.scitotenv.2021.149857
$\delta^{15}N$	NO_x	Biomass Burning	-3.60‰	30.26	120.19	April, 2021	Active sampling	IRMS	Standard Calibration Method	0.2	https://doi.org/10.1016/j.scitotenv.2021.149857

$\delta^{15}N$	NO_x	Biomass Burning	2.60‰	30.26	120.19	July, 2021	Active sampling	IRMS	Standard Calibration Method	0.2	https://doi.org/10.1016/j.scitotenv.2021.149857
$\delta^{15}N$	NO_x	Biomass Burning	0.20‰	30.26	120.19	October, 2021	Active sampling	IRMS	Standard Calibration Method	0.2	https://doi.org/10.1016/j.scitotenv.2021.149857
$\delta^{15}N$	NO_x	Biomass Burning	3.10‰	30.26	120.19	January, 2021	Active sampling	IRMS	Standard Calibration Method	0.2	https://doi.org/10.1016/j.scitotenv.2021.149857
$\delta^{15}N$	NO_x	Biomass Burning	-1.40‰	30.26	120.19	April, 2021	Active sampling	IRMS	Standard Calibration Method	0.2	https://doi.org/10.1016/j.scitotenv.2021.149857
$\delta^{15}N$	NO_x	Biomass Burning	-1.60‰	30.26	120.19	July, 2021	Active sampling	IRMS	Standard Calibration Method	0.2	https://doi.org/10.1016/j.scitotenv.2021.149857
$\delta^{15}N$	NO_x	Biomass Burning	1.50‰	30.26	120.19	October, 2021	Active sampling	IRMS	Standard Calibration Method	0.2	https://doi.org/10.1016/j.scitotenv.2021.149857
$\delta^{15}N$	NO_x	Biomass Burning	2.20‰	30.26	120.19	January, 2021	Active sampling	IRMS	Standard Calibration Method	0.2	https://doi.org/10.1016/j.scitotenv.2021.149857
$\delta^{15}N$	NO_x	Biomass Burning	1.60‰	30.26	120.19	April, 2021	Active sampling	IRMS	Standard Calibration Method	0.2	https://doi.org/10.1016/j.scitotenv.2021.149857
$\delta^{15}N$	NO_x	Biomass Burning	2.10‰	30.26	120.19	July, 2021	Active sampling	IRMS	Standard Calibration Method	0.2	https://doi.org/10.1016/j.scitotenv.2021.149857
$\delta^{15}N$	NO_x	Biomass Burning	-9.80‰	30.26	120.19	October, 2021	Active sampling	IRMS	Standard Calibration Method	0.2	https://doi.org/10.1016/j.scitotenv.2021.149857
$\delta^{15}N$	NO_x	Biomass Burning	-8.70‰	30.26	120.19	January, 2021	Active sampling	IRMS	Standard Calibration Method	0.2	https://doi.org/10.1016/j.scitotenv.2021.149857
$\delta^{15}N$	NO_x	Biomass Burning	-5.40‰	30.26	120.19	April, 2021	Active sampling	IRMS	Standard Calibration Method	0.2	https://doi.org/10.1016/j.scitotenv.2021.149857
$\delta^{15}N$	NO_x	Biomass Burning	-1.40‰	30.26	120.19	July, 2021	Active sampling	IRMS	Standard Calibration Method	0.2	https://doi.org/10.1016/j.scitotenv.2021.149857
$\delta^{15}N$	NO_x	Biomass Burning	-9.40‰	30.26	120.19	October, 2021	Active sampling	IRMS	Standard Calibration Method	0.2	https://doi.org/10.1016/j.scitotenv.2021.149857
$\delta^{15}N$	NO_x	Biomass Burning	-5.80‰	30.26	120.19	January, 2021	Active sampling	IRMS	Standard Calibration Method	0.2	https://doi.org/10.1016/j.scitotenv.2021.149857

$\delta^{15}N$	NO_x	Biomass Burning	-3.50‰	30.26	120.19	April, 2021	Active sampling	IRMS	Standard Calibration Method	0.2	https://doi.org/10.1016/j.scitotenv.2021.149857
$\delta^{15}N$	NO_x	Biomass Burning	-3.70‰	30.26	120.19	July, 2021	Active sampling	IRMS	Standard Calibration Method	0.2	https://doi.org/10.1016/j.scitotenv.2021.149857
$\delta^{15}N$	NO_x	Biomass Burning	-3.30‰	30.26	120.19	October, 2021	Active sampling	IRMS	Standard Calibration Method	0.2	https://doi.org/10.1016/j.scitotenv.2021.149857
$\delta^{15}N$	NO_x	Biomass Burning	-11.90‰	30.26	120.19	January, 2021	Active sampling	IRMS	Standard Calibration Method	0.2	https://doi.org/10.1016/j.scitotenv.2021.149857
$\delta^{15}N$	NO_x	Biomass Burning	-6.60‰	30.26	120.19	April, 2021	Active sampling	IRMS	Standard Calibration Method	0.2	https://doi.org/10.1016/j.scitotenv.2021.149857
$\delta^{15}N$	NO_x	Biomass Burning	-6.80‰	30.26	120.19	July, 2021	Active sampling	IRMS	Standard Calibration Method	0.2	https://doi.org/10.1016/j.scitotenv.2021.149857
$\delta^{15}N$	NO_x	Biomass Burning	-4.50‰	30.26	120.19	October, 2021	Active sampling	IRMS	Standard Calibration Method	0.2	https://doi.org/10.1016/j.scitotenv.2021.149857
$\delta^{15}N$	NO_x	Biomass Burning	-0.50‰	30.26	120.19	January, 2021	Active sampling	IRMS	Standard Calibration Method	0.2	https://doi.org/10.1016/j.scitotenv.2021.149857
$\delta^{15}N$	NO_x	Biomass Burning	-3.30‰	30.26	120.19	April, 2021	Active sampling	IRMS	Standard Calibration Method	0.2	https://doi.org/10.1016/j.scitotenv.2021.149857
$\delta^{15}N$	NO_x	Biomass Burning	-3.50‰	30.26	120.19	July, 2021	Active sampling	IRMS	Standard Calibration Method	0.2	https://doi.org/10.1016/j.scitotenv.2021.149857
$\delta^{15}N$	NO_x	Biomass Burning	-1.30‰	30.26	120.19	October, 2021	Active sampling	IRMS	Standard Calibration Method	0.2	https://doi.org/10.1016/j.scitotenv.2021.149857
$\delta^{15}N$	NO_x	Biomass Burning	0.90‰	30.26	120.19	January, 2021	Active sampling	IRMS	Standard Calibration Method	0.2	https://doi.org/10.1016/j.scitotenv.2021.149857
$\delta^{15}N$	NO_x	Biomass Burning	-11.00‰	30.26	120.19	April, 2021	Active sampling	IRMS	Standard Calibration Method	0.2	https://doi.org/10.1016/j.scitotenv.2021.149857
$\delta^{15}N$	NO_x	Biomass Burning	-9.00‰	30.26	120.19	July, 2021	Active sampling	IRMS	Standard Calibration Method	0.2	https://doi.org/10.1016/j.scitotenv.2021.149857
$\delta^{15}N$	NO_x	Biomass Burning	-8.90‰	30.26	120.19	October, 2021	Active sampling	IRMS	Standard Calibration Method	0.2	https://doi.org/10.1016/j.scitotenv.2021.149857

$\delta^{15}N$	NO_x	Biomass Burning	-2.40‰	30.26	120.19	January, 2021	Active sampling	IRMS	Standard Calibration Method	0.2	https://doi.org/10.1016/j.scitotenv.2021.149857
$\delta^{15}N$	NO_x	Biomass Burning	-1.90‰	30.26	120.19	April, 2021	Active sampling	IRMS	Standard Calibration Method	0.2	https://doi.org/10.1016/j.scitotenv.2021.149857
$\delta^{15}N$	NO_x	Biomass Burning	-0.90‰	30.26	120.19	July, 2021	Active sampling	IRMS	Standard Calibration Method	0.2	https://doi.org/10.1016/j.scitotenv.2021.149857
$\delta^{15}N$	NO_x	Biomass Burning	-9.20‰	30.26	120.19	October, 2021	Active sampling	IRMS	Standard Calibration Method	0.2	https://doi.org/10.1016/j.scitotenv.2021.149857
$\delta^{15}N$	NO_x	Biomass Burning	-9.30‰	30.26	120.19	January, 2021	Active sampling	IRMS	Standard Calibration Method	0.2	https://doi.org/10.1016/j.scitotenv.2021.149857
$\delta^{15}N$	NO_x	Biomass Burning	-8.40‰	30.26	120.19	October, 2021	Active sampling	IRMS	Standard Calibration Method	0.2	https://doi.org/10.1016/j.scitotenv.2021.149857
$\delta^{15}N$	NO_x	Biomass Burning	-2.70‰	46.87	-113.99	April, 2016	Active sampling	IRMS	Standard Calibration Method	0.4	https://doi.org/10.1021/acs.est.6b03510
$\delta^{15}N$	NO_x	Biomass Burning	-5.00‰	46.87	-113.99	January, 2016	Active sampling	IRMS	Standard Calibration Method	0.4	https://doi.org/10.1021/acs.est.6b03510
$\delta^{15}N$	NO_x	Biomass Burning	-5.90‰	46.87	-113.99	July, 2016	Active sampling	IRMS	Standard Calibration Method	0.4	https://doi.org/10.1021/acs.est.6b03510
$\delta^{15}N$	NO_x	Biomass Burning	-7.20‰	46.87	-113.99	October, 2016	Active sampling	IRMS	Standard Calibration Method	0.4	https://doi.org/10.1021/acs.est.6b03510
$\delta^{15}N$	NO_x	Biomass Burning	3.20‰	46.87	-113.99	April, 2016	Active sampling	IRMS	Standard Calibration Method	0.4	https://doi.org/10.1021/acs.est.6b03510
$\delta^{15}N$	NO_x	Biomass Burning	2.60‰	46.87	-113.99	January, 2016	Active sampling	IRMS	Standard Calibration Method	0.4	https://doi.org/10.1021/acs.est.6b03510
$\delta^{15}N$	NO_x	Biomass Burning	3.80‰	46.87	-113.99	July, 2016	Active sampling	IRMS	Standard Calibration Method	0.4	https://doi.org/10.1021/acs.est.6b03510
$\delta^{15}N$	NO_x	Biomass Burning	8.10‰	46.87	-113.99	October, 2016	Active sampling	IRMS	Standard Calibration Method	0.4	https://doi.org/10.1021/acs.est.6b03510
$\delta^{15}N$	NO_x	Biomass Burning	12.00‰	46.87	-113.99	April, 2016	Active sampling	IRMS	Standard Calibration Method	0.4	https://doi.org/10.1021/acs.est.6b03510

$\delta^{15}N$	NO_x	Biomass Burning	0.90‰	46.87	-113.99	January, 2016	Active sampling	IRMS	Standard Calibration Method	0.4	https://doi.org/10.1021/acs.est.6b03510
$\delta^{15}N$	NO_x	Biomass Burning	1.30‰	46.87	-113.99	July, 2016	Active sampling	IRMS	Standard Calibration Method	0.4	https://doi.org/10.1021/acs.est.6b03510
$\delta^{15}N$	NO_x	Biomass Burning	1.60‰	46.87	-113.99	October, 2016	Active sampling	IRMS	Standard Calibration Method	0.4	https://doi.org/10.1021/acs.est.6b03510
$\delta^{15}N$	NO_x	Biomass Burning	-1.50‰	46.87	-113.99	April, 2016	Active sampling	IRMS	Standard Calibration Method	0.4	https://doi.org/10.1021/acs.est.6b03510
$\delta^{15}N$	NO_x	Biomass Burning	-1.30‰	46.87	-113.99	January, 2016	Active sampling	IRMS	Standard Calibration Method	0.4	https://doi.org/10.1021/acs.est.6b03510
$\delta^{15}N$	NO_x	Biomass Burning	-0.10‰	46.87	-113.99	July, 2016	Active sampling	IRMS	Standard Calibration Method	0.4	https://doi.org/10.1021/acs.est.6b03510
$\delta^{15}N$	NO_x	Biomass Burning	0.50‰	46.87	-113.99	October, 2016	Active sampling	IRMS	Standard Calibration Method	0.4	https://doi.org/10.1021/acs.est.6b03510
$\delta^{15}N$	NO_x	Biomass Burning	4.40‰	46.87	-113.99	April, 2016	Active sampling	IRMS	Standard Calibration Method	0.4	https://doi.org/10.1021/acs.est.6b03510
$\delta^{15}N$	NO_x	Biomass Burning	2.00‰	46.87	-113.99	January, 2016	Active sampling	IRMS	Standard Calibration Method	0.4	https://doi.org/10.1021/acs.est.6b03510
$\delta^{15}N$	NO_x	Biomass Burning	2.90‰	46.87	-113.99	July, 2016	Active sampling	IRMS	Standard Calibration Method	0.4	https://doi.org/10.1021/acs.est.6b03510
$\delta^{15}N$	NO_x	Biomass Burning	1.70‰	46.87	-113.99	October, 2016	Active sampling	IRMS	Standard Calibration Method	0.4	https://doi.org/10.1021/acs.est.6b03510
$\delta^{15}N$	NO_x	Biomass Burning	3.20‰	46.87	-113.99	April, 2016	Active sampling	IRMS	Standard Calibration Method	0.4	https://doi.org/10.1021/acs.est.6b03510
$\delta^{15}N$	NO_x	Biomass Burning	1.60‰	46.87	-113.99	January, 2016	Active sampling	IRMS	Standard Calibration Method	0.4	https://doi.org/10.1021/acs.est.6b03510
$\delta^{15}N$	NO_x	Biomass Burning	-0.30‰	46.87	-113.99	July, 2016	Active sampling	IRMS	Standard Calibration Method	0.4	https://doi.org/10.1021/acs.est.6b03510
$\delta^{15}N$	NO_x	Biomass Burning	-0.90‰	46.87	-113.99	October, 2016	Active sampling	IRMS	Standard Calibration Method	0.4	https://doi.org/10.1021/acs.est.6b03510

$\delta^{15}N$	NO_x	Biomass Burning	-1.10‰	46.87	-113.99	April, 2016	Active sampling	IRMS	Standard Calibration Method	1.3	https://doi.org/10.5194/amt-12-6303-2019
$\delta^{15}N$	NO_x	Biomass Burning	2.30‰	46.87	-113.99	January, 2016	Active sampling	IRMS	Standard Calibration Method	1.3	https://doi.org/10.5194/amt-12-6303-2019
$\delta^{15}N$	NO_x	Biomass Burning	-3.60‰	46.87	-113.99	July, 2016	Active sampling	IRMS	Standard Calibration Method	1.3	https://doi.org/10.5194/amt-12-6303-2019
$\delta^{15}N$	NO_x	Biomass Burning	5.20‰	46.87	-113.99	October, 2016	Active sampling	IRMS	Standard Calibration Method	1.3	https://doi.org/10.5194/amt-12-6303-2019
$\delta^{15}N$	NO_x	Biomass Burning	7.00‰	46.87	-113.99	April, 2016	Active sampling	IRMS	Standard Calibration Method	1.3	https://doi.org/10.5194/amt-12-6303-2019
$\delta^{15}N$	NO_x	Biomass Burning	-1.10‰	46.87	-113.99	January, 2016	Active sampling	IRMS	Standard Calibration Method	1.3	https://doi.org/10.5194/amt-12-6303-2019
$\delta^{15}N$	NO_x	Biomass Burning	1.40‰	46.87	-113.99	July, 2016	Active sampling	IRMS	Standard Calibration Method	1.3	https://doi.org/10.5194/amt-12-6303-2019
$\delta^{15}N$	NO_x	Biomass Burning	0.10‰	46.87	-113.99	October, 2016	Active sampling	IRMS	Standard Calibration Method	1.3	https://doi.org/10.5194/amt-12-6303-2019
$\delta^{15}N$	NO_x	Biomass Burning	-1.30‰	46.87	-113.99	April, 2016	Active sampling	IRMS	Standard Calibration Method	1.3	https://doi.org/10.5194/amt-12-6303-2019
$\delta^{15}N$	NO_x	Biomass Burning	-4.30‰	46.87	-113.99	January, 2016	Active sampling	IRMS	Standard Calibration Method	1.3	https://doi.org/10.5194/amt-12-6303-2019
$\delta^{15}N$	NO_x	Biomass Burning	1.90‰	46.87	-113.99	July, 2016	Active sampling	IRMS	Standard Calibration Method	1.3	https://doi.org/10.5194/amt-12-6303-2019
$\delta^{15}N$	NO_x	Biomass Burning	3.30‰	46.87	-113.99	October, 2016	Active sampling	IRMS	Standard Calibration Method	1.3	https://doi.org/10.5194/amt-12-6303-2019
$\delta^{15}N$	NO_x	Biomass Burning	2.10‰	46.87	-113.99	April, 2016	Active sampling	IRMS	Standard Calibration Method	1.3	https://doi.org/10.5194/amt-12-6303-2019
$\delta^{15}N$	NO_x	Biomass Burning	3.40‰	46.87	-113.99	January, 2016	Active sampling	IRMS	Standard Calibration Method	1.3	https://doi.org/10.5194/amt-12-6303-2019
$\delta^{15}N$	NO_x	Biomass Burning	-7.50‰	46.87	-113.99	July, 2016	Active sampling	IRMS	Standard Calibration Method	1.3	https://doi.org/10.5194/amt-12-6303-2019

$\delta^{15}N$	NO_x	Biomass Burning	-7.40‰	46.87	-113.99	October, 2016	Active sampling	IRMS	Standard Calibration Method	1.3	https://doi.org/10.5194/amt-12-6303-2019
$\delta^{15}N$	NO_x	Biomass Burning	-9.90‰	46.87	-113.99	January, 2016	Active sampling	IRMS	Standard Calibration Method	1.3	https://doi.org/10.5194/amt-12-6303-2019
$\delta^{15}N$	NO_x	Biomass Burning	-10.60‰	46.87	-113.99	July, 2016	Active sampling	IRMS	Standard Calibration Method	1.3	https://doi.org/10.5194/amt-12-6303-2019
$\delta^{15}N$	NO_x	Biomass Burning	-8.90‰	46.87	-113.99	October, 2016	Active sampling	IRMS	Standard Calibration Method	1.3	https://doi.org/10.5194/amt-12-6303-2019
$\delta^{15}N$	NO_x	Coal Combustion	21.90‰	32.06	118.80	April, 2016	Active sampling	IRMS	Standard Calibration Method	1.5	10.11766/trxb201604180064 in Chinese with English abstract
$\delta^{15}N$	NO_x	Coal Combustion	2.60‰	38.47	106.27	January, 2022	Active sampling	IRMS	Standard Calibration Method	0.5	https://doi.org/10.1016/j.envpol.2022.119238
$\delta^{15}N$	NO_x	Coal Combustion	4.60‰	33.74	113.30	April, 2022	Active sampling	IRMS	Standard Calibration Method	0.5	https://doi.org/10.1016/j.envpol.2022.119238
$\delta^{15}N$	NO_x	Coal Combustion	-0.10‰	33.74	113.30	July, 2022	Active sampling	IRMS	Standard Calibration Method	0.5	https://doi.org/10.1016/j.envpol.2022.119238
$\delta^{15}N$	NO_x	Coal Combustion	3.50‰	46.65	131.17	October, 2022	Active sampling	IRMS	Standard Calibration Method	0.5	https://doi.org/10.1016/j.envpol.2022.119238
$\delta^{15}N$	NO_x	Coal Combustion	-0.20‰	37.52	111.14	January, 2022	Active sampling	IRMS	Standard Calibration Method	0.5	https://doi.org/10.1016/j.envpol.2022.119238
$\delta^{15}N$	NO_x	Coal Combustion	0.00‰	37.52	111.14	April, 2022	Active sampling	IRMS	Standard Calibration Method	0.5	https://doi.org/10.1016/j.envpol.2022.119238
$\delta^{15}N$	NO_x	Coal Combustion	0.00‰	36.18	113.08	July, 2022	Active sampling	IRMS	Standard Calibration Method	0.5	https://doi.org/10.1016/j.envpol.2022.119238
$\delta^{15}N$	NO_x	Coal Combustion	20.60‰	47.61	-122.33	6 May, 2009	Active sampling	CF-IRMS	Standard Calibration Method	0.2	https://doi.org/10.1021/es203355v
$\delta^{15}N$	NO_x	Coal Combustion	21.00‰	47.61	-122.33	6 May, 2009	Active sampling	CF-IRMS	Standard Calibration Method	0.2	https://doi.org/10.1021/es203355v
$\delta^{15}N$	NO_x	Coal Combustion	20.90‰	47.61	-122.33	6 May, 2009	Active sampling	CF-IRMS	Standard Calibration Method	0.2	https://doi.org/10.1021/es203355v

$\delta^{15}N$	NO_x	Coal Combustion	15.50‰	47.61	-122.33	6 May, 2009	Active sampling	CF-IRMS	Standard Calibration Method	0.2	https://doi.org/10.1021/es203355v
$\delta^{15}N$	NO_x	Coal Combustion	19.30‰	47.61	-122.33	6 May, 2009	Active sampling	CF-IRMS	Standard Calibration Method	0.2	https://doi.org/10.1021/es203355v
$\delta^{15}N$	NO_x	Coal Combustion	9.20‰	47.61	-122.33	8 December, 2009	Active sampling	CF-IRMS	Standard Calibration Method	0.2	https://doi.org/10.1021/es203355v
$\delta^{15}N$	NO_x	Coal Combustion	9.00‰	47.61	-122.33	8 December, 2009	Active sampling	CF-IRMS	Standard Calibration Method	0.2	https://doi.org/10.1021/es203355v
$\delta^{15}N$	NO_x	Coal Combustion	10.40‰	47.61	-122.33	8 December, 2009	Active sampling	CF-IRMS	Standard Calibration Method	0.2	https://doi.org/10.1021/es203355v
$\delta^{15}N$	NO_x	Coal Combustion	10.50‰	47.61	-122.33	8 December, 2009	Active sampling	CF-IRMS	Standard Calibration Method	0.2	https://doi.org/10.1021/es203355v
$\delta^{15}N$	NO_x	Coal Combustion	11.00‰	47.61	-122.33	8 December, 2009	Active sampling	CF-IRMS	Standard Calibration Method	0.2	https://doi.org/10.1021/es203355v
$\delta^{15}N$	NO_x	Coal Combustion	11.50‰	47.61	-122.33	8 December, 2009	Active sampling	CF-IRMS	Standard Calibration Method	0.2	https://doi.org/10.1021/es203355v
$\delta^{15}N$	NO_x	Coal Combustion	9.60‰	47.61	-122.33	8 December, 2009	Active sampling	CF-IRMS	Standard Calibration Method	0.2	https://doi.org/10.1021/es203355v
$\delta^{15}N$	NO_x	Coal Combustion	11.70‰	47.61	-122.33	8 December, 2009	Active sampling	CF-IRMS	Standard Calibration Method	0.2	https://doi.org/10.1021/es203355v
$\delta^{15}N$	NO_x	Coal Combustion	10.20‰	47.61	-122.33	8 December, 2009	Active sampling	CF-IRMS	Standard Calibration Method	0.2	https://doi.org/10.1021/es203355v
$\delta^{15}N$	NO_x	Coal Combustion	10.70‰	47.61	-122.33	8 December, 2009	Active sampling	CF-IRMS	Standard Calibration Method	0.2	https://doi.org/10.1021/es203355v
$\delta^{15}N$	NO_x	Coal Combustion	10.00‰	47.61	-122.33	8 December, 2009	Active sampling	CF-IRMS	Standard Calibration Method	0.2	https://doi.org/10.1021/es203355v
$\delta^{15}N$	NO_x	Coal Combustion	9.50‰	47.61	-122.33	8 December, 2009	Active sampling	CF-IRMS	Standard Calibration Method	0.2	https://doi.org/10.1021/es203355v
$\delta^{15}N$	NO_x	Coal Combustion	15.50‰	47.61	-122.33	25-27 January, 2011	Active sampling	CF-IRMS	Standard Calibration Method	0.2	https://doi.org/10.1021/es203355v

$\delta^{15}N$	NO_x	Coal Combustion	25.60‰	47.61	-122.33	25-27 January, 2011	Active sampling	CF-IRMS	Standard Calibration Method	0.2	https://doi.org/10.1021/es203355v
$\delta^{15}N$	NO_x	Coal Combustion	18.40‰	47.61	-122.33	25-27 January, 2011	Active sampling	CF-IRMS	Standard Calibration Method	0.2	https://doi.org/10.1021/es203355v
$\delta^{15}N$	NO_x	Coal Combustion	13.60‰	47.61	-122.33	25-27 January, 2011	Active sampling	CF-IRMS	Standard Calibration Method	0.2	https://doi.org/10.1021/es203355v
$\delta^{15}N$	NO_x	Coal Combustion	13.90‰	47.61	-122.33	25-27 January, 2011	Active sampling	CF-IRMS	Standard Calibration Method	0.2	https://doi.org/10.1021/es203355v
$\delta^{15}N$	NO_x	Coal Combustion	15.10‰	47.61	-122.33	25-27 January, 2011	Active sampling	CF-IRMS	Standard Calibration Method	0.2	https://doi.org/10.1021/es203355v
$\delta^{15}N$	NO_x	Coal Combustion	12.60‰	47.61	-122.33	25-27 January, 2011	Active sampling	CF-IRMS	Standard Calibration Method	0.2	https://doi.org/10.1021/es203355v
$\delta^{15}N$	NO_x	Coal Combustion	12.10‰	47.61	-122.33	25-27 January, 2011	Active sampling	CF-IRMS	Standard Calibration Method	0.2	https://doi.org/10.1021/es203355v
$\delta^{15}N$	NO_x	Coal Combustion	11.80‰	47.61	-122.33	25-27 January, 2011	Active sampling	CF-IRMS	Standard Calibration Method	0.2	https://doi.org/10.1021/es203355v
$\delta^{15}N$	NO_x	Coal Combustion	18.90‰	47.61	-122.33	5-6 April, 2011	Active sampling	CF-IRMS	Standard Calibration Method	0.2	https://doi.org/10.1021/es203355v
$\delta^{15}N$	NO_x	Coal Combustion	19.10‰	47.61	-122.33	5-6 April, 2011	Active sampling	CF-IRMS	Standard Calibration Method	0.2	https://doi.org/10.1021/es203355v
$\delta^{15}N$	NO_x	Coal Combustion	19.20‰	47.61	-122.33	5-6 April, 2011	Active sampling	CF-IRMS	Standard Calibration Method	0.2	https://doi.org/10.1021/es203355v
$\delta^{15}N$	NO_x	Coal Combustion	19.30‰	47.61	-122.33	5-6 April, 2011	Active sampling	CF-IRMS	Standard Calibration Method	0.2	https://doi.org/10.1021/es203355v
$\delta^{15}N$	NO_x	Coal Combustion	19.20‰	47.61	-122.33	5-6 April, 2011	Active sampling	CF-IRMS	Standard Calibration Method	0.2	https://doi.org/10.1021/es203355v
$\delta^{15}N$	NO_x	Coal Combustion	18.70‰	47.61	-122.33	5-6 April, 2011	Active sampling	CF-IRMS	Standard Calibration Method	0.2	https://doi.org/10.1021/es203355v
$\delta^{15}N$	NO_x	Coal Combustion	18.90‰	47.61	-122.33	5-6 April, 2011	Active sampling	CF-IRMS	Standard Calibration Method	0.2	https://doi.org/10.1021/es203355v

$\delta^{15}N$	NO_x	Coal Combustion	20.40‰	47.61	-122.33	5-6 April, 2011	Active sampling	CF-IRMS	Standard Calibration Method	0.2	https://doi.org/10.1021/es203355v
$\delta^{15}N$	NO_x	Coal Combustion	10.30‰	47.61	-122.33	5-6 April, 2011	Active sampling	CF-IRMS	Standard Calibration Method	0.2	https://doi.org/10.1021/es203355v
$\delta^{15}N$	NO_x	Coal Combustion	11.50‰	47.61	-122.33	5-6 April, 2011	Active sampling	CF-IRMS	Standard Calibration Method	0.2	https://doi.org/10.1021/es203355v
$\delta^{15}N$	NO_x	Coal Combustion	11.70‰	47.61	-122.33	5-6 April, 2011	Active sampling	CF-IRMS	Standard Calibration Method	0.2	https://doi.org/10.1021/es203355v
$\delta^{15}N$	NO_x	Coal Combustion	10.80‰	47.61	-122.33	5-6 April, 2011	Active sampling	CF-IRMS	Standard Calibration Method	0.2	https://doi.org/10.1021/es203355v
$\delta^{15}N$	NO_x	Coal Combustion	10.20‰	47.61	-122.33	5-6 April, 2011	Active sampling	CF-IRMS	Standard Calibration Method	0.2	https://doi.org/10.1021/es203355v
$\delta^{15}N$	NO_x	Coal Combustion	9.80‰	47.61	-122.33	5-6 April, 2011	Active sampling	CF-IRMS	Standard Calibration Method	0.2	https://doi.org/10.1021/es203355v
$\delta^{15}N$	NO_x	Coal Combustion	9.90‰	47.61	-122.33	5-6 April, 2011	Active sampling	CF-IRMS	Standard Calibration Method	0.2	https://doi.org/10.1021/es203355v
$\delta^{15}N$	NO_x	Coal Combustion	9.60‰	47.61	-122.33	5-6 April, 2011	Active sampling	CF-IRMS	Standard Calibration Method	0.2	https://doi.org/10.1021/es203355v
$\delta^{15}N$	NO_x	Coal Combustion	13.00‰	-26.51	29.17	April, 1990	Active sampling	IRMS	Standard Calibration Method	1.0	https://doi.org/10.3402/tellusb.v42i3.15223
$\delta^{15}N$	NO_x	Coal Combustion	9.00‰	-26.51	29.17	January, 1990	Active sampling	IRMS	Standard Calibration Method	1.0	https://doi.org/10.3402/tellusb.v42i3.15223
$\delta^{15}N$	NO_x	Coal Combustion	12.00‰	-26.51	29.17	July, 1990	Active sampling	IRMS	Standard Calibration Method	1.0	https://doi.org/10.3402/tellusb.v42i3.15223
$\delta^{15}N$	NO_x	Coal Combustion	8.00‰	-26.51	29.17	October, 1990	Active sampling	IRMS	Standard Calibration Method	1.0	https://doi.org/10.3402/tellusb.v42i3.15223
$\delta^{15}N$	NO_x	Coal Combustion	6.00‰	-26.51	29.17	December, 1990	Active sampling	IRMS	Standard Calibration Method	1.0	https://doi.org/10.3402/tellusb.v42i3.15223
$\delta^{15}N$	NO_x	Coal Combustion	5.20‰	-25.75	28.25	January, 1987	Active sampling	IRMS	Standard Calibration Method	0.2	https://doi.org/10.1016/0004-6981(87)90080-1

δ^{15}N	NO_x	Vehicle Exhausts-Gasoline	-12.20‰	23.15	113.34	16 April-27 April, 2019	Active sampling	IRMS	Standard Calibration Method	0.4	https://doi.org/10.1021/acs.est.0c04749
δ^{15}N	NO_x	Vehicle Exhausts-Gasoline	-9.40‰	23.15	113.34	16 April-27 April, 2019	Active sampling	IRMS	Standard Calibration Method	0.4	https://doi.org/10.1021/acs.est.0c04749
δ^{15}N	NO_x	Vehicle Exhausts-Gasoline	-9.10‰	23.15	113.34	16 April-27 April, 2019	Active sampling	IRMS	Standard Calibration Method	0.4	https://doi.org/10.1021/acs.est.0c04749
δ^{15}N	NO_x	Vehicle Exhausts-Gasoline	-6.50‰	23.15	113.34	16 April-27 April, 2019	Active sampling	IRMS	Standard Calibration Method	0.4	https://doi.org/10.1021/acs.est.0c04749
δ^{15}N	NO_x	Vehicle Exhausts-Gasoline	-6.80‰	23.15	113.34	16 April-27 April, 2019	Active sampling	IRMS	Standard Calibration Method	0.4	https://doi.org/10.1021/acs.est.0c04749
δ^{15}N	NO_x	Vehicle Exhausts-Gasoline	-7.30‰	23.15	113.34	16 April-27 April, 2019	Active sampling	IRMS	Standard Calibration Method	0.4	https://doi.org/10.1021/acs.est.0c04749
δ^{15}N	NO_x	Vehicle Exhausts-Gasoline	-4.20‰	23.15	113.34	16 April-27 April, 2019	Active sampling	IRMS	Standard Calibration Method	0.4	https://doi.org/10.1021/acs.est.0c04749
δ^{15}N	NO_x	Vehicle Exhausts-Gasoline	-9.60‰	23.15	113.34	16 April-27 April, 2019	Active sampling	IRMS	Standard Calibration Method	0.4	https://doi.org/10.1021/acs.est.0c04749
δ^{15}N	NO_x	Vehicle Exhausts-Gasoline	-8.50‰	23.15	113.34	16 April-27 April, 2019	Active sampling	IRMS	Standard Calibration Method	0.4	https://doi.org/10.1021/acs.est.0c04749
δ^{15}N	NO_x	Vehicle Exhausts-Gasoline	-9.30‰	23.15	113.34	16 April-27 April, 2019	Active sampling	IRMS	Standard Calibration Method	0.4	https://doi.org/10.1021/acs.est.0c04749
δ^{15}N	NO_x	Vehicle Exhausts-Gasoline	4.60‰	23.15	113.34	16 April-27 April, 2019	Active sampling	IRMS	Standard Calibration Method	0.4	https://doi.org/10.1021/acs.est.0c04749
δ^{15}N	NO_x	Vehicle Exhausts-Gasoline	-9.70‰	23.15	113.34	16 April-27 April, 2019	Active sampling	IRMS	Standard Calibration Method	0.4	https://doi.org/10.1021/acs.est.0c04749
δ^{15}N	NO_x	Vehicle Exhausts-Gasoline	-7.20‰	23.15	113.34	16 April-27 April, 2019	Active sampling	IRMS	Standard Calibration Method	0.4	https://doi.org/10.1021/acs.est.0c04749
δ^{15}N	NO_x	Vehicle Exhausts-Gasoline	-7.40‰	23.15	113.34	16 April-27 April, 2019	Active sampling	IRMS	Standard Calibration Method	0.4	https://doi.org/10.1021/acs.est.0c04749
δ^{15}N	NO_x	Vehicle Exhausts-Gasoline	-6.30‰	23.15	113.34	16 April-27 April, 2019	Active sampling	IRMS	Standard Calibration Method	0.4	https://doi.org/10.1021/acs.est.0c04749

$\delta^{15}N$	NO_x	Vehicle Exhausts-Gasoline	-11.00‰	23.15	113.34	16 April-27 April, 2019	Active sampling	IRMS	Standard Calibration Method	0.4	https://doi.org/10.1021/acs.est.0c04749
$\delta^{15}N$	NO_x	Vehicle Exhausts-Gasoline	-7.00‰	23.15	113.34	16 April-27 April, 2019	Active sampling	IRMS	Standard Calibration Method	0.4	https://doi.org/10.1021/acs.est.0c04749
$\delta^{15}N$	NO_x	Vehicle Exhausts-Gasoline	-4.90‰	23.15	113.34	16 April-27 April, 2019	Active sampling	IRMS	Standard Calibration Method	0.4	https://doi.org/10.1021/acs.est.0c04749
$\delta^{15}N$	NO_x	Vehicle Exhausts-Gasoline	-6.20‰	23.15	113.34	16 April-27 April, 2019	Active sampling	IRMS	Standard Calibration Method	0.4	https://doi.org/10.1021/acs.est.0c04749
$\delta^{15}N$	NO_x	Vehicle Exhausts-Gasoline	-5.60‰	23.15	113.34	16 April-27 April, 2019	Active sampling	IRMS	Standard Calibration Method	0.4	https://doi.org/10.1021/acs.est.0c04749
$\delta^{15}N$	NO_x	Vehicle Exhausts-Gasoline	-1.20‰	23.15	113.34	16 April-27 April, 2019	Active sampling	IRMS	Standard Calibration Method	0.4	https://doi.org/10.1021/acs.est.0c04749
$\delta^{15}N$	NO_x	Vehicle Exhausts-Gasoline	-14.60‰	23.15	113.34	16 April-27 April, 2019	Active sampling	IRMS	Standard Calibration Method	0.4	https://doi.org/10.1021/acs.est.0c04749
$\delta^{15}N$	NO_x	Vehicle Exhausts-Gasoline	-13.50‰	23.15	113.34	16 April-27 April, 2019	Active sampling	IRMS	Standard Calibration Method	0.4	https://doi.org/10.1021/acs.est.0c04749
$\delta^{15}N$	NO_x	Vehicle Exhausts-Gasoline	-12.70‰	23.15	113.34	16 April-27 April, 2019	Active sampling	IRMS	Standard Calibration Method	0.4	https://doi.org/10.1021/acs.est.0c04749
$\delta^{15}N$	NO_x	Vehicle Exhausts-Gasoline	-10.40‰	23.15	113.34	16 April-27 April, 2019	Active sampling	IRMS	Standard Calibration Method	0.4	https://doi.org/10.1021/acs.est.0c04749
$\delta^{15}N$	NO_x	Vehicle Exhausts-Gasoline	-7.60‰	23.15	113.34	16 April-27 April, 2019	Active sampling	IRMS	Standard Calibration Method	0.4	https://doi.org/10.1021/acs.est.0c04749
$\delta^{15}N$	NO_x	Vehicle Exhausts-Gasoline	-8.30‰	23.15	113.34	16 April-27 April, 2019	Active sampling	IRMS	Standard Calibration Method	0.4	https://doi.org/10.1021/acs.est.0c04749
$\delta^{15}N$	NO_x	Vehicle Exhausts-Gasoline	-8.40‰	23.15	113.34	16 April-27 April, 2019	Active sampling	IRMS	Standard Calibration Method	0.4	https://doi.org/10.1021/acs.est.0c04749
$\delta^{15}N$	NO_x	Vehicle Exhausts-Gasoline	-4.60‰	23.15	113.34	16 April-27 April, 2019	Active sampling	IRMS	Standard Calibration Method	0.4	https://doi.org/10.1021/acs.est.0c04749
$\delta^{15}N$	NO_x	Vehicle Exhausts-Gasoline	5.00‰	23.15	113.34	16 April-27 April, 2019	Active sampling	IRMS	Standard Calibration Method	0.4	https://doi.org/10.1021/acs.est.0c04749

$\delta^{15}N$	NO_x	Vehicle Exhausts-Gasoline	-10.40‰	23.15	113.34	16 April-27 April, 2019	Active sampling	IRMS	Standard Calibration Method	0.4	https://doi.org/10.1021/acs.est.0c04749
$\delta^{15}N$	NO_x	Vehicle Exhausts-Gasoline	-4.50‰	23.15	113.34	16 April-27 April, 2019	Active sampling	IRMS	Standard Calibration Method	0.4	https://doi.org/10.1021/acs.est.0c04749
$\delta^{15}N$	NO_x	Vehicle Exhausts-Gasoline	6.40‰	23.15	113.34	16 April-27 April, 2019	Active sampling	IRMS	Standard Calibration Method	0.4	https://doi.org/10.1021/acs.est.0c04749
$\delta^{15}N$	NO_x	Vehicle Exhausts-Gasoline	-2.30‰	23.15	113.34	16 April-27 April, 2019	Active sampling	IRMS	Standard Calibration Method	0.4	https://doi.org/10.1021/acs.est.0c04749
$\delta^{15}N$	NO_x	Vehicle Exhausts-Gasoline	-10.50‰	23.15	113.34	16 April-27 April, 2019	Active sampling	IRMS	Standard Calibration Method	0.4	https://doi.org/10.1021/acs.est.0c04749
$\delta^{15}N$	NO_x	Vehicle Exhausts-Gasoline	-13.60‰	23.15	113.34	16 April-27 April, 2019	Active sampling	IRMS	Standard Calibration Method	0.4	https://doi.org/10.1021/acs.est.0c04749
$\delta^{15}N$	NO_x	Vehicle Exhausts-Gasoline	-11.30‰	23.15	113.34	16 April-27 April, 2019	Active sampling	IRMS	Standard Calibration Method	0.4	https://doi.org/10.1021/acs.est.0c04749
$\delta^{15}N$	NO_x	Vehicle Exhausts-Gasoline	-11.80‰	23.15	113.34	16 April-27 April, 2019	Active sampling	IRMS	Standard Calibration Method	0.4	https://doi.org/10.1021/acs.est.0c04749
$\delta^{15}N$	NO_x	Vehicle Exhausts-Gasoline	-10.90‰	23.15	113.34	16 April-27 April, 2019	Active sampling	IRMS	Standard Calibration Method	0.4	https://doi.org/10.1021/acs.est.0c04749
$\delta^{15}N$	NO_x	Vehicle Exhausts-Gasoline	-11.90‰	23.15	113.34	16 April-27 April, 2019	Active sampling	IRMS	Standard Calibration Method	0.4	https://doi.org/10.1021/acs.est.0c04749
$\delta^{15}N$	NO_x	Vehicle Exhausts-Gasoline	-13.40‰	23.15	113.34	16 April-27 April, 2019	Active sampling	IRMS	Standard Calibration Method	0.4	https://doi.org/10.1021/acs.est.0c04749
$\delta^{15}N$	NO_x	Vehicle Exhausts-Gasoline	-7.10‰	23.15	113.34	16 April-27 April, 2019	Active sampling	IRMS	Standard Calibration Method	0.4	https://doi.org/10.1021/acs.est.0c04749
$\delta^{15}N$	NO_x	Vehicle Exhausts-Gasoline	-12.20‰	23.15	113.34	16 April-27 April, 2019	Active sampling	IRMS	Standard Calibration Method	0.4	https://doi.org/10.1021/acs.est.0c04749
$\delta^{15}N$	NO_x	Vehicle Exhausts-Gasoline	-13.20‰	39.80	-83.59	1 October, 2014-1 May, 2015	Active sampling	CF-IRMS	Standard Calibration Method	0.3	https://doi.org/10.1021/acs.est.5b02769
$\delta^{15}N$	NO_x	Vehicle Exhausts-Gasoline	-14.80‰	39.80	-83.59	1 October, 2014-1 May, 2015	Active sampling	CF-IRMS	Standard Calibration Method	0.3	https://doi.org/10.1021/acs.est.5b02769

$\delta^{15}N$	NO_x	Vehicle Exhausts-Gasoline	-12.80‰	39.80	-83.59	1 October, 2014-1 May, 2015	Active sampling	CF-IRMS	Standard Calibration Method	0.3	https://doi.org/10.1021/acs.est.5b02769
$\delta^{15}N$	NO_x	Vehicle Exhausts-Gasoline	-13.80‰	39.80	-83.59	1 October, 2014-1 May, 2015	Active sampling	CF-IRMS	Standard Calibration Method	0.3	https://doi.org/10.1021/acs.est.5b02769
$\delta^{15}N$	NO_x	Vehicle Exhausts-Gasoline	-12.70‰	39.80	-83.59	1 October, 2014-1 May, 2015	Active sampling	CF-IRMS	Standard Calibration Method	0.3	https://doi.org/10.1021/acs.est.5b02769
$\delta^{15}N$	NO_x	Vehicle Exhausts-Gasoline	-14.90‰	39.80	-83.59	1 October, 2014-1 May, 2015	Active sampling	CF-IRMS	Standard Calibration Method	0.3	https://doi.org/10.1021/acs.est.5b02769
$\delta^{15}N$	NO_x	Vehicle Exhausts-Gasoline	-13.60‰	39.80	-83.59	1 October, 2014-1 May, 2015	Active sampling	CF-IRMS	Standard Calibration Method	0.3	https://doi.org/10.1021/acs.est.5b02769
$\delta^{15}N$	NO_x	Vehicle Exhausts-Gasoline	-13.40‰	39.80	-83.59	1 October, 2014-1 May, 2015	Active sampling	CF-IRMS	Standard Calibration Method	0.3	https://doi.org/10.1021/acs.est.5b02769
$\delta^{15}N$	NO_x	Vehicle Exhausts-Gasoline	-13.00‰	39.80	-83.59	1 October, 2014-1 May, 2015	Active sampling	CF-IRMS	Standard Calibration Method	0.3	https://doi.org/10.1021/acs.est.5b02769
$\delta^{15}N$	NO_x	Vehicle Exhausts-Gasoline	-12.90‰	39.80	-83.59	1 October, 2014-1 May, 2015	Active sampling	CF-IRMS	Standard Calibration Method	0.3	https://doi.org/10.1021/acs.est.5b02769
$\delta^{15}N$	NO_x	Vehicle Exhausts-Gasoline	-14.10‰	39.80	-83.59	1 October, 2014-1 May, 2015	Active sampling	CF-IRMS	Standard Calibration Method	0.3	https://doi.org/10.1021/acs.est.5b02769
$\delta^{15}N$	NO_x	Vehicle Exhausts-Gasoline	-9.00‰	39.80	-83.59	1 October, 2014-1 May, 2015	Active sampling	CF-IRMS	Standard Calibration Method	0.3	https://doi.org/10.1021/acs.est.5b02769
$\delta^{15}N$	NO_x	Vehicle Exhausts-Gasoline	-8.50‰	39.80	-83.59	1 October, 2014-1 May, 2015	Active sampling	CF-IRMS	Standard Calibration Method	0.3	https://doi.org/10.1021/acs.est.5b02769
$\delta^{15}N$	NO_x	Vehicle Exhausts-Gasoline	-8.50‰	39.80	-83.59	1 October, 2014-1 May, 2015	Active sampling	CF-IRMS	Standard Calibration Method	0.3	https://doi.org/10.1021/acs.est.5b02769
$\delta^{15}N$	NO_x	Vehicle Exhausts-Gasoline	-10.40‰	39.80	-83.59	1 October, 2014-1 May, 2015	Active sampling	CF-IRMS	Standard Calibration Method	0.3	https://doi.org/10.1021/acs.est.5b02769
$\delta^{15}N$	NO_x	Vehicle Exhausts-Gasoline	-10.90‰	39.80	-83.59	1 October, 2014-1 May, 2015	Active sampling	CF-IRMS	Standard Calibration Method	0.3	https://doi.org/10.1021/acs.est.5b02769
$\delta^{15}N$	NO_x	Vehicle Exhausts-Gasoline	-6.20‰	39.80	-83.59	1 October, 2014-1 May, 2015	Active sampling	CF-IRMS	Standard Calibration Method	0.3	https://doi.org/10.1021/acs.est.5b02769

$\delta^{15}N$	NO_x	Vehicle Exhausts-Gasoline	-7.30‰	39.80	-83.59	1 October, 2014-1 May, 2015	Active sampling	CF-IRMS	Standard Calibration Method	0.3	https://doi.org/10.1021/acs.est.5b02769
$\delta^{15}N$	NO_x	Vehicle Exhausts-Gasoline	-7.50‰	39.80	-83.59	1 October, 2014-1 May, 2015	Active sampling	CF-IRMS	Standard Calibration Method	0.3	https://doi.org/10.1021/acs.est.5b02769
$\delta^{15}N$	NO_x	Vehicle Exhausts-Gasoline	-7.70‰	39.80	-83.59	1 October, 2014-1 May, 2015	Active sampling	CF-IRMS	Standard Calibration Method	0.3	https://doi.org/10.1021/acs.est.5b02769
$\delta^{15}N$	NO_x	Vehicle Exhausts-Gasoline	-9.00‰	39.80	-83.59	1 October, 2014-1 May, 2015	Active sampling	CF-IRMS	Standard Calibration Method	0.3	https://doi.org/10.1021/acs.est.5b02769
$\delta^{15}N$	NO_x	Vehicle Exhausts-Gasoline	-14.20‰	39.80	-83.59	1 October, 2014-1 May, 2015	Active sampling	CF-IRMS	Standard Calibration Method	0.3	https://doi.org/10.1021/acs.est.5b02769
$\delta^{15}N$	NO_x	Vehicle Exhausts-Gasoline	-15.60‰	39.80	-83.59	1 October, 2014-1 May, 2015	Active sampling	CF-IRMS	Standard Calibration Method	0.3	https://doi.org/10.1021/acs.est.5b02769
$\delta^{15}N$	NO_x	Vehicle Exhausts-Gasoline	-14.30‰	39.80	-83.59	1 October, 2014-1 May, 2015	Active sampling	CF-IRMS	Standard Calibration Method	0.3	https://doi.org/10.1021/acs.est.5b02769
$\delta^{15}N$	NO_x	Vehicle Exhausts-Gasoline	-14.80‰	39.80	-83.59	1 October, 2014-1 May, 2015	Active sampling	CF-IRMS	Standard Calibration Method	0.3	https://doi.org/10.1021/acs.est.5b02769
$\delta^{15}N$	NO_x	Vehicle Exhausts-Gasoline	-14.20‰	39.80	-83.59	1 October, 2014-1 May, 2015	Active sampling	CF-IRMS	Standard Calibration Method	0.3	https://doi.org/10.1021/acs.est.5b02769
$\delta^{15}N$	NO_x	Vehicle Exhausts-Gasoline	-14.00‰	39.80	-83.59	1 October, 2014-1 May, 2015	Active sampling	CF-IRMS	Standard Calibration Method	0.3	https://doi.org/10.1021/acs.est.5b02769
$\delta^{15}N$	NO_x	Vehicle Exhausts-Gasoline	-13.20‰	39.80	-83.59	1 October, 2014-1 May, 2015	Active sampling	CF-IRMS	Standard Calibration Method	0.3	https://doi.org/10.1021/acs.est.5b02769
$\delta^{15}N$	NO_x	Vehicle Exhausts-Gasoline	-12.60‰	39.80	-83.59	1 October, 2014-1 May, 2015	Active sampling	CF-IRMS	Standard Calibration Method	0.3	https://doi.org/10.1021/acs.est.5b02769
$\delta^{15}N$	NO_x	Vehicle Exhausts-Gasoline	-8.40‰	39.80	-83.59	1 October, 2014-1 May, 2015	Active sampling	CF-IRMS	Standard Calibration Method	0.3	https://doi.org/10.1021/acs.est.5b02769
$\delta^{15}N$	NO_x	Vehicle Exhausts-Gasoline	-7.40‰	39.80	-83.59	1 October, 2014-1 May, 2015	Active sampling	CF-IRMS	Standard Calibration Method	0.3	https://doi.org/10.1021/acs.est.5b02769
$\delta^{15}N$	NO_x	Vehicle Exhausts-Gasoline	-9.20‰	39.80	-83.59	1 October, 2014-1 May, 2015	Active sampling	CF-IRMS	Standard Calibration Method	0.3	https://doi.org/10.1021/acs.est.5b02769

$\delta^{15}N$	NO_x	Vehicle Exhausts-Gasoline	-10.70‰	39.80	-83.59	1 October, 2014-1 May, 2015	Active sampling	CF-IRMS	Standard Calibration Method	0.3	https://doi.org/10.1021/acs.est.5b02769
$\delta^{15}N$	NO_x	Vehicle Exhausts-Gasoline	-9.70‰	39.80	-83.59	1 October, 2014-1 May, 2015	Active sampling	CF-IRMS	Standard Calibration Method	0.3	https://doi.org/10.1021/acs.est.5b02769
$\delta^{15}N$	NO_x	Vehicle Exhausts-Gasoline	-10.80‰	39.80	-83.59	1 October, 2014-1 May, 2015	Active sampling	CF-IRMS	Standard Calibration Method	0.3	https://doi.org/10.1021/acs.est.5b02769
$\delta^{15}N$	NO_x	Vehicle Exhausts-Gasoline	-11.10‰	39.80	-83.59	1 October, 2014-1 May, 2015	Active sampling	CF-IRMS	Standard Calibration Method	0.3	https://doi.org/10.1021/acs.est.5b02769
$\delta^{15}N$	NO_x	Vehicle Exhausts-Gasoline	-15.60‰	39.80	-83.59	1 October, 2014-1 May, 2015	Active sampling	CF-IRMS	Standard Calibration Method	0.3	https://doi.org/10.1021/acs.est.5b02769
$\delta^{15}N$	NO_x	Vehicle Exhausts-Gasoline	-14.30‰	39.80	-83.59	1 October, 2014-1 May, 2015	Active sampling	CF-IRMS	Standard Calibration Method	0.3	https://doi.org/10.1021/acs.est.5b02769
$\delta^{15}N$	NO_x	Vehicle Exhausts-Gasoline	-15.50‰	39.80	-83.59	1 October, 2014-1 May, 2015	Active sampling	CF-IRMS	Standard Calibration Method	0.3	https://doi.org/10.1021/acs.est.5b02769
$\delta^{15}N$	NO_x	Vehicle Exhausts-Gasoline	-12.80‰	39.80	-83.59	1 October, 2014-1 May, 2015	Active sampling	CF-IRMS	Standard Calibration Method	0.3	https://doi.org/10.1021/acs.est.5b02769
$\delta^{15}N$	NO_x	Vehicle Exhausts-Gasoline	-11.60‰	39.80	-83.59	1 October, 2014-1 May, 2015	Active sampling	CF-IRMS	Standard Calibration Method	0.3	https://doi.org/10.1021/acs.est.5b02769
$\delta^{15}N$	NO_x	Vehicle Exhausts-Gasoline	-10.60‰	39.80	-83.59	1 October, 2014-1 May, 2015	Active sampling	CF-IRMS	Standard Calibration Method	0.3	https://doi.org/10.1021/acs.est.5b02769
$\delta^{15}N$	NO_x	Vehicle Exhausts-Gasoline	-9.70‰	39.80	-83.59	1 October, 2014-1 May, 2015	Active sampling	CF-IRMS	Standard Calibration Method	0.3	https://doi.org/10.1021/acs.est.5b02769
$\delta^{15}N$	NO_x	Vehicle Exhausts-Gasoline	-8.80‰	39.80	-83.59	1 October, 2014-1 May, 2015	Active sampling	CF-IRMS	Standard Calibration Method	0.3	https://doi.org/10.1021/acs.est.5b02769
$\delta^{15}N$	NO_x	Vehicle Exhausts-Gasoline	-8.80‰	39.80	-83.59	1 October, 2014-1 May, 2015	Active sampling	CF-IRMS	Standard Calibration Method	0.3	https://doi.org/10.1021/acs.est.5b02769
$\delta^{15}N$	NO_x	Vehicle Exhausts-Gasoline	-8.20‰	39.80	-83.59	1 October, 2014-1 May, 2015	Active sampling	CF-IRMS	Standard Calibration Method	0.3	https://doi.org/10.1021/acs.est.5b02769
$\delta^{15}N$	NO_x	Vehicle Exhausts-Gasoline	-8.10‰	40.45	-86.91	20 June-26 September, 2014	Active sampling	CF-IRMS	Standard Calibration Method	0.3	https://doi.org/10.1021/es505580v

$\delta^{15}N$	NO_x	Vehicle Exhausts-Gasoline	-9.50‰	40.45	-86.91	20 June-26 September, 2014	Active sampling	CF-IRMS	Standard Calibration Method	0.3	https://doi.org/10.1021/es505580v
$\delta^{15}N$	NO_x	Vehicle Exhausts-Gasoline	-10.30‰	40.45	-86.91	20 June-26 September, 2014	Active sampling	CF-IRMS	Standard Calibration Method	0.3	https://doi.org/10.1021/es505580v
$\delta^{15}N$	NO_x	Vehicle Exhausts-Gasoline	-0.80‰	40.45	-86.91	20 June-26 September, 2014	Active sampling	CF-IRMS	Standard Calibration Method	0.3	https://doi.org/10.1021/es505580v
$\delta^{15}N$	NO_x	Vehicle Exhausts-Gasoline	0.30‰	40.45	-86.91	20 June-26 September, 2014	Active sampling	CF-IRMS	Standard Calibration Method	0.3	https://doi.org/10.1021/es505580v
$\delta^{15}N$	NO_x	Vehicle Exhausts-Gasoline	2.00‰	40.45	-86.91	20 June-26 September, 2014	Active sampling	CF-IRMS	Standard Calibration Method	0.3	https://doi.org/10.1021/es505580v
$\delta^{15}N$	NO_x	Vehicle Exhausts-Gasoline	-0.40‰	40.45	-86.91	20 June-26 September, 2014	Active sampling	CF-IRMS	Standard Calibration Method	0.3	https://doi.org/10.1021/es505580v
$\delta^{15}N$	NO_x	Vehicle Exhausts-Gasoline	-2.90‰	40.45	-86.91	20 June-26 September, 2014	Active sampling	CF-IRMS	Standard Calibration Method	0.3	https://doi.org/10.1021/es505580v
$\delta^{15}N$	NO_x	Vehicle Exhausts-Gasoline	-5.30‰	40.45	-86.91	20 June-26 September, 2014	Active sampling	CF-IRMS	Standard Calibration Method	0.3	https://doi.org/10.1021/es505580v
$\delta^{15}N$	NO_x	Vehicle Exhausts-Gasoline	-3.60‰	40.45	-86.91	20 June-26 September, 2014	Active sampling	CF-IRMS	Standard Calibration Method	0.3	https://doi.org/10.1021/es505580v
$\delta^{15}N$	NO_x	Vehicle Exhausts-Gasoline	-8.30‰	40.45	-86.91	20 June-26 September, 2014	Active sampling	CF-IRMS	Standard Calibration Method	0.3	https://doi.org/10.1021/es505580v
$\delta^{15}N$	NO_x	Vehicle Exhausts-Gasoline	-0.40‰	40.45	-86.91	20 June-26 September, 2014	Active sampling	CF-IRMS	Standard Calibration Method	0.3	https://doi.org/10.1021/es505580v
$\delta^{15}N$	NO_x	Vehicle Exhausts-Gasoline	0.60‰	40.45	-86.91	20 June-26 September, 2014	Active sampling	CF-IRMS	Standard Calibration Method	0.3	https://doi.org/10.1021/es505580v
$\delta^{15}N$	NO_x	Vehicle Exhausts-Gasoline	8.60‰	40.45	-86.91	20 June-26 September, 2014	Active sampling	CF-IRMS	Standard Calibration Method	0.3	https://doi.org/10.1021/es505580v
$\delta^{15}N$	NO_x	Vehicle Exhausts-Gasoline	-4.60‰	40.45	-86.91	20 June-26 September, 2014	Active sampling	CF-IRMS	Standard Calibration Method	0.3	https://doi.org/10.1021/es505580v
$\delta^{15}N$	NO_x	Vehicle Exhausts-Gasoline	1.60‰	40.45	-86.91	20 June-26 September, 2014	Active sampling	CF-IRMS	Standard Calibration Method	0.3	https://doi.org/10.1021/es505580v

$\delta^{15}N$	NO_x	Vehicle Exhausts-Gasoline	3.40‰	40.45	-86.91	20 June-26 September, 2014	Active sampling	CF-IRMS	Standard Calibration Method	0.3	https://doi.org/10.1021/es505580v
$\delta^{15}N$	NO_x	Vehicle Exhausts-Gasoline	-9.60‰	40.45	-86.91	20 June-26 September, 2014	Active sampling	CF-IRMS	Standard Calibration Method	0.3	https://doi.org/10.1021/es505580v
$\delta^{15}N$	NO_x	Vehicle Exhausts-Gasoline	-9.00‰	40.45	-86.91	20 June-26 September, 2014	Active sampling	CF-IRMS	Standard Calibration Method	0.3	https://doi.org/10.1021/es505580v
$\delta^{15}N$	NO_x	Vehicle Exhausts-Gasoline	-4.40‰	40.45	-86.91	20 June-26 September, 2014	Active sampling	CF-IRMS	Standard Calibration Method	0.3	https://doi.org/10.1021/es505580v
$\delta^{15}N$	NO_x	Vehicle Exhausts-Gasoline	2.40‰	40.45	-86.91	20 June-26 September, 2014	Active sampling	CF-IRMS	Standard Calibration Method	0.3	https://doi.org/10.1021/es505580v
$\delta^{15}N$	NO_x	Vehicle Exhausts-Gasoline	-12.20‰	40.45	-86.91	20 June-26 September, 2014	Active sampling	CF-IRMS	Standard Calibration Method	0.3	https://doi.org/10.1021/es505580v
$\delta^{15}N$	NO_x	Vehicle Exhausts-Gasoline	9.80‰	40.45	-86.91	20 June-26 September, 2014	Active sampling	CF-IRMS	Standard Calibration Method	0.3	https://doi.org/10.1021/es505580v
$\delta^{15}N$	NO_x	Vehicle Exhausts-Gasoline	-5.50‰	40.45	-86.91	20 June-26 September, 2014	Active sampling	CF-IRMS	Standard Calibration Method	0.3	https://doi.org/10.1021/es505580v
$\delta^{15}N$	NO_x	Vehicle Exhausts-Gasoline	1.60‰	40.45	-86.91	20 June-26 September, 2014	Active sampling	CF-IRMS	Standard Calibration Method	0.3	https://doi.org/10.1021/es505580v
$\delta^{15}N$	NO_x	Vehicle Exhausts-Gasoline	3.84‰	25.79	-80.23	January, 1977	Active sampling	IRMS	Standard Calibration Method	0.2	https://doi.org/10.1016/0004-6981(77)90102-0
$\delta^{15}N$	NO_x	Vehicle Exhausts-Gasoline	3.38‰	25.79	-80.23	December, 1977	Active sampling	IRMS	Standard Calibration Method	0.2	https://doi.org/10.1016/0004-6981(77)90102-0
$\delta^{15}N$	NO_x	Vehicle Exhausts-Diesel	-17.20‰	23.15	113.34	16 April-27 April, 2019	Active sampling	IRMS	Standard Calibration Method	0.4	https://doi.org/10.1021/acs.est.0c04749
$\delta^{15}N$	NO_x	Vehicle Exhausts-Diesel	-18.80‰	23.15	113.34	16 April-27 April, 2019	Active sampling	IRMS	Standard Calibration Method	0.4	https://doi.org/10.1021/acs.est.0c04749
$\delta^{15}N$	NO_x	Vehicle Exhausts-Diesel	-16.30‰	23.15	113.34	16 April-27 April, 2019	Active sampling	IRMS	Standard Calibration Method	0.4	https://doi.org/10.1021/acs.est.0c04749
$\delta^{15}N$	NO_x	Vehicle Exhausts-Diesel	-16.00‰	23.15	113.34	16 April-27 April, 2019	Active sampling	IRMS	Standard Calibration Method	0.4	https://doi.org/10.1021/acs.est.0c04749

$\delta^{15}N$	NO_x	Vehicle Exhausts-Diesel	-17.80‰	23.15	113.34	16 April-27 April, 2019	Active sampling	IRMS	Standard Calibration Method	0.4	https://doi.org/10.1021/acs.est.0c04749
$\delta^{15}N$	NO_x	Vehicle Exhausts-Diesel	-7.30‰	23.15	113.34	16 April-27 April, 2019	Active sampling	IRMS	Standard Calibration Method	0.4	https://doi.org/10.1021/acs.est.0c04749
$\delta^{15}N$	NO_x	Vehicle Exhausts-Diesel	-11.30‰	23.15	113.34	16 April-27 April, 2019	Active sampling	IRMS	Standard Calibration Method	0.4	https://doi.org/10.1021/acs.est.0c04749
$\delta^{15}N$	NO_x	Vehicle Exhausts-Diesel	-13.80‰	23.15	113.34	16 April-27 April, 2019	Active sampling	IRMS	Standard Calibration Method	0.4	https://doi.org/10.1021/acs.est.0c04749
$\delta^{15}N$	NO_x	Vehicle Exhausts-Diesel	-5.80‰	23.15	113.34	16 April-27 April, 2019	Active sampling	IRMS	Standard Calibration Method	0.4	https://doi.org/10.1021/acs.est.0c04749
$\delta^{15}N$	NO_x	Vehicle Exhausts-Diesel	-10.60‰	23.15	113.34	16 April-27 April, 2019	Active sampling	IRMS	Standard Calibration Method	0.4	https://doi.org/10.1021/acs.est.0c04749
$\delta^{15}N$	NO_x	Vehicle Exhausts-Diesel	-10.20‰	23.15	113.34	16 April-27 April, 2019	Active sampling	IRMS	Standard Calibration Method	0.4	https://doi.org/10.1021/acs.est.0c04749
$\delta^{15}N$	NO_x	Vehicle Exhausts-Diesel	-10.90‰	23.15	113.34	16 April-27 April, 2019	Active sampling	IRMS	Standard Calibration Method	0.4	https://doi.org/10.1021/acs.est.0c04749
$\delta^{15}N$	NO_x	Vehicle Exhausts-Diesel	-11.00‰	23.15	113.34	16 April-27 April, 2019	Active sampling	IRMS	Standard Calibration Method	0.4	https://doi.org/10.1021/acs.est.0c04749
$\delta^{15}N$	NO_x	Vehicle Exhausts-Diesel	-15.90‰	23.15	113.34	16 April-27 April, 2019	Active sampling	IRMS	Standard Calibration Method	0.4	https://doi.org/10.1021/acs.est.0c04749
$\delta^{15}N$	NO_x	Vehicle Exhausts-Diesel	-12.40‰	23.15	113.34	16 April-27 April, 2019	Active sampling	IRMS	Standard Calibration Method	0.4	https://doi.org/10.1021/acs.est.0c04749
$\delta^{15}N$	NO_x	Vehicle Exhausts-Diesel	-12.40‰	39.80	-83.59	1 October, 2014-1 May, 2015	Active sampling	CF-IRMS	Standard Calibration Method	0.3	https://doi.org/10.1021/acs.est.5b02769
$\delta^{15}N$	NO_x	Vehicle Exhausts-Diesel	-19.20‰	39.80	-83.59	1 October, 2014-1 May, 2015	Active sampling	CF-IRMS	Standard Calibration Method	0.3	https://doi.org/10.1021/acs.est.5b02769
$\delta^{15}N$	NO_x	Vehicle Exhausts-Diesel	-23.90‰	39.80	-83.59	1 October, 2014-1 May, 2015	Active sampling	CF-IRMS	Standard Calibration Method	0.3	https://doi.org/10.1021/acs.est.5b02769
$\delta^{15}N$	NO_x	Vehicle Exhausts-Diesel	-28.10‰	39.80	-83.59	1 October, 2014-1 May, 2015	Active sampling	CF-IRMS	Standard Calibration Method	0.3	https://doi.org/10.1021/acs.est.5b02769

$\delta^{15}N$	NO_x	Vehicle Exhausts-Diesel	-15.90‰	39.80	-83.59	1 October, 2014-1 May, 2015	Active sampling	CF-IRMS	Standard Calibration Method	0.3	https://doi.org/10.1021/acs.est.5b02769
$\delta^{15}N$	NO_x	Vehicle Exhausts-Diesel	-15.00‰	39.80	-83.59	1 October, 2014-1 May, 2015	Active sampling	CF-IRMS	Standard Calibration Method	0.3	https://doi.org/10.1021/acs.est.5b02769
$\delta^{15}N$	NO_x	Vehicle Exhausts-Diesel	-13.40‰	39.80	-83.59	1 October, 2014-1 May, 2015	Active sampling	CF-IRMS	Standard Calibration Method	0.3	https://doi.org/10.1021/acs.est.5b02769
$\delta^{15}N$	NO_x	Vehicle Exhausts-Diesel	-18.30‰	39.80	-83.59	1 October, 2014-1 May, 2015	Active sampling	CF-IRMS	Standard Calibration Method	0.3	https://doi.org/10.1021/acs.est.5b02769
$\delta^{15}N$	NO_x	Vehicle Exhausts-Diesel	-5.80‰	39.80	-83.59	1 October, 2014-1 May, 2015	Active sampling	CF-IRMS	Standard Calibration Method	0.3	https://doi.org/10.1021/acs.est.5b02769
$\delta^{15}N$	NO_x	Vehicle Exhausts-Diesel	0.30‰	39.80	-83.59	1 October, 2014-1 May, 2015	Active sampling	CF-IRMS	Standard Calibration Method	0.3	https://doi.org/10.1021/acs.est.5b02769
$\delta^{15}N$	NO_x	Vehicle Exhausts-Diesel	-1.00‰	39.80	-83.59	1 October, 2014-1 May, 2015	Active sampling	CF-IRMS	Standard Calibration Method	0.3	https://doi.org/10.1021/acs.est.5b02769
$\delta^{15}N$	NO_x	Vehicle Exhausts-Diesel	-0.10‰	39.80	-83.59	1 October, 2014-1 May, 2015	Active sampling	CF-IRMS	Standard Calibration Method	0.3	https://doi.org/10.1021/acs.est.5b02769
$\delta^{15}N$	NO_x	Vehicle Exhausts-Diesel	-19.50‰	39.80	-83.59	1 October, 2014-1 May, 2015	Active sampling	CF-IRMS	Standard Calibration Method	0.3	https://doi.org/10.1021/acs.est.5b02769
$\delta^{15}N$	NO_x	Vehicle Exhausts-Diesel	-20.30‰	39.80	-83.59	1 October, 2014-1 May, 2015	Active sampling	CF-IRMS	Standard Calibration Method	0.3	https://doi.org/10.1021/acs.est.5b02769
$\delta^{15}N$	NO_x	Vehicle Exhausts-Diesel	-17.70‰	39.80	-83.59	1 October, 2014-1 May, 2015	Active sampling	CF-IRMS	Standard Calibration Method	0.3	https://doi.org/10.1021/acs.est.5b02769
$\delta^{15}N$	NO_x	Vehicle Exhausts-Diesel	-16.80‰	39.80	-83.59	1 October, 2014-1 May, 2015	Active sampling	CF-IRMS	Standard Calibration Method	0.3	https://doi.org/10.1021/acs.est.5b02769
$\delta^{15}N$	NO_x	Vehicle Exhausts-Diesel	-21.10‰	39.80	-83.59	1 October, 2014-1 May, 2015	Active sampling	CF-IRMS	Standard Calibration Method	0.3	https://doi.org/10.1021/acs.est.5b02769
$\delta^{15}N$	NO_x	Vehicle Exhausts-Diesel	-7.20‰	39.80	-83.59	1 October, 2014-1 May, 2015	Active sampling	CF-IRMS	Standard Calibration Method	0.3	https://doi.org/10.1021/acs.est.5b02769
$\delta^{15}N$	NO_x	Vehicle Exhausts-Diesel	-4.80‰	39.80	-83.59	1 October, 2014-1 May, 2015	Active sampling	CF-IRMS	Standard Calibration Method	0.3	https://doi.org/10.1021/acs.est.5b02769

$\delta^{15}N$	NO_x	Vehicle Exhausts-Diesel	-7.70‰	39.80	-83.59	1 October, 2014-1 May, 2015	Active sampling	CF-IRMS	Standard Calibration Method	0.3	https://doi.org/10.1021/acs.est.5b02769
$\delta^{15}N$	NO_x	Vehicle Exhausts-Diesel	-9.00‰	39.80	-83.59	1 October, 2014-1 May, 2015	Active sampling	CF-IRMS	Standard Calibration Method	0.3	https://doi.org/10.1021/acs.est.5b02769
$\delta^{15}N$	NO_x	Vehicle Exhausts-Diesel	8.20‰	39.80	-83.59	1 October, 2014-1 May, 2015	Active sampling	CF-IRMS	Standard Calibration Method	0.3	https://doi.org/10.1021/acs.est.5b02769
$\delta^{15}N$	NO_x	Vehicle Exhausts-Diesel	8.50‰	39.80	-83.59	1 October, 2014-1 May, 2015	Active sampling	CF-IRMS	Standard Calibration Method	0.3	https://doi.org/10.1021/acs.est.5b02769
$\delta^{15}N$	NO_x	Vehicle Exhausts-Diesel	-12.90‰	39.80	-83.59	1 October, 2014-1 May, 2015	Active sampling	CF-IRMS	Standard Calibration Method	0.3	https://doi.org/10.1021/acs.est.5b02769
$\delta^{15}N$	NO_x	Vehicle Exhausts-Diesel	-13.50‰	39.80	-83.59	1 October, 2014-1 May, 2015	Active sampling	CF-IRMS	Standard Calibration Method	0.3	https://doi.org/10.1021/acs.est.5b02769
$\delta^{15}N$	NO_x	Vehicle Exhausts-Diesel	-9.90‰	39.80	-83.59	1 October, 2014-1 May, 2015	Active sampling	CF-IRMS	Standard Calibration Method	0.3	https://doi.org/10.1021/acs.est.5b02769
$\delta^{15}N$	NO_x	Vehicle Exhausts-Diesel	-7.40‰	39.80	-83.59	1 October, 2014-1 May, 2015	Active sampling	CF-IRMS	Standard Calibration Method	0.3	https://doi.org/10.1021/acs.est.5b02769
$\delta^{15}N$	NO_x	Vehicle Exhausts-Diesel	-19.10‰	40.45	-86.91	20 June-26 September, 2014	Active sampling	CF-IRMS	Standard Calibration Method	0.3	https://doi.org/10.1021/es505580v
$\delta^{15}N$	NO_x	Vehicle Exhausts-Diesel	-1.60‰	-25.75	28.25	April, 1987	Active sampling	IRMS	Standard Calibration Method	0.2	https://doi.org/10.1016/0004-6981(87)90080-1
$\delta^{15}N$	NO_x	Vehicle Exhausts-Diesel	-13.20‰	-25.75	28.25	July, 1987	Active sampling	IRMS	Standard Calibration Method	0.2	https://doi.org/10.1016/0004-6981(87)90080-1
$\delta^{15}N$	NO_x	Vehicle Exhausts-Diesel	-11.50‰	-25.75	28.25	December, 1987	Active sampling	IRMS	Standard Calibration Method	0.2	https://doi.org/10.1016/0004-6981(87)90080-1
$\delta^{15}N$	NO_x	Microbial Processes	-48.50‰	23.12	113.28	April, 2008	Active sampling	EA-IRMS	Standard Calibration Method	0.2	https://doi.org/10.1016/j.atmosenv.2008.01.042
$\delta^{15}N$	NO_x	Microbial Processes	-37.23‰	23.12	113.28	January, 2008	Active sampling	EA-IRMS	Standard Calibration Method	0.2	https://doi.org/10.1016/j.atmosenv.2008.01.042
$\delta^{15}N$	NO_x	Microbial Processes	-29.00‰	23.12	113.28	July, 2008	Active sampling	EA-IRMS	Standard Calibration Method	0.2	https://doi.org/10.1016/j.atmosenv.2008.01.042

$\delta^{15}N$	NO_x	Microbial Processes	-25.30‰	23.12	113.28	October, 2008	Active sampling	EA-IRMS	Standard Calibration Method	0.2	https://doi.org/10.1016/j.atmosenv.2008.01.042
$\delta^{15}N$	NO_x	Microbial Processes	-24.73‰	23.12	113.28	April, 2008	Active sampling	EA-IRMS	Standard Calibration Method	0.2	https://doi.org/10.1016/j.atmosenv.2008.01.042
$\delta^{15}N$	NO_x	Microbial Processes	-29.76‰	23.12	113.28	January, 2008	Active sampling	EA-IRMS	Standard Calibration Method	0.2	https://doi.org/10.1016/j.atmosenv.2008.01.042
$\delta^{15}N$	NO_x	Microbial Processes	-24.39‰	23.12	113.28	July, 2008	Active sampling	EA-IRMS	Standard Calibration Method	0.2	https://doi.org/10.1016/j.atmosenv.2008.01.042
$\delta^{15}N$	NO_x	Microbial Processes	-22.83‰	23.12	113.28	October, 2008	Active sampling	EA-IRMS	Standard Calibration Method	0.2	https://doi.org/10.1016/j.atmosenv.2008.01.042
$\delta^{15}N$	NO_x	Microbial Processes	-19.86‰	23.12	113.28	April, 2008	Active sampling	EA-IRMS	Standard Calibration Method	0.2	https://doi.org/10.1016/j.atmosenv.2008.01.042
$\delta^{15}N$	NO_x	Microbial Processes	-48.92‰	23.12	113.28	January, 2008	Active sampling	EA-IRMS	Standard Calibration Method	0.2	https://doi.org/10.1016/j.atmosenv.2008.01.042
$\delta^{15}N$	NO_x	Microbial Processes	-42.07‰	23.12	113.28	July, 2008	Active sampling	EA-IRMS	Standard Calibration Method	0.2	https://doi.org/10.1016/j.atmosenv.2008.01.042
$\delta^{15}N$	NO_x	Microbial Processes	-43.29‰	23.12	113.28	October, 2008	Active sampling	EA-IRMS	Standard Calibration Method	0.2	https://doi.org/10.1016/j.atmosenv.2008.01.042
$\delta^{15}N$	NO_x	Microbial Processes	-37.23‰	23.12	113.28	April, 2008	Active sampling	EA-IRMS	Standard Calibration Method	0.2	https://doi.org/10.1016/j.atmosenv.2008.01.042
$\delta^{15}N$	NO_x	Microbial Processes	-28.70‰	23.12	113.28	January, 2008	Active sampling	EA-IRMS	Standard Calibration Method	0.2	https://doi.org/10.1016/j.atmosenv.2008.01.042
$\delta^{15}N$	NO_x	Microbial Processes	-30.71‰	23.12	113.28	July, 2008	Active sampling	EA-IRMS	Standard Calibration Method	0.2	https://doi.org/10.1016/j.atmosenv.2008.01.042
$\delta^{15}N$	NO_x	Microbial Processes	-30.75‰	23.12	113.28	October, 2008	Active sampling	EA-IRMS	Standard Calibration Method	0.2	https://doi.org/10.1016/j.atmosenv.2008.01.042
$\delta^{15}N$	NO_x	Microbial Processes	-29.38‰	23.12	113.28	January, 2008	Active sampling	EA-IRMS	Standard Calibration Method	0.2	https://doi.org/10.1016/j.atmosenv.2008.01.042
$\delta^{15}N$	NO_x	Microbial Processes	-27.97‰	23.12	113.28	October, 2008	Active sampling	EA-IRMS	Standard Calibration Method	0.2	https://doi.org/10.1016/j.atmosenv.2008.01.042

$\delta^{15}N$	NO_x	Microbial Processes	-21.33‰	40.80	-77.86	May, 2016	Active sampling	IRMS	Standard Calibration Method	1.5	https://doi.org/10.1029/2018GL079619
$\delta^{15}N$	NO_x	Microbial Processes	-22.57‰	40.80	-77.86	May, 2016	Active sampling	IRMS	Standard Calibration Method	1.5	https://doi.org/10.1029/2018GL079619
$\delta^{15}N$	NO_x	Microbial Processes	-24.18‰	40.80	-77.86	May, 2016	Active sampling	IRMS	Standard Calibration Method	1.5	https://doi.org/10.1029/2018GL079619
$\delta^{15}N$	NO_x	Microbial Processes	-21.14‰	40.80	-77.86	May, 2016	Active sampling	IRMS	Standard Calibration Method	1.5	https://doi.org/10.1029/2018GL079619
$\delta^{15}N$	NO_x	Microbial Processes	-23.94‰	40.80	-77.86	May, 2016	Active sampling	IRMS	Standard Calibration Method	1.5	https://doi.org/10.1029/2018GL079619
$\delta^{15}N$	NO_x	Microbial Processes	-22.66‰	40.80	-77.86	May, 2016	Active sampling	IRMS	Standard Calibration Method	1.5	https://doi.org/10.1029/2018GL079619
$\delta^{15}N$	NO_x	Microbial Processes	-22.14‰	40.80	-77.86	May, 2016	Active sampling	IRMS	Standard Calibration Method	1.5	https://doi.org/10.1029/2018GL079619
$\delta^{15}N$	NO_x	Microbial Processes	-26.51‰	40.80	-77.86	May, 2016	Active sampling	IRMS	Standard Calibration Method	1.5	https://doi.org/10.1029/2018GL079619
$\delta^{15}N$	NO_x	Microbial Processes	-24.80‰	40.80	-77.86	May, 2016	Active sampling	IRMS	Standard Calibration Method	1.5	https://doi.org/10.1029/2018GL079619
$\delta^{15}N$	NO_x	Microbial Processes	-23.18‰	40.80	-77.86	May, 2016	Active sampling	IRMS	Standard Calibration Method	1.5	https://doi.org/10.1029/2018GL079619
$\delta^{15}N$	NO_x	Microbial Processes	-25.46‰	40.80	-77.86	May, 2016	Active sampling	IRMS	Standard Calibration Method	1.5	https://doi.org/10.1029/2018GL079619
$\delta^{15}N$	NO_x	Microbial Processes	-31.78‰	40.80	-77.86	May, 2016	Active sampling	IRMS	Standard Calibration Method	1.5	https://doi.org/10.1029/2018GL079619
$\delta^{15}N$	NO_x	Microbial Processes	-34.58‰	40.80	-77.86	May, 2016	Active sampling	IRMS	Standard Calibration Method	1.5	https://doi.org/10.1029/2018GL079619
$\delta^{15}N$	NO_x	Microbial Processes	-27.84‰	40.80	-77.86	May, 2016	Active sampling	IRMS	Standard Calibration Method	1.5	https://doi.org/10.1029/2018GL079619
$\delta^{15}N$	NO_x	Microbial Processes	-36.86‰	40.80	-77.86	May, 2016	Active sampling	IRMS	Standard Calibration Method	1.5	https://doi.org/10.1029/2018GL079619

$\delta^{15}N$	NO_x	Microbial Processes	-42.00‰	40.80	-77.86	May, 2016	Active sampling	IRMS	Standard Calibration Method	1.5	https://doi.org/10.1029/2018GL079619
$\delta^{15}N$	NO_x	Microbial Processes	-30.21‰	40.80	-77.86	May, 2016	Active sampling	IRMS	Standard Calibration Method	1.5	https://doi.org/10.1029/2018GL079619
$\delta^{15}N$	NO_x	Microbial Processes	-32.26‰	40.80	-77.86	May, 2016	Active sampling	IRMS	Standard Calibration Method	1.5	https://doi.org/10.1029/2018GL079619
$\delta^{15}N$	NO_x	Microbial Processes	-25.70‰	40.80	-77.86	May, 2016	Active sampling	IRMS	Standard Calibration Method	1.5	https://doi.org/10.1029/2018GL079619
$\delta^{15}N$	NO_x	Microbial Processes	-20.00‰	40.80	-77.86	May, 2017	Active sampling	IRMS	Standard Calibration Method	1.5	https://doi.org/10.1029/2018GL079619
$\delta^{15}N$	NO_x	Microbial Processes	-26.79‰	40.80	-77.86	May, 2017	Active sampling	IRMS	Standard Calibration Method	1.5	https://doi.org/10.1029/2018GL079619
$\delta^{15}N$	NO_x	Microbial Processes	-25.23‰	40.80	-77.86	May, 2017	Active sampling	IRMS	Standard Calibration Method	1.5	https://doi.org/10.1029/2018GL079619
$\delta^{15}N$	NO_x	Microbial Processes	-23.28‰	40.80	-77.86	May, 2017	Active sampling	IRMS	Standard Calibration Method	1.5	https://doi.org/10.1029/2018GL079619
$\delta^{15}N$	NO_x	Microbial Processes	-30.07‰	40.80	-77.86	May, 2017	Active sampling	IRMS	Standard Calibration Method	1.5	https://doi.org/10.1029/2018GL079619
$\delta^{15}N$	NO_x	Microbial Processes	-35.25‰	40.80	-77.86	May, 2017	Active sampling	IRMS	Standard Calibration Method	1.5	https://doi.org/10.1029/2018GL079619
$\delta^{15}N$	NO_x	Microbial Processes	-41.52‰	40.80	-77.86	May, 2017	Active sampling	IRMS	Standard Calibration Method	1.5	https://doi.org/10.1029/2018GL079619
$\delta^{15}N$	NO_x	Microbial Processes	-42.19‰	40.80	-77.86	May, 2017	Active sampling	IRMS	Standard Calibration Method	1.5	https://doi.org/10.1029/2018GL079619
$\delta^{15}N$	NO_x	Microbial Processes	-40.71‰	40.80	-77.86	May, 2017	Active sampling	IRMS	Standard Calibration Method	1.5	https://doi.org/10.1029/2018GL079619
$\delta^{15}N$	NO_x	Microbial Processes	-35.20‰	40.80	-77.86	May, 2017	Active sampling	IRMS	Standard Calibration Method	1.5	https://doi.org/10.1029/2018GL079619
$\delta^{15}N$	NO_x	Microbial Processes	-17.01‰	40.80	-77.86	May, 2017	Active sampling	IRMS	Standard Calibration Method	1.5	https://doi.org/10.1029/2018GL079619

$\delta^{15}N$	NO_x	Microbial Processes	-14.20‰	40.80	-77.86	May, 2017	Active sampling	IRMS	Standard Calibration Method	1.5	https://doi.org/10.1029/2018GL079619
$\delta^{15}N$	NO_x	Microbial Processes	-21.47‰	40.80	-77.86	May, 2017	Active sampling	IRMS	Standard Calibration Method	1.5	https://doi.org/10.1029/2018GL079619
$\delta^{15}N$	NO_x	Microbial Processes	-33.68‰	40.80	-77.86	May, 2017	Active sampling	IRMS	Standard Calibration Method	1.5	https://doi.org/10.1029/2018GL079619
$\delta^{15}N$	NO_x	Microbial Processes	-36.25‰	40.80	-77.86	May, 2017	Active sampling	IRMS	Standard Calibration Method	1.5	https://doi.org/10.1029/2018GL079619
$\delta^{15}N$	NO_x	Microbial Processes	-24.18‰	40.80	-77.86	May, 2017	Active sampling	IRMS	Standard Calibration Method	1.5	https://doi.org/10.1029/2018GL079619
$\delta^{15}N$	NO_x	Microbial Processes	-26.50‰	39.03	-76.91	19 June-22 July, 2010	Active sampling	CF-IRMS	Standard Calibration Method	0.2	http://dx.doi.org/10.1016/j.atmosenv.2014.04.005
$\delta^{15}N$	NO_x	Microbial Processes	-30.80‰	39.03	-76.91	19 June-22 July, 2010	Active sampling	CF-IRMS	Standard Calibration Method	0.2	http://dx.doi.org/10.1016/j.atmosenv.2014.04.005
$\delta^{15}N$	NO_x	Microbial Processes	-37.10‰	40.44	-79.96	August, 2016	Active sampling	CF-IRMS	Standard Calibration Method	0.3	https://doi.org/10.1021/acs.est.7b00592
$\delta^{15}N$	NO_x	Microbial Processes	-38.80‰	40.44	-79.96	August, 2016	Active sampling	CF-IRMS	Standard Calibration Method	0.3	https://doi.org/10.1021/acs.est.7b00592
$\delta^{15}N$	NO_x	Microbial Processes	-40.50‰	40.44	-79.96	August, 2016	Active sampling	CF-IRMS	Standard Calibration Method	0.3	https://doi.org/10.1021/acs.est.7b00592
$\delta^{15}N$	NO_x	Microbial Processes	-49.30‰	40.44	-79.96	August, 2016	Active sampling	CF-IRMS	Standard Calibration Method	0.3	https://doi.org/10.1021/acs.est.7b00592
$\delta^{15}N$	NO_x	Microbial Processes	-52.90‰	40.44	-79.96	August, 2016	Active sampling	CF-IRMS	Standard Calibration Method	0.3	https://doi.org/10.1021/acs.est.7b00592
$\delta^{15}N$	NO_x	Microbial Processes	-53.70‰	40.44	-79.96	August, 2016	Active sampling	CF-IRMS	Standard Calibration Method	0.3	https://doi.org/10.1021/acs.est.7b00592
$\delta^{15}N$	NO_x	Microbial Processes	-53.60‰	40.44	-79.96	August, 2016	Active sampling	CF-IRMS	Standard Calibration Method	0.3	https://doi.org/10.1021/acs.est.7b00592
$\delta^{15}N$	NO_x	Microbial Processes	-36.80‰	40.44	-79.96	August, 2016	Active sampling	CF-IRMS	Standard Calibration Method	0.3	https://doi.org/10.1021/acs.est.7b00592

$\delta^{15}N$	NO_x	Microbial Processes	-37.40‰	40.44	-79.96	August, 2016	Active sampling	CF-IRMS	Standard Calibration Method	0.3	https://doi.org/10.1021/acs.est.7b00592
$\delta^{15}N$	NO_x	Microbial Processes	-39.50‰	40.44	-79.96	August, 2016	Active sampling	CF-IRMS	Standard Calibration Method	0.3	https://doi.org/10.1021/acs.est.7b00592
$\delta^{15}N$	NO_x	Microbial Processes	-47.80‰	40.44	-79.96	August, 2016	Active sampling	CF-IRMS	Standard Calibration Method	0.3	https://doi.org/10.1021/acs.est.7b00592
$\delta^{15}N$	NO_x	Microbial Processes	-52.50‰	40.44	-79.96	August, 2016	Active sampling	CF-IRMS	Standard Calibration Method	0.3	https://doi.org/10.1021/acs.est.7b00592
$\delta^{15}N$	NO_x	Microbial Processes	-53.40‰	40.44	-79.96	August, 2016	Active sampling	CF-IRMS	Standard Calibration Method	0.3	https://doi.org/10.1021/acs.est.7b00592
$\delta^{15}N$	NO_x	Microbial Processes	-51.80‰	40.44	-79.96	August, 2016	Active sampling	CF-IRMS	Standard Calibration Method	0.3	https://doi.org/10.1021/acs.est.7b00592
$\delta^{15}N$	NO_x	Microbial Processes	-36.30‰	40.44	-79.96	August, 2016	Active sampling	CF-IRMS	Standard Calibration Method	0.3	https://doi.org/10.1021/acs.est.7b00592
$\delta^{15}N$	NO_x	Microbial Processes	-37.70‰	40.44	-79.96	August, 2016	Active sampling	CF-IRMS	Standard Calibration Method	0.3	https://doi.org/10.1021/acs.est.7b00592
$\delta^{15}N$	NO_x	Microbial Processes	-39.60‰	40.44	-79.96	August, 2016	Active sampling	CF-IRMS	Standard Calibration Method	0.3	https://doi.org/10.1021/acs.est.7b00592
$\delta^{15}N$	NO_x	Microbial Processes	-47.80‰	40.44	-79.96	August, 2016	Active sampling	CF-IRMS	Standard Calibration Method	0.3	https://doi.org/10.1021/acs.est.7b00592
$\delta^{15}N$	NO_x	Microbial Processes	-50.60‰	40.44	-79.96	August, 2016	Active sampling	CF-IRMS	Standard Calibration Method	0.3	https://doi.org/10.1021/acs.est.7b00592
$\delta^{15}N$	NO_x	Microbial Processes	-52.80‰	40.44	-79.96	August, 2016	Active sampling	CF-IRMS	Standard Calibration Method	0.3	https://doi.org/10.1021/acs.est.7b00592
$\delta^{15}N$	NO_x	Microbial Processes	-33.42‰	41.80	123.45	April, 2020	Active sampling	CF-IRMS	Standard Calibration Method	1.5	https://doi.org/10.1029/2020JG005705
$\delta^{15}N$	NO_x	Microbial Processes	-26.98‰	41.80	123.45	January, 2020	Active sampling	CF-IRMS	Standard Calibration Method	1.5	https://doi.org/10.1029/2020JG005705
$\delta^{15}N$	NO_x	Microbial Processes	-23.53‰	41.80	123.45	July, 2020	Active sampling	CF-IRMS	Standard Calibration Method	1.5	https://doi.org/10.1029/2020JG005705

$\delta^{15}N$	NO_x	Microbial Processes	-35.74‰	43.87	126.57	October, 2020	Active sampling	CF-IRMS	Standard Calibration Method	1.5	https://doi.org/10.1029/2020JG005705
$\delta^{15}N$	NO_x	Microbial Processes	-34.11‰	43.87	126.57	April, 2020	Active sampling	CF-IRMS	Standard Calibration Method	1.5	https://doi.org/10.1029/2020JG005705
$\delta^{15}N$	NO_x	Microbial Processes	-33.56‰	43.87	126.57	January, 2020	Active sampling	CF-IRMS	Standard Calibration Method	1.5	https://doi.org/10.1029/2020JG005705
$\delta^{15}N$	NO_x	Microbial Processes	-32.81‰	43.87	126.57	July, 2020	Active sampling	CF-IRMS	Standard Calibration Method	1.5	https://doi.org/10.1029/2020JG005705
$\delta^{15}N$	NO_x	Microbial Processes	-27.64‰	42.10	124.92	October, 2020	Active sampling	CF-IRMS	Standard Calibration Method	1.5	https://doi.org/10.1029/2020JG005705
$\delta^{15}N$	NO_x	Microbial Processes	-17.08‰	42.10	124.92	April, 2020	Active sampling	CF-IRMS	Standard Calibration Method	1.5	https://doi.org/10.1029/2020JG005705
$\delta^{15}N$	NO_x	Microbial Processes	-10.81‰	42.10	124.92	January, 2020	Active sampling	CF-IRMS	Standard Calibration Method	1.5	https://doi.org/10.1029/2020JG005705
$\delta^{15}N$	NO_x	Microbial Processes	-7.93‰	42.10	124.92	July, 2020	Active sampling	CF-IRMS	Standard Calibration Method	1.5	https://doi.org/10.1029/2020JG005705
$\delta^{15}N$	NO_x	Microbial Processes	-23.19‰	42.10	124.92	October, 2020	Active sampling	CF-IRMS	Standard Calibration Method	1.5	https://doi.org/10.1029/2020JG005705
$\delta^{15}N$	NO_x	Microbial Processes	-8.86‰	42.10	124.92	April, 2020	Active sampling	CF-IRMS	Standard Calibration Method	1.5	https://doi.org/10.1029/2020JG005705
$\delta^{15}N$	NO_x	Microbial Processes	14.19‰	42.10	124.92	January, 2020	Active sampling	CF-IRMS	Standard Calibration Method	1.5	https://doi.org/10.1029/2020JG005705
$\delta^{15}N$	NO_x	Microbial Processes	41.54‰	42.10	124.92	July, 2020	Active sampling	CF-IRMS	Standard Calibration Method	1.5	https://doi.org/10.1029/2020JG005705
$\delta^{15}N$	NO_x	Microbial Processes	-37.40‰	50.24	120.19	October, 2020	Active sampling	CF-IRMS	Standard Calibration Method	1.5	https://doi.org/10.1029/2020JG005705
$\delta^{15}N$	NO_x	Microbial Processes	-24.81‰	50.24	120.19	April, 2020	Active sampling	CF-IRMS	Standard Calibration Method	1.5	https://doi.org/10.1029/2020JG005705
$\delta^{15}N$	NO_x	Microbial Processes	-20.60‰	50.24	120.19	January, 2020	Active sampling	CF-IRMS	Standard Calibration Method	1.5	https://doi.org/10.1029/2020JG005705

$\delta^{15}N$	NO_x	Microbial Processes	-13.79‰	50.24	120.19	July, 2020	Active sampling	CF-IRMS	Standard Calibration Method	1.5	https://doi.org/10.1029/2020JG005705
$\delta^{15}N$	NO_x	Microbial Processes	-8.41‰	50.24	120.19	October, 2020	Active sampling	CF-IRMS	Standard Calibration Method	1.5	https://doi.org/10.1029/2020JG005705
$\delta^{15}N$	NO_x	Microbial Processes	-31.33‰	36.96	100.90	April, 2020	Active sampling	CF-IRMS	Standard Calibration Method	1.5	https://doi.org/10.1029/2020JG005705
$\delta^{15}N$	NO_x	Microbial Processes	-29.44‰	36.96	100.90	January, 2020	Active sampling	CF-IRMS	Standard Calibration Method	1.5	https://doi.org/10.1029/2020JG005705
$\delta^{15}N$	NO_x	Microbial Processes	-25.78‰	36.96	100.90	July, 2020	Active sampling	CF-IRMS	Standard Calibration Method	1.5	https://doi.org/10.1029/2020JG005705
$\delta^{15}N$	NO_x	Microbial Processes	-26.11‰	36.96	100.90	October, 2020	Active sampling	CF-IRMS	Standard Calibration Method	1.5	https://doi.org/10.1029/2020JG005705
$\delta^{15}N$	NO_x	Microbial Processes	-25.37‰	36.96	100.90	April, 2020	Active sampling	CF-IRMS	Standard Calibration Method	1.5	https://doi.org/10.1029/2020JG005705
$\delta^{15}N$	NO_x	Microbial Processes	-43.47‰	42.20	116.49	January, 2020	Active sampling	CF-IRMS	Standard Calibration Method	1.5	https://doi.org/10.1029/2020JG005705
$\delta^{15}N$	NO_x	Microbial Processes	-41.76‰	42.20	116.49	July, 2020	Active sampling	CF-IRMS	Standard Calibration Method	1.5	https://doi.org/10.1029/2020JG005705
$\delta^{15}N$	NO_x	Microbial Processes	-41.48‰	42.20	116.49	October, 2020	Active sampling	CF-IRMS	Standard Calibration Method	1.5	https://doi.org/10.1029/2020JG005705
$\delta^{15}N$	NO_x	Microbial Processes	-40.22‰	42.20	116.49	January, 2020	Active sampling	CF-IRMS	Standard Calibration Method	1.5	https://doi.org/10.1029/2020JG005705
$\delta^{15}N$	NO_x	Microbial Processes	-39.31‰	42.20	116.49	October, 2020	Active sampling	CF-IRMS	Standard Calibration Method	1.5	https://doi.org/10.1029/2020JG005705
$\delta^{15}N$	NO_x	Microbial Processes	-44.70‰	40.27	-76.89	July, 2017	Active sampling	CF-IRMS	Standard Calibration Method	0.3	https://doi.org/10.5194/bg-18-805-2021
$\delta^{15}N$	NO_x	Microbial Processes	-43.50‰	40.27	-76.89	July, 2017	Active sampling	CF-IRMS	Standard Calibration Method	0.3	https://doi.org/10.5194/bg-18-805-2021
$\delta^{15}N$	NO_x	Microbial Processes	-40.20‰	40.27	-76.89	July, 2017	Active sampling	CF-IRMS	Standard Calibration Method	0.3	https://doi.org/10.5194/bg-18-805-2021

$\delta^{15}N$	NO_x	Microbial Processes	-37.10‰	40.27	-76.89	July, 2017	Active sampling	CF-IRMS	Standard Calibration Method	0.3	https://doi.org/10.5194/bg-18-805-2021
$\delta^{15}N$	NO_x	Microbial Processes	-32.80‰	40.27	-76.89	July, 2017	Active sampling	CF-IRMS	Standard Calibration Method	0.3	https://doi.org/10.5194/bg-18-805-2021
$\delta^{15}N$	NO_x	Microbial Processes	-29.10‰	40.27	-76.89	July, 2017	Active sampling	CF-IRMS	Standard Calibration Method	0.3	https://doi.org/10.5194/bg-18-805-2021
$\delta^{15}N$	NO_x	Microbial Processes	-26.80‰	40.27	-76.89	July, 2017	Active sampling	CF-IRMS	Standard Calibration Method	0.3	https://doi.org/10.5194/bg-18-805-2021
$\delta^{15}N$	NO_x	Microbial Processes	-20.80‰	40.27	-76.89	July, 2017	Active sampling	CF-IRMS	Standard Calibration Method	0.3	https://doi.org/10.5194/bg-18-805-2021
$\delta^{15}N$	NO_x	Microbial Processes	-54.90‰	40.27	-76.89	July, 2017	Active sampling	CF-IRMS	Standard Calibration Method	0.3	https://doi.org/10.5194/bg-18-805-2021
$\delta^{15}N$	NO_x	Microbial Processes	-53.30‰	40.27	-76.89	July, 2017	Active sampling	CF-IRMS	Standard Calibration Method	0.3	https://doi.org/10.5194/bg-18-805-2021
$\delta^{15}N$	NO_x	Microbial Processes	-37.40‰	40.27	-76.89	July, 2017	Active sampling	CF-IRMS	Standard Calibration Method	0.3	https://doi.org/10.5194/bg-18-805-2021
$\delta^{15}N$	NO_x	Microbial Processes	-33.50‰	40.27	-76.89	July, 2017	Active sampling	CF-IRMS	Standard Calibration Method	0.3	https://doi.org/10.5194/bg-18-805-2021
$\delta^{15}N$	NO_x	Microbial Processes	-17.90‰	40.27	-76.89	July, 2017	Active sampling	CF-IRMS	Standard Calibration Method	0.3	https://doi.org/10.5194/bg-18-805-2021
$\delta^{15}N$	NO_x	Microbial Processes	-16.80‰	40.27	-76.89	July, 2017	Active sampling	CF-IRMS	Standard Calibration Method	0.3	https://doi.org/10.5194/bg-18-805-2021
$\delta^{15}N$	NO_x	Microbial Processes	-50.50‰	40.27	-76.89	July, 2017	Active sampling	CF-IRMS	Standard Calibration Method	0.3	https://doi.org/10.5194/bg-18-805-2021
$\delta^{15}N$	NO_x	Microbial Processes	-51.40‰	40.27	-76.89	July, 2017	Active sampling	CF-IRMS	Standard Calibration Method	0.3	https://doi.org/10.5194/bg-18-805-2021
$\delta^{15}N$	NO_x	Microbial Processes	-38.50‰	40.27	-76.89	July, 2017	Active sampling	CF-IRMS	Standard Calibration Method	0.3	https://doi.org/10.5194/bg-18-805-2021
$\delta^{15}N$	NO_x	Microbial Processes	-37.20‰	40.27	-76.89	July, 2017	Active sampling	CF-IRMS	Standard Calibration Method	0.3	https://doi.org/10.5194/bg-18-805-2021

$\delta^{15}N$	NO_x	Microbial Processes	-24.30‰	40.27	-76.89	July, 2017	Active sampling	CF-IRMS	Standard Calibration Method	0.3	https://doi.org/10.5194/bg-18-805-2021
$\delta^{15}N$	NO_x	Microbial Processes	-21.30‰	40.27	-76.89	July, 2017	Active sampling	CF-IRMS	Standard Calibration Method	0.3	https://doi.org/10.5194/bg-18-805-2021
$\delta^{34}S$	S	Coal Combustion (Not used)	-17.06‰	26.57	106.73	October, 1993	Passive sampling	IRMS	Standard Calibration Method	0.2	10.1007/BF02869045
$\delta^{34}S$	S	Coal Combustion (Not used)	-17.46‰	26.57	106.73	January, 1993	Passive sampling	IRMS	Standard Calibration Method	0.2	10.1007/BF02869045
$\delta^{34}S$	S	Coal Combustion (Not used)	-15.75‰	26.57	106.73	April, 1993	Passive sampling	IRMS	Standard Calibration Method	0.2	10.1007/BF02869045
$\delta^{34}S$	S	Coal Combustion (Not used)	-14.12‰	26.57	106.73	July, 1993	Passive sampling	IRMS	Standard Calibration Method	0.2	10.1007/BF02869045
$\delta^{34}S$	S	Coal Combustion (Not used)	-11.01‰	26.57	106.73	October, 1993	Passive sampling	IRMS	Standard Calibration Method	0.2	10.1007/BF02869045
$\delta^{34}S$	S	Biomass Burning	5.40‰	32.06	118.80	April, 2006	Active sampling	IRMS	Standard Calibration Method	0.2	10.1039/c1em10073d
$\delta^{34}S$	S	Biomass Burning	3.00‰	32.06	118.80	January, 2006	Active sampling	IRMS	Standard Calibration Method	0.2	10.1039/c1em10073d
$\delta^{34}S$	S	Biomass Burning	2.50‰	30.27	120.16	July, 2006	Active sampling	IRMS	Standard Calibration Method	0.2	10.1039/c1em10073d
$\delta^{34}S$	S	Biomass Burning	7.60‰	31.82	117.23	October, 2006	Active sampling	IRMS	Standard Calibration Method	0.2	10.1039/c1em10073d
$\delta^{34}S$	S	Biomass Burning	-5.80‰	28.68	115.86	April, 2006	Active sampling	IRMS	Standard Calibration Method	0.2	10.1039/c1em10073d
$\delta^{34}S$	S	Biomass Burning	-4.30‰	30.59	114.31	January, 2006	Active sampling	IRMS	Standard Calibration Method	0.2	10.1039/c1em10073d
$\delta^{34}S$	S	Biomass Burning	3.10‰	28.23	112.94	July, 2006	Active sampling	IRMS	Standard Calibration Method	0.2	10.1039/c1em10073d
$\delta^{34}S$	S	Biomass Burning	-8.10‰	26.65	106.63	October, 2006	Active sampling	IRMS	Standard Calibration Method	0.2	10.1039/c1em10073d

$\delta^{34}S$	S	Biomass Burning	-4.70‰	26.65	106.63	April, 2006	Active sampling	IRMS	Standard Calibration Method	0.2	10.1039/c1em10073d
$\delta^{34}S$	S	Biomass Burning	-0.30‰	29.56	106.55	January, 2006	Active sampling	IRMS	Standard Calibration Method	0.2	10.1039/c1em10073d
$\delta^{34}S$	S	Biomass Burning	0.50‰	30.57	104.07	July, 2006	Active sampling	IRMS	Standard Calibration Method	0.2	10.1039/c1em10073d
$\delta^{34}S$	S	Biomass Burning	9.70‰	37.87	112.55	October, 2006	Active sampling	IRMS	Standard Calibration Method	0.2	10.1039/c1em10073d
$\delta^{34}S$	S	Biomass Burning	6.00‰	32.10	118.50	July, 2014	Active sampling	IRMS	Standard Calibration Method	0.2	http://dx.doi.org/10.1016/j.atmosres.2016.01.011
$\delta^{34}S$	S	Biomass Burning	5.20‰	32.10	118.50	July, 2014	Active sampling	IRMS	Standard Calibration Method	0.2	http://dx.doi.org/10.1016/j.atmosres.2016.01.011
$\delta^{34}S$	S	Biomass Burning	7.90‰	32.10	118.50	July, 2014	Active sampling	IRMS	Standard Calibration Method	0.2	http://dx.doi.org/10.1016/j.atmosres.2016.01.011
$\delta^{34}S$	S	Biomass Burning	9.10‰	28.70	77.10	April, 2016	Active sampling	IRMS	Standard Calibration Method	0.2	https://doi.org/10.1016/j.apr.2018.12.015
$\delta^{34}S$	S	Biomass Burning	7.30‰	28.70	77.10	October, 2016	Active sampling	IRMS	Standard Calibration Method	0.2	https://doi.org/10.1016/j.apr.2018.12.015
$\delta^{34}S$	S	Biomass Burning	8.90‰	28.70	77.10	April, 2016	Active sampling	IRMS	Standard Calibration Method	0.2	https://doi.org/10.1016/j.apr.2018.12.015
$\delta^{34}S$	S	Coal Combustion	1.40‰	52.33	124.70	January, 1993	Active sampling	IRMS	Standard Calibration Method	0.2	10.1007/BF02869045
$\delta^{34}S$	S	Coal Combustion	11.22‰	39.98	124.35	April, 1993	Active sampling	IRMS	Standard Calibration Method	0.2	10.1007/BF02869045
$\delta^{34}S$	S	Coal Combustion	1.73‰	43.88	125.32	July, 1993	Active sampling	IRMS	Standard Calibration Method	0.2	10.1007/BF02869045
$\delta^{34}S$	S	Coal Combustion	0.80‰	40.82	111.75	October, 1993	Active sampling	IRMS	Standard Calibration Method	0.2	10.1007/BF02869045
$\delta^{34}S$	S	Coal Combustion	2.30‰	38.03	114.47	January, 1993	Active sampling	IRMS	Standard Calibration Method	0.2	10.1007/BF02869045

$\delta^{34}S$	S	Coal Combustion	-3.88‰	36.62	114.53	April, 1993	Active sampling	IRMS	Standard Calibration Method	0.2	10.1007/BF02869045
$\delta^{34}S$	S	Coal Combustion	4.75‰	39.59	116.18	July, 1993	Active sampling	IRMS	Standard Calibration Method	0.2	10.1007/BF02869045
$\delta^{34}S$	S	Coal Combustion	6.43‰	40.07	113.30	October, 1993	Active sampling	IRMS	Standard Calibration Method	0.2	10.1007/BF02869045
$\delta^{34}S$	S	Coal Combustion	4.69‰	36.65	119.15	January, 1993	Active sampling	IRMS	Standard Calibration Method	0.2	10.1007/BF02869045
$\delta^{34}S$	S	Coal Combustion	7.33‰	34.75	111.87	April, 1993	Active sampling	IRMS	Standard Calibration Method	0.2	10.1007/BF02869045
$\delta^{34}S$	S	Coal Combustion	10.19‰	31.97	120.88	July, 1993	Active sampling	IRMS	Standard Calibration Method	0.2	10.1007/BF02869045
$\delta^{34}S$	S	Coal Combustion	5.36‰	32.06	118.80	October, 1993	Active sampling	IRMS	Standard Calibration Method	0.2	10.1007/BF02869045
$\delta^{34}S$	S	Coal Combustion	2.53‰	30.27	120.15	January, 1993	Active sampling	IRMS	Standard Calibration Method	0.2	10.1007/BF02869045
$\delta^{34}S$	S	Coal Combustion	3.79‰	33.95	116.80	April, 1993	Active sampling	IRMS	Standard Calibration Method	0.2	10.1007/BF02869045
$\delta^{34}S$	S	Coal Combustion	-3.06‰	27.12	113.95	July, 1993	Active sampling	IRMS	Standard Calibration Method	0.2	10.1007/BF02869045
$\delta^{34}S$	S	Coal Combustion	-0.65‰	24.87	102.82	October, 1993	Active sampling	IRMS	Standard Calibration Method	0.2	10.1007/BF02869045
$\delta^{34}S$	S	Coal Combustion	-4.71‰	26.57	106.73	January, 1993	Active sampling	IRMS	Standard Calibration Method	0.2	10.1007/BF02869045
$\delta^{34}S$	S	Coal Combustion	-9.89‰	26.57	106.73	April, 1993	Active sampling	IRMS	Standard Calibration Method	0.2	10.1007/BF02869045
$\delta^{34}S$	S	Coal Combustion	-8.09‰	26.57	106.73	July, 1993	Active sampling	IRMS	Standard Calibration Method	0.2	10.1007/BF02869045
$\delta^{34}S$	S	Coal Combustion	-5.71‰	26.57	106.73	October, 1993	Active sampling	IRMS	Standard Calibration Method	0.2	10.1007/BF02869045

$\delta^{34}S$	S	Coal Combustion	-5.22‰	26.57	106.73	January, 1993	Active sampling	IRMS	Standard Calibration Method	0.2	10.1007/BF02869045
$\delta^{34}S$	S	Coal Combustion	-8.87‰	26.57	106.73	April, 1993	Active sampling	IRMS	Standard Calibration Method	0.2	10.1007/BF02869045
$\delta^{34}S$	S	Coal Combustion	-7.05‰	26.57	106.73	July, 1993	Active sampling	IRMS	Standard Calibration Method	0.2	10.1007/BF02869045
$\delta^{34}S$	S	Coal Combustion	-5.51‰	26.57	106.73	October, 1993	Active sampling	IRMS	Standard Calibration Method	0.2	10.1007/BF02869045
$\delta^{34}S$	S	Coal Combustion	-10.34‰	26.57	106.73	January, 1993	Active sampling	IRMS	Standard Calibration Method	0.2	10.1007/BF02869045
$\delta^{34}S$	S	Coal Combustion	-9.61‰	26.57	106.73	April, 1993	Active sampling	IRMS	Standard Calibration Method	0.2	10.1007/BF02869045
$\delta^{34}S$	S	Coal Combustion	-6.49‰	26.57	106.73	July, 1993	Active sampling	IRMS	Standard Calibration Method	0.2	10.1007/BF02869045
$\delta^{34}S$	S	Coal Combustion	-9.14‰	26.57	106.73	October, 1993	Active sampling	IRMS	Standard Calibration Method	0.2	10.1007/BF02869045
$\delta^{34}S$	S	Coal Combustion	-12.01‰	26.57	106.73	January, 1993	Active sampling	IRMS	Standard Calibration Method	0.2	10.1007/BF02869045
$\delta^{34}S$	S	Coal Combustion	-11.24‰	26.57	106.73	April, 1993	Active sampling	IRMS	Standard Calibration Method	0.2	10.1007/BF02869045
$\delta^{34}S$	S	Coal Combustion	-6.56‰	26.57	106.73	July, 1993	Active sampling	IRMS	Standard Calibration Method	0.2	10.1007/BF02869045
$\delta^{34}S$	S	Coal Combustion	-7.00‰	26.57	106.73	October, 1993	Active sampling	IRMS	Standard Calibration Method	0.2	10.1007/BF02869045
$\delta^{34}S$	S	Coal Combustion	-2.51‰	26.57	106.73	January, 1993	Active sampling	IRMS	Standard Calibration Method	0.2	10.1007/BF02869045
$\delta^{34}S$	S	Coal Combustion	-5.56‰	26.57	106.73	April, 1993	Active sampling	IRMS	Standard Calibration Method	0.2	10.1007/BF02869045
$\delta^{34}S$	S	Coal Combustion	-6.39‰	26.57	106.73	July, 1993	Active sampling	IRMS	Standard Calibration Method	0.2	10.1007/BF02869045

$\delta^{34}S$	S	Coal Combustion	15.83‰	39.91	116.39	April, 2011	Active sampling	IRMS	Standard Calibration Method	0.2	10.1016/j.orggeochem.2010.10.011
$\delta^{34}S$	S	Coal Combustion	49.95‰	39.91	116.39	January, 2011	Active sampling	IRMS	Standard Calibration Method	0.2	10.1016/j.orggeochem.2010.10.011
$\delta^{34}S$	S	Coal Combustion	35.11‰	39.91	116.39	July, 2011	Active sampling	IRMS	Standard Calibration Method	0.2	10.1016/j.orggeochem.2010.10.011
$\delta^{34}S$	S	Coal Combustion	24.73‰	39.91	116.39	October, 2011	Active sampling	IRMS	Standard Calibration Method	0.2	10.1016/j.orggeochem.2010.10.011
$\delta^{34}S$	S	Coal Combustion	23.42‰	39.91	116.39	April, 2011	Active sampling	IRMS	Standard Calibration Method	0.2	10.1016/j.orggeochem.2010.10.011
$\delta^{34}S$	S	Coal Combustion	20.89‰	39.91	116.39	January, 2011	Active sampling	IRMS	Standard Calibration Method	0.2	10.1016/j.orggeochem.2010.10.011
$\delta^{34}S$	S	Coal Combustion	13.65‰	39.91	116.39	July, 2011	Active sampling	IRMS	Standard Calibration Method	0.2	10.1016/j.orggeochem.2010.10.011
$\delta^{34}S$	S	Coal Combustion	2.39‰	39.91	116.39	October, 2011	Active sampling	IRMS	Standard Calibration Method	0.2	10.1016/j.orggeochem.2010.10.011
$\delta^{34}S$	S	Coal Combustion	8.67‰	39.91	116.39	April, 2011	Active sampling	IRMS	Standard Calibration Method	0.2	10.1016/j.orggeochem.2010.10.011
$\delta^{34}S$	S	Coal Combustion	-3.28‰	39.91	116.39	January, 2011	Active sampling	IRMS	Standard Calibration Method	0.2	10.1016/j.orggeochem.2010.10.011
$\delta^{34}S$	S	Coal Combustion	-5.64‰	39.91	116.39	July, 2011	Active sampling	IRMS	Standard Calibration Method	0.2	10.1016/j.orggeochem.2010.10.011
$\delta^{34}S$	S	Coal Combustion	-9.30‰	39.91	116.39	October, 2011	Active sampling	IRMS	Standard Calibration Method	0.2	10.1016/j.orggeochem.2010.10.011
$\delta^{34}S$	S	Coal Combustion	-15.50‰	39.91	116.39	April, 2011	Active sampling	IRMS	Standard Calibration Method	0.2	10.1016/j.orggeochem.2010.10.011
$\delta^{34}S$	S	Coal Combustion	6.06‰	39.91	116.39	January, 2011	Active sampling	IRMS	Standard Calibration Method	0.2	10.1016/j.orggeochem.2010.10.011
$\delta^{34}S$	S	Coal Combustion	7.10‰	39.91	116.39	July, 2011	Active sampling	IRMS	Standard Calibration Method	0.2	10.1016/j.orggeochem.2010.10.011

$\delta^{34}S$	S	Coal Combustion	1.69‰	39.91	116.39	October, 2011	Active sampling	IRMS	Standard Calibration Method	0.2	10.1016/j.orggeochem.2010.10.011
$\delta^{34}S$	S	Coal Combustion	-0.66‰	39.91	116.39	April, 2011	Active sampling	IRMS	Standard Calibration Method	0.2	10.1016/j.orggeochem.2010.10.011
$\delta^{34}S$	S	Coal Combustion	1.17‰	39.91	116.39	January, 2011	Active sampling	IRMS	Standard Calibration Method	0.2	10.1016/j.orggeochem.2010.10.011
$\delta^{34}S$	S	Coal Combustion	-5.11‰	39.91	116.39	July, 2011	Active sampling	IRMS	Standard Calibration Method	0.2	10.1016/j.orggeochem.2010.10.011
$\delta^{34}S$	S	Coal Combustion	10.77‰	39.91	116.39	October, 2011	Active sampling	IRMS	Standard Calibration Method	0.2	10.1016/j.orggeochem.2010.10.011
$\delta^{34}S$	S	Coal Combustion	13.39‰	39.91	116.39	April, 2011	Active sampling	IRMS	Standard Calibration Method	0.2	10.1016/j.orggeochem.2010.10.011
$\delta^{34}S$	S	Coal Combustion	9.37‰	39.91	116.39	January, 2011	Active sampling	IRMS	Standard Calibration Method	0.2	10.1016/j.orggeochem.2010.10.011
$\delta^{34}S$	S	Coal Combustion	7.71‰	39.91	116.39	July, 2011	Active sampling	IRMS	Standard Calibration Method	0.2	10.1016/j.orggeochem.2010.10.011
$\delta^{34}S$	S	Coal Combustion	5.45‰	39.91	116.39	October, 2011	Active sampling	IRMS	Standard Calibration Method	0.2	10.1016/j.orggeochem.2010.10.011
$\delta^{34}S$	S	Coal Combustion	1.61‰	39.91	116.39	April, 2011	Active sampling	IRMS	Standard Calibration Method	0.2	10.1016/j.orggeochem.2010.10.011
$\delta^{34}S$	S	Coal Combustion	-0.58‰	39.91	116.39	January, 2011	Active sampling	IRMS	Standard Calibration Method	0.2	10.1016/j.orggeochem.2010.10.011
$\delta^{34}S$	S	Coal Combustion	-0.66‰	39.91	116.39	July, 2011	Active sampling	IRMS	Standard Calibration Method	0.2	10.1016/j.orggeochem.2010.10.011
$\delta^{34}S$	S	Coal Combustion	-2.32‰	39.91	116.39	October, 2011	Active sampling	IRMS	Standard Calibration Method	0.2	10.1016/j.orggeochem.2010.10.011
$\delta^{34}S$	S	Coal Combustion	15.31‰	39.91	116.39	April, 2011	Active sampling	IRMS	Standard Calibration Method	0.2	10.1016/j.orggeochem.2010.10.011
$\delta^{34}S$	S	Coal Combustion	16.53‰	39.91	116.39	January, 2011	Active sampling	IRMS	Standard Calibration Method	0.2	10.1016/j.orggeochem.2010.10.011

$\delta^{34}S$	S	Coal Combustion	22.46‰	39.91	116.39	July, 2011	Active sampling	IRMS	Standard Calibration Method	0.2	10.1016/j.orggeochem.2010.10.011
$\delta^{34}S$	S	Coal Combustion	21.41‰	39.91	116.39	October, 2011	Active sampling	IRMS	Standard Calibration Method	0.2	10.1016/j.orggeochem.2010.10.011
$\delta^{34}S$	S	Coal Combustion	19.84‰	39.91	116.39	April, 2011	Active sampling	IRMS	Standard Calibration Method	0.2	10.1016/j.orggeochem.2010.10.011
$\delta^{34}S$	S	Coal Combustion	17.57‰	39.91	116.39	January, 2011	Active sampling	IRMS	Standard Calibration Method	0.2	10.1016/j.orggeochem.2010.10.011
$\delta^{34}S$	S	Coal Combustion	17.57‰	39.91	116.39	July, 2011	Active sampling	IRMS	Standard Calibration Method	0.2	10.1016/j.orggeochem.2010.10.011
$\delta^{34}S$	S	Coal Combustion	15.65‰	39.91	116.39	October, 2011	Active sampling	IRMS	Standard Calibration Method	0.2	10.1016/j.orggeochem.2010.10.011
$\delta^{34}S$	S	Coal Combustion	10.94‰	39.91	116.39	April, 2011	Active sampling	IRMS	Standard Calibration Method	0.2	10.1016/j.orggeochem.2010.10.011
$\delta^{34}S$	S	Coal Combustion	7.10‰	39.91	116.39	January, 2011	Active sampling	IRMS	Standard Calibration Method	0.2	10.1016/j.orggeochem.2010.10.011
$\delta^{34}S$	S	Coal Combustion	4.66‰	39.91	116.39	July, 2011	Active sampling	IRMS	Standard Calibration Method	0.2	10.1016/j.orggeochem.2010.10.011
$\delta^{34}S$	S	Coal Combustion	-0.23‰	39.91	116.39	October, 2011	Active sampling	IRMS	Standard Calibration Method	0.2	10.1016/j.orggeochem.2010.10.011
$\delta^{34}S$	S	Coal Combustion	-0.92‰	39.91	116.39	April, 2011	Active sampling	IRMS	Standard Calibration Method	0.2	10.1016/j.orggeochem.2010.10.011
$\delta^{34}S$	S	Coal Combustion	-1.27‰	39.91	116.39	January, 2011	Active sampling	IRMS	Standard Calibration Method	0.2	10.1016/j.orggeochem.2010.10.011
$\delta^{34}S$	S	Coal Combustion	-6.16‰	39.91	116.39	July, 2011	Active sampling	IRMS	Standard Calibration Method	0.2	10.1016/j.orggeochem.2010.10.011
$\delta^{34}S$	S	Coal Combustion	-0.05‰	39.91	116.39	October, 2011	Active sampling	IRMS	Standard Calibration Method	0.2	10.1016/j.orggeochem.2010.10.011
$\delta^{34}S$	S	Coal Combustion	1.95‰	39.91	116.39	April, 2011	Active sampling	IRMS	Standard Calibration Method	0.2	10.1016/j.orggeochem.2010.10.011

$\delta^{34}S$	S	Coal Combustion	3.79‰	39.91	116.39	January, 2011	Active sampling	IRMS	Standard Calibration Method	0.2	10.1016/j.orggeochem.2010.10.011
$\delta^{34}S$	S	Coal Combustion	6.06‰	39.91	116.39	July, 2011	Active sampling	IRMS	Standard Calibration Method	0.2	10.1016/j.orggeochem.2010.10.011
$\delta^{34}S$	S	Coal Combustion	9.20‰	39.91	116.39	October, 2011	Active sampling	IRMS	Standard Calibration Method	0.2	10.1016/j.orggeochem.2010.10.011
$\delta^{34}S$	S	Coal Combustion	12.16‰	39.91	116.39	April, 2011	Active sampling	IRMS	Standard Calibration Method	0.2	10.1016/j.orggeochem.2010.10.011
$\delta^{34}S$	S	Coal Combustion	13.04‰	39.91	116.39	January, 2011	Active sampling	IRMS	Standard Calibration Method	0.2	10.1016/j.orggeochem.2010.10.011
$\delta^{34}S$	S	Coal Combustion	11.64‰	39.91	116.39	July, 2011	Active sampling	IRMS	Standard Calibration Method	0.2	10.1016/j.orggeochem.2010.10.011
$\delta^{34}S$	S	Coal Combustion	15.65‰	39.91	116.39	October, 2011	Active sampling	IRMS	Standard Calibration Method	0.2	10.1016/j.orggeochem.2010.10.011
$\delta^{34}S$	S	Coal Combustion	20.02‰	39.91	116.39	April, 2011	Active sampling	IRMS	Standard Calibration Method	0.2	10.1016/j.orggeochem.2010.10.011
$\delta^{34}S$	S	Coal Combustion	17.57‰	39.91	116.39	January, 2011	Active sampling	IRMS	Standard Calibration Method	0.2	10.1016/j.orggeochem.2010.10.011
$\delta^{34}S$	S	Coal Combustion	15.48‰	39.91	116.39	July, 2011	Active sampling	IRMS	Standard Calibration Method	0.2	10.1016/j.orggeochem.2010.10.011
$\delta^{34}S$	S	Coal Combustion	13.39‰	39.91	116.39	October, 2011	Active sampling	IRMS	Standard Calibration Method	0.2	10.1016/j.orggeochem.2010.10.011
$\delta^{34}S$	S	Coal Combustion	13.73‰	39.91	116.39	April, 2011	Active sampling	IRMS	Standard Calibration Method	0.2	10.1016/j.orggeochem.2010.10.011
$\delta^{34}S$	S	Coal Combustion	14.78‰	39.91	116.39	January, 2011	Active sampling	IRMS	Standard Calibration Method	0.2	10.1016/j.orggeochem.2010.10.011
$\delta^{34}S$	S	Coal Combustion	8.32‰	39.91	116.39	July, 2011	Active sampling	IRMS	Standard Calibration Method	0.2	10.1016/j.orggeochem.2010.10.011
$\delta^{34}S$	S	Coal Combustion	9.90‰	39.91	116.39	October, 2011	Active sampling	IRMS	Standard Calibration Method	0.2	10.1016/j.orggeochem.2010.10.011

$\delta^{34}S$	S	Coal Combustion	8.67‰	39.91	116.39	April, 2011	Active sampling	IRMS	Standard Calibration Method	0.2	10.1016/j.orggeochem.2010.10.011
$\delta^{34}S$	S	Coal Combustion	6.23‰	39.91	116.39	January, 2011	Active sampling	IRMS	Standard Calibration Method	0.2	10.1016/j.orggeochem.2010.10.011
$\delta^{34}S$	S	Coal Combustion	3.61‰	39.91	116.39	July, 2011	Active sampling	IRMS	Standard Calibration Method	0.2	10.1016/j.orggeochem.2010.10.011
$\delta^{34}S$	S	Coal Combustion	7.10‰	39.91	116.39	October, 2011	Active sampling	IRMS	Standard Calibration Method	0.2	10.1016/j.orggeochem.2010.10.011
$\delta^{34}S$	S	Coal Combustion	10.94‰	39.91	116.39	April, 2011	Active sampling	IRMS	Standard Calibration Method	0.2	10.1016/j.orggeochem.2010.10.011
$\delta^{34}S$	S	Coal Combustion	2.39‰	39.91	116.39	January, 2011	Active sampling	IRMS	Standard Calibration Method	0.2	10.1016/j.orggeochem.2010.10.011
$\delta^{34}S$	S	Coal Combustion	0.82‰	39.91	116.39	July, 2011	Active sampling	IRMS	Standard Calibration Method	0.2	10.1016/j.orggeochem.2010.10.011
$\delta^{34}S$	S	Coal Combustion	-0.92‰	39.91	116.39	October, 2011	Active sampling	IRMS	Standard Calibration Method	0.2	10.1016/j.orggeochem.2010.10.011
$\delta^{34}S$	S	Coal Combustion	-2.67‰	39.91	116.39	April, 2011	Active sampling	IRMS	Standard Calibration Method	0.2	10.1016/j.orggeochem.2010.10.011
$\delta^{34}S$	S	Coal Combustion	3.61‰	39.91	116.39	January, 2011	Active sampling	IRMS	Standard Calibration Method	0.2	10.1016/j.orggeochem.2010.10.011
$\delta^{34}S$	S	Coal Combustion	5.01‰	39.91	116.39	July, 2011	Active sampling	IRMS	Standard Calibration Method	0.2	10.1016/j.orggeochem.2010.10.011
$\delta^{34}S$	S	Coal Combustion	5.88‰	39.91	116.39	October, 2011	Active sampling	IRMS	Standard Calibration Method	0.2	10.1016/j.orggeochem.2010.10.011
$\delta^{34}S$	S	Coal Combustion	7.80‰	39.91	116.39	April, 2011	Active sampling	IRMS	Standard Calibration Method	0.2	10.1016/j.orggeochem.2010.10.011
$\delta^{34}S$	S	Coal Combustion	13.04‰	39.91	116.39	January, 2011	Active sampling	IRMS	Standard Calibration Method	0.2	10.1016/j.orggeochem.2010.10.011
$\delta^{34}S$	S	Coal Combustion	12.86‰	39.91	116.39	July, 2011	Active sampling	IRMS	Standard Calibration Method	0.2	10.1016/j.orggeochem.2010.10.011

$\delta^{34}S$	S	Coal Combustion	13.73‰	39.91	116.39	October, 2011	Active sampling	IRMS	Standard Calibration Method	0.2	10.1016/j.orggeochem.2010.10.011
$\delta^{34}S$	S	Coal Combustion	10.77‰	39.91	116.39	April, 2011	Active sampling	IRMS	Standard Calibration Method	0.2	10.1016/j.orggeochem.2010.10.011
$\delta^{34}S$	S	Coal Combustion	10.24‰	39.91	116.39	January, 2011	Active sampling	IRMS	Standard Calibration Method	0.2	10.1016/j.orggeochem.2010.10.011
$\delta^{34}S$	S	Coal Combustion	11.99‰	39.91	116.39	July, 2011	Active sampling	IRMS	Standard Calibration Method	0.2	10.1016/j.orggeochem.2010.10.011
$\delta^{34}S$	S	Coal Combustion	6.23‰	39.91	116.39	October, 2011	Active sampling	IRMS	Standard Calibration Method	0.2	10.1016/j.orggeochem.2010.10.011
$\delta^{34}S$	S	Coal Combustion	1.17‰	39.91	116.39	April, 2011	Active sampling	IRMS	Standard Calibration Method	0.2	10.1016/j.orggeochem.2010.10.011
$\delta^{34}S$	S	Coal Combustion	-3.72‰	39.91	116.39	January, 2011	Active sampling	IRMS	Standard Calibration Method	0.2	10.1016/j.orggeochem.2010.10.011
$\delta^{34}S$	S	Coal Combustion	1.52‰	39.91	116.39	July, 2011	Active sampling	IRMS	Standard Calibration Method	0.2	10.1016/j.orggeochem.2010.10.011
$\delta^{34}S$	S	Coal Combustion	5.71‰	39.91	116.39	October, 2011	Active sampling	IRMS	Standard Calibration Method	0.2	10.1016/j.orggeochem.2010.10.011
$\delta^{34}S$	S	Coal Combustion	8.50‰	39.91	116.39	April, 2011	Active sampling	IRMS	Standard Calibration Method	0.2	10.1016/j.orggeochem.2010.10.011
$\delta^{34}S$	S	Coal Combustion	9.55‰	39.91	116.39	January, 2011	Active sampling	IRMS	Standard Calibration Method	0.2	10.1016/j.orggeochem.2010.10.011
$\delta^{34}S$	S	Coal Combustion	6.75‰	39.91	116.39	July, 2011	Active sampling	IRMS	Standard Calibration Method	0.2	10.1016/j.orggeochem.2010.10.011
$\delta^{34}S$	S	Coal Combustion	3.44‰	39.91	116.39	October, 2011	Active sampling	IRMS	Standard Calibration Method	0.2	10.1016/j.orggeochem.2010.10.011
$\delta^{34}S$	S	Coal Combustion	2.39‰	39.91	116.39	April, 2011	Active sampling	IRMS	Standard Calibration Method	0.2	10.1016/j.orggeochem.2010.10.011
$\delta^{34}S$	S	Coal Combustion	-0.92‰	39.91	116.39	January, 2011	Active sampling	IRMS	Standard Calibration Method	0.2	10.1016/j.orggeochem.2010.10.011

$\delta^{34}S$	S	Coal Combustion	5.71‰	39.91	116.39	July, 2011	Active sampling	IRMS	Standard Calibration Method	0.2	10.1016/j.orggeochem.2010.10.011
$\delta^{34}S$	S	Coal Combustion	6.93‰	39.91	116.39	October, 2011	Active sampling	IRMS	Standard Calibration Method	0.2	10.1016/j.orggeochem.2010.10.011
$\delta^{34}S$	S	Coal Combustion	3.09‰	39.91	116.39	April, 2011	Active sampling	IRMS	Standard Calibration Method	0.2	10.1016/j.orggeochem.2010.10.011
$\delta^{34}S$	S	Coal Combustion	2.91‰	39.91	116.39	January, 2011	Active sampling	IRMS	Standard Calibration Method	0.2	10.1016/j.orggeochem.2010.10.011
$\delta^{34}S$	S	Coal Combustion	1.87‰	39.91	116.39	July, 2011	Active sampling	IRMS	Standard Calibration Method	0.2	10.1016/j.orggeochem.2010.10.011
$\delta^{34}S$	S	Coal Combustion	5.01‰	39.91	116.39	October, 2011	Active sampling	IRMS	Standard Calibration Method	0.2	10.1016/j.orggeochem.2010.10.011
$\delta^{34}S$	S	Coal Combustion	7.63‰	39.91	116.39	April, 2011	Active sampling	IRMS	Standard Calibration Method	0.2	10.1016/j.orggeochem.2010.10.011
$\delta^{34}S$	S	Coal Combustion	7.45‰	39.91	116.39	January, 2011	Active sampling	IRMS	Standard Calibration Method	0.2	10.1016/j.orggeochem.2010.10.011
$\delta^{34}S$	S	Coal Combustion	4.83‰	39.91	116.39	July, 2011	Active sampling	IRMS	Standard Calibration Method	0.2	10.1016/j.orggeochem.2010.10.011
$\delta^{34}S$	S	Coal Combustion	4.66‰	39.91	116.39	October, 2011	Active sampling	IRMS	Standard Calibration Method	0.2	10.1016/j.orggeochem.2010.10.011
$\delta^{34}S$	S	Coal Combustion	15.83‰	39.91	116.39	October, 2011	Active sampling	IRMS	Standard Calibration Method	0.2	10.1016/j.orggeochem.2010.10.011
$\delta^{34}S$	S	Coal Combustion	8.00‰	39.59	116.18	October, 2016	Active sampling	IRMS	Standard Calibration Method	0.2	10.1021/acs.est.7b00280
$\delta^{34}S$	S	Coal Combustion	3.30‰	53.73	-0.98	July, 1990	Active sampling	IRMS	Standard Calibration Method	0.15	10.1029/98JD01664
$\delta^{34}S$	S	Coal Combustion	4.70‰	53.73	-0.98	January, 1992	Active sampling	IRMS	Standard Calibration Method	0.15	10.1029/98JD01664
$\delta^{34}S$	S	Coal Combustion	2.90‰	51.51	-0.15	July, 1994	Active sampling	IRMS	Standard Calibration Method	0.15	10.1029/98JD01664

$\delta^{34}S$	S	Coal Combustion	5.60‰	32.06	118.80	April, 2006	Active sampling	IRMS	Standard Calibration Method	0.2	10.1039/c1em10073d
$\delta^{34}S$	S	Coal Combustion	4.70‰	31.30	120.59	January, 2006	Active sampling	IRMS	Standard Calibration Method	0.2	10.1039/c1em10073d
$\delta^{34}S$	S	Coal Combustion	7.40‰	30.27	120.16	July, 2006	Active sampling	IRMS	Standard Calibration Method	0.2	10.1039/c1em10073d
$\delta^{34}S$	S	Coal Combustion	6.50‰	29.08	119.65	October, 2006	Active sampling	IRMS	Standard Calibration Method	0.2	10.1039/c1em10073d
$\delta^{34}S$	S	Coal Combustion	8.00‰	31.82	117.23	April, 2006	Active sampling	IRMS	Standard Calibration Method	0.2	10.1039/c1em10073d
$\delta^{34}S$	S	Coal Combustion	0.30‰	28.68	115.86	January, 2006	Active sampling	IRMS	Standard Calibration Method	0.2	10.1039/c1em10073d
$\delta^{34}S$	S	Coal Combustion	-3.30‰	28.16	115.77	July, 2006	Active sampling	IRMS	Standard Calibration Method	0.2	10.1039/c1em10073d
$\delta^{34}S$	S	Coal Combustion	2.30‰	28.26	117.07	October, 2006	Active sampling	IRMS	Standard Calibration Method	0.2	10.1039/c1em10073d
$\delta^{34}S$	S	Coal Combustion	2.50‰	28.29	117.21	April, 2006	Active sampling	IRMS	Standard Calibration Method	0.2	10.1039/c1em10073d
$\delta^{34}S$	S	Coal Combustion	3.60‰	25.83	114.94	January, 2006	Active sampling	IRMS	Standard Calibration Method	0.2	10.1039/c1em10073d
$\delta^{34}S$	S	Coal Combustion	7.10‰	29.27	117.18	July, 2006	Active sampling	IRMS	Standard Calibration Method	0.2	10.1039/c1em10073d
$\delta^{34}S$	S	Coal Combustion	6.50‰	30.59	114.31	October, 2006	Active sampling	IRMS	Standard Calibration Method	0.2	10.1039/c1em10073d
$\delta^{34}S$	S	Coal Combustion	5.40‰	30.69	111.29	April, 2006	Active sampling	IRMS	Standard Calibration Method	0.2	10.1039/c1em10073d
$\delta^{34}S$	S	Coal Combustion	5.20‰	28.23	112.94	January, 2006	Active sampling	IRMS	Standard Calibration Method	0.2	10.1039/c1em10073d
$\delta^{34}S$	S	Coal Combustion	-3.10‰	26.65	106.63	July, 2006	Active sampling	IRMS	Standard Calibration Method	0.2	10.1039/c1em10073d

$\delta^{34}S$	S	Coal Combustion	0.50‰	27.73	106.93	October, 2006	Active sampling	IRMS	Standard Calibration Method	0.2	10.1039/c1em10073d
$\delta^{34}S$	S	Coal Combustion	1.80‰	29.56	106.55	January, 2006	Active sampling	IRMS	Standard Calibration Method	0.2	10.1039/c1em10073d
$\delta^{34}S$	S	Coal Combustion	3.30‰	30.57	104.07	October, 2006	Active sampling	IRMS	Standard Calibration Method	0.2	10.1039/c1em10073d
$\delta^{34}S$	S	Coal Combustion	12.90‰	47.35	130.30	April, 2000	Active sampling	VG Iso-gas MS	Standard Calibration Method	0.2	10.1246/nikkashi.2000.45 In Japanese with English abstract
$\delta^{34}S$	S	Coal Combustion	4.90‰	45.80	126.53	January, 2000	Active sampling	VG Iso-gas MS	Standard Calibration Method	0.2	10.1246/nikkashi.2000.45 In Japanese with English abstract
$\delta^{34}S$	S	Coal Combustion	7.60‰	41.88	123.96	July, 2000	Active sampling	VG Iso-gas MS	Standard Calibration Method	0.2	10.1246/nikkashi.2000.45 In Japanese with English abstract
$\delta^{34}S$	S	Coal Combustion	5.30‰	41.87	123.92	October, 2000	Active sampling	VG Iso-gas MS	Standard Calibration Method	0.2	10.1246/nikkashi.2000.45 In Japanese with English abstract
$\delta^{34}S$	S	Coal Combustion	13.60‰	41.87	123.92	April, 2000	Active sampling	VG Iso-gas MS	Standard Calibration Method	0.2	10.1246/nikkashi.2000.45 In Japanese with English abstract
$\delta^{34}S$	S	Coal Combustion	6.40‰	41.87	123.92	January, 2000	Active sampling	VG Iso-gas MS	Standard Calibration Method	0.2	10.1246/nikkashi.2000.45 In Japanese with English abstract
$\delta^{34}S$	S	Coal Combustion	22.80‰	41.87	123.92	July, 2000	Active sampling	VG Iso-gas MS	Standard Calibration Method	0.2	10.1246/nikkashi.2000.45 In Japanese with English abstract
$\delta^{34}S$	S	Coal Combustion	1.90‰	41.87	123.92	October, 2000	Active sampling	VG Iso-gas MS	Standard Calibration Method	0.2	10.1246/nikkashi.2000.45 In Japanese with English abstract
$\delta^{34}S$	S	Coal Combustion	-27.30‰	41.87	123.92	April, 2000	Active sampling	VG Iso-gas MS	Standard Calibration Method	0.2	10.1246/nikkashi.2000.45 In Japanese with English abstract

$\delta^{34}S$	S	Coal Combustion	18.20‰	41.87	123.92	January, 2000	Active sampling	VG Iso-gas MS	Standard Calibration Method	0.2	10.1246/nikkashi.2000.45 In Japanese with English abstract
$\delta^{34}S$	S	Coal Combustion	8.60‰	40.08	113.30	July, 2000	Active sampling	VG Iso-gas MS	Standard Calibration Method	0.2	10.1246/nikkashi.2000.45 In Japanese with English abstract
$\delta^{34}S$	S	Coal Combustion	28.90‰	40.08	113.30	October, 2000	Active sampling	VG Iso-gas MS	Standard Calibration Method	0.2	10.1246/nikkashi.2000.45 In Japanese with English abstract
$\delta^{34}S$	S	Coal Combustion	3.20‰	40.08	113.30	April, 2000	Active sampling	VG Iso-gas MS	Standard Calibration Method	0.2	10.1246/nikkashi.2000.45 In Japanese with English abstract
$\delta^{34}S$	S	Coal Combustion	2.70‰	40.08	113.30	January, 2000	Active sampling	VG Iso-gas MS	Standard Calibration Method	0.2	10.1246/nikkashi.2000.45 In Japanese with English abstract
$\delta^{34}S$	S	Coal Combustion	27.10‰	36.20	113.63	July, 2000	Active sampling	VG Iso-gas MS	Standard Calibration Method	0.2	10.1246/nikkashi.2000.45 In Japanese with English abstract
$\delta^{34}S$	S	Coal Combustion	28.60‰	36.20	113.63	October, 2000	Active sampling	VG Iso-gas MS	Standard Calibration Method	0.2	10.1246/nikkashi.2000.45 In Japanese with English abstract
$\delta^{34}S$	S	Coal Combustion	3.80‰	37.87	112.56	April, 2000	Active sampling	VG Iso-gas MS	Standard Calibration Method	0.2	10.1246/nikkashi.2000.45 In Japanese with English abstract
$\delta^{34}S$	S	Coal Combustion	2.10‰	37.87	112.56	January, 2000	Active sampling	VG Iso-gas MS	Standard Calibration Method	0.2	10.1246/nikkashi.2000.45 In Japanese with English abstract
$\delta^{34}S$	S	Coal Combustion	3.30‰	38.84	110.49	July, 2000	Active sampling	VG Iso-gas MS	Standard Calibration Method	0.2	10.1246/nikkashi.2000.45 In Japanese with English abstract
$\delta^{34}S$	S	Coal Combustion	10.20‰	35.56	116.78	October, 2000	Active sampling	VG Iso-gas MS	Standard Calibration Method	0.2	10.1246/nikkashi.2000.45 In Japanese with English abstract
$\delta^{34}S$	S	Coal Combustion	5.40‰	35.75	114.29	April, 2000	Active sampling	VG Iso-gas MS	Standard Calibration Method	0.2	10.1246/nikkashi.2000.45

											In Japanese with English abstract
$\delta^{34}S$	S	Coal Combustion	0.50‰	36.08	111.52	January, 2000	Active sampling	VG Iso-gas MS	Standard Calibration Method	0.2	10.1246/nikkashi.2000.45 In Japanese with English abstract
$\delta^{34}S$	S	Coal Combustion	0.20‰	41.87	123.92	July, 2000	Active sampling	VG Iso-gas MS	Standard Calibration Method	0.2	10.1246/nikkashi.2000.45 In Japanese with English abstract
$\delta^{34}S$	S	Coal Combustion	3.80‰	41.87	123.92	October, 2000	Active sampling	VG Iso-gas MS	Standard Calibration Method	0.2	10.1246/nikkashi.2000.45 In Japanese with English abstract
$\delta^{34}S$	S	Coal Combustion	3.80‰	31.29	120.59	April, 2000	Active sampling	VG Iso-gas MS	Standard Calibration Method	0.2	10.1246/nikkashi.2000.45 In Japanese with English abstract
$\delta^{34}S$	S	Coal Combustion	2.10‰	31.29	120.59	January, 2000	Active sampling	VG Iso-gas MS	Standard Calibration Method	0.2	10.1246/nikkashi.2000.45 In Japanese with English abstract
$\delta^{34}S$	S	Coal Combustion	-0.30‰	41.87	123.92	July, 2000	Active sampling	VG Iso-gas MS	Standard Calibration Method	0.2	10.1246/nikkashi.2000.45 In Japanese with English abstract
$\delta^{34}S$	S	Coal Combustion	3.10‰	41.87	123.92	October, 2000	Active sampling	VG Iso-gas MS	Standard Calibration Method	0.2	10.1246/nikkashi.2000.45 In Japanese with English abstract
$\delta^{34}S$	S	Coal Combustion	22.30‰	41.87	123.92	April, 2000	Active sampling	VG Iso-gas MS	Standard Calibration Method	0.2	10.1246/nikkashi.2000.45 In Japanese with English abstract
$\delta^{34}S$	S	Coal Combustion	7.20‰	41.87	123.92	January, 2000	Active sampling	VG Iso-gas MS	Standard Calibration Method	0.2	10.1246/nikkashi.2000.45 In Japanese with English abstract
$\delta^{34}S$	S	Coal Combustion	4.10‰	56.67	124.72	July, 2000	Active sampling	VG Iso-gas MS	Standard Calibration Method	0.2	10.1246/nikkashi.2000.45 In Japanese with English abstract
$\delta^{34}S$	S	Coal Combustion	9.40‰	51.50	104.80	October, 2000	Active sampling	VG Iso-gas MS	Standard Calibration Method	0.2	10.1246/nikkashi.2000.45 In Japanese with English abstract

$\delta^{34}S$	S	Coal Combustion	8.80‰	51.54	107.36	April, 2000	Active sampling	VG Iso-gas MS	Standard Calibration Method	0.2	10.1246/nikkashi.2000.45 In Japanese with English abstract
$\delta^{34}S$	S	Coal Combustion	2.60‰	53.11	87.56	January, 2000	Active sampling	VG Iso-gas MS	Standard Calibration Method	0.2	10.1246/nikkashi.2000.45 In Japanese with English abstract
$\delta^{34}S$	S	Coal Combustion	-2.20‰	-2.84	115.29	July, 2000	Active sampling	VG Iso-gas MS	Standard Calibration Method	0.2	10.1246/nikkashi.2000.45 In Japanese with English abstract
$\delta^{34}S$	S	Coal Combustion	13.90‰	-33.49	150.15	October, 2000	Active sampling	VG Iso-gas MS	Standard Calibration Method	0.2	10.1246/nikkashi.2000.45 In Japanese with English abstract
$\delta^{34}S$	S	Coal Combustion	2.40‰	-30.99	150.26	April, 2000	Active sampling	VG Iso-gas MS	Standard Calibration Method	0.2	10.1246/nikkashi.2000.45 In Japanese with English abstract
$\delta^{34}S$	S	Coal Combustion	2.30‰	-32.14	133.68	January, 2000	Active sampling	VG Iso-gas MS	Standard Calibration Method	0.2	10.1246/nikkashi.2000.45 In Japanese with English abstract
$\delta^{34}S$	S	Coal Combustion	3.00‰	-32.14	133.68	July, 2000	Active sampling	VG Iso-gas MS	Standard Calibration Method	0.2	10.1246/nikkashi.2000.45 In Japanese with English abstract
$\delta^{34}S$	S	Coal Combustion	0.20‰	-32.14	133.68	October, 2000	Active sampling	VG Iso-gas MS	Standard Calibration Method	0.2	10.1246/nikkashi.2000.45 In Japanese with English abstract
$\delta^{34}S$	S	Coal Combustion	2.40‰	-32.14	133.68	April, 2000	Active sampling	VG Iso-gas MS	Standard Calibration Method	0.2	10.1246/nikkashi.2000.45 In Japanese with English abstract
$\delta^{34}S$	S	Coal Combustion	17.90‰	32.76	130.76	January, 2000	Active sampling	VG Iso-gas MS	Standard Calibration Method	0.2	10.1246/nikkashi.2000.45 In Japanese with English abstract
$\delta^{34}S$	S	Coal Combustion	9.70‰	43.11	141.95	July, 2000	Active sampling	VG Iso-gas MS	Standard Calibration Method	0.2	10.1246/nikkashi.2000.45 In Japanese with English abstract
$\delta^{34}S$	S	Coal Combustion	8.10‰	23.17	112.58	October, 2002	Active sampling	IRMS	Standard Calibration Method	0.2	10.3321/j.issn:1000-6923.2002.02.017

										In Chinese with English abstract
$\delta^{34}S$	S	Coal Combustion	10.10‰	23.17	112.58	April, 2002	Active sampling	IRMS	Standard Calibration Method	0.2	10.3321/j.issn:1000-6923.2002.02.017 In Chinese with English abstract
$\delta^{34}S$	S	Coal Combustion	10.50‰	23.17	112.58	January, 2002	Active sampling	IRMS	Standard Calibration Method	0.2	10.3321/j.issn:1000-6923.2002.02.017 In Chinese with English abstract
$\delta^{34}S$	S	Coal Combustion	4.10‰	23.17	112.58	July, 2002	Active sampling	IRMS	Standard Calibration Method	0.2	10.3321/j.issn:1000-6923.2002.02.017 In Chinese with English abstract
$\delta^{34}S$	S	Coal Combustion	4.70‰	23.17	112.58	October, 2002	Active sampling	IRMS	Standard Calibration Method	0.2	10.3321/j.issn:1000-6923.2002.02.017 In Chinese with English abstract
$\delta^{34}S$	S	Coal Combustion	5.70‰	23.17	112.58	April, 2002	Active sampling	IRMS	Standard Calibration Method	0.2	10.3321/j.issn:1000-6923.2002.02.017 In Chinese with English abstract
$\delta^{34}S$	S	Coal Combustion	11.80‰	23.17	112.58	January, 2002	Active sampling	IRMS	Standard Calibration Method	0.2	10.3321/j.issn:1000-6923.2002.02.017 In Chinese with English abstract
$\delta^{34}S$	S	Coal Combustion	12.30‰	23.17	112.58	July, 2002	Active sampling	IRMS	Standard Calibration Method	0.2	10.3321/j.issn:1000-6923.2002.02.017 In Chinese with English abstract
$\delta^{34}S$	S	Coal Combustion	12.30‰	23.17	112.58	October, 2002	Active sampling	IRMS	Standard Calibration Method	0.2	10.3321/j.issn:1000-6923.2002.02.017 In Chinese with English abstract
$\delta^{34}S$	S	Coal Combustion	-9.50‰	28.23	112.93	April, 2002	Active sampling	IRMS	Standard Calibration Method	0.2	10.3321/j.issn:1000-6923.2002.02.017 In Chinese with English abstract
$\delta^{34}S$	S	Coal Combustion	-10.60‰	22.82	108.32	January, 2002	Active sampling	IRMS	Standard Calibration Method	0.2	10.3321/j.issn:1000-6923.2002.02.017 In Chinese with English abstract
$\delta^{34}S$	S	Coal Combustion	-13.00‰	28.23	112.93	July, 2002	Active sampling	IRMS	Standard Calibration Method	0.2	10.3321/j.issn:1000-6923.2002.02.017 In Chinese with English abstract

$\delta^{34}S$	S	Coal Combustion	-1.70‰	22.82	108.32	October, 2002	Active sampling	IRMS	Standard Calibration Method	0.2	10.3321/j.issn:1000-6923.2002.02.017 In Chinese with English abstract
$\delta^{34}S$	S	Coal Combustion	5.40‰	28.23	112.93	April, 2002	Active sampling	IRMS	Standard Calibration Method	0.2	10.3321/j.issn:1000-6923.2002.02.017 In Chinese with English abstract
$\delta^{34}S$	S	Coal Combustion	-15.80‰	22.82	108.32	January, 2002	Active sampling	IRMS	Standard Calibration Method	0.2	10.3321/j.issn:1000-6923.2002.02.017 In Chinese with English abstract
$\delta^{34}S$	S	Coal Combustion	-19.40‰	28.23	112.93	July, 2002	Active sampling	IRMS	Standard Calibration Method	0.2	10.3321/j.issn:1000-6923.2002.02.017 In Chinese with English abstract
$\delta^{34}S$	S	Coal Combustion	-16.30‰	22.82	108.32	October, 2002	Active sampling	IRMS	Standard Calibration Method	0.2	10.3321/j.issn:1000-6923.2002.02.017 In Chinese with English abstract
$\delta^{34}S$	S	Coal Combustion	-6.80‰	28.23	112.93	April, 2002	Active sampling	IRMS	Standard Calibration Method	0.2	10.3321/j.issn:1000-6923.2002.02.017 In Chinese with English abstract
$\delta^{34}S$	S	Coal Combustion	1.70‰	22.82	108.32	January, 2002	Active sampling	IRMS	Standard Calibration Method	0.2	10.3321/j.issn:1000-6923.2002.02.017 In Chinese with English abstract
$\delta^{34}S$	S	Coal Combustion	-6.40‰	28.23	112.93	July, 2002	Active sampling	IRMS	Standard Calibration Method	0.2	10.3321/j.issn:1000-6923.2002.02.017 In Chinese with English abstract
$\delta^{34}S$	S	Coal Combustion	-4.30‰	22.82	108.32	October, 2002	Active sampling	IRMS	Standard Calibration Method	0.2	10.3321/j.issn:1000-6923.2002.02.017 In Chinese with English abstract
$\delta^{34}S$	S	Coal Combustion	-4.40‰	28.23	112.93	January, 2002	Active sampling	IRMS	Standard Calibration Method	0.2	10.3321/j.issn:1000-6923.2002.02.017 In Chinese with English abstract
$\delta^{34}S$	S	Coal Combustion	-1.10‰	22.82	108.32	July, 2002	Active sampling	IRMS	Standard Calibration Method	0.2	10.3321/j.issn:1000-6923.2002.02.017 In Chinese with English abstract
$\delta^{34}S$	S	Coal Combustion	10.10‰	22.82	108.32	October, 2002	Active sampling	IRMS	Standard Calibration Method	0.2	10.3321/j.issn:1000-6923.2002.02.017

											In Chinese with English abstract
$\delta^{34}S$	S	Coal Combustion	3.70‰	31.85	117.28	July, 2014	Active sampling	IRMS	Standard Calibration Method	0.2	http://dx.doi.org/10.1016/j.atmosres.2016.01.011
$\delta^{34}S$	S	Coal Combustion	1.80‰	43.88	125.32	July, 2014	Active sampling	IRMS	Standard Calibration Method	0.2	http://dx.doi.org/10.1016/j.atmosres.2016.01.011
$\delta^{34}S$	S	Coal Combustion	3.91‰	32.20	118.72	April, 2015	Active sampling	IRMS	Standard Calibration Method	0.2	http://dx.doi.org/10.1016/j.atmosres.2017.04.034
$\delta^{34}S$	S	Coal Combustion	7.51‰	32.20	118.72	January, 2015	Active sampling	IRMS	Standard Calibration Method	0.2	http://dx.doi.org/10.1016/j.atmosres.2017.04.034
$\delta^{34}S$	S	Coal Combustion	3.65‰	32.20	118.72	July, 2015	Active sampling	IRMS	Standard Calibration Method	0.2	http://dx.doi.org/10.1016/j.atmosres.2017.04.034
$\delta^{34}S$	S	Coal Combustion	3.21‰	32.20	118.72	October, 2015	Active sampling	IRMS	Standard Calibration Method	0.2	http://dx.doi.org/10.1016/j.atmosres.2017.04.034
$\delta^{34}S$	S	Coal Combustion	6.61‰	32.20	118.72	April, 2015	Active sampling	IRMS	Standard Calibration Method	0.2	http://dx.doi.org/10.1016/j.atmosres.2017.04.034
$\delta^{34}S$	S	Coal Combustion	2.89‰	32.20	118.72	January, 2015	Active sampling	IRMS	Standard Calibration Method	0.2	http://dx.doi.org/10.1016/j.atmosres.2017.04.034
$\delta^{34}S$	S	Coal Combustion	3.40‰	32.20	118.72	July, 2015	Active sampling	IRMS	Standard Calibration Method	0.2	http://dx.doi.org/10.1016/j.atmosres.2017.04.034
$\delta^{34}S$	S	Coal Combustion	6.79‰	32.20	118.72	October, 2015	Active sampling	IRMS	Standard Calibration Method	0.2	http://dx.doi.org/10.1016/j.atmosres.2017.04.034
$\delta^{34}S$	S	Coal Combustion	2.95‰	32.20	118.72	April, 2015	Active sampling	IRMS	Standard Calibration Method	0.2	http://dx.doi.org/10.1016/j.atmosres.2017.04.034
$\delta^{34}S$	S	Coal Combustion	7.53‰	32.20	118.72	January, 2015	Active sampling	IRMS	Standard Calibration Method	0.2	http://dx.doi.org/10.1016/j.atmosres.2017.04.034
$\delta^{34}S$	S	Coal Combustion	8.74‰	32.20	118.72	July, 2015	Active sampling	IRMS	Standard Calibration Method	0.2	http://dx.doi.org/10.1016/j.atmosres.2017.04.034
$\delta^{34}S$	S	Coal Combustion	6.45‰	32.20	118.72	October, 2015	Active sampling	IRMS	Standard Calibration Method	0.2	http://dx.doi.org/10.1016/j.atmosres.2017.04.034
$\delta^{34}S$	S	Coal Combustion	6.63‰	32.20	118.72	April, 2015	Active sampling	IRMS	Standard Calibration Method	0.2	http://dx.doi.org/10.1016/j.atmosres.2017.04.034

$\delta^{34}S$	S	Coal Combustion	8.29‰	32.20	118.72	January, 2015	Active sampling	IRMS	Standard Calibration Method	0.2	http://dx.doi.org/10.1016/j.atmosres.2017.04.034
$\delta^{34}S$	S	Coal Combustion	6.15‰	32.20	118.72	July, 2015	Active sampling	IRMS	Standard Calibration Method	0.2	http://dx.doi.org/10.1016/j.atmosres.2017.04.034
$\delta^{34}S$	S	Coal Combustion	-2.88‰	32.20	118.72	October, 2015	Active sampling	IRMS	Standard Calibration Method	0.2	http://dx.doi.org/10.1016/j.atmosres.2017.04.034
$\delta^{34}S$	S	Coal Combustion	2.60‰	32.20	118.72	April, 2015	Active sampling	IRMS	Standard Calibration Method	0.2	http://dx.doi.org/10.1016/j.atmosres.2017.04.034
$\delta^{34}S$	S	Coal Combustion	-2.42‰	32.20	118.72	January, 2015	Active sampling	IRMS	Standard Calibration Method	0.2	http://dx.doi.org/10.1016/j.atmosres.2017.04.034
$\delta^{34}S$	S	Coal Combustion	-1.21‰	32.20	118.72	July, 2015	Active sampling	IRMS	Standard Calibration Method	0.2	http://dx.doi.org/10.1016/j.atmosres.2017.04.034
$\delta^{34}S$	S	Coal Combustion	3.83‰	32.20	118.72	October, 2015	Active sampling	IRMS	Standard Calibration Method	0.2	http://dx.doi.org/10.1016/j.atmosres.2017.04.034
$\delta^{34}S$	S	Coal Combustion	-1.08‰	32.20	118.72	October, 2015	Active sampling	IRMS	Standard Calibration Method	0.2	http://dx.doi.org/10.1016/j.atmosres.2017.04.034
$\delta^{34}S$	S	Coal Combustion	3.10‰	28.70	77.10	April, 2016	Active sampling	IRMS	Standard Calibration Method	0.2	https://doi.org/10.1016/j.apr.2018.12.015
$\delta^{34}S$	S	Coal Combustion	6.54‰	-16.40	-71.53	April, 2005	Active sampling	IRMS	Standard Calibration Method	0.4	https://doi.org/10.1016/j.atmosenv.2021.118482
$\delta^{34}S$	S	Coal Combustion	0.10‰	37.95	139.02	April, 1997	Active sampling	VG Iso10 MS	Standard Calibration Method	0.2	https://doi.org/10.1016/S1352-2310(96)00278-6
$\delta^{34}S$	S	Coal Combustion	2.90‰	37.95	139.02	July, 1997	Active sampling	VG Iso10 MS	Standard Calibration Method	0.2	https://doi.org/10.1016/S1352-2310(96)00278-6
$\delta^{34}S$	S	Coal Combustion	6.40‰	39.91	116.39	January, 1997	Active sampling	VG Iso10 MS	Standard Calibration Method	0.2	https://doi.org/10.1016/S1352-2310(96)00278-6
$\delta^{34}S$	S	Coal Combustion	22.80‰	39.91	116.39	October, 1997	Active sampling	VG Iso10 MS	Standard Calibration Method	0.2	https://doi.org/10.1016/S1352-2310(96)00278-6
$\delta^{34}S$	S	Coal Combustion	7.60‰	39.91	116.39	April, 1997	Active sampling	VG Iso10 MS	Standard Calibration Method	0.2	https://doi.org/10.1016/S1352-2310(96)00278-6

$\delta^{34}S$	S	Coal Combustion	10.50‰	39.91	116.39	July, 1997	Active sampling	VG Iso10 MS	Standard Calibration Method	0.2	https://doi.org/10.1016/S1352-2310(96)00278-6
$\delta^{34}S$	S	Coal Combustion	8.60‰	39.91	116.39	January, 1997	Active sampling	VG Iso10 MS	Standard Calibration Method	0.2	https://doi.org/10.1016/S1352-2310(96)00278-6
$\delta^{34}S$	S	Coal Combustion	3.30‰	39.91	116.39	October, 1997	Active sampling	VG Iso10 MS	Standard Calibration Method	0.2	https://doi.org/10.1016/S1352-2310(96)00278-6
$\delta^{34}S$	S	Coal Combustion	13.60‰	39.91	116.39	April, 1997	Active sampling	VG Iso10 MS	Standard Calibration Method	0.2	https://doi.org/10.1016/S1352-2310(96)00278-6
$\delta^{34}S$	S	Coal Combustion	1.90‰	39.91	116.39	July, 1997	Active sampling	VG Iso10 MS	Standard Calibration Method	0.2	https://doi.org/10.1016/S1352-2310(96)00278-6
$\delta^{34}S$	S	Coal Combustion	4.90‰	39.91	116.39	January, 1997	Active sampling	VG Iso10 MS	Standard Calibration Method	0.2	https://doi.org/10.1016/S1352-2310(96)00278-6
$\delta^{34}S$	S	Coal Combustion	3.10‰	39.91	116.39	October, 1997	Active sampling	VG Iso10 MS	Standard Calibration Method	0.2	https://doi.org/10.1016/S1352-2310(96)00278-6
$\delta^{34}S$	S	Coal Combustion	5.30‰	39.91	116.39	April, 1997	Active sampling	VG Iso10 MS	Standard Calibration Method	0.2	https://doi.org/10.1016/S1352-2310(96)00278-6
$\delta^{34}S$	S	Coal Combustion	22.30‰	39.91	116.39	July, 1997	Active sampling	VG Iso10 MS	Standard Calibration Method	0.2	https://doi.org/10.1016/S1352-2310(96)00278-6
$\delta^{34}S$	S	Coal Combustion	3.80‰	39.91	116.39	January, 1997	Active sampling	VG Iso10 MS	Standard Calibration Method	0.2	https://doi.org/10.1016/S1352-2310(96)00278-6
$\delta^{34}S$	S	Coal Combustion	2.10‰	39.91	116.39	October, 1997	Active sampling	VG Iso10 MS	Standard Calibration Method	0.2	https://doi.org/10.1016/S1352-2310(96)00278-6
$\delta^{34}S$	S	Coal Combustion	28.90‰	39.91	116.39	April, 1997	Active sampling	VG Iso10 MS	Standard Calibration Method	0.2	https://doi.org/10.1016/S1352-2310(96)00278-6
$\delta^{34}S$	S	Coal Combustion	-0.30‰	39.91	116.39	July, 1997	Active sampling	VG Iso10 MS	Standard Calibration Method	0.2	https://doi.org/10.1016/S1352-2310(96)00278-6
$\delta^{34}S$	S	Coal Combustion	-27.30‰	39.91	116.39	January, 1997	Active sampling	VG Iso10 MS	Standard Calibration Method	0.2	https://doi.org/10.1016/S1352-2310(96)00278-6
$\delta^{34}S$	S	Coal Combustion	12.70‰	39.91	116.39	October, 1997	Active sampling	VG Iso10 MS	Standard Calibration Method	0.2	https://doi.org/10.1016/S1352-2310(96)00278-6

$\delta^{34}S$	S	Coal Combustion	25.20‰	39.91	116.39	April, 1997	Active sampling	VG Iso10 MS	Standard Calibration Method	0.2	https://doi.org/10.1016/S1352-2310(96)00278-6
$\delta^{34}S$	S	Coal Combustion	10.00‰	39.91	116.39	July, 1997	Active sampling	VG Iso10 MS	Standard Calibration Method	0.2	https://doi.org/10.1016/S1352-2310(96)00278-6
$\delta^{34}S$	S	Coal Combustion	2.00‰	39.91	116.39	January, 1997	Active sampling	VG Iso10 MS	Standard Calibration Method	0.2	https://doi.org/10.1016/S1352-2310(96)00278-6
$\delta^{34}S$	S	Coal Combustion	16.60‰	39.91	116.39	October, 1997	Active sampling	VG Iso10 MS	Standard Calibration Method	0.2	https://doi.org/10.1016/S1352-2310(96)00278-6
$\delta^{34}S$	S	Coal Combustion	7.70‰	39.91	116.39	April, 1997	Active sampling	VG Iso10 MS	Standard Calibration Method	0.2	https://doi.org/10.1016/S1352-2310(96)00278-6
$\delta^{34}S$	S	Coal Combustion	22.20‰	39.91	116.39	July, 1997	Active sampling	VG Iso10 MS	Standard Calibration Method	0.2	https://doi.org/10.1016/S1352-2310(96)00278-6
$\delta^{34}S$	S	Coal Combustion	8.00‰	39.91	116.39	January, 1997	Active sampling	VG Iso10 MS	Standard Calibration Method	0.2	https://doi.org/10.1016/S1352-2310(96)00278-6
$\delta^{34}S$	S	Coal Combustion	11.90‰	39.91	116.39	October, 1997	Active sampling	VG Iso10 MS	Standard Calibration Method	0.2	https://doi.org/10.1016/S1352-2310(96)00278-6
$\delta^{34}S$	S	Coal Combustion	9.70‰	39.91	116.39	April, 1997	Active sampling	VG Iso10 MS	Standard Calibration Method	0.2	https://doi.org/10.1016/S1352-2310(96)00278-6
$\delta^{34}S$	S	Coal Combustion	3.30‰	39.91	116.39	July, 1997	Active sampling	VG Iso10 MS	Standard Calibration Method	0.2	https://doi.org/10.1016/S1352-2310(96)00278-6
$\delta^{34}S$	S	Coal Combustion	15.20‰	39.91	116.39	January, 1997	Active sampling	VG Iso10 MS	Standard Calibration Method	0.2	https://doi.org/10.1016/S1352-2310(96)00278-6
$\delta^{34}S$	S	Coal Combustion	6.10‰	39.91	116.39	October, 1997	Active sampling	VG Iso10 MS	Standard Calibration Method	0.2	https://doi.org/10.1016/S1352-2310(96)00278-6
$\delta^{34}S$	S	Coal Combustion	3.00‰	39.91	116.39	April, 1997	Active sampling	VG Iso10 MS	Standard Calibration Method	0.2	https://doi.org/10.1016/S1352-2310(96)00278-6
$\delta^{34}S$	S	Coal Combustion	0.20‰	39.91	116.39	July, 1997	Active sampling	VG Iso10 MS	Standard Calibration Method	0.2	https://doi.org/10.1016/S1352-2310(96)00278-6
$\delta^{34}S$	S	Coal Combustion	2.40‰	39.91	116.39	January, 1997	Active sampling	VG Iso10 MS	Standard Calibration Method	0.2	https://doi.org/10.1016/S1352-2310(96)00278-6

$\delta^{34}S$	S	Coal Combustion	3.10‰	39.91	116.39	October, 1997	Active sampling	VG Iso10 MS	Standard Calibration Method	0.2	https://doi.org/10.1016/S1352-2310(96)00278-6
$\delta^{34}S$	S	Coal Combustion	3.70‰	40.09	116.3	April, 2023	Active sampling	IRMS	Standard Calibration Method	0.2	https://doi.org/10.1021/acs.est.3c05072
$\delta^{34}S$	S	Coal Combustion	6.67‰	40.09	116.3	January, 2023	Active sampling	IRMS	Standard Calibration Method	0.2	https://doi.org/10.1021/acs.est.3c05072
$\delta^{34}S$	S	Coal Combustion	12.09‰	40.09	116.3	July, 2023	Active sampling	IRMS	Standard Calibration Method	0.2	https://doi.org/10.1021/acs.est.3c05072
$\delta^{34}S$	S	Coal Combustion	5.39‰	40.09	116.3	October, 2023	Active sampling	IRMS	Standard Calibration Method	0.2	https://doi.org/10.1021/acs.est.3c05072
$\delta^{34}S$	S	Coal Combustion	8.93‰	40.09	116.3	April, 2023	Active sampling	IRMS	Standard Calibration Method	0.2	https://doi.org/10.1021/acs.est.3c05072
$\delta^{34}S$	S	Coal Combustion	30.24‰	40.09	116.3	January, 2023	Active sampling	IRMS	Standard Calibration Method	0.2	https://doi.org/10.1021/acs.est.3c05072
$\delta^{34}S$	S	Coal Combustion	-0.44‰	40.09	116.3	July, 2023	Active sampling	IRMS	Standard Calibration Method	0.2	https://doi.org/10.1021/acs.est.3c05072
$\delta^{34}S$	S	Coal Combustion	8.41‰	40.09	116.3	October, 2023	Active sampling	IRMS	Standard Calibration Method	0.2	https://doi.org/10.1021/acs.est.3c05072
$\delta^{34}S$	S	Coal Combustion	2.94‰	40.09	116.3	April, 2023	Active sampling	IRMS	Standard Calibration Method	0.2	https://doi.org/10.1021/acs.est.3c05072
$\delta^{34}S$	S	Coal Combustion	7.94‰	40.09	116.3	January, 2023	Active sampling	IRMS	Standard Calibration Method	0.2	https://doi.org/10.1021/acs.est.3c05072
$\delta^{34}S$	S	Coal Combustion	5.46‰	40.09	116.3	July, 2023	Active sampling	IRMS	Standard Calibration Method	0.2	https://doi.org/10.1021/acs.est.3c05072
$\delta^{34}S$	S	Coal Combustion	5.57‰	40.09	116.3	October, 2023	Active sampling	IRMS	Standard Calibration Method	0.2	https://doi.org/10.1021/acs.est.3c05072
$\delta^{34}S$	S	Coal Combustion	9.54‰	40.09	116.3	April, 2023	Active sampling	IRMS	Standard Calibration Method	0.2	https://doi.org/10.1021/acs.est.3c05072
$\delta^{34}S$	S	Coal Combustion	4.55‰	40.09	116.3	January, 2023	Active sampling	IRMS	Standard Calibration Method	0.2	https://doi.org/10.1021/acs.est.3c05072

$\delta^{34}S$	S	Coal Combustion	9.81‰	40.09	116.3	July, 2023	Active sampling	IRMS	Standard Calibration Method	0.2	https://doi.org/10.1021/acs.est.3c05072
$\delta^{34}S$	S	Coal Combustion	0.60‰	39.91	116.39	October, 2023	Active sampling	VG Iso10 MS	Standard Calibration Method	0.2	https://doi.org/10.1016/S1352-2310(96)00278-6
$\delta^{34}S$	S	Vehicle Exhausts	9.70‰	32.10	118.50	July, 2014	Active sampling	IRMS	Standard Calibration Method	0.2	http://dx.doi.org/10.1016/j.atmosres.2016.01.011
$\delta^{34}S$	S	Vehicle Exhausts	4.60‰	32.10	118.50	July, 2014	Active sampling	IRMS	Standard Calibration Method	0.2	http://dx.doi.org/10.1016/j.atmosres.2016.01.011
$\delta^{34}S$	S	Vehicle Exhausts	4.70‰	32.10	118.50	July, 2014	Active sampling	IRMS	Standard Calibration Method	0.2	http://dx.doi.org/10.1016/j.atmosres.2016.01.011
$\delta^{34}S$	S	Vehicle Exhausts	2.10‰	28.70	77.10	April, 2016	Active sampling	IRMS	Standard Calibration Method	0.2	https://doi.org/10.1016/j.apr.2018.12.015
$\delta^{34}S$	S	Natural Soil	7.40‰	-16.40	-71.53	January, 2005	Active sampling	IRMS	Standard Calibration Method	0.4	https://doi.org/10.1016/j.atmosenv.2021.118482
$\delta^{34}S$	S	Natural Soil	7.31‰	-16.40	-71.53	July, 2005	Active sampling	IRMS	Standard Calibration Method	0.4	https://doi.org/10.1016/j.atmosenv.2021.118482
$\delta^{34}S$	S	Natural Soil	3.30‰	28.67	115.90	January, 2015	Active sampling	IRMS	Standard Calibration Method	0.2	https://doi.org/10.1016/S1002-0160(14)60084-9
$\delta^{34}S$	S	Natural Soil	3.16‰	28.67	115.90	April, 2015	Active sampling	IRMS	Standard Calibration Method	0.2	https://doi.org/10.1016/S1002-0160(14)60084-9
$\delta^{34}S$	S	Natural Soil	10.27‰	28.67	115.90	July, 2015	Active sampling	IRMS	Standard Calibration Method	0.2	https://doi.org/10.1016/S1002-0160(14)60084-9
$\delta^{34}S$	S	Natural Soil	4.05‰	24.47	118.08	October, 2015	Active sampling	IRMS	Standard Calibration Method	0.2	https://doi.org/10.1016/S1002-0160(14)60084-9
$\delta^{34}S$	S	Natural Soil	3.62‰	24.47	118.08	January, 2015	Active sampling	IRMS	Standard Calibration Method	0.2	https://doi.org/10.1016/S1002-0160(14)60084-9
$\delta^{34}S$	S	Natural Soil	8.04‰	24.47	118.08	April, 2015	Active sampling	IRMS	Standard Calibration Method	0.2	https://doi.org/10.1016/S1002-0160(14)60084-9
$\delta^{34}S$	S	Natural Soil	-2.31‰	26.57	106.73	July, 2015	Active sampling	IRMS	Standard Calibration Method	0.2	https://doi.org/10.1016/S1002-0160(14)60084-9

$\delta^{34}S$	S	Natural Soil	-2.28‰	26.57	106.73	October, 2015	Active sampling	IRMS	Standard Calibration Method	0.2	https://doi.org/10.1016/S1002-0160(14)60084-9
$\delta^{34}S$	S	Natural Soil	5.45‰	26.57	106.73	January, 2015	Active sampling	IRMS	Standard Calibration Method	0.2	https://doi.org/10.1016/S1002-0160(14)60084-9
$\delta^{34}S$	S	Natural Soil	3.46‰	39.93	119.58	April, 2015	Active sampling	IRMS	Standard Calibration Method	0.2	https://doi.org/10.1016/S1002-0160(14)60084-9
$\delta^{34}S$	S	Natural Soil	4.31‰	39.93	119.58	July, 2015	Active sampling	IRMS	Standard Calibration Method	0.2	https://doi.org/10.1016/S1002-0160(14)60084-9
$\delta^{34}S$	S	Natural Soil	8.93‰	39.93	119.58	October, 2015	Active sampling	IRMS	Standard Calibration Method	0.2	https://doi.org/10.1016/S1002-0160(14)60084-9
$\delta^{34}S$	S	Natural Soil	11.70‰	51.05	13.73	November, 1996-June, 1998	Active sampling	-	Standard Calibration Method	0.3	https://doi.org/10.1080/10256010701702499
$\delta^{34}S$	S	Natural Soil	20.50‰	39.59	116.18	January, 2010	Active sampling	IRMS	Standard Calibration Method	0.3	http://dx.doi.org/10.1016/j.gexplo.2015.11.010
$\delta^{34}S$	S	Natural Soil	31.30‰	39.59	116.18	April, 2010	Active sampling	IRMS	Standard Calibration Method	0.3	http://dx.doi.org/10.1016/j.gexplo.2015.11.010
$\delta^{34}S$	S	Natural Soil	29.60‰	39.59	116.18	July, 2010	Active sampling	IRMS	Standard Calibration Method	0.3	http://dx.doi.org/10.1016/j.gexplo.2015.11.010
$\delta^{34}S$	S	Natural Soil	21.10‰	39.59	116.18	October, 2010	Active sampling	IRMS	Standard Calibration Method	0.3	http://dx.doi.org/10.1016/j.gexplo.2015.11.010
$\delta^{34}S$	S	Natural Soil	22.00‰	39.59	116.18	December, 2010	Active sampling	IRMS	Standard Calibration Method	0.3	http://dx.doi.org/10.1016/j.gexplo.2015.11.010
$\delta^{34}S$	S	Natural Soil	24.00‰	39.59	116.18	January, 2010	Active sampling	IRMS	Standard Calibration Method	0.3	http://dx.doi.org/10.1016/j.gexplo.2015.11.010
$\delta^{34}S$	S	Natural Soil	20.50‰	39.59	116.18	April, 2010	Active sampling	IRMS	Standard Calibration Method	0.3	http://dx.doi.org/10.1016/j.gexplo.2015.11.010
$\delta^{34}S$	S	Natural Soil	19.40‰	39.59	116.18	July, 2010	Active sampling	IRMS	Standard Calibration Method	0.3	http://dx.doi.org/10.1016/j.gexplo.2015.11.010
$\delta^{34}S$	S	Natural Soil	18.40‰	39.59	116.18	October, 2010	Active sampling	IRMS	Standard Calibration Method	0.3	http://dx.doi.org/10.1016/j.gexplo.2015.11.010

$\delta^{34}S$	S	Natural Soil	16.60‰	39.59	116.18	December, 2010	Active sampling	IRMS	Standard Calibration Method	0.3	http://dx.doi.org/10.1016/j.gexplo.2015.11.010
$\delta^{34}S$	S	Natural Soil	4.90‰	39.59	116.18	January, 2010	Active sampling	IRMS	Standard Calibration Method	0.3	http://dx.doi.org/10.1016/j.gexplo.2015.11.010
$\delta^{34}S$	S	Natural Soil	6.10‰	39.59	116.18	April, 2010	Active sampling	IRMS	Standard Calibration Method	0.3	http://dx.doi.org/10.1016/j.gexplo.2015.11.010
$\delta^{34}S$	S	Natural Soil	6.20‰	39.59	116.18	July, 2010	Active sampling	IRMS	Standard Calibration Method	0.3	http://dx.doi.org/10.1016/j.gexplo.2015.11.010
$\delta^{34}S$	S	Natural Soil	3.90‰	39.59	116.18	October, 2010	Active sampling	IRMS	Standard Calibration Method	0.3	http://dx.doi.org/10.1016/j.gexplo.2015.11.010
$\delta^{34}S$	S	Natural Soil	2.90‰	39.59	116.18	December, 2010	Active sampling	IRMS	Standard Calibration Method	0.3	http://dx.doi.org/10.1016/j.gexplo.2015.11.010
$\delta^{34}S$	S	Natural Soil	1.80‰	39.59	116.18	January, 2010	Active sampling	IRMS	Standard Calibration Method	0.3	http://dx.doi.org/10.1016/j.gexplo.2015.11.010
$\delta^{34}S$	S	Natural Soil	1.81‰	39.59	116.18	April, 2010	Active sampling	IRMS	Standard Calibration Method	0.3	http://dx.doi.org/10.1016/j.gexplo.2015.11.010
$\delta^{34}S$	S	Natural Soil	2.27‰	39.59	116.18	July, 2010	Active sampling	IRMS	Standard Calibration Method	0.3	http://dx.doi.org/10.1016/j.gexplo.2015.11.010
$\delta^{34}S$	S	Natural Soil	5.78‰	39.59	116.18	October, 2010	Active sampling	IRMS	Standard Calibration Method	0.3	http://dx.doi.org/10.1016/j.gexplo.2015.11.010
$\delta^{34}S$	S	Natural Soil	7.92‰	39.59	116.18	December, 2010	Active sampling	IRMS	Standard Calibration Method	0.3	http://dx.doi.org/10.1016/j.gexplo.2015.11.010
$\delta^{34}S$	S	Natural Soil	6.91‰	39.59	116.18	January, 2010	Active sampling	IRMS	Standard Calibration Method	0.3	http://dx.doi.org/10.1016/j.gexplo.2015.11.010
$\delta^{34}S$	S	Natural Soil	9.02‰	39.59	116.18	April, 2010	Active sampling	IRMS	Standard Calibration Method	0.3	http://dx.doi.org/10.1016/j.gexplo.2015.11.010
$\delta^{34}S$	S	Natural Soil	10.17‰	39.59	116.18	July, 2010	Active sampling	IRMS	Standard Calibration Method	0.3	http://dx.doi.org/10.1016/j.gexplo.2015.11.010
$\delta^{34}S$	S	Natural Soil	26.42‰	39.59	116.18	October, 2010	Active sampling	IRMS	Standard Calibration Method	0.3	http://dx.doi.org/10.1016/j.gexplo.2015.11.010

$\delta^{34}S$	S	Natural Soil	21.77‰	39.59	116.18	December, 2010	Active sampling	IRMS	Standard Calibration Method	0.3	http://dx.doi.org/10.1016/j.gexplo.2015.11.010
$\delta^{34}S$	S	Natural Soil	12.97‰	39.59	116.18	January, 2010	Active sampling	IRMS	Standard Calibration Method	0.3	http://dx.doi.org/10.1016/j.gexplo.2015.11.010
$\delta^{34}S$	S	Natural Soil	7.68‰	39.59	116.18	April, 2010	Active sampling	IRMS	Standard Calibration Method	0.3	http://dx.doi.org/10.1016/j.gexplo.2015.11.010
$\delta^{34}S$	S	Natural Soil	6.92‰	39.59	116.18	July, 2010	Active sampling	IRMS	Standard Calibration Method	0.3	http://dx.doi.org/10.1016/j.gexplo.2015.11.010
$\delta^{34}S$	S	Natural Soil	6.78‰	39.59	116.18	October, 2010	Active sampling	IRMS	Standard Calibration Method	0.3	http://dx.doi.org/10.1016/j.gexplo.2015.11.010
$\delta^{34}S$	S	Natural Soil	6.58‰	39.59	116.18	December, 2010	Active sampling	IRMS	Standard Calibration Method	0.3	http://dx.doi.org/10.1016/j.gexplo.2015.11.010
$\delta^{34}S$	S	Natural Soil	6.04‰	39.59	116.18	January, 2010	Active sampling	IRMS	Standard Calibration Method	0.3	http://dx.doi.org/10.1016/j.gexplo.2015.11.010
$\delta^{34}S$	S	Natural Soil	6.57‰	39.59	116.18	April, 2010	Active sampling	IRMS	Standard Calibration Method	0.3	http://dx.doi.org/10.1016/j.gexplo.2015.11.010
$\delta^{34}S$	S	Natural Soil	5.50‰	39.59	116.18	July, 2010	Active sampling	IRMS	Standard Calibration Method	0.3	http://dx.doi.org/10.1016/j.gexplo.2015.11.010
$\delta^{34}S$	S	Natural Soil	6.75‰	39.59	116.18	October, 2010	Active sampling	IRMS	Standard Calibration Method	0.3	http://dx.doi.org/10.1016/j.gexplo.2015.11.010
$\delta^{34}S$	S	Natural Soil	6.40‰	39.59	116.18	December, 2010	Active sampling	IRMS	Standard Calibration Method	0.3	http://dx.doi.org/10.1016/j.gexplo.2015.11.010
$\delta^{34}S$	S	Natural Soil	6.04‰	39.59	116.18	January, 2010	Active sampling	IRMS	Standard Calibration Method	0.3	http://dx.doi.org/10.1016/j.gexplo.2015.11.010
$\delta^{34}S$	S	Natural Soil	6.32‰	39.59	116.18	April, 2010	Active sampling	IRMS	Standard Calibration Method	0.3	http://dx.doi.org/10.1016/j.gexplo.2015.11.010
$\delta^{34}S$	S	Natural Soil	6.23‰	39.59	116.18	July, 2010	Active sampling	IRMS	Standard Calibration Method	0.3	http://dx.doi.org/10.1016/j.gexplo.2015.11.010
$\delta^{34}S$	S	Natural Soil	6.94‰	39.59	116.18	October, 2010	Active sampling	IRMS	Standard Calibration Method	0.3	http://dx.doi.org/10.1016/j.gexplo.2015.11.010

$\delta^{34}S$	S	Natural Soil	6.48‰	39.59	116.18	December, 2010	Active sampling	IRMS	Standard Calibration Method	0.3	http://dx.doi.org/10.1016/j.gexplo.2015.11.010
$\delta^{34}S$	S	Natural Soil	6.61‰	39.59	116.18	January, 2010	Active sampling	IRMS	Standard Calibration Method	0.3	http://dx.doi.org/10.1016/j.gexplo.2015.11.010
$\delta^{34}S$	S	Natural Soil	6.46‰	39.59	116.18	April, 2010	Active sampling	IRMS	Standard Calibration Method	0.3	http://dx.doi.org/10.1016/j.gexplo.2015.11.010
$\delta^{34}S$	S	Natural Soil	6.57‰	39.59	116.18	July, 2010	Active sampling	IRMS	Standard Calibration Method	0.3	http://dx.doi.org/10.1016/j.gexplo.2015.11.010
$\delta^{34}S$	S	Natural Soil	6.77‰	39.59	116.18	October, 2010	Active sampling	IRMS	Standard Calibration Method	0.3	http://dx.doi.org/10.1016/j.gexplo.2015.11.010
$\delta^{34}S$	S	Natural Soil	6.94‰	39.59	116.18	December, 2010	Active sampling	IRMS	Standard Calibration Method	0.3	http://dx.doi.org/10.1016/j.gexplo.2015.11.010
$\delta^{34}S$	S	Natural Soil	6.00‰	39.73	116.55	January, 2010	Active sampling	IRMS	Standard Calibration Method	0.3	http://dx.doi.org/10.1016/j.gexplo.2015.11.010
$\delta^{34}S$	S	Natural Soil	8.00‰	39.73	116.55	April, 2010	Active sampling	IRMS	Standard Calibration Method	0.3	http://dx.doi.org/10.1016/j.gexplo.2015.11.010
$\delta^{34}S$	S	Natural Soil	6.50‰	39.73	116.55	July, 2010	Active sampling	IRMS	Standard Calibration Method	0.3	http://dx.doi.org/10.1016/j.gexplo.2015.11.010
$\delta^{34}S$	S	Natural Soil	7.50‰	39.73	116.55	October, 2010	Active sampling	IRMS	Standard Calibration Method	0.3	http://dx.doi.org/10.1016/j.gexplo.2015.11.010
$\delta^{34}S$	S	Natural Soil	7.60‰	39.73	116.55	December, 2010	Active sampling	IRMS	Standard Calibration Method	0.3	http://dx.doi.org/10.1016/j.gexplo.2015.11.010
$\delta^{34}S$	S	Natural Soil	6.00‰	39.73	116.55	January, 2010	Active sampling	IRMS	Standard Calibration Method	0.3	http://dx.doi.org/10.1016/j.gexplo.2015.11.010
$\delta^{34}S$	S	Natural Soil	7.00‰	39.70	116.78	April, 2010	Active sampling	IRMS	Standard Calibration Method	0.3	http://dx.doi.org/10.1016/j.gexplo.2015.11.010
$\delta^{34}S$	S	Natural Soil	9.20‰	39.70	116.78	July, 2010	Active sampling	IRMS	Standard Calibration Method	0.3	http://dx.doi.org/10.1016/j.gexplo.2015.11.010
$\delta^{34}S$	S	Natural Soil	7.20‰	39.70	116.78	October, 2010	Active sampling	IRMS	Standard Calibration Method	0.3	http://dx.doi.org/10.1016/j.gexplo.2015.11.010

$\delta^{34}S$	S	Natural Soil	8.30‰	39.70	116.78	December, 2010	Active sampling	IRMS	Standard Calibration Method	0.3	http://dx.doi.org/10.1016/j.gexplo.2015.11.010
$\delta^{34}S$	S	Natural Soil	9.10‰	39.70	116.78	January, 2010	Active sampling	IRMS	Standard Calibration Method	0.3	http://dx.doi.org/10.1016/j.gexplo.2015.11.010
$\delta^{34}S$	S	Natural Soil	8.80‰	39.70	116.78	April, 2010	Active sampling	IRMS	Standard Calibration Method	0.3	http://dx.doi.org/10.1016/j.gexplo.2015.11.010
$\delta^{34}S$	S	Natural Soil	8.20‰	39.70	116.78	July, 2010	Active sampling	IRMS	Standard Calibration Method	0.3	http://dx.doi.org/10.1016/j.gexplo.2015.11.010
$\delta^{34}S$	S	Natural Soil	7.60‰	39.70	116.78	October, 2010	Active sampling	IRMS	Standard Calibration Method	0.3	http://dx.doi.org/10.1016/j.gexplo.2015.11.010
$\delta^{34}S$	S	Natural Soil	8.10‰	39.70	116.78	December, 2010	Active sampling	IRMS	Standard Calibration Method	0.3	http://dx.doi.org/10.1016/j.gexplo.2015.11.010
$\delta^{34}S$	S	Natural Soil	7.20‰	39.70	116.78	January, 2010	Active sampling	IRMS	Standard Calibration Method	0.3	http://dx.doi.org/10.1016/j.gexplo.2015.11.010
$\delta^{34}S$	S	Natural Soil	7.25‰	39.70	115.93	April, 2010	Active sampling	IRMS	Standard Calibration Method	0.3	http://dx.doi.org/10.1016/j.gexplo.2015.11.010
$\delta^{34}S$	S	Natural Soil	7.29‰	39.70	115.93	July, 2010	Active sampling	IRMS	Standard Calibration Method	0.3	http://dx.doi.org/10.1016/j.gexplo.2015.11.010
$\delta^{34}S$	S	Natural Soil	7.66‰	39.70	115.93	October, 2010	Active sampling	IRMS	Standard Calibration Method	0.3	http://dx.doi.org/10.1016/j.gexplo.2015.11.010
$\delta^{34}S$	S	Natural Soil	7.09‰	39.70	115.93	December, 2010	Active sampling	IRMS	Standard Calibration Method	0.3	http://dx.doi.org/10.1016/j.gexplo.2015.11.010
$\delta^{34}S$	S	Natural Soil	7.74‰	39.70	115.93	January, 2010	Active sampling	IRMS	Standard Calibration Method	0.3	http://dx.doi.org/10.1016/j.gexplo.2015.11.010
$\delta^{34}S$	S	Natural Soil	7.37‰	39.70	115.93	April, 2010	Active sampling	IRMS	Standard Calibration Method	0.3	http://dx.doi.org/10.1016/j.gexplo.2015.11.010
$\delta^{34}S$	S	Natural Soil	7.54‰	39.70	115.93	July, 2010	Active sampling	IRMS	Standard Calibration Method	0.3	http://dx.doi.org/10.1016/j.gexplo.2015.11.010
$\delta^{34}S$	S	Natural Soil	8.20‰	39.70	115.93	October, 2010	Active sampling	IRMS	Standard Calibration Method	0.3	http://dx.doi.org/10.1016/j.gexplo.2015.11.010

$\delta^{34}S$	S	Natural Soil	8.01‰	39.70	115.93	December, 2010	Active sampling	IRMS	Standard Calibration Method	0.3	http://dx.doi.org/10.1016/j.gexplo.2015.11.010
$\delta^{34}S$	S	Natural Soil	7.97‰	39.70	115.93	January, 2010	Active sampling	IRMS	Standard Calibration Method	0.3	http://dx.doi.org/10.1016/j.gexplo.2015.11.010
$\delta^{34}S$	S	Natural Soil	7.59‰	39.70	115.93	April, 2010	Active sampling	IRMS	Standard Calibration Method	0.3	http://dx.doi.org/10.1016/j.gexplo.2015.11.010
$\delta^{34}S$	S	Natural Soil	12.60‰	39.59	116.18	July, 2010	Active sampling	IRMS	Standard Calibration Method	0.3	http://dx.doi.org/10.1016/j.gexplo.2015.11.010
$\delta^{34}S$	S	Natural Soil	9.40‰	39.59	116.18	October, 2010	Active sampling	IRMS	Standard Calibration Method	0.3	http://dx.doi.org/10.1016/j.gexplo.2015.11.010
$\delta^{34}S$	S	Natural Soil	9.90‰	39.59	116.18	December, 2010	Active sampling	IRMS	Standard Calibration Method	0.3	http://dx.doi.org/10.1016/j.gexplo.2015.11.010
$\delta^{34}S$	S	Natural Soil	14.00‰	39.59	116.18	January, 2010	Active sampling	IRMS	Standard Calibration Method	0.3	http://dx.doi.org/10.1016/j.gexplo.2015.11.010
$\delta^{34}S$	S	Natural Soil	7.60‰	39.59	116.18	April, 2010	Active sampling	IRMS	Standard Calibration Method	0.3	http://dx.doi.org/10.1016/j.gexplo.2015.11.010
$\delta^{34}S$	S	Natural Soil	10.30‰	39.59	116.18	July, 2010	Active sampling	IRMS	Standard Calibration Method	0.3	http://dx.doi.org/10.1016/j.gexplo.2015.11.010
$\delta^{34}S$	S	Natural Soil	3.40‰	39.59	116.18	October, 2010	Active sampling	IRMS	Standard Calibration Method	0.3	http://dx.doi.org/10.1016/j.gexplo.2015.11.010
$\delta^{34}S$	S	Natural Soil	2.50‰	39.59	116.18	December, 2010	Active sampling	IRMS	Standard Calibration Method	0.3	http://dx.doi.org/10.1016/j.gexplo.2015.11.010
$\delta^{34}S$	S	Natural Soil	-0.80‰	39.59	116.18	January, 2010	Active sampling	IRMS	Standard Calibration Method	0.3	http://dx.doi.org/10.1016/j.gexplo.2015.11.010
$\delta^{34}S$	S	Natural Soil	-5.20‰	39.59	116.18	April, 2010	Active sampling	IRMS	Standard Calibration Method	0.3	http://dx.doi.org/10.1016/j.gexplo.2015.11.010
$\delta^{34}S$	S	Natural Soil	-4.90‰	39.59	116.18	July, 2010	Active sampling	IRMS	Standard Calibration Method	0.3	http://dx.doi.org/10.1016/j.gexplo.2015.11.010

$\delta^{34}S$	S	Natural Soil	-6.80‰	39.59	116.18	October, 2010	Active sampling	IRMS	Standard Calibration Method	0.3	http://dx.doi.org/10.1016/j.gexplo.2015.11.010
$\delta^{34}S$	S	Natural Soil	2.00‰	39.73	116.55	December, 2010	Active sampling	IRMS	Standard Calibration Method	0.3	http://dx.doi.org/10.1016/j.gexplo.2015.11.010
$\delta^{34}S$	S	Natural Soil	2.90‰	39.73	116.55	January, 2010	Active sampling	IRMS	Standard Calibration Method	0.3	http://dx.doi.org/10.1016/j.gexplo.2015.11.010
$\delta^{34}S$	S	Natural Soil	1.70‰	39.73	116.55	April, 2010	Active sampling	IRMS	Standard Calibration Method	0.3	http://dx.doi.org/10.1016/j.gexplo.2015.11.010
$\delta^{34}S$	S	Natural Soil	4.50‰	39.73	116.55	July, 2010	Active sampling	IRMS	Standard Calibration Method	0.3	http://dx.doi.org/10.1016/j.gexplo.2015.11.010
$\delta^{34}S$	S	Natural Soil	4.40‰	39.73	116.55	October, 2010	Active sampling	IRMS	Standard Calibration Method	0.3	http://dx.doi.org/10.1016/j.gexplo.2015.11.010
$\delta^{34}S$	S	Natural Soil	6.10‰	39.73	116.55	December, 2010	Active sampling	IRMS	Standard Calibration Method	0.3	http://dx.doi.org/10.1016/j.gexplo.2015.11.010
$\delta^{34}S$	S	Natural Soil	4.80‰	39.73	116.55	January, 2010	Active sampling	IRMS	Standard Calibration Method	0.3	http://dx.doi.org/10.1016/j.gexplo.2015.11.010
$\delta^{34}S$	S	Natural Soil	6.00‰	39.73	116.55	April, 2010	Active sampling	IRMS	Standard Calibration Method	0.3	http://dx.doi.org/10.1016/j.gexplo.2015.11.010
$\delta^{34}S$	S	Natural Soil	5.80‰	39.70	116.78	July, 2010	Active sampling	IRMS	Standard Calibration Method	0.3	http://dx.doi.org/10.1016/j.gexplo.2015.11.010
$\delta^{34}S$	S	Natural Soil	3.50‰	39.70	116.78	October, 2010	Active sampling	IRMS	Standard Calibration Method	0.3	http://dx.doi.org/10.1016/j.gexplo.2015.11.010
$\delta^{34}S$	S	Natural Soil	5.00‰	39.70	116.78	December, 2010	Active sampling	IRMS	Standard Calibration Method	0.3	http://dx.doi.org/10.1016/j.gexplo.2015.11.010
$\delta^{34}S$	S	Natural Soil	4.50‰	39.70	116.78	January, 2010	Active sampling	IRMS	Standard Calibration Method	0.3	http://dx.doi.org/10.1016/j.gexplo.2015.11.010
$\delta^{34}S$	S	Natural Soil	5.60‰	39.70	116.78	April, 2010	Active sampling	IRMS	Standard Calibration Method	0.3	http://dx.doi.org/10.1016/j.gexplo.2015.11.010

$\delta^{34}S$	S	Natural Soil	1.10‰	39.70	116.78	July, 2010	Active sampling	IRMS	Standard Calibration Method	0.3	http://dx.doi.org/10.1016/j.gexplo.2015.11.010
$\delta^{34}S$	S	Natural Soil	-7.10‰	39.70	116.78	October, 2010	Active sampling	IRMS	Standard Calibration Method	0.3	http://dx.doi.org/10.1016/j.gexplo.2015.11.010
$\delta^{34}S$	S	Natural Soil	5.00‰	39.70	116.78	December, 2010	Active sampling	IRMS	Standard Calibration Method	0.3	http://dx.doi.org/10.1016/j.gexplo.2015.11.010
$\delta^{34}S$	S	Natural Soil	-4.30‰	39.70	116.78	January, 2010	Active sampling	IRMS	Standard Calibration Method	0.3	http://dx.doi.org/10.1016/j.gexplo.2015.11.010
$\delta^{34}S$	S	Natural Soil	-0.50‰	39.70	116.78	April, 2010	Active sampling	IRMS	Standard Calibration Method	0.3	http://dx.doi.org/10.1016/j.gexplo.2015.11.010
$\delta^{34}S$	S	Natural Soil	-0.70‰	39.70	116.78	July, 2010	Active sampling	IRMS	Standard Calibration Method	0.3	http://dx.doi.org/10.1016/j.gexplo.2015.11.010
$\delta^{34}S$	S	Natural Soil	4.90‰	39.70	116.78	October, 2010	Active sampling	IRMS	Standard Calibration Method	0.3	http://dx.doi.org/10.1016/j.gexplo.2015.11.010
$\delta^{34}S$	S	Natural Soil	4.90‰	39.70	115.93	December, 2010	Active sampling	IRMS	Standard Calibration Method	0.3	http://dx.doi.org/10.1016/j.gexplo.2015.11.010
$\delta^{34}S$	S	Natural Soil	9.00‰	39.70	115.93	January, 2010	Active sampling	IRMS	Standard Calibration Method	0.3	http://dx.doi.org/10.1016/j.gexplo.2015.11.010
$\delta^{34}S$	S	Natural Soil	8.15‰	39.70	115.93	April, 2010	Active sampling	IRMS	Standard Calibration Method	0.3	http://dx.doi.org/10.1016/j.gexplo.2015.11.010
$\delta^{34}S$	S	Natural Soil	2.80‰	39.59	116.18	July, 2010	Active sampling	IRMS	Standard Calibration Method	0.3	http://dx.doi.org/10.1016/j.gexplo.2015.11.010
$\delta^{34}S$	S	Natural Soil	6.30‰	39.59	116.18	October, 2010	Active sampling	IRMS	Standard Calibration Method	0.3	http://dx.doi.org/10.1016/j.gexplo.2015.11.010
$\delta^{34}S$	S	Natural Soil	6.50‰	39.73	116.55	December, 2010	Active sampling	IRMS	Standard Calibration Method	0.3	http://dx.doi.org/10.1016/j.gexplo.2015.11.010
$\delta^{34}S$	S	Natural Soil	5.60‰	39.70	116.78	January, 2010	Active sampling	IRMS	Standard Calibration Method	0.3	http://dx.doi.org/10.1016/j.gexplo.2015.11.010

$\delta^{34}S$	S	Natural Soil	4.30‰	39.70	116.78	April, 2010	Active sampling	IRMS	Standard Calibration Method	0.3	http://dx.doi.org/10.1016/j.gexplo.2015.11.010
$\delta^{34}S$	S	Natural Soil	4.89‰	39.70	115.93	July, 2010	Active sampling	IRMS	Standard Calibration Method	0.3	http://dx.doi.org/10.1016/j.gexplo.2015.11.010
$\delta^{34}S$	S	Natural Soil	4.55‰	39.70	115.93	October, 2010	Active sampling	IRMS	Standard Calibration Method	0.3	http://dx.doi.org/10.1016/j.gexplo.2015.11.010
$\delta^{34}S$	S	Natural Soil	0.62‰	39.70	115.93	December, 2010	Active sampling	IRMS	Standard Calibration Method	0.3	http://dx.doi.org/10.1016/j.gexplo.2015.11.010
$\delta^{34}S$	S	Natural Soil	0.44‰	39.70	115.93	January, 2010	Active sampling	IRMS	Standard Calibration Method	0.3	http://dx.doi.org/10.1016/j.gexplo.2015.11.010
$\delta^{34}S$	S	Natural Soil	4.15‰	39.70	115.93	April, 2010	Active sampling	IRMS	Standard Calibration Method	0.3	http://dx.doi.org/10.1016/j.gexplo.2015.11.010
$\delta^{34}S$	S	Natural Soil	4.14‰	39.70	115.93	July, 2010	Active sampling	IRMS	Standard Calibration Method	0.3	http://dx.doi.org/10.1016/j.gexplo.2015.11.010
$\delta^{34}S$	S	Natural Soil	0.39‰	39.70	115.93	October, 2010	Active sampling	IRMS	Standard Calibration Method	0.3	http://dx.doi.org/10.1016/j.gexplo.2015.11.010
$\delta^{34}S$	S	Natural Soil	4.30‰	39.70	115.93	December, 2010	Active sampling	IRMS	Standard Calibration Method	0.3	http://dx.doi.org/10.1016/j.gexplo.2015.11.010
$\delta^{34}S$	S	Natural Soil	1.04‰	39.70	115.93	July, 2010	Active sampling	IRMS	Standard Calibration Method	0.3	http://dx.doi.org/10.1016/j.gexplo.2015.11.010
$\delta^{34}S$	S	Natural Soil	4.11‰	39.70	115.93	October, 2010	Active sampling	IRMS	Standard Calibration Method	0.3	http://dx.doi.org/10.1016/j.gexplo.2015.11.010
$\delta^{34}S$	S	Natural Soil	4.11‰	39.70	115.93	December, 2010	Active sampling	IRMS	Standard Calibration Method	0.3	http://dx.doi.org/10.1016/j.gexplo.2015.11.010
$\delta^{34}S$	S	Natural Soil	7.89‰	28.67	115.90	January, 2015	Active sampling	IRMS	Standard Calibration Method	0.2	https://doi.org/10.1016/S1002-0160(14)60084-9
$\delta^{34}S$	S	Natural Soil	7.11‰	24.47	118.08	April, 2015	Active sampling	IRMS	Standard Calibration Method	0.2	https://doi.org/10.1016/S1002-0160(14)60084-9

$\delta^{34}S$	S	Natural Soil	1.96‰	26.57	106.73	July, 2015	Active sampling	IRMS	Standard Calibration Method	0.2	https://doi.org/10.1016/S1002-0160(14)60084-9
$\delta^{34}S$	S	Natural Soil	7.04‰	39.93	119.58	October, 2015	Active sampling	IRMS	Standard Calibration Method	0.2	https://doi.org/10.1016/S1002-0160(14)60084-9
$\delta^{34}S$	S	Industrial Oil Combustion	24.20‰	41.87	123.92	April, 2000	Active sampling	VG Iso-gas MS	Standard Calibration Method	0.2	10.1246/nikkashi.2000.45 In Japanese with English abstract
$\delta^{34}S$	S	Industrial Oil Combustion	23.40‰	37.47	122.43	January, 2000	Active sampling	VG Iso-gas MS	Standard Calibration Method	0.2	10.1246/nikkashi.2000.45 In Japanese with English abstract
$\delta^{34}S$	S	Industrial Oil Combustion	7.20‰	46.59	125.10	July, 2000	Active sampling	VG Iso-gas MS	Standard Calibration Method	0.2	10.1246/nikkashi.2000.45 In Japanese with English abstract
$\delta^{34}S$	S	Industrial Oil Combustion	13.70‰	41.87	123.92	October, 2000	Active sampling	VG Iso-gas MS	Standard Calibration Method	0.2	10.1246/nikkashi.2000.45 In Japanese with English abstract
$\delta^{34}S$	S	Industrial Oil Combustion	20.60‰	41.87	123.92	April, 2000	Active sampling	VG Iso-gas MS	Standard Calibration Method	0.2	10.1246/nikkashi.2000.45 In Japanese with English abstract
$\delta^{34}S$	S	Industrial Oil Combustion	1.10‰	41.87	123.92	January, 2000	Active sampling	VG Iso-gas MS	Standard Calibration Method	0.2	10.1246/nikkashi.2000.45 In Japanese with English abstract
$\delta^{34}S$	S	Industrial Oil Combustion	4.40‰	4.40	113.99	July, 2000	Active sampling	VG Iso-gas MS	Standard Calibration Method	0.2	10.1246/nikkashi.2000.45 In Japanese with English abstract
$\delta^{34}S$	S	Industrial Oil Combustion	4.10‰	5.28	115.23	October, 2000	Active sampling	VG Iso-gas MS	Standard Calibration Method	0.2	10.1246/nikkashi.2000.45 In Japanese with English abstract
$\delta^{34}S$	S	Industrial Oil Combustion	3.70‰	4.61	114.32	April, 2000	Active sampling	VG Iso-gas MS	Standard Calibration Method	0.2	10.1246/nikkashi.2000.45 In Japanese with English abstract
$\delta^{34}S$	S	Industrial Oil Combustion	8.40‰	-32.14	133.68	January, 2000	Active sampling	VG Iso-gas MS	Standard Calibration Method	0.2	10.1246/nikkashi.2000.45 In Japanese with English abstract

$\delta^{34}S$	S	Industrial Oil Combustion	6.80‰	-31.65	116.13	July, 2000	Active sampling	VG Iso-gas MS	Standard Calibration Method	0.2	10.1246/nikkashi.2000.45 In Japanese with English abstract
$\delta^{34}S$	S	Industrial Oil Combustion	-0.70‰	32.43	53.69	October, 2000	Active sampling	VG Iso-gas MS	Standard Calibration Method	0.2	10.1246/nikkashi.2000.45 In Japanese with English abstract
$\delta^{34}S$	S	Industrial Oil Combustion	-2.60‰	24.55	53.91	April, 2000	Active sampling	VG Iso-gas MS	Standard Calibration Method	0.2	10.1246/nikkashi.2000.45 In Japanese with English abstract
$\delta^{34}S$	S	Industrial Oil Combustion	-10.30‰	25.28	55.30	January, 2000	Active sampling	VG Iso-gas MS	Standard Calibration Method	0.2	10.1246/nikkashi.2000.45 In Japanese with English abstract
$\delta^{34}S$	S	Industrial Oil Combustion	-4.40‰	24.09	52.86	July, 2000	Active sampling	VG Iso-gas MS	Standard Calibration Method	0.2	10.1246/nikkashi.2000.45 In Japanese with English abstract
$\delta^{34}S$	S	Industrial Oil Combustion	-4.30‰	23.89	45.08	October, 2000	Active sampling	VG Iso-gas MS	Standard Calibration Method	0.2	10.1246/nikkashi.2000.45 In Japanese with English abstract
$\delta^{34}S$	S	Industrial Oil Combustion	-0.70‰	28.43	48.49	April, 2000	Active sampling	VG Iso-gas MS	Standard Calibration Method	0.2	10.1246/nikkashi.2000.45 In Japanese with English abstract
$\delta^{34}S$	S	Industrial Oil Combustion	-8.80‰	21.47	55.98	January, 2000	Active sampling	VG Iso-gas MS	Standard Calibration Method	0.2	10.1246/nikkashi.2000.45 In Japanese with English abstract
$\delta^{34}S$	S	Industrial Oil Combustion	10.40‰	37.92	139.04	July, 2000	Active sampling	VG Iso-gas MS	Standard Calibration Method	0.2	10.1246/nikkashi.2000.45 In Japanese with English abstract
$\delta^{34}S$	S	Industrial Oil Combustion	10.80‰	37.90	139.02	October, 2000	Active sampling	VG Iso-gas MS	Standard Calibration Method	0.2	10.1246/nikkashi.2000.45 In Japanese with English abstract
$\delta^{34}S$	S	Industrial Oil Combustion	-6.80‰	37.90	139.02	April, 2000	Active sampling	VG Iso-gas MS	Standard Calibration Method	0.2	10.1246/nikkashi.2000.45 In Japanese with English abstract
$\delta^{34}S$	S	Industrial Oil Combustion	-6.00‰	37.90	139.02	January, 2000	Active sampling	VG Iso-gas MS	Standard Calibration Method	0.2	10.1246/nikkashi.2000.45

											In Japanese with English abstract
$\delta^{34}S$	S	Industrial Oil Combustion	0.10‰	37.90	139.02	July, 2000	Active sampling	VG Iso-gas MS	Standard Calibration Method	0.2	10.1246/nikkashi.2000.45 In Japanese with English abstract
$\delta^{34}S$	S	Industrial Oil Combustion	0.20‰	37.90	139.02	October, 2000	Active sampling	VG Iso-gas MS	Standard Calibration Method	0.2	10.1246/nikkashi.2000.45 In Japanese with English abstract
$\delta^{34}S$	S	Industrial Oil Combustion	3.90‰	37.90	139.02	April, 2000	Active sampling	VG Iso-gas MS	Standard Calibration Method	0.2	10.1246/nikkashi.2000.45 In Japanese with English abstract
$\delta^{34}S$	S	Industrial Oil Combustion	9.10‰	37.90	139.02	January, 2000	Active sampling	VG Iso-gas MS	Standard Calibration Method	0.2	10.1246/nikkashi.2000.45 In Japanese with English abstract
$\delta^{34}S$	S	Industrial Oil Combustion	-0.90‰	37.90	139.02	July, 2000	Active sampling	VG Iso-gas MS	Standard Calibration Method	0.2	10.1246/nikkashi.2000.45 In Japanese with English abstract
$\delta^{34}S$	S	Industrial Oil Combustion	-3.40‰	37.90	139.02	October, 2000	Active sampling	VG Iso-gas MS	Standard Calibration Method	0.2	10.1246/nikkashi.2000.45 In Japanese with English abstract
$\delta^{34}S$	S	Industrial Oil Combustion	-5.70‰	37.90	139.02	January, 2000	Active sampling	VG Iso-gas MS	Standard Calibration Method	0.2	10.1246/nikkashi.2000.45 In Japanese with English abstract
$\delta^{34}S$	S	Industrial Oil Combustion	3.10‰	37.90	139.02	October, 2000	Active sampling	VG Iso-gas MS	Standard Calibration Method	0.2	10.1246/nikkashi.2000.45 In Japanese with English abstract
$\delta^{34}S$	S	Industrial Oil Combustion	1.20‰	28.70	77.10	October, 2016	Active sampling	IRMS	Standard Calibration Method	0.2	https://doi.org/10.1016/j.apr.2018.12.015
$\delta^{34}S$	S	Industrial Oil Combustion	-4.40‰	37.95	139.02	April, 1997	Active sampling	VG Iso10 MS	Standard Calibration Method	0.2	https://doi.org/10.1016/S1352-2310(96)00278-6
$\delta^{34}S$	S	Industrial Oil Combustion	-0.30‰	37.95	139.02	July, 1997	Active sampling	VG Iso10 MS	Standard Calibration Method	0.2	https://doi.org/10.1016/S1352-2310(96)00278-6
$\delta^{34}S$	S	Industrial Oil Combustion	-7.30‰	37.95	139.02	January, 1997	Active sampling	VG Iso10 MS	Standard Calibration Method	0.2	https://doi.org/10.1016/S1352-2310(96)00278-6

$\delta^{34}S$	S	Industrial Oil Combustion	2.20‰	37.95	139.02	October, 1997	Active sampling	VG Iso10 MS	Standard Calibration Method	0.2	https://doi.org/10.1016/S1352-2310(96)00278-6
$\delta^{34}S$	S	Industrial Oil Combustion	-6.40‰	37.95	139.02	April, 1997	Active sampling	VG Iso10 MS	Standard Calibration Method	0.2	https://doi.org/10.1016/S1352-2310(96)00278-6
$\delta^{34}S$	S	Industrial Oil Combustion	7.20‰	37.95	139.02	July, 1997	Active sampling	VG Iso10 MS	Standard Calibration Method	0.2	https://doi.org/10.1016/S1352-2310(96)00278-6
$\delta^{34}S$	S	Industrial Oil Combustion	-3.20‰	37.95	139.02	January, 1997	Active sampling	VG Iso10 MS	Standard Calibration Method	0.2	https://doi.org/10.1016/S1352-2310(96)00278-6
$\delta^{34}S$	S	Industrial Oil Combustion	-4.50‰	37.95	139.02	October, 1997	Active sampling	VG Iso10 MS	Standard Calibration Method	0.2	https://doi.org/10.1016/S1352-2310(96)00278-6
$\delta^{34}S$	S	Industrial Oil Combustion	-8.20‰	37.95	139.02	April, 1997	Active sampling	VG Iso10 MS	Standard Calibration Method	0.2	https://doi.org/10.1016/S1352-2310(96)00278-6
$\delta^{34}S$	S	Industrial Oil Combustion	-2.60‰	37.95	139.02	July, 1997	Active sampling	VG Iso10 MS	Standard Calibration Method	0.2	https://doi.org/10.1016/S1352-2310(96)00278-6
$\delta^{34}S$	S	Industrial Oil Combustion	-4.60‰	37.95	139.02	January, 1997	Active sampling	VG Iso10 MS	Standard Calibration Method	0.2	https://doi.org/10.1016/S1352-2310(96)00278-6
$\delta^{34}S$	S	Industrial Oil Combustion	-4.90‰	37.95	139.02	October, 1997	Active sampling	VG Iso10 MS	Standard Calibration Method	0.2	https://doi.org/10.1016/S1352-2310(96)00278-6
$\delta^{34}S$	S	Industrial Oil Combustion	-2.30‰	37.95	139.02	July, 1997	Active sampling	VG Iso10 MS	Standard Calibration Method	0.2	https://doi.org/10.1016/S1352-2310(96)00278-6
$\delta^{34}S$	S	Industrial Oil Combustion	-3.50‰	37.95	139.02	January, 1997	Active sampling	VG Iso10 MS	Standard Calibration Method	0.2	https://doi.org/10.1016/S1352-2310(96)00278-6
$\delta^{34}S$	S	Industrial Oil Combustion	-6.40‰	37.95	139.02	October, 1997	Active sampling	VG Iso10 MS	Standard Calibration Method	0.2	https://doi.org/10.1016/S1352-2310(96)00278-6
$\delta^{34}S$	S	Industrial Oil Combustion	-1.60‰	51.45	0.23	October, 1992	Active sampling	IRMS	Standard Calibration Method	0.15	10.1029/98JD01664
$\delta^{34}S$	S	Industrial Oil Combustion	20.50‰	39.91	116.39	July, 2016	Active sampling	IRMS	Standard Calibration Method	0.2	10.1038/srep29958
$\delta^{34}S$	S	Industrial Oil Combustion	12.30‰	23.17	112.58	April, 2002	Active sampling	IRMS	Standard Calibration Method	0.2	10.3321/j.issn:1000-6923.2002.02.017 In Chinese with English abstract

$\delta^{34}S$	S	Industrial Oil Combustion	12.00‰	23.17	112.58	January, 2002	Active sampling	IRMS	Standard Calibration Method	0.2	10.3321/j.issn:1000-6923.2002.02.017 In Chinese with English abstract
$\delta^{34}S$	S	Industrial Oil Combustion	13.60‰	23.17	112.58	July, 2002	Active sampling	IRMS	Standard Calibration Method	0.2	10.3321/j.issn:1000-6923.2002.02.017 In Chinese with English abstract
$\delta^{34}S$	S	Industrial Oil Combustion	10.00‰	23.17	112.58	October, 2002	Active sampling	IRMS	Standard Calibration Method	0.2	10.3321/j.issn:1000-6923.2002.02.017 In Chinese with English abstract
$\delta^{34}S$	S	Industrial Oil Combustion	10.10‰	23.17	112.58	April, 2002	Active sampling	IRMS	Standard Calibration Method	0.2	10.3321/j.issn:1000-6923.2002.02.017 In Chinese with English abstract
$\delta^{34}S$	S	Industrial Oil Combustion	13.10‰	23.17	112.58	January, 2002	Active sampling	IRMS	Standard Calibration Method	0.2	10.3321/j.issn:1000-6923.2002.02.017 In Chinese with English abstract
$\delta^{34}S$	S	Industrial Oil Combustion	14.80‰	23.17	112.58	July, 2002	Active sampling	IRMS	Standard Calibration Method	0.2	10.3321/j.issn:1000-6923.2002.02.017 In Chinese with English abstract
$\delta^{34}S$	S	Industrial Oil Combustion	14.80‰	23.17	112.58	October, 2002	Active sampling	IRMS	Standard Calibration Method	0.2	10.3321/j.issn:1000-6923.2002.02.017 In Chinese with English abstract
$\delta^{34}S$	S	Industrial Oil Combustion	16.50‰	23.17	112.58	April, 2002	Active sampling	IRMS	Standard Calibration Method	0.2	10.3321/j.issn:1000-6923.2002.02.017 In Chinese with English abstract
$\delta^{34}S$	S	Industrial Oil Combustion	3.30‰	28.23	112.93	January, 2002	Active sampling	IRMS	Standard Calibration Method	0.2	10.3321/j.issn:1000-6923.2002.02.017 In Chinese with English abstract
$\delta^{34}S$	S	Industrial Oil Combustion	1.60‰	22.82	108.32	July, 2002	Active sampling	IRMS	Standard Calibration Method	0.2	10.3321/j.issn:1000-6923.2002.02.017 In Chinese with English abstract
$\delta^{34}S$	S	Industrial Oil Combustion	5.60‰	22.82	108.32	October, 2002	Active sampling	IRMS	Standard Calibration Method	0.2	10.3321/j.issn:1000-6923.2002.02.017 In Chinese with English abstract
$\delta^{34}S$	S	Industrial Oil Combustion	-1.30‰	-16.40	-71.53	October, 2005	Active sampling	IRMS	Standard Calibration Method	0.4	https://doi.org/10.1016/j.atmosenv.2021.118482

δ^{30}Si	Si	Biomass Burning	-0.91‰	38.68	115.26	January, 2013	Active sampling	MC-ICP-MS	Sample-Standard Bracketing	0.18	doi.org/10.1021/acs.est.7b06317
δ^{30}Si	Si	Biomass Burning	-0.41‰	38.68	115.26	April, 2013	Active sampling	MC-ICP-MS	Sample-Standard Bracketing	0.18	doi.org/10.1021/acs.est.7b06317
δ^{30}Si	Si	Biomass Burning	-0.45‰	38.68	115.26	July, 2013	Active sampling	MC-ICP-MS	Sample-Standard Bracketing	0.18	doi.org/10.1021/acs.est.7b06317
δ^{30}Si	Si	Biomass Burning	-0.53‰	38.68	115.26	October, 2013	Active sampling	MC-ICP-MS	Sample-Standard Bracketing	0.18	doi.org/10.1021/acs.est.7b06317
δ^{30}Si	Si	Biomass Burning	-0.07‰	38.68	115.26	January, 2013	Active sampling	MC-ICP-MS	Sample-Standard Bracketing	0.18	doi.org/10.1021/acs.est.7b06317
δ^{30}Si	Si	Biomass Burning	-0.23‰	38.68	115.26	April, 2013	Active sampling	MC-ICP-MS	Sample-Standard Bracketing	0.18	doi.org/10.1021/acs.est.7b06317
δ^{30}Si	Si	Biomass Burning	0.13‰	38.68	115.26	July, 2013	Active sampling	MC-ICP-MS	Sample-Standard Bracketing	0.18	doi.org/10.1021/acs.est.7b06317
δ^{30}Si	Si	Biomass Burning	-0.86‰	38.68	115.26	October, 2013	Active sampling	MC-ICP-MS	Sample-Standard Bracketing	0.18	doi.org/10.1021/acs.est.7b06317
δ^{30}Si	Si	Biomass Burning	-0.51‰	38.68	115.26	January, 2013	Active sampling	MC-ICP-MS	Sample-Standard Bracketing	0.18	doi.org/10.1021/acs.est.7b06317
δ^{30}Si	Si	Biomass Burning	-0.75‰	38.68	115.26	April, 2013	Active sampling	MC-ICP-MS	Sample-Standard Bracketing	0.18	doi.org/10.1021/acs.est.7b06317
δ^{30}Si	Si	Coal Combustion	-1.21‰	40.00	116.33	July, 2013	Active sampling	MC-ICP-MS	Sample-Standard Bracketing	0.18	doi.org/10.1021/acs.est.7b06317
δ^{30}Si	Si	Coal Combustion	-1.41‰	40.00	116.33	October, 2013	Active sampling	MC-ICP-MS	Sample-Standard Bracketing	0.18	doi.org/10.1021/acs.est.7b06317
δ^{30}Si	Si	Coal Combustion	-3.35‰	40.00	116.33	January, 2013	Active sampling	MC-ICP-MS	Sample-Standard Bracketing	0.18	doi.org/10.1021/acs.est.7b06317
δ^{30}Si	Si	Coal Combustion	-2.46‰	40.00	116.33	April, 2013	Active sampling	MC-ICP-MS	Sample-Standard Bracketing	0.18	doi.org/10.1021/acs.est.7b06317
δ^{30}Si	Si	Coal Combustion	-2.91‰	40.00	116.33	July, 2013	Active sampling	MC-ICP-MS	Sample-Standard Bracketing	0.18	doi.org/10.1021/acs.est.7b06317

δ^{30}Si	Si	Coal Combustion	-2.62‰	40.00	116.33	October, 2013	Active sampling	MC-ICP-MS	Sample-Standard Bracketing	0.18	doi.org/10.1021/acs.est.7b06317
δ^{30}Si	Si	Coal Combustion	-2.38‰	40.00	116.33	January, 2013	Active sampling	MC-ICP-MS	Sample-Standard Bracketing	0.18	doi.org/10.1021/acs.est.7b06317
δ^{30}Si	Si	Coal Combustion	-3.28‰	40.00	116.33	April, 2013	Active sampling	MC-ICP-MS	Sample-Standard Bracketing	0.18	doi.org/10.1021/acs.est.7b06317
δ^{30}Si	Si	Coal Combustion	-2.82‰	40.00	116.33	July, 2013	Active sampling	MC-ICP-MS	Sample-Standard Bracketing	0.18	doi.org/10.1021/acs.est.7b06317
δ^{30}Si	Si	Coal Combustion	-1.99‰	40.00	116.33	October, 2013	Active sampling	MC-ICP-MS	Sample-Standard Bracketing	0.18	doi.org/10.1021/acs.est.7b06317
δ^{30}Si	Si	Coal Combustion	-2.32‰	40.00	116.33	January, 2013	Active sampling	MC-ICP-MS	Sample-Standard Bracketing	0.18	doi.org/10.1021/acs.est.7b06317
δ^{30}Si	Si	Ore-related Emissions	-1.46‰	39.70	118.46	April, 2013	Active sampling	MC-ICP-MS	Sample-Standard Bracketing	0.18	doi.org/10.1021/acs.est.7b06317
δ^{30}Si	Si	Ore-related Emissions	-0.87‰	39.70	118.46	July, 2013	Active sampling	MC-ICP-MS	Sample-Standard Bracketing	0.18	doi.org/10.1021/acs.est.7b06317
δ^{30}Si	Si	Ore-related Emissions	-1.80‰	39.70	118.46	October, 2013	Active sampling	MC-ICP-MS	Sample-Standard Bracketing	0.18	doi.org/10.1021/acs.est.7b06317
δ^{30}Si	Si	Ore-related Emissions	-0.92‰	39.70	118.46	January, 2013	Active sampling	MC-ICP-MS	Sample-Standard Bracketing	0.18	doi.org/10.1021/acs.est.7b06317
δ^{30}Si	Si	Ore-related Emissions	-1.51‰	39.70	118.46	April, 2013	Active sampling	MC-ICP-MS	Sample-Standard Bracketing	0.18	doi.org/10.1021/acs.est.7b06317
δ^{30}Si	Si	Ore-related Emissions	-0.87‰	39.70	118.46	July, 2013	Active sampling	MC-ICP-MS	Sample-Standard Bracketing	0.18	doi.org/10.1021/acs.est.7b06317
δ^{30}Si	Si	Ore-related Emissions	-1.75‰	39.70	118.46	October, 2013	Active sampling	MC-ICP-MS	Sample-Standard Bracketing	0.18	doi.org/10.1021/acs.est.7b06317
δ^{30}Si	Si	Ore-related Emissions	-1.84‰	39.70	118.46	January, 2013	Active sampling	MC-ICP-MS	Sample-Standard Bracketing	0.18	doi.org/10.1021/acs.est.7b06317
δ^{30}Si	Si	Natural Soil	0.18‰	38.68	115.26	April, 2013	Active sampling	MC-ICP-MS	Sample-Standard Bracketing	0.18	doi.org/10.1021/acs.est.7b06317

$\delta^{30}Si$	Si	Natural Soil	-0.34‰	38.68	115.26	July, 2013	Active sampling	MC-ICP-MS	Sample-Standard Bracketing	0.18	doi.org/10.1021/acs.est.7b06317
$\delta^{30}Si$	Si	Natural Soil	-0.38‰	38.68	115.26	October, 2013	Active sampling	MC-ICP-MS	Sample-Standard Bracketing	0.18	doi.org/10.1021/acs.est.7b06317
$\delta^{30}Si$	Si	Natural Soil	-0.50‰	38.68	115.26	January, 2013	Active sampling	MC-ICP-MS	Sample-Standard Bracketing	0.18	doi.org/10.1021/acs.est.7b06317
$\delta^{30}Si$	Si	Natural Soil	-0.53‰	38.68	115.26	April, 2013	Active sampling	MC-ICP-MS	Sample-Standard Bracketing	0.18	doi.org/10.1021/acs.est.7b06317
$\delta^{30}Si$	Si	Natural Soil	-0.61‰	38.68	115.26	July, 2013	Active sampling	MC-ICP-MS	Sample-Standard Bracketing	0.18	doi.org/10.1021/acs.est.7b06317
$\delta^{30}Si$	Si	Natural Soil	-0.23‰	38.68	115.26	October, 2013	Active sampling	MC-ICP-MS	Sample-Standard Bracketing	0.18	doi.org/10.1021/acs.est.7b06317
$\delta^{30}Si$	Si	Natural Soil	-0.56‰	38.68	115.26	January, 2013	Active sampling	MC-ICP-MS	Sample-Standard Bracketing	0.18	doi.org/10.1021/acs.est.7b06317
$\delta^{30}Si$	Si	Natural Soil	-0.29‰	38.68	115.26	April, 2013	Active sampling	MC-ICP-MS	Sample-Standard Bracketing	0.18	doi.org/10.1021/acs.est.7b06317
$\delta^{30}Si$	Si	Natural Soil	-0.19‰	38.68	115.26	July, 2013	Active sampling	MC-ICP-MS	Sample-Standard Bracketing	0.18	doi.org/10.1021/acs.est.7b06317
$\delta^{30}Si$	Si	Natural Soil	0.16‰	38.68	115.26	October, 2013	Active sampling	MC-ICP-MS	Sample-Standard Bracketing	0.18	doi.org/10.1021/acs.est.7b06317
$\delta^{30}Si$	Si	Natural Soil	-0.73‰	38.68	115.26	January, 2013	Active sampling	MC-ICP-MS	Sample-Standard Bracketing	0.18	doi.org/10.1021/acs.est.7b06317
$\delta^{30}Si$	Si	Natural Soil	-0.71‰	38.68	115.26	April, 2013	Active sampling	MC-ICP-MS	Sample-Standard Bracketing	0.18	doi.org/10.1021/acs.est.7b06317
$\delta^{30}Si$	Si	Natural Soil	-0.15‰	38.68	115.26	July, 2013	Active sampling	MC-ICP-MS	Sample-Standard Bracketing	0.18	doi.org/10.1021/acs.est.7b06317
$\delta^{30}Si$	Si	Natural Soil	-0.18‰	38.68	115.26	October, 2013	Active sampling	MC-ICP-MS	Sample-Standard Bracketing	0.18	doi.org/10.1021/acs.est.7b06317
$\delta^{30}Si$	Si	Natural Soil	-0.36‰	38.68	115.26	January, 2013	Active sampling	MC-ICP-MS	Sample-Standard Bracketing	0.18	doi.org/10.1021/acs.est.7b06317

$\delta^{30}Si$	Si	Natural Soil	-0.39‰	38.68	115.26	April, 2013	Active sampling	MC-ICP-MS	Sample-Standard Bracketing	0.18	doi.org/10.1021/acs.est.7b06317
$\delta^{30}Si$	Si	Natural Soil	-0.23‰	38.68	115.26	July, 2013	Active sampling	MC-ICP-MS	Sample-Standard Bracketing	0.18	doi.org/10.1021/acs.est.7b06317
$\delta^{30}Si$	Si	Natural Soil	-0.17‰	38.68	115.26	October, 2013	Active sampling	MC-ICP-MS	Sample-Standard Bracketing	0.18	doi.org/10.1021/acs.est.7b06317
$\delta^{30}Si$	Si	Natural Soil	-0.44‰	38.68	115.26	January, 2013	Active sampling	MC-ICP-MS	Sample-Standard Bracketing	0.18	doi.org/10.1021/acs.est.7b06317
$\delta^{30}Si$	Si	Natural Soil	-0.48‰	38.68	115.26	April, 2013	Active sampling	MC-ICP-MS	Sample-Standard Bracketing	0.18	doi.org/10.1021/acs.est.7b06317
$\delta^{30}Si$	Si	Natural Soil	-0.19‰	38.68	115.26	July, 2013	Active sampling	MC-ICP-MS	Sample-Standard Bracketing	0.18	doi.org/10.1021/acs.est.7b06317
$\delta^{30}Si$	Si	Natural Soil	-0.29‰	38.68	115.26	October, 2013	Active sampling	MC-ICP-MS	Sample-Standard Bracketing	0.18	doi.org/10.1021/acs.est.7b06317
$\delta^{30}Si$	Si	Natural Soil	-0.03‰	38.68	115.26	January, 2013	Active sampling	MC-ICP-MS	Sample-Standard Bracketing	0.18	doi.org/10.1021/acs.est.7b06317
$\delta^{30}Si$	Si	Natural Soil	-0.19‰	38.68	115.26	April, 2013	Active sampling	MC-ICP-MS	Sample-Standard Bracketing	0.18	doi.org/10.1021/acs.est.7b06317
$\delta^{30}Si$	Si	Natural Soil	0.15‰	38.68	115.26	July, 2013	Active sampling	MC-ICP-MS	Sample-Standard Bracketing	0.18	doi.org/10.1021/acs.est.7b06317
$\delta^{30}Si$	Si	Natural Soil	0.08‰	38.68	115.26	October, 2013	Active sampling	MC-ICP-MS	Sample-Standard Bracketing	0.18	doi.org/10.1021/acs.est.7b06317
$\delta^{30}Si$	Si	Natural Soil	-0.20‰	38.68	115.26	January, 2013	Active sampling	MC-ICP-MS	Sample-Standard Bracketing	0.18	doi.org/10.1021/acs.est.7b06317
$\delta^{30}Si$	Si	Natural Soil	-0.41‰	38.68	115.26	April, 2013	Active sampling	MC-ICP-MS	Sample-Standard Bracketing	0.18	doi.org/10.1021/acs.est.7b06317
$\delta^{30}Si$	Si	Natural Soil	-0.38‰	38.68	115.26	July, 2013	Active sampling	MC-ICP-MS	Sample-Standard Bracketing	0.18	doi.org/10.1021/acs.est.7b06317
$\delta^{30}Si$	Si	Natural Soil	0.18‰	38.68	115.26	October, 2013	Active sampling	MC-ICP-MS	Sample-Standard Bracketing	0.18	doi.org/10.1021/acs.est.7b06317

δ^{30}Si	Si	Natural Soil	-0.37‰	38.68	115.26	January, 2013	Active sampling	MC-ICP-MS	Sample-Standard Bracketing	0.18	doi.org/10.1021/acs.est.7b06317
δ^{30}Si	Si	Natural Soil	-0.52‰	38.68	115.26	April, 2013	Active sampling	MC-ICP-MS	Sample-Standard Bracketing	0.18	doi.org/10.1021/acs.est.7b06317
δ^{30}Si	Si	Natural Soil	-0.03‰	38.68	115.26	July, 2013	Active sampling	MC-ICP-MS	Sample-Standard Bracketing	0.18	doi.org/10.1021/acs.est.7b06317
δ^{30}Si	Si	Natural Soil	-0.36‰	38.68	115.26	October, 2013	Active sampling	MC-ICP-MS	Sample-Standard Bracketing	0.18	doi.org/10.1021/acs.est.7b06317
δ^{30}Si	Si	Natural Soil	-0.34‰	38.68	115.26	January, 2013	Active sampling	MC-ICP-MS	Sample-Standard Bracketing	0.18	doi.org/10.1021/acs.est.7b06317
δ^{30}Si	Si	Natural Soil	0.11‰	38.68	115.26	April, 2013	Active sampling	MC-ICP-MS	Sample-Standard Bracketing	0.18	doi.org/10.1021/acs.est.7b06317
δ^{30}Si	Si	Natural Soil	-0.32‰	38.68	115.26	July, 2013	Active sampling	MC-ICP-MS	Sample-Standard Bracketing	0.18	doi.org/10.1021/acs.est.7b06317
δ^{30}Si	Si	Natural Soil	-0.55‰	38.68	115.26	October, 2013	Active sampling	MC-ICP-MS	Sample-Standard Bracketing	0.18	doi.org/10.1021/acs.est.7b06317
δ^{30}Si	Si	Vehicle Exhausts	0.75‰	40.32	116.00	April, 2013	Active sampling	MC-ICP-MS	Sample-Standard Bracketing	0.18	doi.org/10.1021/acs.est.7b06317
δ^{30}Si	Si	Vehicle Exhausts	1.20‰	40.32	116.00	July, 2013	Active sampling	MC-ICP-MS	Sample-Standard Bracketing	0.18	doi.org/10.1021/acs.est.7b06317
δ^{30}Si	Si	Vehicle Exhausts	0.94‰	40.32	116.00	October, 2013	Active sampling	MC-ICP-MS	Sample-Standard Bracketing	0.18	doi.org/10.1021/acs.est.7b06317
δ^{87}Sr	Sr	Coal Combustion	14.993‰	39.92	116.13	July, 2010	Active sampling	TIMS	Standard Calibration Method	0.22	10.1016/j.atmosenv.2010.06.036
δ^{87}Sr	Sr	Coal Combustion	15.022‰	39.92	116.13	July, 2010	Active sampling	TIMS	Standard Calibration Method	0.22	10.1016/j.atmosenv.2010.06.036
δ^{87}Sr	Sr	Coal Combustion	14.364‰	39.88	116.32	July, 2010	Active sampling	TIMS	Standard Calibration Method	0.22	10.1016/j.atmosenv.2010.06.036
δ^{87}Sr	Sr	Coal Combustion	14.278‰	35.07	118.33	July, 2010	Active sampling	TIMS	Standard Calibration Method	0.22	10.1016/j.atmosenv.2010.06.036

δ^{87}Sr	Sr	Coal Combustion	67.383‰	21.02	107.32	April, 2015	Active sampling	-	Standard Calibration Method	0.3	dx.doi.org/10.1016/j.crte.2015.02.007
δ^{87}Sr	Sr	Coal Combustion	16.324‰	24.47	118.08	April, 2018	Active sampling	TIMS	Standard Calibration Method	0.3	doi.org/10.1016/j.atmosenv.2018.10.056
δ^{87}Sr	Sr	Coal Combustion	16.066‰	24.47	118.08	April, 2018	Active sampling	TIMS	Standard Calibration Method	0.3	doi.org/10.1016/j.atmosenv.2018.10.056
δ^{87}Sr	Sr	Coal Combustion	15.479‰	24.47	118.08	April, 2018	Active sampling	TIMS	Standard Calibration Method	0.3	doi.org/10.1016/j.atmosenv.2018.10.056
δ^{87}Sr	Sr	Coal Combustion	14.464‰	24.47	118.08	April, 2018	Active sampling	TIMS	Standard Calibration Method	0.3	doi.org/10.1016/j.atmosenv.2018.10.056
δ^{87}Sr	Sr	Vehicle Exhausts	40.816‰	21.07	107.32	April, 2015	Active sampling	-	Standard Calibration Method	0.3	dx.doi.org/10.1016/j.crte.2015.02.007
δ^{87}Sr	Sr	Vehicle Exhausts	47.554‰	21.07	107.32	April, 2015	Active sampling	-	Standard Calibration Method	0.3	dx.doi.org/10.1016/j.crte.2015.02.007
δ^{87}Sr	Sr	Vehicle Exhausts	21.402‰	21.07	107.32	April, 2015	Active sampling	-	Standard Calibration Method	0.3	dx.doi.org/10.1016/j.crte.2015.02.007
δ^{87}Sr	Sr	Vehicle Exhausts	32.461‰	21.07	107.32	April, 2015	Active sampling	-	Standard Calibration Method	0.3	dx.doi.org/10.1016/j.crte.2015.02.007
δ^{87}Sr	Sr	Vehicle Exhausts	31.002‰	21.07	107.32	April, 2015	Active sampling	-	Standard Calibration Method	0.3	dx.doi.org/10.1016/j.crte.2015.02.007
δ^{87}Sr	Sr	Vehicle Exhausts	20.058‰	21.02	107.35	April, 2015	Active sampling	-	Standard Calibration Method	0.3	dx.doi.org/10.1016/j.crte.2015.02.007
δ^{87}Sr	Sr	Vehicle Exhausts	13.505‰	24.47	118.08	April, 2018	Active sampling	TIMS	Standard Calibration Method	0.3	doi.org/10.1016/j.atmosenv.2018.10.056
δ^{87}Sr	Sr	Vehicle Exhausts	16.309‰	24.47	118.08	April, 2018	Active sampling	TIMS	Standard Calibration Method	0.3	doi.org/10.1016/j.atmosenv.2018.10.056
δ^{87}Sr	Sr	Vehicle Exhausts	15.708‰	48.57	7.75	2011	Active sampling	TIMS	Standard Calibration Method	0.13	10.1016/j.apgeochem.2008.02.004
δ^{87}Sr	Sr	Vehicle Exhausts	14.836‰	48.57	7.75	2018	Active sampling	TIMS	Standard Calibration Method	0.13	10.1016/j.apgeochem.2008.02.004

δ^{87}Sr	Sr	Vehicle Exhausts	14.049‰	48.57	7.75	July, 2008	Active sampling	TIMS	Standard Calibration Method	0.13	10.1016/j.apgeochem.2008.02.004
δ^{87}Sr	Sr	Vehicle Exhausts	13.906‰	48.57	7.75	July, 2008	Active sampling	TIMS	Standard Calibration Method	0.13	https://doi.org/10.1021/es071704c
δ^{87}Sr	Sr	Vehicle Exhausts	14.049‰	48.57	7.75	July, 2008	Active sampling	TIMS	Standard Calibration Method	0.13	https://doi.org/10.1021/es071704c
δ^{87}Sr	Sr	Vehicle Exhausts	22.461‰	29.73	-95.21	3 January-2 February, 2013	Active sampling	MC-ICP-MS	Standard Calibration Method	0.08	https://doi.org/10.1016/j.talanta.2022.123236
δ^{87}Sr	Sr	Vehicle Exhausts	23.305‰	29.73	-95.21	3 January-2 February, 2013	Active sampling	MC-ICP-MS	Standard Calibration Method	0.08	https://doi.org/10.1016/j.talanta.2022.123236
δ^{87}Sr	Sr	Natural Soil	14.936‰	26.57	106.71	July, 2021	Active sampling	TIMS	Standard Calibration Method	0.15	https://doi.org/10.1007/s12665-021-10075-0
δ^{87}Sr	Sr	Natural Soil	14.893‰	26.57	106.71	July, 2021	Active sampling	TIMS	Standard Calibration Method	0.15	https://doi.org/10.1007/s12665-021-10075-0
δ^{87}Sr	Sr	Natural Soil	16.138‰	26.57	106.71	July, 2021	Active sampling	TIMS	Standard Calibration Method	0.15	https://doi.org/10.1007/s12665-021-10075-0
δ^{87}Sr	Sr	Natural Soil	22.218‰	39.91	116.39	July, 2010	Active sampling	TIMS	Standard Calibration Method	0.22	10.1016/j.atmosenv.2010.06.036
δ^{87}Sr	Sr	Natural Soil	22.618‰	39.91	116.39	July, 2010	Active sampling	TIMS	Standard Calibration Method	0.22	10.1016/j.atmosenv.2010.06.036
δ^{87}Sr	Sr	Natural Soil	18.298‰	43.63	111.98	July, 2010	Active sampling	TIMS	Standard Calibration Method	0.22	10.1016/j.atmosenv.2010.06.036
δ^{87}Sr	Sr	Natural Soil	20.472‰	41.52	111.70	July, 2010	Active sampling	TIMS	Standard Calibration Method	0.22	10.1016/j.atmosenv.2010.06.036
δ^{87}Sr	Sr	Natural Soil	20.973‰	40.73	107.38	July, 2010	Active sampling	TIMS	Standard Calibration Method	0.22	10.1016/j.atmosenv.2010.06.036
δ^{87}Sr	Sr	Natural Soil	34.722‰	48.65	7.75	July, 2012	Active sampling	MC-ICP-MS	Standard Calibration Method	0.3	http://dx.doi.org/10.1016/j.atmosenv.2012.08.044
δ^{87}Sr	Sr	Natural Soil	24.006‰	42.67	74.52	October, 2015	Active sampling	MC-ICP-MS	Standard Calibration Method	0.1	doi.org/10.1016/j.atmosenv.2015.09.017

$\delta^{87}Sr$	Sr	Natural Soil	24.078‰	42.45	78.52	October, 2015	Active sampling	MC-ICP-MS	Standard Calibration Method	0.1	doi.org/10.1016/j.atmosenv.2015.09.017
$\delta^{87}Sr$	Sr	Natural Soil	12.561‰	21.07	107.32	April, 2015	Active sampling	-	Standard Calibration Method	0.3	dx.doi.org/10.1016/j.crte.2015.02.007
$\delta^{87}Sr$	Sr	Natural Soil	52.976‰	21.07	107.32	April, 2015	Active sampling	-	Standard Calibration Method	0.3	dx.doi.org/10.1016/j.crte.2015.02.007
$\delta^{87}Sr$	Sr	Natural Soil	68.585‰	21.07	107.32	April, 2015	Active sampling	-	Standard Calibration Method	0.3	dx.doi.org/10.1016/j.crte.2015.02.007
$\delta^{87}Sr$	Sr	Natural Soil	68.971‰	21.07	107.32	April, 2015	Active sampling	-	Standard Calibration Method	0.3	dx.doi.org/10.1016/j.crte.2015.02.007
$\delta^{87}Sr$	Sr	Natural Soil	19.857‰	28.67	115.85	2015	Active sampling	TIMS	Standard Calibration Method	0.15	doi.org/10.1016/j.atmosenv.2019.117069
$\delta^{87}Sr$	Sr	Natural Soil	16.610‰	28.67	115.85	2015	Active sampling	TIMS	Standard Calibration Method	0.15	doi.org/10.1016/j.atmosenv.2019.117069
$\delta^{87}Sr$	Sr	Natural Soil	20.372‰	28.67	115.85	2015	Active sampling	TIMS	Standard Calibration Method	0.15	doi.org/10.1016/j.atmosenv.2019.117069
$\delta^{87}Sr$	Sr	Natural Soil	21.846‰	28.67	115.85	2015	Active sampling	TIMS	Standard Calibration Method	0.15	doi.org/10.1016/j.atmosenv.2019.117069
$\delta^{87}Sr$	Sr	Natural Soil	17.854‰	28.67	115.85	2015	Active sampling	TIMS	Standard Calibration Method	0.15	doi.org/10.1016/j.atmosenv.2019.117069
$\delta^{87}Sr$	Sr	Natural Soil	19.271‰	28.67	115.85	2015	Active sampling	TIMS	Standard Calibration Method	0.15	doi.org/10.1016/j.atmosenv.2019.117069
$\delta^{87}Sr$	Sr	Natural Soil	18.698‰	28.67	115.85	2015	Active sampling	TIMS	Standard Calibration Method	0.15	doi.org/10.1016/j.atmosenv.2019.117069
$\delta^{87}Sr$	Sr	Natural Soil	17.554‰	28.67	115.85	2015	Active sampling	TIMS	Standard Calibration Method	0.15	doi.org/10.1016/j.atmosenv.2019.117069
$\delta^{87}Sr$	Sr	Natural Soil	19.857‰	28.67	115.85	2015	Active sampling	TIMS	Standard Calibration Method	0.15	doi.org/10.1016/j.atmosenv.2019.117069
$\delta^{87}Sr$	Sr	Natural Soil	19.042‰	28.67	115.85	2015	Active sampling	TIMS	Standard Calibration Method	0.15	doi.org/10.1016/j.atmosenv.2019.117069

$\delta^{87}Sr$	Sr	Natural Soil	23.219‰	25.90	109.65	July, 2015	Active sampling	TIMS	Standard Calibration Method	0.1	doi.org/10.1016/j.geoderma.2015.08.007
$\delta^{87}Sr$	Sr	Natural Soil	24.621‰	25.90	109.65	July, 2015	Active sampling	TIMS	Standard Calibration Method	0.1	doi.org/10.1016/j.geoderma.2015.08.007
$\delta^{87}Sr$	Sr	Natural Soil	25.422‰	25.90	109.65	July, 2015	Active sampling	TIMS	Standard Calibration Method	0.1	doi.org/10.1016/j.geoderma.2015.08.007
$\delta^{87}Sr$	Sr	Natural Soil	26.152‰	25.90	109.65	July, 2015	Active sampling	TIMS	Standard Calibration Method	0.1	doi.org/10.1016/j.geoderma.2015.08.007
$\delta^{87}Sr$	Sr	Natural Soil	28.427‰	25.90	109.65	July, 2015	Active sampling	TIMS	Standard Calibration Method	0.1	doi.org/10.1016/j.geoderma.2015.08.007
$\delta^{87}Sr$	Sr	Natural Soil	25.966‰	25.90	109.65	July, 2015	Active sampling	TIMS	Standard Calibration Method	0.1	doi.org/10.1016/j.geoderma.2015.08.007
$\delta^{87}Sr$	Sr	Natural Soil	22.032‰	25.90	109.65	July, 2015	Active sampling	TIMS	Standard Calibration Method	0.1	doi.org/10.1016/j.geoderma.2015.08.007
$\delta^{87}Sr$	Sr	Natural Soil	16.023‰	25.90	109.65	July, 2015	Active sampling	TIMS	Standard Calibration Method	0.1	doi.org/10.1016/j.geoderma.2015.08.007
$\delta^{87}Sr$	Sr	Natural Soil	6.910‰	25.90	109.65	July, 2015	Active sampling	TIMS	Standard Calibration Method	0.1	doi.org/10.1016/j.geoderma.2015.08.007
$\delta^{87}Sr$	Sr	Natural Soil	24.607‰	19.73	110.00	July, 2015	Active sampling	TIMS	Standard Calibration Method	0.1	doi.org/10.1016/j.geoderma.2015.08.007
$\delta^{87}Sr$	Sr	Natural Soil	25.966‰	19.73	110.00	July, 2015	Active sampling	TIMS	Standard Calibration Method	0.1	doi.org/10.1016/j.geoderma.2015.08.007
$\delta^{87}Sr$	Sr	Natural Soil	35.666‰	19.73	110.00	July, 2015	Active sampling	TIMS	Standard Calibration Method	0.1	doi.org/10.1016/j.geoderma.2015.08.007
$\delta^{87}Sr$	Sr	Natural Soil	25.966‰	19.73	110.00	July, 2015	Active sampling	TIMS	Standard Calibration Method	0.1	doi.org/10.1016/j.geoderma.2015.08.007
$\delta^{87}Sr$	Sr	Natural Soil	25.966‰	19.73	110.00	July, 2015	Active sampling	TIMS	Standard Calibration Method	0.1	doi.org/10.1016/j.geoderma.2015.08.007
$\delta^{87}Sr$	Sr	Natural Soil	25.937‰	19.73	110.00	July, 2015	Active sampling	TIMS	Standard Calibration Method	0.1	doi.org/10.1016/j.geoderma.2015.08.007

$\delta^{87}Sr$	Sr	Natural Soil	25.852‰	19.73	110.00	July, 2015	Active sampling	TIMS	Standard Calibration Method	0.1	doi.org/10.1016/j.geoderma.2015.08.007
$\delta^{87}Sr$	Sr	Natural Soil	25.780‰	19.73	110.00	July, 2015	Active sampling	TIMS	Standard Calibration Method	0.1	doi.org/10.1016/j.geoderma.2015.08.007
$\delta^{87}Sr$	Sr	Natural Soil	26.510‰	19.73	110.00	July, 2015	Active sampling	TIMS	Standard Calibration Method	0.1	doi.org/10.1016/j.geoderma.2015.08.007
$\delta^{87}Sr$	Sr	Natural Soil	26.295‰	19.73	110.00	July, 2015	Active sampling	TIMS	Standard Calibration Method	0.1	doi.org/10.1016/j.geoderma.2015.08.007
$\delta^{87}Sr$	Sr	Natural Soil	15.894‰	19.73	110.00	July, 2015	Active sampling	TIMS	Standard Calibration Method	0.1	doi.org/10.1016/j.geoderma.2015.08.007
$\delta^{87}Sr$	Sr	Natural Soil	9.843‰	19.73	110.00	July, 2015	Active sampling	TIMS	Standard Calibration Method	0.1	doi.org/10.1016/j.geoderma.2015.08.007
$\delta^{87}Sr$	Sr	Natural Soil	6.953‰	19.73	110.00	July, 2015	Active sampling	TIMS	Standard Calibration Method	0.1	doi.org/10.1016/j.geoderma.2015.08.007
$\delta^{87}Sr$	Sr	Natural Soil	21.603‰	19.95	109.63	July, 2015	Active sampling	TIMS	Standard Calibration Method	0.1	doi.org/10.1016/j.geoderma.2015.08.007
$\delta^{87}Sr$	Sr	Natural Soil	24.135‰	19.95	109.63	July, 2015	Active sampling	TIMS	Standard Calibration Method	0.1	doi.org/10.1016/j.geoderma.2015.08.007
$\delta^{87}Sr$	Sr	Natural Soil	25.108‰	19.95	109.63	July, 2015	Active sampling	TIMS	Standard Calibration Method	0.1	doi.org/10.1016/j.geoderma.2015.08.007
$\delta^{87}Sr$	Sr	Natural Soil	25.666‰	19.95	109.63	July, 2015	Active sampling	TIMS	Standard Calibration Method	0.1	doi.org/10.1016/j.geoderma.2015.08.007
$\delta^{87}Sr$	Sr	Natural Soil	24.836‰	19.95	109.63	July, 2015	Active sampling	TIMS	Standard Calibration Method	0.1	doi.org/10.1016/j.geoderma.2015.08.007
$\delta^{87}Sr$	Sr	Natural Soil	25.437‰	19.95	109.63	July, 2015	Active sampling	TIMS	Standard Calibration Method	0.1	doi.org/10.1016/j.geoderma.2015.08.007
$\delta^{87}Sr$	Sr	Natural Soil	26.324‰	19.95	109.63	July, 2015	Active sampling	TIMS	Standard Calibration Method	0.1	doi.org/10.1016/j.geoderma.2015.08.007
$\delta^{87}Sr$	Sr	Natural Soil	25.937‰	19.95	109.63	July, 2015	Active sampling	TIMS	Standard Calibration Method	0.1	doi.org/10.1016/j.geoderma.2015.08.007

$\delta^{87}Sr$	Sr	Natural Soil	22.661‰	19.95	109.63	July, 2015	Active sampling	TIMS	Standard Calibration Method	0.1	doi.org/10.1016/j.geoderma.2015.08.007
$\delta^{87}Sr$	Sr	Natural Soil	7.024‰	19.95	109.63	July, 2015	Active sampling	TIMS	Standard Calibration Method	0.1	doi.org/10.1016/j.geoderma.2015.08.007
$\delta^{87}Sr$	Sr	Natural Soil	9.714‰	19.93	110.20	July, 2015	Active sampling	TIMS	Standard Calibration Method	0.1	doi.org/10.1016/j.geoderma.2015.08.007
$\delta^{87}Sr$	Sr	Natural Soil	9.943‰	19.93	110.20	July, 2015	Active sampling	TIMS	Standard Calibration Method	0.1	doi.org/10.1016/j.geoderma.2015.08.007
$\delta^{87}Sr$	Sr	Natural Soil	10.243‰	19.93	110.20	July, 2015	Active sampling	TIMS	Standard Calibration Method	0.1	doi.org/10.1016/j.geoderma.2015.08.007
$\delta^{87}Sr$	Sr	Natural Soil	10.358‰	19.93	110.20	July, 2015	Active sampling	TIMS	Standard Calibration Method	0.1	doi.org/10.1016/j.geoderma.2015.08.007
$\delta^{87}Sr$	Sr	Natural Soil	10.286‰	19.93	110.20	July, 2015	Active sampling	TIMS	Standard Calibration Method	0.1	doi.org/10.1016/j.geoderma.2015.08.007
$\delta^{87}Sr$	Sr	Natural Soil	10.701‰	19.93	110.20	July, 2015	Active sampling	TIMS	Standard Calibration Method	0.1	doi.org/10.1016/j.geoderma.2015.08.007
$\delta^{87}Sr$	Sr	Natural Soil	9.442‰	19.93	110.20	July, 2015	Active sampling	TIMS	Standard Calibration Method	0.1	doi.org/10.1016/j.geoderma.2015.08.007
$\delta^{87}Sr$	Sr	Natural Soil	6.939‰	19.93	110.20	July, 2015	Active sampling	TIMS	Standard Calibration Method	0.1	doi.org/10.1016/j.geoderma.2015.08.007
$\delta^{87}Sr$	Sr	Natural Soil	53.162‰	24.47	118.08	April, 2018	Active sampling	TIMS	Standard Calibration Method	0.3	doi.org/10.1016/j.atmosenv.2018.10.056
$\delta^{87}Sr$	Sr	Natural Soil	53.420‰	24.47	118.08	April, 2018	Active sampling	TIMS	Standard Calibration Method	0.3	doi.org/10.1016/j.atmosenv.2018.10.056
$\delta^{87}Sr$	Sr	Natural Soil	18.598‰	29.75	-95.28	April, 2022	Active sampling	MC-ICP-MS	Standard Calibration Method	0.08	https://doi.org/10.1016/j.talanta.2022.123236
$\delta^{87}Sr$	Sr	Natural Soil	18.641‰	29.75	-95.28	April, 2022	Active sampling	MC-ICP-MS	Standard Calibration Method	0.08	https://doi.org/10.1016/j.talanta.2022.123236
$\delta^{87}Sr$	Sr	Natural Soil	18.470‰	29.75	-95.28	April, 2022	Active sampling	MC-ICP-MS	Standard Calibration Method	0.08	https://doi.org/10.1016/j.talanta.2022.123236

$\delta^{87}Sr$	Sr	Natural Soil	18.770‰	29.75	-95.28	April, 2022	Active sampling	MC-ICP-MS	Standard Calibration Method	0.08	https://doi.org/10.1016/j.talanta.2022.123236
$\delta^{87}Sr$	Sr	Natural Soil	51.188‰	21.07	107.32	April, 2015	Active sampling	-	Standard Calibration Method	0.3	dx.doi.org/10.1016/j.crte.2015.02.007
$\delta^{87}Sr$	Sr	Non-exhaust emissions	13.992‰	28.67	115.85	2015	Active sampling	TIMS	Standard Calibration Method	0.15	doi.org/10.1016/j.atmosenv.2019.117069
$\delta^{87}Sr$	Sr	Non-exhaust emissions	14.564‰	28.67	115.85	2015	Active sampling	TIMS	Standard Calibration Method	0.15	doi.org/10.1016/j.atmosenv.2019.117069
$\delta^{87}Sr$	Sr	Non-exhaust emissions	15.365‰	28.67	115.85	2015	Active sampling	TIMS	Standard Calibration Method	0.15	doi.org/10.1016/j.atmosenv.2019.117069
$\delta^{87}Sr$	Sr	Non-exhaust emissions	14.907‰	28.67	115.85	2015	Active sampling	TIMS	Standard Calibration Method	0.15	doi.org/10.1016/j.atmosenv.2019.117069
$\delta^{87}Sr$	Sr	Non-exhaust emissions	13.577‰	28.67	115.85	2015	Active sampling	TIMS	Standard Calibration Method	0.15	doi.org/10.1016/j.atmosenv.2019.117069
$\delta^{87}Sr$	Sr	Non-exhaust emissions	14.492‰	28.67	115.85	2015	Active sampling	TIMS	Standard Calibration Method	0.15	doi.org/10.1016/j.atmosenv.2019.117069
$\delta^{87}Sr$	Sr	Non-exhaust emissions	16.610‰	28.67	115.85	2015	Active sampling	TIMS	Standard Calibration Method	0.15	doi.org/10.1016/j.atmosenv.2019.117069
$\delta^{87}Sr$	Sr	Non-exhaust emissions	17.411‰	28.67	115.85	2015	Active sampling	TIMS	Standard Calibration Method	0.15	doi.org/10.1016/j.atmosenv.2019.117069
$\delta^{87}Sr$	Sr	Non-exhaust emissions	14.321‰	28.67	115.85	2015	Active sampling	TIMS	Standard Calibration Method	0.15	doi.org/10.1016/j.atmosenv.2019.117069
$\delta^{87}Sr$	Sr	Non-exhaust emissions	13.992‰	28.67	115.85	11 September, 2013	Active sampling	TIMS	Standard Calibration Method	0.15	doi.org/10.1016/j.atmosenv.2018.12.050
$\delta^{87}Sr$	Sr	Non-exhaust emissions	14.335‰	28.67	115.85	11 September, 2013	Active sampling	TIMS	Standard Calibration Method	0.15	doi.org/10.1016/j.atmosenv.2018.12.050
$\delta^{87}Sr$	Sr	Non-exhaust emissions	14.263‰	28.67	115.85	11 September, 2013	Active sampling	TIMS	Standard Calibration Method	0.15	doi.org/10.1016/j.atmosenv.2018.12.050
$\delta^{87}Sr$	Sr	Non-exhaust emissions	14.550‰	28.67	115.85	11 September, 2013	Active sampling	TIMS	Standard Calibration Method	0.15	doi.org/10.1016/j.atmosenv.2018.12.050

$\delta^{87}Sr$	Sr	Non-exhaust emissions	15.365‰	28.67	115.85	11 September, 2013	Active sampling	TIMS	Standard Calibration Method	0.15	doi.org/10.1016/j.atmosenv.2018.12.050
$\delta^{87}Sr$	Sr	Non-exhaust emissions	14.950‰	28.67	115.85	11 September, 2013	Active sampling	TIMS	Standard Calibration Method	0.15	doi.org/10.1016/j.atmosenv.2018.12.050
$\delta^{87}Sr$	Sr	Non-exhaust emissions	14.907‰	28.67	115.85	11 September, 2013	Active sampling	TIMS	Standard Calibration Method	0.15	doi.org/10.1016/j.atmosenv.2018.12.050
$\delta^{87}Sr$	Sr	Non-exhaust emissions	13.577‰	28.67	115.85	11 September, 2013	Active sampling	TIMS	Standard Calibration Method	0.15	doi.org/10.1016/j.atmosenv.2018.12.050
$\delta^{87}Sr$	Sr	Non-exhaust emissions	14.478‰	28.67	115.85	11 September, 2013	Active sampling	TIMS	Standard Calibration Method	0.15	doi.org/10.1016/j.atmosenv.2018.12.050
$\delta^{87}Sr$	Sr	Non-exhaust emissions	16.610‰	28.67	115.85	11 September, 2013	Active sampling	TIMS	Standard Calibration Method	0.15	doi.org/10.1016/j.atmosenv.2018.12.050
$\delta^{87}Sr$	Sr	Non-exhaust emissions	17.411‰	28.67	115.85	11 September, 2013	Active sampling	TIMS	Standard Calibration Method	0.15	doi.org/10.1016/j.atmosenv.2018.12.050
$\delta^{87}Sr$	Sr	Ore-related Emissions	18.698‰	35.22	113.43	July, 2010	Active sampling	TIMS	Standard Calibration Method	0.22	10.1016/j.atmosenv.2010.06.036
$\delta^{87}Sr$	Sr	Ore-related Emissions	17.039‰	30.27	-97.74	April, 2022	Active sampling	MC-ICP-MS	Standard Calibration Method	0.08	https://doi.org/10.1016/j.talanta.2022.123236
$\delta^{87}Sr$	Sr	Ore-related Emissions	17.096‰	30.27	-97.74	April, 2022	Active sampling	MC-ICP-MS	Standard Calibration Method	0.08	https://doi.org/10.1016/j.talanta.2022.123236
$\delta^{87}Sr$	Sr	Ore-related Emissions	17.253‰	30.27	-97.74	April, 2022	Active sampling	MC-ICP-MS	Standard Calibration Method	0.08	https://doi.org/10.1016/j.talanta.2022.123236
$\delta^{87}Sr$	Sr	Ore-related Emissions	17.583‰	30.27	-97.74	April, 2022	Active sampling	MC-ICP-MS	Standard Calibration Method	0.08	https://doi.org/10.1016/j.talanta.2022.123236
$^{207}Pb/^{206}Pb$	Pb	Coal Combustion	0.90383	26.63	106.62	2017-2018	Active sampling	MC-ICP-MS	Standard Calibration Method	0.02%	https://doi.org/10.1016/j.atmosenv.2022.119503
$^{207}Pb/^{206}Pb$	Pb	Coal Combustion	0.86620	39.92	116.13	January, 2010	Active sampling	MC-ICP-MS	Standard Calibration Method	0.0002	10.1016/j.atmosenv.2010.06.036
$^{207}Pb/^{206}Pb$	Pb	Coal Combustion	0.86580	39.92	116.13	January, 2010	Active sampling	MC-ICP-MS	Standard Calibration Method	0.0002	10.1016/j.atmosenv.2010.06.036

$^{207}Pb/^{206}Pb$	Pb	Coal Combustion	0.85010	39.88	116.32	January, 2010	Active sampling	MC-ICP-MS	Standard Calibration Method	0.0002	10.1016/j.atmosenv.2010.06.036
$^{207}Pb/^{206}Pb$	Pb	Coal Combustion	0.85100	35.07	118.33	January, 2010	Active sampling	MC-ICP-MS	Standard Calibration Method	0.0002	10.1016/j.atmosenv.2010.06.036
$^{207}Pb/^{206}Pb$	Pb	Coal Combustion	0.86000	31.22	121.47	2002	Active sampling	ICP-MS	Standard Calibration Method	0.07%	10.1016/j.atmosenv.2004.10.041
$^{207}Pb/^{206}Pb$	Pb	Coal Combustion	0.85700	31.22	121.47	2002	Active sampling	ICP-MS	Standard Calibration Method	0.07%	10.1016/j.atmosenv.2004.10.041
$^{207}Pb/^{206}Pb$	Pb	Coal Combustion	0.85800	31.22	121.47	2002	Active sampling	ICP-MS	Standard Calibration Method	0.07%	10.1016/j.atmosenv.2004.10.041
$^{207}Pb/^{206}Pb$	Pb	Coal Combustion	0.85960	31.22	121.47	January, 2006	Active sampling	ICP-MS	Standard Calibration Method	0.0002	doi.org/10.1021/ac061365q
$^{207}Pb/^{206}Pb$	Pb	Coal Combustion	0.74444	26.63	106.62	2008	Active sampling	MC-ICP-MS	Standard Calibration Method	0.00012	dx.doi.org/10.1016/j.atmosenv.2015.05.049
$^{207}Pb/^{206}Pb$	Pb	Coal Combustion	0.70043	26.63	106.62	2008	Active sampling	MC-ICP-MS	Standard Calibration Method	0.00012	dx.doi.org/10.1016/j.atmosenv.2015.05.049
$^{207}Pb/^{206}Pb$	Pb	Coal Combustion	0.83188	26.63	106.62	2008	Active sampling	MC-ICP-MS	Standard Calibration Method	0.00012	dx.doi.org/10.1016/j.atmosenv.2015.05.049
$^{207}Pb/^{206}Pb$	Pb	Coal Combustion	0.79847	26.63	106.62	2008	Active sampling	MC-ICP-MS	Standard Calibration Method	0.00012	dx.doi.org/10.1016/j.atmosenv.2015.05.049
$^{207}Pb/^{206}Pb$	Pb	Coal Combustion	0.81453	26.63	106.62	2008	Active sampling	MC-ICP-MS	Standard Calibration Method	0.00012	dx.doi.org/10.1016/j.atmosenv.2015.05.049
$^{207}Pb/^{206}Pb$	Pb	Coal Combustion	0.62842	26.63	106.62	2008	Active sampling	MC-ICP-MS	Standard Calibration Method	0.00012	dx.doi.org/10.1016/j.atmosenv.2015.05.049
$^{207}Pb/^{206}Pb$	Pb	Coal Combustion	0.51353	26.63	106.62	2008	Active sampling	MC-ICP-MS	Standard Calibration Method	0.00012	dx.doi.org/10.1016/j.atmosenv.2015.05.049
$^{207}Pb/^{206}Pb$	Pb	Coal Combustion	0.48504	26.63	106.62	2008	Active sampling	MC-ICP-MS	Standard Calibration Method	0.00012	dx.doi.org/10.1016/j.atmosenv.2015.05.049
$^{207}Pb/^{206}Pb$	Pb	Coal Combustion	0.82393	26.63	106.62	2008	Active sampling	MC-ICP-MS	Standard Calibration Method	0.00012	dx.doi.org/10.1016/j.atmosenv.2015.05.049

$^{207}Pb/^{206}Pb$	Pb	Coal Combustion	0.82754	26.63	106.62	2008	Active sampling	MC-ICP-MS	Standard Calibration Method	0.00012	dx.doi.org/10.1016/j.atmosenv.2015.05.049
$^{207}Pb/^{206}Pb$	Pb	Coal Combustion	0.80418	26.63	106.62	2008	Active sampling	MC-ICP-MS	Standard Calibration Method	0.00012	dx.doi.org/10.1016/j.atmosenv.2015.05.049
$^{207}Pb/^{206}Pb$	Pb	Coal Combustion	0.80090	26.63	106.62	2008	Active sampling	MC-ICP-MS	Standard Calibration Method	0.00012	dx.doi.org/10.1016/j.atmosenv.2015.05.049
$^{207}Pb/^{206}Pb$	Pb	Coal Combustion	0.83900	37.86	112.51	2005	Active sampling	MC-ICP-MS	Standard Calibration Method	0.00012	dx.doi.org/10.1016/j.atmosenv.2015.05.049
$^{207}Pb/^{206}Pb$	Pb	Coal Combustion	0.83717	36.67	117.07	2005	Active sampling	MC-ICP-MS	Standard Calibration Method	0.00012	dx.doi.org/10.1016/j.atmosenv.2015.05.049
$^{207}Pb/^{206}Pb$	Pb	Coal Combustion	0.84452	36.67	117.07	2005	Active sampling	MC-ICP-MS	Standard Calibration Method	0.00012	dx.doi.org/10.1016/j.atmosenv.2015.05.049
$^{207}Pb/^{206}Pb$	Pb	Coal Combustion	0.84034	43.47	87.41	January, 2017	Active sampling	Q-ICP-MS	Standard Calibration Method	0.0019	10.1021/acs.est.7b04119
$^{207}Pb/^{206}Pb$	Pb	Coal Combustion	0.83472	43.47	87.41	April, 2017	Active sampling	Q-ICP-MS	Standard Calibration Method	0.0019	10.1021/acs.est.7b04119
$^{207}Pb/^{206}Pb$	Pb	Coal Combustion	0.83403	43.47	87.41	July, 2017	Active sampling	Q-ICP-MS	Standard Calibration Method	0.0019	10.1021/acs.est.7b04119
$^{207}Pb/^{206}Pb$	Pb	Coal Combustion	0.84034	43.47	87.41	October, 2017	Active sampling	Q-ICP-MS	Standard Calibration Method	0.0019	10.1021/acs.est.7b04119
$^{207}Pb/^{206}Pb$	Pb	Coal Combustion	0.83822	43.47	87.41	January, 2017	Active sampling	Q-ICP-MS	Standard Calibration Method	0.0019	10.1021/acs.est.7b04119
$^{207}Pb/^{206}Pb$	Pb	Coal Combustion	0.83893	43.47	87.41	April, 2017	Active sampling	Q-ICP-MS	Standard Calibration Method	0.0019	10.1021/acs.est.7b04119
$^{207}Pb/^{206}Pb$	Pb	Coal Combustion	0.83752	43.47	87.41	July, 2017	Active sampling	Q-ICP-MS	Standard Calibration Method	0.0019	10.1021/acs.est.7b04119
$^{207}Pb/^{206}Pb$	Pb	Coal Combustion	0.84104	43.47	87.41	October, 2017	Active sampling	Q-ICP-MS	Standard Calibration Method	0.0019	10.1021/acs.est.7b04119
$^{207}Pb/^{206}Pb$	Pb	Coal Combustion	0.84246	43.47	87.41	January, 2017	Active sampling	Q-ICP-MS	Standard Calibration Method	0.0019	10.1021/acs.est.7b04119

$^{207}Pb/^{206}Pb$	Pb	Coal Combustion	0.84602	43.47	87.41	April, 2017	Active sampling	Q-ICP-MS	Standard Calibration Method	0.0019	10.1021/acs.est.7b04119
$^{207}Pb/^{206}Pb$	Pb	Coal Combustion	0.81967	43.47	87.41	July, 2017	Active sampling	Q-ICP-MS	Standard Calibration Method	0.0019	10.1021/acs.est.7b04119
$^{207}Pb/^{206}Pb$	Pb	Coal Combustion	0.83963	36.06	103.83	October, 2017	Active sampling	Q-ICP-MS	Standard Calibration Method	0.0019	10.1021/acs.est.7b04119
$^{207}Pb/^{206}Pb$	Pb	Coal Combustion	0.87413	40.27	111.19	January, 2017	Active sampling	Q-ICP-MS	Standard Calibration Method	0.0019	10.1021/acs.est.7b04119
$^{207}Pb/^{206}Pb$	Pb	Coal Combustion	0.87032	40.27	111.19	April, 2017	Active sampling	Q-ICP-MS	Standard Calibration Method	0.0019	10.1021/acs.est.7b04119
$^{207}Pb/^{206}Pb$	Pb	Coal Combustion	0.87032	40.27	111.19	July, 2017	Active sampling	Q-ICP-MS	Standard Calibration Method	0.0019	10.1021/acs.est.7b04119
$^{207}Pb/^{206}Pb$	Pb	Coal Combustion	0.86730	40.27	111.19	October, 2017	Active sampling	Q-ICP-MS	Standard Calibration Method	0.0019	10.1021/acs.est.7b04119
$^{207}Pb/^{206}Pb$	Pb	Coal Combustion	0.86505	40.27	111.19	January, 2017	Active sampling	Q-ICP-MS	Standard Calibration Method	0.0019	10.1021/acs.est.7b04119
$^{207}Pb/^{206}Pb$	Pb	Coal Combustion	0.87184	40.27	111.19	April, 2017	Active sampling	Q-ICP-MS	Standard Calibration Method	0.0019	10.1021/acs.est.7b04119
$^{207}Pb/^{206}Pb$	Pb	Coal Combustion	0.86957	40.27	111.19	July, 2017	Active sampling	Q-ICP-MS	Standard Calibration Method	0.0019	10.1021/acs.est.7b04119
$^{207}Pb/^{206}Pb$	Pb	Coal Combustion	0.87108	40.27	111.19	October, 2017	Active sampling	Q-ICP-MS	Standard Calibration Method	0.0019	10.1021/acs.est.7b04119
$^{207}Pb/^{206}Pb$	Pb	Coal Combustion	0.86881	40.27	111.19	January, 2017	Active sampling	Q-ICP-MS	Standard Calibration Method	0.0019	10.1021/acs.est.7b04119
$^{207}Pb/^{206}Pb$	Pb	Coal Combustion	0.86207	40.27	111.19	April, 2017	Active sampling	Q-ICP-MS	Standard Calibration Method	0.0019	10.1021/acs.est.7b04119
$^{207}Pb/^{206}Pb$	Pb	Coal Combustion	0.84317	40.27	111.19	July, 2017	Active sampling	Q-ICP-MS	Standard Calibration Method	0.0019	10.1021/acs.est.7b04119
$^{207}Pb/^{206}Pb$	Pb	Coal Combustion	0.84890	40.27	111.19	October, 2017	Active sampling	Q-ICP-MS	Standard Calibration Method	0.0019	10.1021/acs.est.7b04119

$^{207}Pb/^{206}Pb$	Pb	Coal Combustion	0.84175	40.27	111.19	January, 2017	Active sampling	Q-ICP-MS	Standard Calibration Method	0.0019	10.1021/acs.est.7b04119
$^{207}Pb/^{206}Pb$	Pb	Coal Combustion	0.84175	40.27	111.19	April, 2017	Active sampling	Q-ICP-MS	Standard Calibration Method	0.0019	10.1021/acs.est.7b04119
$^{207}Pb/^{206}Pb$	Pb	Coal Combustion	0.84317	43.66	122.33	July, 2017	Active sampling	Q-ICP-MS	Standard Calibration Method	0.0019	10.1021/acs.est.7b04119
$^{207}Pb/^{206}Pb$	Pb	Coal Combustion	0.83752	43.66	122.33	October, 2017	Active sampling	Q-ICP-MS	Standard Calibration Method	0.0019	10.1021/acs.est.7b04119
$^{207}Pb/^{206}Pb$	Pb	Coal Combustion	0.84246	43.66	122.33	January, 2017	Active sampling	Q-ICP-MS	Standard Calibration Method	0.0019	10.1021/acs.est.7b04119
$^{207}Pb/^{206}Pb$	Pb	Coal Combustion	0.84388	43.66	122.33	April, 2017	Active sampling	Q-ICP-MS	Standard Calibration Method	0.0019	10.1021/acs.est.7b04119
$^{207}Pb/^{206}Pb$	Pb	Coal Combustion	0.84388	43.66	122.33	July, 2017	Active sampling	Q-ICP-MS	Standard Calibration Method	0.0019	10.1021/acs.est.7b04119
$^{207}Pb/^{206}Pb$	Pb	Coal Combustion	0.83893	43.66	122.33	October, 2017	Active sampling	Q-ICP-MS	Standard Calibration Method	0.0019	10.1021/acs.est.7b04119
$^{207}Pb/^{206}Pb$	Pb	Coal Combustion	0.83056	43.66	122.33	January, 2017	Active sampling	Q-ICP-MS	Standard Calibration Method	0.0019	10.1021/acs.est.7b04119
$^{207}Pb/^{206}Pb$	Pb	Coal Combustion	0.83752	43.66	122.33	April, 2017	Active sampling	Q-ICP-MS	Standard Calibration Method	0.0019	10.1021/acs.est.7b04119
$^{207}Pb/^{206}Pb$	Pb	Coal Combustion	0.84175	43.66	122.33	July, 2017	Active sampling	Q-ICP-MS	Standard Calibration Method	0.0019	10.1021/acs.est.7b04119
$^{207}Pb/^{206}Pb$	Pb	Coal Combustion	0.84602	43.66	122.33	October, 2017	Active sampling	Q-ICP-MS	Standard Calibration Method	0.0019	10.1021/acs.est.7b04119
$^{207}Pb/^{206}Pb$	Pb	Coal Combustion	0.84104	43.66	122.33	January, 2017	Active sampling	Q-ICP-MS	Standard Calibration Method	0.0019	10.1021/acs.est.7b04119
$^{207}Pb/^{206}Pb$	Pb	Coal Combustion	0.84388	43.66	122.33	April, 2017	Active sampling	Q-ICP-MS	Standard Calibration Method	0.0019	10.1021/acs.est.7b04119
$^{207}Pb/^{206}Pb$	Pb	Coal Combustion	0.84034	43.66	122.33	July, 2017	Active sampling	Q-ICP-MS	Standard Calibration Method	0.0019	10.1021/acs.est.7b04119

$^{207}Pb/^{206}Pb$	Pb	Coal Combustion	0.83822	43.66	122.33	October, 2017	Active sampling	Q-ICP-MS	Standard Calibration Method	0.0019	10.1021/acs.est.7b04119
$^{207}Pb/^{206}Pb$	Pb	Coal Combustion	0.84818	34.54	109.09	January, 2017	Active sampling	Q-ICP-MS	Standard Calibration Method	0.0019	10.1021/acs.est.7b04119
$^{207}Pb/^{206}Pb$	Pb	Coal Combustion	0.85470	34.54	109.09	April, 2017	Active sampling	Q-ICP-MS	Standard Calibration Method	0.0019	10.1021/acs.est.7b04119
$^{207}Pb/^{206}Pb$	Pb	Coal Combustion	0.84034	34.54	109.09	July, 2017	Active sampling	Q-ICP-MS	Standard Calibration Method	0.0019	10.1021/acs.est.7b04119
$^{207}Pb/^{206}Pb$	Pb	Coal Combustion	0.84818	34.54	109.09	October, 2017	Active sampling	Q-ICP-MS	Standard Calibration Method	0.0019	10.1021/acs.est.7b04119
$^{207}Pb/^{206}Pb$	Pb	Coal Combustion	0.84746	34.54	109.09	January, 2017	Active sampling	Q-ICP-MS	Standard Calibration Method	0.0019	10.1021/acs.est.7b04119
$^{207}Pb/^{206}Pb$	Pb	Coal Combustion	0.84746	34.54	109.09	April, 2017	Active sampling	Q-ICP-MS	Standard Calibration Method	0.0019	10.1021/acs.est.7b04119
$^{207}Pb/^{206}Pb$	Pb	Coal Combustion	0.83542	34.54	109.09	July, 2017	Active sampling	Q-ICP-MS	Standard Calibration Method	0.0019	10.1021/acs.est.7b04119
$^{207}Pb/^{206}Pb$	Pb	Coal Combustion	0.84034	34.54	109.09	October, 2017	Active sampling	Q-ICP-MS	Standard Calibration Method	0.0019	10.1021/acs.est.7b04119
$^{207}Pb/^{206}Pb$	Pb	Coal Combustion	0.84388	34.54	109.09	January, 2017	Active sampling	Q-ICP-MS	Standard Calibration Method	0.0019	10.1021/acs.est.7b04119
$^{207}Pb/^{206}Pb$	Pb	Coal Combustion	0.83612	34.54	109.09	April, 2017	Active sampling	Q-ICP-MS	Standard Calibration Method	0.0019	10.1021/acs.est.7b04119
$^{207}Pb/^{206}Pb$	Pb	Coal Combustion	0.84531	34.54	109.09	July, 2017	Active sampling	Q-ICP-MS	Standard Calibration Method	0.0019	10.1021/acs.est.7b04119
$^{207}Pb/^{206}Pb$	Pb	Coal Combustion	0.84104	34.54	109.09	October, 2017	Active sampling	Q-ICP-MS	Standard Calibration Method	0.0019	10.1021/acs.est.7b04119
$^{207}Pb/^{206}Pb$	Pb	Coal Combustion	0.84034	34.54	109.09	January, 2017	Active sampling	Q-ICP-MS	Standard Calibration Method	0.0019	10.1021/acs.est.7b04119
$^{207}Pb/^{206}Pb$	Pb	Coal Combustion	0.83682	34.54	109.09	April, 2017	Active sampling	Q-ICP-MS	Standard Calibration Method	0.0019	10.1021/acs.est.7b04119

$^{207}Pb/^{206}Pb$	Pb	Coal Combustion	0.80710	34.54	109.09	July, 2017	Active sampling	Q-ICP-MS	Standard Calibration Method	0.0019	10.1021/acs.est.7b04119
$^{207}Pb/^{206}Pb$	Pb	Coal Combustion	0.84388	34.54	109.09	October, 2017	Active sampling	Q-ICP-MS	Standard Calibration Method	0.0019	10.1021/acs.est.7b04119
$^{207}Pb/^{206}Pb$	Pb	Coal Combustion	0.83893	37.86	112.51	January, 2017	Active sampling	Q-ICP-MS	Standard Calibration Method	0.0019	10.1021/acs.est.7b04119
$^{207}Pb/^{206}Pb$	Pb	Coal Combustion	0.87413	37.86	112.51	April, 2017	Active sampling	Q-ICP-MS	Standard Calibration Method	0.0019	10.1021/acs.est.7b04119
$^{207}Pb/^{206}Pb$	Pb	Coal Combustion	0.85179	37.86	112.51	July, 2017	Active sampling	Q-ICP-MS	Standard Calibration Method	0.0019	10.1021/acs.est.7b04119
$^{207}Pb/^{206}Pb$	Pb	Coal Combustion	0.85470	37.86	112.51	October, 2017	Active sampling	Q-ICP-MS	Standard Calibration Method	0.0019	10.1021/acs.est.7b04119
$^{207}Pb/^{206}Pb$	Pb	Coal Combustion	0.85397	37.86	112.51	January, 2017	Active sampling	Q-ICP-MS	Standard Calibration Method	0.0019	10.1021/acs.est.7b04119
$^{207}Pb/^{206}Pb$	Pb	Coal Combustion	0.86281	37.86	112.51	April, 2017	Active sampling	Q-ICP-MS	Standard Calibration Method	0.0019	10.1021/acs.est.7b04119
$^{207}Pb/^{206}Pb$	Pb	Coal Combustion	0.85985	37.86	112.51	July, 2017	Active sampling	Q-ICP-MS	Standard Calibration Method	0.0019	10.1021/acs.est.7b04119
$^{207}Pb/^{206}Pb$	Pb	Coal Combustion	0.84459	41.24	119.40	October, 2017	Active sampling	Q-ICP-MS	Standard Calibration Method	0.0019	10.1021/acs.est.7b04119
$^{207}Pb/^{206}Pb$	Pb	Coal Combustion	0.83403	41.24	119.40	January, 2017	Active sampling	Q-ICP-MS	Standard Calibration Method	0.0019	10.1021/acs.est.7b04119
$^{207}Pb/^{206}Pb$	Pb	Coal Combustion	0.84818	41.24	119.40	April, 2017	Active sampling	Q-ICP-MS	Standard Calibration Method	0.0019	10.1021/acs.est.7b04119
$^{207}Pb/^{206}Pb$	Pb	Coal Combustion	0.85251	41.24	119.40	July, 2017	Active sampling	Q-ICP-MS	Standard Calibration Method	0.0019	10.1021/acs.est.7b04119
$^{207}Pb/^{206}Pb$	Pb	Coal Combustion	0.86881	41.24	119.40	October, 2017	Active sampling	Q-ICP-MS	Standard Calibration Method	0.0019	10.1021/acs.est.7b04119
$^{207}Pb/^{206}Pb$	Pb	Coal Combustion	0.83682	41.24	119.40	January, 2017	Active sampling	Q-ICP-MS	Standard Calibration Method	0.0019	10.1021/acs.est.7b04119

$^{207}Pb/^{206}Pb$	Pb	Coal Combustion	0.85690	41.24	119.40	April, 2017	Active sampling	Q-ICP-MS	Standard Calibration Method	0.0019	10.1021/acs.est.7b04119
$^{207}Pb/^{206}Pb$	Pb	Coal Combustion	0.84531	41.24	119.40	July, 2017	Active sampling	Q-ICP-MS	Standard Calibration Method	0.0019	10.1021/acs.est.7b04119
$^{207}Pb/^{206}Pb$	Pb	Coal Combustion	0.89767	41.24	119.40	October, 2017	Active sampling	Q-ICP-MS	Standard Calibration Method	0.0019	10.1021/acs.est.7b04119
$^{207}Pb/^{206}Pb$	Pb	Coal Combustion	0.88496	41.24	119.40	January, 2017	Active sampling	Q-ICP-MS	Standard Calibration Method	0.0019	10.1021/acs.est.7b04119
$^{207}Pb/^{206}Pb$	Pb	Coal Combustion	0.84317	45.75	111.20	April, 2017	Active sampling	Q-ICP-MS	Standard Calibration Method	0.0019	10.1021/acs.est.7b04119
$^{207}Pb/^{206}Pb$	Pb	Coal Combustion	0.84034	45.75	111.20	July, 2017	Active sampling	Q-ICP-MS	Standard Calibration Method	0.0019	10.1021/acs.est.7b04119
$^{207}Pb/^{206}Pb$	Pb	Coal Combustion	0.83126	45.75	111.20	October, 2017	Active sampling	Q-ICP-MS	Standard Calibration Method	0.0019	10.1021/acs.est.7b04119
$^{207}Pb/^{206}Pb$	Pb	Coal Combustion	0.83056	45.75	111.20	January, 2017	Active sampling	Q-ICP-MS	Standard Calibration Method	0.0019	10.1021/acs.est.7b04119
$^{207}Pb/^{206}Pb$	Pb	Coal Combustion	0.83264	45.75	111.20	April, 2017	Active sampling	Q-ICP-MS	Standard Calibration Method	0.0019	10.1021/acs.est.7b04119
$^{207}Pb/^{206}Pb$	Pb	Coal Combustion	0.82919	45.75	111.20	July, 2017	Active sampling	Q-ICP-MS	Standard Calibration Method	0.0019	10.1021/acs.est.7b04119
$^{207}Pb/^{206}Pb$	Pb	Coal Combustion	0.84674	45.75	111.20	October, 2017	Active sampling	Q-ICP-MS	Standard Calibration Method	0.0019	10.1021/acs.est.7b04119
$^{207}Pb/^{206}Pb$	Pb	Coal Combustion	0.84674	45.75	111.20	January, 2017	Active sampling	Q-ICP-MS	Standard Calibration Method	0.0019	10.1021/acs.est.7b04119
$^{207}Pb/^{206}Pb$	Pb	Coal Combustion	0.83612	45.75	111.20	April, 2017	Active sampling	Q-ICP-MS	Standard Calibration Method	0.0019	10.1021/acs.est.7b04119
$^{207}Pb/^{206}Pb$	Pb	Coal Combustion	0.83403	45.75	111.20	July, 2017	Active sampling	Q-ICP-MS	Standard Calibration Method	0.0019	10.1021/acs.est.7b04119
$^{207}Pb/^{206}Pb$	Pb	Coal Combustion	0.85837	36.67	117.07	October, 2017	Active sampling	Q-ICP-MS	Standard Calibration Method	0.0019	10.1021/acs.est.7b04119

$^{207}Pb/^{206}Pb$	Pb	Coal Combustion	0.85763	36.67	117.07	January, 2017	Active sampling	Q-ICP-MS	Standard Calibration Method	0.0019	10.1021/acs.est.7b04119
$^{207}Pb/^{206}Pb$	Pb	Coal Combustion	0.85985	36.67	117.07	April, 2017	Active sampling	Q-ICP-MS	Standard Calibration Method	0.0019	10.1021/acs.est.7b04119
$^{207}Pb/^{206}Pb$	Pb	Coal Combustion	0.85616	36.67	117.07	July, 2017	Active sampling	Q-ICP-MS	Standard Calibration Method	0.0019	10.1021/acs.est.7b04119
$^{207}Pb/^{206}Pb$	Pb	Coal Combustion	0.85837	36.67	117.07	October, 2017	Active sampling	Q-ICP-MS	Standard Calibration Method	0.0019	10.1021/acs.est.7b04119
$^{207}Pb/^{206}Pb$	Pb	Coal Combustion	0.85179	36.67	117.07	January, 2017	Active sampling	Q-ICP-MS	Standard Calibration Method	0.0019	10.1021/acs.est.7b04119
$^{207}Pb/^{206}Pb$	Pb	Coal Combustion	0.84746	36.67	117.07	April, 2017	Active sampling	Q-ICP-MS	Standard Calibration Method	0.0019	10.1021/acs.est.7b04119
$^{207}Pb/^{206}Pb$	Pb	Coal Combustion	0.83264	36.67	117.07	July, 2017	Active sampling	Q-ICP-MS	Standard Calibration Method	0.0019	10.1021/acs.est.7b04119
$^{207}Pb/^{206}Pb$	Pb	Coal Combustion	0.86356	36.67	117.07	October, 2017	Active sampling	Q-ICP-MS	Standard Calibration Method	0.0019	10.1021/acs.est.7b04119
$^{207}Pb/^{206}Pb$	Pb	Coal Combustion	0.86207	36.67	117.07	January, 2017	Active sampling	Q-ICP-MS	Standard Calibration Method	0.0019	10.1021/acs.est.7b04119
$^{207}Pb/^{206}Pb$	Pb	Coal Combustion	0.84531	36.67	117.07	April, 2017	Active sampling	Q-ICP-MS	Standard Calibration Method	0.0019	10.1021/acs.est.7b04119
$^{207}Pb/^{206}Pb$	Pb	Coal Combustion	0.86957	36.67	117.07	July, 2017	Active sampling	Q-ICP-MS	Standard Calibration Method	0.0019	10.1021/acs.est.7b04119
$^{207}Pb/^{206}Pb$	Pb	Coal Combustion	0.87566	36.67	117.07	October, 2017	Active sampling	Q-ICP-MS	Standard Calibration Method	0.0019	10.1021/acs.est.7b04119
$^{207}Pb/^{206}Pb$	Pb	Coal Combustion	0.86430	39.91	116.39	January, 2017	Active sampling	Q-ICP-MS	Standard Calibration Method	0.0019	10.1021/acs.est.7b04119
$^{207}Pb/^{206}Pb$	Pb	Coal Combustion	0.85324	38.01	114.52	April, 2017	Active sampling	Q-ICP-MS	Standard Calibration Method	0.0019	10.1021/acs.est.7b04119
$^{207}Pb/^{206}Pb$	Pb	Coal Combustion	0.85179	38.01	114.52	July, 2017	Active sampling	Q-ICP-MS	Standard Calibration Method	0.0019	10.1021/acs.est.7b04119

$^{207}Pb/^{206}Pb$	Pb	Coal Combustion	0.84602	38.01	114.52	October, 2017	Active sampling	Q-ICP-MS	Standard Calibration Method	0.0019	10.1021/acs.est.7b04119
$^{207}Pb/^{206}Pb$	Pb	Coal Combustion	0.87032	38.01	114.52	January, 2017	Active sampling	Q-ICP-MS	Standard Calibration Method	0.0019	10.1021/acs.est.7b04119
$^{207}Pb/^{206}Pb$	Pb	Coal Combustion	0.85911	38.01	114.52	April, 2017	Active sampling	Q-ICP-MS	Standard Calibration Method	0.0019	10.1021/acs.est.7b04119
$^{207}Pb/^{206}Pb$	Pb	Coal Combustion	0.87260	38.01	114.52	July, 2017	Active sampling	Q-ICP-MS	Standard Calibration Method	0.0019	10.1021/acs.est.7b04119
$^{207}Pb/^{206}Pb$	Pb	Coal Combustion	0.87566	38.01	114.52	October, 2017	Active sampling	Q-ICP-MS	Standard Calibration Method	0.0019	10.1021/acs.est.7b04119
$^{207}Pb/^{206}Pb$	Pb	Coal Combustion	0.85106	38.01	114.52	January, 2017	Active sampling	Q-ICP-MS	Standard Calibration Method	0.0019	10.1021/acs.est.7b04119
$^{207}Pb/^{206}Pb$	Pb	Coal Combustion	0.86806	38.01	114.52	April, 2017	Active sampling	Q-ICP-MS	Standard Calibration Method	0.0019	10.1021/acs.est.7b04119
$^{207}Pb/^{206}Pb$	Pb	Coal Combustion	0.85985	34.80	113.30	July, 2017	Active sampling	Q-ICP-MS	Standard Calibration Method	0.0019	10.1021/acs.est.7b04119
$^{207}Pb/^{206}Pb$	Pb	Coal Combustion	0.86281	34.80	113.30	October, 2017	Active sampling	Q-ICP-MS	Standard Calibration Method	0.0019	10.1021/acs.est.7b04119
$^{207}Pb/^{206}Pb$	Pb	Coal Combustion	0.86881	34.80	113.30	January, 2017	Active sampling	Q-ICP-MS	Standard Calibration Method	0.0019	10.1021/acs.est.7b04119
$^{207}Pb/^{206}Pb$	Pb	Coal Combustion	0.85837	34.80	113.30	April, 2017	Active sampling	Q-ICP-MS	Standard Calibration Method	0.0019	10.1021/acs.est.7b04119
$^{207}Pb/^{206}Pb$	Pb	Coal Combustion	0.85543	34.80	113.30	July, 2017	Active sampling	Q-ICP-MS	Standard Calibration Method	0.0019	10.1021/acs.est.7b04119
$^{207}Pb/^{206}Pb$	Pb	Coal Combustion	0.85690	34.80	113.30	October, 2017	Active sampling	Q-ICP-MS	Standard Calibration Method	0.0019	10.1021/acs.est.7b04119
$^{207}Pb/^{206}Pb$	Pb	Coal Combustion	0.84890	34.80	113.30	January, 2017	Active sampling	Q-ICP-MS	Standard Calibration Method	0.0019	10.1021/acs.est.7b04119
$^{207}Pb/^{206}Pb$	Pb	Coal Combustion	0.80580	30.71	103.83	April, 2017	Active sampling	Q-ICP-MS	Standard Calibration Method	0.0019	10.1021/acs.est.7b04119

$^{207}Pb/^{206}Pb$	Pb	Coal Combustion	0.81433	30.71	103.83	July, 2017	Active sampling	Q-ICP-MS	Standard Calibration Method	0.0019	10.1021/acs.est.7b04119
$^{207}Pb/^{206}Pb$	Pb	Coal Combustion	0.82988	30.71	103.83	October, 2017	Active sampling	Q-ICP-MS	Standard Calibration Method	0.0019	10.1021/acs.est.7b04119
$^{207}Pb/^{206}Pb$	Pb	Coal Combustion	0.82034	30.71	103.83	January, 2017	Active sampling	Q-ICP-MS	Standard Calibration Method	0.0019	10.1021/acs.est.7b04119
$^{207}Pb/^{206}Pb$	Pb	Coal Combustion	0.82169	29.56	40.28	April, 2017	Active sampling	Q-ICP-MS	Standard Calibration Method	0.0019	10.1021/acs.est.7b04119
$^{207}Pb/^{206}Pb$	Pb	Coal Combustion	0.80257	29.56	40.28	July, 2017	Active sampling	Q-ICP-MS	Standard Calibration Method	0.0019	10.1021/acs.est.7b04119
$^{207}Pb/^{206}Pb$	Pb	Coal Combustion	0.80192	29.56	40.28	October, 2017	Active sampling	Q-ICP-MS	Standard Calibration Method	0.0019	10.1021/acs.est.7b04119
$^{207}Pb/^{206}Pb$	Pb	Coal Combustion	0.84246	29.56	40.28	January, 2017	Active sampling	Q-ICP-MS	Standard Calibration Method	0.0019	10.1021/acs.est.7b04119
$^{207}Pb/^{206}Pb$	Pb	Coal Combustion	0.83822	29.56	40.28	April, 2017	Active sampling	Q-ICP-MS	Standard Calibration Method	0.0019	10.1021/acs.est.7b04119
$^{207}Pb/^{206}Pb$	Pb	Coal Combustion	0.84388	26.56	106.47	July, 2017	Active sampling	Q-ICP-MS	Standard Calibration Method	0.0019	10.1021/acs.est.7b04119
$^{207}Pb/^{206}Pb$	Pb	Coal Combustion	0.77340	26.56	106.47	October, 2017	Active sampling	Q-ICP-MS	Standard Calibration Method	0.0019	10.1021/acs.est.7b04119
$^{207}Pb/^{206}Pb$	Pb	Coal Combustion	0.82713	26.56	106.47	January, 2017	Active sampling	Q-ICP-MS	Standard Calibration Method	0.0019	10.1021/acs.est.7b04119
$^{207}Pb/^{206}Pb$	Pb	Coal Combustion	0.72307	26.56	106.47	April, 2017	Active sampling	Q-ICP-MS	Standard Calibration Method	0.0019	10.1021/acs.est.7b04119
$^{207}Pb/^{206}Pb$	Pb	Coal Combustion	0.83056	26.56	106.47	July, 2017	Active sampling	Q-ICP-MS	Standard Calibration Method	0.0019	10.1021/acs.est.7b04119
$^{207}Pb/^{206}Pb$	Pb	Coal Combustion	0.81367	26.56	106.47	October, 2017	Active sampling	Q-ICP-MS	Standard Calibration Method	0.0019	10.1021/acs.est.7b04119
$^{207}Pb/^{206}Pb$	Pb	Coal Combustion	0.78003	26.56	106.47	January, 2017	Active sampling	Q-ICP-MS	Standard Calibration Method	0.0019	10.1021/acs.est.7b04119

$^{207}Pb/^{206}Pb$	Pb	Coal Combustion	0.80710	26.56	106.47	April, 2017	Active sampling	Q-ICP-MS	Standard Calibration Method	0.0019	10.1021/acs.est.7b04119
$^{207}Pb/^{206}Pb$	Pb	Coal Combustion	0.82781	26.56	106.47	July, 2017	Active sampling	Q-ICP-MS	Standard Calibration Method	0.0019	10.1021/acs.est.7b04119
$^{207}Pb/^{206}Pb$	Pb	Coal Combustion	0.82781	26.56	106.47	October, 2017	Active sampling	Q-ICP-MS	Standard Calibration Method	0.0019	10.1021/acs.est.7b04119
$^{207}Pb/^{206}Pb$	Pb	Coal Combustion	0.82576	26.56	106.47	January, 2017	Active sampling	Q-ICP-MS	Standard Calibration Method	0.0019	10.1021/acs.est.7b04119
$^{207}Pb/^{206}Pb$	Pb	Coal Combustion	0.83264	26.56	106.47	April, 2017	Active sampling	Q-ICP-MS	Standard Calibration Method	0.0019	10.1021/acs.est.7b04119
$^{207}Pb/^{206}Pb$	Pb	Coal Combustion	0.82988	26.56	106.47	July, 2017	Active sampling	Q-ICP-MS	Standard Calibration Method	0.0019	10.1021/acs.est.7b04119
$^{207}Pb/^{206}Pb$	Pb	Coal Combustion	0.83264	26.56	106.47	October, 2017	Active sampling	Q-ICP-MS	Standard Calibration Method	0.0019	10.1021/acs.est.7b04119
$^{207}Pb/^{206}Pb$	Pb	Coal Combustion	0.82237	26.56	106.47	January, 2017	Active sampling	Q-ICP-MS	Standard Calibration Method	0.0019	10.1021/acs.est.7b04119
$^{207}Pb/^{206}Pb$	Pb	Coal Combustion	0.83056	26.56	106.47	April, 2017	Active sampling	Q-ICP-MS	Standard Calibration Method	0.0019	10.1021/acs.est.7b04119
$^{207}Pb/^{206}Pb$	Pb	Coal Combustion	0.82169	26.56	106.47	July, 2017	Active sampling	Q-ICP-MS	Standard Calibration Method	0.0019	10.1021/acs.est.7b04119
$^{207}Pb/^{206}Pb$	Pb	Coal Combustion	0.81699	26.56	106.47	October, 2017	Active sampling	Q-ICP-MS	Standard Calibration Method	0.0019	10.1021/acs.est.7b04119
$^{207}Pb/^{206}Pb$	Pb	Coal Combustion	0.81433	26.56	106.47	January, 2017	Active sampling	Q-ICP-MS	Standard Calibration Method	0.0019	10.1021/acs.est.7b04119
$^{207}Pb/^{206}Pb$	Pb	Coal Combustion	0.83195	26.56	106.47	April, 2017	Active sampling	Q-ICP-MS	Standard Calibration Method	0.0019	10.1021/acs.est.7b04119
$^{207}Pb/^{206}Pb$	Pb	Coal Combustion	0.82169	26.56	106.47	July, 2017	Active sampling	Q-ICP-MS	Standard Calibration Method	0.0019	10.1021/acs.est.7b04119
$^{207}Pb/^{206}Pb$	Pb	Coal Combustion	0.81566	26.56	106.47	October, 2017	Active sampling	Q-ICP-MS	Standard Calibration Method	0.0019	10.1021/acs.est.7b04119

$^{207}Pb/^{206}Pb$	Pb	Coal Combustion	0.82372	26.56	106.47	January, 2017	Active sampling	Q-ICP-MS	Standard Calibration Method	0.0019	10.1021/acs.est.7b04119
$^{207}Pb/^{206}Pb$	Pb	Coal Combustion	0.82576	26.56	106.47	April, 2017	Active sampling	Q-ICP-MS	Standard Calibration Method	0.0019	10.1021/acs.est.7b04119
$^{207}Pb/^{206}Pb$	Pb	Coal Combustion	0.82988	26.56	106.47	July, 2017	Active sampling	Q-ICP-MS	Standard Calibration Method	0.0019	10.1021/acs.est.7b04119
$^{207}Pb/^{206}Pb$	Pb	Coal Combustion	0.82372	26.56	106.47	October, 2017	Active sampling	Q-ICP-MS	Standard Calibration Method	0.0019	10.1021/acs.est.7b04119
$^{207}Pb/^{206}Pb$	Pb	Coal Combustion	0.81766	26.56	106.47	January, 2017	Active sampling	Q-ICP-MS	Standard Calibration Method	0.0019	10.1021/acs.est.7b04119
$^{207}Pb/^{206}Pb$	Pb	Coal Combustion	0.84459	26.56	106.47	April, 2017	Active sampling	Q-ICP-MS	Standard Calibration Method	0.0019	10.1021/acs.est.7b04119
$^{207}Pb/^{206}Pb$	Pb	Coal Combustion	0.81301	24.79	102.80	July, 2017	Active sampling	Q-ICP-MS	Standard Calibration Method	0.0019	10.1021/acs.est.7b04119
$^{207}Pb/^{206}Pb$	Pb	Coal Combustion	0.81367	24.79	102.80	October, 2017	Active sampling	Q-ICP-MS	Standard Calibration Method	0.0019	10.1021/acs.est.7b04119
$^{207}Pb/^{206}Pb$	Pb	Coal Combustion	0.83403	24.79	102.80	January, 2017	Active sampling	Q-ICP-MS	Standard Calibration Method	0.0019	10.1021/acs.est.7b04119
$^{207}Pb/^{206}Pb$	Pb	Coal Combustion	0.83403	24.79	102.80	April, 2017	Active sampling	Q-ICP-MS	Standard Calibration Method	0.0019	10.1021/acs.est.7b04119
$^{207}Pb/^{206}Pb$	Pb	Coal Combustion	0.83612	24.79	102.80	July, 2017	Active sampling	Q-ICP-MS	Standard Calibration Method	0.0019	10.1021/acs.est.7b04119
$^{207}Pb/^{206}Pb$	Pb	Coal Combustion	0.83682	24.79	102.80	October, 2017	Active sampling	Q-ICP-MS	Standard Calibration Method	0.0019	10.1021/acs.est.7b04119
$^{207}Pb/^{206}Pb$	Pb	Coal Combustion	0.84531	24.79	102.80	January, 2017	Active sampling	Q-ICP-MS	Standard Calibration Method	0.0019	10.1021/acs.est.7b04119
$^{207}Pb/^{206}Pb$	Pb	Coal Combustion	0.82919	24.79	102.80	April, 2017	Active sampling	Q-ICP-MS	Standard Calibration Method	0.0019	10.1021/acs.est.7b04119
$^{207}Pb/^{206}Pb$	Pb	Coal Combustion	0.83822	24.79	102.80	July, 2017	Active sampling	Q-ICP-MS	Standard Calibration Method	0.0019	10.1021/acs.est.7b04119

$^{207}Pb/^{206}Pb$	Pb	Coal Combustion	0.82988	24.79	102.80	October, 2017	Active sampling	Q-ICP-MS	Standard Calibration Method	0.0019	10.1021/acs.est.7b04119
$^{207}Pb/^{206}Pb$	Pb	Coal Combustion	0.85324	32.06	118.79	January, 2017	Active sampling	Q-ICP-MS	Standard Calibration Method	0.0019	10.1021/acs.est.7b04119
$^{207}Pb/^{206}Pb$	Pb	Coal Combustion	0.84104	32.06	118.79	April, 2017	Active sampling	Q-ICP-MS	Standard Calibration Method	0.0019	10.1021/acs.est.7b04119
$^{207}Pb/^{206}Pb$	Pb	Coal Combustion	0.85397	32.06	118.79	July, 2017	Active sampling	Q-ICP-MS	Standard Calibration Method	0.0019	10.1021/acs.est.7b04119
$^{207}Pb/^{206}Pb$	Pb	Coal Combustion	0.85763	32.06	118.79	October, 2017	Active sampling	Q-ICP-MS	Standard Calibration Method	0.0019	10.1021/acs.est.7b04119
$^{207}Pb/^{206}Pb$	Pb	Coal Combustion	0.84890	31.86	117.29	January, 2017	Active sampling	Q-ICP-MS	Standard Calibration Method	0.0019	10.1021/acs.est.7b04119
$^{207}Pb/^{206}Pb$	Pb	Coal Combustion	0.84962	31.86	117.29	April, 2017	Active sampling	Q-ICP-MS	Standard Calibration Method	0.0019	10.1021/acs.est.7b04119
$^{207}Pb/^{206}Pb$	Pb	Coal Combustion	0.84388	31.86	117.29	July, 2017	Active sampling	Q-ICP-MS	Standard Calibration Method	0.0019	10.1021/acs.est.7b04119
$^{207}Pb/^{206}Pb$	Pb	Coal Combustion	0.85034	31.86	117.29	October, 2017	Active sampling	Q-ICP-MS	Standard Calibration Method	0.0019	10.1021/acs.est.7b04119
$^{207}Pb/^{206}Pb$	Pb	Coal Combustion	0.85179	31.86	117.29	January, 2017	Active sampling	Q-ICP-MS	Standard Calibration Method	0.0019	10.1021/acs.est.7b04119
$^{207}Pb/^{206}Pb$	Pb	Coal Combustion	0.84818	31.86	117.29	April, 2017	Active sampling	Q-ICP-MS	Standard Calibration Method	0.0019	10.1021/acs.est.7b04119
$^{207}Pb/^{206}Pb$	Pb	Coal Combustion	0.84818	31.86	117.29	July, 2017	Active sampling	Q-ICP-MS	Standard Calibration Method	0.0019	10.1021/acs.est.7b04119
$^{207}Pb/^{206}Pb$	Pb	Coal Combustion	0.85837	28.23	112.93	October, 2017	Active sampling	Q-ICP-MS	Standard Calibration Method	0.0019	10.1021/acs.est.7b04119
$^{207}Pb/^{206}Pb$	Pb	Coal Combustion	0.83893	28.23	112.93	October, 2017	Active sampling	Q-ICP-MS	Standard Calibration Method	0.0019	10.1021/acs.est.7b04119
$^{207}Pb/^{206}Pb$	Pb	Coal Combustion	0.83916	39.90	116.40	January, 2021	Active sampling	MC-ICP-MS	Standard Calibration Method	0.0002	doi.org/10.1016/j.scitotenv.2021.145810

$^{207}Pb/^{206}Pb$	Pb	Coal Combustion	0.84543	39.90	116.40	January, 2021	Active sampling	MC-ICP-MS	Standard Calibration Method	0.0002	doi.org/10.1016/j.scitotenv.2021.145810
$^{207}Pb/^{206}Pb$	Pb	Coal Combustion	0.85484	39.90	116.40	January, 2021	Active sampling	MC-ICP-MS	Standard Calibration Method	0.0002	doi.org/10.1016/j.scitotenv.2021.145810
$^{207}Pb/^{206}Pb$	Pb	Coal Combustion	0.85517	39.90	116.40	January, 2021	Active sampling	MC-ICP-MS	Standard Calibration Method	0.0002	doi.org/10.1016/j.scitotenv.2021.145810
$^{207}Pb/^{206}Pb$	Pb	Coal Combustion	0.85595	39.90	116.40	January, 2021	Active sampling	MC-ICP-MS	Standard Calibration Method	0.0002	doi.org/10.1016/j.scitotenv.2021.145810
$^{207}Pb/^{206}Pb$	Pb	Coal Combustion	0.85976	39.90	116.40	January, 2021	Active sampling	MC-ICP-MS	Standard Calibration Method	0.0002	doi.org/10.1016/j.scitotenv.2021.145810
$^{207}Pb/^{206}Pb$	Pb	Coal Combustion	0.86306	39.90	116.40	January, 2021	Active sampling	MC-ICP-MS	Standard Calibration Method	0.0002	doi.org/10.1016/j.scitotenv.2021.145810
$^{207}Pb/^{206}Pb$	Pb	Coal Combustion	0.86333	39.90	116.40	January, 2021	Active sampling	MC-ICP-MS	Standard Calibration Method	0.0002	doi.org/10.1016/j.scitotenv.2021.145810
$^{207}Pb/^{206}Pb$	Pb	Coal Combustion	0.86969	39.90	116.40	January, 2021	Active sampling	MC-ICP-MS	Standard Calibration Method	0.0002	doi.org/10.1016/j.scitotenv.2021.145810
$^{207}Pb/^{206}Pb$	Pb	Coal Combustion	0.87383	39.90	116.40	January, 2021	Active sampling	MC-ICP-MS	Standard Calibration Method	0.0002	doi.org/10.1016/j.scitotenv.2021.145810
$^{207}Pb/^{206}Pb$	Pb	Coal Combustion	0.83146	22.57	88.36	January, 2018	Active sampling	MC-ICP-MS	Standard Calibration Method	0.0002	doi.org/10.1016/j.atmosenv.2018.08.062
$^{207}Pb/^{206}Pb$	Pb	Coal Combustion	0.82665	22.57	88.36	January, 2018	Active sampling	MC-ICP-MS	Standard Calibration Method	0.0002	doi.org/10.1016/j.atmosenv.2018.08.062
$^{207}Pb/^{206}Pb$	Pb	Coal Combustion	0.82768	22.57	88.36	January, 2018	Active sampling	MC-ICP-MS	Standard Calibration Method	0.0002	doi.org/10.1016/j.atmosenv.2018.08.062
$^{207}Pb/^{206}Pb$	Pb	Coal Combustion	0.82102	22.57	88.36	January, 2018	Active sampling	MC-ICP-MS	Standard Calibration Method	0.0002	doi.org/10.1016/j.atmosenv.2018.08.062
$^{207}Pb/^{206}Pb$	Pb	Coal Combustion	0.82884	22.57	88.36	January, 2018	Active sampling	MC-ICP-MS	Standard Calibration Method	0.0002	doi.org/10.1016/j.atmosenv.2018.08.062
$^{207}Pb/^{206}Pb$	Pb	Coal Combustion	0.87026	24.47	118.08	January, 2018	Active sampling	ICP-MS	Standard Calibration Method	0.0002	doi.org/10.1016/j.atmosenv.2018.10.056

$^{207}Pb/^{206}Pb$	Pb	Coal Combustion	0.86957	24.47	118.08	January, 2018	Active sampling		Standard Calibration Method	0.0002	doi.org/10.1016/j.atmosenv.2018.10.056
$^{207}Pb/^{206}Pb$	Pb	Coal Combustion	0.86505	24.47	118.08	January, 2018	Active sampling	ICP-MS	Standard Calibration Method	0.0002	doi.org/10.1016/j.atmosenv.2018.10.056
$^{207}Pb/^{206}Pb$	Pb	Coal Combustion	0.87108	24.47	118.08	January, 2018	Active sampling	ICP-MS	Standard Calibration Method	0.0002	doi.org/10.1016/j.atmosenv.2018.10.056
$^{207}Pb/^{206}Pb$	Pb	Coal Combustion	0.87190	24.47	118.08	January, 2018	Active sampling	ICP-MS	Standard Calibration Method	0.0002	doi.org/10.1016/j.atmosenv.2018.10.056
$^{207}Pb/^{206}Pb$	Pb	Coal Combustion	0.87489	24.47	118.08	January, 2018	Active sampling	ICP-MS	Standard Calibration Method	0.0002	doi.org/10.1016/j.atmosenv.2018.10.056
$^{207}Pb/^{206}Pb$	Pb	Coal Combustion	0.83680	20.84	106.69	January, 2018	Active sampling	MC-ICP-MS	Standard Calibration Method	0.0007	https://doi.org/10.1007/s11356-018-2722-7
$^{207}Pb/^{206}Pb$	Pb	Coal Combustion	0.84140	20.84	106.69	January, 2018	Active sampling	MC-ICP-MS	Standard Calibration Method	0.0007	https://doi.org/10.1007/s11356-018-2722-7
$^{207}Pb/^{206}Pb$	Pb	Coal Combustion	0.84160	20.84	106.69	January, 2018	Active sampling	MC-ICP-MS	Standard Calibration Method	0.0007	https://doi.org/10.1007/s11356-018-2722-7
$^{207}Pb/^{206}Pb$	Pb	Coal Combustion	0.86253	39.11	117.16	11 December, 2020	Active sampling	MC-ICP-MS	Standard Calibration Method	0.0002	http://dx.doi.org/10.1016/j.scitotenv.2023.164567
$^{207}Pb/^{206}Pb$	Pb	Coal Combustion	0.86578	39.11	117.16	11 December, 2020	Active sampling	MC-ICP-MS	Standard Calibration Method	0.0002	http://dx.doi.org/10.1016/j.scitotenv.2023.164567
$^{207}Pb/^{206}Pb$	Pb	Coal Combustion	0.86633	39.11	117.16	11 December, 2020	Active sampling	MC-ICP-MS	Standard Calibration Method	0.0002	http://dx.doi.org/10.1016/j.scitotenv.2023.164567
$^{207}Pb/^{206}Pb$	Pb	Coal Combustion	0.86833	39.11	117.16	11 December, 2020	Active sampling	MC-ICP-MS	Standard Calibration Method	0.0002	http://dx.doi.org/10.1016/j.scitotenv.2023.164567
$^{207}Pb/^{206}Pb$	Pb	Coal Combustion	0.86751	39.11	117.16	11 December, 2020	Active sampling	MC-ICP-MS	Standard Calibration Method	0.0002	http://dx.doi.org/10.1016/j.scitotenv.2023.164567
$^{207}Pb/^{206}Pb$	Pb	Coal Combustion	0.86415	39.11	117.16	11 December, 2020	Active sampling	MC-ICP-MS	Standard Calibration Method	0.0002	http://dx.doi.org/10.1016/j.scitotenv.2023.164567
$^{207}Pb/^{206}Pb$	Pb	Coal Combustion	0.86769	39.11	117.16	8 July, 2019	Active sampling	MC-ICP-MS	Standard Calibration Method	0.0002	http://dx.doi.org/10.1016/j.scitotenv.2023.164567

$^{207}Pb/^{206}Pb$	Pb	Coal Combustion	0.86620	39.11	117.16	8 July, 2019	Active sampling	MC-ICP-MS	Standard Calibration Method	0.0002	http://dx.doi.org/10.1016/j.scitotenv.2023.164567
$^{207}Pb/^{206}Pb$	Pb	Coal Combustion	0.86745	39.11	117.16	8 July, 2019	Active sampling	MC-ICP-MS	Standard Calibration Method	0.0002	http://dx.doi.org/10.1016/j.scitotenv.2023.164567
$^{207}Pb/^{206}Pb$	Pb	Coal Combustion	0.86743	39.11	117.16	8 July, 2019	Active sampling	MC-ICP-MS	Standard Calibration Method	0.0002	http://dx.doi.org/10.1016/j.scitotenv.2023.164567
$^{207}Pb/^{206}Pb$	Pb	Coal Combustion	0.86542	39.11	117.16	20 December, 2020	Active sampling	MC-ICP-MS	Standard Calibration Method	0.0002	http://dx.doi.org/10.1016/j.scitotenv.2023.164567
$^{207}Pb/^{206}Pb$	Pb	Coal Combustion	0.86495	39.11	117.16	20 December, 2020	Active sampling	MC-ICP-MS	Standard Calibration Method	0.0002	http://dx.doi.org/10.1016/j.scitotenv.2023.164567
$^{207}Pb/^{206}Pb$	Pb	Coal Combustion	0.86383	39.11	117.16	20 December, 2020	Active sampling	MC-ICP-MS	Standard Calibration Method	0.0002	http://dx.doi.org/10.1016/j.scitotenv.2023.164567
$^{207}Pb/^{206}Pb$	Pb	Coal Combustion	0.85976	39.11	117.16	2021	Active sampling	MC-ICP-MS	Standard Calibration Method	0.0002	http://dx.doi.org/10.1016/j.scitotenv.2023.164567
$^{207}Pb/^{206}Pb$	Pb	Coal Combustion	0.85818	39.11	117.16	2021	Active sampling	MC-ICP-MS	Standard Calibration Method	0.0002	http://dx.doi.org/10.1016/j.scitotenv.2023.164567
$^{207}Pb/^{206}Pb$	Pb	Coal Combustion	0.85714	39.11	117.16	2021	Active sampling	MC-ICP-MS	Standard Calibration Method	0.0002	http://dx.doi.org/10.1016/j.scitotenv.2023.164567
$^{207}Pb/^{206}Pb$	Pb	Coal Combustion	0.83612	21.07	107.32	January, 2015	Active sampling	-	Standard Calibration Method	0.0002	dx.doi.org/10.1016/j.crte.2015.02.007
$^{207}Pb/^{206}Pb$	Pb	Coal Combustion	0.83612	21.07	107.32	January, 2015	Active sampling	-	Standard Calibration Method	0.0002	dx.doi.org/10.1016/j.crte.2015.02.007
$^{207}Pb/^{206}Pb$	Pb	Vehicle Exhausts-Gasoline	0.91760	48.57	7.75	January, 2008	Active sampling	TIMS	Standard Calibration Method	0.0004	https://doi.org/10.1021/es071704c
$^{207}Pb/^{206}Pb$	Pb	Vehicle Exhausts-Gasoline	0.86505	23.55	-46.63	January, 2018	Active sampling	TIMS	Standard Calibration Method	0.0002	doi.org/10.1016/j.scitotenv.2018.01.192
$^{207}Pb/^{206}Pb$	Pb	Vehicle Exhausts-Gasoline	0.84388	23.55	-46.63	January, 2018	Active sampling	TIMS	Standard Calibration Method	0.0002	doi.org/10.1016/j.scitotenv.2018.01.192
$^{207}Pb/^{206}Pb$	Pb	Vehicle Exhausts-Gasoline	0.86505	23.55	-46.63	January, 2018	Active sampling	TIMS	Standard Calibration Method	0.0002	doi.org/10.1016/j.scitotenv.2018.01.192

$^{207}Pb/^{206}Pb$	Pb	Vehicle Exhausts-Gasoline	0.86580	23.55	-46.63	January, 2018	Active sampling	TIMS	Standard Calibration Method	0.0002	doi.org/10.1016/j.scitotenv.2018.01.192
$^{207}Pb/^{206}Pb$	Pb	Vehicle Exhausts-Gasoline	0.86281	23.55	-46.63	January, 2018	Active sampling	TIMS	Standard Calibration Method	0.0002	doi.org/10.1016/j.scitotenv.2018.01.192
$^{207}Pb/^{206}Pb$	Pb	Vehicle Exhausts-Gasoline	0.88019	31.22	121.47	2002	Active sampling	ICP-MS	Standard Calibration Method	0.07%	10.1016/j.atmosenv.2004.10.041
$^{207}Pb/^{206}Pb$	Pb	Vehicle Exhausts-Gasoline	0.87492	31.22	121.47	2002	Active sampling	ICP-MS	Standard Calibration Method	0.07%	10.1016/j.atmosenv.2004.10.041
$^{207}Pb/^{206}Pb$	Pb	Vehicle Exhausts-Gasoline	0.86349	31.22	121.47	2002	Active sampling	ICP-MS	Standard Calibration Method	0.07%	10.1016/j.atmosenv.2004.10.041
$^{207}Pb/^{206}Pb$	Pb	Vehicle Exhausts-Gasoline	0.87202	31.22	121.47	2002	Active sampling	ICP-MS	Standard Calibration Method	0.07%	10.1016/j.atmosenv.2004.10.041
$^{207}Pb/^{206}Pb$	Pb	Vehicle Exhausts-Gasoline	0.86972	31.22	121.47	2002	Active sampling	ICP-MS	Standard Calibration Method	0.07%	10.1016/j.atmosenv.2004.10.041
$^{207}Pb/^{206}Pb$	Pb	Vehicle Exhausts-Gasoline	0.90100	31.22	121.47	1995	Active sampling	ICP-MS	Standard Calibration Method	0.07%	10.1016/j.atmosenv.2004.10.041
$^{207}Pb/^{206}Pb$	Pb	Vehicle Exhausts-Gasoline	0.87200	31.22	121.47	2002	Active sampling	ICP-MS	Standard Calibration Method	0.07%	10.1016/j.atmosenv.2004.10.041
$^{207}Pb/^{206}Pb$	Pb	Vehicle Exhausts-Gasoline	0.87032	30.36	112.18	January, 2017	Active sampling	Q-ICP-MS	Standard Calibration Method	0.0019	10.1021/acs.est.7b04119
$^{207}Pb/^{206}Pb$	Pb	Vehicle Exhausts-Gasoline	0.87108	30.36	112.18	April, 2017	Active sampling	Q-ICP-MS	Standard Calibration Method	0.0019	10.1021/acs.est.7b04119
$^{207}Pb/^{206}Pb$	Pb	Vehicle Exhausts-Gasoline	0.87184	30.36	112.18	July, 2017	Active sampling	Q-ICP-MS	Standard Calibration Method	0.0019	10.1021/acs.est.7b04119
$^{207}Pb/^{206}Pb$	Pb	Vehicle Exhausts-Gasoline	0.86281	20.00	110.35	October, 2017	Active sampling	Q-ICP-MS	Standard Calibration Method	0.0019	10.1021/acs.est.7b04119
$^{207}Pb/^{206}Pb$	Pb	Vehicle Exhausts-Gasoline	0.87032	20.00	110.35	October, 2017	Active sampling	Q-ICP-MS	Standard Calibration Method	0.0019	10.1021/acs.est.7b04119
$^{207}Pb/^{206}Pb$	Pb	Vehicle Exhausts-Gasoline	0.86440	20.84	106.69	January, 2018	Active sampling	MC-ICP-MS	Standard Calibration Method	0.0007	https://doi.org/10.1007/s11356-018-2722-7

$^{207}Pb/^{206}Pb$	Pb	Vehicle Exhausts-Gasoline	0.87290	20.84	106.69	January, 2018	Active sampling	MC-ICP-MS	Standard Calibration Method	0.0007	https://doi.org/10.1007/s11356-018-2722-7
$^{207}Pb/^{206}Pb$	Pb	Vehicle Exhausts-Gasoline	0.87160	20.84	106.69	January, 2018	Active sampling	MC-ICP-MS	Standard Calibration Method	0.0007	https://doi.org/10.1007/s11356-018-2722-7
$^{207}Pb/^{206}Pb$	Pb	Vehicle Exhausts-Gasoline	0.92319	26.63	106.62	2017-2018	Active sampling	MC-ICP-MS	Standard Calibration Method	0.02%	https://doi.org/10.1016/j.atmosenv.2022.119503
$^{207}Pb/^{206}Pb$	Pb	Vehicle Exhausts-Gasoline	0.90090	31.22	121.47	January, 2006	Active sampling	ICP-MS	Standard Calibration Method	0.0002	doi.org/10.1021/ac061365q
$^{207}Pb/^{206}Pb$	Pb	Vehicle Exhausts-Gasoline	0.87150	31.22	121.47	January, 2006	Active sampling	ICP-MS	Standard Calibration Method	0.0002	doi.org/10.1021/ac061365q
$^{207}Pb/^{206}Pb$	Pb	Vehicle Exhausts-Gasoline	0.85911	21.02	107.35	January, 2015	Active sampling	-	Standard Calibration Method	0.0002	dx.doi.org/10.1016/j.crte.2015.02.007
$^{207}Pb/^{206}Pb$	Pb	Vehicle Exhausts-Gasoline	0.85985	21.02	107.35	January, 2015	Active sampling	-	Standard Calibration Method	0.0002	dx.doi.org/10.1016/j.crte.2015.02.007
$^{207}Pb/^{206}Pb$	Pb	Vehicle Exhausts-Gasoline	0.85994	21.02	107.35	January, 2015	Active sampling	-	Standard Calibration Method	0.0002	dx.doi.org/10.1016/j.crte.2015.02.007
$^{207}Pb/^{206}Pb$	Pb	Vehicle Exhausts-Gasoline	0.88014	21.02	107.35	January, 2015	Active sampling	-	Standard Calibration Method	0.0002	dx.doi.org/10.1016/j.crte.2015.02.007
$^{207}Pb/^{206}Pb$	Pb	Vehicle Exhausts-Gasoline	0.87482	21.02	107.35	January, 2015	Active sampling	-	Standard Calibration Method	0.0002	dx.doi.org/10.1016/j.crte.2015.02.007
$^{207}Pb/^{206}Pb$	Pb	Vehicle Exhausts-Gasoline	0.87147	21.02	107.35	January, 2015	Active sampling	-	Standard Calibration Method	0.0002	dx.doi.org/10.1016/j.crte.2015.02.007
$^{207}Pb/^{206}Pb$	Pb	Vehicle Exhausts-Gasoline	0.85317	26.57	101.72	January, 2019	Active sampling	MC-ICP-MS	Standard Calibration Method	0.0002	doi.org/10.1016/j.apr.2019.10.014
$^{207}Pb/^{206}Pb$	Pb	Vehicle Exhausts-Gasoline	0.85419	26.57	101.72	January, 2019	Active sampling	MC-ICP-MS	Standard Calibration Method	0.0002	doi.org/10.1016/j.apr.2019.10.014
$^{207}Pb/^{206}Pb$	Pb	Vehicle Exhausts-Gasoline	0.87637	26.57	101.72	January, 2019	Active sampling	MC-ICP-MS	Standard Calibration Method	0.0002	doi.org/10.1016/j.apr.2019.10.014
$^{207}Pb/^{206}Pb$	Pb	Vehicle Exhausts-Gasoline	0.87032	24.47	118.08	January, 2018	Active sampling	ICP-MS	Standard Calibration Method	0.0002	doi.org/10.1016/j.atmosenv.2018.10.056

$^{207}Pb/^{206}Pb$	Pb	Vehicle Exhausts-Gasoline	0.87260	24.47	118.08	January, 2018	Active sampling	ICP-MS	Standard Calibration Method	0.0002	doi.org/10.1016/j.atmosenv.2018.10.056
$^{207}Pb/^{206}Pb$	Pb	Vehicle Exhausts-Gasoline	0.88574	24.47	118.08	January, 2018	Active sampling	ICP-MS	Standard Calibration Method	0.0002	doi.org/10.1016/j.atmosenv.2018.10.056
$^{207}Pb/^{206}Pb$	Pb	Vehicle Exhausts-Gasoline	0.88889	24.47	118.08	January, 2018	Active sampling	ICP-MS	Standard Calibration Method	0.0002	doi.org/10.1016/j.atmosenv.2018.10.056
$^{207}Pb/^{206}Pb$	Pb	Vehicle Exhausts-Gasoline	0.89120	24.47	118.08	January, 2018	Active sampling	ICP-MS	Standard Calibration Method	0.0002	doi.org/10.1016/j.atmosenv.2018.10.056
$^{207}Pb/^{206}Pb$	Pb	Vehicle Exhausts-Gasoline	0.90009	24.47	118.08	January, 2018	Active sampling	ICP-MS	Standard Calibration Method	0.0002	doi.org/10.1016/j.atmosenv.2018.10.056
$^{207}Pb/^{206}Pb$	Pb	Vehicle Exhausts-Gasoline	0.88810	48.57	7.75	January, 2008	Active sampling	TIMS	Standard Calibration Method	0.0006	10.1016/j.apgeochem.2008.02.004
$^{207}Pb/^{206}Pb$	Pb	Vehicle Exhausts-Gasoline	0.89815	48.57	7.75	January, 2008	Active sampling	TIMS	Standard Calibration Method	0.0006	10.1016/j.apgeochem.2008.02.004
$^{207}Pb/^{206}Pb$	Pb	Vehicle Exhausts-Gasoline	0.91760	48.57	7.75	January, 2008	Active sampling	TIMS	Standard Calibration Method	0.0006	10.1016/j.apgeochem.2008.02.004
$^{207}Pb/^{206}Pb$	Pb	Vehicle Exhausts-Gasoline	0.86521	39.11	117.16	22 November, 2019	Active sampling	MC-ICP-MS	Standard Calibration Method	0.0002	http://dx.doi.org/10.1016/j.scitotenv.2023.164567
$^{207}Pb/^{206}Pb$	Pb	Vehicle Exhausts-Gasoline	0.86592	39.11	117.16	22 November, 2019	Active sampling	MC-ICP-MS	Standard Calibration Method	0.0002	http://dx.doi.org/10.1016/j.scitotenv.2023.164567
$^{207}Pb/^{206}Pb$	Pb	Vehicle Exhausts-Gasoline	0.86973	39.11	117.16	22 November, 2019	Active sampling	MC-ICP-MS	Standard Calibration Method	0.0002	http://dx.doi.org/10.1016/j.scitotenv.2023.164567
$^{207}Pb/^{206}Pb$	Pb	Vehicle Exhausts-Gasoline	0.86824	39.11	117.16	22 November, 2019	Active sampling	MC-ICP-MS	Standard Calibration Method	0.0002	http://dx.doi.org/10.1016/j.scitotenv.2023.164567
$^{207}Pb/^{206}Pb$	Pb	Vehicle Exhausts-Gasoline	0.86599	39.11	117.16	22 November, 2019	Active sampling	MC-ICP-MS	Standard Calibration Method	0.0002	http://dx.doi.org/10.1016/j.scitotenv.2023.164567
$^{207}Pb/^{206}Pb$	Pb	Vehicle Exhausts-Gasoline	0.86693	39.11	117.16	22 November, 2019	Active sampling	MC-ICP-MS	Standard Calibration Method	0.0002	http://dx.doi.org/10.1016/j.scitotenv.2023.164567
$^{207}Pb/^{206}Pb$	Pb	Vehicle Exhausts-Gasoline	0.86585	39.11	117.16	22 November, 2019	Active sampling	MC-ICP-MS	Standard Calibration Method	0.0002	http://dx.doi.org/10.1016/j.scitotenv.2023.164567

$^{207}Pb/^{206}Pb$	Pb	Vehicle Exhausts-Gasoline	0.86655	39.11	117.16	22 November, 2019	Active sampling	MC-ICP-MS	Standard Calibration Method	0.0002	http://dx.doi.org/10.1016/j.scitotenv.2023.164567
$^{207}Pb/^{206}Pb$	Pb	Vehicle Exhausts-Gasoline	0.86578	39.11	117.16	22 November, 2019	Active sampling	MC-ICP-MS	Standard Calibration Method	0.0002	http://dx.doi.org/10.1016/j.scitotenv.2023.164567
$^{207}Pb/^{206}Pb$	Pb	Vehicle Exhausts-Gasoline	0.86934	26.63	106.62	2008	Active sampling	MC-ICP-MS	Standard Calibration Method	0.00012	dx.doi.org/10.1016/j.atmosenv.2015.05.049
$^{207}Pb/^{206}Pb$	Pb	Vehicle Exhausts-Gasoline	0.86685	26.63	106.62	2008	Active sampling	MC-ICP-MS	Standard Calibration Method	0.00012	dx.doi.org/10.1016/j.atmosenv.2015.05.049
$^{207}Pb/^{206}Pb$	Pb	Vehicle Exhausts-Gasoline	0.86051	26.63	106.62	2008	Active sampling	MC-ICP-MS	Standard Calibration Method	0.00012	dx.doi.org/10.1016/j.atmosenv.2015.05.049
$^{207}Pb/^{206}Pb$	Pb	Vehicle Exhausts-Gasoline	0.86244	26.63	106.62	2008	Active sampling	MC-ICP-MS	Standard Calibration Method	0.00012	dx.doi.org/10.1016/j.atmosenv.2015.05.049
$^{207}Pb/^{206}Pb$	Pb	Vehicle Exhausts-Diesel	0.86237	48.57	7.75	January, 2008	Active sampling	TIMS	Standard Calibration Method	0.0004	https://doi.org/10.1021/es071704c
$^{207}Pb/^{206}Pb$	Pb	Vehicle Exhausts-Diesel	0.86348	48.57	7.75	January, 2008	Active sampling	TIMS	Standard Calibration Method	0.0004	https://doi.org/10.1021/es071704c
$^{207}Pb/^{206}Pb$	Pb	Vehicle Exhausts-Diesel	0.86430	23.55	-46.63	January, 2018	Active sampling	TIMS	Standard Calibration Method	0.0002	doi.org/10.1016/j.scitotenv.2018.01.192
$^{207}Pb/^{206}Pb$	Pb	Vehicle Exhausts-Diesel	0.86281	23.55	-46.63	January, 2018	Active sampling	TIMS	Standard Calibration Method	0.0002	doi.org/10.1016/j.scitotenv.2018.01.192
$^{207}Pb/^{206}Pb$	Pb	Vehicle Exhausts-Diesel	0.84602	23.55	-46.63	January, 2018	Active sampling	TIMS	Standard Calibration Method	0.0002	doi.org/10.1016/j.scitotenv.2018.01.192
$^{207}Pb/^{206}Pb$	Pb	Vehicle Exhausts-Diesel	0.88417	23.55	-46.63	January, 2018	Active sampling	TIMS	Standard Calibration Method	0.0002	doi.org/10.1016/j.scitotenv.2018.01.192
$^{207}Pb/^{206}Pb$	Pb	Vehicle Exhausts-Diesel	0.86059	30.36	112.18	January, 2017	Active sampling	Q-ICP-MS	Standard Calibration Method	0.0019	10.1021/acs.est.7b04119
$^{207}Pb/^{206}Pb$	Pb	Vehicle Exhausts-Diesel	0.85911	30.36	112.18	April, 2017	Active sampling	Q-ICP-MS	Standard Calibration Method	0.0019	10.1021/acs.est.7b04119
$^{207}Pb/^{206}Pb$	Pb	Vehicle Exhausts-Diesel	0.85985	30.36	112.18	July, 2017	Active sampling	Q-ICP-MS	Standard Calibration Method	0.0019	10.1021/acs.est.7b04119

$^{207}Pb/^{206}Pb$	Pb	Vehicle Exhausts-Diesel	0.86133	30.36	112.18	October, 2017	Active sampling	Q-ICP-MS	Standard Calibration Method	0.0019	10.1021/acs.est.7b04119
$^{207}Pb/^{206}Pb$	Pb	Vehicle Exhausts-Diesel	0.85985	30.36	112.18	April, 2017	Active sampling	Q-ICP-MS	Standard Calibration Method	0.0019	10.1021/acs.est.7b04119
$^{207}Pb/^{206}Pb$	Pb	Vehicle Exhausts-Diesel	0.85985	30.36	112.18	July, 2017	Active sampling	Q-ICP-MS	Standard Calibration Method	0.0019	10.1021/acs.est.7b04119
$^{207}Pb/^{206}Pb$	Pb	Vehicle Exhausts-Diesel	0.85397	20.00	110.35	October, 2017	Active sampling	Q-ICP-MS	Standard Calibration Method	0.0019	10.1021/acs.est.7b04119
$^{207}Pb/^{206}Pb$	Pb	Vehicle Exhausts-Diesel	0.86380	20.84	106.69	January, 2018	Active sampling	MC-ICP-MS	Standard Calibration Method	0.0007	https://doi.org/10.1007/s11356-018-2722-7
$^{207}Pb/^{206}Pb$	Pb	Vehicle Exhausts-Diesel	0.86540	20.84	106.69	January, 2018	Active sampling	MC-ICP-MS	Standard Calibration Method	0.0007	https://doi.org/10.1007/s11356-018-2722-7
$^{207}Pb/^{206}Pb$	Pb	Vehicle Exhausts-Diesel	0.86560	20.84	106.69	January, 2018	Active sampling	MC-ICP-MS	Standard Calibration Method	0.0007	https://doi.org/10.1007/s11356-018-2722-7
$^{207}Pb/^{206}Pb$	Pb	Vehicle Exhausts-Diesel	0.92473	36.20	113.12	2017-2018	Active sampling	MC-ICP-MS	Standard Calibration Method	0.02%	https://doi.org/10.1016/j.atmosenv.2022.119503
$^{207}Pb/^{206}Pb$	Pb	Natural Soil	0.85907	32.25	-110.79	January, 2014	Active sampling	ICP-MS	Standard Calibration Method	0.50%	https://doi.org/10.1016/j.chemosphere.2014.11.057
$^{207}Pb/^{206}Pb$	Pb	Natural Soil	0.85522	32.25	-110.79	January, 2014	Active sampling	ICP-MS	Standard Calibration Method	0.50%	https://doi.org/10.1016/j.chemosphere.2014.11.057
$^{207}Pb/^{206}Pb$	Pb	Natural Soil	0.85739	32.25	-110.79	January, 2014	Active sampling	ICP-MS	Standard Calibration Method	0.50%	https://doi.org/10.1016/j.chemosphere.2014.11.057
$^{207}Pb/^{206}Pb$	Pb	Natural Soil	0.85642	32.25	-110.79	January, 2014	Active sampling	ICP-MS	Standard Calibration Method	0.50%	https://doi.org/10.1016/j.chemosphere.2014.11.057
$^{207}Pb/^{206}Pb$	Pb	Natural Soil	0.85381	32.25	-110.79	January, 2014	Active sampling	ICP-MS	Standard Calibration Method	0.50%	https://doi.org/10.1016/j.chemosphere.2014.11.057
$^{207}Pb/^{206}Pb$	Pb	Natural Soil	0.83963	32.25	-110.79	January, 2014	Active sampling	ICP-MS	Standard Calibration Method	0.50%	https://doi.org/10.1016/j.chemosphere.2014.11.057
$^{207}Pb/^{206}Pb$	Pb	Non-exhaust emissions-Brake Pad	0.81967	51.50	-0.12	January, 2017	Active sampling	MC-ICP-MS	Standard Calibration Method	0.0002	http://dx.doi.org/10.1016/j.atmosenv.2017.06.020

$^{207}Pb/^{206}Pb$	Pb	Non-exhaust emissions-Brake Pad	0.79700	51.50	-0.12	January, 2017	Active sampling	MC-ICP-MS	Standard Calibration Method	0.0002	http://dx.doi.org/10.1016/j.atmosenv.2017.06.020
$^{207}Pb/^{206}Pb$	Pb	Non-exhaust emissions-Brake Pad	0.85830	51.50	-0.12	January, 2017	Active sampling	MC-ICP-MS	Standard Calibration Method	0.0002	http://dx.doi.org/10.1016/j.atmosenv.2017.06.020
$^{207}Pb/^{206}Pb$	Pb	Non-exhaust emissions-Brake Pad	0.77973	35.21	129.07	July, 2021	Active sampling	MC-ICP-MS	Standard Calibration Method	0.0002	https://doi.org/10.1016/j.envpol.2021.118339
$^{207}Pb/^{206}Pb$	Pb	Non-exhaust emissions-Brake Pad	0.77736	35.21	129.07	July, 2021	Active sampling	MC-ICP-MS	Standard Calibration Method	0.0002	https://doi.org/10.1016/j.envpol.2021.118339
$^{207}Pb/^{206}Pb$	Pb	Non-exhaust emissions-Brake Pad	0.61652	35.21	129.07	July, 2021	Active sampling	MC-ICP-MS	Standard Calibration Method	0.0002	https://doi.org/10.1016/j.envpol.2021.118339
$^{207}Pb/^{206}Pb$	Pb	Non-exhaust emissions-Brake Pad	0.85281	35.21	129.07	July, 2021	Active sampling	MC-ICP-MS	Standard Calibration Method	0.0002	https://doi.org/10.1016/j.envpol.2021.118339
$^{207}Pb/^{206}Pb$	Pb	Non-exhaust emissions-Brake Pad	0.78370	35.21	129.07	July, 2021	Active sampling	MC-ICP-MS	Standard Calibration Method	0.0002	https://doi.org/10.1016/j.envpol.2021.118339
$^{207}Pb/^{206}Pb$	Pb	Non-exhaust emissions-Brake Pad	0.85874	35.21	129.07	July, 2021	Active sampling	MC-ICP-MS	Standard Calibration Method	0.0002	https://doi.org/10.1016/j.envpol.2021.118339
$^{207}Pb/^{206}Pb$	Pb	Non-exhaust emissions-Brake Pad	0.85852	35.21	129.07	July, 2021	Active sampling	MC-ICP-MS	Standard Calibration Method	0.0002	https://doi.org/10.1016/j.envpol.2021.118339
$^{207}Pb/^{206}Pb$	Pb	Non-exhaust emissions-Brake Pad	0.83563	35.21	129.07	July, 2021	Active sampling	MC-ICP-MS	Standard Calibration Method	0.0002	https://doi.org/10.1016/j.envpol.2021.118339
$^{207}Pb/^{206}Pb$	Pb	Non-exhaust emissions-Brake Pad	0.82291	35.21	129.07	July, 2021	Active sampling	MC-ICP-MS	Standard Calibration Method	0.0002	https://doi.org/10.1016/j.envpol.2021.118339
$^{207}Pb/^{206}Pb$	Pb	Non-exhaust emissions-Brake Pad	0.85704	35.21	129.07	July, 2021	Active sampling	MC-ICP-MS	Standard Calibration Method	0.0002	https://doi.org/10.1016/j.envpol.2021.118339
$^{207}Pb/^{206}Pb$	Pb	Non-exhaust emissions-Brake Pad	0.85631	35.21	129.07	July, 2021	Active sampling	MC-ICP-MS	Standard Calibration Method	0.0002	https://doi.org/10.1016/j.envpol.2021.118339
$^{207}Pb/^{206}Pb$	Pb	Non-exhaust emissions-Brake Pad	0.82501	35.21	129.07	July, 2021	Active sampling	MC-ICP-MS	Standard Calibration Method	0.0002	https://doi.org/10.1016/j.envpol.2021.118339
$^{207}Pb/^{206}Pb$	Pb	Non-exhaust emissions-Brake Pad	0.84048	35.21	129.07	July, 2021	Active sampling	MC-ICP-MS	Standard Calibration Method	0.0002	https://doi.org/10.1016/j.envpol.2021.118339

$^{207}Pb/^{206}Pb$	Pb	Non-exhaust emissions-Brake Pad	0.82953	47.05	-77.01	July, 2021	Active sampling	MC-ICP-MS	Standard Calibration Method	0.0002	https://doi.org/10.1016/j.envpol.2021.118339
$^{207}Pb/^{206}Pb$	Pb	Non-exhaust emissions-Brake Pad	0.51343	47.05	-77.01	July, 2021	Active sampling	MC-ICP-MS	Standard Calibration Method	0.0002	https://doi.org/10.1016/j.envpol.2021.118339
$^{207}Pb/^{206}Pb$	Pb	Non-exhaust emissions-Brake Pad	0.84168	52.51	13.03	July, 2021	Active sampling	MC-ICP-MS	Standard Calibration Method	0.0002	https://doi.org/10.1016/j.envpol.2021.118339
$^{207}Pb/^{206}Pb$	Pb	Non-exhaust emissions-Brake Pad	0.83563	35.21	129.07	July, 2021	Active sampling	MC-ICP-MS	Standard Calibration Method	0.0002	https://doi.org/10.1016/j.envpol.2021.118339
$^{207}Pb/^{206}Pb$	Pb	Non-exhaust emissions-Brake Pad	0.83556	35.21	129.07	July, 2021	Active sampling	MC-ICP-MS	Standard Calibration Method	0.0002	https://doi.org/10.1016/j.envpol.2021.118339
$^{207}Pb/^{206}Pb$	Pb	Non-exhaust emissions-Brake Pad	0.84545	35.21	129.07	July, 2021	Active sampling	MC-ICP-MS	Standard Calibration Method	0.0002	https://doi.org/10.1016/j.envpol.2021.118339
$^{207}Pb/^{206}Pb$	Pb	Non-exhaust emissions-Brake Pad	0.84524	35.21	129.07	July, 2021	Active sampling	MC-ICP-MS	Standard Calibration Method	0.0002	https://doi.org/10.1016/j.envpol.2021.118339
$^{207}Pb/^{206}Pb$	Pb	Non-exhaust emissions-Tire	0.85288	23.55	-46.63	January, 2018	Active sampling	TIMS	Standard Calibration Method	0.0002	doi.org/10.1016/j.scitotenv.2018.01.192
$^{207}Pb/^{206}Pb$	Pb	Non-exhaust emissions-Tire	0.85749	23.55	-46.63	January, 2018	Active sampling	TIMS	Standard Calibration Method	0.0002	doi.org/10.1016/j.scitotenv.2018.01.192
$^{207}Pb/^{206}Pb$	Pb	Non-exhaust emissions-Tire	0.85543	23.55	-46.63	January, 2018	Active sampling	TIMS	Standard Calibration Method	0.0002	doi.org/10.1016/j.scitotenv.2018.01.192
$^{207}Pb/^{206}Pb$	Pb	Non-exhaust emissions-Tire	0.86214	23.55	-46.63	January, 2018	Active sampling	TIMS	Standard Calibration Method	0.0002	doi.org/10.1016/j.scitotenv.2018.01.192
$^{207}Pb/^{206}Pb$	Pb	Non-exhaust emissions-Tire	0.83836	23.55	-46.63	January, 2018	Active sampling	TIMS	Standard Calibration Method	0.0002	doi.org/10.1016/j.scitotenv.2018.01.192
$^{207}Pb/^{206}Pb$	Pb	Non-exhaust emissions-Tire	0.83956	23.55	-46.63	January, 2018	Active sampling	TIMS	Standard Calibration Method	0.0002	doi.org/10.1016/j.scitotenv.2018.01.192
$^{207}Pb/^{206}Pb$	Pb	Non-exhaust emissions-Tire	0.86941	51.50	-0.12	January, 2017	Active sampling	MC-ICP-MS	Standard Calibration Method	0.0002	http://dx.doi.org/10.1016/j.atmosenv.2017.06.020
$^{207}Pb/^{206}Pb$	Pb	Non-exhaust emissions-Tire	0.85763	51.50	-0.12	January, 2017	Active sampling	MC-ICP-MS	Standard Calibration Method	0.0002	http://dx.doi.org/10.1016/j.atmosenv.2017.06.020

$^{207}Pb/^{206}Pb$	Pb	Non-exhaust emissions-Tire	0.85815	51.50	-0.12	January, 2017	Active sampling	MC-ICP-MS	Standard Calibration Method	0.0002	http://dx.doi.org/10.1016/j.atmosenv.2017.06.020
$^{207}Pb/^{206}Pb$	Pb	Non-exhaust emissions-Tire	0.86110	51.50	-0.12	January, 2017	Active sampling	MC-ICP-MS	Standard Calibration Method	0.0002	http://dx.doi.org/10.1016/j.atmosenv.2017.06.020
$^{207}Pb/^{206}Pb$	Pb	Non-exhaust emissions-Tire	0.86096	35.21	129.07	July, 2021	Active sampling	MC-ICP-MS	Standard Calibration Method	0.0002	https://doi.org/10.1016/j.envpol.2021.118339
$^{207}Pb/^{206}Pb$	Pb	Non-exhaust emissions-Tire	0.86588	35.21	129.07	July, 2021	Active sampling	MC-ICP-MS	Standard Calibration Method	0.0002	https://doi.org/10.1016/j.envpol.2021.118339
$^{207}Pb/^{206}Pb$	Pb	Non-exhaust emissions-Road paint	0.85631	35.21	129.07	July, 2021	Active sampling	MC-ICP-MS	Standard Calibration Method	0.0002	https://doi.org/10.1016/j.envpol.2021.118339
$^{207}Pb/^{206}Pb$	Pb	Non-exhaust emissions-Road paint	0.85624	35.21	129.07	July, 2021	Active sampling	MC-ICP-MS	Standard Calibration Method	0.0002	https://doi.org/10.1016/j.envpol.2021.118339
$^{207}Pb/^{206}Pb$	Pb	Non-exhaust emissions-Road paint	0.87047	35.21	129.07	July, 2021	Active sampling	MC-ICP-MS	Standard Calibration Method	0.0002	https://doi.org/10.1016/j.envpol.2021.118339
$^{207}Pb/^{206}Pb$	Pb	Non-exhaust emissions-Road paint	0.88535	35.21	129.07	July, 2021	Active sampling	MC-ICP-MS	Standard Calibration Method	0.0002	https://doi.org/10.1016/j.envpol.2021.118339
$^{207}Pb/^{206}Pb$	Pb	Non-exhaust emissions-Road paint	0.76905	35.21	129.07	July, 2021	Active sampling	MC-ICP-MS	Standard Calibration Method	0.0002	https://doi.org/10.1016/j.envpol.2021.118339
$^{207}Pb/^{206}Pb$	Pb	Non-exhaust emissions-Road paint	0.78567	35.21	129.07	July, 2021	Active sampling	MC-ICP-MS	Standard Calibration Method	0.0002	https://doi.org/10.1016/j.envpol.2021.118339
$^{207}Pb/^{206}Pb$	Pb	Non-exhaust emissions-Road paint	0.77525	35.21	129.07	July, 2021	Active sampling	MC-ICP-MS	Standard Calibration Method	0.0002	https://doi.org/10.1016/j.envpol.2021.118339
$^{207}Pb/^{206}Pb$	Pb	Non-exhaust emissions-Road paint	0.85339	35.21	129.07	July, 2021	Active sampling	MC-ICP-MS	Standard Calibration Method	0.0002	https://doi.org/10.1016/j.envpol.2021.118339
$^{207}Pb/^{206}Pb$	Pb	Non-exhaust emissions-Road paint	0.69950	35.21	129.07	July, 2021	Active sampling	MC-ICP-MS	Standard Calibration Method	0.0002	https://doi.org/10.1016/j.envpol.2021.118339
$^{207}Pb/^{206}Pb$	Pb	Non-exhaust emissions-Road paint	0.76144	35.21	129.07	July, 2021	Active sampling	MC-ICP-MS	Standard Calibration Method	0.0002	https://doi.org/10.1016/j.envpol.2021.118339
$^{207}Pb/^{206}Pb$	Pb	Ore-related Emissions	0.86866	48.57	7.75	January, 2008	Active sampling	TIMS	Standard Calibration Method	0.0004	https://doi.org/10.1021/es071704c

$^{207}Pb/^{206}Pb$	Pb	Ore-related Emissions	0.86790	48.57	7.75	January, 2008	Active sampling	TIMS	Standard Calibration Method	0.0004	https://doi.org/10.1021/es071704c
$^{207}Pb/^{206}Pb$	Pb	Ore-related Emissions	0.86745	48.57	7.75	January, 2008	Active sampling	TIMS	Standard Calibration Method	0.0004	https://doi.org/10.1021/es071704c
$^{207}Pb/^{206}Pb$	Pb	Waste Incinerator	0.86408	49.12	-6.17	January and July, 2003	Active sampling	ICP-MS	Standard Calibration Method	0.20%	doi.org/10.1021/es0609654
$^{207}Pb/^{206}Pb$	Pb	Waste Incinerator	0.86558	49.12	-6.17	January and July, 2003	Active sampling	ICP-MS	Standard Calibration Method	0.20%	doi.org/10.1021/es0609654
$^{207}Pb/^{206}Pb$	Pb	Waste Incinerator	0.86693	49.12	-6.17	January and July, 2003	Active sampling	ICP-MS	Standard Calibration Method	0.20%	doi.org/10.1021/es0609654
$^{207}Pb/^{206}Pb$	Pb	Waste Incinerator	0.86783	48.57	7.75	January, 2008	Active sampling	TIMS	Standard Calibration Method	0.0004	https://doi.org/10.1021/es071704c
$^{207}Pb/^{206}Pb$	Pb	Waste Incinerator	0.87390	48.57	7.75	January, 2008	Active sampling	TIMS	Standard Calibration Method	0.0004	https://doi.org/10.1021/es071704c
$^{207}Pb/^{206}Pb$	Pb	Waste Incinerator	0.87298	48.57	7.75	January, 2008	Active sampling	TIMS	Standard Calibration Method	0.0004	https://doi.org/10.1021/es071704c
$^{207}Pb/^{206}Pb$	Pb	Waste Incinerator	0.87055	48.57	7.75	January, 2008	Active sampling	TIMS	Standard Calibration Method	0.0004	https://doi.org/10.1021/es071704c
$^{207}Pb/^{206}Pb$	Pb	Waste Incinerator	0.87017	48.57	7.75	January, 2008	Active sampling	TIMS	Standard Calibration Method	0.0004	https://doi.org/10.1021/es071704c
$^{207}Pb/^{206}Pb$	Pb	Waste Incinerator	0.86904	48.57	7.75	January, 2008	Active sampling	TIMS	Standard Calibration Method	0.0004	https://doi.org/10.1021/es071704c
$^{207}Pb/^{206}Pb$	Pb	Waste Incinerator	0.87283	48.57	7.75	January, 2008	Active sampling	TIMS	Standard Calibration Method	0.0004	https://doi.org/10.1021/es071704c
$^{207}Pb/^{206}Pb$	Pb	Waste Incinerator	0.87413	48.57	7.75	January, 2008	Active sampling	TIMS	Standard Calibration Method	0.0004	https://doi.org/10.1021/es071704c
$^{207}Pb/^{206}Pb$	Pb	Waste Incinerator	0.86919	48.57	7.75	January, 2008	Active sampling	TIMS	Standard Calibration Method	0.0004	https://doi.org/10.1021/es071704c
$^{207}Pb/^{206}Pb$	Pb	Waste Incinerator	0.86281	39.11	117.16	31 July, 2019	Active sampling	MC-ICP-MS	Standard Calibration Method	0.0002	http://dx.doi.org/10.1016/j.scitotenv.2023.164567

$^{207}Pb/^{206}Pb$	Pb	Waste Incinerator	0.86336	39.11	117.16	31 July, 2019	Active sampling	MC-ICP-MS	Standard Calibration Method	0.0002	http://dx.doi.org/10.1016/j.scitotenv.2023.164567
$^{207}Pb/^{206}Pb$	Pb	Waste Incinerator	0.86434	39.11	117.16	31 July, 2019	Active sampling	MC-ICP-MS	Standard Calibration Method	0.0002	http://dx.doi.org/10.1016/j.scitotenv.2023.164567
$^{207}Pb/^{206}Pb$	Pb	Waste Incinerator	0.86417	39.11	117.16	31 July, 2019	Active sampling	MC-ICP-MS	Standard Calibration Method	0.0002	http://dx.doi.org/10.1016/j.scitotenv.2023.164567
$^{207}Pb/^{206}Pb$	Pb	Waste Incinerator	0.86306	39.11	117.16	31 July, 2019	Active sampling	MC-ICP-MS	Standard Calibration Method	0.0002	http://dx.doi.org/10.1016/j.scitotenv.2023.164567
$^{207}Pb/^{206}Pb$	Pb	Waste Incinerator	0.86512	39.11	117.16	31 July, 2019	Active sampling	MC-ICP-MS	Standard Calibration Method	0.0002	http://dx.doi.org/10.1016/j.scitotenv.2023.164567
$^{207}Pb/^{206}Pb$	Pb	Waste Incinerator	0.86813	39.11	117.16	31 July, 2019	Active sampling	MC-ICP-MS	Standard Calibration Method	0.0002	http://dx.doi.org/10.1016/j.scitotenv.2023.164567
$^{207}Pb/^{206}Pb$	Pb	Waste Incinerator	0.86412	39.11	117.16	31 July, 2019	Active sampling	MC-ICP-MS	Standard Calibration Method	0.0002	http://dx.doi.org/10.1016/j.scitotenv.2023.164567
$^{207}Pb/^{206}Pb$	Pb	Waste Incinerator	0.86537	39.11	117.16	31 July, 2019	Active sampling	MC-ICP-MS	Standard Calibration Method	0.0002	http://dx.doi.org/10.1016/j.scitotenv.2023.164567
$^{207}Pb/^{206}Pb$	Pb	Waste Incinerator	0.86549	39.11	117.16	31 July, 2019	Active sampling	MC-ICP-MS	Standard Calibration Method	0.0002	http://dx.doi.org/10.1016/j.scitotenv.2023.164567
$^{207}Pb/^{206}Pb$	Pb	Waste Incinerator	0.86525	39.11	117.16	31 July, 2019	Active sampling	MC-ICP-MS	Standard Calibration Method	0.0002	http://dx.doi.org/10.1016/j.scitotenv.2023.164567
$^{207}Pb/^{206}Pb$	Pb	Natural Soil	0.85602	31.96	119.70	8 July-14 October, 2008	Active sampling	ICP-MS	Standard Calibration Method	0.33%	https://doi.org/10.1016/S1001-0742(10)60529-3
$^{207}Pb/^{206}Pb$	Pb	Natural Soil	0.85481	31.96	119.70	8 July-14 October, 2008	Active sampling	ICP-MS	Standard Calibration Method	0.33%	https://doi.org/10.1016/S1001-0742(10)60529-3
$^{207}Pb/^{206}Pb$	Pb	Natural Soil	0.85371	31.96	119.70	8 July-14 October, 2008	Active sampling	ICP-MS	Standard Calibration Method	0.33%	https://doi.org/10.1016/S1001-0742(10)60529-3
$^{207}Pb/^{206}Pb$	Pb	Natural Soil	0.85322	31.96	119.70	8 July-14 October, 2008	Active sampling	ICP-MS	Standard Calibration Method	0.33%	https://doi.org/10.1016/S1001-0742(10)60529-3
$^{207}Pb/^{206}Pb$	Pb	Natural Soil	0.85121	31.96	119.70	8 July-14 October, 2008	Active sampling	ICP-MS	Standard Calibration Method	0.33%	https://doi.org/10.1016/S1001-0742(10)60529-3

$^{207}Pb/^{206}Pb$	Pb	Natural Soil	0.85091	31.96	119.70	8 July-14 October, 2008	Active sampling	ICP-MS	Standard Calibration Method	0.33%	https://doi.org/10.1016/S1001-0742(10)60529-3
$^{207}Pb/^{206}Pb$	Pb	Natural Soil	0.85911	51.75	4.77	July, 2003	Active sampling	ICP-MS	Standard Calibration Method	0.0012	10.1016/j.apgeochem.2008.01.010
$^{207}Pb/^{206}Pb$	Pb	Natural Soil	0.85911	51.75	4.77	July, 2003	Active sampling	ICP-MS	Standard Calibration Method	0.0012	10.1016/j.apgeochem.2008.01.010
$^{207}Pb/^{206}Pb$	Pb	Natural Soil	0.85985	51.75	4.77	July, 2003	Active sampling	ICP-MS	Standard Calibration Method	0.0012	10.1016/j.apgeochem.2008.01.010
$^{207}Pb/^{206}Pb$	Pb	Natural Soil	0.85985	51.75	4.77	July, 2003	Active sampling	ICP-MS	Standard Calibration Method	0.0012	10.1016/j.apgeochem.2008.01.010
$^{207}Pb/^{206}Pb$	Pb	Natural Soil	0.85985	51.75	4.77	July, 2003	Active sampling	ICP-MS	Standard Calibration Method	0.0012	10.1016/j.apgeochem.2008.01.010
$^{207}Pb/^{206}Pb$	Pb	Natural Soil	0.85837	51.80	5.43	July, 2003	Active sampling	ICP-MS	Standard Calibration Method	0.0012	10.1016/j.apgeochem.2008.01.010
$^{207}Pb/^{206}Pb$	Pb	Natural Soil	0.85911	51.80	5.43	July, 2003	Active sampling	ICP-MS	Standard Calibration Method	0.0012	10.1016/j.apgeochem.2008.01.010
$^{207}Pb/^{206}Pb$	Pb	Natural Soil	0.85911	51.80	5.43	July, 2003	Active sampling	ICP-MS	Standard Calibration Method	0.0012	10.1016/j.apgeochem.2008.01.010
$^{207}Pb/^{206}Pb$	Pb	Natural Soil	0.85763	51.80	5.43	July, 2003	Active sampling	ICP-MS	Standard Calibration Method	0.0012	10.1016/j.apgeochem.2008.01.010
$^{207}Pb/^{206}Pb$	Pb	Natural Soil	0.85837	51.80	5.43	July, 2003	Active sampling	ICP-MS	Standard Calibration Method	0.0012	10.1016/j.apgeochem.2008.01.010
$^{207}Pb/^{206}Pb$	Pb	Natural Soil	0.84962	51.80	5.43	July, 2003	Active sampling	ICP-MS	Standard Calibration Method	0.0012	10.1016/j.apgeochem.2008.01.010
$^{207}Pb/^{206}Pb$	Pb	Natural Soil	0.84962	51.80	5.43	July, 2003	Active sampling	ICP-MS	Standard Calibration Method	0.0012	10.1016/j.apgeochem.2008.01.010
$^{207}Pb/^{206}Pb$	Pb	Natural Soil	0.85034	51.80	5.43	July, 2003	Active sampling	ICP-MS	Standard Calibration Method	0.0012	10.1016/j.apgeochem.2008.01.010
$^{207}Pb/^{206}Pb$	Pb	Natural Soil	0.85106	51.80	5.43	July, 2003	Active sampling	ICP-MS	Standard Calibration Method	0.0012	10.1016/j.apgeochem.2008.01.010

$^{207}Pb/^{206}Pb$	Pb	Natural Soil	0.85106	51.80	5.43	July, 2003	Active sampling	ICP-MS	Standard Calibration Method	0.0012	10.1016/j.apgeochem.2008.01.010
$^{207}Pb/^{206}Pb$	Pb	Natural Soil	0.83373	27.00	104.27	January, 2008	Active sampling	ICP-MS	Standard Calibration Method	0.0011	10.1016/j.envpol.2008.11.013
$^{207}Pb/^{206}Pb$	Pb	Natural Soil	0.80757	27.00	104.27	January, 2008	Active sampling	ICP-MS	Standard Calibration Method	0.0011	10.1016/j.envpol.2008.11.013
$^{207}Pb/^{206}Pb$	Pb	Natural Soil	0.84077	27.00	104.27	January, 2008	Active sampling	ICP-MS	Standard Calibration Method	0.0011	10.1016/j.envpol.2008.11.013
$^{207}Pb/^{206}Pb$	Pb	Natural Soil	0.83972	27.00	104.27	January, 2008	Active sampling	ICP-MS	Standard Calibration Method	0.0011	10.1016/j.envpol.2008.11.013
$^{207}Pb/^{206}Pb$	Pb	Natural Soil	0.83885	27.00	104.27	January, 2008	Active sampling	ICP-MS	Standard Calibration Method	0.0011	10.1016/j.envpol.2008.11.013
$^{207}Pb/^{206}Pb$	Pb	Natural Soil	0.83400	39.91	116.39	January, 2010	Active sampling	MC-ICP-MS	Standard Calibration Method	0.0002	10.1016/j.atmosenv.2010.06.036
$^{207}Pb/^{206}Pb$	Pb	Natural Soil	0.83890	39.91	116.39	January, 2010	Active sampling	MC-ICP-MS	Standard Calibration Method	0.0002	10.1016/j.atmosenv.2010.06.036
$^{207}Pb/^{206}Pb$	Pb	Natural Soil	0.84660	43.63	111.98	January, 2010	Active sampling	MC-ICP-MS	Standard Calibration Method	0.0002	10.1016/j.atmosenv.2010.06.036
$^{207}Pb/^{206}Pb$	Pb	Natural Soil	0.85260	41.52	111.70	January, 2010	Active sampling	MC-ICP-MS	Standard Calibration Method	0.0002	10.1016/j.atmosenv.2010.06.036
$^{207}Pb/^{206}Pb$	Pb	Natural Soil	0.87740	40.73	107.38	January, 2010	Active sampling	MC-ICP-MS	Standard Calibration Method	0.0002	10.1016/j.atmosenv.2010.06.036
$^{207}Pb/^{206}Pb$	Pb	Natural Soil	0.86200	31.22	121.47	2002	Active sampling	ICP-MS	Standard Calibration Method	0.07%	10.1016/j.atmosenv.2004.10.041
$^{207}Pb/^{206}Pb$	Pb	Natural Soil	0.84100	31.22	121.47	January, 2006	Active sampling	ICP-MS	Standard Calibration Method	0.0002	doi.org/10.1021/ac061365q
$^{207}Pb/^{206}Pb$	Pb	Natural Soil	0.84750	48.65	7.75	January, 2012	Active sampling	MC-ICP-MS	Standard Calibration Method	0.0001	http://dx.doi.org/10.1016/j.atmosenv.2012.08.044
$^{207}Pb/^{206}Pb$	Pb	Natural Soil	0.80580	21.07	107.32	January, 2015	Active sampling	-	Standard Calibration Method	0.0002	dx.doi.org/10.1016/j.crte.2015.02.007

$^{207}Pb/^{206}Pb$	Pb	Natural Soil	0.80645	21.07	107.32	January, 2015	Active sampling	-	Standard Calibration Method	0.0002	dx.doi.org/10.1016/j.crte.2015.02.007
$^{207}Pb/^{206}Pb$	Pb	Natural Soil	0.68306	21.07	107.32	January, 2015	Active sampling	-	Standard Calibration Method	0.0002	dx.doi.org/10.1016/j.crte.2015.02.007
$^{207}Pb/^{206}Pb$	Pb	Natural Soil	0.83550	21.07	107.32	January, 2015	Active sampling	-	Standard Calibration Method	0.0002	dx.doi.org/10.1016/j.crte.2015.02.007
$^{207}Pb/^{206}Pb$	Pb	Natural Soil	0.84818	26.57	101.72	15-25 May, 2018	Active sampling	MC-ICP-MS	Standard Calibration Method	0.0002	doi.org/10.1016/j.apr.2019.10.014
$^{207}Pb/^{206}Pb$	Pb	Natural Soil	0.84766	26.57	101.72	15-25 May, 2018	Active sampling	MC-ICP-MS	Standard Calibration Method	0.0002	doi.org/10.1016/j.apr.2019.10.014
$^{207}Pb/^{206}Pb$	Pb	Natural Soil	0.84895	26.57	101.72	15-25 May, 2018	Active sampling	MC-ICP-MS	Standard Calibration Method	0.0002	doi.org/10.1016/j.apr.2019.10.014
$^{207}Pb/^{206}Pb$	Pb	Natural Soil	0.85117	26.57	101.72	15-25 May, 2018	Active sampling	MC-ICP-MS	Standard Calibration Method	0.0002	doi.org/10.1016/j.apr.2019.10.014
$^{207}Pb/^{206}Pb$	Pb	Natural Soil	0.85131	26.57	101.72	15-25 May, 2018	Active sampling	MC-ICP-MS	Standard Calibration Method	0.0002	doi.org/10.1016/j.apr.2019.10.014
$^{207}Pb/^{206}Pb$	Pb	Natural Soil	0.85180	26.57	101.72	15-25 May, 2018	Active sampling	MC-ICP-MS	Standard Calibration Method	0.0002	doi.org/10.1016/j.apr.2019.10.014
$^{207}Pb/^{206}Pb$	Pb	Natural Soil	0.85221	26.57	101.72	15-25 May, 2018	Active sampling	MC-ICP-MS	Standard Calibration Method	0.0002	doi.org/10.1016/j.apr.2019.10.014
$^{207}Pb/^{206}Pb$	Pb	Natural Soil	0.85559	26.57	101.72	15-25 May, 2018	Active sampling	MC-ICP-MS	Standard Calibration Method	0.0002	doi.org/10.1016/j.apr.2019.10.014
$^{207}Pb/^{206}Pb$	Pb	Natural Soil	0.85502	26.57	101.72	15-25 May, 2018	Active sampling	MC-ICP-MS	Standard Calibration Method	0.0002	doi.org/10.1016/j.apr.2019.10.014
$^{207}Pb/^{206}Pb$	Pb	Natural Soil	0.85352	26.57	101.72	15-25 May, 2018	Active sampling	MC-ICP-MS	Standard Calibration Method	0.0002	doi.org/10.1016/j.apr.2019.10.014
$^{207}Pb/^{206}Pb$	Pb	Natural Soil	0.85357	26.57	101.72	15-25 May, 2018	Active sampling	MC-ICP-MS	Standard Calibration Method	0.0002	doi.org/10.1016/j.apr.2019.10.014
$^{207}Pb/^{206}Pb$	Pb	Natural Soil	0.85465	26.57	101.72	15-25 May, 2018	Active sampling	MC-ICP-MS	Standard Calibration Method	0.0002	doi.org/10.1016/j.apr.2019.10.014

$^{207}Pb/^{206}Pb$	Pb	Natural Soil	0.85450	26.57	101.72	15-25 May, 2018	Active sampling	MC-ICP-MS	Standard Calibration Method	0.0002	doi.org/10.1016/j.apr.2019.10.014
$^{207}Pb/^{206}Pb$	Pb	Natural Soil	0.85232	26.57	101.72	15-25 May, 2018	Active sampling	MC-ICP-MS	Standard Calibration Method	0.0002	doi.org/10.1016/j.apr.2019.10.014
$^{207}Pb/^{206}Pb$	Pb	Natural Soil	0.85264	26.57	101.72	15-25 May, 2018	Active sampling	MC-ICP-MS	Standard Calibration Method	0.0002	doi.org/10.1016/j.apr.2019.10.014
$^{207}Pb/^{206}Pb$	Pb	Natural Soil	0.85181	26.57	101.72	15-25 May, 2018	Active sampling	MC-ICP-MS	Standard Calibration Method	0.0002	doi.org/10.1016/j.apr.2019.10.014
$^{207}Pb/^{206}Pb$	Pb	Natural Soil	0.85313	26.57	101.72	15-25 May, 2018	Active sampling	MC-ICP-MS	Standard Calibration Method	0.0002	doi.org/10.1016/j.apr.2019.10.014
$^{207}Pb/^{206}Pb$	Pb	Natural Soil	0.84972	26.57	101.72	15-25 May, 2018	Active sampling	MC-ICP-MS	Standard Calibration Method	0.0002	doi.org/10.1016/j.apr.2019.10.014
$^{207}Pb/^{206}Pb$	Pb	Natural Soil	0.84983	26.57	101.72	15-25 May, 2018	Active sampling	MC-ICP-MS	Standard Calibration Method	0.0002	doi.org/10.1016/j.apr.2019.10.014
$^{207}Pb/^{206}Pb$	Pb	Natural Soil	0.85149	26.57	101.72	15-25 May, 2018	Active sampling	MC-ICP-MS	Standard Calibration Method	0.0002	doi.org/10.1016/j.apr.2019.10.014
$^{207}Pb/^{206}Pb$	Pb	Natural Soil	0.82711	26.57	101.72	January, 2019	Active sampling	MC-ICP-MS	Standard Calibration Method	0.0002	doi.org/10.1016/j.apr.2019.10.014
$^{207}Pb/^{206}Pb$	Pb	Natural Soil	0.83484	26.57	101.72	January, 2019	Active sampling	MC-ICP-MS	Standard Calibration Method	0.0002	doi.org/10.1016/j.apr.2019.10.014
$^{207}Pb/^{206}Pb$	Pb	Natural Soil	0.84641	26.57	101.72	January, 2019	Active sampling	MC-ICP-MS	Standard Calibration Method	0.0002	doi.org/10.1016/j.apr.2019.10.014
$^{207}Pb/^{206}Pb$	Pb	Natural Soil	0.83486	38.03	114.50	January, 2019	Active sampling	ICP-MS	Standard Calibration Method	0.00004	doi.org/10.3390/atmos10040222
$^{207}Pb/^{206}Pb$	Pb	Natural Soil	0.85281	38.03	114.50	January, 2019	Active sampling	ICP-MS	Standard Calibration Method	0.00004	doi.org/10.3390/atmos10040222
$^{207}Pb/^{206}Pb$	Pb	Natural Soil	0.85390	38.03	114.50	January, 2019	Active sampling	ICP-MS	Standard Calibration Method	0.00004	doi.org/10.3390/atmos10040222
$^{207}Pb/^{206}Pb$	Pb	Natural Soil	0.84886	32.05	118.78	July, 2013	Active sampling	Q-ICP-MS	Standard Calibration Method	0.0002	doi.org/10.1016/j.envpol.2013.12.025

$^{207}Pb/^{206}Pb$	Pb	Natural Soil	0.85591	32.05	118.78	July, 2013	Active sampling	Q-ICP-MS	Standard Calibration Method	0.0002	doi.org/10.1016/j.envpol.2013.12.025
$^{207}Pb/^{206}Pb$	Pb	Natural Soil	0.84763	32.05	118.78	July, 2013	Active sampling	Q-ICP-MS	Standard Calibration Method	0.0002	doi.org/10.1016/j.envpol.2013.12.025
$^{207}Pb/^{206}Pb$	Pb	Natural Soil	0.84728	32.05	118.78	July, 2013	Active sampling	Q-ICP-MS	Standard Calibration Method	0.0002	doi.org/10.1016/j.envpol.2013.12.025
$^{207}Pb/^{206}Pb$	Pb	Natural Soil	0.84360	32.05	118.78	July, 2013	Active sampling	Q-ICP-MS	Standard Calibration Method	0.0002	doi.org/10.1016/j.envpol.2013.12.025
$^{207}Pb/^{206}Pb$	Pb	Natural Soil	0.85020	32.05	118.78	July, 2013	Active sampling	Q-ICP-MS	Standard Calibration Method	0.0002	doi.org/10.1016/j.envpol.2013.12.025
$^{207}Pb/^{206}Pb$	Pb	Natural Soil	0.84860	32.05	118.78	July, 2013	Active sampling	Q-ICP-MS	Standard Calibration Method	0.0002	doi.org/10.1016/j.envpol.2013.12.025
$^{207}Pb/^{206}Pb$	Pb	Natural Soil	0.84923	32.05	118.78	July, 2013	Active sampling	Q-ICP-MS	Standard Calibration Method	0.0002	doi.org/10.1016/j.envpol.2013.12.025
$^{207}Pb/^{206}Pb$	Pb	Natural Soil	0.85033	32.05	118.78	July, 2013	Active sampling	Q-ICP-MS	Standard Calibration Method	0.0002	doi.org/10.1016/j.envpol.2013.12.025
$^{207}Pb/^{206}Pb$	Pb	Natural Soil	0.85547	32.05	118.78	July, 2013	Active sampling	Q-ICP-MS	Standard Calibration Method	0.0002	doi.org/10.1016/j.envpol.2013.12.025
$^{207}Pb/^{206}Pb$	Pb	Natural Soil	0.83727	32.05	118.78	July, 2013	Active sampling	Q-ICP-MS	Standard Calibration Method	0.0002	doi.org/10.1016/j.envpol.2013.12.025
$^{207}Pb/^{206}Pb$	Pb	Natural Soil	0.83856	32.05	118.78	July, 2013	Active sampling	Q-ICP-MS	Standard Calibration Method	0.0002	doi.org/10.1016/j.envpol.2013.12.025
$^{207}Pb/^{206}Pb$	Pb	Natural Soil	0.83891	32.05	118.78	July, 2013	Active sampling	Q-ICP-MS	Standard Calibration Method	0.0002	doi.org/10.1016/j.envpol.2013.12.025
$^{207}Pb/^{206}Pb$	Pb	Natural Soil	0.84073	32.05	118.78	July, 2013	Active sampling	Q-ICP-MS	Standard Calibration Method	0.0002	doi.org/10.1016/j.envpol.2013.12.025
$^{207}Pb/^{206}Pb$	Pb	Natural Soil	0.84151	32.05	118.78	July, 2013	Active sampling	Q-ICP-MS	Standard Calibration Method	0.0002	doi.org/10.1016/j.envpol.2013.12.025
$^{207}Pb/^{206}Pb$	Pb	Natural Soil	0.84098	32.05	118.78	July, 2013	Active sampling	Q-ICP-MS	Standard Calibration Method	0.0002	doi.org/10.1016/j.envpol.2013.12.025

$^{207}Pb/^{206}Pb$	Pb	Natural Soil	0.84093	32.05	118.78	July, 2013	Active sampling	Q-ICP-MS	Standard Calibration Method	0.0002	doi.org/10.1016/j.envpol.2013.12.025
$^{207}Pb/^{206}Pb$	Pb	Natural Soil	0.84032	32.05	118.78	July, 2013	Active sampling	Q-ICP-MS	Standard Calibration Method	0.0002	doi.org/10.1016/j.envpol.2013.12.025
$^{207}Pb/^{206}Pb$	Pb	Natural Soil	0.84246	32.05	118.78	July, 2013	Active sampling	Q-ICP-MS	Standard Calibration Method	0.0002	doi.org/10.1016/j.envpol.2013.12.025
$^{207}Pb/^{206}Pb$	Pb	Natural Soil	0.84246	32.05	118.78	July, 2013	Active sampling	Q-ICP-MS	Standard Calibration Method	0.0002	doi.org/10.1016/j.envpol.2013.12.025
$^{207}Pb/^{206}Pb$	Pb	Natural Soil	0.84317	32.05	118.78	July, 2013	Active sampling	Q-ICP-MS	Standard Calibration Method	0.0002	doi.org/10.1016/j.envpol.2013.12.025
$^{207}Pb/^{206}Pb$	Pb	Natural Soil	0.84281	32.05	118.78	July, 2013	Active sampling	Q-ICP-MS	Standard Calibration Method	0.0002	doi.org/10.1016/j.envpol.2013.12.025
$^{207}Pb/^{206}Pb$	Pb	Natural Soil	0.84263	32.05	118.78	July, 2013	Active sampling	Q-ICP-MS	Standard Calibration Method	0.0002	doi.org/10.1016/j.envpol.2013.12.025
$^{207}Pb/^{206}Pb$	Pb	Natural Soil	0.84298	32.05	118.78	July, 2013	Active sampling	Q-ICP-MS	Standard Calibration Method	0.0002	doi.org/10.1016/j.envpol.2013.12.025
$^{207}Pb/^{206}Pb$	Pb	Natural Soil	0.84201	32.05	118.78	July, 2013	Active sampling	Q-ICP-MS	Standard Calibration Method	0.0002	doi.org/10.1016/j.envpol.2013.12.025
$^{207}Pb/^{206}Pb$	Pb	Natural Soil	0.84328	32.05	118.78	July, 2013	Active sampling	Q-ICP-MS	Standard Calibration Method	0.0002	doi.org/10.1016/j.envpol.2013.12.025
$^{207}Pb/^{206}Pb$	Pb	Natural Soil	0.84447	32.05	118.78	July, 2013	Active sampling	Q-ICP-MS	Standard Calibration Method	0.0002	doi.org/10.1016/j.envpol.2013.12.025
$^{207}Pb/^{206}Pb$	Pb	Natural Soil	0.84433	32.05	118.78	July, 2013	Active sampling	Q-ICP-MS	Standard Calibration Method	0.0002	doi.org/10.1016/j.envpol.2013.12.025
$^{207}Pb/^{206}Pb$	Pb	Natural Soil	0.84446	32.05	118.78	July, 2013	Active sampling	Q-ICP-MS	Standard Calibration Method	0.0002	doi.org/10.1016/j.envpol.2013.12.025
$^{207}Pb/^{206}Pb$	Pb	Natural Soil	0.84547	32.05	118.78	July, 2013	Active sampling	Q-ICP-MS	Standard Calibration Method	0.0002	doi.org/10.1016/j.envpol.2013.12.025
$^{207}Pb/^{206}Pb$	Pb	Natural Soil	0.84538	32.05	118.78	July, 2013	Active sampling	Q-ICP-MS	Standard Calibration Method	0.0002	doi.org/10.1016/j.envpol.2013.12.025

$^{207}Pb/^{206}Pb$	Pb	Natural Soil	0.84666	32.05	118.78	July, 2013	Active sampling	Q-ICP-MS	Standard Calibration Method	0.0002	doi.org/10.1016/j.envpol.2013.12.025
$^{207}Pb/^{206}Pb$	Pb	Natural Soil	0.84587	32.05	118.78	July, 2013	Active sampling	Q-ICP-MS	Standard Calibration Method	0.0002	doi.org/10.1016/j.envpol.2013.12.025
$^{207}Pb/^{206}Pb$	Pb	Natural Soil	0.84649	32.05	118.78	July, 2013	Active sampling	Q-ICP-MS	Standard Calibration Method	0.0002	doi.org/10.1016/j.envpol.2013.12.025
$^{207}Pb/^{206}Pb$	Pb	Natural Soil	0.84587	32.05	118.78	July, 2013	Active sampling	Q-ICP-MS	Standard Calibration Method	0.0002	doi.org/10.1016/j.envpol.2013.12.025
$^{207}Pb/^{206}Pb$	Pb	Natural Soil	0.84957	32.05	118.78	July, 2013	Active sampling	Q-ICP-MS	Standard Calibration Method	0.0002	doi.org/10.1016/j.envpol.2013.12.025
$^{207}Pb/^{206}Pb$	Pb	Natural Soil	0.85019	32.05	118.78	July, 2013	Active sampling	Q-ICP-MS	Standard Calibration Method	0.0002	doi.org/10.1016/j.envpol.2013.12.025
$^{207}Pb/^{206}Pb$	Pb	Natural Soil	0.85126	32.05	118.78	July, 2013	Active sampling	Q-ICP-MS	Standard Calibration Method	0.0002	doi.org/10.1016/j.envpol.2013.12.025
$^{207}Pb/^{206}Pb$	Pb	Natural Soil	0.85171	32.05	118.78	July, 2013	Active sampling	Q-ICP-MS	Standard Calibration Method	0.0002	doi.org/10.1016/j.envpol.2013.12.025
$^{207}Pb/^{206}Pb$	Pb	Natural Soil	0.85318	32.05	118.78	July, 2013	Active sampling	Q-ICP-MS	Standard Calibration Method	0.0002	doi.org/10.1016/j.envpol.2013.12.025
$^{207}Pb/^{206}Pb$	Pb	Natural Soil	0.85270	32.05	118.78	July, 2013	Active sampling	Q-ICP-MS	Standard Calibration Method	0.0002	doi.org/10.1016/j.envpol.2013.12.025
$^{207}Pb/^{206}Pb$	Pb	Natural Soil	0.884490175	32.05	118.78	July, 2013	Active sampling	Q-ICP-MS	Standard Calibration Method	0.0002	doi.org/10.1016/j.envpol.2013.12.025
$^{207}Pb/^{206}Pb$	Pb	Natural Soil	0.88299221	32.05	118.78	July, 2013	Active sampling	Q-ICP-MS	Standard Calibration Method	0.0002	doi.org/10.1016/j.envpol.2013.12.025
$^{207}Pb/^{206}Pb$	Pb	Natural Soil	0.885638298	32.05	118.78	July, 2013	Active sampling	Q-ICP-MS	Standard Calibration Method	0.0002	doi.org/10.1016/j.envpol.2013.12.025
$^{207}Pb/^{206}Pb$	Pb	Natural Soil	0.885782978	32.05	118.78	July, 2013	Active sampling	Q-ICP-MS	Standard Calibration Method	0.0002	doi.org/10.1016/j.envpol.2013.12.025
$^{207}Pb/^{206}Pb$	Pb	Natural Soil	0.886460488	32.05	118.78	July, 2013	Active sampling	Q-ICP-MS	Standard Calibration Method	0.0002	doi.org/10.1016/j.envpol.2013.12.025

$^{207}Pb/^{206}Pb$	Pb	Natural Soil	0.8868929	32.05	118.78	July, 2013	Active sampling	Q-ICP-MS	Standard Calibration Method	0.0002	doi.org/10.1016/j.envpol.2013.12.025
$^{207}Pb/^{206}Pb$	Pb	Natural Soil	0.887280054	32.05	118.78	July, 2013	Active sampling	Q-ICP-MS	Standard Calibration Method	0.0002	doi.org/10.1016/j.envpol.2013.12.025
$^{207}Pb/^{206}Pb$	Pb	Natural Soil	0.888977637	32.05	118.78	July, 2013	Active sampling	Q-ICP-MS	Standard Calibration Method	0.0002	doi.org/10.1016/j.envpol.2013.12.025
$^{207}Pb/^{206}Pb$	Pb	Natural Soil	0.84739	23.82	120.28	January, 2021	Active sampling	MC-ICP-MS	Standard Calibration Method	0.0002	doi.org/10.1038/s41598-021-87051-y
$^{207}Pb/^{206}Pb$	Pb	Natural Soil	0.84911	23.82	120.28	January, 2021	Active sampling	MC-ICP-MS	Standard Calibration Method	0.0002	doi.org/10.1038/s41598-021-87051-y
$^{207}Pb/^{206}Pb$	Pb	Natural Soil	0.85056	23.82	120.28	January, 2021	Active sampling	MC-ICP-MS	Standard Calibration Method	0.0002	doi.org/10.1038/s41598-021-87051-y
$^{207}Pb/^{206}Pb$	Pb	Natural Soil	0.84524	23.82	120.28	January, 2021	Active sampling	MC-ICP-MS	Standard Calibration Method	0.0002	doi.org/10.1038/s41598-021-87051-y
$^{207}Pb/^{206}Pb$	Pb	Natural Soil	0.84983	23.82	120.28	January, 2021	Active sampling	MC-ICP-MS	Standard Calibration Method	0.0002	doi.org/10.1038/s41598-021-87051-y
$^{207}Pb/^{206}Pb$	Pb	Natural Soil	0.85157	23.82	120.28	January, 2021	Active sampling	MC-ICP-MS	Standard Calibration Method	0.0002	doi.org/10.1038/s41598-021-87051-y
$^{207}Pb/^{206}Pb$	Pb	Natural Soil	0.85135	23.82	120.28	January, 2021	Active sampling	MC-ICP-MS	Standard Calibration Method	0.0002	doi.org/10.1038/s41598-021-87051-y
$^{207}Pb/^{206}Pb$	Pb	Natural Soil	0.84696	23.82	120.28	January, 2021	Active sampling	MC-ICP-MS	Standard Calibration Method	0.0002	doi.org/10.1038/s41598-021-87051-y
$^{207}Pb/^{206}Pb$	Pb	Natural Soil	0.84818	23.82	120.28	January, 2021	Active sampling	MC-ICP-MS	Standard Calibration Method	0.0002	doi.org/10.1038/s41598-021-87051-y
$^{207}Pb/^{206}Pb$	Pb	Natural Soil	0.84760	23.82	120.28	January, 2021	Active sampling	MC-ICP-MS	Standard Calibration Method	0.0002	doi.org/10.1038/s41598-021-87051-y
$^{207}Pb/^{206}Pb$	Pb	Natural Soil	0.84282	23.82	120.28	January, 2021	Active sampling	MC-ICP-MS	Standard Calibration Method	0.0002	doi.org/10.1038/s41598-021-87051-y
$^{207}Pb/^{206}Pb$	Pb	Natural Soil	0.84175	23.82	120.28	January, 2021	Active sampling	MC-ICP-MS	Standard Calibration Method	0.0002	doi.org/10.1038/s41598-021-87051-y

$^{207}Pb/^{206}Pb$	Pb	Natural Soil	0.85070	23.82	120.28	January, 2021	Active sampling	MC-ICP-MS	Standard Calibration Method	0.0002	doi.org/10.1038/s41598-021-87051-y
$^{207}Pb/^{206}Pb$	Pb	Natural Soil	0.83963	24.47	118.08	January, 2018	Active sampling	ICP-MS	Standard Calibration Method	0.0002	doi.org/10.1016/j.atmosenv.2018.10.056
$^{207}Pb/^{206}Pb$	Pb	Natural Soil	0.82645	24.47	118.08	January, 2018	Active sampling	ICP-MS	Standard Calibration Method	0.0002	doi.org/10.1016/j.atmosenv.2018.10.056
$^{207}Pb/^{206}Pb$	Pb	Natural Soil	0.83339	24.47	118.08	January, 2018	Active sampling	ICP-MS	Standard Calibration Method	0.0002	doi.org/10.1016/j.atmosenv.2018.10.056
$^{207}Pb/^{206}Pb$	Pb	Natural Soil	0.82372	24.47	118.08	January, 2018	Active sampling	ICP-MS	Standard Calibration Method	0.0002	doi.org/10.1016/j.atmosenv.2018.10.056
$^{207}Pb/^{206}Pb$	Pb	Natural Soil	0.83270	24.47	118.08	January, 2018	Active sampling	ICP-MS	Standard Calibration Method	0.0002	doi.org/10.1016/j.atmosenv.2018.10.056
$^{207}Pb/^{206}Pb$	Pb	Natural Soil	0.91819	36.20	113.12	2017-2018	Active sampling	MC-ICP-MS	Standard Calibration Method	0.02%	https://doi.org/10.1016/j.atmosenv.2022.119503
$^{207}Pb/^{206}Pb$	Pb	Natural Soil	0.83612	24.47	118.08	January, 2018	Active sampling	ICP-MS	Standard Calibration Method	0.0002	doi.org/10.1016/j.atmosenv.2018.10.056
$^{207}Pb/^{206}Pb$	Pb	Natural Soil	0.83893	24.47	118.08	January, 2018	Active sampling	ICP-MS	Standard Calibration Method	0.0002	doi.org/10.1016/j.atmosenv.2018.10.056
$^{207}Pb/^{206}Pb$	Pb	Natural Soil	0.83822	24.47	118.08	January, 2018	Active sampling	ICP-MS	Standard Calibration Method	0.0002	doi.org/10.1016/j.atmosenv.2018.10.056
$^{207}Pb/^{206}Pb$	Pb	Natural Soil	0.84104	24.47	118.08	January, 2018	Active sampling	ICP-MS	Standard Calibration Method	0.0002	doi.org/10.1016/j.atmosenv.2018.10.056
$^{207}Pb/^{206}Pb$	Pb	Natural Soil	0.84034	24.47	118.08	January, 2018	Active sampling	ICP-MS	Standard Calibration Method	0.0002	doi.org/10.1016/j.atmosenv.2018.10.056
$^{207}Pb/^{206}Pb$	Pb	Natural Soil	0.85907	32.25	-110.79	January, 2014	Active sampling	ICP-MS	Standard Calibration Method	0.50%	https://doi.org/10.1016/j.chemosphere.2014.11.057
$^{207}Pb/^{206}Pb$	Pb	Natural Soil	0.85522	32.25	-110.79	January, 2014	Active sampling	ICP-MS	Standard Calibration Method	0.50%	https://doi.org/10.1016/j.chemosphere.2014.11.057
$^{207}Pb/^{206}Pb$	Pb	Natural Soil	0.85739	32.25	-110.79	January, 2014	Active sampling	ICP-MS	Standard Calibration Method	0.50%	https://doi.org/10.1016/j.chemosphere.2014.11.057

$^{207}Pb/^{206}Pb$	Pb	Natural Soil	0.85642	32.25	-110.79	January, 2014	Active sampling	ICP-MS	Standard Calibration Method	0.50%	https://doi.org/10.1016/j.chemosphere.2014.11.057
$^{207}Pb/^{206}Pb$	Pb	Natural Soil	0.85381	32.25	-110.79	January, 2014	Active sampling	ICP-MS	Standard Calibration Method	0.50%	https://doi.org/10.1016/j.chemosphere.2014.11.057
$^{207}Pb/^{206}Pb$	Pb	Natural Soil	0.83963	32.25	-110.79	January, 2014	Active sampling	ICP-MS	Standard Calibration Method	0.50%	https://doi.org/10.1016/j.chemosphere.2014.11.057
$^{207}Pb/^{206}Pb$	Pb	Natural Soil	0.79076	26.63	106.62	January, 2015	Active sampling	MC-ICP-MS	Standard Calibration Method	0.00012	dx.doi.org/10.1016/j.atmosenv.2015.05.049
$^{207}Pb/^{206}Pb$	Pb	Natural Soil	0.80128	26.63	106.62	January, 2015	Active sampling	MC-ICP-MS	Standard Calibration Method	0.00012	dx.doi.org/10.1016/j.atmosenv.2015.05.049
$^{207}Pb/^{206}Pb$	Pb	Natural Soil	0.78235	26.63	106.62	January, 2015	Active sampling	MC-ICP-MS	Standard Calibration Method	0.00012	dx.doi.org/10.1016/j.atmosenv.2015.05.049
$^{207}Pb/^{206}Pb$	Pb	Natural Soil	0.79158	26.63	106.62	January, 2015	Active sampling	MC-ICP-MS	Standard Calibration Method	0.00012	dx.doi.org/10.1016/j.atmosenv.2015.05.049
$^{207}Pb/^{206}Pb$	Pb	Natural Soil	0.82816	26.63	106.62	January, 2015	Active sampling	MC-ICP-MS	Standard Calibration Method	0.00012	dx.doi.org/10.1016/j.atmosenv.2015.05.049
$^{207}Pb/^{206}Pb$	Pb	Natural Soil	0.81513	26.63	106.62	January, 2015	Active sampling	MC-ICP-MS	Standard Calibration Method	0.00012	dx.doi.org/10.1016/j.atmosenv.2015.05.049
$^{207}Pb/^{206}Pb$	Pb	Natural Soil	0.80483	26.63	106.62	January, 2015	Active sampling	MC-ICP-MS	Standard Calibration Method	0.00012	dx.doi.org/10.1016/j.atmosenv.2015.05.049
$^{207}Pb/^{206}Pb$	Pb	Natural Soil	0.79139	26.63	106.62	January, 2015	Active sampling	MC-ICP-MS	Standard Calibration Method	0.00012	dx.doi.org/10.1016/j.atmosenv.2015.05.049
$^{207}Pb/^{206}Pb$	Pb	Natural Soil	0.79498	26.63	106.62	January, 2015	Active sampling	MC-ICP-MS	Standard Calibration Method	0.00012	dx.doi.org/10.1016/j.atmosenv.2015.05.049
$^{207}Pb/^{206}Pb$	Pb	Natural Soil	0.74755	26.63	106.62	January, 2015	Active sampling	MC-ICP-MS	Standard Calibration Method	0.00012	dx.doi.org/10.1016/j.atmosenv.2015.05.049
$^{207}Pb/^{206}Pb$	Pb	Natural Soil	0.72828	26.63	106.62	January, 2015	Active sampling	MC-ICP-MS	Standard Calibration Method	0.00012	dx.doi.org/10.1016/j.atmosenv.2015.05.049
$^{207}Pb/^{206}Pb$	Pb	Natural Soil	0.78765	26.63	106.62	January, 2015	Active sampling	MC-ICP-MS	Standard Calibration Method	0.00012	dx.doi.org/10.1016/j.atmosenv.2015.05.049

$^{207}Pb/^{206}Pb$	Pb	Natural Soil	0.78204	26.63	106.62	January, 2015	Active sampling	MC-ICP-MS	Standard Calibration Method	0.00012	dx.doi.org/10.1016/j.atmosenv.2015.05.049
$\delta^{144}Nd$	Nd	Coal Combustion	1.536‰	21.02	107.32	January, 2015	Active sampling	-	Standard Calibration Method	0.005%	dx.doi.org/10.1016/j.crte.2015.02.007
$\delta^{144}Nd$	Nd	Coal Combustion	1.551‰	21.02	107.32	January, 2015	Active sampling	-	Standard Calibration Method	0.005%	dx.doi.org/10.1016/j.crte.2015.02.007
$\delta^{144}Nd$	Nd	Coal Combustion	1.249‰	24.72	118.63	April, 2014	Active sampling	TIMS	Standard Calibration Method	0.005%	doi.org/10.4209/aaqr.2017.12.0559
$\delta^{144}Nd$	Nd	Coal Combustion	0.639‰	24.72	118.63	April, 2014	Active sampling	TIMS	Standard Calibration Method	0.005%	doi.org/10.4209/aaqr.2017.12.0559
$\delta^{144}Nd$	Nd	Coal Combustion	0.977‰	24.47	118.08	January, 2018	Active sampling	TIMS	Standard Calibration Method	0.005%	doi.org/10.1016/j.atmosenv.2018.10.056
$\delta^{144}Nd$	Nd	Coal Combustion	0.962‰	24.47	118.08	January, 2018	Active sampling	TIMS	Standard Calibration Method	0.005%	doi.org/10.1016/j.atmosenv.2018.10.056
$\delta^{144}Nd$	Nd	Coal Combustion	1.520‰	21.07	107.32	January, 2015	Active sampling	-	Standard Calibration Method	0.005%	dx.doi.org/10.1016/j.crte.2015.02.007
$\delta^{144}Nd$	Nd	Vehicle Exhausts	1.326‰	21.07	107.32	January, 2015	Active sampling	-	Standard Calibration Method	0.005%	dx.doi.org/10.1016/j.crte.2015.02.007
$\delta^{144}Nd$	Nd	Vehicle Exhausts	1.326‰	21.07	107.32	January, 2015	Active sampling	-	Standard Calibration Method	0.005%	dx.doi.org/10.1016/j.crte.2015.02.007
$\delta^{144}Nd$	Nd	Vehicle Exhausts	1.443‰	21.07	107.32	January, 2015	Active sampling	-	Standard Calibration Method	0.005%	dx.doi.org/10.1016/j.crte.2015.02.007
$\delta^{144}Nd$	Nd	Vehicle Exhausts	0.885‰	21.07	107.32	January, 2015	Active sampling	-	Standard Calibration Method	0.005%	dx.doi.org/10.1016/j.crte.2015.02.007
$\delta^{144}Nd$	Nd	Vehicle Exhausts	0.767‰	21.07	107.32	January, 2015	Active sampling	-	Standard Calibration Method	0.005%	dx.doi.org/10.1016/j.crte.2015.02.007
$\delta^{144}Nd$	Nd	Vehicle Exhausts	0.700‰	24.87	118.67	April, 2014	Active sampling	TIMS	Standard Calibration Method	0.005%	doi.org/10.4209/aaqr.2017.12.0559
$\delta^{144}Nd$	Nd	Vehicle Exhausts	0.700‰	24.47	118.08	January, 2018	Active sampling	TIMS	Standard Calibration Method	0.005%	doi.org/10.1016/j.atmosenv.2018.10.056

δ^{144}Nd	Nd	Vehicle Exhausts	0.603‰	24.47	118.08	January, 2018	Active sampling	TIMS	Standard Calibration Method	0.005%	doi.org/10.1016/j.atmosenv.2018.10.056
δ^{144}Nd	Nd	Vehicle Exhausts	-0.894‰	48.57	7.75	January, 2008	Active sampling	TIMS	Standard Calibration Method	0.005%	10.1016/j.apgeochem.2008.02.004
δ^{144}Nd	Nd	Vehicle Exhausts	-0.951‰	48.57	7.75	January, 2008	Active sampling	TIMS	Standard Calibration Method	0.005%	10.1016/j.apgeochem.2008.02.004
δ^{144}Nd	Nd	Vehicle Exhausts	0.603‰	48.57	7.75	January, 2008	Active sampling	MC-ICP-MS	Standard Calibration Method	0.005%	https://doi.org/10.1021/es071704c
δ^{144}Nd	Nd	Vehicle Exhausts	0.700‰	48.57	7.75	January, 2008	Active sampling	MC-ICP-MS	Standard Calibration Method	0.005%	https://doi.org/10.1021/es071704c
δ^{144}Nd	Nd	Natural Soil	-1.033‰	29.75	-95.28	January, 2022	Active sampling	MC-ICP-MS	Standard Calibration Method	0.005%	https://doi.org/10.1016/j.talanta.2022.123236
δ^{144}Nd	Nd	Natural Soil	-1.120‰	29.75	-95.28	January, 2022	Active sampling	MC-ICP-MS	Standard Calibration Method	0.005%	https://doi.org/10.1016/j.talanta.2022.123236
δ^{144}Nd	Nd	Natural Soil	-1.140‰	29.75	-95.28	January, 2022	Active sampling	MC-ICP-MS	Standard Calibration Method	0.005%	https://doi.org/10.1016/j.talanta.2022.123236
δ^{144}Nd	Nd	Natural Soil	-1.150‰	29.75	-95.28	January, 2022	Active sampling	MC-ICP-MS	Standard Calibration Method	0.005%	https://doi.org/10.1016/j.talanta.2022.123236
δ^{144}Nd	Nd	Non-exhaust emissions	1.541‰	28.67	115.85	January, 2015	Active sampling	TIMS	Standard Calibration Method	0.005%	doi.org/10.1016/j.atmosenv.2019.117069
δ^{144}Nd	Nd	Non-exhaust emissions	1.402‰	28.67	115.85	January, 2015	Active sampling	TIMS	Standard Calibration Method	0.005%	doi.org/10.1016/j.atmosenv.2019.117069
δ^{144}Nd	Nd	Non-exhaust emissions	1.628‰	28.67	115.85	January, 2015	Active sampling	TIMS	Standard Calibration Method	0.005%	doi.org/10.1016/j.atmosenv.2019.117069
δ^{144}Nd	Nd	Non-exhaust emissions	1.531‰	28.67	115.85	January, 2015	Active sampling	TIMS	Standard Calibration Method	0.005%	doi.org/10.1016/j.atmosenv.2019.117069
δ^{144}Nd	Nd	Non-exhaust emissions	1.490‰	28.67	115.85	January, 2015	Active sampling	TIMS	Standard Calibration Method	0.005%	doi.org/10.1016/j.atmosenv.2019.117069
δ^{144}Nd	Nd	Non-exhaust emissions	1.484‰	28.67	115.85	January, 2015	Active sampling	TIMS	Standard Calibration Method	0.005%	doi.org/10.1016/j.atmosenv.2019.117069

δ^{144}Nd	Nd	Non-exhaust emissions	1.367‰	28.67	115.85	January, 2015	Active sampling	TIMS	Standard Calibration Method	0.005%	doi.org/10.1016/j.atmosenv.2019.117069
δ^{144}Nd	Nd	Non-exhaust emissions	1.674‰	28.67	115.85	January, 2015	Active sampling	TIMS	Standard Calibration Method	0.005%	doi.org/10.1016/j.atmosenv.2019.117069
δ^{144}Nd	Nd	Non-exhaust emissions	1.495‰	28.67	115.85	January, 2015	Active sampling	TIMS	Standard Calibration Method	0.005%	doi.org/10.1016/j.atmosenv.2019.117069
δ^{144}Nd	Nd	Non-exhaust emissions	1.531‰	28.67	115.85	January, 2015	Active sampling	TIMS	Standard Calibration Method	0.005%	doi.org/10.1016/j.atmosenv.2019.117069
δ^{144}Nd	Nd	Non-exhaust emissions	1.433‰	28.67	115.85	January, 2015	Active sampling	TIMS	Standard Calibration Method	0.005%	doi.org/10.1016/j.atmosenv.2019.117069
δ^{144}Nd	Nd	Non-exhaust emissions	1.449‰	28.67	115.85	January, 2015	Active sampling	TIMS	Standard Calibration Method	0.005%	doi.org/10.1016/j.atmosenv.2019.117069
δ^{144}Nd	Nd	Non-exhaust emissions	1.346‰	28.67	115.85	January, 2015	Active sampling	TIMS	Standard Calibration Method	0.005%	doi.org/10.1016/j.atmosenv.2019.117069
δ^{144}Nd	Nd	Non-exhaust emissions	1.474‰	28.67	115.85	January, 2015	Active sampling	TIMS	Standard Calibration Method	0.005%	doi.org/10.1016/j.atmosenv.2019.117069
δ^{144}Nd	Nd	Non-exhaust emissions	1.490‰	28.67	115.85	January, 2015	Active sampling	TIMS	Standard Calibration Method	0.005%	doi.org/10.1016/j.atmosenv.2019.117069
δ^{144}Nd	Nd	Non-exhaust emissions	1.346‰	28.67	115.85	January, 2015	Active sampling	TIMS	Standard Calibration Method	0.005%	doi.org/10.1016/j.atmosenv.2019.117069
δ^{144}Nd	Nd	Non-exhaust emissions	0.926‰	29.73	-95.21	3 January-2 February, 2013	Active sampling	MC-ICP-MS	Standard Calibration Method	0.005%	https://doi.org/10.1016/j.talanta.2022.123236
δ^{144}Nd	Nd	Waste Incinerator	0.618‰	24.47	118.08	January, 2018	Active sampling	TIMS	Standard Calibration Method	0.005%	doi.org/10.1016/j.atmosenv.2018.10.056
δ^{144}Nd	Nd	Waste Incinerator	0.270‰	24.47	118.08	January, 2018	Active sampling	TIMS	Standard Calibration Method	0.005%	doi.org/10.1016/j.atmosenv.2018.10.056
δ^{144}Nd	Nd	Waste Incinerator	0.972‰	48.57	7.75	January, 2008	Active sampling	MC-ICP-MS	Standard Calibration Method	0.004%	https://doi.org/10.1021/es071704c
δ^{144}Nd	Nd	Waste Incinerator	0.992‰	48.57	7.75	January, 2008	Active sampling	MC-ICP-MS	Standard Calibration Method	0.004%	https://doi.org/10.1021/es071704c

$\delta^{144}Nd$	Nd	Waste Incinerator	0.972‰	48.57	7.75	January, 2008	Active sampling	MC-ICP-MS	Standard Calibration Method	0.004%	https://doi.org/10.1021/es071704c
$\delta^{144}Nd$	Nd	Waste Incinerator	0.931‰	48.57	7.75	January, 2008	Active sampling	MC-ICP-MS	Standard Calibration Method	0.004%	https://doi.org/10.1021/es071704c
$\delta^{144}Nd$	Nd	Waste Incinerator	0.972‰	48.57	7.75	January, 2008	Active sampling	MC-ICP-MS	Standard Calibration Method	0.004%	https://doi.org/10.1021/es071704c
$\delta^{144}Nd$	Nd	Waste Incinerator	0.874‰	48.57	7.75	January, 2008	Active sampling	MC-ICP-MS	Standard Calibration Method	0.004%	https://doi.org/10.1021/es071704c
$\delta^{144}Nd$	Nd	Waste Incinerator	0.874‰	48.57	7.75	January, 2008	Active sampling	MC-ICP-MS	Standard Calibration Method	0.004%	https://doi.org/10.1021/es071704c
$\delta^{144}Nd$	Nd	Waste Incinerator	0.931‰	48.57	7.75	January, 2008	Active sampling	MC-ICP-MS	Standard Calibration Method	0.004%	https://doi.org/10.1021/es071704c
$\delta^{144}Nd$	Nd	Waste Incinerator	1.249‰	48.57	7.75	January, 2008	Active sampling	MC-ICP-MS	Standard Calibration Method	0.004%	https://doi.org/10.1021/es071704c
$\delta^{144}Nd$	Nd	Waste Incinerator	1.033‰	48.57	7.75	January, 2008	Active sampling	MC-ICP-MS	Standard Calibration Method	0.004%	https://doi.org/10.1021/es071704c
$\delta^{65}Cu$	Cu	Biomass Burning	0.15‰	49.38	19.48	January, 2010	Active sampling	MC-ICP-MS	Sample-Standard Bracketing	0.04	10.1016/j.gca.2010.08.044
$\delta^{65}Cu$	Cu	Biomass Burning	0.15‰	49.38	19.48	January, 2010	Active sampling	MC-ICP-MS	Sample-Standard Bracketing	0.04	10.1016/j.gca.2010.08.044
$\delta^{65}Cu$	Cu	Biomass Burning	0.14‰	48.92	20.95	January, 2010	Active sampling	MC-ICP-MS	Sample-Standard Bracketing	0.04	10.1016/j.gca.2010.08.044
$\delta^{65}Cu$	Cu	Biomass Burning	-0.35‰	69.53	12.70	March, 2015	Active sampling	MC-ICP-MS	Sample-Standard Bracketing	0.05	dx.doi.org/10.1016/j.envpol.2017.05.030
$\delta^{65}Cu$	Cu	Biomass Burning	-0.24‰	69.53	12.70	March, 2015	Active sampling	MC-ICP-MS	Sample-Standard Bracketing	0.05	dx.doi.org/10.1016/j.envpol.2017.05.030
$\delta^{65}Cu$	Cu	Biomass Burning	-0.25‰	69.53	12.70	March, 2015	Active sampling	MC-ICP-MS	Sample-Standard Bracketing	0.05	dx.doi.org/10.1016/j.envpol.2017.05.030
$\delta^{65}Cu$	Cu	Biomass Burning	-0.06‰	69.53	12.70	March, 2015	Active sampling	MC-ICP-MS	Sample-Standard Bracketing	0.05	dx.doi.org/10.1016/j.envpol.2017.05.030

δ^{65}Cu	Cu	Biomass Burning	-0.20‰	38.68	115.25	January, 2021	Active sampling	MC-ICP-MS	Sample-Standard Bracketing	0.09	doi.org/10.1021/acs.est.1c05383
δ^{65}Cu	Cu	Biomass Burning	-0.74‰	38.68	115.25	January, 2021	Active sampling	MC-ICP-MS	Sample-Standard Bracketing	0.09	doi.org/10.1021/acs.est.1c05383
δ^{65}Cu	Cu	Biomass Burning	-0.57‰	38.68	115.25	January, 2021	Active sampling	MC-ICP-MS	Sample-Standard Bracketing	0.09	doi.org/10.1021/acs.est.1c05383
δ^{65}Cu	Cu	Biomass Burning	-0.04‰	38.68	115.25	January, 2021	Active sampling	MC-ICP-MS	Sample-Standard Bracketing	0.09	doi.org/10.1021/acs.est.1c05383
δ^{65}Cu	Cu	Biomass Burning	-0.95‰	38.68	115.25	January, 2021	Active sampling	MC-ICP-MS	Sample-Standard Bracketing	0.09	doi.org/10.1021/acs.est.1c05383
δ^{65}Cu	Cu	Biomass Burning	-0.33‰	38.68	115.25	January, 2021	Active sampling	MC-ICP-MS	Sample-Standard Bracketing	0.09	doi.org/10.1021/acs.est.1c05383
δ^{65}Cu	Cu	Biomass Burning	-0.08‰	38.68	115.25	January, 2021	Active sampling	MC-ICP-MS	Sample-Standard Bracketing	0.09	doi.org/10.1021/acs.est.1c05383
δ^{65}Cu	Cu	Biomass Burning	-0.62‰	38.68	115.25	January, 2021	Active sampling	MC-ICP-MS	Sample-Standard Bracketing	0.09	doi.org/10.1021/acs.est.1c05383
δ^{65}Cu	Cu	Biomass Burning	-0.03‰	38.68	115.25	January, 2021	Active sampling	MC-ICP-MS	Sample-Standard Bracketing	0.09	doi.org/10.1021/acs.est.1c05383
δ^{65}Cu	Cu	Biomass Burning	0.60‰	38.68	115.25	January, 2021	Active sampling	MC-ICP-MS	Sample-Standard Bracketing	0.09	doi.org/10.1021/acs.est.1c05383
δ^{65}Cu	Cu	Vehicle Exhausts	-0.15‰	40.32	116.00	January, 2021	Active sampling	MC-ICP-MS	Sample-Standard Bracketing	0.09	doi.org/10.1021/acs.est.1c05383
δ^{65}Cu	Cu	Vehicle Exhausts	-0.24‰	40.32	116.00	January, 2021	Active sampling	MC-ICP-MS	Sample-Standard Bracketing	0.09	doi.org/10.1021/acs.est.1c05383
δ^{65}Cu	Cu	Vehicle Exhausts	-0.05‰	40.32	116.00	January, 2021	Active sampling	MC-ICP-MS	Sample-Standard Bracketing	0.09	doi.org/10.1021/acs.est.1c05383
δ^{65}Cu	Cu	Vehicle Exhausts	-0.10‰	40.32	116.00	January, 2021	Active sampling	MC-ICP-MS	Sample-Standard Bracketing	0.09	doi.org/10.1021/acs.est.1c05383
δ^{65}Cu	Cu	Vehicle Exhausts	-0.08‰	40.32	116.00	January, 2021	Active sampling	MC-ICP-MS	Sample-Standard Bracketing	0.09	doi.org/10.1021/acs.est.1c05383

δ^{65}Cu	Cu	Vehicle Exhausts	-0.27‰	40.32	116.00	January, 2021	Active sampling	MC-ICP-MS	Sample-Standard Bracketing	0.09	doi.org/10.1021/acs.est.1c05383
δ^{65}Cu	Cu	Natural Soil	0.14‰	48.92	20.90	January, 2010	Active sampling	MC-ICP-MS	Sample-Standard Bracketing	0.04	10.1016/j.gca.2010.08.044
δ^{65}Cu	Cu	Natural Soil	-1.23‰	69.53	12.70	August, 2015	Active sampling	MC-ICP-MS	Sample-Standard Bracketing	0.05	dx.doi.org/10.1016/j.envpol.2017.05.030
δ^{65}Cu	Cu	Natural Soil	-0.98‰	69.53	12.70	August, 2015	Active sampling	MC-ICP-MS	Sample-Standard Bracketing	0.05	dx.doi.org/10.1016/j.envpol.2017.05.030
δ^{65}Cu	Cu	Natural Soil	-1.02‰	69.53	12.70	August, 2015	Active sampling	MC-ICP-MS	Sample-Standard Bracketing	0.05	dx.doi.org/10.1016/j.envpol.2017.05.030
δ^{65}Cu	Cu	Natural Soil	-2.23‰	69.53	12.70	August, 2015	Active sampling	MC-ICP-MS	Sample-Standard Bracketing	0.05	dx.doi.org/10.1016/j.envpol.2017.05.030
δ^{65}Cu	Cu	Natural Soil	-2.07‰	69.53	12.70	August, 2015	Active sampling	MC-ICP-MS	Sample-Standard Bracketing	0.05	dx.doi.org/10.1016/j.envpol.2017.05.030
δ^{65}Cu	Cu	Natural Soil	-3.94‰	69.53	12.70	August, 2015	Active sampling	MC-ICP-MS	Sample-Standard Bracketing	0.05	dx.doi.org/10.1016/j.envpol.2017.05.030
δ^{65}Cu	Cu	Natural Soil	-2.40‰	69.53	12.70	August, 2015	Active sampling	MC-ICP-MS	Sample-Standard Bracketing	0.05	dx.doi.org/10.1016/j.envpol.2017.05.030
δ^{65}Cu	Cu	Natural Soil	-0.43‰	69.53	12.70	August, 2015	Active sampling	MC-ICP-MS	Sample-Standard Bracketing	0.05	dx.doi.org/10.1016/j.envpol.2017.05.030
δ^{65}Cu	Cu	Natural Soil	-1.49‰	69.53	12.70	August, 2015	Active sampling	MC-ICP-MS	Sample-Standard Bracketing	0.05	dx.doi.org/10.1016/j.envpol.2017.05.030
δ^{65}Cu	Cu	Natural Soil	-0.60‰	69.53	12.70	August, 2015	Active sampling	MC-ICP-MS	Sample-Standard Bracketing	0.05	dx.doi.org/10.1016/j.envpol.2017.05.030
δ^{65}Cu	Cu	Natural Soil	-1.68‰	69.53	12.70	August, 2015	Active sampling	MC-ICP-MS	Sample-Standard Bracketing	0.05	dx.doi.org/10.1016/j.envpol.2017.05.030
δ^{65}Cu	Cu	Natural Soil	-3.23‰	69.53	12.70	August, 2015	Active sampling	MC-ICP-MS	Sample-Standard Bracketing	0.05	dx.doi.org/10.1016/j.envpol.2017.05.030
δ^{65}Cu	Cu	Natural Soil	-3.04‰	69.53	12.70	August, 2015	Active sampling	MC-ICP-MS	Sample-Standard Bracketing	0.05	dx.doi.org/10.1016/j.envpol.2017.05.030

$\delta^{65}Cu$	Cu	Natural Soil	-3.52‰	69.53	12.70	August, 2015	Active sampling	MC-ICP-MS	Sample-Standard Bracketing	0.05	dx.doi.org/10.1016/j.envpol.2017.05.030
$\delta^{65}Cu$	Cu	Natural Soil	0.03‰	-23.56	-46.73	January, 2018	Active sampling	MC-ICP-MS	Sample-Standard Bracketing	0.05	doi.org/10.1016/j.atmosenv.2018.11.007
$\delta^{65}Cu$	Cu	Natural Soil	0.05‰	-23.56	-46.73	January, 2018	Active sampling	MC-ICP-MS	Sample-Standard Bracketing	0.05	doi.org/10.1016/j.atmosenv.2018.11.007
$\delta^{65}Cu$	Cu	Natural Soil	0.03‰	37.42	78.50	January, 2007	Active sampling	MC-ICP-MS	Sample-Standard Bracketing	0.09	dx.doi.org/10.1016/j.talanta.2013.03.062
$\delta^{65}Cu$	Cu	Natural Soil	-0.02‰	37.42	78.50	January, 2008	Active sampling	MC-ICP-MS	Sample-Standard Bracketing	0.09	dx.doi.org/10.1016/j.talanta.2013.03.062
$\delta^{65}Cu$	Cu	Natural Soil	0.20‰	37.42	78.50	January, 2009	Active sampling	MC-ICP-MS	Sample-Standard Bracketing	0.09	dx.doi.org/10.1016/j.talanta.2013.03.062
$\delta^{65}Cu$	Cu	Natural Soil	-0.12‰	37.42	78.50	January, 2007	Active sampling	MC-ICP-MS	Sample-Standard Bracketing	0.09	dx.doi.org/10.1016/j.talanta.2013.03.062
$\delta^{65}Cu$	Cu	Natural Soil	0.37‰	37.42	78.50	January, 2008	Active sampling	MC-ICP-MS	Sample-Standard Bracketing	0.09	dx.doi.org/10.1016/j.talanta.2013.03.062
$\delta^{65}Cu$	Cu	Natural Soil	0.20‰	36.80	82.27	January, 2009	Active sampling	MC-ICP-MS	Sample-Standard Bracketing	0.09	dx.doi.org/10.1016/j.talanta.2013.03.062
$\delta^{65}Cu$	Cu	Natural Soil	0.31‰	36.80	82.27	January, 2007	Active sampling	MC-ICP-MS	Sample-Standard Bracketing	0.09	dx.doi.org/10.1016/j.talanta.2013.03.062
$\delta^{65}Cu$	Cu	Natural Soil	0.06‰	36.80	82.27	January, 2008	Active sampling	MC-ICP-MS	Sample-Standard Bracketing	0.09	dx.doi.org/10.1016/j.talanta.2013.03.062
$\delta^{65}Cu$	Cu	Natural Soil	-0.06‰	36.80	82.27	January, 2009	Active sampling	MC-ICP-MS	Sample-Standard Bracketing	0.09	dx.doi.org/10.1016/j.talanta.2013.03.062
$\delta^{65}Cu$	Cu	Natural Soil	-0.07‰	36.80	82.27	January, 2008	Active sampling	MC-ICP-MS	Sample-Standard Bracketing	0.09	dx.doi.org/10.1016/j.talanta.2013.03.062
$\delta^{65}Cu$	Cu	Natural Soil	-0.24‰	12.61	-8.12	January, 2013	Active sampling	MC-ICP-MS	Sample-Standard Bracketing	0.09	dx.doi.org/10.1016/j.talanta.2013.03.062
$\delta^{65}Cu$	Cu	Natural Soil	0.25‰	36.80	82.27	January, 2020	Active sampling	MC-ICP-MS	Sample-Standard Bracketing	0.07	10.3389/feart.2020.00167

$\delta^{65}Cu$	Cu	Natural Soil	0.17‰	36.80	82.27	January, 2020	Active sampling	MC-ICP-MS	Sample-Standard Bracketing	0.07	10.3389/feart.2020.00167
$\delta^{65}Cu$	Cu	Natural Soil	0.03‰	36.80	82.27	January, 2020	Active sampling	MC-ICP-MS	Sample-Standard Bracketing	0.07	10.3389/feart.2020.00167
$\delta^{65}Cu$	Cu	Natural Soil	0.20‰	36.80	82.27	January, 2020	Active sampling	MC-ICP-MS	Sample-Standard Bracketing	0.07	10.3389/feart.2020.00167
$\delta^{65}Cu$	Cu	Natural Soil	-0.54‰	36.80	82.27	January, 2020	Active sampling	MC-ICP-MS	Sample-Standard Bracketing	0.07	10.3389/feart.2020.00167
$\delta^{65}Cu$	Cu	Natural Soil	-0.31‰	36.80	82.27	January, 2020	Active sampling	MC-ICP-MS	Sample-Standard Bracketing	0.07	10.3389/feart.2020.00167
$\delta^{65}Cu$	Cu	Natural Soil	-0.02‰	36.80	82.27	January, 2020	Active sampling	MC-ICP-MS	Sample-Standard Bracketing	0.07	10.3389/feart.2020.00167
$\delta^{65}Cu$	Cu	Natural Soil	0.31‰	36.80	82.27	January, 2020	Active sampling	MC-ICP-MS	Sample-Standard Bracketing	0.07	10.3389/feart.2020.00167
$\delta^{65}Cu$	Cu	Natural Soil	-0.06‰	36.80	82.27	January, 2020	Active sampling	MC-ICP-MS	Sample-Standard Bracketing	0.07	10.3389/feart.2020.00167
$\delta^{65}Cu$	Cu	Natural Soil	0.15‰	36.80	82.27	January, 2020	Active sampling	MC-ICP-MS	Sample-Standard Bracketing	0.07	10.3389/feart.2020.00167
$\delta^{65}Cu$	Cu	Natural Soil	0.20‰	36.80	82.27	January, 2020	Active sampling	MC-ICP-MS	Sample-Standard Bracketing	0.07	10.3389/feart.2020.00167
$\delta^{65}Cu$	Cu	Natural Soil	0.06‰	36.80	82.27	January, 2020	Active sampling	MC-ICP-MS	Sample-Standard Bracketing	0.07	10.3389/feart.2020.00167
$\delta^{65}Cu$	Cu	Natural Soil	0.08‰	36.80	82.27	January, 2020	Active sampling	MC-ICP-MS	Sample-Standard Bracketing	0.07	10.3389/feart.2020.00167
$\delta^{65}Cu$	Cu	Natural Soil	0.38‰	36.80	82.27	January, 2020	Active sampling	MC-ICP-MS	Sample-Standard Bracketing	0.07	10.3389/feart.2020.00167
$\delta^{65}Cu$	Cu	Natural Soil	-0.12‰	36.80	82.27	January, 2020	Active sampling	MC-ICP-MS	Sample-Standard Bracketing	0.07	10.3389/feart.2020.00167
$\delta^{65}Cu$	Cu	Natural Soil	-0.06‰	36.80	82.27	January, 2020	Active sampling	MC-ICP-MS	Sample-Standard Bracketing	0.07	10.3389/feart.2020.00167

$\delta^{65}Cu$	Cu	Natural Soil	0.43‰	36.80	82.27	January, 2020	Active sampling	MC-ICP-MS	Sample-Standard Bracketing	0.07	10.3389/feart.2020.00167
$\delta^{65}Cu$	Cu	Natural Soil	0.39‰	36.80	82.27	January, 2020	Active sampling	MC-ICP-MS	Sample-Standard Bracketing	0.07	10.3389/feart.2020.00167
$\delta^{65}Cu$	Cu	Natural Soil	0.37‰	36.80	82.27	January, 2020	Active sampling	MC-ICP-MS	Sample-Standard Bracketing	0.07	10.3389/feart.2020.00167
$\delta^{65}Cu$	Cu	Natural Soil	-0.07‰	36.80	82.27	January, 2020	Active sampling	MC-ICP-MS	Sample-Standard Bracketing	0.07	10.3389/feart.2020.00167
$\delta^{65}Cu$	Cu	Natural Soil	0.15‰	43.73	107.45	January, 2020	Active sampling	MC-ICP-MS	Sample-Standard Bracketing	0.07	10.3389/feart.2020.00167
$\delta^{65}Cu$	Cu	Natural Soil	0.38‰	35.55	107.45	January, 2020	Active sampling	MC-ICP-MS	Sample-Standard Bracketing	0.07	10.3389/feart.2020.00167
$\delta^{65}Cu$	Cu	Natural Soil	0.31‰	35.55	107.45	January, 2020	Active sampling	MC-ICP-MS	Sample-Standard Bracketing	0.07	10.3389/feart.2020.00167
$\delta^{65}Cu$	Cu	Natural Soil	0.16‰	35.55	107.45	January, 2020	Active sampling	MC-ICP-MS	Sample-Standard Bracketing	0.07	10.3389/feart.2020.00167
$\delta^{65}Cu$	Cu	Natural Soil	0.23‰	35.55	107.45	January, 2020	Active sampling	MC-ICP-MS	Sample-Standard Bracketing	0.07	10.3389/feart.2020.00167
$\delta^{65}Cu$	Cu	Natural Soil	0.16‰	36.43	105.18	January, 2020	Active sampling	MC-ICP-MS	Sample-Standard Bracketing	0.07	10.3389/feart.2020.00167
$\delta^{65}Cu$	Cu	Natural Soil	0.16‰	36.43	105.18	January, 2020	Active sampling	MC-ICP-MS	Sample-Standard Bracketing	0.07	10.3389/feart.2020.00167
$\delta^{65}Cu$	Cu	Natural Soil	0.44‰	28.75	75.17	January, 2020	Active sampling	MC-ICP-MS	Sample-Standard Bracketing	0.07	10.3389/feart.2020.00167
$\delta^{65}Cu$	Cu	Natural Soil	0.52‰	28.75	75.17	January, 2020	Active sampling	MC-ICP-MS	Sample-Standard Bracketing	0.07	10.3389/feart.2020.00167
$\delta^{65}Cu$	Cu	Natural Soil	0.09‰	12.62	3.00	January, 2020	Active sampling	MC-ICP-MS	Sample-Standard Bracketing	0.07	10.3389/feart.2020.00167
$\delta^{65}Cu$	Cu	Natural Soil	0.11‰	12.62	3.00	January, 2020	Active sampling	MC-ICP-MS	Sample-Standard Bracketing	0.07	10.3389/feart.2020.00167

δ^{65}Cu	Cu	Natural Soil	-0.04‰	12.62	3.00	January, 2020	Active sampling	MC-ICP-MS	Sample-Standard Bracketing	0.07	10.3389/feart.2020.00167
δ^{65}Cu	Cu	Natural Soil	-0.20‰	12.62	3.00	January, 2020	Active sampling	MC-ICP-MS	Sample-Standard Bracketing	0.07	10.3389/feart.2020.00167
δ^{65}Cu	Cu	Natural Soil	-0.11‰	38.68	115.26	January, 2021	Active sampling	MC-ICP-MS	Sample-Standard Bracketing	0.09	doi.org/10.1021/acs.est.1c05383
δ^{65}Cu	Cu	Natural Soil	0.17‰	38.68	115.26	January, 2021	Active sampling	MC-ICP-MS	Sample-Standard Bracketing	0.09	doi.org/10.1021/acs.est.1c05383
δ^{65}Cu	Cu	Natural Soil	-0.04‰	38.68	115.26	January, 2021	Active sampling	MC-ICP-MS	Sample-Standard Bracketing	0.09	doi.org/10.1021/acs.est.1c05383
δ^{65}Cu	Cu	Natural Soil	-0.06‰	38.68	115.26	January, 2021	Active sampling	MC-ICP-MS	Sample-Standard Bracketing	0.09	doi.org/10.1021/acs.est.1c05383
δ^{65}Cu	Cu	Natural Soil	-0.17‰	38.68	115.26	January, 2021	Active sampling	MC-ICP-MS	Sample-Standard Bracketing	0.09	doi.org/10.1021/acs.est.1c05383
δ^{65}Cu	Cu	Natural Soil	0.03‰	38.68	115.26	January, 2021	Active sampling	MC-ICP-MS	Sample-Standard Bracketing	0.09	doi.org/10.1021/acs.est.1c05383
δ^{65}Cu	Cu	Natural Soil	-0.05‰	38.68	115.26	January, 2021	Active sampling	MC-ICP-MS	Sample-Standard Bracketing	0.09	doi.org/10.1021/acs.est.1c05383
δ^{65}Cu	Cu	Natural Soil	-0.09‰	38.68	115.26	January, 2021	Active sampling	MC-ICP-MS	Sample-Standard Bracketing	0.09	doi.org/10.1021/acs.est.1c05383
δ^{65}Cu	Cu	Natural Soil	-0.17‰	38.68	115.26	January, 2021	Active sampling	MC-ICP-MS	Sample-Standard Bracketing	0.09	doi.org/10.1021/acs.est.1c05383
δ^{65}Cu	Cu	Natural Soil	0.06‰	38.68	115.26	January, 2021	Active sampling	MC-ICP-MS	Sample-Standard Bracketing	0.09	doi.org/10.1021/acs.est.1c05383
δ^{65}Cu	Cu	Natural Soil	0.07‰	38.68	115.26	January, 2021	Active sampling	MC-ICP-MS	Sample-Standard Bracketing	0.09	doi.org/10.1021/acs.est.1c05383
δ^{65}Cu	Cu	Natural Soil	-0.08‰	38.68	115.26	January, 2021	Active sampling	MC-ICP-MS	Sample-Standard Bracketing	0.09	doi.org/10.1021/acs.est.1c05383
δ^{65}Cu	Cu	Natural Soil	0.14‰	38.68	115.26	January, 2021	Active sampling	MC-ICP-MS	Sample-Standard Bracketing	0.09	doi.org/10.1021/acs.est.1c05383

δ^{65}Cu	Cu	Natural Soil	0.07‰	38.68	115.26	January, 2021	Active sampling	MC-ICP-MS	Sample-Standard Bracketing	0.09	doi.org/10.1021/acs.est.1c05383
δ^{65}Cu	Cu	Natural Soil	-0.05‰	38.68	115.26	January, 2021	Active sampling	MC-ICP-MS	Sample-Standard Bracketing	0.09	doi.org/10.1021/acs.est.1c05383
δ^{65}Cu	Cu	Natural Soil	0.09‰	38.68	115.26	January, 2021	Active sampling	MC-ICP-MS	Sample-Standard Bracketing	0.09	doi.org/10.1021/acs.est.1c05383
δ^{65}Cu	Cu	Natural Soil	-0.06‰	38.68	115.26	January, 2021	Active sampling	MC-ICP-MS	Sample-Standard Bracketing	0.09	doi.org/10.1021/acs.est.1c05383
δ^{65}Cu	Cu	Natural Soil	-0.02‰	38.68	115.26	January, 2021	Active sampling	MC-ICP-MS	Sample-Standard Bracketing	0.09	doi.org/10.1021/acs.est.1c05383
δ^{65}Cu	Cu	Natural Soil	0.11‰	38.68	115.26	January, 2021	Active sampling	MC-ICP-MS	Sample-Standard Bracketing	0.09	doi.org/10.1021/acs.est.1c05383
δ^{65}Cu	Cu	Natural Soil	0.03‰	38.68	115.26	January, 2021	Active sampling	MC-ICP-MS	Sample-Standard Bracketing	0.09	doi.org/10.1021/acs.est.1c05383
δ^{65}Cu	Cu	Non-exhaust emissions-Brake Pad	0.62‰	51.50	-0.18	January, 2017	Active sampling	MC-ICP-MS	Sample-Standard Bracketing	0.05	http://dx.doi.org/10.1016/j.atmosenv.2017.06.020
δ^{65}Cu	Cu	Non-exhaust emissions-Brake Pad	0.63‰	51.50	-0.18	January, 2017	Active sampling	MC-ICP-MS	Sample-Standard Bracketing	0.05	http://dx.doi.org/10.1016/j.atmosenv.2017.06.020
δ^{65}Cu	Cu	Non-exhaust emissions-Brake Pad	0.28‰	51.50	-0.18	January, 2017	Active sampling	MC-ICP-MS	Sample-Standard Bracketing	0.05	http://dx.doi.org/10.1016/j.atmosenv.2017.06.020
δ^{65}Cu	Cu	Non-exhaust emissions-Brake Pad	0.18‰	35.21	129.07	January, 2021	Active sampling	MC-ICP-MS	Sample-Standard Bracketing	0.09	https://doi.org/10.1016/j.envpol.2021.118339
δ^{65}Cu	Cu	Non-exhaust emissions-Brake Pad	0.18‰	35.21	129.07	January, 2021	Active sampling	MC-ICP-MS	Sample-Standard Bracketing	0.09	https://doi.org/10.1016/j.envpol.2021.118339
δ^{65}Cu	Cu	Non-exhaust emissions-Brake Pad	0.16‰	35.21	129.07	January, 2021	Active sampling	MC-ICP-MS	Sample-Standard Bracketing	0.09	https://doi.org/10.1016/j.envpol.2021.118339
δ^{65}Cu	Cu	Non-exhaust emissions-Brake Pad	0.18‰	35.21	129.07	January, 2021	Active sampling	MC-ICP-MS	Sample-Standard Bracketing	0.09	https://doi.org/10.1016/j.envpol.2021.118339
δ^{65}Cu	Cu	Non-exhaust emissions-Brake Pad	0.23‰	35.21	129.07	January, 2021	Active sampling	MC-ICP-MS	Sample-Standard Bracketing	0.09	https://doi.org/10.1016/j.envpol.2021.118339

δ^{65}Cu	Cu	Non-exhaust emissions-Brake Pad	0.19‰	35.21	129.07	January, 2021	Active sampling	MC-ICP-MS	Sample-Standard Bracketing	0.09	https://doi.org/10.1016/j.envpol.2021.118339
δ^{65}Cu	Cu	Non-exhaust emissions-Brake Pad	0.18‰	35.21	129.07	January, 2021	Active sampling	MC-ICP-MS	Sample-Standard Bracketing	0.09	https://doi.org/10.1016/j.envpol.2021.118339
δ^{65}Cu	Cu	Non-exhaust emissions-Brake Pad	0.15‰	35.21	129.07	January, 2021	Active sampling	MC-ICP-MS	Sample-Standard Bracketing	0.09	https://doi.org/10.1016/j.envpol.2021.118339
δ^{65}Cu	Cu	Non-exhaust emissions-Brake Pad	0.17‰	35.21	129.07	January, 2021	Active sampling	MC-ICP-MS	Sample-Standard Bracketing	0.09	https://doi.org/10.1016/j.envpol.2021.118339
δ^{65}Cu	Cu	Non-exhaust emissions-Brake Pad	0.08‰	35.21	129.07	January, 2021	Active sampling	MC-ICP-MS	Sample-Standard Bracketing	0.09	https://doi.org/10.1016/j.envpol.2021.118339
δ^{65}Cu	Cu	Non-exhaust emissions-Brake Pad	0.16‰	35.21	129.07	January, 2021	Active sampling	MC-ICP-MS	Sample-Standard Bracketing	0.09	https://doi.org/10.1016/j.envpol.2021.118339
δ^{65}Cu	Cu	Non-exhaust emissions-Brake Pad	0.04‰	35.21	129.07	January, 2021	Active sampling	MC-ICP-MS	Sample-Standard Bracketing	0.09	https://doi.org/10.1016/j.envpol.2021.118339
δ^{65}Cu	Cu	Non-exhaust emissions-Brake Pad	0.12‰	35.21	129.07	January, 2021	Active sampling	MC-ICP-MS	Sample-Standard Bracketing	0.09	https://doi.org/10.1016/j.envpol.2021.118339
δ^{65}Cu	Cu	Non-exhaust emissions-Brake Pad	-0.02‰	47.05	-77.01	January, 2021	Active sampling	MC-ICP-MS	Sample-Standard Bracketing	0.09	https://doi.org/10.1016/j.envpol.2021.118339
δ^{65}Cu	Cu	Non-exhaust emissions-Brake Pad	-0.04‰	47.05	-77.01	January, 2021	Active sampling	MC-ICP-MS	Sample-Standard Bracketing	0.09	https://doi.org/10.1016/j.envpol.2021.118339
δ^{65}Cu	Cu	Non-exhaust emissions-Brake Pad	-0.03‰	52.51	13.03	January, 2021	Active sampling	MC-ICP-MS	Sample-Standard Bracketing	0.09	https://doi.org/10.1016/j.envpol.2021.118339
δ^{65}Cu	Cu	Non-exhaust emissions-Brake Pad	0.16‰	35.21	129.07	January, 2021	Active sampling	MC-ICP-MS	Sample-Standard Bracketing	0.09	https://doi.org/10.1016/j.envpol.2021.118339
δ^{65}Cu	Cu	Non-exhaust emissions-Brake Pad	0.21‰	35.21	129.07	January, 2021	Active sampling	MC-ICP-MS	Sample-Standard Bracketing	0.09	https://doi.org/10.1016/j.envpol.2021.118339
δ^{65}Cu	Cu	Non-exhaust emissions-Brake Pad	0.02‰	35.21	129.07	January, 2021	Active sampling	MC-ICP-MS	Sample-Standard Bracketing	0.09	https://doi.org/10.1016/j.envpol.2021.118339
δ^{65}Cu	Cu	Non-exhaust emissions-Brake Pad	0.06‰	35.21	129.07	January, 2021	Active sampling	MC-ICP-MS	Sample-Standard Bracketing	0.09	https://doi.org/10.1016/j.envpol.2021.118339

$\delta^{65}Cu$	Cu	Non-exhaust emissions-Tire	0.27‰	51.50	-0.18	January, 2017	Active sampling	MC-ICP-MS	Sample-Standard Bracketing	0.05	http://dx.doi.org/10.1016/j.atmosenv.2017.06.020
$\delta^{65}Cu$	Cu	Non-exhaust emissions-Tire	0.17‰	51.50	-0.18	January, 2017	Active sampling	MC-ICP-MS	Sample-Standard Bracketing	0.05	http://dx.doi.org/10.1016/j.atmosenv.2017.06.020
$\delta^{65}Cu$	Cu	Non-exhaust emissions-Tire	0.33‰	51.50	-0.18	January, 2017	Active sampling	MC-ICP-MS	Sample-Standard Bracketing	0.05	http://dx.doi.org/10.1016/j.atmosenv.2017.06.020
$\delta^{65}Cu$	Cu	Non-exhaust emissions-Tire	-0.42‰	35.21	129.07	January, 2021	Active sampling	MC-ICP-MS	Sample-Standard Bracketing	0.09	https://doi.org/10.1016/j.envpol.2021.118339
$\delta^{65}Cu$	Cu	Non-exhaust emissions-Tire	-0.56‰	35.21	129.07	January, 2021	Active sampling	MC-ICP-MS	Sample-Standard Bracketing	0.09	https://doi.org/10.1016/j.envpol.2021.118339
$\delta^{65}Cu$	Cu	Non-exhaust emissions-Road paint	-0.18‰	51.52	-0.20	8-28 July, 2010	Active sampling	MC-ICP-MS	Sample-Standard Bracketing	0.05	http://dx.doi.org/10.1016/j.atmosenv.2017.06.020
$\delta^{65}Cu$	Cu	Non-exhaust emissions-Road paint	0.56‰	51.52	-0.15	8-28 July, 2010	Active sampling	MC-ICP-MS	Sample-Standard Bracketing	0.05	http://dx.doi.org/10.1016/j.atmosenv.2017.06.020
$\delta^{65}Cu$	Cu	Non-exhaust emissions-Road paint	0.35‰	51.52	-0.15	8-28 July, 2010	Active sampling	MC-ICP-MS	Sample-Standard Bracketing	0.05	http://dx.doi.org/10.1016/j.atmosenv.2017.06.020
$\delta^{65}Cu$	Cu	Non-exhaust emissions-Road paint	0.71‰	51.52	-0.20	8-28 July, 2010	Active sampling	MC-ICP-MS	Sample-Standard Bracketing	0.05	http://dx.doi.org/10.1016/j.atmosenv.2017.06.020
$\delta^{65}Cu$	Cu	Non-exhaust emissions-Road paint	0.04‰	35.21	129.07	January, 2021	Active sampling	MC-ICP-MS	Sample-Standard Bracketing	0.09	https://doi.org/10.1016/j.envpol.2021.118339
$\delta^{65}Cu$	Cu	Non-exhaust emissions-Road paint	0.00‰	35.21	129.07	January, 2021	Active sampling	MC-ICP-MS	Sample-Standard Bracketing	0.09	https://doi.org/10.1016/j.envpol.2021.118339
$\delta^{65}Cu$	Cu	Non-exhaust emissions-Road paint	0.02‰	35.21	129.07	January, 2021	Active sampling	MC-ICP-MS	Sample-Standard Bracketing	0.09	https://doi.org/10.1016/j.envpol.2021.118339
$\delta^{65}Cu$	Cu	Non-exhaust emissions-Road paint	-0.02‰	35.21	129.07	January, 2021	Active sampling	MC-ICP-MS	Sample-Standard Bracketing	0.09	https://doi.org/10.1016/j.envpol.2021.118339
$\delta^{65}Cu$	Cu	Non-exhaust emissions-Road paint	0.02‰	35.21	129.07	January, 2021	Active sampling	MC-ICP-MS	Sample-Standard Bracketing	0.09	https://doi.org/10.1016/j.envpol.2021.118339
$\delta^{65}Cu$	Cu	Non-exhaust emissions-Road paint	-0.05‰	35.21	129.07	January, 2021	Active sampling	MC-ICP-MS	Sample-Standard Bracketing	0.09	https://doi.org/10.1016/j.envpol.2021.118339

$\delta^{65}Cu$	Cu	Non-exhaust emissions-Road paint	0.16‰	35.21	129.07	January, 2021	Active sampling	MC-ICP-MS	Sample-Standard Bracketing	0.09	https://doi.org/10.1016/j.envpol.2021.118339
$\delta^{65}Cu$	Cu	Non-exhaust emissions-Road paint	-0.01‰	35.21	129.07	January, 2021	Active sampling	MC-ICP-MS	Sample-Standard Bracketing	0.09	https://doi.org/10.1016/j.envpol.2021.118339
$\delta^{65}Cu$	Cu	Non-exhaust emissions-Road paint	0.23‰	35.21	129.07	January, 2021	Active sampling	MC-ICP-MS	Sample-Standard Bracketing	0.09	https://doi.org/10.1016/j.envpol.2021.118339
$\delta^{65}Cu$	Cu	Non-exhaust emissions-Road paint	0.44‰	35.21	129.07	January, 2021	Active sampling	MC-ICP-MS	Sample-Standard Bracketing	0.09	https://doi.org/10.1016/j.envpol.2021.118339
$\delta^{65}Cu$	Cu	Ore-related Emissions	-1.67‰	69.53	12.70	January, 2015	Active sampling	MC-ICP-MS	Sample-Standard Bracketing	0.05	dx.doi.org/10.1016/j.envpol.2017.05.030
$\delta^{65}Cu$	Cu	Ore-related Emissions	-1.68‰	69.53	12.70	January, 2015	Active sampling	MC-ICP-MS	Sample-Standard Bracketing	0.05	dx.doi.org/10.1016/j.envpol.2017.05.030
$\delta^{65}Cu$	Cu	Ore-related Emissions	0.48‰	40.00	116.33	January, 2021	Active sampling	MC-ICP-MS	Sample-Standard Bracketing	0.09	doi.org/10.1021/acs.est.1c05383
$\delta^{65}Cu$	Cu	Ore-related Emissions	0.66‰	40.00	116.33	January, 2021	Active sampling	MC-ICP-MS	Sample-Standard Bracketing	0.09	doi.org/10.1021/acs.est.1c05383
$\delta^{65}Cu$	Cu	Ore-related Emissions	0.62‰	40.00	116.33	January, 2021	Active sampling	MC-ICP-MS	Sample-Standard Bracketing	0.09	doi.org/10.1021/acs.est.1c05383
$\delta^{65}Cu$	Cu	Ore-related Emissions	0.62‰	40.00	116.33	January, 2021	Active sampling	MC-ICP-MS	Sample-Standard Bracketing	0.09	doi.org/10.1021/acs.est.1c05383
$\delta^{65}Cu$	Cu	Ore-related Emissions	0.68‰	40.00	116.33	January, 2021	Active sampling	MC-ICP-MS	Sample-Standard Bracketing	0.09	doi.org/10.1021/acs.est.1c05383
$\delta^{65}Cu$	Cu	Ore-related Emissions	-0.80‰	40.00	116.33	January, 2021	Active sampling	MC-ICP-MS	Sample-Standard Bracketing	0.09	doi.org/10.1021/acs.est.1c05383
$\delta^{65}Cu$	Cu	Ore-related Emissions	-0.82‰	40.00	116.33	January, 2021	Active sampling	MC-ICP-MS	Sample-Standard Bracketing	0.09	doi.org/10.1021/acs.est.1c05383
$\delta^{65}Cu$	Cu	Ore-related Emissions	-1.16‰	40.00	116.33	January, 2021	Active sampling	MC-ICP-MS	Sample-Standard Bracketing	0.09	doi.org/10.1021/acs.est.1c05383
$\delta^{65}Cu$	Cu	Ore-related Emissions	-1.92‰	40.00	116.33	January, 2021	Active sampling	MC-ICP-MS	Sample-Standard Bracketing	0.09	doi.org/10.1021/acs.est.1c05383

δ^{65}Cu	Cu	Ore-related Emissions	-1.06‰	40.00	116.33	January, 2021	Active sampling	MC-ICP-MS	Sample-Standard Bracketing	0.09	doi.org/10.1021/acs.est.1c05383
δ^{65}Cu	Cu	Ore-related Emissions	-1.24‰	39.21	117.93	January, 2021	Active sampling	MC-ICP-MS	Sample-Standard Bracketing	0.09	doi.org/10.1021/acs.est.1c05383
δ^{65}Cu	Cu	Ore-related Emissions	-0.99‰	39.21	117.93	January, 2021	Active sampling	MC-ICP-MS	Sample-Standard Bracketing	0.09	doi.org/10.1021/acs.est.1c05383
δ^{65}Cu	Cu	Ore-related Emissions	-1.09‰	39.21	117.93	January, 2021	Active sampling	MC-ICP-MS	Sample-Standard Bracketing	0.09	doi.org/10.1021/acs.est.1c05383
δ^{65}Cu	Cu	Ore-related Emissions	-0.66‰	39.21	117.93	January, 2021	Active sampling	MC-ICP-MS	Sample-Standard Bracketing	0.09	doi.org/10.1021/acs.est.1c05383
δ^{65}Cu	Cu	Ore-related Emissions	-1.06‰	39.21	117.93	January, 2021	Active sampling	MC-ICP-MS	Sample-Standard Bracketing	0.09	doi.org/10.1021/acs.est.1c05383
δ^{65}Cu	Cu	Ore-related Emissions	-1.24‰	39.21	117.93	January, 2021	Active sampling	MC-ICP-MS	Sample-Standard Bracketing	0.09	doi.org/10.1021/acs.est.1c05383
δ^{65}Cu	Cu	Ore-related Emissions	-0.99‰	39.21	117.93	January, 2021	Active sampling	MC-ICP-MS	Sample-Standard Bracketing	0.09	doi.org/10.1021/acs.est.1c05383
δ^{65}Cu	Cu	Ore-related Emissions	-1.09‰	39.21	117.93	January, 2021	Active sampling	MC-ICP-MS	Sample-Standard Bracketing	0.09	doi.org/10.1021/acs.est.1c05383
δ^{65}Cu	Cu	Ore-related Emissions	-0.66‰	39.21	117.93	January, 2021	Active sampling	MC-ICP-MS	Sample-Standard Bracketing	0.09	doi.org/10.1021/acs.est.1c05383
δ^{65}Cu	Cu	Ore-related Emissions	0.05‰	48.92	20.93	January, 2010	Active sampling	MC-ICP-MS	Sample-Standard Bracketing	0.04	10.1016/j.gca.2010.08.044
δ^{65}Cu	Cu	Ore-related Emissions	1.81‰	48.92	20.95	January, 2010	Active sampling	MC-ICP-MS	Sample-Standard Bracketing	0.04	10.1016/j.gca.2010.08.044
δ^{65}Cu	Cu	Ore-related Emissions	0.81‰	48.92	20.93	January, 2010	Active sampling	MC-ICP-MS	Sample-Standard Bracketing	0.04	10.1016/j.gca.2010.08.044
δ^{65}Cu	Cu	Ore-related Emissions	0.11‰	48.92	20.93	January, 2010	Active sampling	MC-ICP-MS	Sample-Standard Bracketing	0.04	10.1016/j.gca.2010.08.044
δ^{56}Fe	Fe	Biomass Burning	0.08‰	36.10	139.40	March, 2016	Active sampling	MC-ICP-MS	Sample-Standard Bracketing	0.07	10.3390/atmos10020076

δ^{56}Fe	Fe	Biomass Burning	0.09‰	36.10	139.40	March, 2016	Active sampling	MC-ICP-MS	Sample-Standard Bracketing	0.07	10.3390/atmos10020076
δ^{56}Fe	Fe	Biomass Burning	-0.55‰	52.37	9.75	January, 2007	Active sampling	MC-ICP-MS	Sample-Standard Bracketing	0.04	10.1021/es062288j
δ^{56}Fe	Fe	Biomass Burning	-0.40‰	52.37	9.75	January, 2007	Active sampling	MC-ICP-MS	Sample-Standard Bracketing	0.04	10.1021/es062288j
δ^{56}Fe	Fe	Biomass Burning	-1.13‰	52.37	9.75	January, 2007	Active sampling	MC-ICP-MS	Sample-Standard Bracketing	0.04	10.1021/es062288j
δ^{56}Fe	Fe	Biomass Burning	-0.63‰	52.37	9.75	January, 2007	Active sampling	MC-ICP-MS	Sample-Standard Bracketing	0.04	10.1021/es062288j
δ^{56}Fe	Fe	Biomass Burning	-0.61‰	52.37	9.75	January, 2007	Active sampling	MC-ICP-MS	Sample-Standard Bracketing	0.04	10.1021/es062288j
δ^{56}Fe	Fe	Biomass Burning	-0.22‰	52.37	9.75	January, 2007	Active sampling	MC-ICP-MS	Sample-Standard Bracketing	0.04	10.1021/es062288j
δ^{56}Fe	Fe	Biomass Burning	-0.54‰	52.37	9.75	January, 2007	Active sampling	MC-ICP-MS	Sample-Standard Bracketing	0.04	10.1021/es062288j
δ^{56}Fe	Fe	Biomass Burning	-0.28‰	52.37	9.75	January, 2007	Active sampling	MC-ICP-MS	Sample-Standard Bracketing	0.04	10.1021/es062288j
δ^{56}Fe	Fe	Biomass Burning	-0.31‰	52.37	9.75	January, 2007	Active sampling	MC-ICP-MS	Sample-Standard Bracketing	0.04	10.1021/es062288j
δ^{56}Fe	Fe	Biomass Burning	-0.20‰	52.37	9.75	January, 2007	Active sampling	MC-ICP-MS	Sample-Standard Bracketing	0.04	10.1021/es062288j
δ^{56}Fe	Fe	Biomass Burning	-0.64‰	52.37	9.75	January, 2007	Active sampling	MC-ICP-MS	Sample-Standard Bracketing	0.04	10.1021/es062288j
δ^{56}Fe	Fe	Biomass Burning	-0.51‰	52.37	9.75	January, 2007	Active sampling	MC-ICP-MS	Sample-Standard Bracketing	0.04	10.1021/es062288j
δ^{56}Fe	Fe	Biomass Burning	-0.30‰	52.37	9.75	January, 2007	Active sampling	MC-ICP-MS	Sample-Standard Bracketing	0.04	10.1021/es062288j
δ^{56}Fe	Fe	Biomass Burning	-0.24‰	52.37	9.75	January, 2007	Active sampling	MC-ICP-MS	Sample-Standard Bracketing	0.04	10.1021/es062288j

δ^{56}Fe	Fe	Biomass Burning	-0.69‰	52.37	9.75	January, 2007	Active sampling	MC-ICP-MS	Sample-Standard Bracketing	0.04	10.1021/es062288j
δ^{56}Fe	Fe	Biomass Burning	-0.72‰	52.37	9.75	January, 2007	Active sampling	MC-ICP-MS	Sample-Standard Bracketing	0.04	10.1021/es062288j
δ^{56}Fe	Fe	Biomass Burning	-0.48‰	52.37	9.75	January, 2007	Active sampling	MC-ICP-MS	Sample-Standard Bracketing	0.04	10.1021/es062288j
δ^{56}Fe	Fe	Biomass Burning	-0.70‰	52.37	9.75	January, 2007	Active sampling	MC-ICP-MS	Sample-Standard Bracketing	0.04	10.1021/es062288j
δ^{56}Fe	Fe	Biomass Burning	0.02‰	52.37	9.75	January, 2007	Active sampling	MC-ICP-MS	Sample-Standard Bracketing	0.04	10.1021/es062288j
δ^{56}Fe	Fe	Biomass Burning	0.06‰	52.37	9.75	January, 2007	Active sampling	MC-ICP-MS	Sample-Standard Bracketing	0.04	10.1021/es062288j
δ^{56}Fe	Fe	Biomass Burning	0.01‰	52.37	9.75	January, 2007	Active sampling	MC-ICP-MS	Sample-Standard Bracketing	0.04	10.1021/es062288j
δ^{56}Fe	Fe	Biomass Burning	0.14‰	52.37	9.75	January, 2007	Active sampling	MC-ICP-MS	Sample-Standard Bracketing	0.04	10.1021/es062288j
δ^{56}Fe	Fe	Biomass Burning	0.09‰	52.37	9.75	January, 2007	Active sampling	MC-ICP-MS	Sample-Standard Bracketing	0.04	10.1021/es062288j
δ^{56}Fe	Fe	Biomass Burning	0.06‰	52.37	9.75	January, 2007	Active sampling	MC-ICP-MS	Sample-Standard Bracketing	0.04	10.1021/es062288j
δ^{56}Fe	Fe	Biomass Burning	0.07‰	52.37	9.75	January, 2007	Active sampling	MC-ICP-MS	Sample-Standard Bracketing	0.04	10.1021/es062288j
δ^{56}Fe	Fe	Biomass Burning	0.07‰	52.37	9.75	January, 2007	Active sampling	MC-ICP-MS	Sample-Standard Bracketing	0.04	10.1021/es062288j
δ^{56}Fe	Fe	Biomass Burning	-0.01‰	52.37	9.75	January, 2007	Active sampling	MC-ICP-MS	Sample-Standard Bracketing	0.04	10.1021/es062288j
δ^{56}Fe	Fe	Biomass Burning	0.07‰	52.37	9.75	January, 2007	Active sampling	MC-ICP-MS	Sample-Standard Bracketing	0.04	10.1021/es062288j
δ^{56}Fe	Fe	Biomass Burning	0.02‰	52.37	9.75	January, 2007	Active sampling	MC-ICP-MS	Sample-Standard Bracketing	0.04	10.1021/es062288j

δ^{56}Fe	Fe	Biomass Burning	-0.05‰	52.37	9.75	January, 2007	Active sampling	MC-ICP-MS	Sample-Standard Bracketing	0.04	10.1021/es062288j
δ^{56}Fe	Fe	Biomass Burning	0.17‰	52.37	9.75	January, 2007	Active sampling	MC-ICP-MS	Sample-Standard Bracketing	0.04	10.1021/es062288j
δ^{56}Fe	Fe	Biomass Burning	0.08‰	52.37	9.75	January, 2007	Active sampling	MC-ICP-MS	Sample-Standard Bracketing	0.04	10.1021/es062288j
δ^{56}Fe	Fe	Biomass Burning	-0.02‰	52.37	9.75	January, 2007	Active sampling	MC-ICP-MS	Sample-Standard Bracketing	0.04	10.1021/es062288j
δ^{56}Fe	Fe	Biomass Burning	0.09‰	52.37	9.75	January, 2007	Active sampling	MC-ICP-MS	Sample-Standard Bracketing	0.04	10.1021/es062288j
δ^{56}Fe	Fe	Biomass Burning	-0.03‰	11.52	-3.85	30 June, 2009	Active sampling	MC-ICP-MS	Sample-Standard Bracketing	0.07	http://dx.doi.org/10.1016/j.chemgeo.2014.07.003
δ^{56}Fe	Fe	Biomass Burning	-0.13‰	11.52	-3.85	30 November, 2009	Active sampling	MC-ICP-MS	Sample-Standard Bracketing	0.07	http://dx.doi.org/10.1016/j.chemgeo.2014.07.003
δ^{56}Fe	Fe	Biomass Burning	0.02‰	11.52	-3.85	30 June, 2009	Active sampling	MC-ICP-MS	Sample-Standard Bracketing	0.07	http://dx.doi.org/10.1016/j.chemgeo.2014.07.003
δ^{56}Fe	Fe	Biomass Burning	-0.18‰	11.52	-3.85	30 November, 2009	Active sampling	MC-ICP-MS	Sample-Standard Bracketing	0.07	http://dx.doi.org/10.1016/j.chemgeo.2014.07.003
δ^{56}Fe	Fe	Biomass Burning	-0.44‰	11.52	-3.85	January, 2003	Active sampling	MC-ICP-MS	Sample-Standard Bracketing	0.07	http://dx.doi.org/10.1016/j.chemgeo.2014.07.003
δ^{56}Fe	Fe	Biomass Burning	-0.75‰	11.52	-3.85	January, 2003	Active sampling	MC-ICP-MS	Sample-Standard Bracketing	0.07	http://dx.doi.org/10.1016/j.chemgeo.2014.07.003
δ^{56}Fe	Fe	Biomass Burning	-2.14‰	46.64	8.46	January, 2019	Active sampling	MC-ICP-MS	Sample-Standard Bracketing	0.07	10.16258/j.cnki.1674-5906.2019.06.022 in Chinese with English abstract
δ^{56}Fe	Fe	Biomass Burning	-1.94‰	46.64	8.46	January, 2019	Active sampling	MC-ICP-MS	Sample-Standard Bracketing	0.07	10.16258/j.cnki.1674-5906.2019.06.022 in Chinese with English abstract
δ^{56}Fe	Fe	Biomass Burning	-1.80‰	46.64	8.46	January, 2019	Active sampling	MC-ICP-MS	Sample-Standard Bracketing	0.07	10.16258/j.cnki.1674-5906.2019.06.022 in Chinese with English abstract

$\delta^{56}Fe$	Fe	Biomass Burning	-1.68‰	46.64	8.46	January, 2019	Active sampling	MC-ICP-MS	Sample-Standard Bracketing	0.07	10.16258/j.cnki.1674-5906.2019.06.022 in Chinese with English abstract
$\delta^{56}Fe$	Fe	Biomass Burning	-1.63‰	46.64	8.46	January, 2019	Active sampling	MC-ICP-MS	Sample-Standard Bracketing	0.07	10.16258/j.cnki.1674-5906.2019.06.022 in Chinese with English abstract
$\delta^{56}Fe$	Fe	Biomass Burning	-1.43‰	46.64	8.46	January, 2019	Active sampling	MC-ICP-MS	Sample-Standard Bracketing	0.07	10.16258/j.cnki.1674-5906.2019.06.022 in Chinese with English abstract
$\delta^{56}Fe$	Fe	Biomass Burning	-1.39‰	46.64	8.46	January, 2019	Active sampling	MC-ICP-MS	Sample-Standard Bracketing	0.07	10.16258/j.cnki.1674-5906.2019.06.022 in Chinese with English abstract
$\delta^{56}Fe$	Fe	Biomass Burning	-1.29‰	46.64	8.46	January, 2019	Active sampling	MC-ICP-MS	Sample-Standard Bracketing	0.07	10.16258/j.cnki.1674-5906.2019.06.022 in Chinese with English abstract
$\delta^{56}Fe$	Fe	Biomass Burning	-1.18‰	46.64	8.46	January, 2019	Active sampling	MC-ICP-MS	Sample-Standard Bracketing	0.07	10.16258/j.cnki.1674-5906.2019.06.022 in Chinese with English abstract
$\delta^{56}Fe$	Fe	Biomass Burning	-0.75‰	46.64	8.46	January, 2019	Active sampling	MC-ICP-MS	Sample-Standard Bracketing	0.07	10.16258/j.cnki.1674-5906.2019.06.022 in Chinese with English abstract
$\delta^{56}Fe$	Fe	Biomass Burning	-0.59‰	46.64	8.46	January, 2019	Active sampling	MC-ICP-MS	Sample-Standard Bracketing	0.07	10.16258/j.cnki.1674-5906.2019.06.022 in Chinese with English abstract
$\delta^{56}Fe$	Fe	Biomass Burning	-0.43‰	46.64	8.46	January, 2019	Active sampling	MC-ICP-MS	Sample-Standard Bracketing	0.07	10.16258/j.cnki.1674-5906.2019.06.022 in Chinese with English abstract
$\delta^{56}Fe$	Fe	Biomass Burning	-0.37‰	46.64	8.46	January, 2019	Active sampling	MC-ICP-MS	Sample-Standard Bracketing	0.07	10.16258/j.cnki.1674-5906.2019.06.022 in Chinese with English abstract
$\delta^{56}Fe$	Fe	Biomass Burning	-0.23‰	46.64	8.46	January, 2019	Active sampling	MC-ICP-MS	Sample-Standard Bracketing	0.07	10.16258/j.cnki.1674-5906.2019.06.022 in Chinese with English abstract
$\delta^{56}Fe$	Fe	Biomass Burning	0.16‰	46.64	8.46	January, 2019	Active sampling	MC-ICP-MS	Sample-Standard Bracketing	0.07	10.16258/j.cnki.1674-5906.2019.06.022

										in Chinese with English abstract
$\delta^{56}Fe$	Fe	Biomass Burning	0.47‰	46.64	8.46	January, 2019	Active sampling	MC-ICP-MS	Sample-Standard Bracketing	0.07	10.16258/j.cnki.1674-5906.2019.06.022 in Chinese with English abstract
$\delta^{56}Fe$	Fe	Biomass Burning	0.45‰	46.64	8.46	January, 2019	Active sampling	MC-ICP-MS	Sample-Standard Bracketing	0.07	10.16258/j.cnki.1674-5906.2019.06.022 in Chinese with English abstract
$\delta^{56}Fe$	Fe	Biomass Burning	0.28‰	46.64	8.46	January, 2019	Active sampling	MC-ICP-MS	Sample-Standard Bracketing	0.07	10.16258/j.cnki.1674-5906.2019.06.022 in Chinese with English abstract
$\delta^{56}Fe$	Fe	Biomass Burning	0.18‰	46.64	8.46	January, 2019	Active sampling	MC-ICP-MS	Sample-Standard Bracketing	0.07	10.16258/j.cnki.1674-5906.2019.06.022 in Chinese with English abstract
$\delta^{56}Fe$	Fe	Biomass Burning	-0.12‰	46.64	8.46	January, 2019	Active sampling	MC-ICP-MS	Sample-Standard Bracketing	0.07	10.16258/j.cnki.1674-5906.2019.06.022 in Chinese with English abstract
$\delta^{56}Fe$	Fe	Biomass Burning	-0.16‰	46.64	8.46	January, 2019	Active sampling	MC-ICP-MS	Sample-Standard Bracketing	0.07	10.16258/j.cnki.1674-5906.2019.06.022 in Chinese with English abstract
$\delta^{56}Fe$	Fe	Biomass Burning	-0.46‰	46.64	8.46	January, 2019	Active sampling	MC-ICP-MS	Sample-Standard Bracketing	0.07	10.16258/j.cnki.1674-5906.2019.06.022 in Chinese with English abstract
$\delta^{56}Fe$	Fe	Biomass Burning	-0.54‰	46.64	8.46	January, 2019	Active sampling	MC-ICP-MS	Sample-Standard Bracketing	0.07	10.16258/j.cnki.1674-5906.2019.06.022 in Chinese with English abstract
$\delta^{56}Fe$	Fe	Biomass Burning	-0.64‰	46.64	8.46	January, 2019	Active sampling	MC-ICP-MS	Sample-Standard Bracketing	0.07	10.16258/j.cnki.1674-5906.2019.06.022 in Chinese with English abstract
$\delta^{56}Fe$	Fe	Biomass Burning	-0.93‰	46.64	8.46	January, 2019	Active sampling	MC-ICP-MS	Sample-Standard Bracketing	0.07	10.16258/j.cnki.1674-5906.2019.06.022 in Chinese with English abstract
$\delta^{56}Fe$	Fe	Biomass Burning	-1.45‰	46.64	8.46	January, 2019	Active sampling	MC-ICP-MS	Sample-Standard Bracketing	0.07	10.16258/j.cnki.1674-5906.2019.06.022 in Chinese with English abstract

$\delta^{56}Fe$	Fe	Biomass Burning	-1.52‰	46.64	8.46	January, 2019	Active sampling	MC-ICP-MS	Sample-Standard Bracketing	0.07	10.16258/j.cnki.1674-5906.2019.06.022 in Chinese with English abstract
$\delta^{56}Fe$	Fe	Biomass Burning	-1.74‰	46.64	8.46	January, 2019	Active sampling	MC-ICP-MS	Sample-Standard Bracketing	0.07	10.16258/j.cnki.1674-5906.2019.06.022 in Chinese with English abstract
$\delta^{56}Fe$	Fe	Biomass Burning	-2.93‰	46.64	8.46	January, 2019	Active sampling	MC-ICP-MS	Sample-Standard Bracketing	0.07	10.16258/j.cnki.1674-5906.2019.06.022 in Chinese with English abstract
$\delta^{56}Fe$	Fe	Biomass Burning	-3.92‰	46.64	8.46	January, 2019	Active sampling	MC-ICP-MS	Sample-Standard Bracketing	0.07	10.16258/j.cnki.1674-5906.2019.06.022 in Chinese with English abstract
$\delta^{56}Fe$	Fe	Biomass Burning	-0.21‰	23.13	113.26	January, 2019	Active sampling	MC-ICP-MS	Sample-Standard Bracketing	0.07	10.16258/j.cnki.1674-5906.2019.06.022 in Chinese with English abstract
$\delta^{56}Fe$	Fe	Biomass Burning	-0.26‰	23.13	113.26	January, 2019	Active sampling	MC-ICP-MS	Sample-Standard Bracketing	0.07	10.16258/j.cnki.1674-5906.2019.06.022 in Chinese with English abstract
$\delta^{56}Fe$	Fe	Biomass Burning	-0.32‰	23.13	113.26	January, 2019	Active sampling	MC-ICP-MS	Sample-Standard Bracketing	0.07	10.16258/j.cnki.1674-5906.2019.06.022 in Chinese with English abstract
$\delta^{56}Fe$	Fe	Biomass Burning	-0.36‰	23.13	113.26	January, 2019	Active sampling	MC-ICP-MS	Sample-Standard Bracketing	0.07	10.16258/j.cnki.1674-5906.2019.06.022 in Chinese with English abstract
$\delta^{56}Fe$	Fe	Biomass Burning	-0.44‰	23.13	113.26	January, 2019	Active sampling	MC-ICP-MS	Sample-Standard Bracketing	0.07	10.16258/j.cnki.1674-5906.2019.06.022 in Chinese with English abstract
$\delta^{56}Fe$	Fe	Biomass Burning	-0.54‰	23.13	113.26	January, 2019	Active sampling	MC-ICP-MS	Sample-Standard Bracketing	0.07	10.16258/j.cnki.1674-5906.2019.06.022 in Chinese with English abstract
$\delta^{56}Fe$	Fe	Biomass Burning	-0.68‰	23.13	113.26	January, 2019	Active sampling	MC-ICP-MS	Sample-Standard Bracketing	0.07	10.16258/j.cnki.1674-5906.2019.06.022 in Chinese with English abstract
$\delta^{56}Fe$	Fe	Biomass Burning	-0.76‰	23.13	113.26	January, 2019	Active sampling	MC-ICP-MS	Sample-Standard Bracketing	0.07	10.16258/j.cnki.1674-5906.2019.06.022

											in Chinese with English abstract
δ^{56}Fe	Fe	Biomass Burning	-0.84‰	23.13	113.26	January, 2019	Active sampling	MC-ICP-MS	Sample-Standard Bracketing	0.07	10.16258/j.cnki.1674-5906.2019.06.022 in Chinese with English abstract
δ^{56}Fe	Fe	Biomass Burning	-1.04‰	23.13	113.26	January, 2019	Active sampling	MC-ICP-MS	Sample-Standard Bracketing	0.07	10.16258/j.cnki.1674-5906.2019.06.022 in Chinese with English abstract
δ^{56}Fe	Fe	Biomass Burning	-1.14‰	23.13	113.26	January, 2019	Active sampling	MC-ICP-MS	Sample-Standard Bracketing	0.07	10.16258/j.cnki.1674-5906.2019.06.022 in Chinese with English abstract
δ^{56}Fe	Fe	Biomass Burning	-1.19‰	23.13	113.26	January, 2019	Active sampling	MC-ICP-MS	Sample-Standard Bracketing	0.07	10.16258/j.cnki.1674-5906.2019.06.022 in Chinese with English abstract
δ^{56}Fe	Fe	Biomass Burning	-1.31‰	23.13	113.26	January, 2019	Active sampling	MC-ICP-MS	Sample-Standard Bracketing	0.07	10.16258/j.cnki.1674-5906.2019.06.022 in Chinese with English abstract
δ^{56}Fe	Fe	Biomass Burning	-1.37‰	23.13	113.26	January, 2019	Active sampling	MC-ICP-MS	Sample-Standard Bracketing	0.07	10.16258/j.cnki.1674-5906.2019.06.022 in Chinese with English abstract
δ^{56}Fe	Fe	Biomass Burning	-1.58‰	23.13	113.26	January, 2019	Active sampling	MC-ICP-MS	Sample-Standard Bracketing	0.07	10.16258/j.cnki.1674-5906.2019.06.022 in Chinese with English abstract
δ^{56}Fe	Fe	Biomass Burning	-1.64‰	23.13	113.26	January, 2019	Active sampling	MC-ICP-MS	Sample-Standard Bracketing	0.07	10.16258/j.cnki.1674-5906.2019.06.022 in Chinese with English abstract
δ^{56}Fe	Fe	Biomass Burning	-1.68‰	23.13	113.26	January, 2019	Active sampling	MC-ICP-MS	Sample-Standard Bracketing	0.07	10.16258/j.cnki.1674-5906.2019.06.022 in Chinese with English abstract
δ^{56}Fe	Fe	Biomass Burning	-1.82‰	23.13	113.26	January, 2019	Active sampling	MC-ICP-MS	Sample-Standard Bracketing	0.07	10.16258/j.cnki.1674-5906.2019.06.022 in Chinese with English abstract
δ^{56}Fe	Fe	Biomass Burning	-1.91‰	23.13	113.26	January, 2019	Active sampling	MC-ICP-MS	Sample-Standard Bracketing	0.07	10.16258/j.cnki.1674-5906.2019.06.022 in Chinese with English abstract

δ^{56}Fe	Fe	Biomass Burning	-0.24‰	52.37	9.72	January, 2019	Active sampling	MC-ICP-MS	Sample-Standard Bracketing	0.07	10.16258/j.cnki.1674-5906.2019.06.022 in Chinese with English abstract
δ^{56}Fe	Fe	Biomass Burning	-0.30‰	52.37	9.72	January, 2019	Active sampling	MC-ICP-MS	Sample-Standard Bracketing	0.07	10.16258/j.cnki.1674-5906.2019.06.022 in Chinese with English abstract
δ^{56}Fe	Fe	Biomass Burning	-0.50‰	52.37	9.72	January, 2019	Active sampling	MC-ICP-MS	Sample-Standard Bracketing	0.07	10.16258/j.cnki.1674-5906.2019.06.022 in Chinese with English abstract
δ^{56}Fe	Fe	Biomass Burning	-0.64‰	52.37	9.72	January, 2019	Active sampling	MC-ICP-MS	Sample-Standard Bracketing	0.07	10.16258/j.cnki.1674-5906.2019.06.022 in Chinese with English abstract
δ^{56}Fe	Fe	Biomass Burning	-0.74‰	52.37	9.72	January, 2019	Active sampling	MC-ICP-MS	Sample-Standard Bracketing	0.07	10.16258/j.cnki.1674-5906.2019.06.022 in Chinese with English abstract
δ^{56}Fe	Fe	Biomass Burning	-0.74‰	52.37	9.72	January, 2019	Active sampling	MC-ICP-MS	Sample-Standard Bracketing	0.07	10.16258/j.cnki.1674-5906.2019.06.022 in Chinese with English abstract
δ^{56}Fe	Fe	Biomass Burning	-0.93‰	52.37	9.72	January, 2019	Active sampling	MC-ICP-MS	Sample-Standard Bracketing	0.07	10.16258/j.cnki.1674-5906.2019.06.022 in Chinese with English abstract
δ^{56}Fe	Fe	Biomass Burning	-0.14‰	52.37	9.72	January, 2019	Active sampling	MC-ICP-MS	Sample-Standard Bracketing	0.07	10.16258/j.cnki.1674-5906.2019.06.022 in Chinese with English abstract
δ^{56}Fe	Fe	Biomass Burning	-0.40‰	52.37	9.72	January, 2019	Active sampling	MC-ICP-MS	Sample-Standard Bracketing	0.07	10.16258/j.cnki.1674-5906.2019.06.022 in Chinese with English abstract
δ^{56}Fe	Fe	Biomass Burning	-0.96‰	52.37	9.72	January, 2019	Active sampling	MC-ICP-MS	Sample-Standard Bracketing	0.07	10.16258/j.cnki.1674-5906.2019.06.022 in Chinese with English abstract
δ^{56}Fe	Fe	Biomass Burning	-1.03‰	52.37	9.72	January, 2019	Active sampling	MC-ICP-MS	Sample-Standard Bracketing	0.07	10.16258/j.cnki.1674-5906.2019.06.022 in Chinese with English abstract
δ^{56}Fe	Fe	Biomass Burning	-1.15‰	52.37	9.72	January, 2019	Active sampling	MC-ICP-MS	Sample-Standard Bracketing	0.07	10.16258/j.cnki.1674-5906.2019.06.022

											in Chinese with English abstract
δ^{56}Fe	Fe	Biomass Burning	-1.49‰	52.37	9.72	January, 2019	Active sampling	MC-ICP-MS	Sample-Standard Bracketing	0.07	10.16258/j.cnki.1674-5906.2019.06.022 in Chinese with English abstract
δ^{56}Fe	Fe	Biomass Burning	-1.20‰	52.37	9.72	January, 2019	Active sampling	MC-ICP-MS	Sample-Standard Bracketing	0.07	10.16258/j.cnki.1674-5906.2019.06.022 in Chinese with English abstract
δ^{56}Fe	Fe	Biomass Burning	-0.92‰	52.37	9.72	January, 2019	Active sampling	MC-ICP-MS	Sample-Standard Bracketing	0.07	10.16258/j.cnki.1674-5906.2019.06.022 in Chinese with English abstract
δ^{56}Fe	Fe	Biomass Burning	-0.75‰	52.37	9.72	January, 2019	Active sampling	MC-ICP-MS	Sample-Standard Bracketing	0.07	10.16258/j.cnki.1674-5906.2019.06.022 in Chinese with English abstract
δ^{56}Fe	Fe	Biomass Burning	-1.54‰	52.37	9.72	January, 2019	Active sampling	MC-ICP-MS	Sample-Standard Bracketing	0.07	10.16258/j.cnki.1674-5906.2019.06.022 in Chinese with English abstract
δ^{56}Fe	Fe	Biomass Burning	-1.19‰	52.37	9.72	January, 2019	Active sampling	MC-ICP-MS	Sample-Standard Bracketing	0.07	10.16258/j.cnki.1674-5906.2019.06.022 in Chinese with English abstract
δ^{56}Fe	Fe	Biomass Burning	-1.01‰	52.37	9.72	January, 2019	Active sampling	MC-ICP-MS	Sample-Standard Bracketing	0.07	10.16258/j.cnki.1674-5906.2019.06.022 in Chinese with English abstract
δ^{56}Fe	Fe	Biomass Burning	-0.31‰	52.37	9.72	January, 2019	Active sampling	MC-ICP-MS	Sample-Standard Bracketing	0.07	10.16258/j.cnki.1674-5906.2019.06.022 in Chinese with English abstract
δ^{56}Fe	Fe	Biomass Burning	-1.05‰	52.37	9.72	January, 2019	Active sampling	MC-ICP-MS	Sample-Standard Bracketing	0.07	10.16258/j.cnki.1674-5906.2019.06.022 in Chinese with English abstract
δ^{56}Fe	Fe	Biomass Burning	-0.22‰	52.37	9.72	January, 2019	Active sampling	MC-ICP-MS	Sample-Standard Bracketing	0.07	10.16258/j.cnki.1674-5906.2019.06.022 in Chinese with English abstract
δ^{56}Fe	Fe	Biomass Burning	-0.13‰	52.37	9.72	January, 2019	Active sampling	MC-ICP-MS	Sample-Standard Bracketing	0.07	10.16258/j.cnki.1674-5906.2019.06.022 in Chinese with English abstract

$\delta^{56}Fe$	Fe	Biomass Burning	-0.38‰	52.37	9.72	January, 2019	Active sampling	MC-ICP-MS	Sample-Standard Bracketing	0.07	10.16258/j.cnki.1674-5906.2019.06.022 in Chinese with English abstract
$\delta^{56}Fe$	Fe	Biomass Burning	-0.31‰	52.37	9.72	January, 2019	Active sampling	MC-ICP-MS	Sample-Standard Bracketing	0.07	10.16258/j.cnki.1674-5906.2019.06.022 in Chinese with English abstract
$\delta^{56}Fe$	Fe	Biomass Burning	-1.46‰	52.37	9.72	January, 2019	Active sampling	MC-ICP-MS	Sample-Standard Bracketing	0.07	10.16258/j.cnki.1674-5906.2019.06.022 in Chinese with English abstract
$\delta^{56}Fe$	Fe	Biomass Burning	-1.13‰	52.37	9.72	January, 2019	Active sampling	MC-ICP-MS	Sample-Standard Bracketing	0.07	10.16258/j.cnki.1674-5906.2019.06.022 in Chinese with English abstract
$\delta^{56}Fe$	Fe	Biomass Burning	-0.62‰	52.37	9.72	January, 2019	Active sampling	MC-ICP-MS	Sample-Standard Bracketing	0.07	10.16258/j.cnki.1674-5906.2019.06.022 in Chinese with English abstract
$\delta^{56}Fe$	Fe	Biomass Burning	-0.55‰	52.37	9.72	January, 2019	Active sampling	MC-ICP-MS	Sample-Standard Bracketing	0.07	10.16258/j.cnki.1674-5906.2019.06.022 in Chinese with English abstract
$\delta^{56}Fe$	Fe	Biomass Burning	-0.41‰	52.37	9.72	January, 2019	Active sampling	MC-ICP-MS	Sample-Standard Bracketing	0.07	10.16258/j.cnki.1674-5906.2019.06.022 in Chinese with English abstract
$\delta^{56}Fe$	Fe	Biomass Burning	0.60‰	46.64	8.46	January, 2019	Active sampling	MC-ICP-MS	Sample-Standard Bracketing	0.07	10.16258/j.cnki.1674-5906.2019.06.022 in Chinese with English abstract
$\delta^{56}Fe$	Fe	Biomass Burning	0.49‰	46.64	8.46	January, 2019	Active sampling	MC-ICP-MS	Sample-Standard Bracketing	0.07	10.16258/j.cnki.1674-5906.2019.06.022 in Chinese with English abstract
$\delta^{56}Fe$	Fe	Biomass Burning	0.24‰	46.64	8.46	January, 2019	Active sampling	MC-ICP-MS	Sample-Standard Bracketing	0.07	10.16258/j.cnki.1674-5906.2019.06.022 in Chinese with English abstract
$\delta^{56}Fe$	Fe	Biomass Burning	0.16‰	46.64	8.46	January, 2019	Active sampling	MC-ICP-MS	Sample-Standard Bracketing	0.07	10.16258/j.cnki.1674-5906.2019.06.022 in Chinese with English abstract
$\delta^{56}Fe$	Fe	Biomass Burning	0.11‰	46.64	8.46	January, 2019	Active sampling	MC-ICP-MS	Sample-Standard Bracketing	0.07	10.16258/j.cnki.1674-5906.2019.06.022

										in Chinese with English abstract
δ^{56}Fe	Fe	Biomass Burning	0.00‰	46.64	8.46	January, 2019	Active sampling	MC-ICP-MS	Sample-Standard Bracketing	0.07	10.16258/j.cnki.1674-5906.2019.06.022 in Chinese with English abstract
δ^{56}Fe	Fe	Biomass Burning	-0.18‰	46.64	8.46	January, 2019	Active sampling	MC-ICP-MS	Sample-Standard Bracketing	0.07	10.16258/j.cnki.1674-5906.2019.06.022 in Chinese with English abstract
δ^{56}Fe	Fe	Biomass Burning	-0.26‰	46.64	8.46	January, 2019	Active sampling	MC-ICP-MS	Sample-Standard Bracketing	0.07	10.16258/j.cnki.1674-5906.2019.06.022 in Chinese with English abstract
δ^{56}Fe	Fe	Biomass Burning	-0.31‰	46.64	8.46	January, 2019	Active sampling	MC-ICP-MS	Sample-Standard Bracketing	0.07	10.16258/j.cnki.1674-5906.2019.06.022 in Chinese with English abstract
δ^{56}Fe	Fe	Biomass Burning	-0.47‰	46.64	8.46	January, 2019	Active sampling	MC-ICP-MS	Sample-Standard Bracketing	0.07	10.16258/j.cnki.1674-5906.2019.06.022 in Chinese with English abstract
δ^{56}Fe	Fe	Biomass Burning	-0.68‰	46.64	8.46	January, 2019	Active sampling	MC-ICP-MS	Sample-Standard Bracketing	0.07	10.16258/j.cnki.1674-5906.2019.06.022 in Chinese with English abstract
δ^{56}Fe	Fe	Biomass Burning	-1.08‰	46.64	8.46	January, 2019	Active sampling	MC-ICP-MS	Sample-Standard Bracketing	0.07	10.16258/j.cnki.1674-5906.2019.06.022 in Chinese with English abstract
δ^{56}Fe	Fe	Biomass Burning	-1.27‰	46.64	8.46	January, 2019	Active sampling	MC-ICP-MS	Sample-Standard Bracketing	0.07	10.16258/j.cnki.1674-5906.2019.06.022 in Chinese with English abstract
δ^{56}Fe	Fe	Biomass Burning	-1.45‰	46.64	8.46	January, 2019	Active sampling	MC-ICP-MS	Sample-Standard Bracketing	0.07	10.16258/j.cnki.1674-5906.2019.06.022 in Chinese with English abstract
δ^{56}Fe	Fe	Biomass Burning	-1.70‰	46.64	8.46	January, 2019	Active sampling	MC-ICP-MS	Sample-Standard Bracketing	0.07	10.16258/j.cnki.1674-5906.2019.06.022 in Chinese with English abstract
δ^{56}Fe	Fe	Biomass Burning	-2.09‰	46.64	8.46	January, 2019	Active sampling	MC-ICP-MS	Sample-Standard Bracketing	0.07	10.16258/j.cnki.1674-5906.2019.06.022 in Chinese with English abstract

$\delta^{56}Fe$	Fe	Biomass Burning	-0.02‰	52.37	9.72	January, 2019	Active sampling	MC-ICP-MS	Sample-Standard Bracketing	0.07	10.16258/j.cnki.1674-5906.2019.06.022 in Chinese with English abstract
$\delta^{56}Fe$	Fe	Biomass Burning	0.04‰	52.37	9.72	January, 2019	Active sampling	MC-ICP-MS	Sample-Standard Bracketing	0.07	10.16258/j.cnki.1674-5906.2019.06.022 in Chinese with English abstract
$\delta^{56}Fe$	Fe	Biomass Burning	0.14‰	52.37	9.72	January, 2019	Active sampling	MC-ICP-MS	Sample-Standard Bracketing	0.07	10.16258/j.cnki.1674-5906.2019.06.022 in Chinese with English abstract
$\delta^{56}Fe$	Fe	Biomass Burning	0.21‰	52.37	9.72	January, 2019	Active sampling	MC-ICP-MS	Sample-Standard Bracketing	0.07	10.16258/j.cnki.1674-5906.2019.06.022 in Chinese with English abstract
$\delta^{56}Fe$	Fe	Biomass Burning	0.28‰	52.37	9.72	January, 2019	Active sampling	MC-ICP-MS	Sample-Standard Bracketing	0.07	10.16258/j.cnki.1674-5906.2019.06.022 in Chinese with English abstract
$\delta^{56}Fe$	Fe	Biomass Burning	-0.08‰	52.37	9.72	January, 2019	Active sampling	MC-ICP-MS	Sample-Standard Bracketing	0.07	10.16258/j.cnki.1674-5906.2019.06.022 in Chinese with English abstract
$\delta^{56}Fe$	Fe	Biomass Burning	0.00‰	52.37	9.72	January, 2019	Active sampling	MC-ICP-MS	Sample-Standard Bracketing	0.07	10.16258/j.cnki.1674-5906.2019.06.022 in Chinese with English abstract
$\delta^{56}Fe$	Fe	Biomass Burning	0.01‰	52.37	9.72	January, 2019	Active sampling	MC-ICP-MS	Sample-Standard Bracketing	0.07	10.16258/j.cnki.1674-5906.2019.06.022 in Chinese with English abstract
$\delta^{56}Fe$	Fe	Biomass Burning	0.06‰	52.37	9.72	January, 2019	Active sampling	MC-ICP-MS	Sample-Standard Bracketing	0.07	10.16258/j.cnki.1674-5906.2019.06.022 in Chinese with English abstract
$\delta^{56}Fe$	Fe	Biomass Burning	0.14‰	52.37	9.72	January, 2019	Active sampling	MC-ICP-MS	Sample-Standard Bracketing	0.07	10.16258/j.cnki.1674-5906.2019.06.022 in Chinese with English abstract
$\delta^{56}Fe$	Fe	Biomass Burning	0.16‰	52.37	9.72	January, 2019	Active sampling	MC-ICP-MS	Sample-Standard Bracketing	0.07	10.16258/j.cnki.1674-5906.2019.06.022 in Chinese with English abstract
$\delta^{56}Fe$	Fe	Biomass Burning	0.02‰	52.37	9.72	January, 2019	Active sampling	MC-ICP-MS	Sample-Standard Bracketing	0.07	10.16258/j.cnki.1674-5906.2019.06.022

											in Chinese with English abstract
$\delta^{56}Fe$	Fe	Biomass Burning	-0.05‰	52.37	9.72	January, 2019	Active sampling	MC-ICP-MS	Sample-Standard Bracketing	0.07	10.16258/j.cnki.1674-5906.2019.06.022 in Chinese with English abstract
$\delta^{56}Fe$	Fe	Biomass Burning	-0.11‰	52.37	9.72	January, 2019	Active sampling	MC-ICP-MS	Sample-Standard Bracketing	0.07	10.16258/j.cnki.1674-5906.2019.06.022 in Chinese with English abstract
$\delta^{56}Fe$	Fe	Coal Combustion	-0.10‰	34.25	132.59	January, 2011	Active sampling	MC-ICP-MS	Sample-Standard Bracketing	0.09	10.1246/cl.160451
$\delta^{56}Fe$	Fe	Coal Combustion	-0.66‰	34.41	132.78	January, 2012	Active sampling	MC-ICP-MS	Sample-Standard Bracketing	0.09	10.1246/cl.160451
$\delta^{56}Fe$	Fe	Coal Combustion	-0.08‰	34.41	132.78	January, 2011	Active sampling	MC-ICP-MS	Sample-Standard Bracketing	0.09	10.1246/cl.160451
$\delta^{56}Fe$	Fe	Coal Combustion	0.22‰	40.43	-79.98	January, 2013	Active sampling	MC-ICP-MS	Sample-Standard Bracketing	0.07	10.1002/2013GL057713
$\delta^{56}Fe$	Fe	Coal Combustion	0.22‰	33.90	-112.10	January, 2013	Active sampling	MC-ICP-MS	Sample-Standard Bracketing	0.07	10.1002/2013GL057713
$\delta^{56}Fe$	Fe	Coal Combustion	0.20‰	40.05	116.42	January, 2020	Active sampling	MC-ICP-MS	Sample-Standard Bracketing	0.03	10.7524/j.issn.0254-6108.2020081502 in Chinese with English abstract
$\delta^{56}Fe$	Fe	Coal Combustion	0.05‰	29.43	-98.49	January, 2020	Active sampling	MC-ICP-MS	Sample-Standard Bracketing	0.03	10.7524/j.issn.0254-6108.2020081502 in Chinese with English abstract
$\delta^{56}Fe$	Fe	Coal Combustion	0.75‰	39.01	-98.48	January, 2020	Active sampling	MC-ICP-MS	Sample-Standard Bracketing	0.03	10.7524/j.issn.0254-6108.2020081502 in Chinese with English abstract
$\delta^{56}Fe$	Fe	Natural Soil	-0.05‰	33.29	-111.72	January, 2008	Active sampling	MC-ICP-MS	Sample-Standard Bracketing	0.08	10.1021/es900023w
$\delta^{56}Fe$	Fe	Natural Soil	-0.02‰	33.31	-111.71	January, 2008	Active sampling	MC-ICP-MS	Sample-Standard Bracketing	0.08	10.1021/es900023w
$\delta^{56}Fe$	Fe	Natural Soil	0.04‰	33.31	-111.71	January, 2008	Active sampling	MC-ICP-MS	Sample-Standard Bracketing	0.08	10.1021/es900023w

$\delta^{56}Fe$	Fe	Natural Soil	-0.04‰	33.29	-111.72	January, 2008	Active sampling	MC-ICP-MS	Sample-Standard Bracketing	0.08	10.1021/es900023w
$\delta^{56}Fe$	Fe	Natural Soil	0.01‰	33.31	-111.71	January, 2008	Active sampling	MC-ICP-MS	Sample-Standard Bracketing	0.08	10.1021/es900023w
$\delta^{56}Fe$	Fe	Natural Soil	-0.07‰	33.31	-111.71	January, 2008	Active sampling	MC-ICP-MS	Sample-Standard Bracketing	0.08	10.1021/es900023w
$\delta^{56}Fe$	Fe	Natural Soil	0.13‰	34.27	-112.37	January, 2013	Active sampling	MC-ICP-MS	Sample-Standard Bracketing	0.07	10.1002/2013GL057713
$\delta^{56}Fe$	Fe	Natural Soil	0.09‰	16.72	-22.92	January, 2013	Active sampling	MC-ICP-MS	Sample-Standard Bracketing	0.07	10.1002/2013GL057713
$\delta^{56}Fe$	Fe	Natural Soil	-0.04‰	34.27	-112.37	January, 2020	Active sampling	MC-ICP-MS	Sample-Standard Bracketing	0.03	10.7524/j.issn.0254-6108.2020081502 in Chinese with English abstract
$\delta^{56}Fe$	Fe	Natural Soil	-0.05‰	35.77	109.44	January, 2020	Active sampling	MC-ICP-MS	Sample-Standard Bracketing	0.03	10.7524/j.issn.0254-6108.2020081502 in Chinese with English abstract
$\delta^{56}Fe$	Fe	Natural Soil	0.21‰	42.56	89.11	23 April, 2010	Active sampling	MC-ICP-MS	Sample-Standard Bracketing	0.03	10.7524/j.issn.0254-6108.2020081502 in Chinese with English abstract
$\delta^{56}Fe$	Fe	Vehicle Exhausts	0.61‰	33.90	-112.10	January, 2013	Active sampling	MC-ICP-MS	Sample-Standard Bracketing	0.07	10.1002/2013GL057713
$\delta^{56}Fe$	Fe	Vehicle Exhausts	0.24‰	41.90	12.62	January, 2013	Active sampling	MC-ICP-MS	Sample-Standard Bracketing	0.07	10.1002/2013GL057713
$\delta^{56}Fe$	Fe	Vehicle Exhausts	0.10‰	41.90	12.62	January, 2013	Active sampling	MC-ICP-MS	Sample-Standard Bracketing	0.07	10.1002/2013GL057713
$\delta^{56}Fe$	Fe	Vehicle Exhausts	0.40‰	41.90	12.62	January, 2013	Active sampling	MC-ICP-MS	Sample-Standard Bracketing	0.07	10.1002/2013GL057713
$\delta^{56}Fe$	Fe	Vehicle Exhausts	0.47‰	33.90	-112.10	January, 2013	Active sampling	MC-ICP-MS	Sample-Standard Bracketing	0.07	10.1002/2013GL057713
$\delta^{56}Fe$	Fe	Vehicle Exhausts	0.04‰	33.47	-112.02	January, 2009	Active sampling	MC-ICP-MS	Sample-Standard Bracketing	0.03	10.1016/j.scitotenv.2009.05.053

$\delta^{56}Fe$	Fe	Vehicle Exhausts	0.11‰	33.47	-112.02	January, 2009	Active sampling	MC-ICP-MS	Sample-Standard Bracketing	0.03	10.1016/j.scitotenv.2009.05.053
$\delta^{56}Fe$	Fe	Vehicle Exhausts	-0.03‰	33.90	-112.10	January, 2013	Active sampling	MC-ICP-MS	Sample-Standard Bracketing	0.07	10.1002/2013GL057713
$\delta^{56}Fe$	Fe	Ore-related Emissions	0.80‰	51.03	2.37	1 July, 2003	Active sampling	MC-ICP-MS	Sample-Standard Bracketing	0.07	10.1016/j.chemosphere.2008.08.042
$\delta^{56}Fe$	Fe	Ore-related Emissions	0.53‰	51.03	2.37	20 March, 2003	Active sampling	MC-ICP-MS	Sample-Standard Bracketing	0.07	10.1016/j.chemosphere.2008.08.042
$\delta^{56}Fe$	Fe	Ore-related Emissions	0.08‰	51.03	2.37	January, 2008	Active sampling	MC-ICP-MS	Sample-Standard Bracketing	0.07	10.1016/j.chemosphere.2008.08.042
$\delta^{56}Fe$	Fe	Ore-related Emissions	-0.16‰	-27.15	-50.53	January, 2002	Active sampling	MC-ICP-MS	Sample-Standard Bracketing	0.07	10.1016/j.chemosphere.2008.08.042
$\delta^{56}Fe$	Fe	Ore-related Emissions	1.19‰	-27.15	-50.53	January, 2003	Active sampling	MC-ICP-MS	Sample-Standard Bracketing	0.07	10.1016/j.chemosphere.2008.08.042
$\delta^{56}Fe$	Fe	Ore-related Emissions	-0.07‰	-27.15	-50.53	January, 2004	Active sampling	MC-ICP-MS	Sample-Standard Bracketing	0.07	10.1016/j.chemosphere.2008.08.042
$\delta^{56}Fe$	Fe	Non-exhaust emissions	0.12‰	33.47	-112.02	January, 2009	Active sampling	MC-ICP-MS	Sample-Standard Bracketing	0.03	10.1016/j.scitotenv.2009.05.053
$\delta^{56}Fe$	Fe	Non-exhaust emissions	-0.08‰	33.47	-112.02	January, 2009	Active sampling	MC-ICP-MS	Sample-Standard Bracketing	0.03	10.1016/j.scitotenv.2009.05.053
$\delta^{56}Fe$	Fe	Non-exhaust emissions	-0.10‰	33.49	-111.72	January, 2008	Active sampling	MC-ICP-MS	Sample-Standard Bracketing	0.08	10.1021/es900023w
$\delta^{56}Fe$	Fe	Non-exhaust emissions	0.14‰	33.31	-111.72	January, 2008	Active sampling	MC-ICP-MS	Sample-Standard Bracketing	0.08	10.1021/es900023w
$\delta^{56}Fe$	Fe	Non-exhaust emissions	-0.02‰	33.49	-111.72	January, 2008	Active sampling	MC-ICP-MS	Sample-Standard Bracketing	0.08	10.1021/es900023w
$\delta^{56}Fe$	Fe	Non-exhaust emissions	0.00‰	33.31	-111.72	January, 2008	Active sampling	MC-ICP-MS	Sample-Standard Bracketing	0.08	10.1021/es900023w
$\delta^{56}Fe$	Fe	Non-exhaust emissions	0.19‰	33.47	-112.02	January, 2009	Active sampling	MC-ICP-MS	Sample-Standard Bracketing	0.03	10.1016/j.scitotenv.2009.05.053

$\delta^{56}Fe$	Fe	Non-exhaust emissions	0.18‰	33.47	-112.02	January, 2009	Active sampling	MC-ICP-MS	Sample-Standard Bracketing	0.03	10.1016/j.scitotenv.2009.05.053
$\delta^{56}Fe$	Fe	Non-exhaust emissions	0.42‰	33.47	-112.02	January, 2009	Active sampling	MC-ICP-MS	Sample-Standard Bracketing	0.03	10.1016/j.scitotenv.2009.05.053
$\delta^{56}Fe$	Fe	Non-exhaust emissions	0.61‰	33.47	-112.02	January, 2009	Active sampling	MC-ICP-MS	Sample-Standard Bracketing	0.03	10.1016/j.scitotenv.2009.05.053
$\delta^{66}Zn$	Zn	Biomass Burning	0.36‰	49.07	6.20	January, 2001	Active sampling	MC-ICP-MS	Sample-Standard Bracketing	0.08	doi.org/10.1021/es0609654
$\delta^{66}Zn$	Zn	Biomass Burning	0.51‰	49.07	6.20	January, 2001	Active sampling	MC-ICP-MS	Sample-Standard Bracketing	0.08	doi.org/10.1021/es0609654
$\delta^{66}Zn$	Zn	Biomass Burning	0.79‰	49.07	6.20	January, 2001	Active sampling	MC-ICP-MS	Sample-Standard Bracketing	0.08	doi.org/10.1021/es0609654
$\delta^{66}Zn$	Zn	Biomass Burning	0.27‰	49.07	6.20	January, 2001	Active sampling	MC-ICP-MS	Sample-Standard Bracketing	0.08	doi.org/10.1021/es0609654
$\delta^{66}Zn$	Zn	Biomass Burning	0.29‰	49.07	6.20	January, 2001	Active sampling	MC-ICP-MS	Sample-Standard Bracketing	0.08	doi.org/10.1021/es0609654
$\delta^{66}Zn$	Zn	Biomass Burning	0.37‰	49.07	6.20	January, 2001	Active sampling	MC-ICP-MS	Sample-Standard Bracketing	0.08	doi.org/10.1021/es0609654
$\delta^{66}Zn$	Zn	Biomass Burning	0.49‰	49.07	6.20	January, 2001	Active sampling	MC-ICP-MS	Sample-Standard Bracketing	0.08	doi.org/10.1021/es0609654
$\delta^{66}Zn$	Zn	Biomass Burning	0.52‰	49.07	6.20	January, 2001	Active sampling	MC-ICP-MS	Sample-Standard Bracketing	0.08	doi.org/10.1021/es0609654
$\delta^{66}Zn$	Zn	Biomass Burning	0.30‰	49.07	6.20	January, 2001	Active sampling	MC-ICP-MS	Sample-Standard Bracketing	0.08	doi.org/10.1021/es0609654
$\delta^{66}Zn$	Zn	Biomass Burning	0.63‰	49.07	6.20	January, 2001	Active sampling	MC-ICP-MS	Sample-Standard Bracketing	0.08	doi.org/10.1021/es0609654
$\delta^{66}Zn$	Zn	Biomass Burning	0.37‰	49.07	6.20	January, 2001	Active sampling	MC-ICP-MS	Sample-Standard Bracketing	0.08	doi.org/10.1021/es0609654
$\delta^{66}Zn$	Zn	Biomass Burning	0.29‰	49.07	6.20	January, 2001	Active sampling	MC-ICP-MS	Sample-Standard Bracketing	0.08	doi.org/10.1021/es0609654

$\delta^{66}Zn$	Zn	Biomass Burning	0.31‰	49.07	6.20	January, 2001	Active sampling	MC-ICP-MS	Sample-Standard Bracketing	0.08	doi.org/10.1021/es0609654
$\delta^{66}Zn$	Zn	Biomass Burning	0.14‰	49.07	6.20	January, 2001	Active sampling	MC-ICP-MS	Sample-Standard Bracketing	0.08	doi.org/10.1021/es0609654
$\delta^{66}Zn$	Zn	Biomass Burning	0.42‰	49.07	6.20	January, 2001	Active sampling	MC-ICP-MS	Sample-Standard Bracketing	0.08	doi.org/10.1021/es0609654
$\delta^{66}Zn$	Zn	Biomass Burning	0.46‰	49.07	6.20	January, 2001	Active sampling	MC-ICP-MS	Sample-Standard Bracketing	0.08	doi.org/10.1021/es0609654
$\delta^{66}Zn$	Zn	Biomass Burning	0.37‰	49.07	6.20	January, 2001	Active sampling	MC-ICP-MS	Sample-Standard Bracketing	0.08	doi.org/10.1021/es0609654
$\delta^{66}Zn$	Zn	Biomass Burning	0.49‰	49.07	6.20	January, 2001	Active sampling	MC-ICP-MS	Sample-Standard Bracketing	0.08	doi.org/10.1021/es0609654
$\delta^{66}Zn$	Zn	Biomass Burning	0.50‰	49.07	6.20	January, 2001	Active sampling	MC-ICP-MS	Sample-Standard Bracketing	0.08	doi.org/10.1021/es0609654
$\delta^{66}Zn$	Zn	Biomass Burning	0.64‰	46.00	7.50	January, 2023	Active sampling	MC-ICP-MS	Sample-Standard Bracketing	0.07	http://dx.doi.org/10.1016/j.scitotenv.2023.162490
$\delta^{66}Zn$	Zn	Biomass Burning	0.62‰	46.00	7.50	January, 2023	Active sampling	MC-ICP-MS	Sample-Standard Bracketing	0.07	http://dx.doi.org/10.1016/j.scitotenv.2023.162490
$\delta^{66}Zn$	Zn	Biomass Burning	0.57‰	46.00	7.50	January, 2023	Active sampling	MC-ICP-MS	Sample-Standard Bracketing	0.07	http://dx.doi.org/10.1016/j.scitotenv.2023.162490
$\delta^{66}Zn$	Zn	Biomass Burning	0.34‰	46.00	7.50	January, 2023	Active sampling	MC-ICP-MS	Sample-Standard Bracketing	0.07	http://dx.doi.org/10.1016/j.scitotenv.2023.162490
$\delta^{66}Zn$	Zn	Biomass Burning	0.33‰	46.00	7.50	January, 2023	Active sampling	MC-ICP-MS	Sample-Standard Bracketing	0.07	http://dx.doi.org/10.1016/j.scitotenv.2023.162490
$\delta^{66}Zn$	Zn	Biomass Burning	0.28‰	46.00	7.50	January, 2023	Active sampling	MC-ICP-MS	Sample-Standard Bracketing	0.07	http://dx.doi.org/10.1016/j.scitotenv.2023.162490
$\delta^{66}Zn$	Zn	Biomass Burning	0.28‰	46.00	7.50	January, 2023	Active sampling	MC-ICP-MS	Sample-Standard Bracketing	0.07	http://dx.doi.org/10.1016/j.scitotenv.2023.162490
$\delta^{66}Zn$	Zn	Biomass Burning	0.26‰	46.00	7.50	January, 2023	Active sampling	MC-ICP-MS	Sample-Standard Bracketing	0.07	http://dx.doi.org/10.1016/j.scitotenv.2023.162490

$\delta^{66}Zn$	Zn	Biomass Burning	0.21‰	46.00	7.50	January, 2023	Active sampling	MC-ICP-MS	Sample-Standard Bracketing	0.07	http://dx.doi.org/10.1016/j.scitotenv.2023.162490
$\delta^{66}Zn$	Zn	Biomass Burning	0.22‰	46.00	7.50	January, 2023	Active sampling	MC-ICP-MS	Sample-Standard Bracketing	0.07	http://dx.doi.org/10.1016/j.scitotenv.2023.162490
$\delta^{66}Zn$	Zn	Biomass Burning	0.20‰	46.00	7.50	January, 2023	Active sampling	MC-ICP-MS	Sample-Standard Bracketing	0.07	http://dx.doi.org/10.1016/j.scitotenv.2023.162490
$\delta^{66}Zn$	Zn	Biomass Burning	0.16‰	46.00	7.50	January, 2023	Active sampling	MC-ICP-MS	Sample-Standard Bracketing	0.07	http://dx.doi.org/10.1016/j.scitotenv.2023.162490
$\delta^{66}Zn$	Zn	Biomass Burning	0.66‰	46.00	7.50	January, 2018	Active sampling	MC-ICP-MS	Sample-Standard Bracketing	0.06	10.1111/nph.15146
$\delta^{66}Zn$	Zn	Biomass Burning	1.06‰	46.00	7.50	January, 2018	Active sampling	MC-ICP-MS	Sample-Standard Bracketing	0.06	10.1111/nph.15146
$\delta^{66}Zn$	Zn	Biomass Burning	1.08‰	46.00	7.50	January, 2018	Active sampling	MC-ICP-MS	Sample-Standard Bracketing	0.06	10.1111/nph.15146
$\delta^{66}Zn$	Zn	Biomass Burning	0.79‰	46.00	7.50	January, 2018	Active sampling	MC-ICP-MS	Sample-Standard Bracketing	0.06	10.1111/nph.15146
$\delta^{66}Zn$	Zn	Biomass Burning	0.86‰	46.00	7.50	January, 2018	Active sampling	MC-ICP-MS	Sample-Standard Bracketing	0.06	10.1111/nph.15146
$\delta^{66}Zn$	Zn	Biomass Burning	0.78‰	46.00	7.50	January, 2018	Active sampling	MC-ICP-MS	Sample-Standard Bracketing	0.06	10.1111/nph.15146
$\delta^{66}Zn$	Zn	Biomass Burning	1.07‰	46.00	7.50	January, 2018	Active sampling	MC-ICP-MS	Sample-Standard Bracketing	0.06	10.1111/nph.15146
$\delta^{66}Zn$	Zn	Biomass Burning	1.28‰	46.00	7.50	January, 2018	Active sampling	MC-ICP-MS	Sample-Standard Bracketing	0.06	10.1111/nph.15146
$\delta^{66}Zn$	Zn	Biomass Burning	0.96‰	46.00	7.50	January, 2018	Active sampling	MC-ICP-MS	Sample-Standard Bracketing	0.06	10.1111/nph.15146
$\delta^{66}Zn$	Zn	Biomass Burning	1.08‰	46.00	7.50	January, 2018	Active sampling	MC-ICP-MS	Sample-Standard Bracketing	0.06	10.1111/nph.15146
$\delta^{66}Zn$	Zn	Biomass Burning	0.48‰	26.35	106.42	January, 2010	Active sampling	MC-ICP-MS	Sample-Standard Bracketing	-	CNKI:SUN:DXQY.0.2010-04-032

$\delta^{66}Zn$	Zn	Biomass Burning	0.52‰	26.35	106.42	January, 2010	Active sampling	MC-ICP-MS	Sample-Standard Bracketing	-	CNKI:SUN:DXQY.0.2010-04-032
$\delta^{66}Zn$	Zn	Biomass Burning	0.69‰	26.35	106.42	January, 2010	Active sampling	MC-ICP-MS	Sample-Standard Bracketing	-	CNKI:SUN:DXQY.0.2010-04-032
$\delta^{66}Zn$	Zn	Biomass Burning	0.69‰	26.35	106.42	January, 2010	Active sampling	MC-ICP-MS	Sample-Standard Bracketing	-	CNKI:SUN:DXQY.0.2010-04-032
$\delta^{66}Zn$	Zn	Biomass Burning	0.17‰	39.91	116.39	January, 2014	Active sampling	MC-ICP-MS	Sample-Standard Bracketing	-	10.14050/j.cnki.1672-9250.2014.04.004 in Chinese with English abstract
$\delta^{66}Zn$	Zn	Biomass Burning	0.20‰	39.91	116.39	January, 2014	Active sampling	MC-ICP-MS	Sample-Standard Bracketing	-	10.14050/j.cnki.1672-9250.2014.04.004 in Chinese with English abstract
$\delta^{66}Zn$	Zn	Biomass Burning	0.18‰	39.91	116.39	January, 2014	Active sampling	MC-ICP-MS	Sample-Standard Bracketing	-	10.14050/j.cnki.1672-9250.2014.04.004 in Chinese with English abstract
$\delta^{66}Zn$	Zn	Coal Combustion	0.18‰	48.92	20.93	2010	Active sampling	MC-ICP-MS	Sample-Standard Bracketing	0.06	10.1016/j.gca.2010.08.044
$\delta^{66}Zn$	Zn	Coal Combustion	1.03‰	48.92	20.93	January, 2015	Active sampling	MC-ICP-MS	Sample-Standard Bracketing	0.07	doi.org/10.1021/acs.est.5b02402
$\delta^{66}Zn$	Zn	Coal Combustion	1.07‰	48.92	20.93	January, 2015	Active sampling	MC-ICP-MS	Sample-Standard Bracketing	0.07	doi.org/10.1021/acs.est.5b02402
$\delta^{66}Zn$	Zn	Coal Combustion	1.18‰	48.92	20.93	January, 2015	Active sampling	MC-ICP-MS	Sample-Standard Bracketing	0.07	doi.org/10.1021/acs.est.5b02402
$\delta^{66}Zn$	Zn	Coal Combustion	1.14‰	48.92	20.93	January, 2015	Active sampling	MC-ICP-MS	Sample-Standard Bracketing	0.07	doi.org/10.1021/acs.est.5b02402
$\delta^{66}Zn$	Zn	Coal Combustion	0.72‰	48.92	20.93	January, 2015	Active sampling	MC-ICP-MS	Sample-Standard Bracketing	0.07	doi.org/10.1021/acs.est.5b02402
$\delta^{66}Zn$	Zn	Coal Combustion	0.53‰	40.50	2.90	January, 2015	Active sampling	MC-ICP-MS	Sample-Standard Bracketing	0.07	doi.org/10.1021/acs.est.5b02402
$\delta^{66}Zn$	Zn	Coal Combustion	0.84‰	40.50	2.90	January, 2015	Active sampling	MC-ICP-MS	Sample-Standard Bracketing	0.07	doi.org/10.1021/acs.est.5b02402

$\delta^{66}Zn$	Zn	Coal Combustion	0.25‰	40.50	2.90	January, 2015	Active sampling	MC-ICP-MS	Sample-Standard Bracketing	0.07	doi.org/10.1021/acs.est.5b02402
$\delta^{66}Zn$	Zn	Coal Combustion	0.45‰	41.75	-85.67	January, 2010	Active sampling	MC-ICP-MS	Sample-Standard Bracketing	0.07	10.1021/es102439g
$\delta^{66}Zn$	Zn	Coal Combustion	0.47‰	41.75	-85.67	January, 2010	Active sampling	MC-ICP-MS	Sample-Standard Bracketing	0.07	10.1021/es102439g
$\delta^{66}Zn$	Zn	Coal Combustion	0.61‰	41.75	-85.67	January, 2010	Active sampling	MC-ICP-MS	Sample-Standard Bracketing	0.07	10.1021/es102439g
$\delta^{66}Zn$	Zn	Coal Combustion	0.39‰	37.98	-87.53	27 April, 2001	Active sampling	MC-ICP-MS	Sample-Standard Bracketing	0.07	10.1021/es102439g
$\delta^{66}Zn$	Zn	Coal Combustion	0.36‰	37.98	-87.53	27 April, 2001	Active sampling	MC-ICP-MS	Sample-Standard Bracketing	0.07	10.1021/es102439g
$\delta^{66}Zn$	Zn	Coal Combustion	0.45‰	40.42	-86.92	30 April, 2001	Active sampling	MC-ICP-MS	Sample-Standard Bracketing	0.07	10.1021/es102439g
$\delta^{66}Zn$	Zn	Coal Combustion	0.36‰	40.42	-86.92	30 April, 2001	Active sampling	MC-ICP-MS	Sample-Standard Bracketing	0.07	10.1021/es102439g
$\delta^{66}Zn$	Zn	Coal Combustion	0.54‰	40.42	-86.92	April, 2001	Active sampling	MC-ICP-MS	Sample-Standard Bracketing	0.07	10.1021/es102439g
$\delta^{66}Zn$	Zn	Coal Combustion	0.61‰	40.42	-86.92	April, 2001	Active sampling	MC-ICP-MS	Sample-Standard Bracketing	0.07	10.1021/es102439g
$\delta^{66}Zn$	Zn	Coal Combustion	-0.21‰	40.42	-86.92	April, 2001	Active sampling	MC-ICP-MS	Sample-Standard Bracketing	0.07	10.1021/es102439g
$\delta^{66}Zn$	Zn	Coal Combustion	0.58‰	40.42	-86.92	April, 2001	Active sampling	MC-ICP-MS	Sample-Standard Bracketing	0.07	10.1021/es102439g
$\delta^{66}Zn$	Zn	Coal Combustion	0.90‰	40.42	-86.92	April, 2001	Active sampling	MC-ICP-MS	Sample-Standard Bracketing	0.07	10.1021/es102439g
$\delta^{66}Zn$	Zn	Coal Combustion	-0.35‰	40.42	-86.92	April, 2001	Active sampling	MC-ICP-MS	Sample-Standard Bracketing	0.07	10.1021/es102439g
$\delta^{66}Zn$	Zn	Coal Combustion	-0.35‰	40.42	-86.92	April, 2001	Active sampling	MC-ICP-MS	Sample-Standard Bracketing	0.07	10.1021/es102439g

$\delta^{66}Zn$	Zn	Coal Combustion	-0.30‰	40.42	-86.92	April, 2001	Active sampling	MC-ICP-MS	Sample-Standard Bracketing	0.07	10.1021/es102439g
$\delta^{66}Zn$	Zn	Coal Combustion	-0.52‰	40.42	-86.92	April, 2001	Active sampling	MC-ICP-MS	Sample-Standard Bracketing	0.07	10.1021/es102439g
$\delta^{66}Zn$	Zn	Coal Combustion	-0.58‰	40.42	-86.92	April, 2001	Active sampling	MC-ICP-MS	Sample-Standard Bracketing	0.07	10.1021/es102439g
$\delta^{66}Zn$	Zn	Coal Combustion	-0.52‰	40.42	-86.92	April, 2001	Active sampling	MC-ICP-MS	Sample-Standard Bracketing	0.07	10.1021/es102439g
$\delta^{66}Zn$	Zn	Coal Combustion	0.47‰	40.42	-86.92	April, 2001	Active sampling	MC-ICP-MS	Sample-Standard Bracketing	0.07	10.1021/es102439g
$\delta^{66}Zn$	Zn	Coal Combustion	0.73‰	40.42	-86.92	April, 2001	Active sampling	MC-ICP-MS	Sample-Standard Bracketing	0.07	10.1021/es102439g
$\delta^{66}Zn$	Zn	Coal Combustion	0.80‰	40.42	-86.92	April, 2001	Active sampling	MC-ICP-MS	Sample-Standard Bracketing	0.07	10.1021/es102439g
$\delta^{66}Zn$	Zn	Coal Combustion	1.58‰	40.50	2.90	January, 2015	Active sampling	MC-ICP-MS	Sample-Standard Bracketing	0.07	doi.org/10.1021/acs.est.5b02402
$\delta^{66}Zn$	Zn	Coal Combustion	1.48‰	40.50	2.90	January, 2015	Active sampling	MC-ICP-MS	Sample-Standard Bracketing	0.07	doi.org/10.1021/acs.est.5b02402
$\delta^{66}Zn$	Zn	Coal Combustion	1.66‰	40.50	2.90	January, 2015	Active sampling	MC-ICP-MS	Sample-Standard Bracketing	0.07	doi.org/10.1021/acs.est.5b02402
$\delta^{66}Zn$	Zn	Coal Combustion	1.60‰	40.50	2.90	January, 2015	Active sampling	MC-ICP-MS	Sample-Standard Bracketing	0.07	doi.org/10.1021/acs.est.5b02402
$\delta^{66}Zn$	Zn	Coal Combustion	0.93‰	40.50	2.90	January, 2015	Active sampling	MC-ICP-MS	Sample-Standard Bracketing	0.07	doi.org/10.1021/acs.est.5b02402
$\delta^{66}Zn$	Zn	Coal Combustion	1.08‰	40.50	2.90	January, 2015	Active sampling	MC-ICP-MS	Sample-Standard Bracketing	0.07	doi.org/10.1021/acs.est.5b02402
$\delta^{66}Zn$	Zn	Natural Soil	-0.28‰	48.92	20.90	2010	Active sampling	MC-ICP-MS	Sample-Standard Bracketing	0.06	10.1016/j.gca.2010.08.044
$\delta^{66}Zn$	Zn	Natural Soil	0.52‰	37.42	78.50	2007-2009	Active sampling	MC-ICP-MS	Sample-Standard Bracketing	-	dx.doi.org/10.1016/j.talanta.2013.03.062

$\delta^{66}Zn$	Zn	Natural Soil	0.43‰	37.42	78.50	2007-2009	Active sampling	MC-ICP-MS	Sample-Standard Bracketing	-	dx.doi.org/10.1016/j.talanta.2013.03.062
$\delta^{66}Zn$	Zn	Natural Soil	0.37‰	37.42	78.50	2007-2009	Active sampling	MC-ICP-MS	Sample-Standard Bracketing	-	dx.doi.org/10.1016/j.talanta.2013.03.062
$\delta^{66}Zn$	Zn	Natural Soil	0.46‰	37.42	78.50	2007-2009	Active sampling	MC-ICP-MS	Sample-Standard Bracketing	-	dx.doi.org/10.1016/j.talanta.2013.03.062
$\delta^{66}Zn$	Zn	Natural Soil	0.67‰	37.42	78.50	2007-2009	Active sampling	MC-ICP-MS	Sample-Standard Bracketing	-	dx.doi.org/10.1016/j.talanta.2013.03.062
$\delta^{66}Zn$	Zn	Natural Soil	0.57‰	36.80	82.27	2007-2009	Active sampling	MC-ICP-MS	Sample-Standard Bracketing	-	dx.doi.org/10.1016/j.talanta.2013.03.062
$\delta^{66}Zn$	Zn	Natural Soil	0.43‰	36.80	82.27	2007-2009	Active sampling	MC-ICP-MS	Sample-Standard Bracketing	-	dx.doi.org/10.1016/j.talanta.2013.03.062
$\delta^{66}Zn$	Zn	Natural Soil	0.42‰	36.80	82.27	2007-2009	Active sampling	MC-ICP-MS	Sample-Standard Bracketing	-	dx.doi.org/10.1016/j.talanta.2013.03.062
$\delta^{66}Zn$	Zn	Natural Soil	0.64‰	36.80	82.27	2007-2009	Active sampling	MC-ICP-MS	Sample-Standard Bracketing	-	dx.doi.org/10.1016/j.talanta.2013.03.062
$\delta^{66}Zn$	Zn	Natural Soil	0.77‰	36.80	82.27	2007-2009	Active sampling	MC-ICP-MS	Sample-Standard Bracketing	-	dx.doi.org/10.1016/j.talanta.2013.03.062
$\delta^{66}Zn$	Zn	Natural Soil	0.72‰	12.61	-8.12	January, 2013	Active sampling	MC-ICP-MS	Sample-Standard Bracketing	-	dx.doi.org/10.1016/j.talanta.2013.03.062
$\delta^{66}Zn$	Zn	Natural Soil	0.75‰	36.80	82.27	January, 2020	Active sampling	MC-ICP-MS	Sample-Standard Bracketing	0.08	10.3389/feart.2020.00167
$\delta^{66}Zn$	Zn	Natural Soil	0.48‰	36.80	82.27	January, 2020	Active sampling	MC-ICP-MS	Sample-Standard Bracketing	0.08	10.3389/feart.2020.00167
$\delta^{66}Zn$	Zn	Natural Soil	0.52‰	36.80	82.27	January, 2020	Active sampling	MC-ICP-MS	Sample-Standard Bracketing	0.08	10.3389/feart.2020.00167
$\delta^{66}Zn$	Zn	Natural Soil	0.57‰	36.80	82.27	January, 2020	Active sampling	MC-ICP-MS	Sample-Standard Bracketing	0.08	10.3389/feart.2020.00167
$\delta^{66}Zn$	Zn	Natural Soil	0.52‰	36.80	82.27	January, 2020	Active sampling	MC-ICP-MS	Sample-Standard Bracketing	0.08	10.3389/feart.2020.00167

$\delta^{66}Zn$	Zn	Natural Soil	0.36‰	36.80	82.27	January, 2020	Active sampling	MC-ICP-MS	Sample-Standard Bracketing	0.08	10.3389/feart.2020.00167
$\delta^{66}Zn$	Zn	Natural Soil	0.43‰	36.80	82.27	January, 2020	Active sampling	MC-ICP-MS	Sample-Standard Bracketing	0.08	10.3389/feart.2020.00167
$\delta^{66}Zn$	Zn	Natural Soil	0.43‰	36.80	82.27	January, 2020	Active sampling	MC-ICP-MS	Sample-Standard Bracketing	0.08	10.3389/feart.2020.00167
$\delta^{66}Zn$	Zn	Natural Soil	0.50‰	36.80	82.27	January, 2020	Active sampling	MC-ICP-MS	Sample-Standard Bracketing	0.08	10.3389/feart.2020.00167
$\delta^{66}Zn$	Zn	Natural Soil	0.23‰	36.80	82.27	January, 2020	Active sampling	MC-ICP-MS	Sample-Standard Bracketing	0.08	10.3389/feart.2020.00167
$\delta^{66}Zn$	Zn	Natural Soil	0.37‰	36.80	82.27	January, 2020	Active sampling	MC-ICP-MS	Sample-Standard Bracketing	0.08	10.3389/feart.2020.00167
$\delta^{66}Zn$	Zn	Natural Soil	0.42‰	36.80	82.27	January, 2020	Active sampling	MC-ICP-MS	Sample-Standard Bracketing	0.08	10.3389/feart.2020.00167
$\delta^{66}Zn$	Zn	Natural Soil	0.56‰	36.80	82.27	January, 2020	Active sampling	MC-ICP-MS	Sample-Standard Bracketing	0.08	10.3389/feart.2020.00167
$\delta^{66}Zn$	Zn	Natural Soil	0.22‰	36.80	82.27	January, 2020	Active sampling	MC-ICP-MS	Sample-Standard Bracketing	0.08	10.3389/feart.2020.00167
$\delta^{66}Zn$	Zn	Natural Soil	0.46‰	36.80	82.27	January, 2020	Active sampling	MC-ICP-MS	Sample-Standard Bracketing	0.08	10.3389/feart.2020.00167
$\delta^{66}Zn$	Zn	Natural Soil	0.64‰	36.80	82.27	January, 2020	Active sampling	MC-ICP-MS	Sample-Standard Bracketing	0.08	10.3389/feart.2020.00167
$\delta^{66}Zn$	Zn	Natural Soil	0.57‰	36.80	82.27	January, 2020	Active sampling	MC-ICP-MS	Sample-Standard Bracketing	0.08	10.3389/feart.2020.00167
$\delta^{66}Zn$	Zn	Natural Soil	0.28‰	36.80	82.27	January, 2020	Active sampling	MC-ICP-MS	Sample-Standard Bracketing	0.08	10.3389/feart.2020.00167
$\delta^{66}Zn$	Zn	Natural Soil	0.67‰	36.80	82.27	January, 2020	Active sampling	MC-ICP-MS	Sample-Standard Bracketing	0.08	10.3389/feart.2020.00167
$\delta^{66}Zn$	Zn	Natural Soil	0.77‰	36.80	82.27	January, 2020	Active sampling	MC-ICP-MS	Sample-Standard Bracketing	0.08	10.3389/feart.2020.00167

$\delta^{66}Zn$	Zn	Natural Soil	0.62‰	43.73	107.45	January, 2020	Active sampling	MC-ICP-MS	Sample-Standard Bracketing	0.08	10.3389/feart.2020.00167
$\delta^{66}Zn$	Zn	Natural Soil	0.62‰	35.55	107.45	January, 2020	Active sampling	MC-ICP-MS	Sample-Standard Bracketing	0.08	10.3389/feart.2020.00167
$\delta^{66}Zn$	Zn	Natural Soil	0.53‰	35.55	107.45	January, 2020	Active sampling	MC-ICP-MS	Sample-Standard Bracketing	0.08	10.3389/feart.2020.00167
$\delta^{66}Zn$	Zn	Natural Soil	0.64‰	35.55	107.45	January, 2020	Active sampling	MC-ICP-MS	Sample-Standard Bracketing	0.08	10.3389/feart.2020.00167
$\delta^{66}Zn$	Zn	Natural Soil	0.61‰	35.55	107.45	January, 2020	Active sampling	MC-ICP-MS	Sample-Standard Bracketing	0.08	10.3389/feart.2020.00167
$\delta^{66}Zn$	Zn	Natural Soil	0.76‰	36.43	105.18	January, 2020	Active sampling	MC-ICP-MS	Sample-Standard Bracketing	0.08	10.3389/feart.2020.00167
$\delta^{66}Zn$	Zn	Natural Soil	0.78‰	36.43	105.18	January, 2020	Active sampling	MC-ICP-MS	Sample-Standard Bracketing	0.08	10.3389/feart.2020.00167
$\delta^{66}Zn$	Zn	Natural Soil	0.86‰	28.75	75.17	January, 2020	Active sampling	MC-ICP-MS	Sample-Standard Bracketing	0.08	10.3389/feart.2020.00167
$\delta^{66}Zn$	Zn	Natural Soil	0.70‰	28.75	75.17	January, 2020	Active sampling	MC-ICP-MS	Sample-Standard Bracketing	0.08	10.3389/feart.2020.00167
$\delta^{66}Zn$	Zn	Natural Soil	0.27‰	12.62	3.00	January, 2020	Active sampling	MC-ICP-MS	Sample-Standard Bracketing	0.08	10.3389/feart.2020.00167
$\delta^{66}Zn$	Zn	Natural Soil	0.60‰	12.62	3.00	January, 2020	Active sampling	MC-ICP-MS	Sample-Standard Bracketing	0.08	10.3389/feart.2020.00167
$\delta^{66}Zn$	Zn	Natural Soil	0.53‰	12.62	3.00	January, 2020	Active sampling	MC-ICP-MS	Sample-Standard Bracketing	0.08	10.3389/feart.2020.00167
$\delta^{66}Zn$	Zn	Natural Soil	0.52‰	12.62	3.00	January, 2020	Active sampling	MC-ICP-MS	Sample-Standard Bracketing	0.08	10.3389/feart.2020.00167
$\delta^{66}Zn$	Zn	Vehicle Exhausts	-0.14‰	-23.56	-46.72	September, 2006	Active sampling	MC-ICP-MS	Sample-Standard Bracketing	0.05	10.1021/ac8019587
$\delta^{66}Zn$	Zn	Vehicle Exhausts	0.04‰	49.10	6.17	July, 2002-March, 2003	Active sampling	MC-ICP-MS	Sample-Standard Bracketing	0.08	doi.org/10.1021/es0609654

$\delta^{66}Zn$	Zn	Vehicle Exhausts	0.10‰	49.10	6.17	July, 2002-March, 2003	Active sampling	MC-ICP-MS	Sample-Standard Bracketing	0.08	doi.org/10.1021/es0609654
$\delta^{66}Zn$	Zn	Vehicle Exhausts	-0.04‰	49.10	6.17	July, 2002-March, 2003	Active sampling	MC-ICP-MS	Sample-Standard Bracketing	0.08	doi.org/10.1021/es0609654
$\delta^{66}Zn$	Zn	Vehicle Exhausts	0.18‰	49.10	6.17	July, 2002-March, 2003	Active sampling	MC-ICP-MS	Sample-Standard Bracketing	0.08	doi.org/10.1021/es0609654
$\delta^{66}Zn$	Zn	Non-exhaust emissions-Brake Pad	0.44‰	51.50	0.18	January, 2017	Active sampling	MC-ICP-MS	Sample-Standard Bracketing	0.08	http://dx.doi.org/10.1016/j.atmosenv.2017.06.020
$\delta^{66}Zn$	Zn	Non-exhaust emissions-Brake Pad	0.49‰	51.50	0.18	January, 2017	Active sampling	MC-ICP-MS	Sample-Standard Bracketing	0.08	http://dx.doi.org/10.1016/j.atmosenv.2017.06.020
$\delta^{66}Zn$	Zn	Non-exhaust emissions-Brake Pad	-0.07‰	35.21	129.07	January, 2021	Active sampling	MC-ICP-MS	Sample-Standard Bracketing	-	https://doi.org/10.1016/j.envpol.2021.118339
$\delta^{66}Zn$	Zn	Non-exhaust emissions-Brake Pad	-0.08‰	35.21	129.07	January, 2021	Active sampling	MC-ICP-MS	Sample-Standard Bracketing	-	https://doi.org/10.1016/j.envpol.2021.118339
$\delta^{66}Zn$	Zn	Non-exhaust emissions-Brake Pad	-0.02‰	35.21	129.07	January, 2021	Active sampling	MC-ICP-MS	Sample-Standard Bracketing	-	https://doi.org/10.1016/j.envpol.2021.118339
$\delta^{66}Zn$	Zn	Non-exhaust emissions-Brake Pad	0.01‰	35.21	129.07	January, 2021	Active sampling	MC-ICP-MS	Sample-Standard Bracketing	-	https://doi.org/10.1016/j.envpol.2021.118339
$\delta^{66}Zn$	Zn	Non-exhaust emissions-Brake Pad	-0.03‰	35.21	129.07	January, 2021	Active sampling	MC-ICP-MS	Sample-Standard Bracketing	-	https://doi.org/10.1016/j.envpol.2021.118339
$\delta^{66}Zn$	Zn	Non-exhaust emissions-Brake Pad	-0.01‰	35.21	129.07	January, 2021	Active sampling	MC-ICP-MS	Sample-Standard Bracketing	-	https://doi.org/10.1016/j.envpol.2021.118339
$\delta^{66}Zn$	Zn	Non-exhaust emissions-Brake Pad	-0.06‰	35.21	129.07	January, 2021	Active sampling	MC-ICP-MS	Sample-Standard Bracketing	-	https://doi.org/10.1016/j.envpol.2021.118339
$\delta^{66}Zn$	Zn	Non-exhaust emissions-Brake Pad	-0.06‰	35.21	129.07	January, 2021	Active sampling	MC-ICP-MS	Sample-Standard Bracketing	-	https://doi.org/10.1016/j.envpol.2021.118339
$\delta^{66}Zn$	Zn	Non-exhaust emissions-Brake Pad	-0.03‰	35.21	129.07	January, 2021	Active sampling	MC-ICP-MS	Sample-Standard Bracketing	-	https://doi.org/10.1016/j.envpol.2021.118339
$\delta^{66}Zn$	Zn	Non-exhaust emissions-Brake Pad	0.01‰	35.21	129.07	January, 2021	Active sampling	MC-ICP-MS	Sample-Standard Bracketing	-	https://doi.org/10.1016/j.envpol.2021.118339

$\delta^{66}Zn$	Zn	Non-exhaust emissions-Brake Pad	0.03‰	35.21	129.07	January, 2021	Active sampling	MC-ICP-MS	Sample-Standard Bracketing	-	https://doi.org/10.1016/j.envpol.2021.118339
$\delta^{66}Zn$	Zn	Non-exhaust emissions-Brake Pad	-0.08‰	35.21	129.07	January, 2021	Active sampling	MC-ICP-MS	Sample-Standard Bracketing	-	https://doi.org/10.1016/j.envpol.2021.118339
$\delta^{66}Zn$	Zn	Non-exhaust emissions-Brake Pad	-0.09‰	35.21	129.07	January, 2021	Active sampling	MC-ICP-MS	Sample-Standard Bracketing	-	https://doi.org/10.1016/j.envpol.2021.118339
$\delta^{66}Zn$	Zn	Non-exhaust emissions-Brake Pad	0.04‰	47.05	-77.01	January, 2021	Active sampling	MC-ICP-MS	Sample-Standard Bracketing	-	https://doi.org/10.1016/j.envpol.2021.118339
$\delta^{66}Zn$	Zn	Non-exhaust emissions-Brake Pad	-0.07‰	47.05	-77.01	January, 2021	Active sampling	MC-ICP-MS	Sample-Standard Bracketing	-	https://doi.org/10.1016/j.envpol.2021.118339
$\delta^{66}Zn$	Zn	Non-exhaust emissions-Brake Pad	-0.03‰	52.51	13.03	January, 2021	Active sampling	MC-ICP-MS	Sample-Standard Bracketing	-	https://doi.org/10.1016/j.envpol.2021.118339
$\delta^{66}Zn$	Zn	Non-exhaust emissions-Brake Pad	-0.16‰	35.21	129.07	January, 2021	Active sampling	MC-ICP-MS	Sample-Standard Bracketing	-	https://doi.org/10.1016/j.envpol.2021.118339
$\delta^{66}Zn$	Zn	Non-exhaust emissions-Brake Pad	-0.19‰	35.21	129.07	January, 2021	Active sampling	MC-ICP-MS	Sample-Standard Bracketing	-	https://doi.org/10.1016/j.envpol.2021.118339
$\delta^{66}Zn$	Zn	Non-exhaust emissions-Brake Pad	0.15‰	35.21	129.07	January, 2021	Active sampling	MC-ICP-MS	Sample-Standard Bracketing	-	https://doi.org/10.1016/j.envpol.2021.118339
$\delta^{66}Zn$	Zn	Non-exhaust emissions-Brake Pad	0.16‰	35.21	129.07	January, 2021	Active sampling	MC-ICP-MS	Sample-Standard Bracketing	-	https://doi.org/10.1016/j.envpol.2021.118339
$\delta^{66}Zn$	Zn	Non-exhaust emissions-Road paint	0.36‰	51.52	0.20	8-28 July, 2010	Active sampling	MC-ICP-MS	Sample-Standard Bracketing	0.08	http://dx.doi.org/10.1016/j.atmosenv.2017.06.020
$\delta^{66}Zn$	Zn	Non-exhaust emissions-Road paint	0.67‰	51.52	0.15	8-28 July, 2010	Active sampling	MC-ICP-MS	Sample-Standard Bracketing	0.08	http://dx.doi.org/10.1016/j.atmosenv.2017.06.020
$\delta^{66}Zn$	Zn	Non-exhaust emissions-Road paint	0.73‰	51.52	0.15	8-28 July, 2010	Active sampling	MC-ICP-MS	Sample-Standard Bracketing	0.08	http://dx.doi.org/10.1016/j.atmosenv.2017.06.020
$\delta^{66}Zn$	Zn	Non-exhaust emissions-Road paint	0.87‰	51.52	0.15	8-28 July, 2010	Active sampling	MC-ICP-MS	Sample-Standard Bracketing	0.08	http://dx.doi.org/10.1016/j.atmosenv.2017.06.020
$\delta^{66}Zn$	Zn	Non-exhaust emissions-Road paint	0.30‰	51.52	0.20	8-28 July, 2010	Active sampling	MC-ICP-MS	Sample-Standard Bracketing	0.08	http://dx.doi.org/10.1016/j.atmosenv.2017.06.020

$\delta^{66}Zn$	Zn	Non-exhaust emissions-Road paint	0.00‰	35.21	129.07	January, 2021	Active sampling	MC-ICP-MS	Sample-Standard Bracketing	-	https://doi.org/10.1016/j.envpol.2021.118339
$\delta^{66}Zn$	Zn	Non-exhaust emissions-Road paint	0.01‰	35.21	129.07	January, 2021	Active sampling	MC-ICP-MS	Sample-Standard Bracketing	-	https://doi.org/10.1016/j.envpol.2021.118339
$\delta^{66}Zn$	Zn	Non-exhaust emissions-Road paint	0.00‰	35.21	129.07	January, 2021	Active sampling	MC-ICP-MS	Sample-Standard Bracketing	-	https://doi.org/10.1016/j.envpol.2021.118339
$\delta^{66}Zn$	Zn	Non-exhaust emissions-Road paint	-0.02‰	35.21	129.07	January, 2021	Active sampling	MC-ICP-MS	Sample-Standard Bracketing	-	https://doi.org/10.1016/j.envpol.2021.118339
$\delta^{66}Zn$	Zn	Non-exhaust emissions-Road paint	-0.01‰	35.21	129.07	January, 2021	Active sampling	MC-ICP-MS	Sample-Standard Bracketing	-	https://doi.org/10.1016/j.envpol.2021.118339
$\delta^{66}Zn$	Zn	Non-exhaust emissions-Road paint	0.01‰	35.21	129.07	January, 2021	Active sampling	MC-ICP-MS	Sample-Standard Bracketing	-	https://doi.org/10.1016/j.envpol.2021.118339
$\delta^{66}Zn$	Zn	Non-exhaust emissions-Road paint	-0.66‰	35.21	129.07	January, 2021	Active sampling	MC-ICP-MS	Sample-Standard Bracketing	-	https://doi.org/10.1016/j.envpol.2021.118339
$\delta^{66}Zn$	Zn	Non-exhaust emissions-Road paint	-0.46‰	35.21	129.07	January, 2021	Active sampling	MC-ICP-MS	Sample-Standard Bracketing	-	https://doi.org/10.1016/j.envpol.2021.118339
$\delta^{66}Zn$	Zn	Non-exhaust emissions-Road paint	0.11‰	35.21	129.07	January, 2021	Active sampling	MC-ICP-MS	Sample-Standard Bracketing	-	https://doi.org/10.1016/j.envpol.2021.118339
$\delta^{66}Zn$	Zn	Non-exhaust emissions-Road paint	-0.40‰	35.21	129.07	January, 2021	Active sampling	MC-ICP-MS	Sample-Standard Bracketing	-	https://doi.org/10.1016/j.envpol.2021.118339
$\delta^{66}Zn$	Zn	Non-exhaust emissions-Tire	0.41‰	40.50	-85.60	January, 2010	Active sampling	MC-ICP-MS	Sample-Standard Bracketing	0.07	10.1021/es102439g
$\delta^{66}Zn$	Zn	Non-exhaust emissions-Tire	-0.41‰	-23.56	-46.72	September, 2006	Active sampling	MC-ICP-MS	Sample-Standard Bracketing	0.05	10.1021/ac8019587
$\delta^{66}Zn$	Zn	Non-exhaust emissions-Tire	0.29‰	-23.56	-46.72	January, 2018	Active sampling	MC-ICP-MS	Sample-Standard Bracketing	-	doi.org/10.1016/j.scitotenv.2018.01.192
$\delta^{66}Zn$	Zn	Non-exhaust emissions-Tire	0.51‰	-23.56	-46.72	January, 2018	Active sampling	MC-ICP-MS	Sample-Standard Bracketing	-	doi.org/10.1016/j.scitotenv.2018.01.192
$\delta^{66}Zn$	Zn	Non-exhaust emissions-Tire	0.45‰	-23.56	-46.72	January, 2018	Active sampling	MC-ICP-MS	Sample-Standard Bracketing	-	doi.org/10.1016/j.scitotenv.2018.01.192

$\delta^{66}Zn$	Zn	Non-exhaust emissions-Tire	0.51‰	-23.56	-46.72	January, 2018	Active sampling	MC-ICP-MS	Sample-Standard Bracketing	-	doi.org/10.1016/j.scitotenv.2018.01.192
$\delta^{66}Zn$	Zn	Non-exhaust emissions-Tire	0.42‰	-23.56	-46.72	January, 2018	Active sampling	MC-ICP-MS	Sample-Standard Bracketing	-	doi.org/10.1016/j.scitotenv.2018.01.192
$\delta^{66}Zn$	Zn	Non-exhaust emissions-Tire	0.41‰	-23.56	-46.72	January, 2018	Active sampling	MC-ICP-MS	Sample-Standard Bracketing	-	doi.org/10.1016/j.scitotenv.2018.01.192
$\delta^{66}Zn$	Zn	Non-exhaust emissions-Tire	0.50‰	51.50	0.18	January, 2017	Active sampling	MC-ICP-MS	Sample-Standard Bracketing	0.08	http://dx.doi.org/10.1016/j.atmosenv.2017.06.020
$\delta^{66}Zn$	Zn	Non-exhaust emissions-Tire	0.48‰	51.50	0.18	January, 2017	Active sampling	MC-ICP-MS	Sample-Standard Bracketing	0.08	http://dx.doi.org/10.1016/j.atmosenv.2017.06.020
$\delta^{66}Zn$	Zn	Non-exhaust emissions-Tire	0.50‰	51.50	0.18	January, 2017	Active sampling	MC-ICP-MS	Sample-Standard Bracketing	0.08	http://dx.doi.org/10.1016/j.atmosenv.2017.06.020
$\delta^{66}Zn$	Zn	Non-exhaust emissions-Tire	-0.07‰	35.21	129.07	January, 2021	Active sampling	MC-ICP-MS	Sample-Standard Bracketing	-	https://doi.org/10.1016/j.envpol.2021.118339
$\delta^{66}Zn$	Zn	Non-exhaust emissions-Tire	-0.04‰	35.21	129.07	January, 2021	Active sampling	MC-ICP-MS	Sample-Standard Bracketing	-	https://doi.org/10.1016/j.envpol.2021.118339
$\delta^{66}Zn$	Zn	Ore-related Emissions	0.48‰	50.40	3.05	July, 2001-August, 2002	Active sampling	MC-ICP-MS	Sample-Standard Bracketing	0.03	10.1016/j.atmosenv.2008.11.030
$\delta^{66}Zn$	Zn	Ore-related Emissions	0.42‰	50.40	3.05	July, 2001-August, 2002	Active sampling	MC-ICP-MS	Sample-Standard Bracketing	0.03	10.1016/j.atmosenv.2008.11.030
$\delta^{66}Zn$	Zn	Ore-related Emissions	0.54‰	50.40	3.05	July, 2001-August, 2002	Active sampling	MC-ICP-MS	Sample-Standard Bracketing	0.03	10.1016/j.atmosenv.2008.11.030
$\delta^{66}Zn$	Zn	Ore-related Emissions	0.30‰	50.40	3.05	July, 2001-August, 2002	Active sampling	MC-ICP-MS	Sample-Standard Bracketing	0.03	10.1016/j.atmosenv.2008.11.030
$\delta^{66}Zn$	Zn	Ore-related Emissions	0.25‰	50.40	3.05	July, 2001-August, 2002	Active sampling	MC-ICP-MS	Sample-Standard Bracketing	0.03	10.1016/j.atmosenv.2008.11.030
$\delta^{66}Zn$	Zn	Ore-related Emissions	0.25‰	50.40	3.05	July, 2001-August, 2002	Active sampling	MC-ICP-MS	Sample-Standard Bracketing	0.03	10.1016/j.atmosenv.2008.11.030
$\delta^{66}Zn$	Zn	Ore-related Emissions	0.25‰	50.40	3.05	July, 2001-August, 2002	Active sampling	MC-ICP-MS	Sample-Standard Bracketing	0.03	10.1016/j.atmosenv.2008.11.030

$\delta^{66}Zn$	Zn	Ore-related Emissions	-0.05‰	50.40	3.05	July, 2001-August, 2002	Active sampling	MC-ICP-MS	Sample-Standard Bracketing	0.03	10.1016/j.atmosenv.2008.11.030
$\delta^{66}Zn$	Zn	Ore-related Emissions	-0.03‰	50.40	3.05	July, 2001-August, 2002	Active sampling	MC-ICP-MS	Sample-Standard Bracketing	0.03	10.1016/j.atmosenv.2008.11.030
$\delta^{66}Zn$	Zn	Ore-related Emissions	0.41‰	50.40	3.05	July, 2001-August, 2002	Active sampling	MC-ICP-MS	Sample-Standard Bracketing	0.03	10.1016/j.atmosenv.2008.11.030
$\delta^{66}Zn$	Zn	Ore-related Emissions	0.40‰	50.40	3.05	July, 2001-August, 2002	Active sampling	MC-ICP-MS	Sample-Standard Bracketing	0.03	10.1016/j.atmosenv.2008.11.030
$\delta^{66}Zn$	Zn	Ore-related Emissions	0.37‰	50.40	3.05	July, 2001-August, 2002	Active sampling	MC-ICP-MS	Sample-Standard Bracketing	0.03	10.1016/j.atmosenv.2008.11.030
$\delta^{66}Zn$	Zn	Ore-related Emissions	0.37‰	50.40	3.05	July, 2001-August, 2002	Active sampling	MC-ICP-MS	Sample-Standard Bracketing	0.03	10.1016/j.atmosenv.2008.11.030
$\delta^{66}Zn$	Zn	Ore-related Emissions	0.53‰	50.40	3.05	July, 2001-August, 2002	Active sampling	MC-ICP-MS	Sample-Standard Bracketing	0.03	10.1016/j.atmosenv.2008.11.030
$\delta^{66}Zn$	Zn	Ore-related Emissions	0.42‰	50.40	3.05	July, 2001-August, 2002	Active sampling	MC-ICP-MS	Sample-Standard Bracketing	0.03	10.1016/j.atmosenv.2008.11.030
$\delta^{66}Zn$	Zn	Ore-related Emissions	0.44‰	50.40	3.05	July, 2001-August, 2002	Active sampling	MC-ICP-MS	Sample-Standard Bracketing	0.03	10.1016/j.atmosenv.2008.11.030
$\delta^{66}Zn$	Zn	Ore-related Emissions	-0.34‰	50.40	3.05	July, 2001-August, 2002	Active sampling	MC-ICP-MS	Sample-Standard Bracketing	0.03	10.1016/j.atmosenv.2008.11.030
$\delta^{66}Zn$	Zn	Ore-related Emissions	-0.31‰	50.40	3.05	July, 2001-August, 2002	Active sampling	MC-ICP-MS	Sample-Standard Bracketing	0.03	10.1016/j.atmosenv.2008.11.030
$\delta^{66}Zn$	Zn	Ore-related Emissions	-0.41‰	50.40	3.05	July, 2001-August, 2002	Active sampling	MC-ICP-MS	Sample-Standard Bracketing	0.03	10.1016/j.atmosenv.2008.11.030
$\delta^{66}Zn$	Zn	Ore-related Emissions	-0.35‰	50.40	3.05	July, 2001-August, 2002	Active sampling	MC-ICP-MS	Sample-Standard Bracketing	0.03	10.1016/j.atmosenv.2008.11.030
$\delta^{66}Zn$	Zn	Ore-related Emissions	0.18‰	-23.56	-46.72	September, 2006	Active sampling	MC-ICP-MS	Sample-Standard Bracketing	0.05	10.1021/ac8019587
$\delta^{66}Zn$	Zn	Ore-related Emissions	0.28‰	-23.56	-46.72	September, 2006	Active sampling	MC-ICP-MS	Sample-Standard Bracketing	0.05	10.1021/ac8019587

$\delta^{66}Zn$	Zn	Ore-related Emissions	0.27‰	-23.56	-46.72	September, 2006	Active sampling	MC-ICP-MS	Sample-Standard Bracketing	0.05	10.1021/ac8019587
$\delta^{66}Zn$	Zn	Waste Incinerator	0.07‰	49.12	6.18	January and July, 2003	Active sampling	MC-ICP-MS	Sample-Standard Bracketing	0.08	doi.org/10.1021/es0609654
$\delta^{66}Zn$	Zn	Waste Incinerator	0.19‰	49.12	6.18	January and July, 2003	Active sampling	MC-ICP-MS	Sample-Standard Bracketing	0.08	doi.org/10.1021/es0609654
$\delta^{66}Zn$	Zn	Waste Incinerator	0.12‰	49.12	6.18	January and July, 2003	Active sampling	MC-ICP-MS	Sample-Standard Bracketing	0.08	doi.org/10.1021/es0609654

4. References for SI

1. Pan, Y. P. *et al.* Fossil fuel combustion-related emissions dominate atmospheric ammonia sources during severe haze episodes: Evidence from ^{15}N-stable isotope in size-resolved aerosol ammonium. *Environ. Sci. Technol.* **50**, 8049-8056 (2016).
2. Zhang, Y. Y. *et al.* Persistent nonagricultural and periodic agricultural emissions dominate sources of ammonia in urban Beijing: Evidence from ^{15}N stable isotope in vertical profiles. *Environ. Sci. Technol.* **54**, 102-109 (2020).
3. Chen, Z. L. *et al.* Significant contributions of combustion-related sources to ammonia emissions. *Nat. Commun.* **13,** 7710 (2022).
4. Zong, Z. *et al.* First assessment of NO_x sources at a regional background site in north China using isotopic analysis linked with modeling. *Environ. Sci. Technol.* **51**, 5923-5931 (2017).
5. Alexander, B. *et al.* Quantifying atmospheric nitrate formation pathways based on a global model of the oxygen isotopic composition (Δ^{17}O) of atmospheric nitrate. *Atmos. Chem. Phys.* **9**, 5043-5056 (2009).
6. Zong, Z. *et al.* Dual-modelling-based source apportionment of NO_x in five Chinese megacities: Providing the isotopic footprint from 2013 to 2014. *Environ. Int.* **137**, 105592 (2020).
7. Walters, W. W. & Michalski, G. Theoretical calculation of nitrogen isotope equilibrium exchange fractionation factors for various NO_y molecules. *Geochim. Cosmochim. Ac.* **164**, 284-297 (2015).
8. Walters, W. W. & Michalski, G. Theoretical calculation of oxygen equilibrium isotope fractionation factors involving various NO_y molecules, OH, and H_2O and its implications for isotope variations in atmospheric nitrate. *Geochim. Cosmochim. Ac* **191**, 89-101 (2016).
9. Lin, Y. -C., Yu, M. Y., Xie, F., & Zhang, Y. L. Anthropogenic emission sources of sulfate aerosols in Hangzhou, East China: insights from isotope techniques with consideration of fractionation effects between gas-to-particle transformations. *Environ. Sci. Technol.* **56**, 3905-3914 (2022).
10. Fan, M.-Y. *et al.* Roles of sulfur oxidation pathways in the variability in stable sulfur isotopic composition of sulfate aerosols at an urban site in Beijing, China. *Environ. Sci. Technol. Lett.* **7**, 883-888 (2020).
11. Zhao, H. Y. Z. *et al.* Measurement report: source apportionment of carbonaceous aerosol using dual-carbon isotopes (^{13}C and ^{14}C) and levoglucosan in three northern Chinese cities during 2018–2019. *Atmos. Chem. Phys.* **22**, 6255-6274 (2022).
12. Vlachou, A. *et al.* Advanced source apportionment of carbonaceous aerosols by coupling offline AMS and radiocarbon size-segregated measurements over a nearly 2-year period. *Atmos. Chem. Phys.* **18**, 6187-6206 (2018).
13. Hou, S. Q. *et al.* Source apportionment of carbonaceous aerosols in Beijing with radiocarbon and organic tracers: insight into the differences between urban and rural sites. *Atmos. Chem. Phys.* **21**, 8273-8292 (2021).
14. Levin, I. *et al.* Observations and modelling of the global distribution and long-term trend of atmospheric $^{14}CO_2$. *Tellus B.* **62**, 26-46 (2010).
15. Ni, H. *et al*. Measurement report: dual-carbon isotopic characterization of carbonaceous aerosol reveals different primary and secondary sources in Beijing and Xi'an during severe haze events. *Atmos. Chem. Phys.* **20**, 16041-16053 (2020).
16. Wang, J., Lu, N., & Fu, B. Inter-comparison of stable isotope mixing models for determining plant water source partitioning. *Sci. Total Environ.*, **666**, 685-693 (2019).
17. Yao, P. *et al.* ^{13}C signatures of aerosol organic and elemental carbon from major combustion sources in China compared to worldwide estimates. *Sci. Total Environ.* **810**, 151284 (2022).
18. Kirillova, E. N. *et al.* ^{13}C- and ^{14}C-based study of sources and atmospheric processing of water-soluble organic carbon (WSOC) in South Asian aerosols. *J. Geophys. Res. Atmospheres* **118**, 614-626 (2013).
19. Shen, C. D. *et al.* ^{14}C and ^{10}Be in dust deposited during the storm of 16-17 April 2006 in Beijing. *Radiocarbon* **55**, 1790-1800 (2013).
20. Gómez, V. *et al.* ^{14}C content in aerosols in Mexico City. *Nucl. Instrum. Methods Phys. Res. Sect. B Beam Interact. Mater. At.* **371**, 365-369 (2016).
21. Sun, X. *et al.*. ^{14}C-Based source assessment of carbonaceous aerosols at a rural site. *Atmos. Environ.* **50**, 36-40 (2012).
22. Andersson, A. *et al.* ^{14}C-Based source assessment of soot aerosols in Stockholm and the Swedish EMEP-Aspvreten regional background site. *Atmos. Environ.* **45**, 215-222 (2011).
23. Chen, Y. J. *et al*. Stable carbon isotope of black carbon from typical emission sources in China. *Environ. Sci.* **33**, 673-678 (2012). In Chinese with English abstract
24. Liu, G. *et al*. The isotopic composition of organic carbon in $PM_{2.5}$ in Hangzhou. *Chinese Sci. Bull.* 1935-

1937 (2007). In Chinese with English abstract

25. He, Y. X. *et al.* Emission characteristics of particulate emitted bymotor vehicles in Nanjing based on $PM_{2.5}$ sampling in tunnel. *Acta Scient. Circumst.* **41**, 4430-4438 (2021). In Chinese with English abstract
26. Zhou, R. W. *et al.* Pollution characteristics and formation mechanisms of dicarboxylic acids and related SOA in Jinan based on stable carbon isotope. *Acta Scient. Circumst.* **41**, 863-873 (2021). In Chinese with English abstract
27. Huang, J. *et al.* Composition and seasonal variations of carbon isotopes in aerosols of Lhasa, Tibet. *Environ. Sci.* **31**, 1139-1145 (2010). In Chinese with English abstract
28. Zhou, Y. M. *et al.* Chemical constitution and carbon isotopic compositions of $PM_{2.5}$ in the northern suburb of Nanjing in spring. *Environ. Sci.* **39**, 4439-4445 (2018). In Chinese with English abstract
29. Wu, M. L. *et al.* Stable carbon isotopic composition in $PM_{2.1}$ in Nanjing region. *Environ. Sci.* **34**, 3727-3732 (2013). In Chinese with English abstract
30. Shi, L. *et al.* Investigations on sulfur and carbon isotopic compositions of potential polluted sources in atmospheric $PM_{2.5}$ in Nanjing region. *Environ. Sci.* **37**, 22-27 (2016). In Chinese with English abstract
31. Bai, H. L. *et al.* Carbon isotope compositions and source apportionments of PAHs in PM_{10} of Taiyuan City during heating period. *China Environ. Sci.* **34**, 7-13 (2014). In Chinese with English abstract
32. Zhang, J. Q. *et al.* Isotopic compositions of carbon in PM_{10} and its emission sources in Taiyuan. *China Environ. Sci.* **32**, 968-972 (2012). In Chinese with English abstract
33. Huang, R. J. *et al.* Carbon isotope composition and source analysis of element carbon aerosol in winter of Xi'an. *Bull. Miner. Petrol. Geochem*. **38**, 1073-1080 (2019). In Chinese with English abstract
34. Zeng, Z. Q. *et al.* Stable carbon isotope composition in marine aerosols over the western North Pacific in winter. *Acta Scient. Circumst.* **40**, 2384-2390 (2020). In Chinese with English abstract
35. Yan, G. X. *et al.* Analysis of stable carbon isotope characteristics of $PM_{2.5}$ in summer and winter season in Xinxiang City. *Environ. Sci.* **41**, 82-89 (2020). In Chinese with English abstract
36. Xiao, Y. N. *et al.* Composition characteristics of chemical and stable carbon isotopes in $PM_{2.5}$ of Yulong Snow Mountain. *China Environ. Sci.* **42**, 2034-2040 (2022). In Chinese with English abstract
37. Kaul, D. S. *et al.* A novel tandem of thermal desorption carbon analyzer and off-axis integrated cavity output spectroscopy for aerosol stable carbon isotope ratio measurement. *Aerosol Air Qual. Res.* **16**, 1345-1355 (2016).
38. Weber, R. J. *et al.* A study of secondary organic aerosol formation in the anthropogenic-influenced southeastern United States. *J. Geophys. Res. Atmospheres* **112**, (2007).
39. Vodička, P. *et al.* A year-round observation of $\delta^{13}C$ of dicarboxylic acids and related compounds in fine aerosols: Implications from Central European background site. *Chemosphere* **337**, 139393 (2023).
40. Lewis, C. W. *et al.* Absence of ^{14}C in $PM_{2.5}$ emissions from gasohol combustion in small engines. *Aerosol Sci. Technol.* **40**, 657-663 (2006).
41. Weissenbök, R. H. *et al.* Accelerator mass spectrometry analysis of non-soluble carbon in aerosol particles from high alpine snow (Mt. Sonnblich, Austria). *Radiocarbon* **42**, 285-294 (2000).
42. Vlachou, A. *et al.* Advanced source apportionment of carbonaceous aerosols by coupling offline AMS and radiocarbon size-segregated measurements over a nearly 2-year period. *Atmospheric Chem. Phys.* **18**, 6187-6206 (2018).
43. Garbaras, A. *et al.* Aerosol source (biomass, traffic and coal emission) apportionment in Lithuania using stable carbon and radiocarbon analysis. *Isotopes Environ. Health Stud.* **54**, 463-474 (2018).
44. Bikkina, S. *et al.* Air quality in megacity Delhi affected by countryside biomass burning. *Nat. Sustain.* **2**, 200-205 (2019).
45. Solís, C. *et al.* AMS ^{14}C and chemical composition of atmospheric aerosols from Mexico City. *Radiocarbon* **59**, 321-332 (2017).
46. Solís, C. *et al.* AMS-C14 analysis of graphite obtained with an Automated Graphitization Equipment (AGE III) from aerosol collected on quartz filters. *Nucl. Instrum. Methods Phys. Res. Sect. B Beam Interact. Mater. At.* **361**, 419-422 (2015).
47. Rauber, M. *et al.* An optimised organic carbon/elemental carbon (OC/EC) fraction separation method for radiocarbon source apportionment applied to low-loaded Arctic aerosol filters. *Atmospheric Meas. Tech.* **16**, 825-844 (2023).
48. Buchholz, B. A. *et al.* Anomalous elevated radiocarbon measurements of $PM_{2.5}$. *Nucl. Instrum. Methods Phys. Res. Sect. B Beam Interact. Mater. At.* **294**, 631-635 (2013).
49. Heal, M. R. *et al.* Application of ^{14}C analyses to source apportionment of carbonaceous $PM_{2.5}$ in the UK. *Atmos. Environ.* **45**, 2341-2348 (2011).

50. Zimnoch, M. *et al.* Application of natural carbon isotopes for emission source apportionment of carbonaceous particulate matter in urban atmosphere: a case study from Krakow, southern Poland. *Sustainability* **12**, 5777 (2020).
51. Yoon, S. *et al.* Apportioned primary and secondary organic aerosol during pollution events of DISCOVER-AQ Houston. *Atmos. Environ.* **244**, 117954 (2021).
52. Kalashnikova, D. A. *et al.* Approbation of a procedure for determining the isotopic composition of individual carbon fractions in an atmospheric aerosol by isotope ratio mass spectrometry. *J. Anal. Chem.* **78**, 1055-1061 (2023).
53. Maia, P. D. *et al.* Assessment of atmospheric particulate matter (PM_{10}) in Central Brazil: Chemical and morphological aspects. *Atmospheric Pollut. Res.* **13**, 101362 (2022).
54. Attri, P. *et al.* Atmospheric aerosol chemistry and source apportionment of PM_{10} using stable carbon isotopes and PMF modelling during fireworks over Hyderabad, southern India. *Heliyon* **10**, (2024).
55. Kirago, L. *et al.* Atmospheric black carbon loadings and sources over eastern sub-saharan Africa are governed by the regional savanna fires. *Environ. Sci. Technol.* **56**, 15460-15469 (2022).
56. Ren, P. *et al.* Atmospheric particles are major sources of aged anthropogenic organic carbon in Marginal Seas. *Environ. Sci. Technol.* **56**, 14198-14207 (2022).
57. Khundadze, N. *et al.* Benchmarking source specific isotopic ratios of levoglucosan to better constrain the contribution of domestic heating to the air pollution. *Atmos. Environ.* **268**, 118842 (2022).
58. Bennett, M. *et al.* Biodiesel effects on particulate radiocarbon (^{14}C) emissions from a diesel engine. *J. Aerosol Sci.* **39**, 667-678 (2008).
59. Yoon, S. *et al.* Biomass and fossil fuel combustion contributions to elemental carbon across the San Francisco Bay Area. *Atmos. Environ.* **195**, 229-242 (2018).
60. Jiang, H. *et al.* Biomass burning organic aerosols significantly influence the light absorption properties of polarity-dependent organic compounds in the Pearl River Delta Region, China. *Environ. Int.* **144**, 106079 (2020).
61. Nizam, S. *et al.* Biomass-derived provenance dominates glacial surface organic carbon in the western Himalaya. *Environ. Sci. Technol.* **54**, 8612-8621 (2020).
62. Mouteva, G. O. *et al.* Black carbon aerosol dynamics and isotopic composition in Alaska linked with boreal fire emissions and depth of burn in organic soils. *Glob. Biogeochem. Cycles* **29**, 1977-2000 (2015).
63. Budhavant, K. *et al.* Black carbon aerosols over Indian Ocean have unique source fingerprint and optical characteristics during monsoon season. *Proc. Natl. Acad. Sci.* **120**, e2210005120 (2023).
64. Kirago, L. *et al*. Black carbon emissions from traffic contribute substantially to air pollution in Nairobi, Kenya. *Commun. Earth Environ.* **3**, 1-8 (2022).
65. Gustafsson, Ö. *et al.* Brown clouds over South Asia: biomass or fossil fuel combustion? *Science* **323**, 495-498 (2009).
66. Stojanowska, A. *et al.* Can abies alba needles be used as bio-passive samplers to assess air quality? *Aerosol Air Qual. Res.* **21**, 210097 (2021).
67. Górka, M. *et al*. Carbon isotope compositions and TC/OC/EC levels in atmospheric PM_{10} from Lower Silesia (SW Poland): Spatial variations, seasonality, sources and implications. *Atmospheric Pollut. Res.* **11**, 1099-1114 (2020).
68. Bai, H. *et al.* Carbon isotope seasonal characteristics of fine carbonaceous aerosol in Jinzhong City, Shanxi Province, China. *Atmos. Environ.* **246**, 118164 (2021).
69. Bikkina, S. *et al.* Carbon isotope-constrained seasonality of carbonaceous aerosol sources from an urban location (Kanpur) in the Indo-Gangetic Plain. *J. Geophys. Res. Atmospheres* **122**, 4903-4923 (2017).
70. Fang, J. *et al.* Carbon isotopic composition of fatty acids in the marine aerosols from the Western North Pacific: implication for the source and atmospheric transport. *Environ. Sci. Technol.* **36**, 2598-2604 (2002).
71. Minoura, H. *et al*. Carbonaceous aerosol and its characteristics observed in Tokyo and south Kanto region. *Atmos. Environ.* **61**, 605-613 (2012).
72. Geron, C. Carbonaceous aerosol over a *Pinus taeda* forest in Central North Carolina, USA. *Atmos. Environ.* **43**, 959-969 (2009).
73. Martinsson, J. *et al.* Carbonaceous aerosol source apportionment using the Aethalometer model - evaluation by radiocarbon and levoglucosan analysis at a rural background site in southern Sweden. *Atmospheric Chem. Phys.* **17**, 4265-4281 (2017).
74. Chen, P. *et al.* Carbonaceous aerosol transport from the Indo-Gangetic Plain to the Himalayas: Carbon isotope evidence and light absorption characteristics. *Geosci. Front.* **14**, 101516 (2023).
75. Cachier, H. *et al*. Carbonaceous aerosols from different tropical biomass burning sources. *Nature* **340**, 371-

373 (1989).

76. Aggarwal, S. G. & Kawamura, K. Carbonaceous and inorganic composition in long-range transported aerosols over northern Japan: Implication for aging of water-soluble organic fraction. *Atmos. Environ.* **43**, 2532-2540 (2009).
77. Morera-Gómez, Y. *et al.* Carbonaceous Fractions Contents and Carbon Stable Isotope Compositions of Aerosols Collected in the Atmosphere of Montreal (Canada): Seasonality, Sources, and Implications. *Front. Environ. Sci.* **9**, (2021).
78. Niu, Z. *et al.* Characteristics and source apportionment of particulate carbon in precipitation based on dual-carbon isotopes (^{13}C and ^{14}C) in Xi'an, China. *Environ. Pollut.* **299**, 118908 (2022).
79. Cao, J. J. *et al.* Characteristics and sources of carbonaceous aerosols from Shanghai, China. *Atmospheric Chem. Phys.* **13**, 803-817 (2013).
80. Wozniak, A. S. *et al.* Characteristics of water-soluble organic carbon associated with aerosol particles in the eastern United States. *Atmos. Environ.* **46**, 181-188 (2012).
81. Mu, L. *et al.* Characterization and emission factors of carbonaceous aerosols originating from coke production in China. *Environ. Pollut.* **268**, 115768 (2021).
82. Devaprasad, M. *et al.* Characterization of paddy-residue burning derived carbonaceous aerosols using dual carbon isotopes. *Sci. Total Environ.* **864**, 161044 (2023).
83. Ulevicius, V. *et al.* Characterization of pollution events in the East Baltic region affected by regional biomass fire emissions. *Atmospheric Res.* **98**, 190-200 (2010).
84. Romano, S. *et al.* Characterization of the $PM_{2.5}$ aerosol fraction monitored at a suburban site in south-eastern Italy by integrating isotopic techniques and ion beam analysis. *Front. Environ. Sci.* **10**, (2022).
85. Chalbot, M. C. G. *et al.* Characterization of water-soluble organic matter in urban aerosol by 1H-NMR spectroscopy. *Atmos. Environ.* **128**, 235-245 (2016).
86. Yu, K. *et al.* Characterizing and sourcing ambient $PM_{2.5}$ over key emission regions in China III: Carbon isotope based source apportionment of black carbon. *Atmos. Environ.* **177**, 12-17 (2018).
87. Bandowe, B. A. M. *et al.* Chemical (C, N, S, black carbon, soot and char) and stable carbon isotope composition of street dusts from a major West African metropolis: Implications for source apportionment and exposure. *Sci. Total Environ.* **655**, 1468-1478 (2019).
88. Song, J. *et al*. Chemical and Isotopic Composition of Humic-Like Substances (HULIS) in Ambient Aerosols in Guangzhou, South China. *Aerosol Sci. Technol.* **46**, 533-546 (2012).
89. Dai, S. *et al.* Chemical and stable carbon isotopic composition of $PM_{2.5}$ from on-road vehicle emissions in the PRD region and implications for vehicle emission control policy. *Atmospheric Chem. Phys.* **15**, 3097-3108 (2015).
90. Guillon, A. *et al.* Chemical characterization and stable carbon isotopic composition of particulate Polycyclic Aromatic Hydrocarbons issued from combustion of 10 Mediterranean woods. *Atmospheric Chem. Phys.* **13**, 2703-2719 (2013).
91. Nishikawa, M. *et al.* Chemical composition of urban airborne particulate matter in Ulaanbaatar. *Atmos. Environ.* **45**, 5710-5715 (2011).
92. Miyazaki, Y. *et al.* Chemical transfer of dissolved organic matter from surface seawater to sea spray water-soluble organic aerosol in the marine atmosphere. *Sci. Rep.* **8**, 14861 (2018).
93. Wang, X. *et al.* Combining positive matrix factorization and radiocarbon measurements for source apportionment of $PM_{2.5}$ from a national background site in North China. *Sci. Rep.* **7**, 10648 (2017).
94. Zhao, H. *et al.* Comparing sources of carbonaceous aerosols during haze and nonhaze periods in two northern Chinese cities. *J. Environ. Manage.* **346**, 119024 (2023).
95. Yamamoto, N. *et al.* Comparison of carbonaceous aerosols in Tokyo before and after Implementation of diesel exhaust restrictions. *Environ. Sci. Technol.* **41**, 6357-6362 (2007).
96. Samek, L. *et al.* Comparison of PM_{10} sources at traffic and urban background sites based on elemental, chemical and isotopic composition: case study from Krakow, Southern Poland. *Atmosphere* **12**, 1364 (2021).
97. Wu, Y. *et al.* Composition and sources of aerosol organic matter in a highly anthropogenic influenced semi-enclosed bay: Insights from excitation-emission matrix spectroscopy and isotopic evidence. *Atmospheric Res.* **241**, 104958 (2020).
98. Glasius, M. *et al.* Composition and sources of carbonaceous aerosols in Northern Europe during winter. *Atmos. Environ.* **173**, 127-141 (2018).
99. Eglinton, T. I. *et al.* Composition, age, and provenance of organic matter in NW African dust over the Atlantic Ocean. *Geochem. Geophys. Geosystems* **3**, 1-27 (2002).
100. Kumata, H. *et al.* Compound class specific ^{14}C analysis of polycyclic aromatic hydrocarbons associated with

PM_{10} and $PM_{1.1}$ aerosols from residential areas of suburban Tokyo. *Environ. Sci. Technol.* **40**, 3474-3480 (2006).

101. Matsumoto, K. *et al*. Compound specific radiocarbon and $\delta^{13}C$ measurements of fatty acids in a continental aerosol sample. *Geophys. Res. Lett.* **28**, 4587-4590 (2001).
102. Simoneit, B. R. T. Compound-specific carbon isotope analyses of individual long-chain alkanes and alkanoic acids in Harmattan aerosols. *Atmos. Environ.* **31**, 2225-2233 (1997).
103. Yi, X. *et al.* Compound-specific radiocarbon analysis of benzene polycarboxylic acids for source apportionment of polyaromatic organic matter in ambient aerosols. *Atmos. Environ.* **307**, 119832 (2023).
104. Xu, B. *et al.* Compound-specific radiocarbon analysis of low molecular weight dicarboxylic acids in ambient aerosols using preparative gas chromatography: method development. *Environ. Sci. Technol. Lett.* **8**, 135-141 (2021).
105. Ren, L. *et al.* Compound-specific stable carbon isotope ratios of terrestrial biomarkers in urban aerosols from Beijing, China. *ACS Earth Space Chem.* **3**, 1896-1904 (2019).
106. Nguyen, D. L. *et al.* Comprehensive $PM_{2.5}$ organic molecular composition and stable carbon isotope ratios at Sonla, Vietnam: fingerprint of biomass burning components. *Aerosol Air Qual. Res.* **16**, 2618-2634 (2016).
107. Wang, J. *et al.* Concentrations and stable carbon isotope compositions of oxalic acid and related SOA in Beijing before, during, and after the 2014 APEC. *Atmospheric Chem. Phys.* **17**, 981-992 (2017).
108. Turekian, V. C. *et al.* Concentrations, isotopic compositions, and sources of size-resolved, particulate organic carbon and oxalate in near-surface marine air at Bermuda during spring. *J. Geophys. Res. Atmospheres* **108**, (2003).
109. Piletic, I. R. *et al.* Constraining carbonaceous aerosol sources in a receptor model by including ^{14}C data with redox species, organic tracers, and elemental/organic carbon measurements. *Atmos. Environ.* **80**, 216-225 (2013).
110. Sheesley, R. J. *et al.* Constraints on primary and secondary particulate carbon sources using chemical tracer and ^{14}C methods during CalNex-Bakersfield. *Atmos. Environ.* **166**, 204-214 (2017).
111. Ding, X. *et al.* Contemporary or fossil origin: split of estimated secondary organic carbon in the Southeastern United States. *Environ. Sci. Technol.* **42**, 9122-9128 (2008).
112. Moffett, C. E. *et al.* Contemporary sources dominate carbonaceous aerosol on the North Slope of Alaska. *Sci. Total Environ.* **831**, 154641 (2022).
113. Miyazaki, Y. *et al.* Contribution of dissolved organic matter to submicron water-soluble organic aerosols in the marine boundary layer over the eastern equatorial Pacific. *Atmospheric Chem. Phys.* **16**, 7695-7707 (2016).
114. Kawamura, K. *et al.* Contributions of modern and dead organic carbon to individual fatty acid homologues in spring aerosols collected from northern Japan. *J. Geophys. Res. Atmospheres* **115**, (2010).
115. Major, I. *et al.* Detailed carbon isotope study of $PM_{2.5}$ aerosols at urban background, suburban background and regional background sites in Hungary. *Atmosphere* **13**, 716 (2022).
116. Górka, M. *et al.* Determination of multiple organic matter sources in aerosol PM_{10} from Wrocław, Poland using molecular and stable carbon isotope compositions. *Atmos. Environ.* **89**, 739-748 (2014).
117. Fisseha, R. *et al.* Determination of primary and secondary sources of organic acids and carbonaceous aerosols using stable carbon isotopes. *Atmos. Environ.* **43**, 431-437 (2009).
118. Kawamura, K. & Watanabe, T. Determination of stable carbon isotopic compositions of low molecular weight dicarboxylic acids and ketocarboxylic acids in atmospheric aerosol and snow samples. *Anal. Chem.* **76**, 5762-5768 (2004).
119. Li, L. *et al.* Determination of the stable carbon isotopic compositions of 2-methyltetrols for four forest areas in Southwest China: The implications for the $\delta^{13}C$ values of atmospheric isoprene and C3/C4 vegetation distribution. *Sci. Total Environ.* **678**, 780-792 (2019).
120. Li, L. *et al.* Determination of the stable carbon isotopic compositions of 2-methyltetrols in ambient aerosols from the Changbai Mountains. *Rapid Commun. Mass Spectrom.* **24**, 1625-1628 (2010).
121. Li, Q. *et al.* Development of a Compound-Specific Carbon Isotope Analysis Method for 2-Methyltetrols, Biomarkers for Secondary Organic Aerosols from Atmospheric Isoprene. *Anal. Chem.* **82**, 6764-6769 (2010).
122. Lin, Y. C. *et al.* Development of a monitoring system for semicontinuous measurements of stable carbon isotope ratios in atmospheric carbonaceous aerosols: optimized methods and application to field measurements. *Anal. Chem.* **92**, 14373-14382 (2020).
123. Butkus, L. *et al.* Development of graphitization method for low carbon aerosol filter samples with Automated Graphitization System AGE-3. *Appl. Radiat. Isot.* **190**, 110461 (2022).
124. Klinedinst, D. B. & Currie, L. A. Direct quantification of $PM_{2.5}$ fossil and biomass carbon within the northern

front range air quality study's domain. *Environ. Sci. Technol.* **33**, 4146-4154 (1999).

125. Xu, Z. *et al.* Distribution and ^{13}C Signature of dicarboxylic acids and related compounds in fine aerosols near underground coal fires in North China: Implications for fossil origin of azelaic acid. *J. Geophys. Res. Atmospheres* **128**, e2023JD038748 (2023).
126. Shen, M. *et al.* Distribution and stable carbon isotopic composition of dicarboxylic acids, ketocarboxylic acids and α-dicarbonyls in fresh and aged biomass burning aerosols. *Atmospheric Chem. Phys.* **22**, 7489-7504 (2022).
127. Narukawa, M. *et al.* Distribution of dicarboxylic acids and carbon isotopic compositions in aerosols from 1997 Indonesian forest fires. *Geophys. Res. Lett.* **26**, 3101-3104 (1999).
128. Kawamura, K. *et al.* Distributions of low molecular weight dicarboxylic acids, ketoacids and α-dicarbonyls in the marine aerosols collected over the Arctic Ocean during late summer. *Biogeosciences* **9**, 4725-4737 (2012).
129. Zotter, P. *et al.* Diurnal cycle of fossil and nonfossil carbon using radiocarbon analyses during CalNex. *J. Geophys. Res. Atmospheres* **119**, 6818-6835 (2014).
130. Fu, P. Q. *et al.* Diurnal variations of organic molecular tracers and stable carbon isotopic composition in atmospheric aerosols over Mt. Tai in the North China Plain: an influence of biomass burning. *Atmospheric Chem. Phys.* **12**, 8359-8375 (2012).
131. Fang, W. *et al.* Divergent evolution of carbonaceous aerosols during dispersal of East Asian haze. *Sci. Rep.* **7**, 10422 (2017).
132. Szidat, S. *et al.* Dominant impact of residential wood burning on particulate matter in Alpine valleys during winter. *Geophys. Res. Lett.* **34**, (2007).
133. Bikkina, S. *et al.* Dual carbon isotope characterization of total organic carbon in wintertime carbonaceous aerosols from northern India. *J. Geophys. Res. Atmospheres* **121**, 4797-4809 (2016).
134. Devaprasad, M. *et al.* Dual carbon isotope-based brown carbon aerosol characteristics at a high-altitude site in the northeastern Himalayas: Role of biomass burning. *Sci. Total Environ.* **912**, 169451 (2024).
135. Mo, Y. *et al.* Dual carbon isotope-based source apportionment and light absorption properties of water-soluble organic carbon in $PM_{2.5}$ over China. *J. Geophys. Res. Atmospheres* **126**, e2020JD033920 (2021).
136. Jiang, F. *et al.* Dual-carbon isotope constraints on source apportionment of black carbon in the megacity Guangzhou of the Pearl River Delta region, China for 2018 autumn season. *Environ. Pollut.* **294**, 118638 (2022).
137. Kawashima, H. & Haneishi, Y. Effects of combustion emissions from the Eurasian continent in winter on seasonal $\delta^{13}C$ of elemental carbon in aerosols in Japan. *Atmos. Environ.* **46**, 568-579 (2012).
138. Masalaite, A. *et al.* Elucidating carbonaceous aerosol sources by the stable carbon $\delta^{13}C_{TC}$ ratio in size-segregated particles. *Atmospheric Res.* **158-159**, 1-12 (2015).
139. Yau, Y. Y. Y. *et al.* Elucidating sources of atmospheric NO_x pollution in a heavily urbanized environment using multiple stable isotopes. *Sci. Total Environ.* **832**, 154781 (2022).
140. Wang, R. *et al.* Emission characterization and $\delta^{13}C$ values of parent PAHs and nitro-PAHs in size-segregated particulate matters from coal-fired power plants. *J. Hazard. Mater.* **318**, 487-496 (2016).
141. Wang, J. *et al.* Enhanced aqueous-phase formation of secondary organic aerosols due to the regional biomass burning over North China Plain. *Environ. Pollut.* **256**, 113401 (2020).
142. Pavuluri, C. M. *et al.* Enhanced modern carbon and biogenic organic tracers in Northeast Asian aerosols during spring/summer. *J. Geophys. Res. Atmospheres* **118**, 2362-2371 (2013).
143. Pavuluri, C. M. & Kawamura, K. Enrichment of ^{13}C in diacids and related compounds during photochemical processing of aqueous aerosols: New proxy for organic aerosols aging. *Sci. Rep.* **6**, 36467 (2016).
144. Bonvalot, L. *et al.* Estimating contributions from biomass burning, fossil fuel combustion, and biogenic carbon to carbonaceous aerosols in the Valley of Chamonix: A dual approach based on radiocarbon and levoglucosan. *Atmospheric Chem. Phys.* **16**, 13753-13772 (2016).
145. Zenker, K. *et al.* Evaluation and inter-comparison of oxygen-based OC-EC separation methods for radiocarbon analysis of ambient aerosol particle samples. *Atmosphere* **8**, 226 (2017).
146. Xiao, H. W. *et al.* Evaluation of black carbon source apportionment based on one year's daily observations in Beijing. *Sci. Total Environ.* **773**, 145668 (2021).
147. Irei, S. *et al.* Evaluation of transboundary secondary organic aerosol in the urban air of Western Japan: direct comparison of two site observations. *ACS Earth Space Chem.* **2**, 1231-1239 (2018).
148. Martinsson, J. *et al.* Evaluation of $\delta^{13}C$ in carbonaceous aerosol source apportionment at a rural measurement site. *Aerosol Air Qual. Res.* **17**, 2081-2094 (2017).
149. Odwuor, A. *et al.* Evidence for multi-decadal fuel buildup in a large California wildfire from smoke

radiocarbon measurements. *Environ. Res. Lett.* **18**, 094030 (2023).
150. Xu, B. *et al.* Fates of secondary organic aerosols in the atmosphere identified from compound-specific dual-carbon isotope analysis of oxalic acid. *Atmospheric Chem. Phys.* **23**, 1565-1578 (2023).
151. Lemire, K. R. *et al.* Fine particulate matter source attribution for Southeast Texas using $^{14}C/^{13}C$ ratios. *J. Geophys. Res. Atmospheres* **107**, ACH 3-1-ACH 3-7 (2002).
152. Fu, P. *et al.* Fluorescent water-soluble organic aerosols in the High Arctic atmosphere. *Sci. Rep.* **5**, 9845 (2015).
153. Wozniak, A. S. *et al.* M. Fossil and contemporary aerosol particulate organic carbon in the eastern United States: Implications for deposition and inputs to watersheds. *Glob. Biogeochem. Cycles* **25**, (2011).
154. Schichtel, B. A. *et al.* Fossil and contemporary fine particulate carbon fractions at 12 rural and urban sites in the United States. *J. Geophys. Res. Atmospheres* **113**, (2008).
155. Liu, D. *et al.* Fossil and non-fossil fuel sources of organic and elemental carbonaceous aerosol in Beijing, Shanghai, and Guangzhou: Seasonal carbon source variation. *Aerosol Air Qual. Res.* **20**, 2495-2506 (2020).
156. Ulevicius, V. *et al.* Fossil and non-fossil source contributions to atmospheric carbonaceous aerosols during extreme spring grassland fires in Eastern Europe. *Atmospheric Chem. Phys.* **16**, 5513-5529 (2016).
157. Zhang, Y. L. *et al.* Fossil and non-fossil sources of different carbonaceous fractions in fine and coarse particles by radiocarbon measurement. *Radiocarbon* **55**, 1510-1520 (2013).
158. Szidat, S. *et al.* Fossil and non-fossil sources of organic carbon (OC) and elemental carbon (EC) in Göteborg, Sweden. *Atmospheric Chem. Phys.* **9**, 1521-1535 (2009).
159. Cha, J. Y. *et al.* Fossil and non-fossil sources of the carbonaceous component of $PM_{2.5}$ in forest and urban areas. *Sci. Rep.* **13**, 5486 (2023).
160. Glasius, M. *et al.* Fossil and nonfossil carbon in fine particulate matter: A study of five European cities. *J. Geophys. Res. Atmospheres* **116**, (2011).
161. Zhang, Y. L. *et al.* Fossil and nonfossil sources of organic and elemental carbon aerosols in the outflow from Northeast China. *Environ. Sci. Technol.* **50**, 6284-6292 (2016).
162. Zhang, Y. *et al.* Fossil and nonfossil sources of winter organic aerosols in the regional background atmosphere of China. *Environ. Sci. Technol.* **58**, 1244-1254 (2024).
163. Salma, I. *et al.* Fossil fuel combustion, biomass burning and biogenic sources of fine carbonaceous aerosol in the Carpathian Basin. *Atmospheric Chem. Phys.* **20**, 4295-4312 (2020).
164. Tanner, R. L. *et al.* Fossil Sources of Ambient Aerosol Carbon Based on ^{14}C Measurements Special Issue of Aerosol Science and Technology on Findings from the Fine Particulate Matter Supersites Program. *Aerosol Sci. Technol.* **38**, 133-139 (2004).
165. Minguillón, M. C. *et al.* Fossil versus contemporary sources of fine elemental and organic carbonaceous particulate matter during the DAURE campaign in Northeast Spain. *Atmospheric Chem. Phys.* **11**, 12067-12084 (2011).
166. Zhang, Y. L. *et al.* Fossil vs. non-fossil sources of fine carbonaceous aerosols in four Chinese cities during the extreme winter haze episode of 2013. *Atmospheric Chem. Phys.* **15**, 1299-1312 (2015).
167. Di Palma, A. *et al.* Geochemistry and carbon isotopic ratio for assessment of PM_{10} composition, source and seasonal trends in urban environment. *Environ. Pollut.* **239**, 590-598 (2018).
168. Sirignano, C. *et al.* High Contribution of Biomass Combustion to $PM_{2.5}$ in the City Centre of Naples (Italy). *Atmosphere* **10**, 451 (2019).
169. Zhang, Y. *et al.* High Contribution of nonfossil sources to submicrometer organic aerosols in Beijing, China. *Environ. Sci. Technol.* **51**, 7842-7852 (2017).
170. Ni, H. *et al.* High contributions of fossil sources to more volatile organic aerosol. *Atmospheric Chem. Phys.* **19**, 10405-10422 (2019).
171. Zhang, W. *et al.* High time-resolved measurement of stable carbon isotope composition in water-soluble organic aerosols: method optimization and a case study during winter haze in eastern China. *Atmospheric Chem. Phys.* **19**, 11071-11087 (2019).
172. Peng, P. *et al.* Humic acid, kerogen, and black carbon isolated from atmospheric total suspended particulate from Guangzhou, China. *Aerosol Sci. Technol.* **47**, 1342-1352 (2013).
173. Garbarienė, I. *et al.* Identification of wintertime carbonaceous fine particulate matter ($PM_{2.5}$) sources in Kaunas, Lithuania using polycyclic aromatic hydrocarbons and stable carbon isotope analysis. *Atmos. Environ.* **237**, 117673 (2020).
174. Bao, M. *et al.* Impact of fossil and non-fossil fuel sources on the molecular compositions of water-soluble humic-like substances in $PM_{2.5}$ at a suburban site of Yangtze River Delta, China. *Atmospheric Chem. Phys.* **23**, 8305-8324 (2023).

175. Zheng, L. *et al.* Impacts of springtime biomass burning in the northern Southeast Asia on marine organic aerosols over the Gulf of Tonkin, China. *Environ. Pollut.* **237**, 285-297 (2018).
176. Wang, S. *et al.* Implications for biomass/coal combustion emissions and secondary formation of carbonaceous aerosols in North China. *RSC Adv.* **8**, 38108-38117 (2018).
177. Yan, C. *et al.* Important fossil source contribution to brown carbon in Beijing during winter. *Sci. Rep.* **7**, 43182 (2017).
178. Song, J. *et al.* Influence of biomass burning on atmospheric aerosols over the western South China Sea: Insights from ions, $PM_{2.5}$ carbonaceous fractions and stable carbon isotope ratios. *Environ. Pollut.* **242**, 1800-1809 (2018).
179. Ikemori, F. *et al*. Influence of contemporary carbon originating from the 2003 Siberian forest fire on organic carbon in Nagoya, Japan. *Sci. Total Environ.* **530-531**, 403-410 (2015).
180. Shen, M. *et al.* Influence of vertical transport on chemical evolution of dicarboxylic acids and related secondary organic aerosol from surface emission to the top of Mount Hua, Northwest China. *Sci. Total Environ.* **858**, 159892 (2023).
181. Cao, F. *et al*. Inorganic markers, carbonaceous components and stable carbon isotope from biomass burning aerosols in Northeast China. *Sci. Total Environ.* **572**, 1244-1251 (2016).
182. Singh, G. K. *et al.* Investigation of sources and atmospheric transformation of carbonaceous aerosols from Shyamnagar, eastern Indo-Gangetic Plains: Insights from $\delta^{13}C$ and carbon fractions. *Chemosphere* **326**, 138422 (2023).
183. Song, W. *et al.* Is biomass burning always a dominant contributor of fine aerosols in upper northern Thailand? *Environ. Int.* **168**, 107466 (2022).
184. Liu, J. *et al.* Isotope constraints of the strong influence of biomass burning to climate-forcing Black Carbon aerosols over Southeast Asia. *Sci. Total Environ.* **744**, 140359 (2020).
185. Winiger, P. *et al*. Isotope-based source apportionment of EC aerosol particles during winter high-pollution events at the Zeppelin Observatory, Svalbard. *Environ. Sci. Technol.* **49**, 11959-11966 (2015).
186. Wozniak, A. S. *et al*. Isotopic characterization of aerosol organic carbon components over the eastern United States. *J. Geophys. Res. Atmospheres* **117**, (2012).
187. Liu, G. *et al.* Isotopic compositions of elemental carbon in smoke and ash derived from crop straw combustion. *Atmos. Environ.* **92**, 303-308 (2014).
188. Zhang, Y. L. *et al.* Large contribution of fossil fuel derived secondary organic carbon to water soluble organic aerosols in winter haze in China. *Atmospheric Chem. Phys.* **18**, 4005-4017 (2018).
189. Xu, B. *et al.* Large contribution of fossil-derived components to aqueous secondary organic aerosols in China. *Nat. Commun.* **13**, 5115 (2022).
190. Bendle, J. *et al*. Latitudinal distribution of terrestrial lipid biomarkers and *n*-alkane compound-specific stable carbon isotope ratios in the atmosphere over the western Pacific and Southern Ocean. *Geochim. Cosmochim. Acta* **71**, 5934-5955 (2007).
191. Miyazaki, Y. *et al*. Latitudinal distributions of organic nitrogen and organic carbon in marine aerosols over the western North Pacific. *Atmospheric Chem. Phys.* **11**, 3037-3049 (2011).
192. Gu, W. *et al.* Marine fresh carbon pool dominates summer carbonaceous aerosols over Arctic Ocean. *J. Geophys. Res. Atmospheres* **128**, e2022JD037692 (2023).
193. Dobashi, T. *et al.* Marine nitrogen fixation as a possible source of atmospheric water-soluble organic nitrogen aerosols in the subtropical North Pacific. *Biogeosciences* **20**, 439-449 (2023).
194. Ni, H. *et al.* Measurement report: dual-carbon isotopic characterization of carbonaceous aerosol reveals different primary and secondary sources in Beijing and Xi'an during severe haze events. *Atmospheric Chem. Phys.* **20**, 16041-16053 (2020).
195. Meng, J. *et al.* Measurement report: Investigation on the sources and formation processes of dicarboxylic acids and related species in urban aerosols before and during the COVID-19 lockdown in Jinan, East China. *Atmospheric Chem. Phys.* **23**, 14481-14503 (2023).
196. Zhao, H. *et al.* Measurement report: Source apportionment of carbonaceous aerosol using dual-carbon isotopes (^{13}C and ^{14}C) and levoglucosan in three northern Chinese cities during 2018-2019. *Atmospheric Chem. Phys.* **22**, 6255-6274 (2022).
197. Suto, N. & Kawashima, H. Measurement report: Source characteristics of water-soluble organic carbon in $PM_{2.5}$ at two sites in Japan, as assessed by long-term observation and stable carbon isotope ratio. *Atmospheric Chem. Phys.* **21**, 11815-11828 (2021).
198. Moukhtar, S. *et al.* Method for determination of stable carbon isotope ratio of methylnitrophenols in atmospheric particulate matter. *Atmospheric Meas. Tech.* **4**, 2453-2464 (2011).

199. Xu, L. *et al.* Modern and fossil contributions to polycyclic aromatic hydrocarbons in $PM_{2.5}$ from North Birmingham, Alabama in the Southeastern U.S. *Environ. Sci. Technol.* **46**, 1422-1429 (2012).
200. Meng, J. *et al.* Molecular characteristics and stable carbon isotope compositions of dicarboxylic acids and related compounds in the urban atmosphere of the North China Plain: Implications for aqueous phase formation of SOA during the haze periods. *Sci. Total Environ.* **705**, 135256 (2020).
201. Qi, W. *et al.* Molecular characteristics and stable carbon isotope compositions of dicarboxylic acids and related compounds in wintertime aerosols of Northwest China. *Sci. Rep.* **12**, 11266 (2022).
202. Li, L. *et al.* Molecular characterization and seasonal variation in primary and secondary organic aerosols in Beijing, China. *J. Geophys. Res. Atmospheres* **123**, 12,394-12,412 (2018).
203. Okuda, T. *et al.* Molecular composition and compound-specific stable carbon isotope ratio of polycyclic aromatic hydrocarbons (PAHs) in the atmosphere in suburban areas. *Geochem. J.* **38**, 89-100 (2004).
204. Li, J. *et al.* Molecular compositions and stable carbon isotopes ($\delta^{13}C$) of PAHs in wintertime $PM_{2.5}$ in urban Xi'an, China: Implications for source distribution and atmospheric oxidation. *Atmospheric Res.* **292**, 106890 (2023).
205. Zhao, W. *et al.* Molecular distribution and compound-specific stable carbon isotopic composition of dicarboxylic acids, oxocarboxylic acids and α-dicarbonyls in $PM_{2.5}$ from Beijing, China. *Atmospheric Chem. Phys.* **18**, 2749-2767 (2018).
206. Meng, J. *et al.* Molecular distribution and stable carbon isotopic compositions of dicarboxylic acids and related SOA from biogenic sources in the summertime atmosphere of Mt. Tai in the North China Plain. *Atmospheric Chem. Phys.* **18**, 15069-15086 (2018).
207. Zhao, X. *et al.* Molecular distributions and ^{13}C isotopic composition of dicarboxylic acids, oxocarboxylic acids, and α-dicarbonyls in wintertime $PM_{2.5}$ at three sites over Northeast Asia: Implications for origins and long-range atmospheric transport. *J. Geophys. Res. Atmospheres* **128**, e2023JD038864 (2023).
208. Ren, L. *et al.* Molecular distributions and compound-specific stable carbon isotopic compositions of lipids in wintertime aerosols from Beijing. *Sci. Rep.* **6**, 27481 (2016).
209. Kim, J. H. *et al.* Molecular distributions and compound-specific stable carbon isotopic compositions of plant wax n-alkanes in marine aerosols along a north-south transect in the Arctic-Northwest Pacific Region. *Atmosphere* **11**, 499 (2020).
210. Kawamura, K. *et al.* Molecular distributions and isotopic compositions of organic aerosols over the western North Atlantic: Dicarboxylic acids, related compounds, sugars, and secondary organic aerosol tracers. *Org. Geochem.* **113**, 229-238 (2017).
211. Kirillova, E. N. *et al.* Natural abundance ^{13}C and ^{14}C analysis of water-soluble organic carbon in atmospheric aerosols. *Anal. Chem.* **82**, 7973-7978 (2010).
212. Ni, H. *et al.* Non-Fossil origin explains the large seasonal variation of highly processed organic aerosol in the Northeastern Tibetan Plateau (3,200 m a.s.l.). *Geophys. Res. Lett.* **50**, e2023GL104710 (2023).
213. Kosztowniak, E. *et al.* OC/EC from PM10 in the vicinity of Turów lignite open-pit mine (SW Poland): Carbon isotopic approach. *Atmospheric Pollut. Res.* **7**, 40-48 (2016).
214. Zhang, Y. *et al.* On the fossil and non-fossil fuel sources of carbonaceous aerosol with radiocarbon and AMS-PMF methods during winter hazy days in a rural area of North China plain. *Environ. Res.* **208**, 112672 (2022).
215. Wagner, S. *et al.* Online quantification and compound-specific stable isotopic analysis of black carbon in environmental matrices via liquid chromatography-isotope ratio mass spectrometry. *Limnol. Oceanogr. Methods* **15**, 995-1006 (2017).
216. Suto, N. & Kawashima, H. Online wet oxidation/isotope ratio mass spectrometry method for determination of stable carbon isotope ratios of water-soluble organic carbon in particulate matter. *Rapid Commun. Mass Spectrom.* **32**, 1668-1674 (2018).
217. Liu, J. *et al.* Optimizing isolation protocol of organic carbon and elemental carbon for ^{14}C analysis using fine particulate samples. *Atmos. Environ.* **154**, 9-19 (2017).
218. Ni, H. *et al.* Organic aerosol formation and aging processes in Beijing constrained by size-resolved measurements of radiocarbon and stable isotopic ^{13}C. *Environ. Int.* **158**, 106890 (2022).
219. Garbarienė, I. *et al.* Origin Identification of Carbonaceous Aerosol Particles by Carbon Isotope Ratio Analysis. *Aerosol Air Qual. Res.* **16**, 1356-1365 (2016).
220. Simu, S. A. *et al.* Origin of water-soluble organic aerosols at the Maïdo high-altitude observatory, Réunion Island, in the tropical Indian Ocean. *Atmospheric Chem. Phys.* **21**, 17017-17029 (2021).
221. Verrilli, S. *et al.* $PM_{2.5}$ Size distribution and characterization by carbon isotope in Tuscany (Italy). *Chem. Eng. Trans.* **22**, (2010).
222. Chen, Y. *et al.* Polluting characteristics, sources, cancer risk, and cellular toxicity of PAHs bound in

atmospheric particulates sampled from an economic transformation demonstration area of Dongguan in the Pearl River Delta, China. *Environ. Res.* **215**, 114383 (2022).

223. Geng, X. *et al.* Provenance of aerosol black carbon over Northeast Indian Ocean and South China Sea and implications for oceanic black carbon cycling. *Environ. Sci. Technol.* **57**, 13067-13078 (2023).
224. Zencak, Z. *et al.* Quantification and radiocarbon source apportionment of black carbon in atmospheric aerosols using the CTO-375 method. *Atmos. Environ.* **41**, 7895-7906 (2007).
225. Yu, M. *et al.* Quantification of fossil and non-fossil sources to the reduction of carbonaceous aerosols in the Yangtze River Delta, China: Insights from radiocarbon analysis during 2014-2019. *Atmos. Environ.* **292**, 119421 (2023).
226. Ceburnis, D. *et al.* Quantification of the carbonaceous matter origin in submicron marine aerosol by ^{13}C and ^{14}C isotope analysis. *Atmospheric Chem. Phys.* **11**, 8593-8606 (2011).
227. Jiang, F. *et al.* Quantitative evaluation for the sources and aging processes of organic aerosols in urban Guangzhou: Insights from a comprehensive method of dual-carbon isotopes and macro tracers. *Sci. Total Environ.* **888**, 164182 (2023).
228. Fushimi, A. *et al.* Radiocarbon (^{14}C) Diurnal Variations in Fine Particles at Sites Downwind from Tokyo, Japan in Summer. *Environ. Sci. Technol.* **45**, 6784-6792 (2011).
229. Szidat, S. *et al.* Radiocarbon (^{14}C)-deduced biogenic and anthropogenic contributions to organic carbon (OC) of urban aerosols from Zürich, Switzerland. *Atmos. Environ.* **38**, 4035-4044 (2004).
230. Handa, D. *et al.* Radiocarbon analysis of BC and OC in PM_{10} aerosols at Cape Hedo, Okinawa, Japan, during long-range transport events from East Asian countries. *Nucl. Instrum. Methods Phys. Res. Sect. B Beam Interact. Mater. At.* **268**, 1125-1128 (2010).
231. Kontuľ, I. *et al.* Radiocarbon analysis of carbonaceous aerosols in Bratislava, Slovakia. *J. Environ. Radioact.* **218**, 106221 (2020).
232. Pang, Y. *et al.* Radiocarbon characterization study of atmospheric $PM_{2.5}$ in Beijing during the 2014 APEC summit. *Radiocarbon* **61**, 1643-1652 (2019).
233. Takahashi, K. *et al.* Radiocarbon content in urban atmospheric aerosols. *Water. Air. Soil Pollut.* **185**, 305-310 (2007).
234. Lewis, C. W. & Stiles, D. C. Radiocarbon content of $PM_{2.5}$ ambient aerosol in Tampa, FL. *Aerosol Sci. Technol.* **40**, 189-196 (2006).
235. Isley, C. F. *et al.* Radiocarbon determination of fossil and contemporary carbon contribution to aerosol in the Pacific Islands. *Sci. Total Environ.* **643**, 183-192 (2018).
236. Jordan, T. B. *et al.* Radiocarbon determination of woodsmoke contribution to air particulate matter in Launceston, Tasmania. *Atmos. Environ.* **40**, 2575-2582 (2006).
237. Varga, T. *et al.* Radiocarbon in the atmospheric gases and PM_{10} aerosol around the Paks Nuclear Power Plant, Hungary. *J. Environ. Radioact.* **237**, 106670 (2021).
238. Lewis, C. W. *et al.* Radiocarbon measurement of the biogenic contribution to summertime $PM_{2.5}$ ambient aerosol in Nashville, TN. *Atmos. Environ.* **38**, 6053-6061 (2004).
239. Matsumoto, K. *et al.* Radiocarbon variability of fatty acids in semi-urban aerosol samples. *Nucl. Instrum. Methods Phys. Res. Sect. B Beam Interact. Mater. At.* **223-224**, 842-847 (2004).
240. Zong, Z. *et al.* Radiocarbon-based impact assessment of open biomass burning on regional carbonaceous aerosols in North China. *Sci. Total Environ.* **518-519**, 1-7 (2015).
241. Uchida, M. *et al.* Radiocarbon-based source apportionment of black carbon (BC) in PM_{10} aerosols from residential area of suburban Tokyo. *Nucl. Instrum. Methods Phys. Res. Sect. B Beam Interact. Mater. At.* **268**, 1120-1124 (2010).
242. Zhang, Y. L. *et al.* Radiocarbon-based source apportionment of carbonaceous aerosols at a regional background site on Hainan Island, South China. *Environ. Sci. Technol.* **48**, 2651-2659 (2014).
243. Budhavant, K. *et al.* Radiocarbon-based source apportionment of elemental carbon aerosols at two South Asian receptor observatories over a full annual cycle. *Environ. Res. Lett.* **10**, 064004 (2015).
244. Liu, J. *et al.* Radiocarbon-derived source apportionment of fine carbonaceous aerosols before, during, and after the 2014 Asia-Pacific Economic Cooperation (APEC) summit in Beijing, China. *J. Geophys. Res. Atmospheres* **121**, 4177-4187 (2016).
245. Andersson, A. *et al.* Regionally-varying combustion sources of the January 2013 severe haze events over Eastern China. *Environ. Sci. Technol.* **49**, 2038-2043 (2015).
246. Yan, C. *et al.* Residential coal combustion as a source of levoglucosan in China. *Environ. Sci. Technol.* **52**, 1665-1674 (2018).
247. Vodička, P. *et al.* Seasonal changes in stable carbon isotopic composition in the bulk aerosol and gas phases

at a suburban site in Prague. *Sci. Total Environ.* **803**, 149767 (2022).
248. Bendle, J. A. *et al*. Seasonal changes in stable carbon isotopic composition of *n*-alkanes in the marine aerosols from the western North Pacific: Implications for the source and atmospheric transport. *Geochim. Cosmochim. Acta* **70**, 13-26 (2006).
249. Pavuluri, C. M. & Kawamura, K. Seasonal changes in TC and WSOC and their ^{13}C isotope ratios in Northeast Asian aerosols: land surface-biosphere-atmosphere interactions. *Acta Geochim.* **36**, 355-358 (2017).
250. Masalaite, A. *et al.* Seasonal changes of sources and volatility of carbonaceous aerosol at urban, coastal and forest sites in Eastern Europe (Lithuania). *Atmos. Environ.* **225**, 117374 (2020).
251. Rodríguez, B. T. *et al.* Seasonal cycle of isotope-based source apportionment of elemental carbon in airborne particulate matter and snow at Alert, Canada. *J. Geophys. Res. Atmospheres* **125**, e2020JD033125 (2020).
252. Masalaite, A. *et al.* Seasonal observation and source apportionment of carbonaceous aerosol from forested rural site (Lithuania). *Atmos. Environ.* **272**, 118934 (2022).
253. Zou, D. *et al*. Seasonal source analysis of nitrogen and carbon aerosols of $PM_{2.5}$ in typical cities of Zhejiang, China. *Chemosphere* **303**, 135026 (2022).
254. Andersson, A. *et al.* Seasonal source variability of carbonaceous aerosols at the Rwanda Climate Observatory. *Atmospheric Chem. Phys.* **20**, 4561-4573 (2020).
255. Miyazaki, Y. *et al*. Seasonal variations of stable carbon isotopic composition and biogenic tracer compounds of water-soluble organic aerosols in a deciduous forest. *Atmospheric Chem. Phys.* **12**, 1367-1376 (2012).
256. Kundu, S. & Kawamura, K. Seasonal variations of stable carbon isotopic composition of bulk aerosol carbon from Gosan site, Jeju Island in the East China Sea. *Atmos. Environ.* **94**, 316-322 (2014).
257. Chen, Q. *et al*. Seasonal variations of terrestrial OC sources in aerosols over the East China Sea: The influence of long-range air mass transport. *J. Ocean Univ. China* **20**, 1147-1156 (2021).
258. Shi, H. *et al.* Secondary formation and source analysis of carbonaceous components in PM_1 in a typical city, Southwest of China. *Atmos. Environ.* **299**, 119671 (2023).
259. Minguillón, M. C. *et al.* Secondary organic aerosol origin in an urban environment: Influence of biogenic and fuel combustion precursors. *Faraday Discuss.* **189**, 337-359 (2016).
260. Winiger, P. *et al.* Siberian Arctic black carbon sources constrained by model and observation. *Proc. Natl. Acad. Sci.* **114**, E1054-E1061 (2017).
261. Kawichai, S. *et al.* Significant contribution of C3 - type forest plants' burning to airborne $PM_{2.5}$ pollutions in Chiang Mai Province, Northern Thailand. *Chiang Mai Univ. J. Nat. Sci.* **20**, 1-16 (2021).
262. Miyazaki, Y. *et al*. Size distributions of organic nitrogen and carbon in remote marine aerosols: Evidence of marine biological origin based on their isotopic ratios. *Geophys. Res. Lett.* **37**, (2010).
263. Wiggins, E. B. *et al.* Smoke radiocarbon measurements from Indonesian fires provide evidence for burning of millennia-aged peat. *Proc. Natl. Acad. Sci.* **115**, 12419-12424 (2018).
264. Zong, Z. *et al.* Source and formation characteristics of water-soluble organic carbon in the anthropogenic-influenced Yellow River Delta, North China. *Atmos. Environ.* **144**, 124-132 (2016).
265. Liu, J. *et al.* Source apportionment and dynamic changes of carbonaceous aerosols during the haze bloom-decay process in China based on radiocarbon and organic molecular tracers. *Atmospheric Chem. Phys.* **16**, 2985-2996 (2016).
266. Wang, G. *et al.* Source apportionment of atmospheric carbonaceous particulate matter based on the radiocarbon. *J. Radioanal. Nucl. Chem.* **295**, 1545-1552 (2013).
267. Mousavi, A. *et al.* Source apportionment of black carbon (BC) from fossil fuel and biomass burning in metropolitan Milan, Italy. *Atmos. Environ.* **203**, 252-261 (2019).
268. Uchida, M. *et al.* Source apportionment of black carbon aerosols by isotopes (^{14}C and ^{13}C) and Bayesian modeling from two remote islands in east Asian outflow region. *Nucl. Instrum. Methods Phys. Res. Sect. B Beam Interact. Mater. At.* **538**, 64-74 (2023).
269. Xiao, H. W. *et al*. Source apportionment of black carbon aerosols in winter across China. *Atmos. Environ.* **298**, 119622 (2023).
270. Genberg, J. *et al.* Source apportionment of carbonaceous aerosol in southern Sweden. *Atmospheric Chem. Phys.* **11**, 11387-11400 (2011).
271. Hou, S. *et al.* Source apportionment of carbonaceous aerosols in Beijing with radiocarbon and organic tracers: insight into the differences between urban and rural sites. *Atmospheric Chem. Phys.* **21**, 8273-8292 (2021).
272. Li, M. *et al.* Source apportionment of carbonaceous aerosols in diverse atmospheric environments of China by dual-carbon isotope method. *Sci. Total Environ.* **806**, 150654 (2022).
273. Bonvalot, L. *et al.* Source apportionment of carbonaceous aerosols in the vicinity of a Mediterranean industrial harbor: A coupled approach based on radiocarbon and molecular tracers. *Atmos. Environ.* **212**, 250-

261 (2019).
274. Ni, H. *et al.* Source apportionment of carbonaceous aerosols in Xi'an, China: insights from a full year of measurements of radiocarbon and the stable isotope ^{13}C. *Atmospheric Chem. Phys.* **18**, 16363-16383 (2018).
275. Salma, I. *et al.* Source apportionment of carbonaceous chemical species to fossil fuel combustion, biomass burning and biogenic emissions by a coupled radiocarbon-levoglucosan marker method. *Atmospheric Chem. Phys.* **17**, 13767-13781 (2017).
276. Wei, N. *et al.* Source apportionment of carbonaceous particulate matter during haze days in Shanghai based on the radiocarbon. *J. Radioanal. Nucl. Chem.* **313**, 145-153 (2017).
277. Wang, G. *et al.* Source apportionment of carbonaceous particulate matter in a Shanghai suburb based on carbon isotope composition. *Aerosol Sci. Technol.* **47**, 239-248 (2013).
278. Winiger, P. *et al.* Source apportionment of circum-Arctic atmospheric black carbon from isotopes and modeling. *Sci. Adv.* **5**, eaau8052 (2019).
279. Zhang, Y. L. *et al.* Source apportionment of elemental carbon in Beijing, China: Insights from radiocarbon and organic marker measurements. *Environ. Sci. Technol.* **49**, 8408-8415 (2015).
280. Al-Naiema, I. M. *et al.* Source apportionment of fine particulate matter organic carbon in Shenzhen, China by chemical mass balance and radiocarbon methods. *Environ. Pollut.* **240**, 34-43 (2018).
281. Mari, M. *et al.* Source apportionment of inorganic and organic PM in the ambient air around a cement plant: Assessment of complementary tools. *Aerosol Air Qual. Res.* **16**, 3230-3242 (2016).
282. Zong, Z. *et al.* Source apportionment of $PM_{2.5}$ at a regional background site in North China using PMF linked with radiocarbon analysis: insight into the contribution of biomass burning. *Atmospheric Chem. Phys.* **16**, 11249-11265 (2016).
283. Jiang, H. *et al.* Source apportionment of $PM_{2.5}$ carbonaceous aerosols during a long-lasting winter haze episode in Xiangyang, central China. *Atmospheric Pollut. Res.* **12**, 470-479 (2021).
284. Gelencsér, A. *et al.* Source apportionment of $PM_{2.5}$ organic aerosol over Europe: Primary/secondary, natural/anthropogenic, and fossil/biogenic origin. *J. Geophys. Res. Atmospheres* **112**, (2007).
285. Ramos-Contreras, C. *et al.* Source apportionment of PM_{10} and health risk assessment related in a narrow tropical valley. Study case: Metropolitan area of Aburrá Valley (Colombia). *Environ. Sci. Pollut. Res.* **30**, 60036-60049 (2023).
286. Skiba, A. *et al.* Source apportionment of suspended particulate matter (PM_1, $PM_{2.5}$ and PM_{10}) collected in road and tram tunnels in Krakow, Poland. *Environ. Sci. Pollut. Res.* **31**, 14690-14703 (2024).
287. Liu, J. *et al.* Source apportionment using radiocarbon and organic tracers for $PM_{2.5}$ carbonaceous aerosols in Guangzhou, South China: Contrasting local- and regional-scale haze events. *Environ. Sci. Technol.* **48**, 12002-12011 (2014).
288. Ke, L. *et al*. Source contributions to carbonaceous aerosols in the Tennessee Valley Region. *Atmos. Environ.* **41**, 8898-8923 (2007).
289. Barrett, T. E. *et al*. Source contributions to wintertime elemental and organic carbon in the Western Arctic based on radiocarbon and tracer apportionment. *Environ. Sci. Technol.* **49**, 11631-11639 (2015).
290. Tang, T. *et al.* Source diversity of intermediate volatility n-alkanes revealed by compound-specific $\delta^{13}C-\delta D$ isotopes. *Environ. Sci. Technol.* **56**, 14262-14271 (2022).
291. Chen, B. *et al.* Source forensics of black carbon aerosols from China. *Environ. Sci. Technol.* **47**, 9102-9108 (2013).
292. Ren, L. *et al.* Source forensics of n-alkanes and n-fatty acids in urban aerosols using compound specific radiocarbon/stable carbon isotopic composition. *Environ. Res. Lett.* **15**, 074007 (2020).
293. Okuda, T. *et al.* Source identification of Malaysian atmospheric polycyclic aromatic hydrocarbons nearby forest fires using molecular and isotopic compositions. *Atmos. Environ.* **36**, 611-618 (2002).
294. Major, I. *et al.* Source identification of $PM_{2.5}$ carbonaceous aerosol using combined carbon fraction, radiocarbon and stable carbon isotope analyses in Debrecen, Hungary. *Sci. Total Environ.* **782**, 146520 (2021).
295. Dasari, S. *et al.* Source quantification of South Asian black carbon aerosols with isotopes and modeling. *Environ. Sci. Technol.* **54**, 11771-11779 (2020).
296. Bosch, C. *et al.* Source-diagnostic dual-isotope composition and optical properties of water-soluble organic carbon and elemental carbon in the South Asian outflow intercepted over the Indian Ocean. *J. Geophys. Res. Atmospheres* **119**, 11,743-11,759 (2014).
297. Masalaite, A. *et al.* Sources and atmospheric processing of size segregated aerosol particles revealed by stable carbon isotope ratios and chemical speciation. *Environ. Pollut.* **240**, 286-296 (2018).
298. Dusek, U. *et al.* Sources and formation mechanisms of carbonaceous aerosol at a regional background site in the Netherlands: insights from a year-long radiocarbon study. *Atmospheric Chem. Phys.* **17**, 3233-3251

(2017).

299. Ni, H. *et al.* Sources and formation of carbonaceous aerosols in Xi'an, China: primary emissions and secondary formation constrained by radiocarbon. *Atmospheric Chem. Phys.* **19**, 15609-15628 (2019).
300. Kirillova, E. N. *et al.* Sources and light absorption of water-soluble organic carbon aerosols in the outflow from northern China. *Atmospheric Chem. Phys.* **14**, 1413-1422 (2014).
301. Yu, M. *et al.* Sources and radiocarbon ages of aerosol organic carbon along the east coast of China and implications for atmospheric fossil carbon contributions to China marginal seas. *Sci. Total Environ.* **619-620**, 957-965 (2018).
302. Li, C. *et al.* Sources of black carbon to the Himalayan-Tibetan Plateau glaciers. *Nat. Commun.* **7**, 12574 (2016).
303. Liu, D. *et al.* Sources of non-fossil-fuel emissions in carbonaceous aerosols during early winter in Chinese cities. *Atmospheric Chem. Phys.* **17**, 11491-11502 (2017).
304. Hildemann, L. M. *et al.* Sources of urban contemporary carbon aerosol. *Environ. Sci. Technol.* **28**, 1565-1576 (1994).
305. Mo, Y. *et al.* Sources, compositions, and optical properties of humic-like substances in Beijing during the 2014 APEC summit: Results from dual carbon isotope and Fourier-transform ion cyclotron resonance mass spectrometry analyses. *Environ. Pollut.* **239**, 322-331 (2018).
306. Jung, C. C. *et al.* Spatial and seasonal variations in the carbon and lead isotopes of $PM_{2.5}$ in air of residential buildings and their applications for source identification. *Environ. Pollut.* **316**, 120654 (2023).
307. Zheng, M. *et al.* Spatial distribution of carbonaceous aerosol in the southeastern United States using molecular markers and carbon isotope data. *J. Geophys. Res. Atmospheres* **111**, (2006).
308. Jung, J. & Kawamura, K. Springtime carbon emission episodes at the Gosan background site revealed by total carbon, stable carbon isotopic composition, and thermal characteristics of carbonaceous particles. *Atmospheric Chem. Phys.* **11**, 10911-10928 (2011).
309. Singh, G. K. *et al.* Stable carbon isotope and bulk composition of wintertime aerosols from Kanpur. in *Environmental Contaminants: Measurement, Modelling and Control* 209-220 (Springer, Singapore, 2018).
310. Peng, L. *et al.* Stable carbon isotope evidence for origin of atmospheric polycyclic aromatic hydrocarbons in Zhengzhou and Urumchi, China. *Geochem. J.* **40**, 219-226 (2006).
311. Sang, X. F. *et al.* Stable carbon isotope ratio analysis of anhydrosugars in biomass burning aerosol particles from source samples. *Environ. Sci. Technol.* **46**, 3312-3318 (2012).
312. Zhao, Z. *et al.* Stable carbon isotopes and levoglucosan for $PM_{2.5}$ elemental carbon source apportionments in the largest city of Northwest China. *Atmos. Environ.* **185**, 253-261 (2018).
313. Cao, J. *et al.* Stable carbon isotopes in aerosols from Chinese cities: Influence of fossil fuels. *Atmos. Environ.* **45**, 1359-1363 (2011).
314. Liu, X. *et al.* Stable carbon isotopic compositions and source apportionment of the carbonaceous components in $PM_{2.5}$ in Taiyuan, China. *Atmos. Environ.* **261**, 118601 (2021).
315. Mkoma, S. *et al.* Stable carbon isotopic compositions of low-molecularweight dicarboxylic acids, glyoxylic acid and glyoxal in tropical aerosols: implications for photochemical processes of organic aerosols. **66**, 23702 (2014).
316. Pavuluri, C. *et al.* Stable carbon isotopic compositions of total carbon, dicarboxylic acids and glyoxylic acid in the tropical Indian aerosols: Implications for sources and photochemical processing of organic aerosols. *J. Geophys. Res. Atmospheres* **116**, (2011).
317. Narukawa, M. *et al.* Stable carbon isotopic ratios and ionic composition of the high-Arctic aerosols: An increase in δ^{13}C values from winter to spring. *J. Geophys. Res. Atmospheres* **113**, (2008).
318. Huang, L. *et al.* Stable isotope measurements of carbon fractions (OC/EC) in airborne particulate: A new dimension for source characterization and apportionment. *Atmos. Environ.* **40**, 2690-2705 (2006).
319. Ceburnis, D. *et al.* Stable isotopes measurements reveal dual carbon pools contributing to organic matter enrichment in marine aerosol. *Sci. Rep.* **6**, 36675 (2016).
320. Guo, Z. *et al.* Stable isotopic compositions of elemental carbon in $PM_{1.1}$ in north suburb of Nanjing Region, China. *Atmospheric Res.* **168**, 105-111 (2016).
321. Zimnoch, M. *et al.* Summer-winter contrast in carbon isotope and elemental composition of total suspended particulate matter in the urban atmosphere of Krakow, Southern Poland. *Nukleonika* **65**, 181-191 (2020).
322. May, B. *et al.* The anthropogenic influence on carbonaceous aerosol in the European background. **61**, 464 (2009).
323. Simonova, G. V. *et al.* The atmospheric aerosol carbon isotope composition studies at the Svalbard and the Severnaya Zemlya archipelagos. in *26th International Symposium on Atmospheric and Ocean Optics,*

Atmospheric Physics **11560**, 1148-1160 (SPIE, 2020).
324. Wu, C. *et al.* The characteristics of atmospheric brown carbon in Xi'an, inland China: sources, size distributions and optical properties. *Atmospheric Chem. Phys.* **20**, 2017-2030 (2020).
325. Dusek, U. *et al.* The contribution of fossil sources to the organic aerosol in the Netherlands. *Atmos. Environ.* **74**, 169-176 (2013).
326. Yttri, K. E. *et al.* The EMEP intensive measurement period campaign, 2008-2009: Characterizing carbonaceous aerosol at nine rural sites in Europe. *Atmospheric Chem. Phys.* **19**, 4211-4233 (2019).
327. Marley, N. A. *et al.* The impact of biogenic carbon sources on aerosol absorption in Mexico City. *Atmospheric Chem. Phys.* **9**, 1537-1549 (2009).
328. Górka, M. *et al.* The impact of seasonality and meteorological conditions on $PM_{2.5}$ carbonaceous fractions coupled with carbon isotope analysis: Advantages, weaknesses and interpretation pitfalls. *Atmospheric Res.* **290**, 106800 (2023).
329. Liu, J. *et al.* The importance of non-fossil sources in carbonaceous aerosols in a megacity of central China during the 2013 winter haze episode: A source apportionment constrained by radiocarbon and organic tracers. *Atmos. Environ.* **144**, 60-68 (2016).
330. Ren, H. *et al.* The organic molecular composition, diurnal variation, and stable carbon isotope ratios of $PM_{2.5}$ in Beijing during the 2014 APEC summit. *Environ. Pollut.* **243**, 919-928 (2018).
331. Chesselet, R. *et al.* The origin of particulate organic carbon in the marine atmosphere as indicated by its stable carbon isotopic composition. *Geophys. Res. Lett.* **8**, 345-348 (1981).
332. Mo, Y. *et al.* The sources and atmospheric processes of strong light-absorbing components in water soluble brown carbon: Insights from a multi-proxy study of $PM_{2.5}$ in 10 Chinese cities. *J. Geophys. Res. Atmospheres* **129**, e2023JD039512 (2024).
333. Winiger, P. *et al.* The sources of atmospheric black carbon at a European gateway to the Arctic. *Nat. Commun.* **7**, 12776 (2016).
334. Norman, A. L. *et al.* The stable carbon isotope composition of atmospheric PAHs. *Atmos. Environ.* **33**, 2807-2814 (1999).
335. López-Veneroni, D. The stable carbon isotope composition of $PM_{2.5}$ and PM_{10} in Mexico City Metropolitan Area air. *Atmos. Environ.* **43**, 4491-4502 (2009).
336. Liu, D. *et al.* The use of levoglucosan and radiocarbon for source apportionment of $PM_{2.5}$ carbonaceous aerosols at a background site in East China. *Environ. Sci. Technol.* **47**, 10454-10461 (2013).
337. Tyagi, P. *et al.* Tracing atmospheric transport of soil microorganisms and higher plant waxes in the East Asian outflow to the North Pacific Rim by using hydroxy fatty acids: Year-round observations at Gosan, Jeju Island. *J. Geophys. Res. Atmospheres* **122**, 4112-4131 (2017).
338. Garbaras, A. Tracing of atmospheric aerosol sources using stable carbon isotopes. *Lith. J. Phys.* **48**, 259-264 (2008).
339. Bikkina, P. *et al.* Tracing the biomass burning emissions over the Arabian Sea in winter season: Implications from the molecular distributions and relative abundances of sugar compounds. *Sci. Total Environ.* **848**, 157643 (2022).
340. Bikkina, S. *et al.* Tracing the relative significance of primary versus secondary organic aerosols from biomass burning plumes over coastal ocean using sugar compounds and stable carbon isotopes. *ACS Earth Space Chem.* **3**, 1471-1484 (2019).
341. Buzek, F. *et al.* Tracking sources of PM_{10} emissions and deposition in the industrial city of Ostrava, Czech Republic: A carbonaceous δ^{13}C-based approach. *Atmos. Environ.* **295**, 119556 (2023).
342. Irei, S. *et al.* Transboundary secondary organic aerosol in Western Japan indicated by the δ^{13}C of water-soluble organic carbon and the m/z 44 signal in organic aerosol mass spectra. *Environ. Sci. Technol.* **48**, 6273-6281 (2014).
343. Irei, S. *et al.* Transboundary secondary organic aerosol in western Japan: An observed limitation of the *f*44 oxidation indicator. *Atmos. Environ.* **120**, 71-75 (2015).
344. Singh, G. K. *et al.* Understanding the origin of carbonaceous aerosols during periods of extensive biomass burning in northern India. *Environ. Pollut.* **270**, 116082 (2021).
345. Bikkina, P. *et al.* Unraveling the sources of atmospheric organic aerosols over the Arabian Sea: Insights from the stable carbon and nitrogen isotopic composition. *Sci. Total Environ.* **827**, 154260 (2022).
346. Mouteva, G. O. *et al.* Using radiocarbon to constrain black and organic carbon aerosol sources in Salt Lake City. *J. Geophys. Res. Atmospheres* **122**, 9843-9857 (2017).
347. Gensch, I. *et al.* Using δ^{13}C of levoglucosan as a chemical clock. *Environ. Sci. Technol.* **52**, 11094-11101 (2018).

348. Qadri, A. M. *et al.* Variabilities of $\delta^{13}C$ and carbonaceous components in ambient $PM_{2.5}$ in Northeast India: Insights into sources and atmospheric processes. *Environ. Res.* **214**, 113801 (2022).
349. Ho, K. F. *et al.* Variability of organic and elemental carbon, water soluble organic carbon, and isotopes in Hong Kong. *Atmospheric Chem. Phys.* **6**, 4569-4576 (2006).
350. Popova, S. A. *et al.* Variations in the concentrations of elements, carbon-containing particles and in carbon isotope composition in the Arctic aerosols at the Ice Base "Cape Baranov". in *27th International Symposium on Atmospheric and Ocean Optics, Atmospheric Physics* **11916**, 505-511 (SPIE, 2021).
351. Rybicki, M. *et al.* Variations in $\delta^{13}C$ values of levoglucosan from low-temperature burning of lignite and biomass. *Sci. Total Environ.* **733**, 138991 (2020).
352. Popova, S. A. *et al.* Variations of the carbon isotope composition and of organic and elemental carbon concentrations of the North Atlantic aerosols. in *25th International Symposium on Atmospheric and Ocean Optics: Atmospheric Physics* 11208 607-611 (SPIE, 2019).
353. Kirillova, E. N. *et al.* Water-soluble organic carbon aerosols during a full New Delhi winter: Isotope-based source apportionment and optical properties. *J. Geophys. Res. Atmospheres* **119**, 3476-3485 (2014).
354. Li, C. *et al.* Weak transport of atmospheric water-insoluble particulate carbon from South Asia to the inner Tibetan Plateau in the monsoon season. *Sci. Total Environ.* **922**, 171321 (2024).
355. Salam, A. *et al.* Wintertime air quality in Megacity Dhaka, Bangladesh strongly affected by influx of black carbon aerosols from regional biomass burning. *Environ. Sci. Technol.* **55**, 12243-12249 (2021).
356. Xu, J. *et al.* Wintertime organic and inorganic aerosols in Lanzhou, China: sources, processes, and comparison with the results during summer. *Atmospheric Chem. Phys.* **16**, 14937-14957 (2016).
357. Singh, G. K. *et al.* Wintertime study on bulk composition and stable carbon isotope analysis of ambient aerosols from North India. *J. Aerosol Sci.* **126**, 231-241 (2018).
358. Li, P. *et al.* Year-round observations of stable carbon isotopic composition of carboxylic acids, oxoacids and α-Dicarbonyls in fine aerosols at Tianjin, North China: Implications for origins and aging. *Sci. Total Environ.* **834**, 155385 (2022).
359. Barrett, T. E. & Sheesley, R. J. Year-round optical properties and source characterization of Arctic organic carbon aerosols on the North Slope Alaska. *J. Geophys. Res. Atmospheres* **122**, 9319-9331 (2017).
360. Garbaras, A. *et al.* $\delta^{13}C$ values in size-segregated atmospheric carbonaceous aerosols at a rural site in Lithuania. *Lith. J. Phys.* **49**, 229-236 (2009).
361. Zenker, K. *et al.* $\delta^{13}C$ signatures of organic aerosols: Measurement method evaluation and application in a source study. *J. Aerosol Sci.* **145**, 105534 (2020).
362. Irei, S. Isotopic Characterization of gaseous mercury and particulate water-soluble organic carbon emitted from open grass field burning in Aso, Japan. *Appl. Sci.* **12**, 109 (2022).
363. Shi, L. H. *et al.* Temporal variation of $PM_{2.5}$ and its carbon and nitrogen isotopic composition at an urban site in guangzhou in winter. *Environ. Monit. China* **39**, 81-91 (2023). In Chinese with English abstract
364. Wang, C. M. *et al.* Pollution characteristics and source analysis of carbon and nitrogen components in ambient $PM_{2.5}$ In Huangshi City. *Environ. Sci.* **44**, 626-633 (2023). In Chinese with English abstract
365. Huang, Q. W. *et al.* Stable isotopic compositions of carbon and nitrogen in aerosol samples collected from the Pingxiang City and their source apportionment. *Bull. Miner. Petrol. Geochem.* **38**, 114-120 (2019). In Chinese with English abstract
366. Mladenov, N. *et al.* Applications of optical spectroscopy and stable isotope analyses to organic aerosol source discrimination in an urban area. *Atmos. Environ.* **45**, 1960-1969 (2011).
367. Yuan, C. *et al.* Assessing the impacts of CPM emitted from stationary sources on $PM_{2.5}$ source appointment of Wuhan, China. *Fuel* **337**, 126869 (2023).
368. Xie, L. *et al.* Atmospheric deposition as a direct source of particulate organic carbon in region coastal surface seawater: Evidence from stable carbon and nitrogen isotope analysis. *Sci. Total Environ.* **854**, 158540 (2023).
369. Górka, M *et al.* Carbon and nitrogen isotope analyses coupled with palynological data of PM_{10} in Wrocław city (SW Poland) - assessment of anthropogenic impact. *Isotopes Environ. Health Stud.* **48**, 327-344 (2012).
370. Morera-Gómez, Y. *et al.* Carbon and nitrogen isotopes unravels sources of aerosol contamination at Caribbean rural and urban coastal sites. *Sci. Total Environ.* **642**, 723-732 (2018).
371. Shakya, K. M. *et al.* Characteristics and sources of carbonaceous, ionic, and isotopic species of wintertime atmospheric aerosols in Kathmandu Valley, Nepal. *Aerosol Air Qual. Res.* **10**, 219-230 (2010).
372. Sun, H. *et al.* Chemical characterizations and sources of $PM_{2.5}$ over the offshore Eastern China sea: Water soluble ions, stable isotopic compositions, and metal elements. *Atmospheric Pollut. Res.* **13**, 101410 (2022).
373. Chen, Y. *et al.* Chemical composition, structural properties, and source apportionment of organic macromolecules in atmospheric PM_{10} in a coastal city of Southeast China. *Environ. Sci. Pollut. Res.* **24**,

5877-5887 (2017).

374. Vercauteren, J. *et al.* Chemkar PM_{10}: An extensive look at the local differences in chemical composition of PM10 in Flanders, Belgium. *Atmos. Environ.* **45**, 108-116 (2011).
375. Wang, G. *et al.* Dicarboxylic acids, metals and isotopic compositions of C and N in atmospheric aerosols from inland China: implications for dust and coal burning emission and secondary aerosol formation. *Atmospheric Chem. Phys.* **10**, 6087-6096 (2010).
376. Kundu, S. *et al.* Diurnal variation in the water-soluble inorganic ions, organic carbon and isotopic compositions of total carbon and nitrogen in biomass burning aerosols from the LBA-SMOCC campaign in Rondônia, Brazil. *J. Aerosol Sci.* **41**, 118-133 (2010).
377. He, N. *et al.* Diurnal variations of carbonaceous components, major ions, and stable carbon and nitrogen isotope ratios in suburban aerosols from northern vicinity of Beijing. *Atmos. Environ.* **123**, 18-24 (2015).
378. Morera-Gómez, Y. *et al.* Elucidating the sources and dynamics of PM_{10} aerosols in Cienfuegos (Cuba) using their multi-stable and radioactive isotope and ion compositions. *Atmospheric Res.* **243**, 105038 (2020).
379. Xiao, Y. *et al.* Enhanced aerosols over the southeastern Tibetan Plateau induced by open biomass burning in spring 2020. *Sci. Total Environ.* **867**, 161509 (2023).
380. Liu, J. *et al.* Evidence of rural and suburban sources of urban haze formation in China: A case study from the Pearl River Delta Region. *J. Geophys. Res. Atmospheres* **123**, 4712-4726 (2018).
381. Lim, S. *et al.* Fossil-driven secondary inorganic $PM_{2.5}$ enhancement in the North China Plain: Evidence from carbon and nitrogen isotopes. *Environ. Pollut.* **266**, 115163 (2020).
382. Agnihotri, R. *et al.* Geochemical, stable isotopic, palynological characterization of surface dry soils and atmospheric particles over Jodhpur city (Thar Desert, Rajasthan) during peak summer of 2013. *MAPAN* **35**, 53-67 (2020).
383. Xiao, H. *et al.* Identifying the impacts of climate on the regional transport of haze pollution and inter-cities correspondence within the Yangtze River Delta. *Environ. Pollut.* **228**, 26-34 (2017).
384. Singh, G. K. *et al.* Insights into sources and atmospheric processing at two polluted urban locations in the Indo-Gangetic plains from stable carbon and nitrogen isotope ratios and polycyclic aromatic hydrocarbons in ambient $PM_{2.5}$. *Atmos. Environ.* **271**, 118904 (2022).
385. Plasencia Sánchez, E. *et al.* Integrating dual C and N isotopic approach to elemental and mathematical solutions for improving the PM source apportionment in complex urban and industrial cities: Case of Tarragona - Spain. *Atmos. Environ.* **293**, 119449 (2023).
386. Fernández-Amado, M. *et al.* Interrelationships between major components of PM_{10} and sub-micron particles: Influence of Atlantic air masses. *Atmospheric Res.* **212**, 64-76 (2018).
387. Dong, Z. *et al.* Measurement report: Chemical components and ^{13}C and ^{15}N isotope ratios of fine aerosols over Tianjin, North China: year-round observations. *Atmospheric Chem. Phys.* **23**, 2119-2143 (2023).
388. Dey, S. *et al.* Optical source apportionment of aqueous brown carbon (BrC) on a daytime and nighttime basis in the eastern Indo-Gangetic Plain (IGP) and insights from ^{13}C and ^{15}N isotopic signatures. *Sci. Total Environ.* **894**, 164872 (2023).
389. Hegde, P. *et al.* Organic and inorganic components of aerosols over the central Himalayas: winter and summer variations in stable carbon and nitrogen isotopic composition. *Environ. Sci. Pollut. Res.* **23**, 6102-6118 (2016).
390. Kawamura, K. *et al.* Organic and inorganic compositions of marine aerosols from East Asia: Seasonal variations of water-soluble dicarboxylic acids, major ions, total carbon and nitrogen, and stable C and N isotopic composition. *The Geochem. Soc. Special Publ.* **9**, 243-265 (2004).
391. Aggarwal, S. G. *et al.* Organic and inorganic markers and stable C-, N-isotopic compositions of tropical coastal aerosols from megacity Mumbai: sources of organic aerosols and atmospheric processing. *Atmospheric Chem. Phys.* **13**, 4667-4680 (2013).
392. Lim, S. *et al.* Robust evidence of ^{14}C, ^{13}C, and ^{15}N analyses indicating fossil fuel sources for total carbon and ammonium in fine aerosols in Seoul Megacity. *Environ. Sci. Technol.* **56**, 6894-6904 (2022).
393. Vodička, P. *et al.* Seasonal study of stable carbon and nitrogen isotopic composition in fine aerosols at a Central European rural background station. *Atmospheric Chem. Phys.* **19**, 3463-3479 (2019).
394. Mackey, K. R. M. *et al.* Seasonal variation of aerosol composition in Orange County, Southern California. *Atmos. Environ.* **244**, 117795 (2021).
395. Kawichai, S. *et al.* Source identification of $PM_{2.5}$ during a smoke haze period in Chiang Mai, Thailand, using stable carbon and nitrogen isotopes. *Atmosphere* **13**, 1149 (2022).
396. Lim, S. *et al.* Source signatures from combined isotopic analyses of $PM_{2.5}$ carbonaceous and nitrogen aerosols at the peri-urban Taehwa Research Forest, South Korea in summer and fall. *Sci. Total Environ.* **655**,

1505-1514 (2019).
397. Guo, X. *et al.* Sources of organic matter (PAHs and n-alkanes) in $PM_{2.5}$ of Beijing in haze weather analyzed by combining the C-N isotopic and PCA-MLR analyses. *Environ. Sci. Process. Impacts* **18**, 314-322 (2016).
398. Xiao, H. W. *et al.* Stable carbon and nitrogen isotope compositions of bulk aerosol samples over the South China Sea. *Atmos. Environ.* **193**, 1-10 (2018).
399. Sharma, S. K. *et al.* Stable carbon and nitrogen isotopic characteristics of $PM_{2.5}$ and PM_{10} in Delhi, India. *J. Atmospheric Chem.* **79**, 67-79 (2022).
400. Martinelli, L. A. *et al.* Stable carbon and nitrogen isotopic composition of bulk aerosol particles in a C4 plant landscape of southeast Brazil. *Atmos. Environ.* **36**, 2427-2432 (2002).
401. Agnihotri, R. *et al.* Stable carbon and nitrogen isotopic composition of bulk aerosols over India and northern Indian Ocean. *Atmos. Environ.* **45**, 2828-2835 (2011).
402. Bikkina, S. *et al.* Stable carbon and nitrogen isotopic composition of fine mode aerosols ($PM_{2.5}$) over the Bay of Bengal: impact of continental sources. **68**, 31518 (2016).
403. Sen, A. *et al.* Stable carbon and nitrogen isotopic composition of PM_{10} over Indo-Gangetic Plains (IGP), adjoining regions and Indo-Himalayan Range (IHR) during a winter 2014 campaign. *Environ. Sci. Pollut. Res.* **25**, 26279-26296 (2018).
404. Kunwar, B. *et al.* Stable carbon and nitrogen isotopic compositions of ambient aerosols collected from Okinawa Island in the western North Pacific Rim, an outflow region of Asian dusts and pollutants. *Atmos. Environ.* **131**, 243-253 (2016).
405. Boreddy, S. K. R. *et al.* Stable carbon and nitrogen isotopic compositions of fine aerosols ($PM_{2.5}$) during an intensive biomass burning over Southeast Asia: Influence of SOA and aging. *Atmos. Environ.* **191**, 478-489 (2018).
406. Mkoma, S. L. *et al*. Stable carbon and nitrogen isotopic compositions of tropical atmospheric aerosols: sources and contribution from burning of C3 and C4 plants to organic aerosols. **66**, 20176 (2014).
407. Zhang, H. *et al.* Stable isotopes unravel the sources and transport of $PM_{2.5}$ in the Yangtze River Delta, China. *Atmosphere* **14**, 1120 (2023).
408. Sharma, S. K. *et al.* Study on ambient air quality of Megacity Delhi, India during odd-even strategy. *MAPAN* **32**, 155-165 (2017).
409. Shi, Y. *et al.* The impact of emission reduction policies on the results of $PM_{2.5}$ emission sources during the 2016 G20 summit: Insights from carbon and nitrogen isotopic signatures. *Atmospheric Pollut. Res.* **14**, 101784 (2023).
410. Pavuluri, C. M. *et al.* Time-resolved distributions of bulk parameters, diacids, ketoacids and α-dicarbonyls and stable carbon and nitrogen isotope ratios of TC and TN in tropical Indian aerosols: Influence of land/sea breeze and secondary processes. *Atmospheric Res.* **153**, 188-199 (2015).
411. Sharma, S. K. *et al.* Variation of Stable Carbon and Nitrogen Isotopic Composition of PM_{10} at Urban Sites of Indo Gangetic Plain (IGP) of India. *Bull. Environ. Contam. Toxicol.* **95**, 661-669 (2015).
412. Park, Y. *et al.* Characterizing isotopic compositions of TC-C, NO_3^--N, and NH_4^+-N in $PM_{2.5}$ in South Korea: Impact of China's winter heating. *Environ. Pollut.* **233**, 735-744 (2018).
413. Sawlani, R. *et al.* Chemical and isotopic characteristics of $PM_{2.5}$ over New Delhi from September 2014 to May 2015: Evidences for synergy between air-pollution and meteorological changes. *Sci. Total Environ.* **763**, 142966 (2021).
414. Rastogi, N. *et al.* Chemical and isotopic characteristics of PM_{10} over the Bay of Bengal: Effects of continental outflow on a marine environment. *Sci. Total Environ.* **726**, 138438 (2020).
415. Agnihotri, R. *et al.* Stable isotopic and chemical characteristics of bulk aerosols during winter and summer seasons at a station in western coast of India (Goa). *Aerosol Air Qual. Res.* **15**, 888-900 (2015).
416. Sawlani, R. *et al.* The severe Delhi SMOG of 2016: A case of delayed crop residue burning, coincident firecracker emissions, and atypical meteorology. *Atmospheric Pollut. Res.* **10**, 868-879 (2019).
417. Widory, D. *et al.* The origin of atmospheric particles in Paris: a view through carbon and lead isotopes. *Atmos. Environ.* **38**, 953-961 (2004).
418. Chen, S. L. *et al.* Investigations on $PM_{2.5}$ sources in winter haze event in the northern suburb of Nanjing based on sulfurand carbon isotope. *Environ. Monit. China* **34**, 60-67 (2018). In Chinese wite English abstract
419.Song, L. *et al.* ^{15}N natural abundance of vehicular exhaust ammonia, quantified by active sampling techniques. *Atmos. Environ.* **255**, 118430 (2021).
420. Heaton, T. H. E. $^{15}N/^{14}N$ ratios of NO_x from vehicle engines and coal-fired power stations. **42**, 304 (1990).
421. Hideaki, H. *et al.* Nitrogen isotipic ratios of gaseous ammonia and annonium aerosols in the atmosphere. *J. Jpn. Soc. Atmos. Environ.* **39**, 272-279 (2004). In Japanese with English abstract

422. Jiang, M. *et al.* Source apportionment of ammonium in atmospheric $PM_{2.5}$ in the Pearl River Delta based on nitrogen isotope. *Ecol. Environ. Sci.* **31**, 1840-1848 (2022). In Chinese with English abstract
423. Xiang, Y. K. *et al.* Hypobromite oxidation combined with hydroxylamine hydrochloride reduction method for analyzing ammonium nitrogen isotope in atmospheric samples. *China J. Appl. Ecol.* **30**, 1847-1853 (2019). In Chinses with English abstract
424. Wen, Z. Q. *et al.* Concentration and composition characteristics of hydrolyzed amino acids in coarse aerosol in Nanchang. *Environ. Pollut. Control.* **43**, 1416-1420 (2021). In Chinses with English abstract
425. Zhu, W. Y. *et al.* The levels, sources and atmospheric photochemical processes of amino acids in $PM_{2.5}$ in forest areas. *China Environ. Sci.* **41**, 81-90 (2021). In Chinese with English abstract
426. Cao, Y. K. *et al.* Analysis of formation processes and sources of $PM_{2.5}$ ammonium during winter and summer in suburban area of the Yangtze River Delta. *Environ. Sci.* **44**, 6486-6494 (2023). In Chinese with English abstract
427. Wang, X. *et al.* Determination of ^{15}N natural abundance in nitrogen oxides from major anthropogenic emission sources. *Acta Pedol. Sin.* **53**, 1552-1562 (2016). In Chinese with English abstract
428. Altieri, K. E., Burger, J., Language, B. & Piketh, S. J. A case study in the wintertime Vaal Triangle Air-Shed Priority Area on the utility of the nitrogen stable isotopic composition of aerosol nitrate to identify NO_x sources. *Clean Air J.* **32**, (2022).
429. Wu, L. *et al.* Aerosol ammonium in the urban boundary layer in Beijing: Insights from nitrogen isotope ratios and simulations in summer 2015. *Environ. Sci. Technol. Lett.* **6**, 389-395 (2019).
430. Lin, C. T. *et al.* Aerosol isotopic ammonium signatures over the remote Atlantic Ocean. *Atmos. Environ.* **133**, 165-169 (2016).
431. Li, Y. *et al.* Apportioning atmospheric ammonia sources across spatial and seasonal scales by their isotopic fingerprint. *Environ. Sci. Technol.* **57**, 16424-16434 (2023).
432. Pavuluri, C. M. *et al.* Atmospheric chemistry of nitrogenous aerosols in northeastern Asia: biological sources and secondary formation. *Atmospheric Chem. Phys.* **15**, 9883-9896 (2015).
433. Feng, X. *et al.* Biomass burning is a non-negligible source for ammonium during winter haze episodes in rural North China: Evidence from high time resolution ^{15}N-stable isotope. *J. Geophys. Res. Atmospheres* **128**, e2022JD038012 (2023).
434. Zhou, Y. *et al.* Biomass burning related ammonia emissions promoted a self-amplifying loop in the urban environment in Kunming (SW China). *Atmos. Environ.* **253**, 118138 (2021).
435. Kelly, S. D. *et al.* Carbon and nitrogen isotopic analysis of atmospheric organic matter. *Atmos. Environ.* **39**, 6007-6011 (2005).
436. Ti, C. *et al.* Changes of $\delta^{15}N$ values during the volatilization process after applying urea on soil. *Environ. Pollut.* **270**, 116204 (2021).
437. David Felix, J. *et al.* Characterizing the isotopic composition of atmospheric ammonia emission sources using passive samplers and a combined oxidation-bacterial denitrifier approach. *Rapid Commun. Mass Spectrom.* **27**, 2239-2246 (2013).
438. Walters, W. W. *et al.* Characterizing the spatiotemporal nitrogen stable isotopic composition of ammonia in vehicle plumes. *Atmospheric Chem. Phys.* **20**, 11551-11567 (2020).
439. Singh, G. K. *et al.* Chemical characterization and stable nitrogen isotope composition of nitrogenous component of ambient aerosols from Kanpur in the Indo-Gangetic Plains. *Sci. Total Environ.* **763**, 143032 (2021).
440. Wen, Z. *et al.* Combined positive matrix factorization (PMF) and nitrogen isotope signature analysis to provide insights into the source contribution to aerosol free amino acids. *Atmos. Environ.* **268**, 118799 (2022).
441. Yeatman, S. G. *et al.* Comparisons of aerosol nitrogen isotopic composition at two polluted coastal sites. *Atmos. Environ.* **35**, 1307-1320 (2001).
442. Zhao, Z. Y. *et al.* Continental emissions influence the sources and formation mechanisms of marine nitrate aerosols in spring over the Bohai Sea and Yellow Sea inferred from stable isotopes. *J. Geophys. Res. Atmospheres* **129**, e2023JD040541 (2024).
443. Hall, S. J. *et al.* Convergence in nitrogen deposition and cryptic isotopic variation across urban and agricultural valleys in northern Utah. *J. Geophys. Res. Biogeosciences* **121**, 2340-2355 (2016).
444. Wang, C. *et al.* Developing nitrogen isotopic source profiles of atmospheric ammonia for source apportionment of ammonia in urban Beijing. *Front. Environ. Sci.* **10**, (2022).
445. Wu, C. *et al.* Different physicochemical behaviors of nitrate and ammonium during transport: A case study on Mt. Hua, China. *Atmospheric Chem. Phys.* **22**, 15621-15635 (2022).
446. Li, Q. *et al.* Diurnal and seasonal variations in water-soluble inorganic ions and nitrate dual isotopes of $PM_{2.5}$:

Implications for source apportionment and formation processes of urban aerosol nitrate. *Atmospheric Res.* **248**, 105197 (2021).
447. Wu, L. *et al.* Dominant contribution of combustion-related ammonium during haze pollution in Beijing. *Sci. Bull.* **69**, 978-987 (2024).
448. Baker, A. R. *et al.* Dry and wet deposition of nutrients from the tropical Atlantic atmosphere: Links to primary productivity and nitrogen fixation. *Deep Sea Res. Part Oceanogr. Res. Pap.* **54**, 1704-1720 (2007).
449. Pavuluri, C. M. *et al.* Elevated nitrogen isotope ratios of tropical Indian aerosols from Chennai: Implication for the origins of aerosol nitrogen in South and Southeast Asia. *Atmos. Environ.* **44**, 3597-3604 (2010).
450. Xiao, H. W. *et al.* Enhanced biomass burning as a source of aerosol ammonium over cities in central China in autumn. *Environ. Pollut.* **266**, 115278 (2020).
451. David Felix, J. *et al.* Examining the transport of ammonia emissions across landscapes using nitrogen isotope ratios. *Atmos. Environ.* **95**, 563-570 (2014).
452. Fibiger, D. L. & Hastings, M. G. First measurements of the nitrogen isotopic composition of NO_x from biomass burning. *Environ. Sci. Technol.* **50**, 11569-11574 (2016).
453. Zhang, Z. *et al.* Fossil fuel-related emissions were the major source of NH_3 pollution in urban cities of northern China in the autumn of 2017. *Environ. Pollut.* **256**, 113428 (2020).
454. Michalski, G. *et al.* Identifying NO_x sources in Arequipa, Peru using nitrogen isotopes in particulate nitrate. *Front. Environ. Sci.* **10**, (2022).
455. Kawashima, H. & Kurahashi, T. Inorganic ion and nitrogen isotopic compositions of atmospheric aerosols at Yurihonjo, Japan: Implications for nitrogen sources. *Atmos. Environ.* **45**, 6309-6316 (2011).
456. Zong, Z. *et al.* Insight into the variability of the nitrogen isotope composition of vehicular NO_x in China. *Environ. Sci. Technol.* **54**, 14246-14253 (2020).
457. Fan, M. Y. *et al.* Isotope-based source apportionment of nitrogen-containing aerosols: A case study in an industrial city in China. *Atmos. Environ.* **212**, 96-105 (2019).
458. Chen, X. *et al.* Isotopic characteristics and source analysis of atmospheric ammonia during agricultural periods in the Xichuan area of the Danjiangkou Reservoir. *J. Environ. Sci.* **136**, 460-469 (2024).
459. Ti, C. *et al.* Isotopic characterization of NH_x-N in deposition and major emission sources. *Biogeochemistry* **138**, 85-102 (2018).
460. Chai, J. *et al.* Isotopic characterization of nitrogen oxides (NO_x), nitrous acid (HONO), and nitrate (pNO_3^-) from laboratory biomass burning during FIREX. *Atmospheric Meas. Tech.* **12**, 6303-6317 (2019).
461. Miller, D. J. *et al.* Isotopic Composition of In Situ Soil NO_x Emissions in Manure-Fertilized Cropland. *Geophys. Res. Lett.* **45**, 12,058-12,066 (2018).
462. Felix, J. D. & Elliott, E. M. Isotopic composition of passively collected nitrogen dioxide emissions: Vehicle, soil and livestock source signatures. *Atmos. Environ.* **92**, 359-366 (2014).
463. Song, W. *et al.* Isotopic evaluation on relative contributions of major NO_x sources to nitrate of $PM_{2.5}$ in Beijing. *Environ. Pollut.* **248**, 183-190 (2019).
464.Pan, Y. *et al.* Isotopic evidence for enhanced fossil fuel sources of aerosol ammonium in the urban atmosphere. *Environ. Pollut.* **238**, 942-947 (2018).
465. Kawashima, H. *et al.* Long-term source apportionment of ammonium in $PM_{2.5}$ at a suburban and a rural site using stable nitrogen isotopes. *Environ. Sci. Technol.* **57**, 1268-1277 (2023).
466. Bekker, C. *et al.* Nitrate chemistry in the northeast US - Part 1: Nitrogen isotope seasonality tracks nitrate formation chemistry. *Atmospheric Chem. Phys.* **23**, 4185-4201 (2023).
467. Xiang, Y. K. *et al.* Nitrogen isotope characteristics and source apportionment of atmospheric ammonium in urban cities during a haze event in Northern China Plain. *Atmos. Environ.* **269**, 118800 (2022).
468. Wu, S. P. *et al.* Nitrogen isotope composition of ammonium in $PM_{2.5}$ in the Xiamen, China: impact of non-agricultural ammonia. *Environ. Sci. Pollut. Res.* **26**, 25596-25608 (2019).
469. Walters, W. W. *et al.* Nitrogen isotope composition of thermally produced NO_x from various fossil-fuel combustion sources. *Environ. Sci. Technol.* **49**, 11363-11371 (2015).
470. Liu, X. Y. *et al.* Nitrogen isotope differences between major atmospheric NO_y species: Implications for transformation and deposition processes. *Environ. Sci. Technol. Lett.* **7**, 227-233 (2020).
471. Bhattarai, N. *et al.* Nitrogen isotopes suggest agricultural and non-agricultural sources contribute equally to NH_3 and NH_4^+ in urban Beijing during December 2018. *Environ. Pollut.* **326**, 121455 (2023).
472. Widory, D. Nitrogen isotopes: Tracers of origin and processes affecting PM_{10} in the atmosphere of Paris. *Atmos. Environ.* **41**, 2382-2390 (2007).
473. Li, Z. *et al.* Nitrogen isotopic characteristics of aerosol ammonium in a Chinese megacity indicate the reduction from vehicle emissions during the lockdown period. *Sci. Total Environ.* **922**, 171265 (2024).

474. Felix, J. D. *et al.* Nitrogen Isotopic composition of coal-fired power plant NO_x: Influence of emission controls and implications for global emission inventories. *Environ. Sci. Technol.* **46**, 3528-3535 (2012).
475. Zhu, R. *et al.* Nitrogen isotopic composition of free Gly in aerosols at a forest site. *Atmos. Environ.* **222**, 117179 (2020).
476. Zong, Z. *et al.* Nitrogen isotopic composition of NO_x from residential biomass burning and coal combustion in North China. *Environ. Pollut.* **304**, 119238 (2022).
477. Li, D. & Wang, X. Nitrogen isotopic signature of soil-released nitric oxide (NO) after fertilizer application. *Atmos. Environ.* **42**, 4747-4754 (2008).
478. Bhattarai, H. *et al.* Nitrogen speciation and isotopic composition of aerosols collected at himalayan forest (3326 m a.s.l.): Seasonality, sources, and implications. *Environ. Sci. Technol.* **53**, 12247-12256 (2019).
479. Walters, W. W. *et al.* Nitrogen stable isotope composition ($\delta^{15}N$) of vehicle-emitted NO_x. *Environ. Sci. Technol.* **49**, 2278-2285 (2015).
480. Chen, Z. *et al.* Non-agricultural source dominates the ammonium aerosol in the largest city of South China based on the vertical $\delta^{15}N$ measurements. *Sci. Total Environ.* **848**, 157750 (2022).
481. Wu, C. *et al.* Non-agricultural sources dominate the atmospheric NH_3 in Xi'an, a megacity in the semi-arid region of China. *Sci. Total Environ.* **722**, 137756 (2020).
482. Yu, Z. & Elliott, E. M. Novel method for nitrogen isotopic analysis of soil-emitted nitric oxide. *Environ. Sci. Technol.* **51**, 6268-6278 (2017).
483. Luo, L. *et al.* Origins of aerosol nitrate in Beijing during late winter through spring. *Sci. Total Environ.* **653**, 776-782 (2019).
484. Zhou, J. *et al.* Pollution characterization and source identification of nitrogen-containing species in fine particulates: A case study in Hefei city $PM_{2.5}$, East China. *Chemosphere* **285**, 131316 (2021).
485. Zhou, X. *et al.* Precipitation frequency controls nitrogenous aerosol in a tropical coastal city and its implications for plant carbon sequestration. *Chemosphere* **326**, 138473 (2023).
486. Lin, X. *et al.* Proteinaceous Matter in Suburban Guiyang, Southwestern China: Decreased Importance in Long-Range Transport and Atmospheric Degradation. *J. Geophys. Res. Atmospheres* **128**, e2023JD038516 (2023).
487. Walters, W. W. *et al.* Quantifying the importance of vehicle ammonia emissions in an urban area of northeastern USA utilizing nitrogen isotopes. *Atmospheric Chem. Phys.* **22**, 13431-13448 (2022).
488. Zhou, X. *et al.* Regime shift in aerosol ammonium between the rainy and dry season: Perspective from stable isotopes in bulk deposition. *Atmospheric Pollut. Res.* **13**, 101462 (2022).
489. Freyer, H. D. Seasonal trends of NH_4^+ and NO_3^- nitrogen isotope composition in rain collected at Jülich, Germany. *Tellus* **30**, 83 (1978).
490. Kawashima, H. Seasonal trends of the stable nitrogen isotope ratio in particulate nitrogen compounds and their gaseous precursors in Akita, Japan. *Tellus B.* **71**, 1627846 (2019).
491. Freyer, H. D. Seasonal variation of $^{15}N/^{14}N$ ratios in atmospheric nitrate species. *Tellus B.* **43**, 30 (1991).
492. Kundu, S. *et al.* Seasonal variation of the concentrations of nitrogenous species and their nitrogen isotopic ratios in aerosols at Gosan, Jeju Island: Implications for atmospheric processing and source changes of aerosols. *J. Geophys. Res. Atmospheres* **115**, (2010).
493. Wang, Y. L. *et al.* Source appointment of nitrogen in $PM_{2.5}$ based on bulk $\delta^{15}N$ signatures and a Bayesian isotope mixing model. *Tellus B.* **69**, 1299672 (2017).
494. Pan, Y. *et al.* Source Apportionment of aerosol ammonium in an ammonia-rich atmosphere: An isotopic study of summer clean and hazy days in urban Beijing. *J. Geophys. Res. Atmospheres* **123**, 5681-5689 (2018).
495. Zhang, Y. *et al.* Source apportionment of atmospheric ammonia at 16 sites in China using a bayesian isotope mixing model based on $\delta^{15}N$-NH_x Signatures. *Environ. Sci. Technol.* **57**, 6599-6608 (2023).
496. Zhu, R. *et al.* Sources and transformation processes of proteinaceous matter and free amino acids in $PM_{2.5}$. *J. Geophys. Res. Atmospheres* **125**, e2020JD032375 (2020).
497. Kawashima, H. *et al.* Sources identification of ammonium in $PM_{2.5}$ during monsoon season in Dhaka, Bangladesh. *Sci. Total Environ.* **838**, 156433 (2022).
498. Bhattarai, N. *et al.* Sources of gaseous NH_3 in urban Beijing from parallel sampling of NH_3 and NH_4^+, their nitrogen isotope measurement and modeling. *Sci. Total Environ.* **747**, 141361 (2020).
499. Wiedenhaus, H. *et al.* Stable ^{15}N isotopes in fine and coarse urban particulate matter. *Aerosol Sci. Technol.* **55**, 859-870 (2021).
500. Shi, Y. *et al.* Stable nitrogen isotope composition of NO_x of biomass burning in China. *Sci. Total Environ.* **803**, 149857 (2022).
501. Guo, W. *et al.* Stable nitrogen isotopic signatures reveal the NH_4^+ evolution processes in pollution episodes

in urban southwestern China. *Atmospheric Res.* **253**, 105474 (2021).

502. Moore, H. The isotopic composition of ammonia, nitrogen dioxide and nitrate in the atmosphere. *Atmospheric Environ. 1967* **11**, 1239-1243 (1977).
503. Swap, R. *et al.* The long-range transport of southern African aerosols to the tropical South Atlantic. *J. Geophys. Res. Atmospheres* **101**, 23777-23791 (1996).
504. Miller, D. J. *et al.* Vehicle NO_x emission plume isotopic signatures: Spatial variability across the eastern United States. *J. Geophys. Res. Atmospheres* **122**, 4698-4717 (2017).
505. Fan, M. Y. *et al.* Vertical differences of nitrate sources in urban boundary layer based on tower measurements. *Environ. Sci. Technol. Lett.* **9**, 906-912 (2022).
506. Savard, M. M. *et al.* δ^{15}N values of atmospheric N species simultaneously collected using sector-based samplers distant from sources - Isotopic inheritance and fractionation. *Atmos. Environ.* **162**, 11-22 (2017).
507. Yang, S. D. *et al.* Dry deposition fluxes, formation mechanisms and sources of nitrate in total suspended particles in springtime on Dongsha Island, South China Sea. *J. Earth Environ.* **14**, 193-206 (2023). In Chinese with English abstract
508. Burger, J. M. *et al.* A seasonal analysis of aerosol NO_3^- sources and NO_x oxidation pathways in the Southern Ocean marine boundary layer. *Atmospheric Chem. Phys.* **23**, 5605-5622 (2023).
509. Zong, Z. *et al.* Assessment and quantification of NO_x sources at a regional background site in North China: Comparative results from a Bayesian isotopic mixing model and a positive matrix factorization model. *Environ. Pollut.* **242**, 1379-1386 (2018).
510. Luo, L. *et al.* Assessment of the seasonal cycle of nitrate in $PM_{2.5}$ using chemical compositions and stable nitrogen and oxygen isotopes at Nanchang, China. *Atmos. Environ.* **225**, 117371 (2020).
511. Zhang, Y. *et al.* Changes in atmospheric oxidants over Arctic Ocean atmosphere: evidence of oxygen isotope anomaly in nitrate aerosols. *Npj Clim. Atmospheric Sci.* **6**, 1-9 (2023).
512. Luo, L. *et al.* Changes in nitrate accumulation mechanisms as $PM_{2.5}$ levels increase on the North China Plain: A perspective from the dual isotopic compositions of nitrate. *Chemosphere* **263**, 127915 (2021).
513. Fan, M. Y. *et al.* Changes of emission sources to nitrate aerosols in Beijing after the clean air actions: Evidence from dual isotope compositions. *J. Geophys. Res. Atmospheres* **125**, e2019JD031998 (2020).
514. Zhao, Z. Y. *et al.* Coal and biomass burning as major emissions of NO_x in Northeast China: Implication from dual isotopes analysis of fine nitrate aerosols. *Atmos. Environ.* **242**, 117762 (2020).
515. Zhang, W. *et al.* Diesel vehicle emission accounts for the dominate NO_x source to atmospheric particulate nitrate in a coastal city: Insights from nitrate dual isotopes of $PM_{2.5}$. *Atmospheric Res.* **278**, 106328 (2022).
516. Elliott, E. M. *et al.* Dual nitrate isotopes in dry deposition: Utility for partitioning NO_x source contributions to landscape nitrogen deposition. *J. Geophys. Res. Biogeosciences* **114**, (2009).
517. Zong, Z. *et al.* Dual-modelling-based source apportionment of NO_x in five Chinese megacities: Providing the isotopic footprint from 2013 to 2014. *Environ. Int.* **137**, 105592 (2020).
518. Lin, Y. C. *et al.* Formation mechanisms and source apportionments of airborne nitrate aerosols at a Himalayan-Tibetan Plateau site: Insights from nitrogen and oxygen isotopic compositions. *Environ. Sci. Technol.* **55**, 12261-12271 (2021).
519. Fan, M. Y. *et al.* Formation mechanisms and source apportionments of nitrate aerosols in a megacity of Eastern China based on multiple isotope observations. *J. Geophys. Res. Atmospheres* **128**, e2022JD038129 (2023).
520. Xi, D. *et al.* Formation pathways and source apportionments of inorganic nitrogen-containing aerosols in urban environment: Insights from nitrogen and oxygen isotopic compositions in Guangzhou, China. *Atmos. Environ.* **309**, 119888 (2023).
521. Yan, X. *et al.* Investigating atmospheric nitrate sources and formation pathways between heating and non-heating seasons in urban North China. *Environ. Res. Lett.* **18**, 034006 (2023).
522. Zhang, K. *et al.* Isotopic components and source analysis of inorganic nitrogen in coastal aerosols of the Yellow Sea. *Front. Mar. Sci.* **9**, (2022).
523. Zong, Z. *et al.* Isotopic interpretation of particulate nitrate in the Metropolitan City of Karachi, Pakistan: Insight into the oceanic contribution to NO_x. *Environ. Sci. Technol.* **54**, 7787-7797 (2020).
524. Guha, T. *et al.* Isotopic ratios of nitrate in aerosol samples from Mt. Lulin, a high-altitude station in Central Taiwan. *Atmos. Environ.* **154**, 53-69 (2017).
525. Zhao, Z. Y. *et al.* Nitrate aerosol formation and source assessment in winter at different regions in Northeast China. *Atmos. Environ.* **267**, 118767 (2021).
526. Kim, H. *et al.* Nitrate chemistry in the northeast US - Part 2: Oxygen isotopes reveal differences in particulate and gas-phase formation. *Atmospheric Chem. Phys.* **23**, 4203-4219 (2023).

527. Deng, M. *et al.* Nitrogen and oxygen isotope characteristics, formation mechanism, and source apportionment of nitrate aerosols in Wuhan, Central China. *Sci. Total Environ.* **921**, 170715 (2024).
528. Savarino, J. *et al.* Nitrogen and oxygen isotopic constraints on the origin of atmospheric nitrate in coastal Antarctica. *Atmospheric Chem. Phys.* **7**, 1925-1945 (2007).
529. Chang, Y. *et al.* Nitrogen isotope fractionation during gas-to-particle conversion of NO_x to NO_3^- in the atmosphere - implications for isotope-based NO_x source apportionment. *Atmospheric Chem. Phys.* **18**, 11647-11661 (2018).
530. Zhang, Z. *et al.* Oxidation and sources of atmospheric NO_x during winter in Beijing based on $\delta^{18}O$-$\delta^{15}N$ space of particulate nitrate. *Environ. Pollut.* **276**, 116708 (2021).
531. Lim, S. *et al.* Oxidation pathways and emission sources of atmospheric particulate nitrate in Seoul: based on $\delta^{15}N$ and $\Delta^{17}O$ measurements. *Atmospheric Chem. Phys.* **22**, 5099-5115 (2022).
532. Zhang, W. *et al.* Quantification of NO_x sources contribution to ambient nitrate aerosol, uncertainty analysis and sensitivity analysis in a megacity. *Sci. Total Environ.* **926**, 171583 (2024).
533. Jin, Z. *et al.* Quantifying major NO_x sources of aerosol nitrate in Hangzhou, China, by using stable isotopes and a Bayesian isotope mixing model. *Atmos. Environ.* **244**, 117979 (2021).
534. Cheng, C. *et al.* Quantifying the source and formation of nitrate in $PM_{2.5}$ using dual isotopes combined with Bayesian mixing model: A case study in an inland city of southeast China. *Chemosphere* **308**, 136097 (2022).
535. Zhang, Z. *et al.* Rayleigh based concept to track NO_x emission sources in urban areas of China. *Sci. Total Environ.* **704**, 135362 (2020).
536. Li, Y. *et al.* Seasonal differences in sources and formation processes of $PM_{2.5}$ nitrate in an urban environment of North China. *J. Environ. Sci.* **120**, 94-104 (2022).
537. Wang, X. *et al.* Sources and formation of atmospheric nitrate over China-Indochina Peninsula in spring: A perspective from oxygen and nitrogen isotopic compositions based on passive air samplers. *Front. Environ. Sci.* **10**, (2022).
538. Wankel, S. D. *et al.* Sources of aerosol nitrate to the Gulf of Aqaba: Evidence from $\delta^{15}N$ and $\delta^{18}O$ of nitrate and trace metal chemistry. *Mar. Chem.* **120**, 90-99 (2010).
539. Kim, H. *et al.* Stable isotope ratio of atmospheric and seawater nitrate in the East Sea in the northwestern Pacific ocean. *Mar. Pollut. Bull.* **149**, 110610 (2019).
540. Xiao, H. *et al.* Strict control of biomass burning inhibited particulate matter nitrate pollution over Tianjin: Perspective from dual isotopes of nitrate. *Atmos. Environ.* **293**, 119460 (2023).
541. Guo, W. *et al.* The use of stable oxygen and nitrogen isotopic signatures to reveal variations in the nitrate formation pathways and sources in different seasons and regions in China. *Environ. Res.* **201**, 111537 (2021).
542. Xiao, H. W. *et al.* Use of isotopic compositions of nitrate in TSP to identify sources and chemistry in South China Sea. *Atmos. Environ.* **109**, 70-78 (2015).
543. Wang, Y. *et al.* Vertical measurements of stable nitrogen and oxygen isotope composition of fine particulate nitrate aerosol in Guangzhou city: Source apportionment and oxidation pathway. *Sci. Total Environ.* **865**, 161239 (2023).
544. Wen, L. R. *et al.* Source apportionment of ammonium in atmospheric $PM_{2.5}$ in the Pearl River Delta based on nitrogen isotope. *Ecol. Environ. Sci.* **32**, 1654-1662 (2023). In Chinese with English abstract
545. Proemse, B. C. *et al.* Isotopic characterization of nitrate, ammonium and sulfate in stack $PM_{2.5}$ emissions in the Athabasca Oil Sands Region, Alberta, Canada. *Atmos. Environ.* **60**, 555-563 (2012).
546. Han, X. *et al.* Sulfur sources and oxidation pathways in summer aerosols from Nanjing northern suburbs using S and O Isotopes. *Environ. Sci.* **39**, 2010-2014 (2018). In Chinese with English abstract
547. He, P. Z. & Xie, Z. Q. Using oxygen isotopes to trace the formation pathways of atmospheric nitrate over summer Arctic Ocean (62.3°-74.7°N). *J. Glaciol. Geocryol.* **43**, 1344-1353 (2021). In Chinese with English abstract
548. Liang, Y. *et al.* Chemical characteristics and sources of $PM_{2.5}$ and water-soluble ions in autumn and winter in central China. *Environ. Chem.* **41**, 470-481 (2022). In Chinese with English abstract
549. Lin, M. *et al.* A complete isotope ($\delta^{15}N$, $\delta^{18}O$, $\Delta^{17}O$) investigation of atmospherically deposited nitrate in glacial-hydrologic systems across the third pole region. *J. Geophys. Res. Atmospheres* **125**, e2019JD031878 (2020).
550. Zhang, Y. L. *et al.* A diurnal story of $\Delta^{17}O(NO_3^-)$ in urban Nanjing and its implication for nitrate aerosol formation. *Npj Clim. Atmospheric Sci.* **5**, 1-10 (2022).
551. Bigio, L. *et al.* Are the phosphate oxygen isotopes of Saharan dust a robust tracer of atmospheric P source? *Atmos. Environ.* **235**, 117561 (2020).
552. Wang, Y. Q. *et al.* Characteristics of carbonate content and carbon and oxygen isotopic composition of

northern China soil and dust aerosol and its application to tracing dust sources. *Atmos. Environ.* **39**, 2631-2642 (2005).

553. Morin, S. *et al.* Comprehensive isotopic composition of atmospheric nitrate in the Atlantic Ocean boundary layer from 65°S to 79°N. *J. Geophys. Res. Atmospheres* **114**, (2009).
554. Shaheen, R. *et al.* Detection of oxygen isotopic anomaly in terrestrial atmospheric carbonates and its implications to Mars. *Proc. Natl. Acad. Sci.* **107**, 20213-20218 (2010).
555. Hill-Falkenthal, J. *et al.* Differentiating sulfate aerosol oxidation pathways for varying source altitudes using ^{35}S and Δ^{17}O tracers. *J. Geophys. Res. Atmospheres* **117**, (2012).
556. Dominguez, G. *et al.* Discovery and measurement of an isotopically distinct source of sulfate in Earth's atmosphere. *Proc. Natl. Acad. Sci.* **105**, 12769-12773 (2008).
557. Zhang, Z. *et al.* Dominance of heterogeneous chemistry in summertime nitrate accumulation: Insights from oxygen isotope of nitrate (δ^{18}O-NO_3^-). *ACS Earth Space Chem.* **4**, 818-824 (2020).
558. Chen, B. *et al.* Dust transport from northeastern China inferred from carbon isotopes of atmospheric dust carbonate. *Atmos. Environ.* **42**, 4790-4796 (2008).
559. Zong, Z. *et al.* First Assessment of NO_x sources at a regional background site in North China using isotopic analysis linked with modeling. *Environ. Sci. Technol.* **51**, 5923-5931 (2017).
560. Michalski, G. *et al.* First measurements and modeling of Δ^{17}O in atmospheric nitrate. *Geophys. Res. Lett.* **30**, (2003).
561. Katsura, H. Historically heavy rainfalls in Singapore on 19 November 2009 and 17 July 2010. *Asian J. Chem.* **25**, 2116-2120 (2013).
562. Cao, Y. *et al.* How aerosol pH responds to nitrate to sulfate ratio of fine-mode particulate. *Environ. Sci. Pollut. Res.* **27**, 35031-35039 (2020).
563. Zong, Z. *et al.* Impact of an accidental explosion in Tianjin Port on enhanced atmospheric nitrogen deposition over the Bohai Sea inferred from aerosol nitrate dual isotopes. *Atmospheric Ocean. Sci. Lett.* **13**, 195-201 (2020).
564. Zhang, Z. *et al.* Importance of NO_3 radical in particulate nitrate formation in a southeast Chinese urban city: New constraints by δ^{15}N-δ^{18}O space of NO_3^-. *Atmos. Environ.* **253**, 118387 (2021).
565. Wu, C. *et al.* Important contribution of N_2O_5 hydrolysis to the daytime nitrate in Xi'an, China during haze periods: Isotopic analysis and WRF-Chem model simulation. *Environ. Pollut.* **288**, 117712 (2021).
566. Fan, M. Y. *et al.* Important role of NO_3 radical to nitrate formation aloft in urban Beijing: Insights from triple oxygen isotopes measured at the tower. *Environ. Sci. Technol.* **56**, 6870-6879 (2022).
567. Wang, Y. L. *et al.* Influences of atmospheric pollution on the contributions of major oxidation pathways to $PM_{2.5}$ nitrate formation in Beijing. *J. Geophys. Res. Atmospheres* **124**, 4174-4185 (2019).
568. Wang, K. *et al.* Isotopic constraints on atmospheric sulfate formation pathways in the Mt. Everest region, southern Tibetan Plateau. *Atmospheric Chem. Phys.* **21**, 8357-8376 (2021).
569. He, P. *et al.* Isotopic constraints on heterogeneous sulfate production in Beijing haze. *Atmospheric Chem. Phys.* **18**, 5515-5528 (2018).
570. McCabe, J. R. *et al.* Isotopic constraints on non-photochemical sulfate production in the Arctic winter. *Geophys. Res. Lett.* **33**, (2006).
571. Chen, Q. *et al.* Isotopic constraints on the role of hypohalous acids in sulfate aerosol formation in the remote marine boundary layer. *Atmospheric Chem. Phys.* **16**, 11433-11450 (2016).
572. Song, W. *et al.* Nitrogen isotope differences between atmospheric nitrate and corresponding nitrogen oxides: A new constraint using oxygen isotopes. *Sci. Total Environ.* **701**, 134515 (2020).
573. Mather, T. A. *et al.* Oxygen and sulfur isotopic composition of volcanic sulfate aerosol at the point of emission. *J. Geophys. Res. Atmospheres* **111**, (2006).
574. Blees, J. *et al.* Oxygen isotope analysis of levoglucosan, a tracer of wood burning, in experimental and ambient aerosol samples. *Rapid Commun. Mass Spectrom.* **31**, 2101-2108 (2017).
575. Bigio, L. & Angert, A. Oxygen isotope signatures of phosphate in wildfire ash. *ACS Earth Space Chem.* **3**, 760-769 (2019).
576. Carter, T. S. *et al.* Quantifying nitrate formation pathways in the equatorial pacific atmosphere from the GEOTRACES Peru-Tahiti transect. *ACS Earth Space Chem.* **5**, 2638-2651 (2021).
577. Zhang, Z. *et al.* Response of fine aerosol nitrate chemistry to Clean Air Action in winter Beijing: Insights from the oxygen isotope signatures. *Sci. Total Environ.* **746**, 141210 (2020).
578. Itahashi, S. *et al.* Role of dust and iron solubility in sulfate formation during the long-range transport in East Asia evidenced by ^{17}O-excess signatures. *Environ. Sci. Technol.* **56**, 13634-13643 (2022).
579. Ishino, S. *et al.* Seasonal variations of triple oxygen isotopic compositions of atmospheric sulfate, nitrate,

and ozone at Dumont d'Urville, coastal Antarctica. *Atmospheric Chem. Phys.* **17**, 3713-3727 (2017).
580. Zhang, Z. *et al.* Sources and transformation of nitrate aerosol in winter 2017-2018 of megacity Beijing: Insights from an alternative approach. *Atmos. Environ.* **241**, 117842 (2020).
581. Cao, J. J. *et al.* Stable carbon and oxygen isotopic composition of carbonate in fugitive dust in the Chinese Loess Plateau. *Atmos. Environ.* **42**, 9118-9122 (2008).
582. Luo, L. *et al.* Stable oxygen isotope constraints on nitrate formation in Beijing in springtime. *Environ. Pollut.* **263**, 114515 (2020).
583. Alexander, B. *et al.* Sulfate formation in sea-salt aerosols: Constraints from oxygen isotopes. *J. Geophys. Res. Atmospheres* **110**, (2005).
584. He, P. *et al.* The observation of isotopic compositions of atmospheric nitrate in Shanghai China and its implication for reactive nitrogen chemistry. *Sci. Total Environ.* **714**, 136727 (2020).
585. Lee, C. C. W. & Thiemens, M. H. The $\delta^{17}O$ and $\delta^{18}O$ measurements of atmospheric sulfate from a coastal and high alpine region: A mass-independent isotopic anomaly. *J. Geophys. Res. Atmospheres* **106**, 17359-17373 (2001).
586. Savard, M. M. *et al.* The $\Delta^{17}O$ and $\delta^{18}O$ values of atmospheric nitrates simultaneously collected downwind of anthropogenic sources - implications for polluted air masses. *Atmospheric Chem. Phys.* **18**, 10373-10389 (2018).
587. Gross, A. *et al.* Tracing the sources of atmospheric phosphorus deposition to a tropical rain forest in Panama using stable oxygen isotopes. *Environ. Sci. Technol.* **50**, 1147-1156 (2016).
588. Gillette, D. A. *et al.* Tropospheric aerosols from some major dust storms of the Southwestern United States. *J. Appl. Meteorol. Climatol.* **17**, 832-845 (1978).
589. Wei, Y. *et al.* Tracing sources of sulfate aerosol in Nanjing northern suburb using sulfur and oxygen isotopes. *Environ. Sci* **36**, 1182-1186 (2015). In Chinese with English abstract
590. Norman, A. L. *et al.* Aerosol sulphate and its oxidation on the Pacific NW coast: S and O isotopes in $PM_{2.5}$. *Atmos. Environ.* **40**, 2676-2689 (2006).
591. Han, X. *et al.* Effect of the pollution control measures on $PM_{2.5}$ during the 2015 China Victory Day Parade: Implication from water-soluble ions and sulfur isotope. *Environ. Pollut.* **218**, 230-241 (2016).
592. Li, T. *et al.* In situ biomass burning enhanced the contribution of biogenic sources to sulfate aerosol in subtropical cities. *Sci. Total Environ.* **908**, 168384 (2024).
593. Patris, N. *et al.* Isotopic analysis of aerosol sulfate and nitrate during ITCT-2k2: Determination of different formation pathways as a function of particle size. *J. Geophys. Res. Atmospheres* **112**, (2007).
594. Guo, Z. *et al*. Multiple sulfur and oxygen isotope compositions in Beijing aerosol. *Sci. China Earth Sci.* **57**, 2671-2675 (2014).
595. Han, X. *et al.* Using stable isotopes to trace sources and formation processes of sulfate aerosols from Beijing, China. *Sci. Rep.* **6**, 29958 (2016).
596. Yao, W. H. *et al.* The research on the environmental significance of atmospheric sulfur isotopic composition in Hengyang. *Res. Environ. Sci*. 3-5 (2003). In Chinese with English abstract
597. Zhang, H. B. *et al.* Sulfur isotopic composition of acid deposition in South China Regions and its environmental significance. *China Environ. Sci* 70-74 (2002). In Chinese with English abstract
598. Guo, Z. B. *et al*. Analysis of sulfur isotopes in Taihu aerosol. *Geochimica* **41**, 411-414 (2012). In Chinese with English abstract
599 Takao, H. *et al.* Sulfur isotope ratios of coals and oils used in China and Japan. *Nippon Kagaku Kaishi* **2000**, 45-51 (2000). In Japanese with English abstract
600. Zhou, Y. *et al.* Aerosol pollution in a megacity of southwest China inferred from variation characteristics of sulfate-$\delta^{34}S$ and water-soluble inorganic compositions in TSP. *Particuology* **43**, 202-209 (2019).
601. McArdle, N. *et al.* An isotopic study of atmospheric sulphur at three sites in Wales and at Mace Head, Eire. *J. Geophys. Res. Atmospheres* **103**, 31079-31094 (1998).
602. Lin, Y. C. *et al.* Anthropogenic emission sources of sulfate aerosols in Hangzhou, East China: Insights from isotope techniques with consideration of fractionation effects between gas-to-particle transformations. *Environ. Sci. Technol.* **56**, 3905-3914 (2022).
603. Turekian, V. C. *et al.* Application of stable sulfur isotopes to differentiate sources of size-resolved Particulate sulfate in polluted marine air at Bermuda during spring. *Geophys. Res. Lett.* **28**, 1491-1494 (2001).
604. Nriagu, J. O. *et al.* Biogenic sulfur and the acidity of rainfall in remote areas of Canada. *Science* **237**, 1189-1192 (1987).
605. Sakata, M. *et al.* Contribution of Asian outflow to atmospheric concentrations of sulfate and trace elements in aerosols during winter in Japan. *Geochem. J.* **48**, 479-490 (2014).

606. Sakata, M. *et al.* Effectiveness of sulfur and boron isotopes in aerosols as tracers of emissions from coal burning in Asian continent. *Atmos. Environ.* **67**, 296-303 (2013).
607. Han, X. *et al.* Enhanced oxidation of SO_2 by H_2O_2 during haze events: Constraints from sulfur isotopes. *J. Geophys. Res. Atmospheres* **127**, e2022JD036960 (2022).
608. Feng, X. *et al.* Exploring the influence of ^{34}S fractionation from emission sources and SO_2 atmospheric oxidation on sulfate source apportionment based on hourly resolution $\delta^{34}S\text{-}SO_4^{2-}$. *J. Geophys. Res. Atmospheres* **128**, e2023JD038595 (2023).
609. Guo, Z. *et al.* Identification of sources and formation processes of atmospheric sulfate by sulfur isotope and scanning electron microscope measurements. *J. Geophys. Res. Atmospheres* **115**, (2010).
610. Li, J. *et al.* Investigating Source Contributions of Size-Aggregated Aerosols Collected in Southern Ocean and Baring Head, New Zealand Using Sulfur Isotopes. *Geophys. Res. Lett.* **45**, 3717-3727 (2018).
611. McArdle, N. C. & Liss, P. S. Isotopes and atmospheric sulphur. *Atmos. Environ.* **29**, 2553-2556 (1995).
612. Ding, T. *et al.* Mass independent sulfur isotope fractionation discovered in aerosol of Beijing. *Geochim. Cosmochim. Acta* **70**, A142 (2006).
613. Romero, A. B. & Thiemens, M. H. Mass-independent sulfur isotopic compositions in present-day sulfate aerosols. *J. Geophys. Res. Atmospheres* **108**, (2003).
614. Olson, E. *et al.* Mineral dust and fossil fuel combustion dominate sources of aerosol sulfate in urban Peru identified by sulfur stable isotopes and water-soluble ions. *Atmos. Environ.* **260**, 118482 (2021).
615. Han, X. *et al.* Multiple Sulfur isotope constraints on sources and formation processes of sulfate in Beijing $PM_{2.5}$ aerosol. *Environ. Sci. Technol.* **51**, 7794-7803 (2017).
616. Tostevin, R. *et al.* Multiple sulfur isotope constraints on the modern sulfur cycle. *Earth Planet. Sci. Lett.* **396**, 14-21 (2014).
617. Han, X. *et al.* Multiple sulfur isotopic evidence for sulfate formation in haze pollution. *Environ. Sci. Technol.* **57**, 20647-20656 (2023).
618. Nriagu, J. O. *et al*. Origin of sulphur in Canadian Arctic haze from isotope measurements. *Nature* **349**, 142-145 (1991).
619. Tichomirowa, M. *et al.* Regional and temporal (1992-2004) evolution of air-borne sulphur isotope composition in Saxony, southeastern Germany, central Europe. *Isotopes Environ. Health Stud.* **43**, 295-305 (2007).
620. Wang, X. *et al.* Regional characteristics of atmospheric $\delta^{34}S\text{-}SO_4^{2-}$ over three parts of Asia monitored by quartz wool-based passive samplers. *Sci. Total Environ.* **778**, 146107 (2021).
621. Fan, M. Y. *et al.* Roles of sulfur oxidation pathways in the variability in stable sulfur isotopic composition of sulfate aerosols at an urban site in Beijing, China. *Environ. Sci. Technol. Lett.* **7**, 883-888 (2020).
622. Seguin, A. M. *et al.* Seasonality in size segregated biogenic, anthropogenic and sea salt sulfate aerosols over the North Atlantic. *Atmos. Environ.* **45**, 6947-6954 (2011).
623. Au Yang, D. *et al.* Seasonality in the $\Delta^{33}S$ measured in urban aerosols highlights an additional oxidation pathway for atmospheric SO_2. *Atmospheric Chem. Phys.* **19**, 3779-3796 (2019).
624. Xiao, H. Y. *et al.* Source identification of sulfur in uncultivated surface soils from four Chinese provinces. *Pedosphere* **25**, 140-149 (2015).
625.Wei, L. *et al.* Stable sulfur isotope ratios and chemical compositions of fine aerosols ($PM_{2.5}$) in Beijing, China. *Sci. Total Environ.* **633**, 1156-1164 (2018).
626. Yang, Z. *et al.* Stable sulfur isotope ratios and water-soluble inorganic compositions of PM_{10} in Yichang City, central China. *Environ. Sci. Pollut. Res.* **22**, 13564-13572 (2015).
627. Li, J. *et al.* Stable Sulfur isotopes revealed a major role of transition-metal ion-catalyzed SO_2 oxidation in haze episodes. *Environ. Sci. Technol.* **54**, 2626-2634 (2020).
628. Guo, Z. *et al.* Study on pollution behavior and sulfate formation during the typical haze event in Nanjing with water soluble inorganic ions and sulfur isotopes. *Atmospheric Res.* **217**, 198-207 (2019).
629. Chung, C. H. *et al.* Sulfur isotope analysis for representative regional background atmospheric aerosols collected at Mt. Lulin, Taiwan. *Sci. Rep.* **9**, 19707 (2019).
630. Dasari, S. *et al.* Sulfur isotope anomalies ($\Delta^{33}S$) in urban air pollution linked to mineral-dust-associated sulfate. *Environ. Sci. Technol. Lett.* **9**, 604-610 (2022).
631. Amrani, A. *et al.* Sulfur isotope homogeneity of oceanic DMSP and DMS. *Proc. Natl. Acad. Sci.* **110**, 18413-18418 (2013).
632. Calhoun, J. A. *et al.* Sulfur isotope measurements of submicrometer sulfate aerosol particles over the Pacific Ocean. *Geophys. Res. Lett.* **18**, 1877-1880 (1991).
633. Ludwig, F. L. Sulfur isotope ratios and the origins of the aerosols and cloud droplets in California stratus.

Tellus **28**, 427-433 (1976).

634. Kawamura, H. *et al*. Sulfur isotope variations in atmospheric sulfur oxides, particulate matter and deposits collected at Kyushu Island, Japan. *Water. Air. Soil Pollut.* **130**, 1775-1780 (2001).
635. Lee, G. *et al.* Sulfur isotope-based source apportionment and control mechanisms of $PM_{2.5}$ sulfate in Seoul, South Korea during winter and early spring (2017-2020). *Sci. Total Environ.* **905**, 167112 (2023).
636. Hong, Y. *et al.* Sulfur isotopic characteristics of coal in China and sulfur isotopic fractionation during coal-burning process. *Chin. J. Geochem.* **12**, 51-59 (1993).
637. Zhang, M. *et al.* Sulfur isotopic composition and source identification of atmospheric environment in central Zhejiang, China. *Sci. China Earth Sci.* **53**, 1717-1725 (2010).
638. Chen, S. *et al.* Sulfur isotopic fractionation and its implication: Sulfate formation in $PM_{2.5}$ and coal combustion under different conditions. *Atmospheric Res.* **194**, 142-149 (2017).
639. Guo, Z. *et al.* Sulfur isotopic fractionation and source appointment of $PM_{2.5}$ in Nanjing region around the second session of the Youth Olympic Games. *Atmospheric Res.* **174-175**, 9-17 (2016).
640. Ohizumi, T. *et al.* Sulfur isotopic view on the sources of sulfur in atmospheric fallout along the coast of the Sea of Japan. *Atmos. Environ.* **31**, 1339-1348 (1997).
641. Akata, N. *et al.* Ten-year observation of sulfur isotopic composition of sulfate in aerosols collected at Tsuruoka, a coastal area on the Sea of Japan in northern Japan. *Geochem. J.* **44**, 571-577 (2010).
642. Xiao, H. Y. & Liu, C. Q. The elemental and isotopic composition of sulfur and nitrogen in Chinese coals. *Org. Geochem.* **42**, 84-93 (2011).
643. Saltzman, E. S. *et al.* The mechanism of sulfate aerosol formation: Chemical and sulfur isotopic evidence. *Geophys. Res. Lett.* **10**, 513-516 (1983).
644. Rees, C. E. *et al.* The sulphur isotopic composition of ocean water sulphate. *Geochim. Cosmochim. Acta* **42**, 377-381 (1978).
645. Xiao, H. Y. *et al.* Tracing sources of coal combustion using stable sulfur isotope ratios in epilithic mosses and coals from China. *J. Environ. Monit.* **13**, 2243-2249 (2011).
646. Guo, Q. *et al.* Tracing the sources of sulfur in Beijing soils with stable sulfur isotopes. *J. Geochem. Explor.* **161**, 112-118 (2016).
647. Inomata, Y. *et al.* Transboundary transport of anthropogenic sulfur in $PM_{2.5}$ at a coastal site in the Sea of Japan as studied by sulfur isotopic ratio measurement. *Sci. Total Environ.* **553**, 617-625 (2016).
648. Ding, S. *et al.* Using stable sulfur isotope to trace sulfur oxidation pathways during the winter of 2017-2019 in Tianjin, North China. *Int. J. Environ. Res. Public. Health* **19**, 10966 (2022).
649. Mukai, H. *et al.* Regional characteristics of sulfur and lead isotope ratios in the atmosphere at several Chinese urban sites. *Environ. Sci. Technol.* **35**, 1064-1071 (2001).
650. Zuo, P. *et al.* New insights into unexpected severe $PM_{2.5}$ pollution during the SARS and COVID-19 pandemic periods in Beijing. *Environ. Sci. Technol.* **56**, 155-164 (2022).
651. Takano, S. *et al.* Sources of particulate Ni and Cu in the water column of the northern South China Sea: Evidence from elemental and isotope ratios in aerosols and sinking particles. *Mar. Chem.* **219**, 103751 (2020).
652. Šillerová, H. *et al.* Stable isotope tracing of Ni and Cu pollution in North-East Norway: Potentials and drawbacks. *Environ. Pollut.* **228**, 149-157 (2017).
653. Souto-Oliveira, C. E. *et al.* Multi-isotope approach of Pb, Cu and Zn in urban aerosols and anthropogenic sources improves tracing of the atmospheric pollutant sources in megacities. *Atmos. Environ.* **198**, 427-437 (2019).
654. Schleicher, N. J. *et al.* A global assessment of copper, zinc, and lead isotopes in mineral dust sources and aerosols. *Front. Earth Sci.* **8**, (2020).
655. Jeong, H. *et al.* Characteristics of potentially toxic elements and multi-isotope signatures (Cu, Zn, Pb) in non-exhaust traffic emission sources. *Environ. Pollut.* **292**, 118339 (2022).
656. Dong, S. *et al.* Isotopic signatures suggest important contributions from recycled gasoline, road dust and non-exhaust traffic sources for copper, zinc and lead in PM_{10} in London, United Kingdom. *Atmos. Environ.* **165**, 88-98 (2017).
657. Jeong, H. & Ra, K. Multi-isotope signatures (Cu, Zn, Pb) of different particle sizes in road-deposited sediments: A case study from industrial area. *J. Anal. Sci. Technol.* **12**, 39 (2021).
658. Souto-Oliveira, C. E. *et al.* Multi-isotopic fingerprints (Pb, Zn, Cu) applied for urban aerosol source apportionment and discrimination. *Sci. Total Environ.* **626**, 1350-1366 (2018).
659. Gonzalez, R. O. *et al.* New insights from zinc and copper isotopic compositions into the sources of atmospheric particulate matter from two major European cities. *Environ. Sci. Technol.* **50**, 9816-9824 (2016).
660. Bigalke, M. *et al.* Stable Cu and Zn isotope ratios as tracers of sources and transport of Cu and Zn in

contaminated soil. *Geochim. Cosmochim. Acta* **74**, 6801-6813 (2010).
661. Dong, S. *et al.* Stable isotope ratio measurements of Cu and Zn in mineral dust (bulk and size fractions) from the Taklimakan Desert and the Sahel and in aerosols from the eastern tropical North Atlantic Ocean. *Talanta* **114**, 103-109 (2013).
662. Packman, H. *et al.* Tracing natural and anthropogenic sources of aerosols to the Atlantic Ocean using Zn and Cu isotopes. *Chem. Geol.* **610**, 121091 (2022).
663. Li, F. B. & Li, Y, Z. Biogeochemical process of iron and its isotope fractionation mechanism in paddy field system: a review. *Ecol. Environ. Sci.* **28**, 1251-1260 (2019). In Chinese with English abstract
664. Li, R. *et al.* Iron isotopic compositions of combustion source particles and mineral dust. *Environ. Chem.* **40**, 990-998 (2021). In Chinese with English abstract
665. Kurisu, M. *et al.* Contribution of combustion Fe in marine aerosols over the northwestern Pacific estimated by Fe stable isotope ratios. *Atmospheric Chem. Phys.* **21**, 16027-16050 (2021).
666. Majestic, B. J. *et al.* Elemental and iron isotopic composition of aerosols collected in a parking structure. *Sci. Total Environ.* **407**, 5104-5109 (2009).
667. Guelke, M. & von Blanckenburg, F. Fractionation of stable iron isotopes in higher plants. *Environ. Sci. Technol.* **41**, 1896-1901 (2007).
668.Waeles, M. *et al.* Global dust teleconnections: Aerosol iron solubility and stable isotope composition. *Environ. Chem.* **4**, 233-237 (2007).
669. Flament, P. *et al.* Iron isotopic fractionation in industrial emissions and urban aerosols. *Chemosphere* **73**, 1793-1798 (2008).
670. Mead, C. *et al*. Source apportionment of aerosol iron in the marine environment using iron isotope analysis. *Geophys. Res. Lett.* **40**, 5722-5727 (2013).
671. Zuo, P. *et al.* Stable iron isotopic signature reveals multiple sources of magnetic particulate matter in the 2021 Beijing sandstorms. *Environ. Sci. Technol. Lett.* **9**, 299-305 (2022).
672. Kurisu, M. *et al.* Stable isotope ratios of combustion iron produced by evaporation in a steel plant. *ACS Earth Space Chem.* **3**, 588-598 (2019).
673. Majestic, B. J. *et al.* Stable isotopes as a tool to apportion atmospheric iron. *Environ. Sci. Technol.* **43**, 4327-4333 (2009).
674. Kurisu, M. & Takahashi, Y. Testing Iron stable isotope ratios as a signature of biomass burning. *Atmosphere* **10**, 76 (2019).
675. Akerman, A. *et al.* The isotopic fingerprint of Fe cycling in an equatorial soil-plant-water system: The Nsimi watershed, South Cameroon. *Chem. Geol.* **385**, 104-116 (2014).
676. Conway, T. M. *et al.* Tracing and constraining anthropogenic aerosol iron fluxes to the North Atlantic Ocean using iron isotopes. *Nat. Commun.* **10**, 2628 (2019).
677. Kurisu, M. *et al.* Variation of iron isotope ratios in anthropogenic materials emitted through combustion processes. *Chem. Lett.* **45**, 970-972 (2016).
678. Kurisu, M. *et al*. Very low isotope ratio of iron in fine aerosols related to its contribution to the surface ocean. *J. Geophys. Res. Atmospheres* **121**, 11,119-11,136 (2016).
679. Lu, D. *et al.* Natural silicon isotopic signatures reveal the sources of airborne fine particulate matter. *Environ. Sci. Technol.* **52**, 1088-1095 (2018).
680. Yang, X. *et al.* Two-dimensional silicon fingerprints reveal dramatic variations in the sources of particulate matter in Beijing during 2013-2017. *Environ. Sci. Technol.* **54**, 7126-7135 (2020).
681. Lu, D. *et al.* Unraveling the role of silicon in atmospheric aerosol secondary formation: a new conservative tracer for aerosol chemistry. *Atmospheric Chem. Phys.* **19**, 2861-2870 (2019).
682. Zhang, X. *et al.* Tracing anthropogenic aerosol trace metal sources in the North Atlantic Ocean using Pb, Zn and Ni isotopes. *Mar. Chem.* **258**, 104347 (2024).
683. Cloquet, C *et al*. Isotopic composition of Zn and Pb atmospheric depositions in an urban/periurban area of Northeastern France. *Environ. Sci. Technol.* **40**, 6594-6600 (2006).
684. Liang, L. L. *et al.* Chromatographic separation of Cu and Zn from environmental samples for determination of isotopic composition by MC-ICP-MS. *Earth Sci. Front.* **17**, 262-269 (2010). In Chinese with English abstract
685. Chen, M. & Zhu, J. M. Progress in research on Zn isotopes in plant. *Earth Environ.* **42**, 567-573 (2014). In Chinese with English abstract
686. Gioia, S. *et al.* Accurate and precise zinc isotope ratio measurements in urban aerosols. *Anal. Chem.* **80**, 9776-9780 (2008).
687. Schleicher, N. J. & Weiss, D. J. Identification of atmospheric particulate matter derived from coal and

biomass burning and from non-exhaust traffic emissions using zinc isotope signatures. *Environ. Pollut.* **329**, 121664 (2023).
688. Liu, X. *et al.* Soil (microbial) disturbance affect the zinc isotope biogeochemistry but has little effect on plant zinc uptake. *Sci. Total Environ.* **875**, 162490 (2023).
689. Natori, S. *et al.* Speciation and isotopic analysis of zinc in size-fractionated aerosol samples related to its source and chemical processes. *Atmos. Environ.* **294**, 119504 (2023).
690. Wiggenhauser, M. *et al.* Zinc isotope fractionation during grain filling of wheat and a comparison of zinc and cadmium isotope ratios in identical soil-plant systems. *New Phytol.* **219**, 195-205 (2018).
691. Ochoa Gonzalez, R. & Weiss, D. Zinc isotope variability in three coal-fired power plants: A predictive model for determining isotopic fractionation during combustion. *Environ. Sci. Technol.* **49**, 12560-12567 (2015).
692. Borrok, D. M. *et al.* Zinc isotopic composition of particulate matter generated during the combustion of coal and coal + tire-derived fuels. *Environ. Sci. Technol.* **44**, 9219-9224 (2010).
693. Mattielli, N. *et al.* Zn isotope study of atmospheric emissions and dry depositions within a 5 km radius of a Pb-Zn refinery. *Atmos. Environ.* **43**, 1265-1272 (2009).
694. Khondoker, R. *et al.* New constraints on elemental and Pb and Nd isotope compositions of South American and Southern African aerosol sources to the South Atlantic Ocean. *Geochemistry* **78**, 372-384 (2018).
695. Kumar, A. *et al.* A radiogenic isotope tracer study of transatlantic dust transport from Africa to the Caribbean. *Atmos. Environ.* **82**, 130-143 (2014).
696. Guéguen, F. *et al.* Atmospheric pollution in an urban environment by tree bark biomonitoring - Part II: Sr, Nd and Pb isotopic tracing. *Chemosphere* **86**, 641-647 (2012).
697. Lahd Geagea, M. *et al.* Baseline determination of the atmospheric Pb, Sr and Nd isotopic compositions in the Rhine valley, Vosges mountains (France) and the Central Swiss Alps. *Appl. Geochem.* **23**, 1703-1714 (2008).
698. Guéguen, F. *et al.* Chemical and isotopic properties and origin of coarse airborne particles collected by passive samplers in industrial, urban, and rural environments. *Atmos. Environ.* **62**, 631-645 (2012).
699. Bozlaker, A. *et al.* Linking Barbados mineral dust aerosols to North African sources using elemental composition and radiogenic Sr, Nd, and Pb isotope signatures. *J. Geophys. Res. Atmospheres* **123**, 1384-1400 (2018).
700. Hoàng-Hòa, T. B. *et al.* Pb, Sr and Nd isotopic composition and trace element characteristics of coarse airborne particles collected with passive samplers. *CR. Geosci.* **347**, 267-276 (2015).
701. Wang, S. *et al.* Source apportionment of metal elements in $PM_{2.5}$ in a coastal city in Southeast China: Combined Pb-Sr-Nd isotopes with PMF method. *Atmos. Environ.* **198**, 302-312 (2019).
702. Lahd Geagea, M. *et al.* Tracing of industrial aerosol sources in an urban environment using Pb, Sr, and Nd isotopes. *Environ. Sci. Technol.* **42**, 692-698 (2008).
703. Zhu, L. M. *et al.* A study on lead isotope geochemistry of atmospheric aerosol of Chukchi and Bering sea in Arctic. *Acta Scient. Circumst.* 846-851 (2004). In Chinese with English abstract
704. Hu, Y. *et al.* Lead and its isotope characteristics in atmospheric $PM_{2.5}$ in Pudong New Area, Shanghai. *J. Environ. Occup. Med.* **35**, 892-897 (2018). In Chinese with English abstract
705. Chen, J. *et al.* A lead isotope record of Shanghai atmospheric lead emissions in total suspended particles during the period of phasing out of leaded gasoline. *Atmos. Environ.* **39**, 1245-1253 (2005).
706. Bi, X. *et al.* Allocation and source attribution of lead and cadmium in maize (*Zea mays* L.) impacted by smelting emissions. *Environ. Pollut.* **157**, 834-839 (2009).
707. Gioia, S. M. C. L. *et al.* An isotopic study of atmospheric lead in a megacity after phasing out of leaded gasoline. *Atmos. Environ.* **149**, 70-83 (2017).
708. Zhao, Z. Q. *et al.* Atmospheric lead in urban Guiyang, Southwest China: Isotopic source signatures. *Atmos. Environ.* **115**, 163-169 (2015).
709. Tao, Z. *et al.* Atmospheric lead pollution in a typical megacity: Evidence from lead isotopes. *Sci. Total Environ.* **778**, 145810 (2021).
710. Chiaradia, M. & Cupelin, F. Behaviour of airborne lead and temporal variations of its source effects in Geneva (Switzerland): comparison of anthropogenic versus natural processes. *Atmos. Environ.* **34**, 959-971 (2000).
711. Bagur, M. & Widory, D. Characterising the levels and sources of the historical metal contamination in the atmosphere of Montreal (Canada) from 1973 to 2013 by coupling chemistry and Lead and Osmium isotope ratios. *Atmospheric Res.* **235**, 104794 (2020).
712. Zheng, J. *et al.* Characteristics of lead isotope ratios and elemental concentrations in PM_{10} fraction of airborne particulate matter in Shanghai after the phase-out of leaded gasoline. *Atmos. Environ.* **38**, 1191-1200 (2004).

713. Chen, J. *et al.* Characteristics of trace elements and lead isotope ratios in $PM_{2.5}$ from four sites in Shanghai. *J. Hazard. Mater.* **156**, 36-43 (2008).
714. Lee, S. *et al.* Characterization of trace elements and Pb isotopes in $PM_{2.5}$ and isotopic source identification during haze episodes in Seoul, Korea. *Atmospheric Pollut. Res.* **13**, 101442 (2022).
715. Gulson, B. *et al.* Comparison of lead isotopes with source apportionment models, including SOM, for air particulates. *Sci. Total Environ.* **381**, 169-179 (2007).
716. Tan, M. G. *et al.* Comprehensive Study of Lead Pollution in Shanghai by Multiple Techniques. *Anal. Chem.* **78**, 8044-8050 (2006).
717. Wang, J. *et al.* Contamination, morphological status and sources of atmospheric dust in different land-using areas of a steel-industry city, China. *Atmospheric Pollut. Res.* **11**, 283-289 (2020).
718. Chien, C. T. *et al.* Contributions of atmospheric deposition to Pb concentration and isotopic composition in seawater and particulate matters in the Gulf of Aqaba, Red Sea. *Environ. Sci. Technol.* **53**, 6162-6170 (2019).
719. Shen, Y. W. *et al.* Decryption analysis of antimony pollution sources in $PM_{2.5}$ through a multi-source isotope mixing model based on lead isotopes. *Environ. Pollut.* **328**, 121600 (2023).
720. Dewan, N. *et al.* Effect of pollution controls on atmospheric $PM_{2.5}$ composition during universiade in Shenzhen, China. *Atmosphere* **7**, 57 (2016).
721. Sakata, M., Kurata, M. & Tanaka, N. Estimating contribution from municipal solid waste incineration to trace metal concentrations in Japanese urban atmosphere using lead as a marker element. *Geochem. J.* **34**, 23-32 (2000).
722. Flament, P. *et al*. European isotopic signatures for lead in atmospheric aerosols: a source apportionment based upon $^{206}Pb/^{207}Pb$ ratios. *Sci. Total Environ.* **296**, 35-57 (2002).
723. Noble, S. R. *et al.* Evolving Pb isotope signatures of London airborne particulate matter (PM_{10}) - constraints from on-filter and solution-mode MC-ICP-MS. *J. Environ. Monit.* **10**, 830-836 (2008).
724. Chen, H. *et al*. High contribution of vehicular exhaust and coal combustion to $PM_{2.5}$ - bound Pb pollution in an industrial city in North China: An insight from isotope. *Atmos. Environ.* **294**, 119503 (2023).
725. Dai, W. J. *et al.* Identification and contribution of potential sources to atmospheric lead pollution in a typical megacity: Insights from isotope analysis and the Bayesian mixing model. *Sci. Total Environ.* **892**, 164567 (2023).
726. Ziegler, D. *et al.* Influence of start-up phase of an incinerator on inorganic composition and lead isotope ratios of the atmospheric PM_{10}. *Chemosphere* **266**, 129091 (2021).
727. Gioia, S. M. C. L. *et al.* Insights into the dynamics and sources of atmospheric lead and particulate matter in São Paulo, Brazil, from high temporal resolution sampling. *Atmospheric Res.* **98**, 478-485 (2010).
728. Notten, M. J. M. *et al.* Investigating the origin of Pb pollution in a terrestrial soil-plant-snail food chain by means of Pb isotope ratios. *Appl. Geochem.* **23**, 1581-1593 (2008).
729. Véron, A. *et al.* Isotopic evidence of pollutant lead sources in Northwestern France. *Atmos. Environ.* **33**, 3377-3388 (1999).
730. Zhu, L. *et al.* Lead concentrations and isotopes in aerosols from Xiamen, China. *Mar. Pollut. Bull.* **60**, 1946-1955 (2010).
731. Hu, X. *et al.* Lead contamination and transfer in urban environmental compartments analyzed by lead levels and isotopic compositions. *Environ. Pollut.* **187**, 42-48 (2014).
732. Liang, F. *et al.* Lead in children's blood is mainly caused by coal-fired ash after phasing out of leaded gasoline in Shanghai. *Environ. Sci. Technol.* **44**, 4760-4765 (2010).
733. Mukai, H. *et al.* Lead isotope ratios in the urban air of eastern and central Russia. *Atmos. Environ.* **35**, 2783-2793 (2001).
734. Widory, D. Lead isotopes decipher multiple origins within single PM_{10} samples in the atmosphere of Paris. *Isotopes Environ. Health Stud.* **42**, 97-105 (2006).
735. Bi, X. Y. *et al.* Lead isotopic compositions of selected coals, Pb/Zn ores and fuels in China and the application for source tracing. *Environ. Sci. Technol.* **51**, 13502-13508 (2017).
736. Sen, I. S. *et al.* Lead isotopic fingerprinting of aerosols to characterize the sources of atmospheric lead in an industrial city of India. *Atmos. Environ.* **129**, 27-33 (2016).
737. Kayee, J. *et al.* Metal concentrations and source apportionment of $PM_{2.5}$ in Chiang Rai and Bangkok, Thailand during a biomass burning season. *ACS Earth Space Chem.* **4**, 1213-1226 (2020).
738. Yu, Y. *et al.* Metal enrichment and lead isotope analysis for source apportionment in the urban dust and rural surface soil. *Environ. Pollut.* **216**, 764-772 (2016).
739. Kylander, M. E. *et al.* Natural lead isotope variations in the atmosphere. *Earth Planet. Sci. Lett.* **290**, 44-53 (2010).

740. Chifflet, S. *et al.* Origins and discrimination between local and regional atmospheric pollution in Haiphong (Vietnam), based on metal (loid) concentrations and lead isotopic ratios in PM_{10}. *Environ. Sci. Pollut. Res.* **25**, 26653-26668 (2018).
741. Bollhöfer, A. *et al.* Sampling aerosols for lead isotopes on a global scale. *Anal. Chim. Acta* **390**, 227-235 (1999).
742. Lee, P. K. *et al.* Seasonal variation in trace element concentrations and Pb isotopic composition of airborne particulates during Asian dust and non-Asian dust periods in Daejeon, Korea. *Environ. Earth Sci.* **74**, 3613-3628 (2015).
743. Ray, I. *et al.* Seasonal variation of atmospheric Pb sources in Singapore - Elemental and lead isotopic compositions of PM_{10} as source tracer. *Chemosphere* **307**, 136029 (2022).
744. Feng, J. *et al.* Source attributions of heavy metals in rice plant along highway in Eastern China. *J. Environ. Sci.* **23**, 1158-1164 (2011).
745. Kayee, J. *et al.* Sources of atmospheric lead (Pb) after quarter century of phasing out of leaded gasoline in Bangkok, Thailand. *Atmos. Environ.* **253**, 118355 (2021).
746. Das, R. *et al.* Sources of atmospheric lead (Pb) in and around an Indian megacity. *Atmos. Environ.* **193**, 57-65 (2018).
747. Kousehlar, M. & Widom, E. Sources of metals in atmospheric particulate matter in Tehran, Iran: Tree bark biomonitoring. *Appl. Geochem.* **104**, 71-82 (2019).
748. Cai, K. *et al.* Spatial Distribution, pollution source, and health risk assessment of heavy metals in atmospheric depositions: A case study from the sustainable city of Shijiazhuang, China. *Atmosphere* **10**, 222 (2019).
749. Resongles, E. *et al.* Strong evidence for the continued contribution of lead deposited during the 20th century to the atmospheric environment in London of today. *Proc. Natl. Acad. Sci.* **118**, e2102791118 (2021).
750. Das, R. *et al.* Suspension of crustal materials from wildfire in Indonesia as revealed by Pb isotope analysis. *ACS Earth Space Chem.* **7**, 379-387 (2023).
751. Watmough, S. A. & Hutchinson, T. C. The quantification and distribution of pollution Pb at a woodland in rural south central Ontario, Canada. *Environ. Pollut.* **128**, 419-428 (2004).
752. Kumar, S. *et al.* Tracing dust transport from Middle-East over Delhi in March 2012 using metal and lead isotope composition. *Atmos. Environ.* **132**, 179-187 (2016).
753. Wu, P. C. & Huang, K. F. Tracing local sources and long-range transport of PM_{10} in central Taiwan by using chemical characteristics and Pb isotope ratios. *Sci. Rep.* **11**, 7593 (2021).
754. Kumar, S. *et al.* Understanding the influence of open-waste burning on urban aerosols using metal tracers and lead isotopic composition. *Aerosol Air Qual. Res.* **18**, 2433-2446 (2018).
755. Félix, O. I. *et al.* Use of lead isotopes to identify sources of metal and metalloid contaminants in atmospheric aerosol from mining operations. *Chemosphere* **122**, 219-226 (2015).
756. Véron, A. J. & Church, T. M. Use of stable lead isotopes and trace metals to characterize air mass sources into the eastern North Atlantic. *J. Geophys. Res. Atmospheres* **102**, 28049-28058 (1997).
757. Salcedo, D. *et al.* Using trace element content and lead isotopic composition to assess sources of PM in Tijuana, Mexico. *Atmos. Environ.* **132**, 171-178 (2016).
758. Martinková, E. *et al.* Winter-time pollution in Central European cities shifts the $^{208}Pb/^{207}Pb$ isotope ratio of atmospheric $PM_{2.5}$ to higher values: Implications for lead source apportionment. *Atmos. Environ.* **310**, 119941 (2023).
759. Beltrán, B. *et al.* Automated method for simultaneous lead and strontium isotopic analysis applied to rainwater samples and airborne particulate filters (PM_{10}). *Environ. Sci. Technol.* **47**, 9850-9857 (2013).
760. Widory, D. *et al.* Isotopes as tracers of sources of lead and strontium in aerosols (TSP & $PM_{2.5}$) in Beijing. *Atmos. Environ.* **44**, 3679-3687 (2010).
761. Zhao, L. *et al.* Source apportionment of heavy metals in urban road dust in a continental city of eastern China: Using Pb and Sr isotopes combined with multivariate statistical analysis. *Atmos. Environ.* **201**, 201-211 (2019).
762. Dewan, N. *et al.* Stable isotopes of lead and strontium as tracers of sources of airborne particulate matter in Kyrgyzstan. *Atmos. Environ.* **120**, 438-446 (2015).
763. Das, S. *et al.* Coupling Sr-Nd-Hf isotope ratios and elemental analysis to accurately quantify North African dust contributions to $PM_{2.5}$ in a complex urban atmosphere by reducing mineral dust collinearity. *Environ. Sci. Technol.* **56**, 7729-7740 (2022).
764. Das, S. *et al.* Sr-Nd-Hf isotopic analysis of reference materials and natural and anthropogenic particulate matter sources: Implications for accurately tracing North African dust in complex urban atmospheres. *Talanta* **241**, 123236 (2022).

765. Aarons, S. M. *et al.* Variable Hf Sr Nd radiogenic isotopic compositions in a Saharan dust storm over the Atlantic: Implications for dust flux to oceans, ice sheets and the terrestrial biosphere. *Chem. Geol.* **349-350**, 18-26 (2013).
766. Yan, Y. *et al.* Characteristics and provenance implications of rare earth elements and Sr-Nd isotopes in $PM_{2.5}$ aerosols and $PM_{2.5}$ fugitive dusts from an inland city of southeastern China. *Atmos. Environ.* **220**, 117069 (2020).
767. Wang, S. *et al.* Influence of pollution reduction interventions on atmospheric $PM_{2.5}$: A case study from the 2017 Xiamen. *Atmospheric Pollut. Res.* **12**, 101137 (2021).
768. Bikkina, S. *et al.* Link of the short-term temporal trends of Sr and Nd isotopic composition of aeolian dust over the Arabian Sea with the source emissions. *Sci. Total Environ.* **892**, 164680 (2023).
769. van der Does, M. *et al.* North African mineral dust across the tropical Atlantic Ocean: Insights from dust particle size, radiogenic Sr-Nd-Hf isotopes and rare earth elements (REE). *Aeolian Res.* **33**, 106-116 (2018).
770. Nakano, T. *et al.* Source and evolution of the "perfect Asian dust storm" in early April 2001: Implications of the Sr-Nd isotope ratios. *Atmos. Environ.* **39**, 5568-5575 (2005).
771. Li, J. *et al.* Sr-Nd elements and isotopes as tracers of dust input in a tropical soil chronosequence. *Geoderma* **262**, 227-234 (2016).
772. Wong, M. *et al.* A bioavailable baseline strontium isotope map of southwestern Turkey for mobility studies. *J. Archaeol. Sci. Rep.* **37**, 102922 (2021).
773. Jung, C. C. *et al.* C-Sr-Pb isotopic characteristics of $PM_{2.5}$ transported on the East-Asian continental outflows. *Atmospheric Res.* **223**, 88-97 (2019).
774. Flockhart, D. T. T. *et al.* Experimental evidence shows no fractionation of strontium isotopes ($^{87}Sr/^{86}Sr$) among soil, plants, and herbivores: implications for tracking wildlife and forensic science. *Isotopes Environ. Health Stud.* **51**, 372-381 (2015).
775. Han, G. & Eisenhauer, A. Stable and radiogenic strontium isotope cycling in a representative karst forest ecosystem, Southwest China. *Environ. Earth Sci.* **80**, 741 (2021).
776. Choi, H. B. *et al.* Stable strontium isotope fractionation in hydroponically grown mung and soy bean sprouts. *J. Food Compos. Anal.* **116**, 105081 (2023).
777. Bertinetti, S. *et al.* Strontium isotopic analysis of environmental microsamples by inductively coupled plasma - tandem mass spectrometry. *J. Anal. At. Spectrom.* **37**, 103-113 (2022).
778. Grotti, M. Strontium isotopic fingerprinting of atmospheric particulate matter by quadrupole inductively coupled plasma mass spectrometry. *At. Spectrosc.* **44**, 311-317 (2023).
779. Price, T. D. *et al.* The characterization of biologically available strontium isotope ratios for the study of prehistoric migration. *Archaeometry* **44**, 117-135 (2002).
780. Kanayama, S. *et al.* The chemical and strontium isotope composition of atmospheric aerosols over Japan: the contribution of long-range-transported Asian dust (Kosa). *Atmos. Environ.* **36**, 5159-5175 (2002).
781. Marcy, M. J. *et al.* Trace element chemistry and strontium isotope ratios of atmospheric particulate matter reveal air quality impacts from mineral dust, urban pollution, and fireworks in the Wasatch Front, Utah, USA. *Appl. Geochem.* **162**, 105906 (2024).
782. Duarte, R. M. B. O. *et al.* Tracing of aerosol sources in an urban environment using chemical, Sr isotope, and mineralogical characterization. *Environ. Sci. Pollut. Res.* **24**, 11006-11016 (2017).
783. Grousset, F. E. *et al.* Neodymium isotopes as tracers in marine sediments and aerosols: North Atlantic. *Earth Planet. Sci. Lett.* **87**, 367-378 (1988).
784. Amr, M. A. *et al.* Rare earth elements and $^{143}Nd/^{144}Nd$ isotope ratio measurements using tandem ICP-CRC-MS/MS: characterization of date palm (Phoenix dactylifera L.). *J. Anal. At. Spectrom.* **32**, 1554-1565 (2017).
785. Hu, G. *et al.* Source apportionment of rare earth elements in $PM_{2.5}$ in a Southeast coastal city of China. *Aerosol Air Qual. Res.* **19**, 92-102 (2019).
786. Huang, Q. *et al.* An improved dual-stage protocol to pre-concentrate mercury from airborne particles for precise isotopic measurement. *J. Anal. At. Spectrom.* **30**, 957-966 (2015).
787. Guo, J. *et al.* Atmospheric particle-bound mercury in the northern Indo-Gangetic Plain region: Insights into sources from mercury isotope analysis and influencing factors. *Geosci. Front.* **13**, 101274 (2022).
788. Huang, Q. *et al.* Diel variation in mercury stable isotope ratios records photoreduction of $PM_{2.5}$-bound mercury. *Atmospheric Chem. Phys.* **19**, 315-325 (2019).
789. Fu, X. *et al.* Domestic and Transboundary Sources of Atmospheric Particulate Bound Mercury in Remote Areas of China: Evidence from Mercury Isotopes. *Environ. Sci. Technol.* **53**, 1947-1957 (2019).
790. Qiu, Y. *et al.* Identification of potential sources of elevated $PM_{2.5}$-Hg using mercury isotopes during haze events. *Atmos. Environ.* **247**, 118203 (2021).

791. Huang, Q. *et al.* Isotopic composition for source identification of mercury in atmospheric fine particles. *Atmospheric Chem. Phys.* **16**, 11773-11786 (2016).
792. Yu, B. *et al.* Isotopic composition of atmospheric mercury in China: New evidence for sources and transformation processes in air and in vegetation. *Environ. Sci. Technol.* **50**, 9262-9269 (2016).
793. Rolison, J. M. *et al.* Isotopic composition of species-specific atmospheric Hg in a coastal environment. *Chem. Geol.* **336**, 37-49 (2013).
794. Sun, L. *et al.* Mercury concentration and isotopic composition on different atmospheric particles (PM_{10} and $PM_{2.5}$) in the subtropical coastal suburb of Xiamen Bay, Southern China. *Atmos. Environ.* **261**, 118604 (2021).
795. Zambardi, T. *et al.* Mercury emissions and stable isotopic compositions at Vulcano Island (Italy). *Earth Planet. Sci. Lett.* **277**, 236-243 (2009).
796. Das, R. *et al.* Mercury isotopes of atmospheric particle bound mercury for source apportionment study in urban Kolkata, India. *Elem. Sci. Anthr.* **4**, 000098 (2016).
797. Xu, L. *et al.* Mercury isotopic compositions in fine particles and offshore surface seawater in a coastal area of East China: implications for Hg sources and atmospheric transformations. *Atmospheric Chem. Phys.* **21**, 18543-18555 (2021).
798. Liu, H. *et al.* Mercury isotopic compositions of mosses, conifer needles, and surface soils: Implications for mercury distribution and sources in Shergyla Mountain, Tibetan Plateau. *Ecotoxicol. Environ. Saf.* **172**, 225-231 (2019).
799. Xu, H. M. *et al.* Mercury stable isotope compositions of Chinese urban fine particulates in winter haze days: Implications for Hg sources and transformations. *Chem. Geol.* **504**, 267-275 (2019).
800. Zheng, W. *et al.* Mercury stable isotopes reveal the sources and transformations of atmospheric Hg in the high Arctic. *Appl. Geochem.* **131**, 105002 (2021).
801. Huang, S. *et al.* Natural stable isotopic compositions of mercury in aerosols and wet precipitations around a coal-fired power plant in Xiamen, southeast China. *Atmos. Environ.* **173**, 72-80 (2018).
802. Yu, B. *et al.* New evidence for atmospheric mercury transformations in the marine boundary layer from stable mercury isotopes. *Atmospheric Chem. Phys.* **20**, 9713-9723 (2020).
803. Qiu, Y. *et al.* Potential factors impacting $PM_{2.5}$-Hg during haze evolution revealed by mercury isotope: Emission sources and photochemical processes. *Atmospheric Res.* **277**, 106318 (2022).
804. Xu, H. *et al.* Seasonal and annual variations in atmospheric hg and pb isotopes in Xi'an, China. *Environ. Sci. Technol.* **51**, 3759-3766 (2017).
805. Li, C. *et al.* Seasonal variation of mercury and its isotopes in atmospheric particles at the coastal Zhongshan Station, Eastern Antarctica. *Environ. Sci. Technol.* **54**, 11344-11355 (2020).
806. AuYang, D. *et al.* Seasonal variations of the mercury multiple isotopic compositions of subrural and urban aerosols highlight an additional atmospheric Hg^0 oxidation pathway. *Front. Environ. Sci.* **9**, (2022).
807. Guo, J. *et al.* Source identification of atmospheric particle-bound mercury in the Himalayan foothills through non-isotopic and isotope analyses. *Environ. Pollut.* **286**, 117317 (2021).
808. Liu, C. *et al.* Sources and transformation mechanisms of atmospheric particulate bound mercury revealed by mercury stable isotopes. *Environ. Sci. Technol.* **56**, 5224-5233 (2022).
809. Zhang, K. *et al.* Stable isotopes reveal photoreduction of particle-bound mercury driven by water-soluble organic carbon during severe haze. *Environ. Sci. Technol.* **56**, 10619-10628 (2022).
810. Qin, X. *et al.* Tracing the transboundary transport of atmospheric particulate bound mercury driven by the East Asian monsoon. *J. Hazard. Mater.* **446**, 130678 (2023).
811. Huang, Q. *et al.* Variation in the mercury concentration and stable isotope composition of atmospheric total suspended particles in Beijing, China. *J. Hazard. Mater.* **383**, 121131 (2020).
812. Wei, T. *et al.* Hf-Nd-Sr isotopic composition of the Tibetan Plateau Dust as a fingerprint for regional to hemispherical transport. *Environ. Sci. Technol.* **55**, 10121-10132 (2021).
813. Chang, Y. *et al.* Source apportionment of atmospheric ammonia before, during, and after the 2014 APEC summit in Beijing using stable nitrogen isotope signatures. *Atmospheric Chem. Phys.* **16**, 11635-11647 (2016).
814. Berner, A. *et al.* Investigating ammonia emissions in a coastal urban airshed using stable isotope techniques. *Sci. Total Environ.* **707**, 134952 (2020).
815. Chang, Y. *et al.* Human excreta as a stable and important source of atmospheric ammonia in the megacity of Shanghai. *PLoS ONE.* **10**, e0144661 (2015).
816. Heaton, T. *et al.* $^{15}N/^{14}N$ ratios of nitrate and ammonium in rain at Pretoria, South Africa. *Atmos. Environ.* **21**, 843-852 (1987).
817. Yu, Z. *et al.* Nitrogen isotopic fractionations during nitric oxide production in an agricultural soil.

Biogeosciences. **18**, 805-829 (2021).
818. Sun, Z. *et al.* Characterization of the nitrogen stable isotope composition ($\delta^{15}N$) of ship-emitted NO_x. *Atmospheric Chem. Phys.* **23**, 12851–12865 (2023).
819. Hoering, T. *et al.* The isotopic composition of the ammonia and the nitrate ion in rain. *Geochim Cosmochim Ac.* **12**, 97-102 (1957).
820. Hoering, T. *et al.* $\delta^{15}N$ of nitric oxide produced under aerobic or anaerobic conditions from seven soils and their associated N isotope fractionations. *J. Geophys. Res. Biogeo.* **125**, e2020JG005705 (2020).
821. Liu, D. *et al.* Chemical Method for Nitrogen Isotopic Analysis of Ammonium at Natural Abundance. *Anal. Chem.* **86**, 3787-3792 (2014).
822. Zhang, L., Altabet, M. A., Wu, T. & Hadas, O. Sensitive Measurement of NH4+ 15N/14N (δ15NH4+) at Natural Abundance Levels in Fresh and Saltwaters. *Anal. Chem.* **79**, 5297-5303 (2007).
823. Sigman, D. M. et al. A Bacterial Method for the Nitrogen Isotopic Analysis of Nitrate in Seawater and Freshwater. *Anal. Chem.* **73**, 4145-4153 (2001)
